# Supplementary material for: Skeletal reorganization divergence of N-sulfonyl ynamides
Source: Nat Commun. 2020 Nov 6;11:5639. doi: 10.1038/s41467-020-19467-5 (PMC7648764; doi:10.1038/s41467-020-19467-5)
Supplement: Supplementary file 1 — Supplementary Information [file 41467_2020_19467_MOESM1_ESM.pdf]

Supplementary Information

Skeletal Reorganization Divergence of *N*-Sulfonyl Ynamides

Linwei Zeng[1], Yuxin Lin[1], Jiaming Li[1], Hironao Sajiki[2], Hujun Xie[3], & Sunliang Cui[1]*

[1] *Institute of Drug Discovery and Design, College of Pharmaceutical Sciences, Zhejiang University, Hangzhou 310058, China*
[2] *Laboratory of Organic Chemistry, Gifu Pharmaceutical University, Gifu 501-1196, Japan*
[3] *Department of Applied Chemistry, Zhejiang Gongshang University, Hangzhou 310018, China*

Email: slcui@zju.edu.cn

Contents

1	General Information	2
2	Starting Materials	3 – 19
3	Typical Procedure for the Synthesis of **2a**	20
4	Typical Procedure for the Synthesis of **3a**	20
5	Gram-Scale Reactions of **2a** and **3a**	21
6	Procedure for the Synthesis of **4**	22
7	Procedure for the Synthesis of **5**	22 – 23
8	Crossover Experiment	23
9	α-Methylation Experiment	24
10	^{13}C-Labelled Experiment	24 – 27
11	Characterization of Products	27 – 50
12	X-ray Crystallographic Data	51 – 57
13	DFT Calculations	58 – 59
14	Copies of NMR Spectra	60 – 316
15	Supplementary References	317 – 318

1. General Information

Reactions were monitored by thin layer chromatography (TLC) using silicycle pre-coated silica gel plates. Column chromatography was performed over silica gel (200–300mesh).

Melting points were measured with X–4 micro melting point apparatus.

Infrared spectra were obtained on a FT/IR spectrometer (College of Pharmaceutical Sciences, Zhejiang University).

HRMS were performed on Waters GCT premier time of flight mass spectrometer (EI-TOF) (Department of Chemistry, Zhejiang University) or Agilent Technologies 6546-LC/Q-TOF mass spectrometer (ESI-TOF) (Pharmaceutical Informatics Institute, Zhejiang University).

^{1}H NMR spectra and ^{13}C NMR spectra were recorded on a Bruker AV-600 spectrometer (College of Life Sciences, Zhejiang University), a Bruker AV-500 spectrometer (Pharmaceutical Informatics Institute, Zhejiang University) or a WNMR-I-400 spectrometer (Department of Chemistry, Zhejiang University) in chloroform-*d* ($CDCl_3$, contain internal TMS) or DMSO-d_6. For $CDCl_3$ as solvent, chemical shifts of ^{1}H NMR spectra were reported in ppm with the internal TMS signal at 0 ppm as a standard, and chemical shifts of ^{13}C NMR spectra were reported in ppm with the chloroform signal at 77.16 ppm as a standard. With respect to DMSO-d_6 as solvent, chemical shifts of ^{1}H NMR and ^{13}C NMR spectra were reported in ppm with the DMSO signal at 2.500 ppm and 39.52 ppm as the standard respectively.[1] The data is being reported as (s = singlet, d = doublet, t = triplet, q = quartet, quint = quintet, hept = heptet, dd = double doublet, dt = double of triplet, m = multiplet or unresolved, br = broad singlet, coupling constant(s) in Hz, integration).

Lithium diisopropylamide (LDA, 2 mol/L in THF) was purchased from Energy Chemical Technology Co., Ltd.

Solvents, such as Ethyl acetate (EA), petroleum ether (PE) Methanol (MeOH) were obtained commercially and used without further purification unless otherwise noted. Tetrahydrofuran (THF) was purified by distillation after treating with sodium. *N*,*N'*-Dimethylpropyleneurea (DMPU) was purified by distillation after treating with CaH_2.

2. Starting Material

2.1 Synthesis of Starting Ynamides

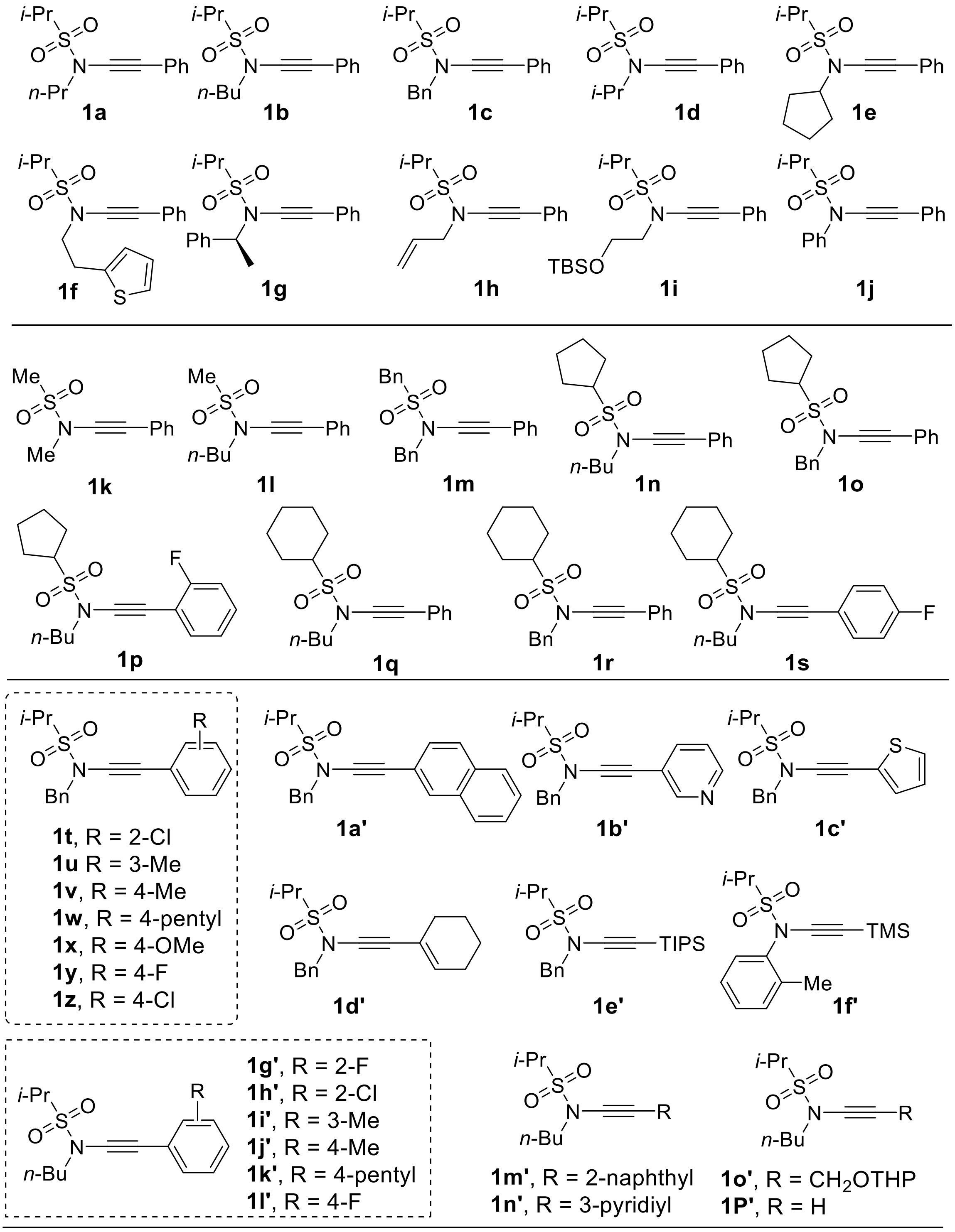

Supplementary Fig. 1 Starting materials. Chemical structues of all prepared starting ynamides.

All starting ynamides are listed in Supplementary Fig. 1. Ynamides **1a-1e'** and **1g'- 1o'** were prepared according to the procedure of reported literatures (Supplementary Fig. 2).[2] Ynamide **1f'** was

prepared according to the method of literature (Supplementary Fig. 3).[3]

Caution: All ynamides, especially those in oily and liquid form, are unstable. They should be used immediately after preparation and stored at -20 °C to slow down the decomposition.

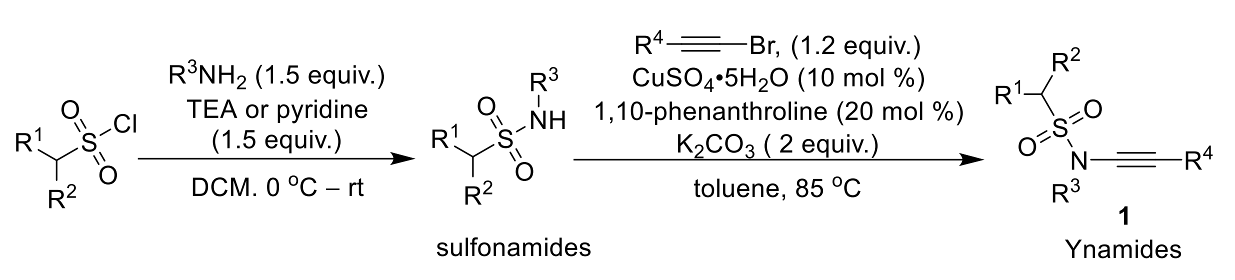

Supplementary Fig. 2 Synthesis of ynamides. Ynamides were prepared by coupling between sulfonamides and alkynyl bromides

Generatural procedure for the synthesis of sulfonamides:

A two-neck flask equipped with a magnetic stirrer bar was charged with corresponding amine (7.5 mmol) and purged with argon three times. Anhydrous DCM (20 mL) was added as solvent. Then base (7.5 mmol, for aliphatic amines, TEA was used; for aryl amines, pyridine was used) was added. The mixture was cooled to 0 °C and the sulfonyl chloride (5 mmol) was added dropwise by a syringe. The reaction was warmed to room temperature and stirred for 5h. Aqueous HCl (2 mol/L) was added and the mixture was extracted with DCM (2 x 20mL). The combined organic layer was washed by aqueous HCl (2 mol/L) and brine, dried over anhydrous Na_2SO_4, filtered and concentrated under vacuum to obtain the corresponding sulphonamides which were used directly in next step without further pruficiation. (Caution: *N*-methylmethanesulfonamide was obtained commercially.)

Generatural procedure for the synthesis of ynamides (1a-1e' and 1g'-1n') :

An oven-dried schlenk tube equipped with a magnetic stirrer bar was charged with corresponding sulfonamide (1 mmol), $CuSO_4$•$5H_2O$ (25 mg, 0.1 mmol, 10 mol %), 1,10- phenanthrolline (36 mg, 0.2 mmol, 20 mol %) and K_2CO_3 (277 mg, 2 mmol, 2 equiv.). After purged with argon three times, anhydrous toluene (1 mL) and alkynyl bromides (1.2 mmol, 1.2 equiv.) were added. The reaction was heated at 85 °C for 12 h and then filtered. The solid was washed by DCM and the filtrate was concentrated under vacuum to obtain the residue, which was further purified by silica gel column chromatography using ethyl acetate/petroleum ether as eluent to give corresponding ynamides.

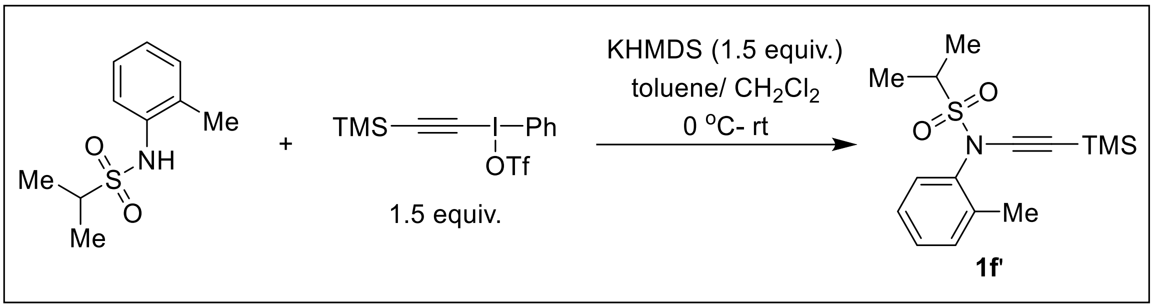

Supplementary Fig. 3 Synthesis of ynamide 1f'. Ynamide **1f'** was prepared by the reaction of sulfonamide and hypervalent iodine reagent.

Procedure for the synthesis of ynamide 1f':

An oven-dried schlenk tube equipped with a magnetic stirrer bar was charged with corresponding sulfonamide (213 mg, 1 mmol) and purged with argon three times. Anhydrous toluene (2 mL) was added and the reaction was cooled to 0 °C. KHMDS (1.5 mL, 1 mol/L in THF) was added slowly and the mixture was stirred for 30 mins. Then alkynyl hypervalent iodine (680 mg, 1.5 equiv) was suspended in DCM (2 mL) and added to the mixture. The reaction was warmed to room temperature and stirred for another 12 h. Water was added and the mixture was extracted with DCM (3x 10 mL). The combined organic layer was washed by brine, dried over anhydrous Na2SO4, filtered and concentrated under vacuum to obtain the residue, which was further purified by silica gel column chromatography using ethyl acetate/petroleum ether as eluent to give ynamide **1f'** (80 mg, 26% yield) as a yellow solid.

2.2 Characterization of New Ynamides

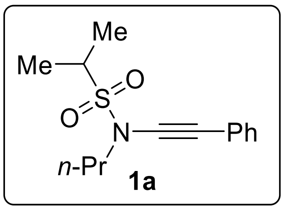

***N*-(phenylethynyl)-*N*-propylpropane-2-sulfonamide**

1a, Light brown oil (234 mg, 89% yield).

^{1}H NMR (400 MHz, $CDCl_3$): δ 7.41 – 7.35 (m, 2H), 7.32 – 7.24 (m, 3H), 3.61 (hept, *J* = 6.8 Hz, 1H), 3.51 (t, *J* = 7.2 Hz, 2H), 1.89 – 1.76 (m, 2H), 1.47 (d, *J* = 6.8 Hz, 6H), 1.02 (t, *J* = 7.2 Hz, 3H); ^{13}C

NMR (100 MHz, $CDCl_3$): δ 131.4, 128.3, 127.8, 123.0, 82.6, 70.2, 54.4, 54.1, 22.2, 16.7, 10.9; HRMS (EI-TOF) *m/z*: $[M]^+$ calcd. for $C_{14}H_{19}NO_2S$, 265.1136; found, 265.1133.

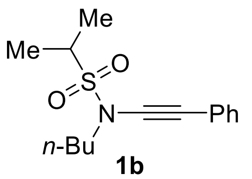

1b

***N*-butyl-*N*-(phenylethynyl)propane-2-sulfonamide**

1b, Light brown oil (234 mg, 84% yield).

^{1}H NMR (400 MHz, $CDCl_3$): δ 7.41 – 7.36 (m, 2H), 7.32 – 7.24 (m, 3H), 3.61 (hept, *J* = 6.8 Hz, 1H), 3.55 (t, *J* = 7.2 Hz, 2H), 1.82 – 1.74 (m, 2H), 1.50 – 1.39 (m, 8H), 0.97 (t, *J* = 7.2 Hz, 3H); ^{13}C NMR (100 MHz, $CDCl_3$): δ 131.4, 128.4, 127.8, 123.0, 82.6, 70.2, 54.3, 52.3, 30.9, 19.6, 16.7, 13.8; HRMS (EI-TOF) *m/z*: $[M]^+$ calcd. for $C_{15}H_{21}NO_2S$, 279.1293; found, 279.1296.

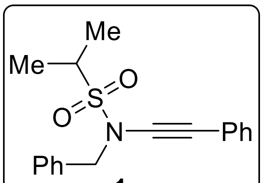

1c

***N*-benzyl-*N*-(phenylethynyl)propane-2-sulfonamide**

1c, White solid (284 mg, 91% yield).

^{1}H NMR (500 MHz, $CDCl_3$): δ 7.49 – 7.44 (m, 2H), 7.41 – 7.32 (m, 3H), 7.30 – 7.22 (m, 5H), 4.70 (s, 2H), 3.53 (hept, *J* = 7.0 Hz, 1H), 1.44 (d, *J* = 7.0 Hz, 6H); ^{13}C NMR (125 MHz, $CDCl_3$): δ 135.3, 131.3, 129.0, 128.8, 128.6, 128.3, 127.8, 122.9, 82.9, 70.9, 56.3, 54.9, 16.7; HRMS (EI-TOF) *m/z*: $[M]^+$ calcd. for $C_{18}H_{19}NO_2S$, 313.1136; found, 313.1135.

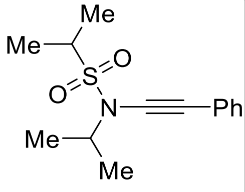

1d

***N*-isopropyl-*N*-(phenylethynyl)propane-2-sulfonamide**

1d, K_3PO_4 instead of K_2CO_3, yellow oil (148 mg, 56% yield).

^{1}H NMR (500 MHz, $CDCl_3$): δ 7.42 – 7.38 (m, 2H), 7.32 – 7.24 (m, 3H), 4.20 (hept, *J* = 7.0 Hz, 1H), 3.52 (hept, *J* = 7.0 Hz, 1H), 1.46 (d, *J* = 7.0 Hz, 6H), 1.38 (d, *J* = 7.0 Hz, 6H); ^{13}C NMR (125MHz, $CDCl_3$): δ 131.3, 128.4, 127.7, 123.3, 79.7, 72.3, 54.8, 52.8, 21.6, 16.7; HRMS (EI-TOF) *m/z*: $[M]^+$ calcd. for $C_{14}H_{19}NO_2S$, 265.1136; found, 265.1131.

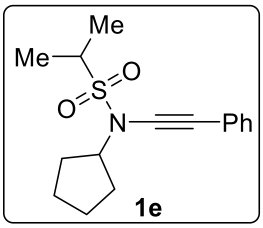

N-cyclopentyl-N-(phenylethynyl)propane-2-sulfonamide

1e, K_3PO_4 instead of K_2CO_3, yellow oil (215 mg, 74% yield).

^{1}H NMR (400 MHz, $CDCl_3$): δ 7.42 – 7.36 (m, 2H), 7.33 – 7.27 (m, 3H), 4.32 (quint, *J* = 7.6 Hz, 1H), 3.55 (hept, *J* = 6.8 Hz, 1H), 2.08 – 1.87 (m, 4H), 1.86 – 1.75 (m, 2H), 1.65 – 1.55 (m, 2H), 1.47 (d, *J* = 6.8 Hz, 6H); ^{13}C NMR (100MHz, $CDCl_3$): δ 131.3, 128.4, 127.8, 123.2, 80.4, 72.3, 61.4, 54.6, 31.1, 24.3, 16.8; HRMS (EI-TOF) *m/z*: $[M]^+$ calcd. for $C_{16}H_{21}NO_2S$, 291.1293; found, 291.1294.

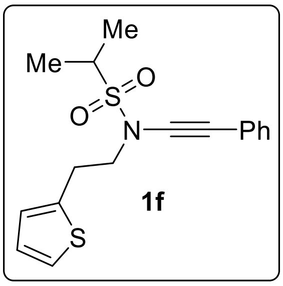

N-(phenylethynyl)-N-(2-(thiophen-2-yl)ethyl)propane-2-sulfonamide

1f, Yellow oil (239 mg, 72% yield).

^{1}H NMR (400 MHz, $CDCl_3$): δ 7.44 – 7.38 (m, 2H), 7.34 – 7.27 (m, 3H), 7.22 – 7.14 (m, 1H), 6.98 – 6.92 (m, 2H), 3.81 (t, *J* = 7.2 Hz, 2H), 3.44 (hept, *J* = 6.8 Hz, 1H), 3.33 (t, *J* = 7.2 Hz, 2H), 1.40 (d, *J* = 6.8 Hz, 6H); ^{13}C NMR (100 MHz, $CDCl_3$): δ 139.5, 131.6, 128.4, 128.1, 127.2, 126.2, 124.4, 122.8, 82.0, 70.9, 54.7, 53.5, 29.4, 16.6; HRMS (EI-TOF) *m/z*: $[M]^+$ calcd. for $C_{17}H_{19}NO_2S_2$, 333.0857; found, 333.0855.

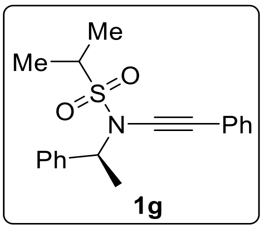

(*S*)-*N*-(1-phenylethyl)-*N*-(phenylethynyl)propane-2-sulfonamide

1g, Yellow oil (75 mg, 23% yield).

^{1}H NMR (400 MHz, CDCl$_3$): δ 7.53 – 7.49 (m, 2H), 7.42 – 7.27 (m, 8H), 5.20 (q, *J* = 7.2 Hz, 1H), 3.34 (hept, *J* = 6.8 Hz, 1H), 1.78 (d, *J* = 7.0 Hz, 3H), 1.37 (d, *J* = 6.8 Hz, 3H), 1.34 (d, *J* = 6.8 Hz, 3H); ^{13}C NMR (100MHz, CDCl$_3$): δ 140.4, 131.3, 128.8, 128.5, 128.4, 127.8, 127.1, 123.2, 80.7, 72.9, 58.9, 55.2, 20.3, 16.7, 16.6; HRMS (EI-TOF) *m/z*: [M]$^+$ calcd. for $C_{19}H_{21}NO_2S$, 327.1293; found, 327.1295.

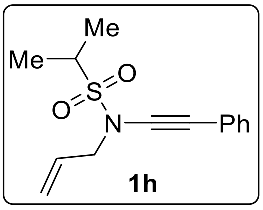

***N*-allyl-*N*-(phenylethynyl)propane-2-sulfonamide**

1h, Yellow oil (208 mg, 79% yield).

^{1}H NMR (600 MHz, CDCl$_3$): δ 7.42 – 7.34 (m, 2H), 7.32 – 7.23 (m, 3H), 6.05 – 5.92 (m, 1H), 5.41 (d, *J* = 17.4 Hz, 1H), 5.34 (d, *J* = 10.2 Hz, 1H), 4.15 (d, *J* = 6.6 Hz, 2H), 3.59 (hept, *J* = 6.6 Hz, 1H), 1.48 (d, *J* = 6.6 Hz, 6H); ^{13}C NMR (150 MHz, CDCl$_3$): δ 131.7, 131.4, 128.3, 127.9, 122.9, 120.2, 82.6, 70.4, 54.9, 54.8, 16.7; HRMS (EI-TOF) *m/z*: [M]$^+$ calcd. for $C_{14}H_{17}NO_2S$, 263.0980; found, 263.0983.

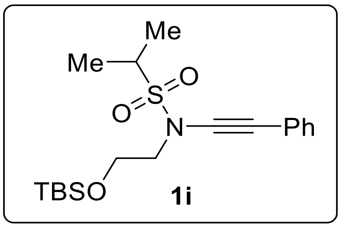

***N*-(2-((*tert*-butyldimethylsilyl)oxy)ethyl)-*N*-(phenylethynyl)propane-2-sulfonamide**

1i, Yellow oil (297 mg, 78% yield).

^{1}H NMR (400 MHz, CDCl$_3$): δ 7.40 – 7.35 (m, 2H), 7.32 – 7.26 (m, 3H), 3.93 (t, *J* = 5.6 Hz, 2H), 3.72 – 3.61 (m, 3H), 1.48 (d, *J* = 6.8 Hz, 6H), 0.91 (s, 9H), 0.10 (s, 6H); ^{13}C NMR (100 MHz, CDCl$_3$): δ 131.5, 128.4, 127.9, 123.1, 82.7, 70.0, 60.7, 54.9, 54.3, 26.0, 18.5, 16.8, -5.3; HRMS (EI-TOF) *m/z*: [M]$^+$ calcd. for $C_{19}H_{31}NO_3SSi$, 381.1794; found, 381.1795.

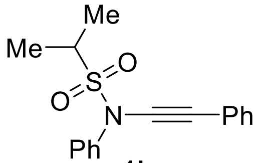

1j

***N*-phenyl-*N*-(phenylethynyl)propane-2-sulfonamide**

1j, Yellow solid (75 mg, 25% yield).

^{1}H NMR (400 MHz, $CDCl_3$): δ 7.65 – 7.59 (m, 2H), 7.46 – 7.38 (m, 4H), 7.35 – 7.26 (m, 4H), 3.69 (hept, J = 6.8 Hz, 1H), 1.50 (d, J = 6.8 Hz, 6H); ^{13}C NMR (100 MHz, $CDCl_3$): δ 139.3, 131.5, 129.5, 128.4, 128.1, 127.8, 125.1, 122.7, 83.0, 70.4, 54.5, 16.8; HRMS (EI-TOF) *m/z*: $[M]^+$ calcd. for $C_{17}H_{17}NO_2S$, 299.0980; found, 299.0985.

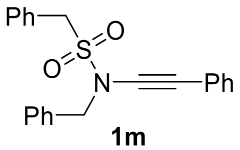

1m

***N*-benzyl-1-phenyl-*N*-(phenylethynyl)methanesulfonamide**

1m, White solid (317 mg, 88% yield).

^{1}H NMR (400 MHz, $CDCl_3$): δ 7.45 – 7.38 (m, 5H), 7.37 – 7.25 (m, 10H), 4.47 (s, 2H), 4.28 (s, 2H); ^{13}C NMR (100 MHz, $CDCl_3$): δ 134.9, 131.5, 131.1, 129.4, 129.1, 129.0, 128.8, 128.6, 128.4, 128.1, 127.8, 122.7, 82.1, 71.9, 57.9, 56.7; HRMS (EI-TOF) *m/z*: $[M]^+$ calcd. for $C_{22}H_{19}NO_2S$, 361.1136; found, 361.1135.

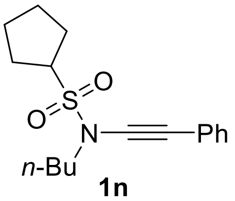

1n

***N*-butyl-*N*-(phenylethynyl)cyclopentanesulfonamide**

1n, Yellow oil (262 mg, 86% yield).

^{1}H NMR (500 MHz, $CDCl_3$): δ 7.43 – 7.37 (m, 2H), 7.32 – 7.26 (m, 3H), 3.90 – 3.82 (m, 1H), 3.55 (t, J = 7.5 Hz, 2H), 2.20 – 2.05 (m, 4H), 1.89 – 1.74 (m, 4H), 1.70 – 1.63 (m, 2H), 1.49 – 1.39 (m, 2H), 0.97 (t, J = 7.5 Hz, 3H); ^{13}C NMR (125 MHz, $CDCl_3$): δ 131.4, 128.4, 127.8, 123.1, 82.7, 70.2, 62.1,

51.9, 30.8, 28.1, 25.8, 19.6, 13.8; HRMS (EI-TOF) *m/z*: $[M]^+$ calcd. for $C_{17}H_{23}NO_2S$, 305.1449; found, 305.1445.

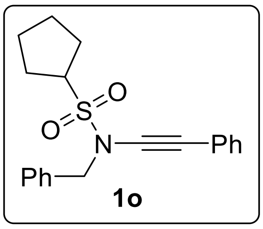

***N*-benzyl-*N*-(phenylethynyl)cyclopentanesulfonamide**

1o, White solid (268 mg, 79% yield).

^{1}H NMR (400 MHz, $CDCl_3$): δ 7.49 – 7.44 (m, 2H), 7.42 – 7.24 (m, 8H), 4.71 (s, 2H), 3.81 – 3.67 (m, 1H), 2.20 – 2.10 (m, 2H), 2.09 – 1.99 (m, 2H), 1.88 – 1.77 (m, 2H), 1.68 – 1.58 (m, 2H); ^{13}C NMR (100 MHz, $CDCl_3$): δ 135.3, 131.3, 129.0, 128.8, 128.6, 128.3, 127.8, 123.0, 83.0, 70.9, 62.8, 56.1, 28.1, 25.8; HRMS (EI-TOF) *m/z*: $[M]^+$ calcd. for $C_{20}H_{21}NO_2S$, 339.1293; found, 339.1295.

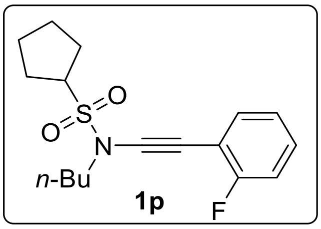

***N*-butyl-*N*-((2-fluorophenyl)ethynyl)cyclopentanesulfonamide**

1p, Yellow oil (301 mg, 94% yield).

^{1}H NMR (500 MHz, $CDCl_3$): δ 7.41 – 7.37 (m, 1H), 7.28 – 7.23 (m, 1H), 7.10 – 7.02 (m, 2H), 3.93 – 3.83 (m, 1H), 3.57 (t, *J* = 7.5 Hz, 2H), 2.22 – 2.08 (m, 4H), 1.90 – 1.75 (m, 4H), 1.71 – 1.62 (m, 2H), 1.50 – 1.40 (m, 2H), 0.98 (t, *J* = 7.5 Hz, 3H); ^{13}C NMR (125 MHz, $CDCl_3$): δ 162.6 (d, *J* = 250.3 Hz), 133.1 (d, *J* = 1.1 Hz), 129.4 (d, *J* = 7.8 Hz), 124.0 (d, *J* = 3.7 Hz), 115.4 (d, *J* = 20.9 Hz), 111.7 (d, *J* = 15.7 Hz), 87.5 (d, *J* = 3.0 Hz), 64.0, 62.2, 51.9, 30.6, 28.1, 25.8, 19.6, 13.7; ^{19}F NMR (376 MHz, $CDCl_3$): δ -111.10; HRMS (EI-TOF) *m/z*: $[M]^+$ calcd. for $C_{17}H_{22}FNO_2S$, 323.1355; found, 323.1358.

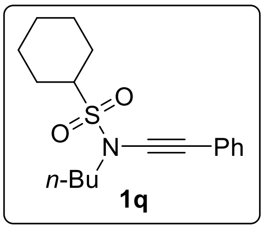

***N*-butyl-*N*-(phenylethynyl)cyclohexanesulfonamide**

1q, Yellow oil (268 mg, 84% yield).

^{1}H NMR (400 MHz, $CDCl_3$): δ 7.42 – 7.37 (m, 2H), 7.33 – 7.24 (m, 3H), 3.54 (t, *J* = 7.2 Hz, 2H), 3.37 – 3.27 (m, 1H), 2.28 – 2.19 (m, 2H), 1.97 – 1.88 (m, 2H), 1.82 – 1.60 (m, 5H), 1.50 – 1.38 (m, 2H), 1.37 – 1.19 (m, 3H), 0.97 (t, *J* = 7.2 Hz, 3H); ^{13}C NMR (100 MHz, $CDCl_3$): δ 131.4, 128.4, 127.8, 123.1, 82.7, 70.2, 62.2, 52.3, 30.9, 26.5, 25.2, 25.1, 19.6, 13.8; HRMS (EI-TOF) *m/z*: $[M]^+$ calcd. for $C_{18}H_{25}NO_2S$, 319.1606; found, 319.1601.

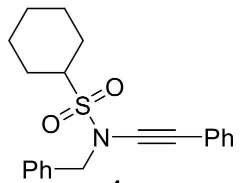

1r

***N*-benzyl-*N*-(phenylethynyl)cyclohexanesulfonamide**

1r, White solid (318 mg, 90% yield).

^{1}H NMR (400 MHz, $CDCl_3$): δ 7.46 (d, *J* = 6.8 Hz, 2H), 7.42 – 7.22 (m, 8H), 4.69 (s, 2H), 3.27 – 3.15 (m, 1H), 2.27 – 2.16 (m, 2H), 1.95 – 1.82 (m, 2H), 1.72 – 1.57 (m, 3H), 1.32 – 1.14 (m, 3H); ^{13}C NMR (100 MHz, $CDCl_3$): δ 135.3, 131.3, 129.0, 128.8, 128.6, 128.3, 127.8, 123.0, 83.1, 70.9, 62.8, 56.3, 26.5, 25.2, 25.1; HRMS (EI-TOF) *m/z*: $[M]^+$ calcd. for $C_{21}H_{23}NO_2S$, 353.1449; found, 353.1445.

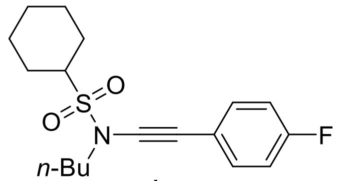

1s

***N*-butyl-*N*-((4-fluorophenyl)ethynyl)cyclohexanesulfonamide**

1s, Yellow oil (320 mg, 95% yield).

^{1}H NMR (500 MHz, $CDCl_3$): δ 7.42 – 7.31 (m, 2H), 7.05 – 6.94 (m, 2H), 3.53 (t, *J* = 7.5 Hz, 2H), 3.37 – 3.20 (m, 1H), 2.27 – 2.19 (m, 2H), 1.96 – 1.90 (m, 2H), 1.80 – 1.61 (m, 4H), 1.48 – 1.40 (m, 2H), 1.34 – 1.27 (m, 4H), 0.97 (t, *J* = 7.5 Hz, 3H); ^{13}C NMR (125 MHz, $CDCl_3$): δ 162.3 (d, *J* = 248.8 Hz), 133.5 (d, *J* = 8.3 Hz), 119.2 (d, *J* = 3.4 Hz), 115.6 (d, *J* = 22.1 Hz), 82.3 (d, *J* = 1.0 Hz), 69.1, 62.2, 52.3, 30.9, 26.5, 25.2, 25.1, 19.6, 13.8; ^{19}F NMR (376 MHz, $CDCl_3$): δ -112.30; HRMS (EI-TOF) *m/z*: $[M]^+$ calcd. for $C_{18}H_{24}FNO_2S$, 337.1512; found, 337.1517.

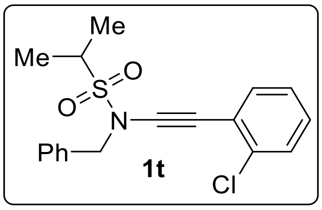

N-benzyl-N-((2-chlorophenyl)ethynyl)propane-2-sulfonamide

1t, White solid (277 mg, 80% yield).

^{1}H NMR (400 MHz, $CDCl_3$): δ 7.53 – 7.47 (m, 2H), 7.41 – 7.26 (m, 5H), 7.19 – 7.11 (m, 2H), 4.74 (s, 2H), 3.57 (hept, *J* = 7.2 Hz, 1H), 1.45 (d, *J* = 7.2 Hz, 6H); ^{13}C NMR (100 MHz, $CDCl_3$): δ 135.3, 135.1, 132.6, 129.2, 129.1, 128.8, 128.7, 128.6, 126.5, 123.0, 88.0, 68.5, 56.3, 55.1, 16.7; HRMS (EI-TOF) *m/z*: [M]$^+$calcd. for $C_{18}H_{18}ClNO_2S$, 347.0747; found, 347.0741.

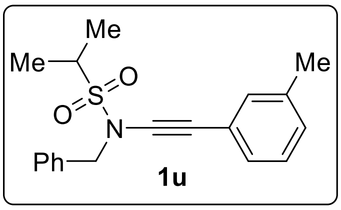

N-benzyl-N-(m-tolylethynyl)propane-2-sulfonamide

1u, White solid (281 mg, 86% yield).

^{1}H NMR (400 MHz, $CDCl_3$): δ 7.49 – 7.44 (m, 2H), 7.41 – 7.32 (m, 3H), 7.17 – 7.04 (m, 4H), 4.70 (s, 2H), 3.53 (hept, *J* = 6.8 Hz, 1H), 2.29 (s, 3H), 1.44 (d, *J* = 6.8 Hz, 6H); ^{13}C NMR (100 MHz, $CDCl_3$): δ 138.0, 135.3, 131.9, 129.0, 128.8, 128.7, 128.6, 128.4, 128.2, 122.7, 82.6, 71.1, 56.3, 54.9, 21.3, 16.7; HRMS (EI-TOF) *m/z*: [M]$^+$ calcd. for $C_{19}H_{21}NO_2S$, 327.1293; found, 327.1291.

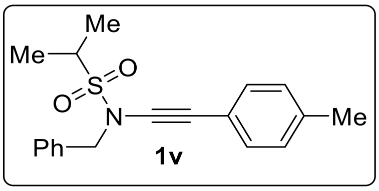

N-benzyl-N-(p-tolylethynyl)propane-2-sulfonamide

1v, White solid (294 mg, 90% yield).

^{1}H NMR (400 MHz, $CDCl_3$): δ 7.49 – 7.31 (m, 5H), 7.20 (d, *J* = 8.0 Hz, 2H), 7.06 (d, *J* = 8.0 Hz, 2H), 4.69 (s, 2H), 3.53 (hept, *J* = 6.8 Hz, 1H), 2.32 (s, 3H), 1.44 (d, *J* = 6.8 Hz, 6H); ^{13}C NMR (100 MHz, $CDCl_3$): δ 138.1, 135.4, 131.5, 129.1, 129.0, 128.8, 128.6, 119.7, 82.1, 70.9, 56.3, 54.8, 21.5, 16.7; HRMS (EI-TOF) *m/z*: [M]$^+$ calcd. for $C_{19}H_{21}NO_2S$, 327.1293; found, 327.1295.

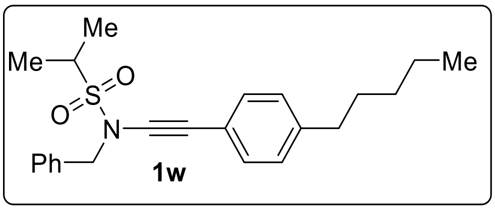

***N*-benzyl-*N*-((4-pentylphenyl)ethynyl)propane-2-sulfonamide**

1w, White solid (310 mg, 81% yield).

^{1}H NMR (400 MHz, $CDCl_3$): δ 7.50 – 7.31 (m, 5H), 7.22 (d, *J* = 7.2 Hz, 2H), 7.07 (d, *J* = 7.2 Hz, 2H), 4.69 (s, 2H), 3.53 (hept, *J* = 6.8 Hz, 1H), 2.56 (t, *J* = 7.2 Hz, 2H), 1.62 – 1.53 (m, 2H), 1.44 (d, *J* = 7.2 Hz, 6H), 1.37 – 1.21 (m, 4H), 0.88 (t, *J* = 6.8 Hz, 3H); ^{13}C NMR (100 MHz, $CDCl_3$): δ 143.1, 135.4, 131.5, 129.0, 128.8, 128.6, 128.5, 119.9, 82.2, 70.9, 56.3, 54.8, 35.9, 31.5, 31.1, 22.6, 16.7, 14.1; HRMS (EI-TOF) *m/z*: $[M]^+$ calcd. for $C_{23}H_{29}NO_2S$, 383.1919; found, 383.1916.

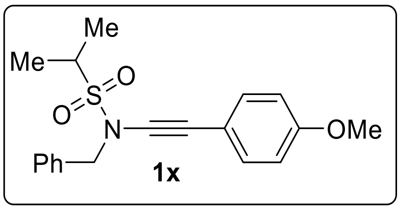

***N*-benzyl-*N*-((4-methoxyphenyl)ethynyl)propane-2-sulfonamide**

1x, White solid (268 mg, 78% yield).

^{1}H NMR (400 MHz, $CDCl_3$): δ 7.49 – 7.30 (m, 5H), 7.25 (d, *J* = 8.4 Hz, 2H), 6.79 (d, *J* = 8.4 Hz, 2H), 4.69 (s, 2H), 3.78 (s, 3H), 3.53 (hept, *J* = 6.8 Hz, 1H), 1.43 (d, *J* = 6.8 Hz, 6H); ^{13}C NMR (100 MHz, $CDCl_3$): δ 159.6, 135.4, 133.4, 129.0, 128.7, 128.5, 114.8, 114.0, 81.4, 70.5, 56.3, 55.4, 54.7, 16.7; HRMS (EI-TOF) *m/z*: $[M]^+$ calcd. for $C_{19}H_{21}NO_3S$, 343.1242; found, 343.1247.

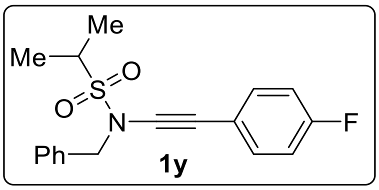

***N*-benzyl-*N*-((4-fluorophenyl)ethynyl)propane-2-sulfonamide**

1y, White solid (298 mg, 90% yield).

^{1}H NMR (400 MHz, $CDCl_3$): δ 7.49 – 7.34 (m, 5H), 7.30 – 7.23 (m, 2H), 6.99 – 6.92 (m, 2H), 4.70 (s, 2H), 3.52 (hept, *J* = 6.8 Hz, 1H), 1.45 (d, *J* = 6.8 Hz, 6H); ^{13}C NMR (100 MHz, $CDCl_3$): δ 162.4 (d, *J* = 249.1 Hz), 135.2, 133.4 (d, *J* = 8.3 Hz), 129.0, 128.8, 128.7, 118.9 (d, *J* = 3.4 Hz), 115.6 (d, *J* = 22.0

Hz), 82.4, 69.9, 56.3, 55.0, 16.7; ^{19}F NMR (376 MHz, $CDCl_3$): δ -112.14; HRMS (EI-TOF) *m/z*: $[M]^+$ calcd. for $C_{18}H_{18}FNO_2S$, 331.1042; found, 331.1045.

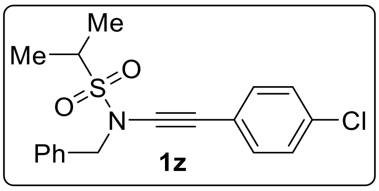

***N*-benzyl-*N*-((4-chlorophenyl)ethynyl)propane-2-sulfonamide**

1z, White solid (323 mg, 93% yield).

^{1}H NMR (400 MHz, $CDCl_3$): δ 7.48 – 7.33 (m, 5H), 7.25 – 7.17 (m, 4H), 4.70 (s, 2H), 3.51 (hept, *J* = 6.8 Hz, 1H), 1.45 (d, *J* = 6.8 Hz, 6H); ^{13}C NMR (150 MHz, $CDCl_3$): δ 135.1, 133.8, 132.5, 129.0, 128.9, 128.7 (two peaks: 128.72 and 182.66), 121.4, 83.8, 70.1, 56.3, 55.1, 16.7; HRMS (EI-TOF) *m/z*: $[M]^+$ calcd. for $C_{18}H_{18}ClNO_2S$, 347.0747; found, 347.0745.

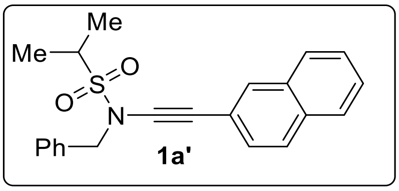

***N*-benzyl-*N*-(naphthalen-2-ylethynyl)propane-2-sulfonamide**

1a', White solid (290 mg, 80% yield).

^{1}H NMR (600 MHz, $CDCl_3$): δ 7.80 (s, 1H), 7.79 – 7.76 (m, 1H), 7.75 – 7.70 (m, 2H), 7.52 – 7.49 (m, 2H), 7.47 – 7.35 (m, 5H), 7.33 (dd, J_1= 8.4 Hz, J_2 = 1.8 Hz, 1H), 4.75 (s, 2H), 3.57 (hept, *J* = 7.2 Hz, 1H), 1.47 (d, *J* = 7.2 Hz, 6H); ^{13}C NMR (150 MHz, $CDCl_3$): δ 135.3, 133.1, 132.6, 130.8, 129.1, 128.9, 128.7, 128.4, 128.0, 127.9, 127.7, 126.6, 126.5, 120.2, 83.3, 71.5, 56.4, 55.0, 16.8; HRMS (EI-TOF) *m/z*: $[M]^+$ calcd. for $C_{22}H_{21}NO_2S$, 363.1293; found, 363.1295.

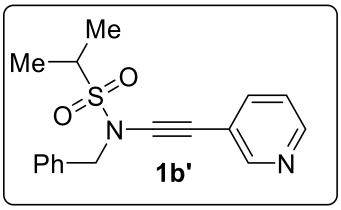

***N*-benzyl-*N*-(pyridin-3-ylethynyl)propane-2-sulfonamide**

1b', White solid (242 mg, 77% yield).

^{1}H NMR (600 MHz, $CDCl_3$): δ 8.57 – 8.41 (m, 2H), 7.59 – 7.35 (m, 6H), 7.21 – 7.15 (m, 1H), 4.72 (s, 2H), 3.52 (hept, *J* = 6.6 Hz, 1H), 1.47 (d, *J* = 6.6 Hz, 6H); ^{13}C NMR (150 MHz, $CDCl_3$): δ 151.9,

148.2, 138.1, 134.9, 129.0, 128.9, 128.8, 123.0, 120.2, 86.0, 68.1, 56.3, 55.3, 16.7; HRMS (EI-TOF) *m/z*: $[M]^+$ calcd. for $C_{17}H_{18}N_2O_2S$, 314.1089; found, 314.1093.

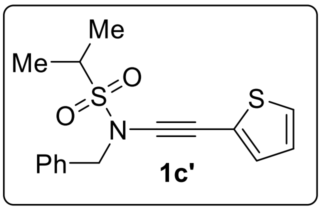

***N*-benzyl-*N*-(thiophen-2-ylethynyl)propane-2-sulfonamide**

1c', White solid (156 mg, 49% yield).

^{1}H NMR (400 MHz, $CDCl_3$): δ 7.48 – 7.33 (m, 5H), 7.24 (d, *J* = 5.2 Hz, 1H), 7.13 (d, *J* = 3.6 Hz, 1H), 6.94 (dd, J_1 = 5.2 Hz, J_2 = 3.6 Hz, 1H), 4.70 (s, 2H), 3.51 (hept, *J* = 6.8 Hz, 1H), 1.43 (d, *J* = 6.8 Hz, 6H); ^{13}C NMR (100 MHz, $CDCl_3$): δ 135.2, 133.2, 129.0, 128.8, 128.7, 127.9, 127.1, 122.8, 86.4, 64.2, 56.5, 55.0, 16.7; HRMS (EI-TOF) *m/z*: $[M]^+$ calcd. for $C_{16}H_{17}NO_2S_2$, 319.0701; found, 319.0706.

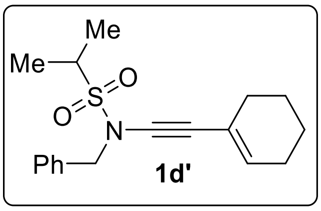

***N*-benzyl-*N*-(cyclohex-1-en-1-ylethynyl)propane-2-sulfonamide**

1d', Yellow oil (234 mg, 74% yield).

^{1}H NMR (600 MHz, $CDCl_3$): δ 7.46 – 7.30 (m, 5H), 5.99 – 5.89 (m, 1H), 4.61 (s, 2H), 3.48 (hept, *J* = 6.6 Hz, 1H), 2.09 – 1.99 (m, 4H), 1.63 – 1.51 (m, 4H), 1.40 (d, *J* = 6.6 Hz, 6H); ^{13}C NMR (150 MHz, $CDCl_3$): δ 135.5, 133.8, 128.9, 128.7, 128.4, 120.0, 80.4, 72.5, 56.2, 54.5, 29.5, 25.7, 22.4, 21.6, 16.7; HRMS (EI-TOF) *m/z*: $[M]^+$ calcd. for $C_{18}H_{23}NO_2S$, 317.1449; found, 317.1443.

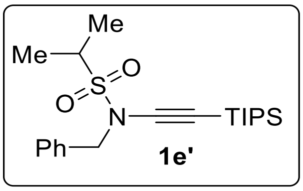

***N*-benzyl-*N*-((triisopropylsilyl)ethynyl)propane-2-sulfonamide**

1e', K_3PO_4 instead of K_2CO_3, colorless oil (342 mg, 87% yield).

^{1}H NMR (400 MHz, $CDCl_3$): δ 7.44 – 7.38 (m, 2H), 7.37 – 7.29 (m, 3H), 4.61 (s, 2H), 3.47 (hept, *J* = 6.8 Hz, 1H), 1.41 (d, *J* = 6.8 Hz, 6H), 0.99 (s, 21H); ^{13}C NMR (100 MHz, $CDCl_3$): δ 135.2, 129.0,

128.7, 128.5, 96.9, 69.78 56.0, 55.1, 18.6, 16.7, 11.4; HRMS (EI-TOF) *m/z*: $[M]^+$ calcd. for $C_{21}H_{35}NO_2SSi$, 393.2158; found, 393.2154.

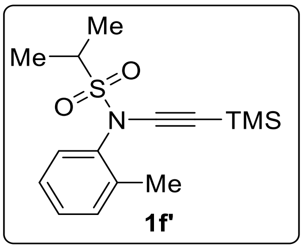

N-(o-tolyl)-N-((trimethylsilyl)ethynyl)propane-2-sulfonamide

1f', Yellow solid (80 mg, 26% yield).

^{1}H NMR (500 MHz, $CDCl_3$): δ 7.33 (d, *J* = 7.5 Hz, 1H), 7.30 – 7.27 (m, 2H), 7.26 – 7.21 (m, 1H), 3.74 (hept, *J* = 7.0 Hz, 1H), 2.47 (s, 3H), 1.56 (d, *J* = 7.0 Hz, 6H), 0.15 (s, 9H); ^{13}C NMR (125 MHz, $CDCl_3$): δ 137.9, 137.3, 131.9, 129.4, 128.2, 127.1, 95.7, 71.6, 55.0, 18.5, 17.1, 0.2; HRMS (EI-TOF) *m/z*: $[M]^+$ calcd. for $C_{15}H_{23}NO_2SSi$, 309.1219; found, 309.1215.

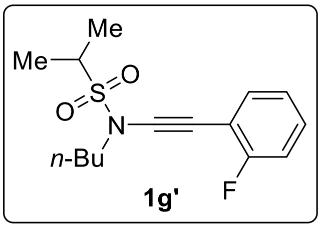

N-butyl-N-((2-fluorophenyl)ethynyl)propane-2-sulfonamide

1g', Light yellow oil (267 mg, 90% yield).

^{1}H NMR (500 MHz, $CDCl_3$): δ 7.42 – 7.36 (m, 1H), 7.28 – 7.23 (m, 1H), 7.10 – 7.03 (m, 2H), 3.64 (hept, *J* = 7.0 Hz, 1H), 3.57 (t, *J* = 7.5 Hz, 2H), 1.84 – 1.76 (m, 2H), 1.49 (d, *J* = 7.0 Hz, 6H), 1.47 – 1.41 (m, 2H), 0.98 (t, *J* = 7.5 Hz, 3H); ^{13}C NMR (125 MHz, $CDCl_3$): δ 162.6 (d, *J* = 250.3 Hz), 133.2 (d, *J* = 1.0 Hz), 129.5 (d, *J* = 7.8 Hz), 124.0 (d, *J* = 3.7 Hz), 115.5 (d, *J* = 20.9 Hz), 111.7 (d, *J* = 15.7 Hz), 87.4 (d, *J* = 3.0 Hz) ,64.0, 54.6, 52.3, 30.8, 19.6, 16.7, 13.8; ^{19}F NMR (376 MHz, $CDCl_3$): δ -110.55; HRMS (EI-TOF) *m/z*: $[M]^+$ calcd. for $C_{15}H_{20}FNO_2S$, 297.1199; found, 297.1190.

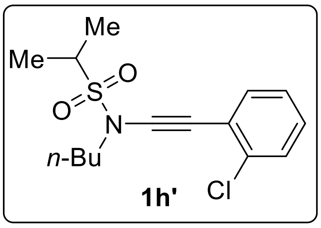

N-butyl-N-((2-chlorophenyl)ethynyl)propane-2-sulfonamide

1h’, Light yellow oil (253 mg, 81% yield).

^{1}H NMR (500 MHz, $CDCl_3$): δ 7.44 – 7.33 (m, 2H), 7.23 – 7.14 (m, 2H), 3.67 (hept, *J* = 7.0 Hz, 1H), 3.58 (t, *J* = 7.0 Hz, 2H), 1.87 – 1.78 (m, 2H), 1.53 – 1.41 (m, 8H), 0.98 (t, *J* = 7.5 Hz, 3H); ^{13}C NMR (125 MHz, $CDCl_3$): δ 135.2, 132.4, 129.2, 128.5, 126.5, 123.1, 87.7, 67.8, 54.4, 52.2, 30.65, 19.5, 16.6, 13.7; HRMS (EI-TOF) *m/z*: $[M]^+$ calcd. for $C_{15}H_{20}ClNO_2S$, 313.0903; found, 313.0908.

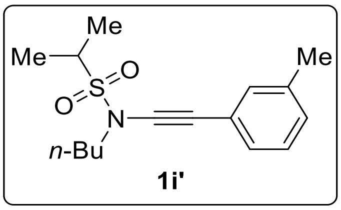

***N*-butyl-*N*-(*m*-tolylethynyl)propane-2-sulfonamide**

1i’, Light yellow oil (234 mg, 80% yield).

^{1}H NMR (500 MHz, $CDCl_3$): δ 7.23 – 7.15 (m, 3H), 7.10 – 7.07 (m, 1H), 3.60 (hept, *J* = 7.0 Hz, 1H), 3.54 (t, *J* = 7.0 Hz, 2H), 2.31 (s, 3H), 1.81 – 1.74 (m, 2H), 1.48 – 1.41 (m, 8H), 0.97 (t, *J* = 7.5 Hz, 3H); ^{13}C NMR (125 MHz, $CDCl_3$): δ 138.0, 131.9, 128.7, 128.4, 128.2, 122.8, 82.2, 70.3, 54.2, 52.2, 30.8, 21.2, 19.5, 16.6, 13.7; HRMS (EI-TOF) *m/z*: $[M]^+$ calcd. for $C_{16}H_{23}NO_2S$, 293.1449; found, 293.1444.

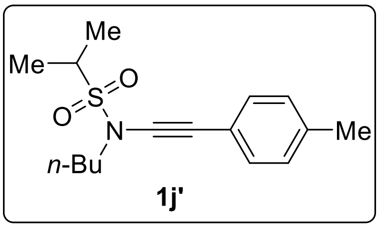

***N*-butyl-*N*-(*p*-tolylethynyl)propane-2-sulfonamide**

1j’, Light yellow oil (240 mg, 82% yield).

^{1}H NMR (500 MHz, $CDCl_3$): δ 7.31 – 7.27 (m, 2H), 7.12 – 7.08 (m, 2H), 3.61 (hept, *J* = 7.0 Hz, 1H), 3.54 (t, *J* = 7.0 Hz, 2H), 2.33 (s, 3H), 1.81 – 1.74 (m, 2H), 1.48 – 1.40 (m, 8H), 0.97 (t, *J* = 7.5 Hz, 3H); ^{13}C NMR (125 MHz, $CDCl_3$): δ 138.0, 131.5, 129.1, 119.9, 81.8, 70.1, 54.2, 52.3, 30.9, 21.5, 19.6, 16.7, 13.8; HRMS (EI-TOF) *m/z*: $[M]^+$ calcd. for $C_{16}H_{23}NO_2S$, 293.1449; found, 293.1448.

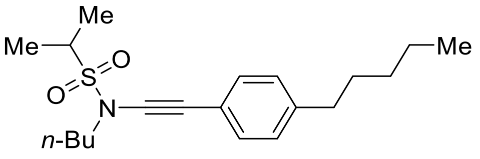

1k'

***N*-butyl-*N*-((4-pentylphenyl)ethynyl)propane-2-sulfonamide**

1k', Light yellow oil (258 mg, 74% yield).

^{1}H NMR (500 MHz, $CDCl_3$): δ 7.33 – 7.30 (m, 2H), 7.11 (d, *J* = 8.3 Hz, 2H), 3.61 (hept, *J* = 7.0 Hz, 1H), 3.54 (t, *J* = 7.0 Hz, 2H), 2.58 (t, *J* = 7.5 Hz, 2H), 1.81 – 1.74 (m, 2H), 1.62 – 1.55 (m, 2H), 1.47 (d, *J* = 7.0 Hz, 6H), 1.45 – 1.40 (m, 2H), 1.34 – 1.26 (m, 4H), 0.97 (t, *J* = 7.5 Hz, 3H), 0.88 (t, *J* = 7.0 Hz, 3H); ^{13}C NMR (125 MHz, $CDCl_3$): δ 143.1, 131.6, 128.5, 120.1, 81.9, 70.2, 54.2, 52.3, 35.9, 31.5, 31.1, 30.9, 22.6, 19.6, 16.7, 14.2, 13.8; HRMS (EI-TOF) *m/z*: $[M]^+$ calcd. for $C_{20}H_{31}NO_2S$, 349.2075; found, 349.2071.

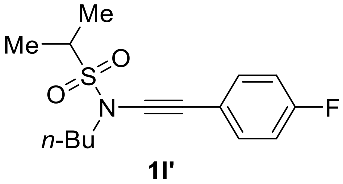

1l'

***N*-butyl-*N*-((4-fluorophenyl)ethynyl)propane-2-sulfonamide**

1l', Light yellow oil (276 mg, 93% yield).

^{1}H NMR (500 MHz, $CDCl_3$): δ 7.42 – 7.34 (m, 2H), 7.03 – 6.96 (m, 2H), 3.64 – 3.57 (m, 1H), 3.54 (t, *J* = 7.2 Hz, 2H), 1.81 – 1.73 (m, 2H), 1.47 (d, *J* = 7.2 Hz, 6H), 1.46 – 1.40 (m, 2H), 0.97 (t, *J* = 7.5 Hz, 3H); ^{13}C NMR (125 MHz, $CDCl_3$): δ 162.3 (d, *J* = 248.9 Hz), 133.5 (d, *J* = 8.3 Hz), 119.0 (d, *J* = 3.4 Hz), 115.6 (d, *J* = 22.1 Hz), 82.2 (d, *J* = 0.7 Hz), 69.0, 54.3, 52.2, 30.8, 19.5, 16.6, 13.7; ^{19}F NMR (376 MHz, $CDCl_3$): δ -112.22; HRMS (EI-TOF) *m/z*: $[M]^+$ calcd. for $C_{15}H_{20}FNO_2S$, 297.1199; found, 297.1190.

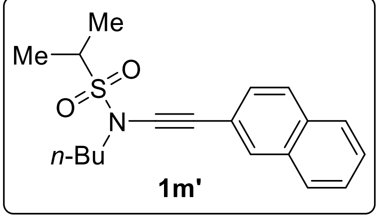

1m'

***N*-butyl-*N*-(naphthalen-2-ylethynyl)propane-2-sulfonamide**

1m', Yellow oil (230 mg, 70% yield).

^{1}H NMR (500 MHz, $CDCl_3$): δ 7.90 (s, 1H), 7.81 – 7.74 (m, 3H), 7.50 – 7.42 (m, 3H), 3.65 (hept, *J* = 7.0 Hz, 1H), 3.59 (t, *J* = 7.5 Hz, 2H), 1.86 – 1.79 (m, 2H), 1.51 – 1.43 (m, 8H), 0.99 (t, *J* = 7.5 Hz, 3H); ^{13}C NMR (125 MHz, $CDCl_3$): δ 133.1, 132.6, 130.8, 128.5, 128.0, 127.9, 127.7, 126.6, 126.5, 120.4, 83.0, 70.7, 54.4, 52.4, 31.0, 19.6, 16.8, 13.8; HRMS (EI-TOF) *m/z*: $[M]^+$ calcd. for $C_{19}H_{23}NO_2S$, 329.1449; found, 329.1443.

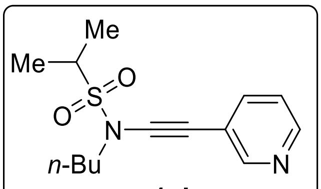

1n'

***N*-butyl-*N*-(pyridin-3-ylethynyl)propane-2-sulfonamide**

1n', Light yellow oil (210 mg, 75% yield)

^{1}H NMR (400 MHz, $CDCl_3$): δ 8.62 (d, *J* = 1.6 Hz, 1H), 8.49 (dd, J_1 = 4.8 Hz, J_2 = 1.2 Hz, 1H), 7.68 (dt, J_1 = 8.0 Hz, J_2 = 2.0 Hz, 1H), 7.26 – 7.21 (m, 1H), 3.66 – 3.54 (m, 3H), 1.84 – 1.75 (m, 2H), 1.52 – 1.40 (m, 8H), 0.98 (t, *J* = 7.2 Hz, 3H); ^{13}C NMR (100 MHz, $CDCl_3$): δ 151.9, 148.1, 138.2, 123.0, 120.3, 85.8, 67.2, 54.7, 52.3, 30.9, 19.5, 16.7, 13.7; HRMS (EI-TOF) *m/z*: $[M]^+$ calcd. for $C_{14}H_{20}N_2O_2S$, 280.1245; found, 280.1250.

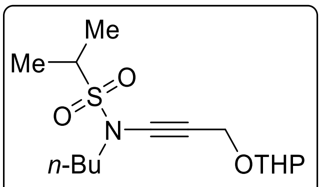

1o'

***N*-butyl-*N*-(3-((tetrahydro-2*H*-pyran-2-yl)oxy)prop-1-yn-1-yl)propane-2-sulfonamide**

1o', Light yellow oil (246 mg, 70% yield)

^{1}H NMR (400 MHz, $CDCl_3$): δ 7.48 – 7.28 (m, 5H), 4.69 – 4.62 (m, 3H), 4.36 (d, *J* = 1.6 Hz, 2H), 3.90 – 3.72 (m, 1H), 3.56 – 3.43 (m, 2H), 1.85 – 1.76 (m, 1H), 1.74 – 1.66 (m, 1H), 1.62 – 1.50 (m, 4H), 1.42 (d, *J* = 6.8 Hz, 6H); ^{13}C NMR (100 MHz, $CDCl_3$): δ 135.3, 128.8 (two peaks: 128.81 and 128.76), 128.5, 96.6, 79.7, 67.8, 62.3, 56.0, 54.6, 54.5, 30.4, 25.5, 19.4, 16.6 (two peaks: 16.61, 16.60); HRMS (EI-TOF) *m/z*: $[M]^+$ calcd. for $C_{18}H_{25}NO_4S$, 351.1504; found, 351.1500.

3. Typical Procedure for the Synthesis of 2a

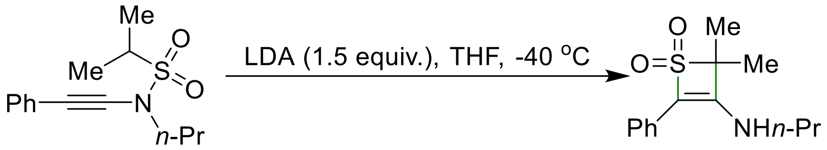

1a **2a**

An oven-dried Schlenk tube equipped with a magnetic stirrer bar was purged with argon three times. Ynamide **1a** (53 mg, 0.2 mmol) was dissolved in 2 mL anhydrous THF and added by a syringe. The mixture was cooled to -40 °C and LDA (2 mol/L in THF, 0.15 mL, 0.3 mmol) was added dropwise. The reaction was stirred at -40 °C for another 1 h. MeOH (0.1 mL) was added to quench the reaction and the mixture was concentrated under vacuum to obtain the residue, which was purified by silica gel column chromatography using ethyl acetate/petroleum ether (v/v, 1:2) as eluent to give 2,2-dimethyl-4-phenyl-3-(propylamino)-2*H*-thiete 1,1-dioxide **2a** (38 mg, 72% yield) as a light yellow solid.

4. Typical Procedure for the Synthesis of 3a

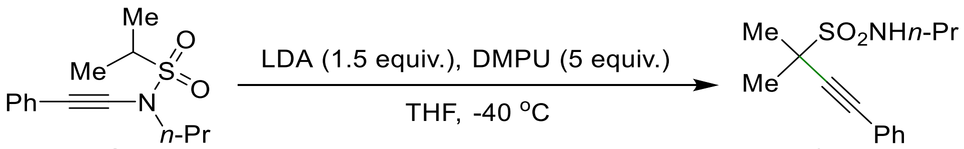

1a **3a**

An oven-dried schlenk tube equipped with a magnetic stirrer bar was purged with argon three times. Ynamide **1a** (53 mg, 0.2 mmol) was dissolved in 2 mL anhydrous THF and added by a syringe. DMPU (122 μL, 1 mmol) was added and the mixture was cooled to -40 °C. Subsequently, LDA (2 mol/L in THF, 0.15 mL, 0.3 mmol) was added dropwise. The reaction was stirred at -40 °C for another 1 h, and then MeOH (0.1 mL) was added to quench the reaction. The mixture was concentrated under vacuum to obtain the residue, which was purified by silica gel column chromatography using ethyl acetate/petroleum ether (v/v, 1:10) as eluent to give 2-methyl-4-phenyl-*N*-propylbut-3-yne-2-sulfonamide **3a** (43 mg, 82% yield) as a white solid.

5. Gram-Scale reactions of 2a and 3a

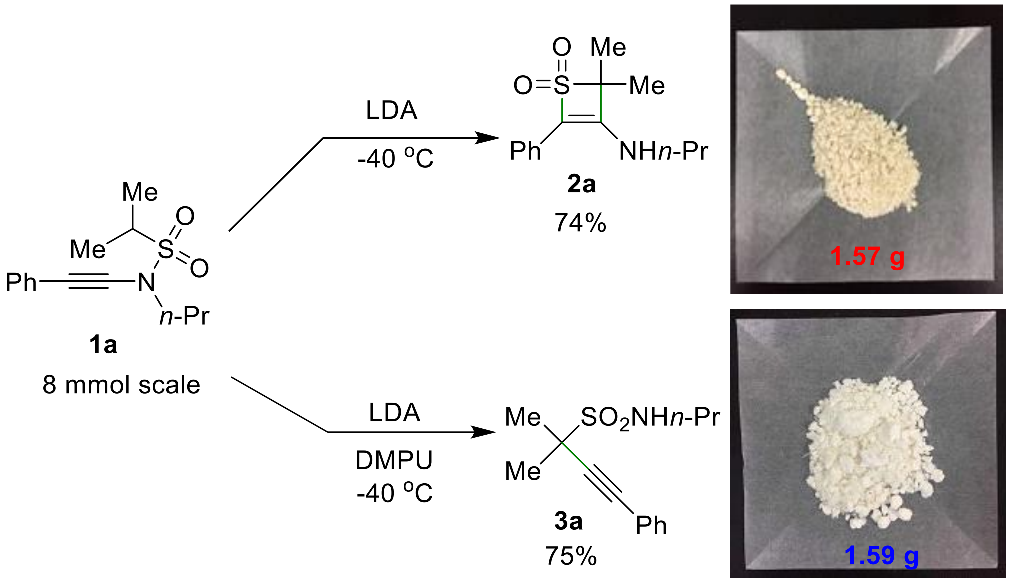

An oven-dried flask equipped with a magnetic stirrer bar was purged with argon three times. Ynamide **1a** (2.12 g, 8 mmol) was dissolved in 80 mL anhydrous THF and added by a syringe. The mixture was cooled to -40 °C and LDA (2 mol/L in THF, 6 mL, 12 mmol) was added dropwise. The reaction was stirred at -40 °C for another 1 h. MeOH (0.5 mL) was added to quench the reaction and then the mixture was concentrated under vacuum to obtain the residue, which was further purified by silica gel column chromatography using ethyl acetate/petroleum ether (v/v, 1:2) as eluent to give 2,2-dimethyl-4-phenyl-3-(propylamino)-2*H*-thiete 1,1-dioxide **2a** (1.57 g, 74% yield) as a light yellow solid.

An oven-dried flask equipped with a magnetic stirrer bar was purged with argon three times. Ynamide **1a** (2.12 g, 8 mmol) was dissolved in 80 mL anhydrous THF and added by a syringe. DMPU (4.8 mL, 40 mmol) was added and the mixture was cooled to -40 °C. Subsequently, LDA (2 mol/L in THF, 6 mL, 12 mmol) was added dropwise. The reaction was stirred at -40 °C for another 1 h, and then MeOH (0.5 mL) was added to quench the reaction. The mixture was concentrated under vacuum to obtain the residue, which was further purified by silica gel column chromatography using ethyl acetate/petroleum ether (v/v, 1:10) as eluent to give 2-methyl-4-phenyl-*N*-propylbut-3-yne-2-sulfonamide **3a** (1.59 g, 75 % yield) as a white solid.

6. Procedure for the Synthesis of 4

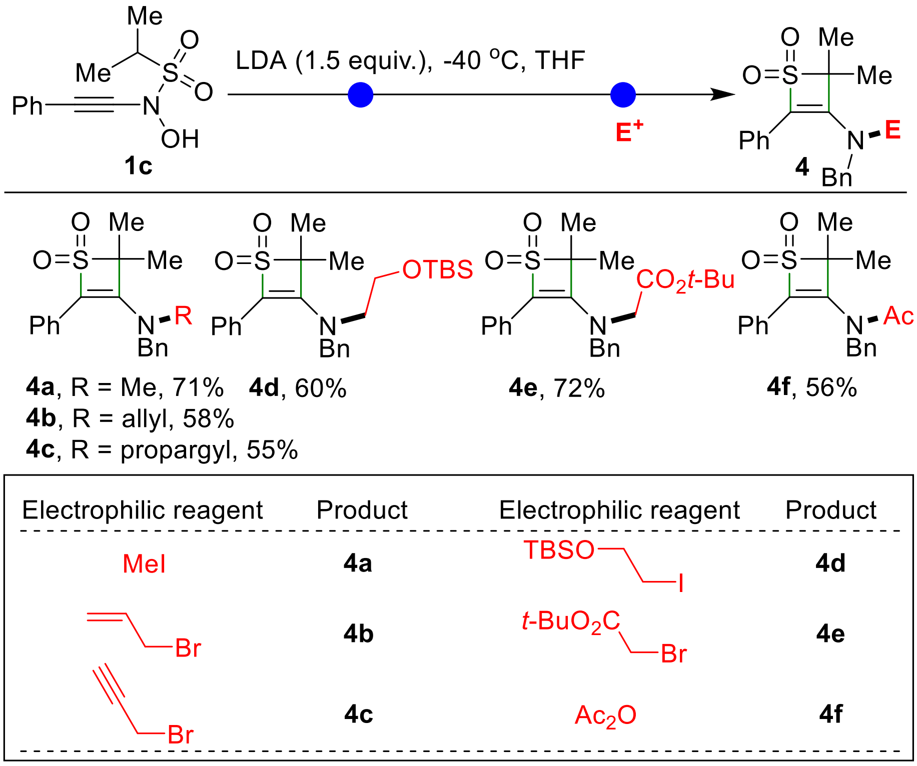

An oven-dried schlenk tube equipped with a magnetic stirrer bar was charged with ynamide **1c** (63 mg, 0.2 mmol) and purged with argon three times. 2 mL anhydrous THF was added by a syringe. The mixture was cooled to -40 °C and LDA (2 mol/L in THF, 0.15 mL, 0.3 mmol) was added dropwise. The reaction was stirred at -40 °C for another 1 h. Corresponding electrophilic reagent (0.5 mmol) was added to the reaction and the reaction was warmed to room temperature. After stirring for about 30 mins, the mixture was concentrated under vacuum to obtain the residue, which was further purified by silica gel column chromatography using ethyl acetate/petroleum ether as eluent system to give products **4a–4f** in 55% to 72% yields.

7. Procedure for the Synthesis of 5

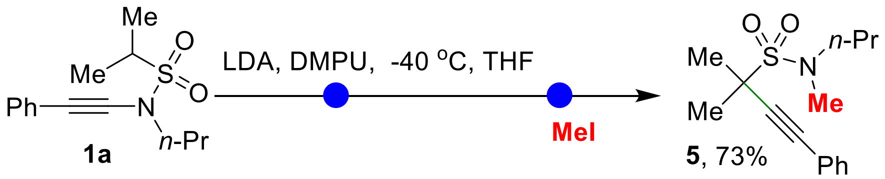

An oven-dried schlenk tube equipped with a magnetic stirrer bar was purged with argon three times. Ynamide **1a** (53 mg, 0.2 mmol) was dissolved in 2 mL anhydrous THF and added by a syringe.

DMPU (120 µL, 1 mmol) was added and the mixture was cooled to -40 °C. Subsequently, LDA (2 mol/L in THF, 0.15 mL, 0.3 mmol) was added dropwise. The reaction was stirred at -40 °C for another 1 h. MeI (35 µL, 0.5 mmol) was added and the reaction was warmed to room temperature. After stirring for 30 mins, the mixture was concentrated under vacuum to obtain the residue, which was further purified by silica gel column chromatography using ethyl acetate/petroleum ether (v/v, 1:20) as eluent to give *N*,2-dimethyl-4-phenyl-*N*-propylbut-3-yne-2-sulfonamide **5** (41 mg, 73% yield) as a colorless oil.

8. Crossover Experiment

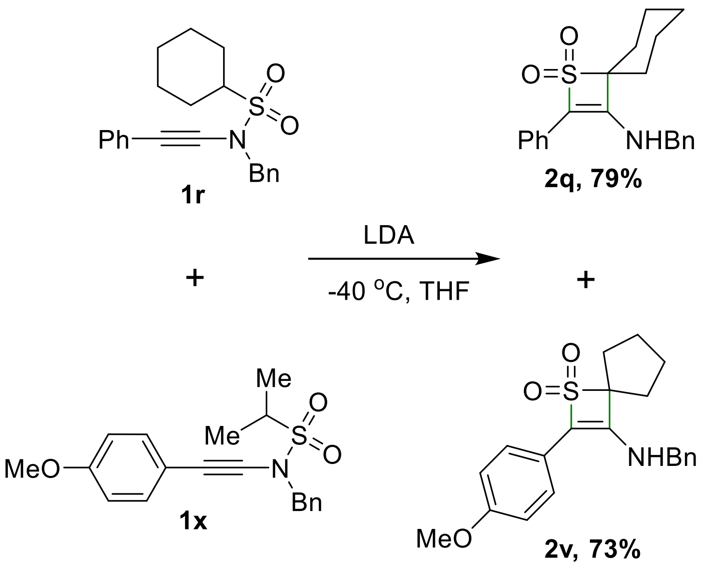

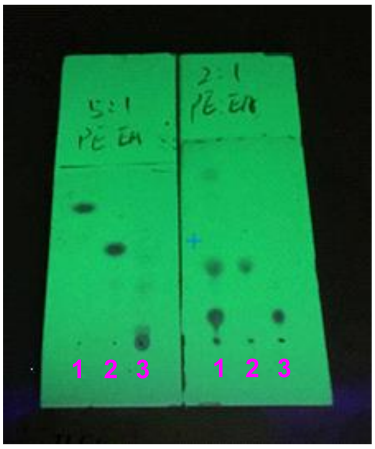

TLC:

left: PE: EA = 5:1
Line 1: **1r**
Line 2: **1x**
Line 3: **Reaction**

right: PE: EA = 2:1
Line 1: **Reaction**
Line 2: **2q**
Line 3: **2v**

An oven-dried schlenk tube equipped with a magnetic stirrer bar was charged with Ynamide **1r** (71 mg, 0.2 mmol) and Ynamide **1x** (69 mg, 0.2 mmol), then purged with argon three times. 4 mL anhydrous THF was added by a syringe. The mixture was cooled to -40 °C and LDA (2 mol/L in THF, 0.3 mL, 0.6 mmol) was added dropwise. The reaction was stirred at -40 °C for another 1 h. MeOH (0.1 mL) was added to quench the reaction and then the mixture was concentrated under vacuum to obtain the residue, which was further purified by silica gel column chromatography using ethyl acetate/petroleum ether (v/v, 1:3 to 2:1) as eluent to give **2q** (56 mg, 79% yield) and **2v** (51 mg, 73% yield) respectively.

9. α-Methylation Experiment

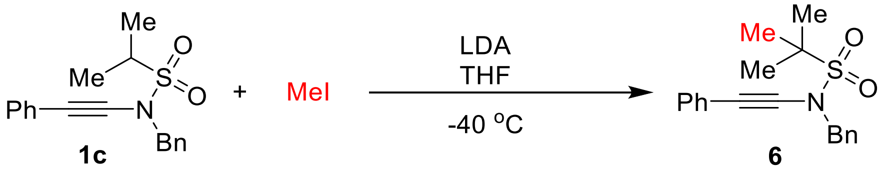

An oven-dried schlenk tube equipped with a magnetic stirrer bar was charged with Ynamide **1c** (63 mg, 0.2 mmol), then purged with argon three times. 2 mL anhydrous THF and MeI (70 µL, 1 mmol) were added. The mixture was cooled to -40 °C and LDA (2 mol/L in THF, 0.15 mL, 0.3 mmol) was added dropwise. The reaction was stirred at -40 °C for another 1 h, and then concentrated under vacuum to obtain the residue, which was further purified by silica gel column chromatography using ethyl acetate/petroleum ether (v/v, 1:20) as eluent to give **6** (59 mg, 91% yield).

10. ^{13}C-Labelled Experiments

10.1 Synthesis of (β-^{13}C)-1a

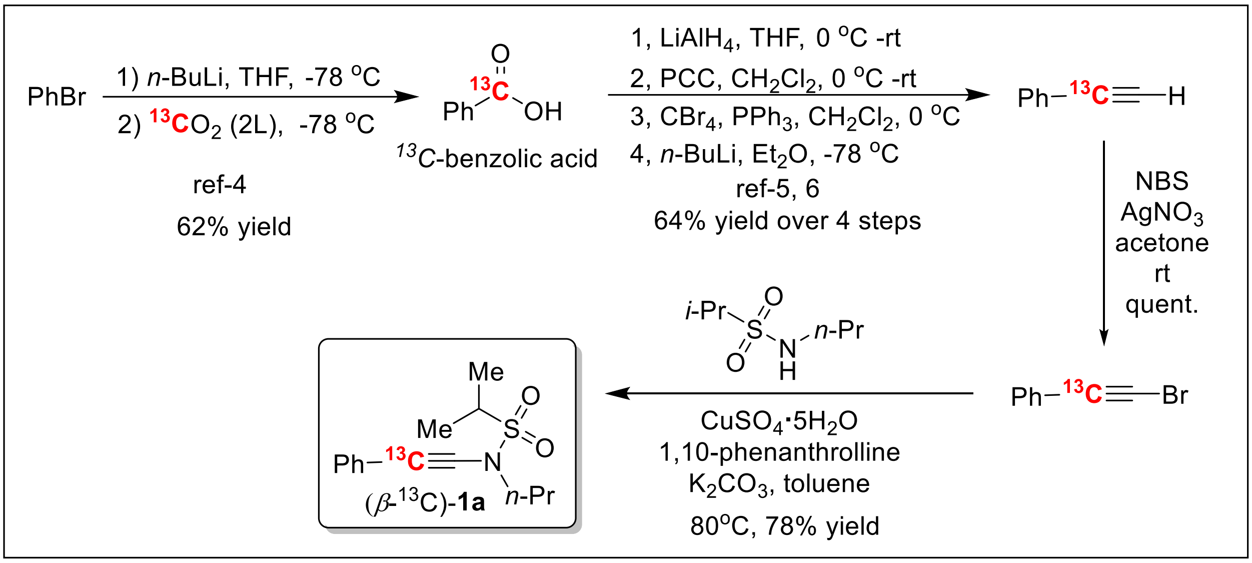

Supplementary Fig. 4 Synthesis of (β-^{13}C)-1a. (β-^{13}C)-**1a** could be successfully prepared via this synthetic route.

(β-^{13}C)-**1a** was prepared by above synthetic route (Supplementary Fig. 4). ^{13}C-benzolic acid was synthesized via the reported method[4] and ^{13}C-phenylacetylene was obtained according to the literature[5-6]. (β-^{13}C)-**1a** was synthesized via the general procedure described in Supplementary Fig. 2.

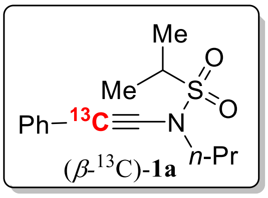

***N*-(phenylethynyl-2-^{13}C)-*N*-propylpropane-2-sulfonamide**

^{1}H NMR (500 MHz, $CDCl_3$): δ 7.42 – 7.37 (m, 2H), 7.32 – 7.24 (m, 3H), 3.62 (hept, J = 7.0 Hz, 1H), 3.52 (t, J = 7.0 Hz, 1H), 1.89 – 1.76 (m, 2H), 1.48 (d, J = 7.0 Hz, 3H), 1.02 (t, J = 7.0 Hz, 2H); **^{13}C NMR (125 MHz, $CDCl_3$):** δ 131.5 (d, J = 1.9 Hz), 128.4 (d, J = 5.8 Hz), 127.9 (d, J = 1.6 Hz), 123.1 (d, J = 94.8 Hz), 82.7 (d, J = 215.9 Hz), 70.3 (*^{13}C*-labelled), 54.4, 54.2 (d, J = 0.9 Hz), 22.2, 16.8, 11.0. **HRMS (ESI-TOF)** *m/z*: $[M]^+$ calcd. for $C_{13}{}^{13}CH_{19}NNaO_2S$, 289.1068; found, 289.1060.

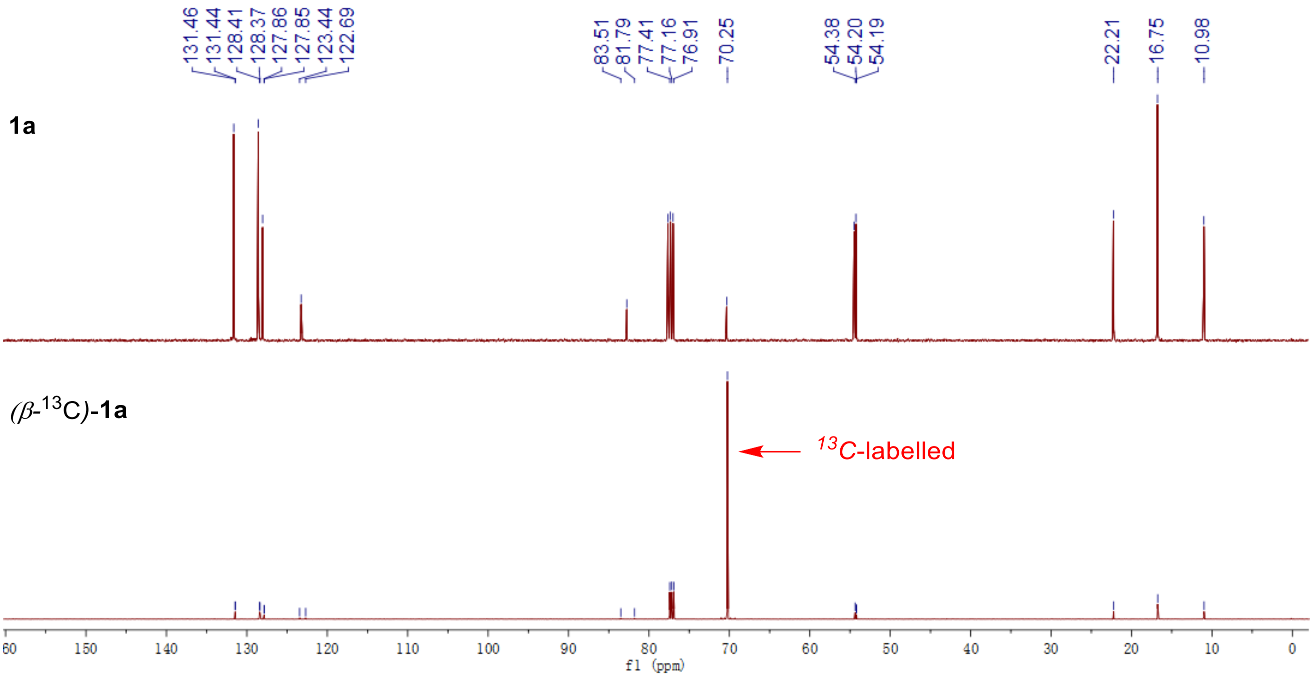

Supplementary Fig. 5 ^{13}C NMR spectrum of 1a and (β-^{13}C)-1a. .^{13}C-atom signal was presented at 70.3 ppm.

10.2 Procedure for the synthesis of ^{13}C-2a

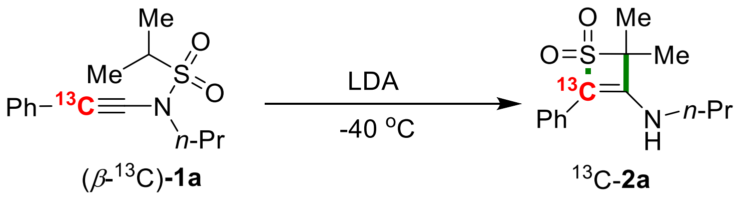

An oven-dried schlenk tube equipped with a magnetic stirrer bar was purged with argon three times. (β-^{13}C)-**1a** (53 mg, 0.2 mmol) was dissolved in 2 mL anhydrous THF and added by a syringe. The mixture was cooled to -40 °C and LDA (2 mol/L in THF, 0.15 mL, 0.3 mmol) was added dropwise. The reaction was stirred at -40 °C for another 1 h. MeOH (0.1 mL) was added to quench the reaction

and then the mixture was concentrated under vacuum to obtain the residue, which was further purified by silica gel column chromatography using ethyl acetate/petroleum ether (v/v, 1:2) as eluent to give ^{13}C-**2a** (37 mg, 70% yield) as a white solid.

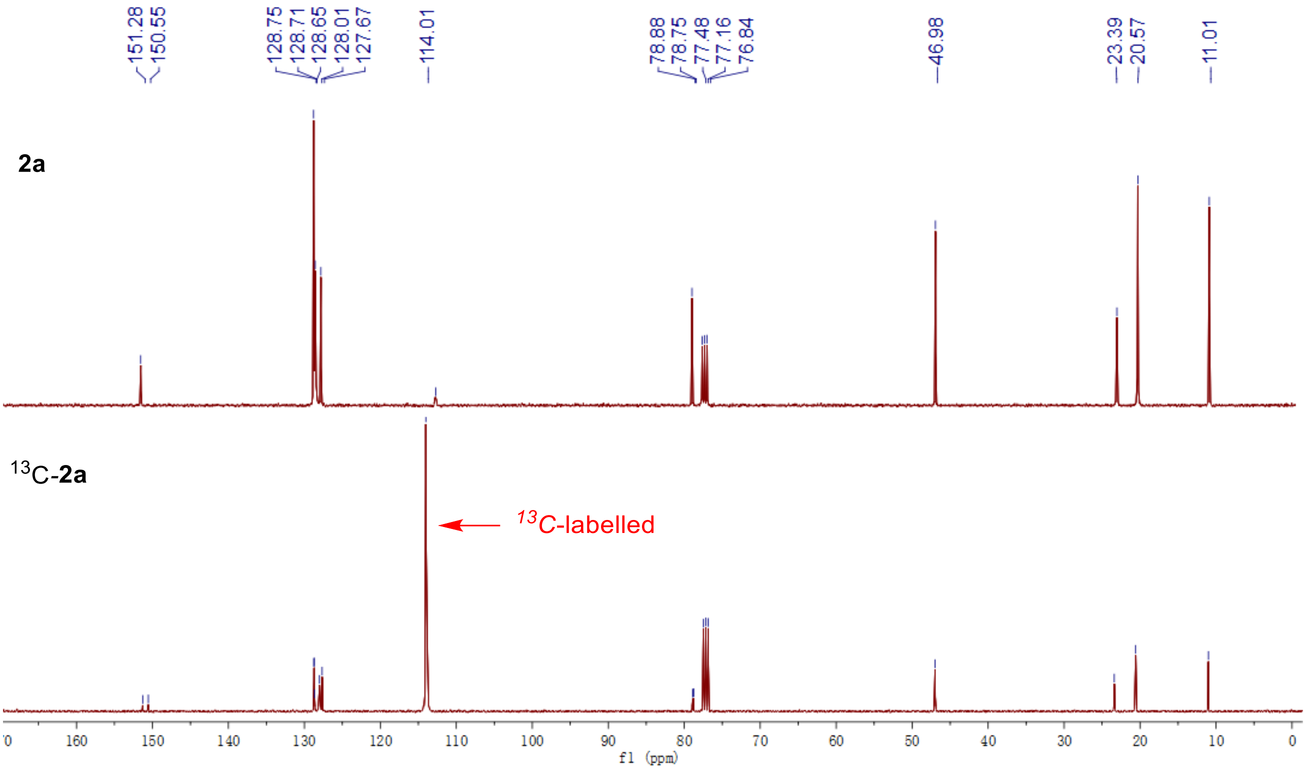

Supplementary Fig. 6 ^{13}C NMR spectrum of 2a and ^{13}C-2a. .^{13}C-atom signal was presented at 114.0 ppm.

10.3 Procedure for the synthesis of ^{13}C-3a

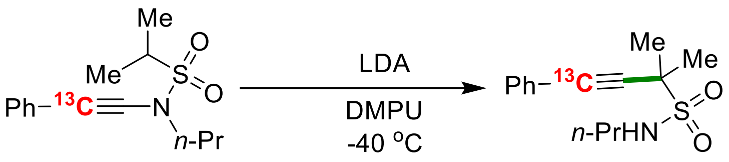

^{13}C-**1a** ^{13}C-**3a**

An oven-dried schlenk tube equipped with a magnetic stirrer bar was purged with argon three times. (β-^{13}C)-**1a** (53 mg, 0.2 mmol) was dissolved in 2 mL anhydrous THF and added by a syringe. DMPU (122 μL, 1 mmol) was added and the mixture was cooled to -40 °C. Subsequently, LDA (2 mol/L in THF, 0.15 mL, 0.3 mmol) was added dropwise. The reaction was stirred at -40 °C for another 1 h, and then MeOH (0.1 mL) was added to quench the reaction. The mixture was concentrated under vacuum to obtain the residue, which was further purified by silica gel column chromatography using

ethyl acetate/petroleum ether (v/v, 1:10) as eluent to give ^{13}C-**3a** (42 mg, 79% yield) as a white solid.

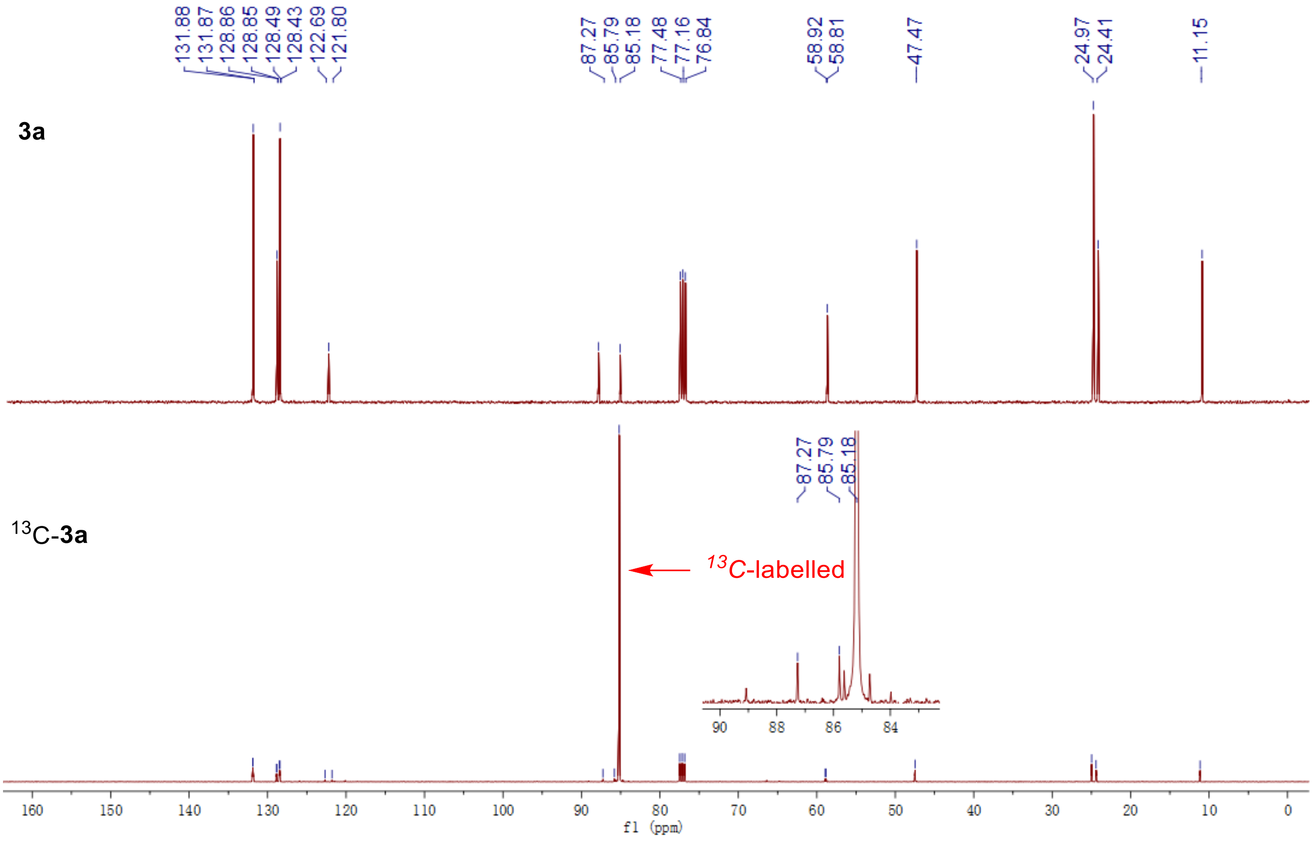

Supplementary Fig. 7 ^{13}C NMR spectrum of 3a and ^{13}C-3a. .^{13}C-atom signal was presented at 85.2 ppm.

11. Characterization of Products

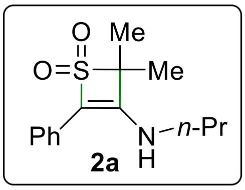

2,2-dimethyl-4-phenyl-3-(propylamino)-2*H*-thiete 1,1-dioxide

2a: Light yellow solid (38 mg, 72% yield); mp: 129 – 131 °C; **TLC** (PE: EA, 1:1, v/v): R_f = 0.38; **^{1}H NMR (400 MHz, $CDCl_3$):** δ 7.37 – 7.29 (m, 4H), 7.28 – 7.22 (m, 1H), 5.29 – 5.23 (m, 1H), 3.02 (q, *J* = 6.4 Hz, 2H), 1.66 (s, 6H), 1.48 – 1.35 (m, 2H), 0.78 (t, *J* = 7.2 Hz, 3H); **^{13}C NMR (100 MHz, $CDCl_3$):** δ 151.1, 128.6, 128.3, 128.2, 127.6, 113.10 78.8, 46.9, 23.2, 20.4, 11.0; **IR (KBr):** *ν* 3321, 3062, 2963, 2934, 2872, 1639, 1538, 1444, 1349, 1247, 1160, 1109, 761, 699, 606, 505 cm^{-1}; **HRMS (EI-TOF)** *m/z*: [M]$^+$ calcd. for $C_{14}H_{19}NO_2S$, 265.1136; found, 265.1137.

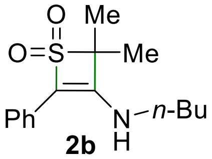

2b

3-(butylamino)-2,2-dimethyl-4-phenyl-2*H*-thiete 1,1-dioxide

2b: White solid (39 mg, 70% yield); mp: 137 – 139 °C; **TLC** (PE: EA, 1:1, v/v): R_f = 0.45; **^{1}H NMR (500 MHz, $CDCl_3$):** δ 7.37 – 7.31 (m, 4H), 7.28 – 7.23 (m, 1H), 4.95 (t, *J* = 5.5 Hz, 1H), 3.08 (q, *J* = 7.0 Hz, 2H), 1.68 (s, 6H), 1.46 – 1.38 (m, 2H), 1.27 – 1.17 (m, 2H), 0.81 (t, *J* = 7.5 Hz, 3H); **^{13}C NMR (125 MHz, $CDCl_3$):** δ 151.0, 128.7, 128.3, 128.1, 127.7, 113.8, 78.8, 45.1, 32.1, 20.5, 19.7, 13.7; **IR (KBr):** *ν* 3309, 3075, 2949, 2926, 2869, 1639, 1597, 1543, 1492, 1470, 1447, 1249, 1156, 1109, 843, 762, 701, 607, 508 cm^{-1}; **HRMS (EI-TOF)** *m/z*: $[M]^+$ calcd. for $C_{15}H_{21}NO_2S$, 279.1293; found, 279.1295.

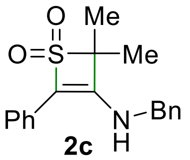

2c

3-(benzylamino)-2,2-dimethyl-4-phenyl-2*H*-thiete 1,1-dioxide

2c: White solid (53 mg, 85% yield); mp: 157 – 158 °C; **TLC** (PE: EA, 1:1, v/v): R_f = 0.50; **^{1}H NMR (500 MHz, $CDCl_3$):** δ 7.35 – 7.19 (m, 8H), 7.17 – 7.04 (m, 2H), 5.24 (t, *J* = 5.5 Hz, 1H), 4.26 (d, *J* = 5.5 Hz, 2H), 1.66 (s, 6H); **^{13}C NMR (125 MHz, $CDCl_3$):** δ 150.7, 137.1, 129.0, 128.7, 128.3, 128.1, 128.0, 127.9, 127.2, 114.9, 79.0, 49.0, 20.5; **IR (KBr):** *ν* 3291, 3057, 3028, 2932, 2866, 1631, 1540, 1495, 1444, 1335, 1257, 1098, 862, 758, 696, 606, 567, 508 cm^{-1}; **HRMS (EI-TOF)** *m/z*: $[M]^+$ calcd. for $C_{18}H_{19}NO_2S$, 313.1136; found, 313.1135.

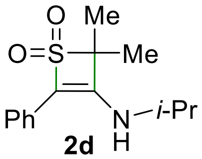

2d

3-(isopropylamino)-2,2-dimethyl-4-phenyl-2*H*-thiete 1,1-dioxide

2d: White solid (44 mg, 83% yield); mp: 200 – 202 °C; **TLC** (PE: EA, 1:1, v/v): R_f = 0.40;**^{1}H NMR (600 MHz, $CDCl_3$):** δ 7.40 – 7.31 (m, 4H), 7.29 – 7.23 (m, 1H), 4.79 (d, *J* = 7.8 Hz, 1H), 3.56 – 3.45 (m, 1H), 1.66 (s, 6H), 1.08 (d, *J* = 6.0 Hz, 6H); **^{13}C NMR (125 MHz, $CDCl_3$):** δ 150.1, 128.7, 128.5,

128.2, 127.8, 113.3, 78.9, 46.8, 23.3, 20.4; **IR (KBr):** *v* 3319, 3061, 2978, 2930, 2871, 1636, 1540, 1492, 1461, 1446, 1390, 1369, 1332, 1249, 1154, 1104, 828, 762, 701, 651, 609, 516, 440 cm^{-1}; **HRMS (EI-TOF)** *m/z*: $[M]^+$ calcd. for $C_{14}H_{19}NO_2S$, 265.1136; found, 265.1139.

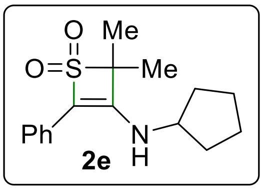

3-(cyclopentylamino)-2,2-dimethyl-4-phenyl-2*H*-thiete 1,1-dioxide

2e: White solid (43 mg, 74% yield); mp: 199 – 201 °C; **TLC** (PE: EA, 1:1, v/v): R_f= 0.45;**^{1}H NMR (500 MHz, $CDCl_3$):** δ 7.39 – 7.31 (m, 4H), 7.29 – 7.24 (m, 1H), 4.77 (d, *J* = 8.0 Hz, 1H), 3.76 – 3.65 (m, 1H), 1.76 – 1.70 (m, 2H), 1.67 (s, 6H), 1.65 – 1.57 (m, 2H), 1.51 – 1.35 (m, 4H); **^{13}C NMR (125 MHz, $CDCl_3$):** δ 150.3, 128.7, 128.4, 128.3, 127.7, 114.0, 78.9, 56.4, 34.0, 23.9, 20.6; **IR (KBr):** *v* 3312, 3062, 2967, 2868, 1637, 1544, 1443, 1343, 1252, 1156, 1110, 1098, 761, 701, 603, 494 cm^{-1}; **HRMS (EI-TOF)** *m/z*: $[M]^+$ calcd. for $C_{16}H_{21}NO_2S$, 291.1293; found, 291.1295.

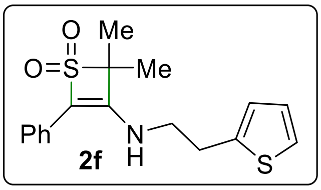

2,2-dimethyl-4-phenyl-3-((2-(thiophen-2-yl)ethyl)amino)-2*H*-thiete 1,1-dioxide

2f: White solid (56 mg, 84% yield); mp: 123 – 125 °C; **TLC** (PE: EA, 1:1, v/v): R_f= 0.40; **^{1}H NMR (400 MHz, $CDCl_3$):** δ 7.35 – 7.24 (m, 5H), 7.12 (dd, J_1 = 5.2 Hz, J_2 = 1.2 Hz, 1H), 6.89 (dd, J_1 = 5.2 Hz, J_2 = 3.6 Hz, 1H), 6.62 (d, *J* = 3.6 Hz, 1H), 5.30 (t, *J* = 6.4 Hz, 1H), 3.32 (q, *J* = 6.4 Hz, 2H), 2.87 (t, *J* = 6.4 Hz, 2H), 1.62 (s, 6H); **^{13}C NMR (125 MHz, $CDCl_3$):** δ 150.7, 139.7, 128.8, 128.4, 128.0, 127.9, 127.1, 125.8, 124.3, 113.8, 79.0, 46.2, 30.2, 20.3; **IR (KBr):** *v* 3310, 3061, 2966, 2937, 1648, 1594, 1543, 1452, 1440, 1347, 1248, 1158, 1108, 842, 753, 701, 598, 571, 500 cm^{-1}; **HRMS (EI-TOF)** *m/z*: $[M]^+$ calcd. for $C_{17}H_{19}NO_2S_2$, 333.0857; found, 333.0858.

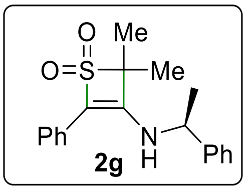

(*S*)-2,2-dimethyl-4-phenyl-3-((1-phenylethyl)amino)-2*H*-thiete 1,1-dioxide

2g: Colourless gum (50 mg, 77% yield); **TLC** (PE: EA, 1:1, v/v): R_f = 0.46; **^{1}H NMR (500 MHz, $CDCl_3$):** δ 7.24 – 7.11 (m, 8H), 6.90 – 6.84 (m, 2H), 5.52 (d, *J* = 8.5 Hz, 1H), 4.40 (quint, *J* = 7.0 Hz, 1H), 1.64 (s, 3H), 1.61 (s, 3H), 1.35 (d, *J* = 7.0 Hz, 3H); **^{13}C NMR (125 MHz, $CDCl_3$):** δ 150.0, 142.8, 129.2, 128.6, 128.3, 128.0, 127.9, 127.4, 125.4, 114.4, 79.0, 54.2, 23.8, 20.2; **IR (KBr):** *v* 3331, 3061, 2982, 2927, 2868, 1643, 1532, 1444, 1252, 1097, 841, 761, 698, 602, 500 cm^{-1}; **HRMS (EI-TOF)** *m/z*: $[M]^+$ calcd. for $C_{19}H_{21}NO_2S$, 327.1293; found, 327.1298.

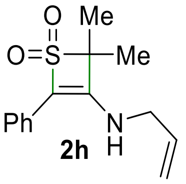

2h

3-(allylamino)-2,2-dimethyl-4-phenyl-2*H*-thiete 1,1-dioxide

2h: Light yellow solid (41 mg, 78% yield); mp: 112 – 114 °C; **TLC** (PE: EA, 1:1, v/v): R_f = 0.43; **^{1}H NMR (400 MHz, $CDCl_3$):** δ 7.42 – 7.18 (m, 5H), 5.82 – 5.65 (m, 1H), 5.23 – 4.56 (m, 3H), 3.80 – 3.65 (m, 2H), 1.68 (s, 6H); **^{13}C NMR (100 MHz, $CDCl_3$):** δ 150.8, 133.5, 128.7, 128.1 (two peaks: 128.14 and 128.10), 127.8, 117.4, 114.5, 79.0, 47.3, 20.6; **IR (KBr):** *v* 3316, 3060, 2985, 2925, 1651, 1631, 1538, 1491, 1434, 1343, 1250, 1099, 903, 755, 699, 642, 603, 544, 488 cm^{-1}; **HRMS (EI-TOF)** *m/z*: $[M]^+$ calcd. for $C_{14}H_{17}NO_2S$, 263.0980; found, 263.0988.

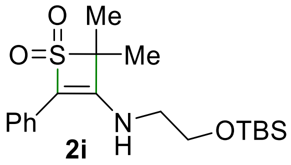

2i

3-((2-((*tert*-butyldimethylsilyl)oxy)ethyl)amino)-2,2-dimethyl-4-phenyl-2*H*-thiete 1,1-dioxide

2i: White solid (62 mg, 81% yield); mp: 106 – 108 °C; **TLC** (PE: EA, 1:1, v/v): R_f = 0.11; **^{1}H NMR (500 MHz, $CDCl_3$):** δ 7.37 – 7.29 (m, 4H), 7.26 – 7.21 (m, 1H), 5.14 (t, *J* = 5.5 Hz, 1H), 3.63 (t, *J* = 5.5 Hz, 2H), 3.22 (q, *J* = 5.5 Hz, 2H), 1.68 (s, 6H), 0.87 (s, 9H), 0.03 (s, 6H); **^{13}C NMR (125 MHz, $CDCl_3$):** δ 150.7, 128.8, 128.3, 127.5 (two peaks: 127.52 and 127.46), 114.8, 78.8, 61.4, 46.7, 25.9, 20.6, 18.2, -5.4; **IR (KBr):** *v* 3325, 3063, 2951, 2925, 2851, 1642, 1545, 1461, 1443, 1359, 1254, 1157, 1105, 836, 781, 753, 701, 607, 579, 505 cm^{-1}; **HRMS (EI-TOF)** *m/z*: $[M]^+$ calcd. for $C_{19}H_{31}NO_3SSi$, 381.1794; found, 381.1798.

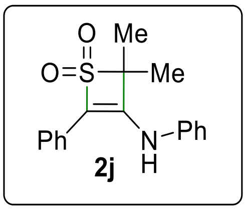

2,2-dimethyl-4-phenyl-3-(phenylamino)-2*H*-thiete 1,1-dioxide

2j: Yellow solid (55 mg, 92% yield); mp: 187 – 189 °C; **TLC** (PE: EA, 1:1, v/v): R_f= 0.51; **^{1}H NMR (400 MHz, $CDCl_3$):** δ 7.18 – 7.05 (m, 6H), 6.95 – 6.89 (m, 2H), 6.88 – 6.81 (m, 2H), 6.66 (s, 1H), 1.75 (s, 6H); **^{13}C NMR (100 MHz, $CDCl_3$):** δ 145.4, 137.6, 128.9, 128.1, 128.0, 127.7, 127.5, 125.2, 122.4, 118.5, 80.4, 20.5; **IR (KBr):** *v* 3287, 3060, 2928, 2880, 1644, 1593, 1538, 1494, 1445, 1351, 1257, 1158, 1126, 1098, 764, 695, 630, 594, 558, 530, 488 cm^{-1}; **HRMS (EI-TOF)** *m/z*: $[M]^+$ calcd. for $C_{17}H_{17}NO_2S$, 299.0980; found, 299.0984.

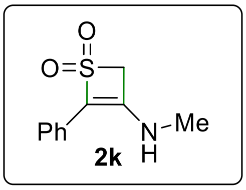

3-(methylamino)-4-phenyl-2*H*-thiete 1,1-dioxide

2k: White solid (23 mg, 54% yield); mp: 197 – 199 °C; **TLC** (PE: EA, 1:1, v/v): R_f= 0.18; **^{1}H NMR (500 MHz, DMSO-*d*$_6$):** δ 7.62 (q, *J* = 4.5 Hz, 1H), 7.39 – 7.35 (m, 2H), 7.29 (d, *J* = 7.0 Hz, 2H), 7.17 (t, *J* = 7.0 Hz, 1H), 4.65 (s, 2H), 2.85 (d, *J* = 4.5 Hz, 3H); **^{13}C NMR (125 MHz, DMSO-*d*$_6$):** δ 143.6, 129.4, 128.9, 125.5, 123.6, 114.9, 66.3, 32.0; **IR (KBr):** *v* 3339, 3060, 2982, 2932, 1651, 1512, 1398, 1253, 1191, 1106, 758, 750, 688, 570, 462, 412 cm^{-1}; **HRMS (EI-TOF)** *m/z*: $[M]^+$ calcd. for $C_{10}H_{11}NO_2S$, 209.0510; found, 209.0516.

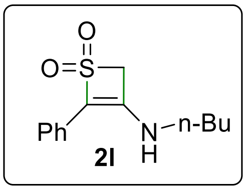

3-(butylamino)-4-phenyl-2*H*-thiete 1,1-dioxide

2l: White solid (28 mg, 55% yield); mp: 133 – 134 °C; **TLC** (PE: EA, 2:1, v/v): R_f= 0.15; **^{1}H NMR (600 MHz, $CDCl_3$):** δ 7.42 – 7.19 (m, 5H), 5.12 (t, *J* = 6.6 Hz, 1H), 4.39 (s, 2H), 3.11 (q, *J* = 6.6 Hz, 2H), 1.57 – 1.48 (m, 2H), 1.39 – 1.29 (m, 2H), 0.92 (t, *J* = 7.2 Hz, 3H); **^{13}C NMR (100 MHz, $CDCl_3$):** δ 142.1, 129.2, 128.6, 127.2, 125.5, 119.0, 66.4, 46.3, 33.0, 19.8, 13.8; **IR (KBr):** *v* 3314, 3053, 2950,

2930, 2867, 1656, 1597, 1510, 1432, 1378, 1267, 1176, 1116, 761, 738, 696, 587, 456 cm^{-1}; **HRMS (EI-TOF)** *m/z*: $[M]^+$ calcd. for $C_{13}H_{17}NO_2S$, 251.0980; found, 251.0982.

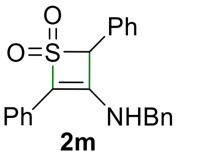

2m

3-(benzylamino)-2,4-diphenyl-2*H*-thiete 1,1-dioxide

2m: White solid (34 mg, 47% yield); mp: 183 – 185 °C; **TLC** (PE: EA, 2:1, v/v): R_f= 0.33; **^{1}H NMR (400 MHz, DMSO-*d*$_6$):** δ 8.23 (s, 1H), 7.50 – 7.38 (m, 9H), 7.33 – 7.19 (m, 4H), 7.12 – 7.03 (m, 2H), 6.12 (s, 1H), 4.22 – 3.93 (m, 2H); **^{13}C NMR (100 MHz, DMSO-*d*$_6$):** δ 144.7, 138.4, 130.8, 129.2, 128.9, 128.8, 128.5, 127.3, 127.2, 126.5, 124.4, 117.9, 81.9, 48.7; **IR (KBr):** *v* 3365, 3030, 2947, 2913, 1634, 1600, 1503, 1452, 1443, 1403, 1345, 1260, 1139, 1097, 763, 734, 714, 691, 575, 496 cm^{-1}; **HRMS (EI-TOF)** *m/z*: $[M]^+$ calcd. for $C_{22}H_{19}NO_2S$, 361.1136; found, 361.1132.

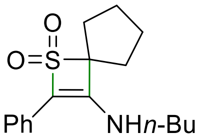

2n

3-(butylamino)-2-phenyl-1-thiaspiro[3.4]oct-2-ene 1,1-dioxide

2n: White solid (26 mg, 43% yield); mp: 105 – 107 °C; **TLC** (PE: EA, 2:1, v/v): R_f= 0.27; **^{1}H NMR (500 MHz, $CDCl_3$):** δ 7.39 – 7.23 (m, 5H), 4.67 (t, *J* = 6.0 Hz, 1H), 3.09 (q, *J* = 7.0 Hz, 2H), 2.71 – 2.62 (m, 2H), 2.09 – 2.01 (m, 2H), 1.81 – 1.74 (m, 4H), 1.49 – 1.40 (m, 2H), 1.29 – 1.19 (m, 2H), 0.83 (t, *J* = 7.5 Hz, 3H); **^{13}C NMR (125 MHz, $CDCl_3$):** δ 148.9, 128.7, 128.4, 128.0, 127.6, 115.4, 89.0, 45.1, 32.2, 31.4, 26.5, 19.8, 13.7; **IR (KBr):** *v* 3312, 3052, 2962, 2932, 2869, 1643, 1595, 1474, 1442, 1243, 1111, 950, 899, 758, 699, 537, 475 cm^{-1}; **HRMS (EI-TOF)** *m/z*: $[M]^+$ calcd. for $C_{17}H_{23}NO_2S$, 305.1449; found, 305.1445.

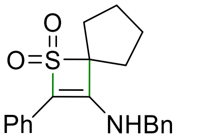

2o

3-(benzylamino)-2-phenyl-1-thiaspiro[3.4]oct-2-ene 1,1-dioxide

2o: White solid (55 mg, 81% yield); mp: 170 – 172 °C; **TLC** (PE: EA, 2:1, v/v): R_f= 0.27; **^{1}H NMR**

(500 MHz, CDCl₃): δ 7.32 – 7.19 (m, 8H), 7.14 – 7.05 (m, 2H), 5.36 – 5.15 (m, 1H), 4.25 (d, *J* = 6.0 Hz, 2H), 2.66 – 2.55 (m, 2H), 2.11 – 2.01 (m, 2H), 1.77 – 1.63 (m, 4H); **^{13}C NMR (125 MHz, $CDCl_3$):** δ 148.7, 137.2, 129.0, 128.7, 128.2, 128.0 (two peaks: 128.03 and 128.02), 127.8, 127.1, 116.1, 89.2, 48.9, 31.3, 26.4; **IR (KBr):** *v* 3363, 3050, 2967, 2930, 2868, 1631, 1505, 1452, 1442, 1414, 1359, 1241, 1187, 1110, 960, 763, 733, 695, 628, 575, 525, 473 cm^{-1}; **HRMS (EI-TOF)** *m/z*: $[M]^+$ calcd. for $C_{20}H_{21}NO_2S$, 339.1293; found, 339.1295.

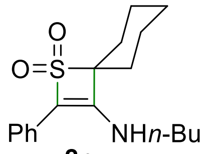

2p

3-(butylamino)-2-phenyl-1-thiaspiro[3.5]non-2-ene 1,1-dioxide

2p: White solid (22 mg, 34% yield); mp: 128– 130 °C; **TLC** (PE: EA, 2:1, v/v): R_f= 0.30; **^{1}H NMR (400 MHz, $CDCl_3$):** δ 7.41 – 7.20 (m, 5H), 4.62 (s, 1H), 3.10 – 2.96 (m, 2H), 2.48 – 2.30 (m, 2H), 1.87 – 1.63 (m, 7H), 1.40 – 1.31 (m, 2H), 1.26 – 1.10 (m, 3H), 0.78 (t, *J* = 7.2 Hz, 3H); **^{13}C NMR (100 MHz, $CDCl_3$):** δ 150.4, 128.9, 128.6, 128.4, 127.8, 113.5, 83.6, 45.0, 32.0, 30.5, 24.7, 24.2, 19.7, 13.7; **IR (KBr):** *v* 3301, 3059, 2932, 2857, 1647, 1599, 1547, 1445, 1250, 1148, 1101, 760, 698, 529 cm^{-1}.; **HRMS (EI-TOF)** *m/z*: $[M]^+$ calcd. for $C_{18}H_{25}NO_2S$, 319.1606; found, 319.1609.

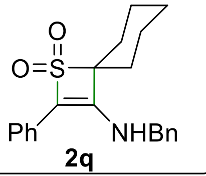

2q

3-(benzylamino)-2-phenyl-1-thiaspiro[3.5]non-2-ene 1,1-dioxide

2q: White solid (52 mg, 74% yield); mp: 175 – 177 °C; **TLC** (PE: EA, 2:1, v/v): R_f= 0.30; **^{1}H NMR (500 MHz, $CDCl_3$):** δ 7.31 – 7.21 (m, 8H), 7.08 – 7.04 (m, 2H), 4.95 (t, *J* = 6.0 Hz, 1H), 4.22 (d, *J* = 6.0 Hz, 2H), 2.47 – 2.37 (m, 2H), 1.83 – 1.60 (m, 7H), 1.22 – 1.09 (m, 1H); **^{13}C NMR (150 MHz, $CDCl_3$):** δ 150.2, 137.2, 129.0 (two peaks: 128.99 and 128.95), 128.6, 128.0 (three peaks: 128.03, 128.00 and 127.97), 127.2, 114.7, 83.8, 49.0, 30.5, 24.7, 24.2; **IR (KBr):** *v* 3289, 3080, 2943, 2926, 2856, 1640 1555, 1495, 1444, 1344, 1245, 1146, 1102, 1028, 962, 942, 760, 729, 699, 661, 630, 567, 516, 452 cm^{-1}; **HRMS (EI-TOF)** *m/z*: $[M]^+$ calcd. for $C_{21}H_{23}NO_2S$, 353.1449; found, 353.1445.

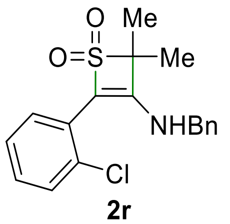

2r

3-(benzylamino)-4-(2-chlorophenyl)-2,2-dimethyl-2*H*-thiete 1,1-dioxide

2r: White solid (60 mg, 86% yield); mp: 194 – 196 °C; **TLC** (PE: EA, 2:1, v/v): R_f = 0.20; **^{1}H NMR (500 MHz, $CDCl_3$):** δ 7.40 – 7.32 (m, 2H), 7.27 – 7.21 (m, 4H), 7.18 – 7.12 (m, 1H), 7.08 – 6.97 (m, 2H), 5.21 (t, *J* = 5.0 Hz, 1H), 4.15 (d, *J* = 5.0 Hz, 2H), 1.68 (s, 6H); **^{13}C NMR (125 MHz, $CDCl_3$):** δ 152.5, 137.1, 132.2, 130.2, 129.7, 128.9, 128.0, 127.3, 126.9 (two peaks: 126.89 and 126.85), 111.4, 79.0, 48.5, 20.4; **IR (KBr):** *ν* 3322, 3060, 2999, 2929, 2871, 1658, 1597, 1539, 1452, 1345, 1252, 1160, 1103, 747, 729, 586, 467 cm^{-1}; **HRMS (EI-TOF)** *m/z*: $[M]^+$ calcd. for $C_{18}H_{18}ClNO_2S$, 347.0747; found, 347.0749.

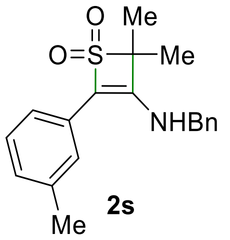

2s

3-(benzylamino)-2,2-dimethyl-4-(*m*-tolyl)-2*H*-thiete 1,1-dioxide

2s: White solid (48 mg, 74% yield); mp: 145 – 147 °C; **TLC** (PE: EA, 2:1, v/v): R_f = 0.20; **^{1}H NMR (500 MHz, $CDCl_3$):** δ 7.34 – 7.26 (m, 3H), 7.20 – 7.03 (m, 6H), 5.11 (br, 1H), 4.27 (d, *J* = 6.0 Hz, 2H), 2.26 (s, 3H), 1.67 (s, 6H); **^{13}C NMR (125 MHz, $CDCl_3$):** δ 150.6, 138.4, 137.3, 129.0, 128.9, 128.7, 128.5, 127.9, 127.8, 127.1, 125.5, 114.8, 78.9, 48.8, 21.4, 20.4; **IR (KBr):** *ν* 3319, 3061, 2989, 2919, 2865, 1658, 1602, 1583, 1541, 1496, 1443, 1346, 1250, 1158, 1103, 947, 776, 735, 697, 630, 590, 458, 440 cm^{-1}; **HRMS (EI-TOF)** *m/z*: $[M]^+$ calcd. for $C_{19}H_{21}NO_2S$, 327.1293; found, 327.1295.

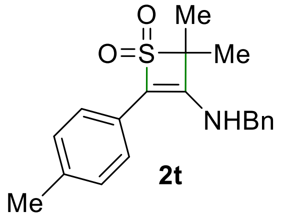

2t

3-(benzylamino)-2,2-dimethyl-4-(*p*-tolyl)-2*H*-thiete 1,1-dioxide

2t: White solid (52 mg, 79% yield); mp: 147 – 149 °C; **TLC** (PE: EA, 2:1, v/v): R_f= 0.20; **^{1}H NMR (500 MHz, $CDCl_3$):** δ 7.30 – 7.24 (m, 3H), 7.19 – 7.14 (m, 2H), 7.11 – 7.05 (m, 4H), 5.28 (t, *J* = 6.0 Hz, 1H), 4.23 (d, *J* = 6.0 Hz, 2H), 2.31 (s, 3H), 1.63 (s, 6H); **^{13}C NMR (125 MHz, $CDCl_3$):** δ 150.4, 137.8, 137.3, 129.3, 128.9, 128.4, 127.9, 127.2, 124.9, 114.7, 78.9, 48.9, 21.4, 20.4. **IR (KBr)** *v* 3313, 3078, 3049, 3026, 2993, 2974, 2917, 2871, 1651, 1547, 1449, 1349, 1251, 1163, 1105, 808, 730, 691, 607, 584, 504, 458 cm^{-1}; **HRMS (EI-TOF)** *m/z*: $[M]^+$ calcd. for $C_{19}H_{21}NO_2S$, 327.1293; found, 327.1299.

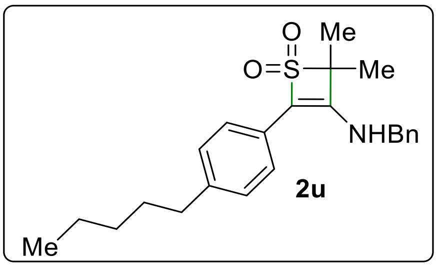

3-(benzylamino)-2,2-dimethyl-4-(4-pentylphenyl)-2*H*-thiete 1,1-dioxide

2u: White solid (57 mg, 74% yield); mp: 99 – 101 °C; **TLC** (PE: EA, 2:1, v/v): R_f= 0.28; **^{1}H NMR (400 MHz, $CDCl_3$):** δ 7.35 – 7.25 (m, 3H), 7.24 – 7.16 (m, 2H), 7.16 – 7.05 (m, 4H), 5.28 – 5.00 (m, 1H), 4.26 (d, *J* = 5.2 Hz, 2H), 2.55 (t, *J* = 7.6 Hz, 2H), 1.66 (s, 6H), 1.61 – 1.51 (m, 2H), 1.38 – 1.23 (m, 4H), 0.88 (t, *J* = 6.8 Hz, 3H); **^{13}C NMR (100 MHz, $CDCl_3$):** δ 150.3, 142.9, 137.3, 128.9, 128.7, 128.2, 128.0, 127.3, 125.1, 115.2, 78.8, 49.0, 35.8, 31.5, 31.1, 22.6, 20.5, 14.2; **IR (KBr):** *v* 3265, 3049, 2926, 2860, 1644, 1548, 1454, 1446, 1369, 1342, 1249, 1147, 1098, 970, 836, 737, 694, 608, 568, 477, 465 cm^{-1}; **HRMS (EI-TOF)** *m/z*: $[M]^+$ calcd. for $C_{23}H_{29}NO_2S$, 383.1919; found, 383.1917.

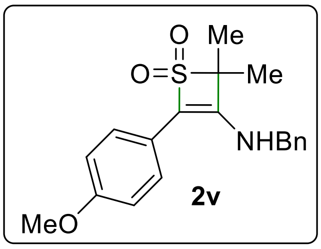

3-(benzylamino)-4-(4-methoxyphenyl)-2,2-dimethyl-2*H*-thiete 1,1-dioxide

2v: White solid (48 mg, 70% yield); mp: 144 – 145 °C; **TLC** (PE: EA, 2:1, v/v): R_f= 0.13; **^{1}H NMR (500 MHz, $CDCl_3$):** δ 7.29 – 7.22 (m, 3H), 7.20 – 7.14 (m, 2H), 7.08 – 7.02 (m, 2H), 6.77 (d, *J* = 8.5 Hz, 2H), 5.48 – 5.35 (m, 1H), 4.19 (d, *J* = 6.0 Hz, 2H), 3.76 (s, 3H), 1.62 (s, 6H); **^{13}C NMR (125 MHz,**

CDCl3): δ 159.4, 150.4, 137.4, 130.3, 128.8, 127.8, 127.1, 120.0, 114.2, 114.0, 78.7, 55.4, 48.6, 20.3; **IR (KBr):** *v* 3316, 3065, 3028, 2961, 2930, 2837, 1657, 1637, 1610, 1546, 1512, 1454, 1351, 1288, 1248, 1174, 1160, 1106, 1029, 823, 731, 695, 626, 608, 584, 521 cm^{-1}; **HRMS (EI-TOF)** *m/z*: $[M]^+$ calcd. for $C_{19}H_{21}NO_3S$, 343.1242; found, 343.1245.

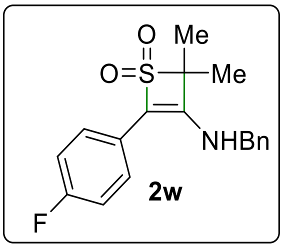

3-(benzylamino)-4-(4-fluorophenyl)-2,2-dimethyl-2*H*-thiete 1,1-dioxide

2w: White solid (52 mg, 78% yield); mp: 148 – 150 °C; **TLC** (PE: EA, 2:1, v/v): R_f= 0.20; **^{1}H NMR (500 MHz, CDCl3):** δ 7.32 – 7.22 (m, 5H), 7.11 – 7.04 (m, 2H), 6.98 – 6.92 (m, 2H), 5.26 – 5.10 (m, 1H), 4.22 (d, *J* = 6.0 Hz, 2H), 1.67 (s, 6H); **^{13}C NMR (125 MHz, CDCl3):** δ 162.4 (d, *J* = 248.5 Hz), 150.8, 136.9, 130.6 (d, *J* = 7.2 Hz), 129.0, 128.1, 127.1, 123.8 (d, *J* = 3.4 Hz), 115.8 (d, *J* = 21.8 Hz), 113.9, 79.0, 48.9, 20.5; **^{19}F NMR (376 MHz, CDCl3):** δ -112.47; **IR (KBr):** *v* 3293, 3061, 3033, 2931, 2877, 1632, 1539, 1505, 1469, 1455, 1336, 1254, 1223, 1163, 1103, 834, 794, 753, 697, 627, 606, 519, 490, 468 cm^{-1}; **HRMS (EI-TOF)** *m/z*: $[M]^+$ calcd. for $C_{18}H_{18}FNO_2S$, 331.1042; found, 331.1040.

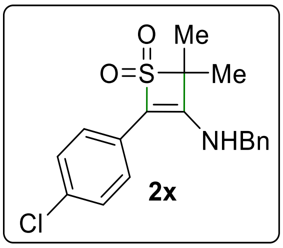

3-(benzylamino)-4-(4-chlorophenyl)-2,2-dimethyl-2*H*-thiete 1,1-dioxide

2x: White solid (59 mg, 85% yield); mp: 166 – 168 °C; **TLC** (PE: EA, 2:1, v/v): R_f= 0.20; **^{1}H NMR (600 MHz, CDCl3):** δ 7.30 – 7.24 (m, 3H), 7.20 (d, *J* = 8.4 Hz, 2H), 7.15 (d, *J* = 8.4 Hz, 2H), 7.08 – 7.03 (m, 2H), 5.53 (t, *J* = 6.0 Hz, 1H), 4.21 (d, *J* = 6.0 Hz, 2H), 1.64 (s, 6H); **^{13}C NMR (150 MHz, CDCl3):** δ 151.2, 136.9, 133.8, 129.7, 128.9, 128.8, 128.0, 126.9, 126.3, 113.3, 79.2, 48.8, 20.4; **IR (KBr):** *v* 3225, 3063, 3033, 2914, 1656, 1545, 1491, 1454, 1436, 1344, 1227, 1160, 1100, 1017, 814, 737, 723, 696, 632, 598, 512, 465 cm^{-1}; **HRMS (EI-TOF)** *m/z*: $[M]^+$ calcd. for $C_{18}H_{18}ClNO_2S$, 347.0747; found, 347.0744.

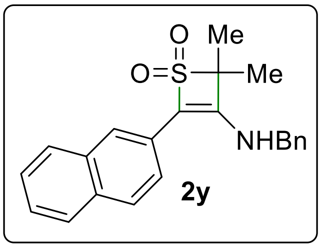

3-(benzylamino)-2,2-dimethyl-4-(naphthalen-2-yl)-2*H*-thiete 1,1-dioxide

2y: White solid (58 mg, 80% yield); mp: 170 – 172 °C; **TLC** (PE: EA, 2:1, v/v): $R_f = 0.19$;
^{1}H NMR (400 MHz, $CDCl_3$): δ 7.78 – 7.62 (m, 4H), 7.47 – 7.40 (m, 2H), 7.36 (dd, J_1 = 8.4 Hz, J_2 = 1.6 Hz, 1H), 7.26 – 7.19 (m, 3H), 7.08 – 7.01 (m, 2H), 5.48 (t, J = 6.0 Hz, 1H), 4.25 (d, J = 6.0 Hz, 2H), 1.66 (s, 6H); **^{13}C NMR (100 MHz, $CDCl_3$):** δ 151.0, 137.1, 133.1, 132.6, 128.9, 128.4, 128.0, 127.8, 127.4, 127.0, 126.6, 126.4, 125.8, 125.3, 114.6, 79.1, 48.9, 20.5; **IR (KBr):** *v* 3286, 3071, 3055, 2923, 1645, 1594, 1550, 1499, 1452, 1346, 1253, 1164, 1095, 943, 861, 821, 750, 696, 607, 475 cm^{-1}; **HRMS (EI-TOF)** *m/z*: $[M]^+$ calcd. for $C_{22}H_{21}NO_2S$, 363.1293; found, 363.1299.

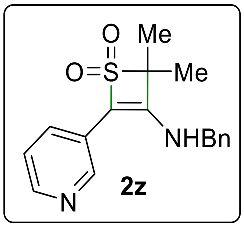

3-(benzylamino)-2,2-dimethyl-4-(pyridin-3-yl)-2*H*-thiete 1,1-dioxide

2z: White solid (53 mg, 85% yield); mp: 169 – 171 °C; **TLC** (PE: EA, 1:1, v/v): $R_f = 0.10$; **^{1}H NMR (600 MHz, $CDCl_3$):** δ 8.50 (d, J = 1.2 Hz, 1H), 8.40 (dd, J_1 = 4.2 Hz, J_2 = 1.2 Hz, 1H), 7.60 – 7.56 (m, 1H), 7.31 – 7.23 (m, 3H), 7.16 (dd, J_1 = 7.8 Hz, J_2 = 4.8 Hz, 1H), 7.06 (d, J = 6.0 Hz, 2H), 5.69 (t, J = 6.0 Hz, 1H), 4.25 (d, J = 6.0 Hz, 2H), 1.71 (s, 6H); **^{13}C NMR (150 MHz, $CDCl_3$):** δ 152.0, 149.1, 148.6, 136.4, 135.7, 129.1, 128.3, 127.0, 124.7, 123.5, 111.4, 79.6, 49.1, 20.5; **IR (KBr):** *v* 3297, 3052, 3035, 2933, 2867, 1636, 1588, 1538, 1455, 1412, 1339, 1255, 1162, 1108, 1025, 911, 862, 809, 755, 710, 693, 630, 607, 568, 482cm^{-1}; **HRMS (EI-TOF)** *m/z*: $[M]^+$ calcd. for $C_{17}H_{18}N_2O_2S$, 314.1089; found, 314.1083.

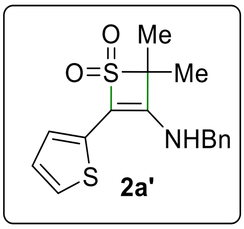

3-(benzylamino)-2,2-dimethyl-4-(thiophen-2-yl)-2*H*-thiete 1,1-dioxide

2a': White solid (54 mg, 85% yield); mp: 154 – 156 °C; **TLC** (PE: EA, 2:1, v/v): R_f = 0.19; **^{1}H NMR (500 MHz, $CDCl_3$):** δ 7.34 – 7.25 (m, 4H), 7.16 – 7.11 (m, 2H), 7.02 (dd, J_1 = 3.5 Hz, J_2 = 1.0 Hz, 1H), 6.97 (dd, J_1 = 5.0 Hz, J_2 = 3.5 Hz, 1H), 5.16 (t, J = 5.5 Hz, 1H), 4.35 (d, J = 5.5 Hz, 2H), 1.67 (s, 6H); **^{13}C NMR (125 MHz, $CDCl_3$):** δ 151.4, 137.0, 129.1, 128.4, 128.2, 127.4, 127.3 (two peaks: 127.33 and 127.30), 126.5, 108.6, 78.9, 49.0, 20.5; **IR (KBr):** *v* 3315, 3052, 3033, 2930, 2865, 1652, 1551, 1453, 1359, 1253, 1163, 1106, 952, 803, 732, 693, 615, 593, 438 cm^{-1}; **HRMS (EI-TOF)** *m/z*: $[M]^+$ calcd. for $C_{16}H_{17}NO_2S_2$, 319.0701; found, 319.0707.

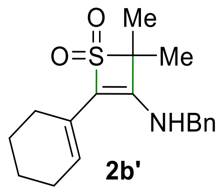

2b'

3-(benzylamino)-4-(cyclohex-1-en-1-yl)-2,2-dimethyl-2*H*-thiete 1,1-dioxide

2b': Yellow solid (39 mg, 62% yield); mp: 143 – 145 °C; **TLC** (PE: EA, 2:1, v/v): R_f = 0.22; **^{1}H NMR (400 MHz, $CDCl_3$):** δ 7.41 – 7.19 (m, 5H), 5.76 (t, J = 4.0 Hz, 1H), 4.83 (t, J = 5.6 Hz, 1H), 4.37 (d, J = 5.6 Hz, 2H), 2.14 – 2.03 (m, 4H), 1.65 (s, 6H), 1.61 – 1.49 (m, 4H); **^{13}C NMR (100 MHz, $CDCl_3$):** δ 148.8, 137.5, 129.1, 128.3, 128.2, 127.1, 126.3, 118.7, 78.1, 48.7, 27.7, 25.4, 22.2, 21.6, 20.8; **IR (KBr):** *v* 3353, 3052, 3030, 2926, 1860, 1659, 1632, 1535, 1452, 1438, 1364, 1326, 1251, 1161, 1105, 960, 802. 732, 695, 594, 505, 457 cm^{-1}; **HRMS (EI-TOF)** *m/z*: $[M]^+$ calcd. for $C_{18}H_{23}NO_2S$, 317.1449; found, 317.1444.

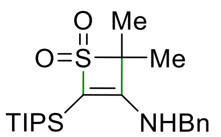

2c'

3-(benzylamino)-2,2-dimethyl-4-(triisopropylsilyl)-2*H*-thiete 1,1-dioxide

2c': White solid (46 mg, 58% yield); mp: 156 – 158 °C; **TLC** (PE: EA, 2:1, v/v): R_f = 0.47; **^{1}H NMR (400 MHz, $CDCl_3$):** δ 7.43 – 7.32 (m, 3H), 7.29 – 7.24 (m, 2H), 4.64 (t, J = 6.0 Hz, 1H), 4.39 (d, J = 6.0 Hz, 2H), 1.71 (s, 6H), 1.23 – 1.11 (m, 21H); **^{13}C NMR (125 MHz, $CDCl_3$):** δ 164.6, 137.1, 129.2, 128.4, 127.0, 114.3, 81.2, 49.2, 21.6, 18.8, 12.1; **IR (KBr):** *v* 3270, 3076, 2955, 2865, 1598, 1579, 1454, 1435, 1358, 1244, 1095, 1016, 882, 732, 670, 624, 590, 568, 445 cm^{-1}; **HRMS (EI-TOF)** *m/z*:

[M]$^+$ calcd. for $C_{21}H_{35}NO_2SSi$, 393.2158; found, 393.2154.

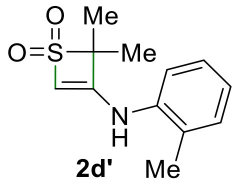

2d'

2,2-dimethyl-3-(o-tolylamino)-2*H*-thiete 1,1-dioxide

2d': Yellow solid (45 mg, 94% yield); mp: 135 – 137 °C; **TLC** (PE: EA, 2:1, v/v): R_f = 0.20; **^{1}H NMR (500 MHz, $CDCl_3$):** δ 7.24 – 7.17 (m, 2H), 7.15 – 7.08 (m, 2H), 6.70 (s, 1H), 5.16 (s, 1H), 2.26 (s, 3H), 1.73 (s, 6H); **^{13}C NMR (125 MHz, $CDCl_3$):** δ 154.4, 136.8, 131.4, 131.1, 127.2, 126.4, 122.6, 102.4, 80.4, 20.2, 17.5; **IR (KBr):** *v* 3314, 3094, 3046, 2986, 2973, 2931, 1620, 1589, 1534, 1489, 1459, 1385, 1256, 1202, 1179, 1117, 1096, 1084, 867, 825, 808, 757, 733, 607, 479, 461 cm^{-1}; **HRMS (EI-TOF)** *m/z*: [M]$^+$ calcd. for $C_{12}H_{15}NO_2S$, 237.0823; found, 237.0825.

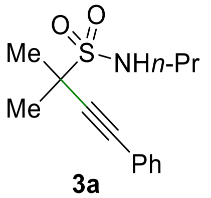

3a

2-methyl-4-phenyl-*N*-propylbut-3-yne-2-sulfonamide

3a: White solid (43 mg, 82% yield); mp: 79 – 81 °C; **TLC** (PE: EA, 10:1, v/v): R_f = 0.14; **^{1}H NMR (400 MHz, $CDCl_3$):** δ 7.45 – 7.40 (m, 2H), 7.34 – 7.28 (m, 3H), 4.71 (t, *J* = 6.0 Hz, 1H), 3.31 ((q, *J* = 6.8 Hz, 2H), 1.73 (s, 6H), 1.67 – 1.55 (m, 2H), 0.93 (t, *J* = 7.2 Hz, 3H); **^{13}C NMR (100 MHz, $CDCl_3$):** δ 131.8, 128.8, 128.4, 122.2, 87.9, 85.1, 58.8, 47.4, 24.9, 24.3, 11.1; **IR (KBr):** *v* 3303, 2980, 2963, 2934, 2862, 2222, 1441, 1316, 1163, 1118, 1077, 918, 856, 763, 696, 653, 566, 473 cm^{-1}; **HRMS (EI-TOF)** *m/z*: [M]$^+$ calcd. for $C_{14}H_{19}NO_2S$, 265.1136; found, 265.1133.

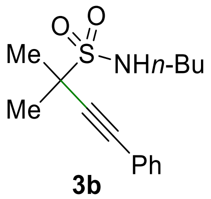

3b

***N*-butyl-2-methyl-4-phenylbut-3-yne-2-sulfonamide**

3b: White solid (50 mg, 89% yield); mp: 58 – 60 °C; **TLC** (PE: EA, 10:1, v/v): R_f = 0.18; **^{1}H NMR**

(400 MHz, CDCl$_3$): δ 7.45 – 7.40 (m, 2H), 7.36 – 7.27 (m, 3H), 4.66 (t, J = 6.0 Hz, 1H), 3.34 (q, J = 6.8 Hz, 2H), 1.73 (s, 6H), 1.61 – 1.51 (m, 2H), 1.41 – 1.29 (m, 2H), 0.89 (t, J = 7.2 Hz, 3H); **^{13}C NMR (100 MHz, CDCl$_3$):** δ 131.8, 128.8, 128.4, 122.2, 87.9, 85.1, 58.8, 45.5, 33.1, 24.9, 19.8, 13.7; **IR (KBr):** v 3293, 2981, 2962, 2934, 2869, 2221, 1437, 1316, 1169, 1119, 1071, 925, 877, 763, 695, 656, 565, 508, 473 cm^{-1}; **HRMS (EI-TOF)** *m/z*: [M]$^+$ calcd. for $C_{15}H_{21}NO_2S$, 279.1293; found, 279.1299.

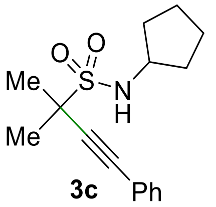

3c

***N*-cyclopentyl-2-methyl-4-phenylbut-3-yne-2-sulfonamide**

3c: White solid (42 mg, 72% yield); mp: 98 – 100 °C; **TLC** (PE: EA, 10:1, v/v): R_f = 0.14; **^{1}H NMR (400 MHz, CDCl$_3$):** δ 7.45 – 7.40 (m, 2H), 7.36 – 7.28 (m, 3H), 4.61 (d, J = 8.8 Hz, 1H), 4.10 – 3.96 (m, 1H), 2.08 – 1.95 (m, 2H), 1.73 (s, 6H), 1.71 – 1.64 (m, 2H), 1.60 – 1.48 (m, 4H); **^{13}C NMR (100 MHz, CDCl$_3$):** δ 131.8, 128.8, 128.4, 122.3, 88.0, 85.0, 58.7, 57.6, 34.6, 25.0, 23.3; **IR (KBr):** v 3264, 2966, 2937, 2902, 2867, 2221, 1452, 1307, 1166, 1124, 1090, 915, 756, 697, 654, 563, 542, 514 cm^{-1}; **HRMS (EI-TOF)** *m/z*: [M]$^+$ calcd. for $C_{16}H_{21}NO_2S$, 291.1293; found, 291.1289.

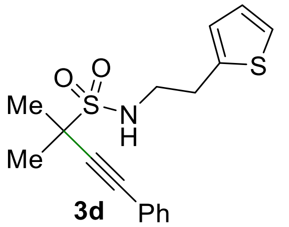

3d

2-methyl-4-phenyl-*N*-(2-(thiophen-2-yl)ethyl)but-3-yne-2-sulfonamide

3d: Light yellow solid (31 mg, 46% yield); mp: 91 – 93 °C; **TLC** (PE: EA, 10:1, v/v): R_f = 0.10; **^{1}H NMR (400 MHz, CDCl$_3$):** δ 7.44 – 7.38 (m, 2H), 7.36 – 7.27 (m, 3H), 7.16 (dd, J_1 = 5.2 Hz, J_2 = 1.2 Hz, 1H), 6.92 (dd, J_1 = 5.2 Hz, J_2 = 3.2 Hz, 1H), 6.87 – 6.84 (m, 1H), 4.44 (t, J = 6.4 Hz, 1H), 3.63 (q, J = 6.4 Hz, 2H), 3.12 (t, J = 6.4 Hz, 2H), 1.73 (s, 6H); **^{13}C NMR (100 MHz, CDCl$_3$):** δ 140.2, 131.9, 128.9, 128.5, 127.3, 126.1, 124.5, 122.1, 87.7, 85.4, 59.0, 46.8, 31.7, 24.9; **IR (KBr):** v 3284, 2989, 2931, 2869, 2222, 1442, 1314, 1166, 1119, 1083, 893, 848, 761, 713, 695, 653, 562, 497, 468 cm^{-1}; **HRMS (EI-TOF)** *m/z*: [M]$^+$ calcd. for $C_{17}H_{19}NO_2S_2$, 333.0857; found, 333.0855.

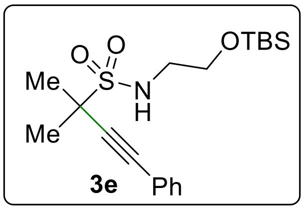

***N*-(2-((tert-butyldimethylsilyl)oxy)ethyl)-2-methyl-4-phenylbut-3-yne-2-sulfonamide**

3e: Light yellow gum (33 mg, 44% yield); **TLC** (PE: EA, 10:1, v/v): R_f= 0.20; **^{1}H NMR (400 MHz, CDCl$_3$):** δ 7.45 – 7.39 (m, 2H), 7.36 – 7.28 (m, 3H), 4.71 (t, *J* = 5.6 Hz, 1H), 3.75 (t, *J* = 5.2 Hz, 2H), 3.45 (q, *J* = 5.6 Hz, 2H), 1.75 (s, 6H), 0.88 (s, 9H), 0.06 (s, 6H); **^{13}C NMR (100 MHz, CDCl$_3$):** δ 131.9, 128.9, 128.5, 122.2, 87.8, 85.3, 62.9, 58.9, 47.6, 26.0, 24.9, 18.4, -5.3; **IR (KBr):** *v* 3309, 2955, 2927, 2858, 2223, 1488, 1323, 1255, 1171, 1125, 1088, 969, 841, 778, 757, 692, 566 cm^{-1}; **HRMS (EI-TOF)** *m/z*: [M]$^+$ calcd. for $C_{19}H_{31}NO_3SSi$, 381.1794; found, 381.1795.

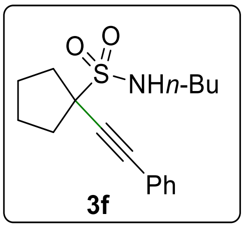

***N*-butyl-1-(phenylethynyl)cyclopentane-1-sulfonamide**

3f: Light yellow solid (50 mg, 82% yield); mp: 44 – 46 °C; **TLC** (PE: EA, 10:1, v/v): R_f= 0.18; **^{1}H NMR (500 MHz, CDCl$_3$):** δ 7.45 – 7.40 (m, 2H), 7.35 – 7.29 (m, 3H), 4.59 (t, *J* = 6.0 Hz, 1H), 3.37 – 3.31 (m, 2H), 2.50 – 2.41 (m, 2H), 2.28 – 2.20 (m, 2H), 1.93 – 1.85 (m, 4H), 1.60 – 1.52 (m, 2H), 1.40 – 1.31 (m, 2H), 0.90 (t, *J* = 7.5 Hz, 3H); **^{13}C NMR (125 MHz, CDCl$_3$):** δ 131.8, 128.8, 128.4, 122.4, 88.7, 85.2, 68.0, 45.1, 37.8, 33.0, 25.4, 19.8, 13.7; **IR (KBr):** *v* 3270, 2960, 2933, 2871, 2226, 1445, 1302, 1146, 1088, 969, 918, 876, 757, 693, 609, 537, 475 cm^{-1}; **HRMS (EI-TOF)** *m/z*: [M]$^+$ calcd. for $C_{17}H_{23}NO_2S$, 305.1449; found, 305.1445.

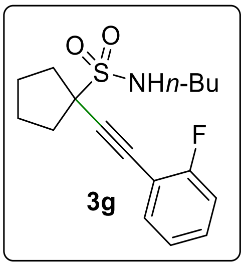

***N*-butyl-1-((2-fluorophenyl)ethynyl)cyclopentane-1-sulfonamide**

3g: Yellow oil (55 mg, 85% yield); **TLC** (PE: EA, 10:1, v/v): R_f= 0.20; **^{1}H NMR (500 MHz, CDCl$_3$):**

δ 7.45 – 7.39 (m, 1H), 7.35 – 7.29 (m, 1H), 7.13 – 7.04 (m, 2H), 4.70 (t, *J* = 6.0 Hz, 1H), 3.40 – 3.31 (m, 2H), 2.51 – 2.40 (m, 2H), 2.31 – 2.19 (m, 2H), 1.96 – 1.83 (m, 4H), 1.61 – 1.51 (m, 2H), 1.42 – 1.31 (m, 2H), 0.91 (t, *J* = 7.5 Hz, 3H); **^{13}C NMR (125 MHz, CDCl$_3$):** δ 163.0 (d, *J* = 251.4 Hz), 133.5 (d, *J* = 0.9 Hz), 130.5 (d, *J* = 8.0 Hz), 124.1 (d, *J* = 3.7 Hz), 115.6 (d, *J* = 20.9 Hz), 111.0 (d, *J* = 15.5 Hz), 94.0 (d, *J* = 3.3 Hz), 78.8, 68.0, 45.0, 37.8, 32.9, 25.4, 19.7, 13.7; **^{19}F NMR (376 MHz, CDCl$_3$):** δ -110.18; **IR (KBr):** *v* 3302, 2964, 2938, 2873, 2234, 1494, 1449, 1320, 1262, 1222, 1144, 1082, 1031, 755, 606, 535 cm^{-1}; **HRMS (EI-TOF)** *m/z*: [M]$^+$ calcd. for $C_{17}H_{22}FNO_2S$, 323.1355; found, 323.1359.

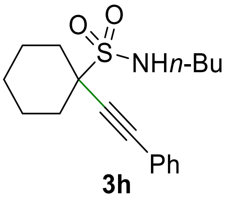

3h

***N*-butyl-1-(phenylethynyl)cyclohexane-1-sulfonamide**

3h: White solid (54 mg, 84% yield); mp: 79 – 81 °C; **TLC** (PE: EA, 10:1, v/v): R_f = 0.19; **^{1}H NMR (400 MHz, CDCl$_3$):** δ 7.49 – 7.42 (m, 2H), 7.37 – 7.27 (m, 3H), 4.46 (t, *J* = 5.6 Hz, 1H), 3.34 (q, *J* = 6.8 Hz, 2H), 2.27 – 2.16 (m, 2H), 1.96 – 1.86 (m, 2H), 1.84 – 1.67 (m, 5H), 1.60 – 1.50 (m, 2H), 1.40 – 1.29 (m, 2H), 1.27 – 1.15 (m, 1H), 0.89 (t, *J* = 7.2 Hz, 3H); **^{13}C NMR (100 MHz, CDCl$_3$):** δ 131.9, 128.8, 128.4, 122.4, 87.8, 86.0, 64.7, 45.4, 33.2, 32.3, 25.1, 22.7, 19.8, 13.7; **IR (KBr):** *v* 3294, 2954, 2937, 2858, 2231, 1493, 1443, 1321, 1141, 1075, 916, 883, 758, 693, 653, 601, 537, 505 cm^{-1}; **HRMS (EI-TOF)** *m/z*: [M]$^+$ calcd. for $C_{18}H_{25}NO_2S$, 319.1606; found, 319.1600.

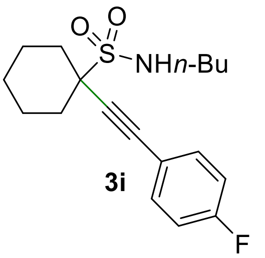

3i

***N*-butyl-1-((4-fluorophenyl)ethynyl)cyclohexane-1-sulfonamide**

3i: White solid (57 mg, 84% yield); mp: 95 – 97 °C; **TLC** (PE: EA, 10:1, v/v): R_f = 0.13; **^{1}H NMR (500 MHz, CDCl$_3$):** δ 7.51 – 7.40 (m, 2H), 7.07 – 6.99 (m, 2H), 4.65 (br, 1H), 3.32 (q, *J* = 6.5 Hz, 2H), 2.24 – 2.17 (m, 2H), 1.93 – 1.85 (m, 2H), 1.84 – 1.78 (m, 2H), 1.76 – 1.64 (m, 3H), 1.60 – 1.51 (m, 2H), 1.39 – 1.30 (m, 2H), 1.26 – 1.15 (m, 1H), 0.89 (t, *J* = 7.5 Hz, 3H); **^{13}C NMR (125 MHz,

CDCl$_3$): δ 162.8 (d, *J* = 250.1 Hz), 133.8 (d, *J* = 8.4 Hz), 118.5 (d, *J* = 3.5 Hz), 115.7 (d, *J* = 22.1 Hz), 86.7, 85.7 (d, *J* = 1.1 Hz), 64.6, 45.3, 33.2, 32.2, 25.0, 22.6, 19.8, 13.7; **^{19}F NMR (376 MHz, CDCl$_3$)**: δ -110.15; **IR (KBr):** *v* 3290, 2956, 2935, 2861, 2223, 1601, 1509, 1445, 1437, 1320, 1236, 1222, 1160, 1143, 1074, 1014, 883, 839, 735, 596, 534, 461 cm^{-1}; **HRMS (EI-TOF)** *m/z*: [M]$^+$ calcd. for $C_{18}H_{24}FNO_2S$, 337.1512; found, 337.1510.

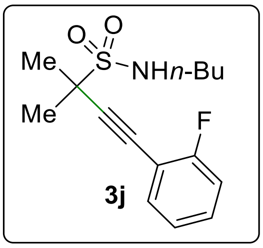

***N*-butyl-4-(2-fluorophenyl)-2-methylbut-3-yne-2-sulfonamide**

3j: Colourless oil (43 mg, 72% yield). **TLC** (PE: EA, 10:1, v/v): R_f = 0.18; **^{1}H NMR (500 MHz, CDCl$_3$):** δ 7.45 – 7.40 (m, 1H), 7.35 – 7.29 (m, 1H), 7.13 – 7.05 (m, 2H), 4.43 (t, *J* = 6.0 Hz, 1H), 3.42 – 3.28 (m, 2H), 1.75 (s, 6H), 1.61 – 1.54 (m, 2H), 1.40 – 1.33 (m, 2H), 0.91 (t, *J* = 7.5 Hz, 3H); **^{13}C NMR (125 MHz, CDCl$_3$):** δ 163.1 (d, *J* = 251.7 Hz), 133.7 (d, *J* = 0.9 Hz), 130.6 (d, *J* = 8.0 Hz), 124.1 (d, *J* = 3.7 Hz), 115.6 (d, *J* = 20.8 Hz), 110.9 (d, *J* = 15.5 Hz), 93.2 (d, *J* = 3.3 Hz), 78.8, 58.9, 45.5, 33.2, 24.9, 19.8, 13.7; **^{19}F NMR (376 MHz, CDCl$_3$):** δ -110.03; **IR (KBr):** *v* 3303, 2961, 2936, 2872, 2233, 1517, 1493, 1453, 1320, 1263, 1224, 1169, 1127, 1083, 1033, 863, 806, 758, 649, 567, 554, 475 cm^{-1}; **HRMS (EI-TOF)** *m/z*: [M]$^+$ calcd. for $C_{15}H_{20}FNO_2S$, 297.1199; found, 297.1195.

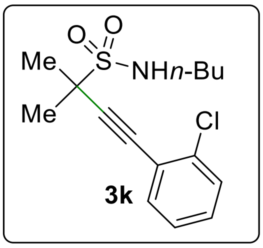

***N*-butyl-4-(2-chlorophenyl)-2-methylbut-3-yne-2-sulfonamide**

3k: Colourless oil (54 mg, 86% yield); **TLC** (PE: EA, 10:1, v/v): R_f = 0.18; **^{1}H NMR (400 MHz, CDCl$_3$):** δ 7.50 – 7.44 (m, 1H), 7.43 – 7.37 (m, 1H), 7.31 – 7.19 (m, 2H), 4.53 (t, *J* = 5.6 Hz, 1H), 3.41 – 3.29 (m, 2H), 1.76 (s, 6H), 1.61 – 1.52 (m, 2H), 1.41 – 1.30 (m, 2H), 0.90 (t, *J* = 7.2 Hz, 3H); **^{13}C NMR (100 MHz, CDCl$_3$):** δ 136.3, 133.5, 129.9, 129.4, 126.7, 122.1, 93.2, 82.1, 59.0, 45.4, 33.2, 24.8, 19.8, 13.7; **IR (KBr):** *v* 3305, 2959, 2934, 2871, 2229, 1711, 1474, 1432, 1319, 1170, 1125,

1082, 1059, 1033, 896, 864, 757, 680, 647, 572, 540, 498 cm^{-1}; **HRMS (EI-TOF)** *m/z*: $[M]^+$ calcd. for $C_{15}H_{20}ClNO_2S$, 313.0903; found, 313.0905.

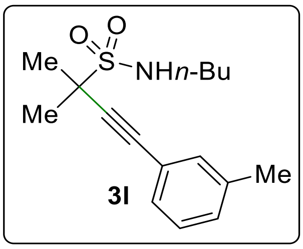

***N*-butyl-2-methyl-4-(m-tolyl)but-3-yne-2-sulfonamide**

3l: Colourless oil (39 mg, 66% yield); **TLC** (PE: EA, 10:1, v/v): R_f = 0.25; **^{1}H NMR (400 MHz, $CDCl_3$):** δ 7.30 – 7.09 (m, 4H), 4.56 (t, *J* = 6.0 Hz, 1H), 3.34 (q, *J* = 6.4 Hz, 2H), 2.32 (s, 3H), 1.73 (s, 6H), 1.62 – 1.52 (m, 2H), 1.40 – 1.31 (m, 2H), 0.89 (t, *J* = 7.2 Hz, 3H); **^{13}C NMR (100 MHz, $CDCl_3$):** δ 138.1, 132.5, 129.7, 128.9, 128.3, 122.0, 87.5, 85.3, 58.8, 45.5, 33.1, 25.0, 21.3, 19.8, 13.7; **IR (KBr):** *v* 3307, 2960, 2935, 2873, 2225, 1604, 1462, 1313, 1170, 1128, 1082, 903, 786, 692, 652, 574, 553 cm^{-1}; **HRMS (EI-TOF)** *m/z*: $[M]^+$ calcd. for $C_{16}H_{23}NO_2S$, 293.1449; found, 293.1445.

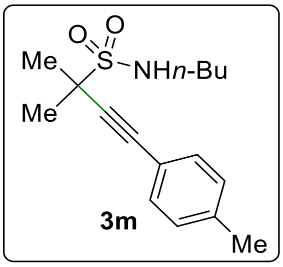

***N*-butyl-2-methyl-4-(*p*-tolyl)but-3-yne-2-sulfonamide**

3m: Colourless gum (38 mg, 64% yield); **TLC** (PE: EA, 10:1, v/v): R_f = 0.16; **^{1}H NMR (400 MHz, $CDCl_3$):** δ 7.32 (d, *J* = 8.0 Hz, 2H), 7.12 (d, *J* = 8.0 Hz, 2H), 4.43 (t, *J* = 5.6 Hz, 1H), 3.34 (q, *J* = 6.8 Hz, 2H), 2.35 (s, 3H), 1.73 (s, 6H), 1.60 – 1.52 (m, 2H), 1.40 – 1.30 (m, 2H), 0.89 (t, *J* = 7.2 Hz, 3H); **^{13}C NMR (100 MHz, $CDCl_3$):** δ 139.0, 131.8, 129.2, 119.2, 87.2, 85.3, 58.9, 45.5, 33.2, 25.0, 21.6, 19.8, 13.7; **IR (KBr):** *v* 3292, 2960, 2935, 2870, 2224, 1510, 1461, 1432, 1316, 1167, 1119, 1087, 1038, 953, 896, 818, 687, 630, 566, 539, 459 cm^{-1}; **HRMS (EI-TOF)** *m/z*: $[M]^+$ calcd. for $C_{16}H_{23}NO_2S$, 293.1449; found, 293.1444.

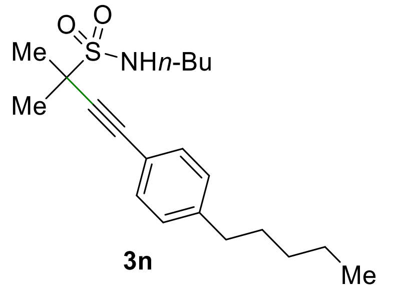

3n

***N*-butyl-2-methyl-4-(4-pentylphenyl)but-3-yne-2-sulfonamide**

3n: Light yellow oil (56 mg, 81% yield); **TLC** (PE: EA, 10:1, v/v): R_f = 0.25; **^{1}H NMR (600 MHz, $CDCl_3$):** δ 7.33 (d, *J* = 7.8 Hz, 2H), 7.12 (d, *J* = 7.8 Hz, 2H), 4.39 (t, *J* = 6.0 Hz, 1H), 3.35 (q, *J* = 6.6 Hz, 2H), 2.59 (t, *J* = 6.6 Hz, 2H), 1.72 (s, *J* = 7.2 Hz, 6H), 1.63 – 1.53 (m, 4H), 1.39 – 1.26 (m, 6H), 0.93 – 0.86 (m, 6H); **^{13}C NMR (150 MHz, $CDCl_3$):** δ 144.1, 131.8, 128.6, 119.4, 87.3, 85.4, 58.9, 45.5, 36.0, 33.2, 31.5, 31.0, 25.0, 22.6, 19.8, 14.1, 13.7; **IR (KBr):** *v* 3297, 2960, 2929, 2870, 2195, 1603, 1511, 1463, 1380, 1315, 1166, 1116, 1088, 1038, 982, 892, 844, 799, 688, 634, 589, 554 cm^{-1}; **HRMS (EI-TOF)** *m/z*: $[M]^+$ calcd. for $C_{20}H_{31}NO_2S$, 349.2075; found, 349.2074.

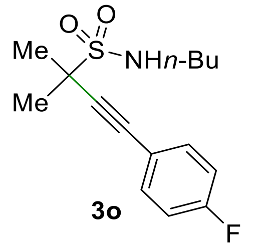

3o

***N*-butyl-4-(4-fluorophenyl)-2-methylbut-3-yne-2-sulfonamide**

3o: Colourless gum (42 mg, 70% yield); **TLC** (PE: EA, 10:1, v/v): R_f = 0.16; **^{1}H NMR (400 MHz, $CDCl_3$):** δ 7.45 – 7.39 (m, 2H), 7.06 – 6.97 (m, 2H), 4.58 (br, 1H), 3.37 – 3.29 (m, 2H), 1.73 (s, 6H), 1.60 – 1.52 (m, 2H), 1.41 – 1.31 (m, 2H), 0.89 (t, *J* = 7.2 Hz, 3H); **^{13}C NMR (100 MHz, $CDCl_3$):** δ 162.8 (d, *J* = 250.1 Hz), 133.8 (d, *J* = 8.4 Hz), 118.3 (d, *J* = 3.4 Hz), 115.8 (d, *J* = 22.1 Hz), 87.6, 84.1, 58.8, 45.5, 33.1, 24.9, 19.8, 13.7; **^{19}F NMR (376 MHz, $CDCl_3$):** δ -110.05; **IR (KBr):** *v* 3288, 2957, 2937, 2872, 2222, 1602, 1508, 1465, 1442, 1318, 1225, 1164, 1125, 1083, 839, 799, 687, 625, 566 cm^{-1}; **HRMS (EI-TOF)** *m/z*: $[M]^+$ calcd. for $C_{15}H_{20}FNO_2S$, 297.1199; found, 297.1198.

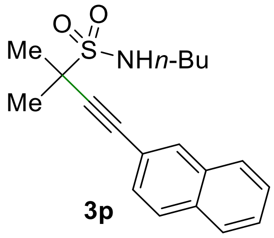

3p

***N*-butyl-2-methyl-4-(naphthalen-2-yl)but-3-yne-2-sulfonamide**

3p: White solid (57 mg, 86% yield); mp: 96 – 98 °C; **TLC** (PE: EA, 10:1, v/v): R_f = 0.16; **^{1}H NMR (400 MHz, $CDCl_3$):** δ 7.97 (s, 1H), 7.85 – 7.68 (m, 3H), 7.52 – 7.41 (m, 3H), 4.67 (t, *J* = 5.6 Hz, 1H), 3.37 (q, *J* = 6.4 Hz, 2H), 1.77 (s, 6H), 1.63 – 1.50 (m, 2H), 1.40 – 1.28 (m, 2H), 0.87 (t, *J* = 7.2 Hz, 3H); **^{13}C NMR (100 MHz, $CDCl_3$):** δ 133.0, 132.9, 132.0, 128.3, 128.1, 127.8 (two peaks: 127.84 and 127.82), 127.0, 126.8, 119.5, 88.2, 85.5, 58.9, 45.5, 33.1, 25.0, 19.8, 13.7; **IR (KBr):** *ν* 3290, 2957, 2929, 2862, 2222, 1595, 1435, 1317, 1170, 1116, 1078, 864, 835, 744, 685, 640, 570, 562, 531 474 cm^{-1}; **HRMS (EI-TOF)** *m/z*: $[M]^+$ calcd. for $C_{19}H_{23}NO_2S$, 329.1449; found, 329.1447.

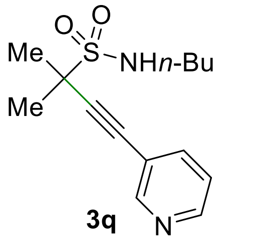

3q

***N*-butyl-2-methyl-4-(pyridin-3-yl)but-3-yne-2-sulfonamide**

3q: White solid (37 mg, 66% yield); mp: 94 – 96 °C; **TLC** (PE: EA, 1:1, v/v): R_f = 0.33; **^{1}H NMR (500 MHz, $CDCl_3$):** δ 8.64 (dd, J_1 = 2.0 Hz, J_2 = 1.0 Hz, 1H), 8.55 (dd, J_1 = 5.0 Hz, J_2 = 2.0 Hz, 1H), 7.75 – 7.67 (m, 1H), 7.27 – 7.23 (m, 1H), 4.91 (t, *J* = 6.0 Hz, 1H), 3.40 – 3.29 (m, 2H), 1.75 (s, 6H), 1.63 – 1.54 (m, 2H), 1.42 – 1.32 (m, 2H), 0.90 (t, *J* = 7.5 Hz, 3H); **^{13}C NMR (125 MHz, $CDCl_3$):** δ 152.4, 149.1, 138.9, 123.2, 119.5, 91.6, 81.8, 58.8, 45.5, 33.2, 24.8, 19.8, 13.7; **IR (KBr):** *ν* 3296, 2961, 2929, 2867, 2223, 1590, 1452, 1312, 1162, 1130, 1083, 1042, 870, 842, 757, 705, 654, 570, 464 cm^{-1}; **HRMS (EI-TOF)** *m/z*: $[M]^+$ calcd. for $C_{14}H_{20}N_2O_2S$, 280.1245; found, 280.1247.

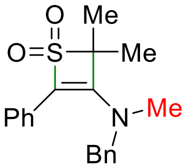

4a

3-(benzyl(methyl)amino)-2,2-dimethyl-4-phenyl-2*H*-thiete 1,1-dioxide

4a: White solid (46 mg, 71% yield); mp: 149 – 151 °C; **TLC**: (PE: EA, 3:1, v/v): R_f = 0.20; **^{1}H NMR (500 MHz, $CDCl_3$):** δ 7.40 – 7.23 (m, 8H), 7.15 (d, *J* = 7.5 Hz, 2H), 4.35 (s, 2H), 2.77 (s, 3H), 1.82 (s, 6H); **^{13}C NMR (125 MHz, $CDCl_3$):** δ 152.8, 135.5, 129.9, 129.1, 128.4, 128.2 (two peaks: 128.17 and 128.15), 128.0, 127.0, 115.6, 78.1, 56.5, 38.7, 21.6; **IR (KBr):** *ν* 3064, 3031, 2967, 2926, 1623, 1500, 1456, 1445, 1418, 1262, 1153, 1113, 1084, 875, 771, 708, 697, 608, 561, 497, 456 cm^{-1}; **HRMS (EI-TOF)** *m/z*: $[M]^+$ calcd. for $C_{19}H_{21}NO_2S$, 327.1293; found, 327.1297.

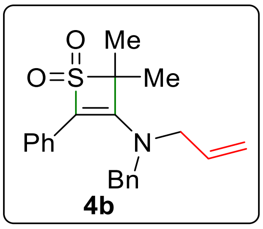

3-(allyl(benzyl)amino)-2,2-dimethyl-4-phenyl-2*H*-thiete 1,1-dioxide

4b: White solid (41 mg, 58% yield); mp: 122 – 124 °C; **TLC**: (PE: EA, 5:1, v/v): R_f = 0.14; **^{1}H NMR (600 MHz, $CDCl_3$):** δ 7.42 – 7.23 (m, 8H), 7.09 (d, *J* = 7.2 Hz, 2H), 5.74 – 5.58 (m, 1H), 5.26 (d, *J* = 10.2 Hz, 1H), 5.09 (d, *J* = 16.8 Hz, 1H), 4.32 (s, 2H), 3.66 (d, *J* = 5.4 Hz, 2H), 1.83 (s, 6H); **^{13}C NMR (125 MHz, $CDCl_3$):** δ 152.6, 135.7, 131.7, 130.1, 129.1, 128.5, 128.4, 128.1, 127.9, 127.1, 119.0, 116.0, 78.3, 53.0, 51.8, 21.7; **IR (KBr):** *ν* 3033, 2985, 2924, 2867, 1642, 1626, 1594, 1496, 1433, 1257, 1239, 1154, 1114, 1100, 862, 764, 741, 702, 647, 602, 562, 511, 478 cm^{-1}; **HRMS (EI-TOF)** *m/z*: $[M]^+$ calcd. for $C_{21}H_{23}NO_2S$, 353.1449; found, 353.1445.

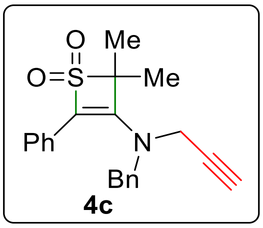

3-(benzyl(prop-2-yn-1-yl)amino)-2,2-dimethyl-4-phenyl-2*H*-thiete 1,1-dioxide

4c: Yellow oil (39 mg, 55% yield); **TLC**: (PE: EA, 5:1, v/v): R_f = 0.12; **^{1}H NMR (500 MHz, $CDCl_3$):** δ 7.46 – 7.41 (m, 2H), 7.38 – 7.27 (m, 6H), 7.20 – 7.16 (m, 2H), 4.41 (s, 2H), 3.72 (d, *J* = 2.5 Hz, 2H), 2.38 (t, *J* = 2.5 Hz, 1H), 1.86 (s, 6H); **^{13}C NMR (125 MHz, $CDCl_3$):** δ 152.1, 134.8, 129.8, 129.2, 128.7, 128.6, 128.4, 127.7, 127.4, 118.4, 78.6, 77.2, 74.5, 53.3, 39.1, 21.6; **IR (KBr):** *ν* 3262, 3056, 3030, 2974, 2932, 2116, 1624, 1496, 1455, 1427, 1362, 1265, 1157, 1107, 1027, 966, 867, 755, 698,

607, 535, 495, 454 cm^{-1}; **HRMS (EI-TOF)** *m/z*: $[M]^+$ calcd. for $C_{21}H_{21}NO_2S$, 351.1293; found, 351.1295.

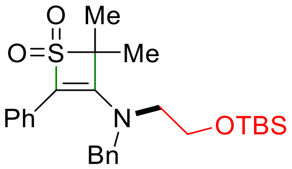

4d

3-(benzyl(2-((tert-butyldimethylsilyl)oxy)ethyl)amino)-2,2-dimethyl-4-phenyl-2*H*-thiete 1,1-dioxide

4d: Light yellow solid (56 mg, 60% yield); mp: 80 – 81 °C; **TLC**: (PE: EA, 5:1, v/v): $R_f = 0.20$; **^{1}H NMR (500 MHz, $CDCl_3$):** δ 7.38 – 7.24 (m, 8H), 7.12 (d, *J* = 7.0 Hz, 2H), 4.46 (s, 2H), 3.56 (t, *J* = 5.0 Hz, 2H), 3.20 (t, *J* = 5.0 Hz, 2H), 1.82 (s, 6H), 0.89 (s, 9H), 0.03 (s, 6H); **^{13}C NMR (125 MHz, $CDCl_3$):** δ 152.7, 136.2, 130.1, 129.1, 128.5, 128.3, 128.1 (two peaks: 128.08 and 128.05), 127.1, 115.8, 78.5, 60.5, 54.8, 51.3, 26.0, 21.9, 18.3, -5.4; **IR (KBr):** *ν* 2953, 2926, 2881, 2856, 1625, 1499, 1436, 1386, 1361, 1262, 1155, 1107, 1085, 918, 832, 781, 740, 700, 600, 513, 483 cm^{-1}; **HRMS (EI-TOF)** *m/z*: $[M]^+$ calcd. for $C_{26}H_{37}NO_3SSi$, 471.2263; found, 471.2260.

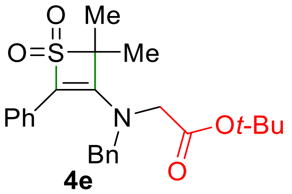

4e

tert-butyl *N*-benzyl-*N*-(2,2-dimethyl-1,1-dioxido-4-phenyl-2*H*-thiet-3-yl)glycinate

4e: White solid (61 mg, 72% yield); mp: 126 – 127 °C; **TLC**: (PE: EA, 5:1, v/v): $R_f = 0.22$; **^{1}H NMR (400 MHz, $CDCl_3$):** δ 7.46 – 7.27 (m, 8H), 7.12 (d, *J* = 7.2 Hz, 2H), 4.38 (s, 2H), 3.59 (s, 2H), 1.83 (s, 6H), 1.41 (s, 9H); **^{13}C NMR (100 MHz, $CDCl_3$):** δ 167.5, 152.8, 135.0, 130.2, 129.2, 128.6 (two peaks: 128.61 and 128.57), 128.4, 127.8, 127.5, 117.8, 82.9, 78.4, 55.5, 50.8, 28.1, 21.4; **IR (KBr):** *ν* 2979, 2930, 2883, 2854, 1739, 1626, 1372, 1272, 1227, 1155, 1107, 830, 740, 700, 607, 573, 492cm^{-1}; **HRMS (EI-TOF)** *m/z*: $[M]^+$ calcd. for $C_{24}H_{29}NO_4S$, 427.1817; found, 427.1820.

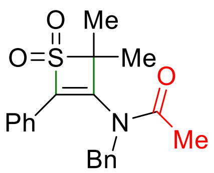

4f

***N*-benzyl-*N*-(2,2-dimethyl-1,1-dioxido-4-phenyl-2*H*-thiet-3-yl)acetamide**

4f: White solid (40 mg, 56% yield); mp: 119 – 120 °C; **TLC**: (PE: EA, 5:1, v/v): R_f = 0.18; **^{1}H NMR (400 MHz, $CDCl_3$):** δ 7.39 – 7.25 (m, 8H), 7.18 (d, *J* = 7.6 Hz, 2H), 4.83 (s, 2H), 2.03 (s, 3H), 1.75 (s, 6H); **^{13}C NMR (101 MHz, $CDCl_3$):** δ 169.7, 145.0, 136.0, 131.0, 129.5, 129.1, 128.4, 128.1, 127.2, 125.7, 83.9, 50.0, 22.1, 21.2; **IR (KBr):** *v* 3050, 3031, 2986, 2933, 1677, 1633, 1496, 1448, 1433, 1392, 1365, 1295, 1251, 1161, 1113, 976, 835, 767, 738, 698, 648, 592, 520, 432 cm^{-1}; **HRMS (EI-TOF)** *m/z*: $[M]^+$ calcd. for $C_{20}H_{21}NO_3S$, 355.1242; found, 355.1240.

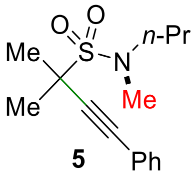

5

***N*,2-dimethyl-4-phenyl-*N*-propylbut-3-yne-2-sulfonamide**

5: Colourless oil (41mg, 73% yield); **TLC** (PE: EA, 5:1, v/v): R_f = 0.58; **^{1}H NMR (500 MHz, $CDCl_3$):** δ 7.44 – 7.40 (m, 2H), 7.36 – 7.29 (m, 3H), 3.37 (t, *J* = 7.5 Hz, 2H), 3.06 (s, 3H), 1.72 (s, 6H), 1.68 – 1.60 (m, 2H), 0.90 (t, *J* = 7.5 Hz, 3H); **^{13}C NMR (125 MHz, $CDCl_3$):** δ 131.8, 128.8, 128.5, 122.3, 88.4, 85.3, 59.7, 53.7, 36.3, 25.6, 21.4, 10.9; **IR (KBr):** *v* 2971, 2956, 2933, 2872, 2220, 1488, 1471, 1441, 1323, 1222, 1164, 1116, 983, 889, 848, 762, 694, 635, 571, 537, 519, 460 cm^{-1}; **HRMS (EI-TOF)** *m/z*: $[M]^+$ calcd. for $C_{15}H_{21}NO_2S$, 279.1293; found, 279.1292.

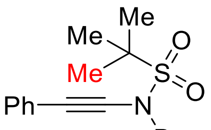

6

***N*-benzyl-2-methyl-*N*-(phenylethynyl)propane-2-sulfonamide**

6: White solid (60 mg, 91%); mp: 89 – 91 °C; **TLC** (PE: EA, 10:1, v/v): R_f = 0.50; **^{1}H NMR (500 MHz, $CDCl_3$):** δ 7.49 – 7.44 (m, 2H), 7.40 – 7.32 (m, 3H), 7.25 – 7.21 (m, 5H), 4.71 (s, 2H), 1.56 (s, 9H); **^{13}C NMR (125 MHz, $CDCl_3$):** δ 135.4, 131.0, 129.0, 128.7, 128.5, 128.3, 127.6, 123.1, 84.1,

70.5, 64.6, 57.3, 25.0; **IR (KBr):** *v* 3057, 2973, 2956, 2238, 1637, 1617, 1495, 1455, 1339, 1131, 1020, 928, 804, 783, 757, 696, 642, 594, 554, 474 cm^{-1}; **HRMS (EI-TOF)** *m/z*: $[M]^+$ calcd. for $C_{19}H_{21}NO_2S$, 327.1293; found, 327.1298.

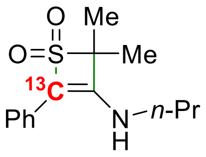

^{13}*C*-**2a**

2,2-dimethyl-4-phenyl-3-(propylamino)-2*H*-thiete 1,1-dioxide-4-^{13}C

^{13}C-**2a:** White solid (37 mg, 70% yield); mp: 139 – 141 °C; **TLC** (PE: EA, 1:1, v/v): R_f = 0.38; **^{1}H NMR (400 MHz, $CDCl_3$):** δ 7.39 – 7.23 (m, 5H), 4.96 – 4.80 (m, 1H), 3.06 (q, *J* = 6.5 Hz, 2H), 1.68 (s, 6H), 1.52 – 1.40 (m, 2H), 0.82 (t, *J* = 7.2 Hz, 3H); **^{13}C NMR (100 MHz, $CDCl_3$):** δ 150.9 (d, *J* = 74.1 Hz), 128.7 (d, *J* = 4.4 Hz), 128.7, 128.0, 127.7, 114.0 (^{13}C-labelled), 78.8 (d, *J* = 12.9 Hz), 47.0, 23.4, 20.6, 11.0; **IR (KBr):** *v* 3321, 3054, 2984, 2961, 2934, 2874, 1623, 1536, 1490, 1470, 1460, 1444, 1385, 1337, 1253, 1159, 1103, 831, 762, 701, 650, 608, 502 cm^{-1}; **HRMS (ESI-TOF)** *m/z*: $[M+Na]^+$ calcd. for $C_{13}{}^{13}CH_{19}NNaO_2S$, 289.1068; found, 289.1066.

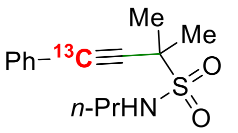

^{13}*C*-**3a**

2-methyl-4-phenyl-*N*-propylbut-3-yne-2-sulfonamide-4-^{13}C

^{13}C-**3a:** White solid (42 mg, 79% yield); mp: 81 – 83 °C; **TLC** (PE: EA, 10:1, v/v): R_f = 0.14; **^{1}H NMR (400 MHz, $CDCl_3$):** δ 7.46 – 7.40 (m, 2H), 7.37 – 7.27 (m, 3H), 4.55 – 4.36 (m, 1H), 3.32 (q, *J* = 6.8 Hz, 2H), 1.74 (s, 6H), 1.65 – 1.56 (m, 2H), 0.93 (t, *J* = 7.2 Hz, 3H); **^{13}C NMR (100 MHz, $CDCl_3$):** δ 131.9 (d, *J* = 1.4 Hz), 128.9 (d, *J* = 1.0 Hz), 128.5 (d, *J* = 5.6 Hz), 122.2 (d, *J* = 90.2 Hz), 86.5 (d, *J* = 148.2 Hz), 85.2 (*^{13}C*-labelled), 58.9 (d, *J* = 11.0 Hz), 47.5, 25.0, 24.4, 11.2; **IR (KBr):** *v* 3300, 2982, 2962, 2935, 2864, 2186, 1442, 1314, 1163, 1117, 1074, 1032, 1010, 917, 859, 763, 695, 651, 562, 515, 470 cm^{-1}; **HRMS (ESI-TOF)** *m/z*: $[M+Na]^+$ calcd. for $C_{13}{}^{13}CH_{19}NNaO_2S$, 289.1068; found, 289.1059.

12. X-ray Crystallographic Data

Crystals suitable for X-ray diffraction experiments were obtained by following methods: compound **2a**, **2j**, **2k**, **2q** and **2a'** were crystallized from their solution in PE/EA. Compound **3a** was crystallized from its solution in Hexane/Chloroform. Intensity data for compounds was collected on 'Bruke Apex2' diffractometer at 296(2) (MoK/α radiation, radiation wavelength = 0.7107). The structures were solved by direct methods and refined by the full-matrix least-squares method using the SHELX-97 program package.[7] The geometrical parameters and the figures were analyzed using the program OLEX2.[8]

2,2-dimethyl-4-phenyl-3-(propylamino)-2*H*-thiete 1,1-dioxide (2a)

CCDC Number = 1999861

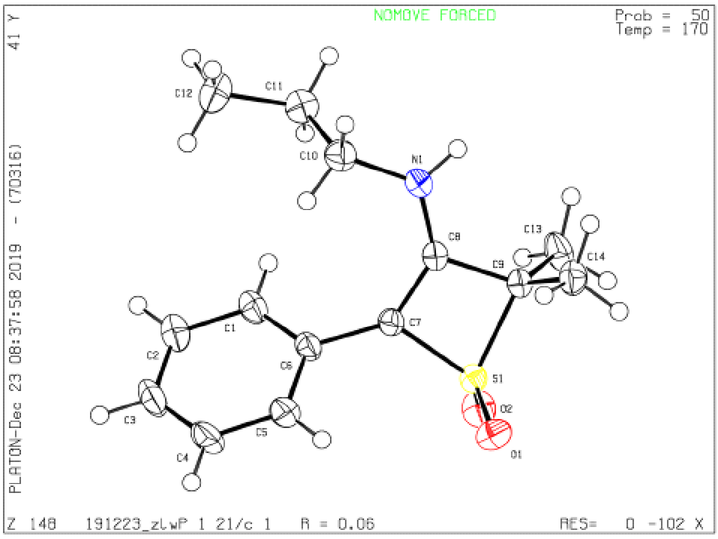

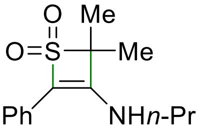

2a

Bond precision: C-C = 0.0030 A Wavelength=0.71073

Cell:	a=9.9779(13)	b=10.1376(11)	c=14.1180(18)
	alpha=90	beta=96.423(5)	gamma=90
Temperature:	170 K		

	Calculated	Reported
Volume	1419.1(3)	1419.1(3)
Space group	P 21/c	P 1 21/c 1
Hall group	-P 2ybc	-P 2ybc
Moiety formula	C14 H19 N O2 S	C14 H19 N O2 S
Sum formula	C14 H19 N O2 S	C14 H19 N O2 S
Mr	265.36	265.36
Dx,g cm-3	1.242	1.242
Z	4	4
Mu (mm-1)	0.223	0.223
F000	568.0	568.0
F000'	568.72	
h,k,lmax	12,13,18	12,13,18
Nref	3133	3123
Tmin,Tmax	0.950,0.982	0.523,0.746
Tmin'	0.919	

Correction method= # Reported T Limits: Tmin=0.523 Tmax=0.746
AbsCorr = MULTI-SCAN

Data completeness= 0.997 Theta(max)= 27.125

R(reflections)= 0.0612(2734) wR2(reflections)= 0.1521(3123)

S = 1.133 Npar= 166

Supplementary Fig. 8 X-ray analysis of 2a. Detail X-ray crystallographic data of **2a**.

2-methyl-4-phenyl-*N*-propylbut-3-yne-2-sulfonamide (3a)

CCDC Number = 1999862

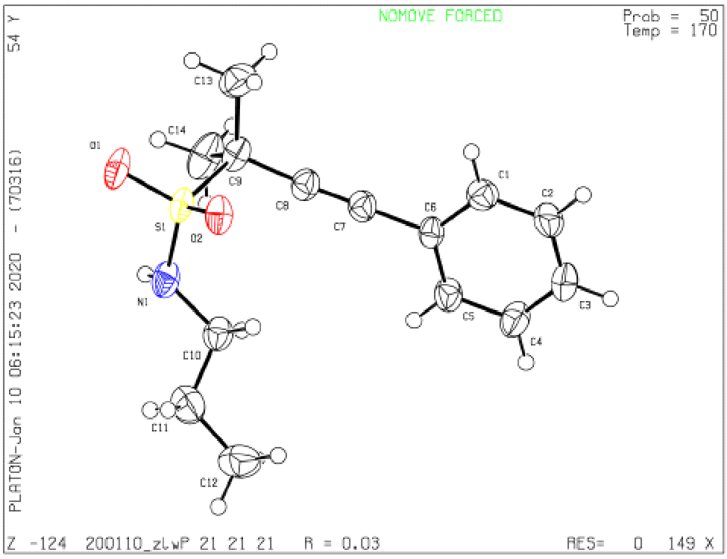

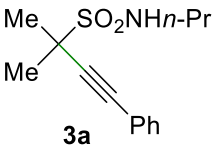

3a

Bond precision: C-C = 0.0037 A Wavelength=0.71073

Cell:	a=5.8496(7)	b=8.6981(11)	c=29.048(3)
	alpha=90	beta=90	gamma=90
Temperature:	170 K		

	Calculated	Reported
Volume	1478.0(3)	1478.0(3)
Space group	P 21 21 21	P 21 21 21
Hall group	P 2ac 2ab	P 2ac 2ab
Moiety formula	C14 H19 N O2 S	C14 H19 N O2 S
Sum formula	C14 H19 N O2 S	C14 H19 N O2 S
Mr	265.36	265.36
Dx,g cm-3	1.193	1.193
Z	4	4
Mu (mm-1)	0.214	0.214
F000	568.0	568.0
F000'	568.72	
h,k,lmax	7,11,37	7,11,37
Nref	3281[1934]	3236
Tmin,Tmax	0.967,0.979	0.585,0.746
Tmin'	0.902	

Correction method= # Reported T Limits: Tmin=0.585 Tmax=0.746
AbsCorr = MULTI-SCAN

Data completeness= 1.67/0.99 Theta(max)= 27.137

R(reflections)= 0.0344(2965) wR2(reflections)= 0.0851(3236)

S = 1.040 Npar= 166

Supplementary Fig. 9 X-ray analysis of 3a. Detail X-ray crystallographic data of **3a**.

2,2-dimethyl-4-phenyl-3-(phenylamino)-2*H*-thiete 1,1-dioxide (2j)

CCDC Number = 1999863

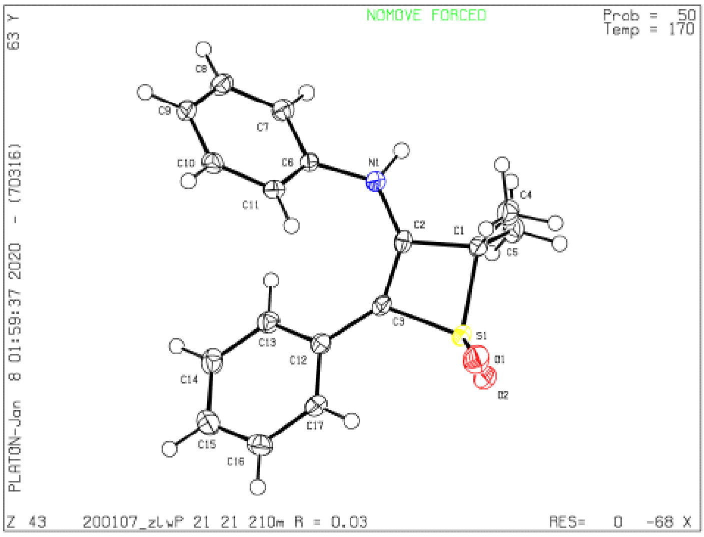

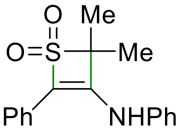

2j

Bond precision: C-C = 0.0026 A Wavelength=0.71073

Cell:	a=5.7268(2)	b=12.2097(3)	c=21.0218(7)
	alpha=90	beta=90	gamma=90
Temperature:	170 K		

	Calculated	Reported
Volume	1469.90(8)	1469.90(8)
Space group	P 21 21 21	P 21 21 21
Hall group	P 2ac 2ab	P 2ac 2ab
Moiety formula	C17 H17 N O2 S	C17 H17 N O2 S
Sum formula	C17 H17 N O2 S	C17 H17 N O2 S
Mr	299.38	299.37
Dx,g cm-3	1.353	1.353
Z	4	4
Mu (mm-1)	0.224	0.224
F000	632.0	632.0
F000'	632.75	
h,k,lmax	7,15,26	7,15,26
Nref	3251[1903]	3247
Tmin,Tmax	0.903,0.987	0.708,0.746
Tmin'	0.902	

Correction method= # Reported T Limits: Tmin=0.708 Tmax=0.746
AbsCorr = MULTI-SCAN

Data completeness= 1.71/1.00 Theta(max)= 27.103

R(reflections)= 0.0263(3157) wR2(reflections)= 0.0697(3247)

S = 1.074 Npar= 192

Supplementary Fig. 10 X-ray analysis of 2j. Detail X-ray crystallographic data of **2j**.

3-(methylamino)-4-phenyl-2*H*-thiete 1,1-dioxide (2k)

CCDC Number = 1999864

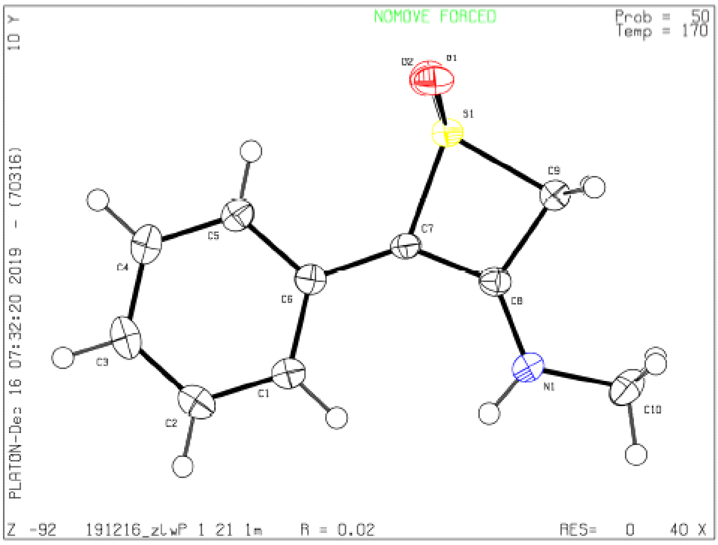

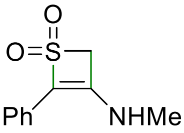

2k

Bond precision: C-C = 0.0030 A Wavelength=0.71073

Cell:	a=6.926(4)	b=5.416(4)	c=13.399(10)
	alpha=90	beta=96.47(3)	gamma=90
Temperature:	170 K		

	Calculated	Reported
Volume	499.4(6)	499.4(6)
Space group	P 21	P 1 21 1
Hall group	P 2yb	P 2yb
Moiety formula	C10 H11 N O2 S	C10 H11 N O2 S
Sum formula	C10 H11 N O2 S	C10 H11 N O2 S
Mr	209.26	209.26
Dx,g cm-3	1.392	1.392
Z	2	2
Mu (mm-1)	0.296	0.296
F000	220.0	220.0
F000'	220.34	
h,k,lmax	8,6,17	8,6,17
Nref	2202[1220]	2101
Tmin,Tmax	0.935,0.954	0.673,0.746
Tmin'	0.888	

Correction method= # Reported T Limits: Tmin=0.673 Tmax=0.746
AbsCorr = MULTI-SCAN

Data completeness= 1.72/0.95 Theta(max)= 27.108

R(reflections)= 0.0238(2070) wR2(reflections)= 0.0629(2101)

S = 1.100 Npar= 128

Supplementary Fig. 11 X-ray analysis of 2k. Detail X-ray crystallographic data of **2k**.

3-(benzylamino)-2-phenyl-1-thiaspiro[3.5]non-2-ene 1,1-dioxide (2q)

CCDC Number = 1999865

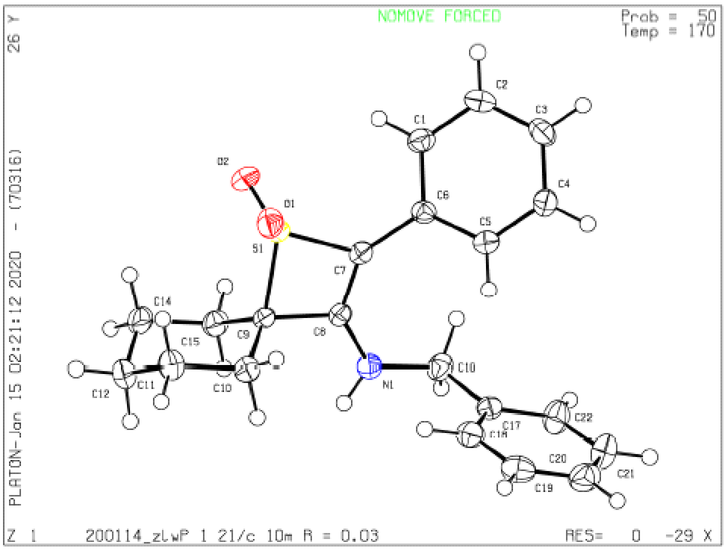

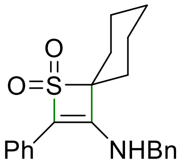

2q

Bond precision: C-C = 0.0019 A   Wavelength=0.71073

Cell:	a=9.749(2)	b=12.888(3)	c=14.638(5)
	alpha=90	beta=97.784(14)	gamma=90
Temperature:	170 K		

	Calculated	Reported
Volume	1822.3(8)	1822.3(9)
Space group	P 21/c	P 1 21/c 1
Hall group	-P 2ybc	-P 2ybc
Moiety formula	C21 H23 N O2 S	C21 H23 N O2 S
Sum formula	C21 H23 N O2 S	C21 H23 N O2 S
Mr	353.46	353.46
Dx,g cm-3	1.288	1.288
Z	4	4
Mu (mm-1)	0.192	0.192
F000	752.0	752.0
F000'	752.79	
h,k,lmax	12,16,18	12,16,18
Nref	4015	4012
Tmin,Tmax	0.957,0.975	0.699,0.746
Tmin'	0.912	

Correction method= # Reported T Limits: Tmin=0.699 Tmax=0.746
AbsCorr = MULTI-SCAN

Data completeness= 0.999   Theta(max)= 27.096

R(reflections)= 0.0319(3560)   wR2(reflections)= 0.0845(4012)

S = 1.039   Npar= 226

Supplementary Fig. 12 X-ray analysis of 2q. Detail X-ray crystallographic data of **2q**.

3-(benzylamino)-2,2-dimethyl-4-(thiophen-2-yl)-2*H*-thiete 1,1-dioxide (2a’)

CCDC Number = 1999866

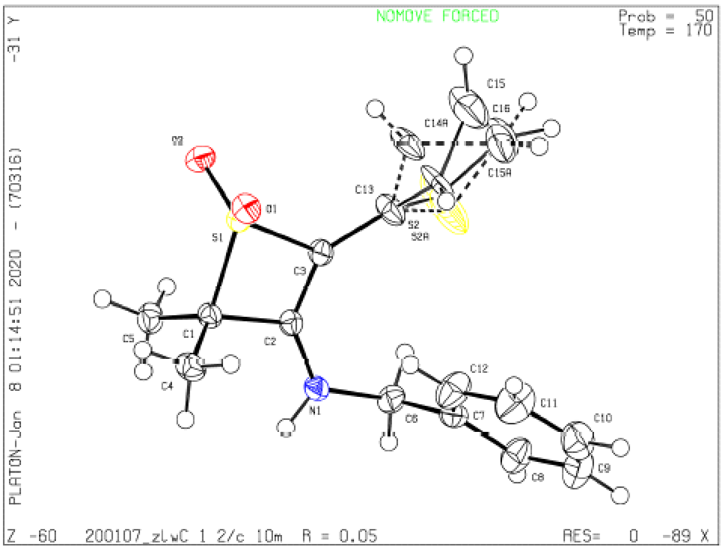

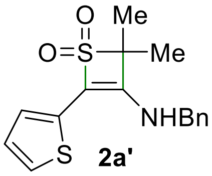

Bond precision:	C-C = 0.0033 A		Wavelength=0.71073
Cell:	a=24.138(10)	b=10.432(4)	c=13.397(5)
	alpha=90	beta=113.679(18)	gamma=90
Temperature:	170 K		

	Calculated	Reported
Volume	3090(2)	3090(2)
Space group	C 2/c	C 1 2/c 1
Hall group	-C 2yc	-C 2yc
Moiety formula	C16 H17 N O2 S2	C16 H17 N O2 S2
Sum formula	C16 H17 N O2 S2	C16 H17 N O2 S2
Mr	319.43	319.42
Dx,g cm-3	1.373	1.373
Z	8	8
Mu (mm-1)	0.348	0.348
F000	1344.0	1344.0
F000’	1346.47	
h,k,lmax	30,13,17	30,13,17
Nref	3410	3405
Tmin,Tmax	0.873,0.976	0.696,0.746
Tmin’	0.873	

Correction method= # Reported T Limits: Tmin=0.696 Tmax=0.746
AbsCorr = MULTI-SCAN

Data completeness= 0.999 Theta(max)= 27.117

R(reflections)= 0.0466(3028) wR2(reflections)= 0.1324(3405)

S = 1.070 Npar= 220

Supplementary Fig. 13 X-ray analysis of 2a’. Detail X-ray crystallographic data of **2a’**.

13. DFT calculations

Density functional theory (DFT) calculations were conducted at the level of M06[9,10] using the Gaussian 09 suite[11] of computational programs. The 6-31G(d,p) basis set[12,13] was applied for the C, H, O, N, S and Li atoms. The initial α-lithiation of ynamides was verified by the α-methylation experiment. We think the following intramolecular cyclization is the essential step of the reaction. We conducted DFT calculations to compare several possible cyclization modes in the skeleton reorganizations.

In the LDA-mediated process, there are three possible cyclizations might occur after α-lithiation, resulting in **Int-B**, **Int-C** or **Int-D**. The DFT calculations showed that **Path 1** is more favored, in which **Int-A** transforms to **Int-B** via **TS$_1$** with the barrier of 17.6 kcal/mol (Supplementary Fig. 14).

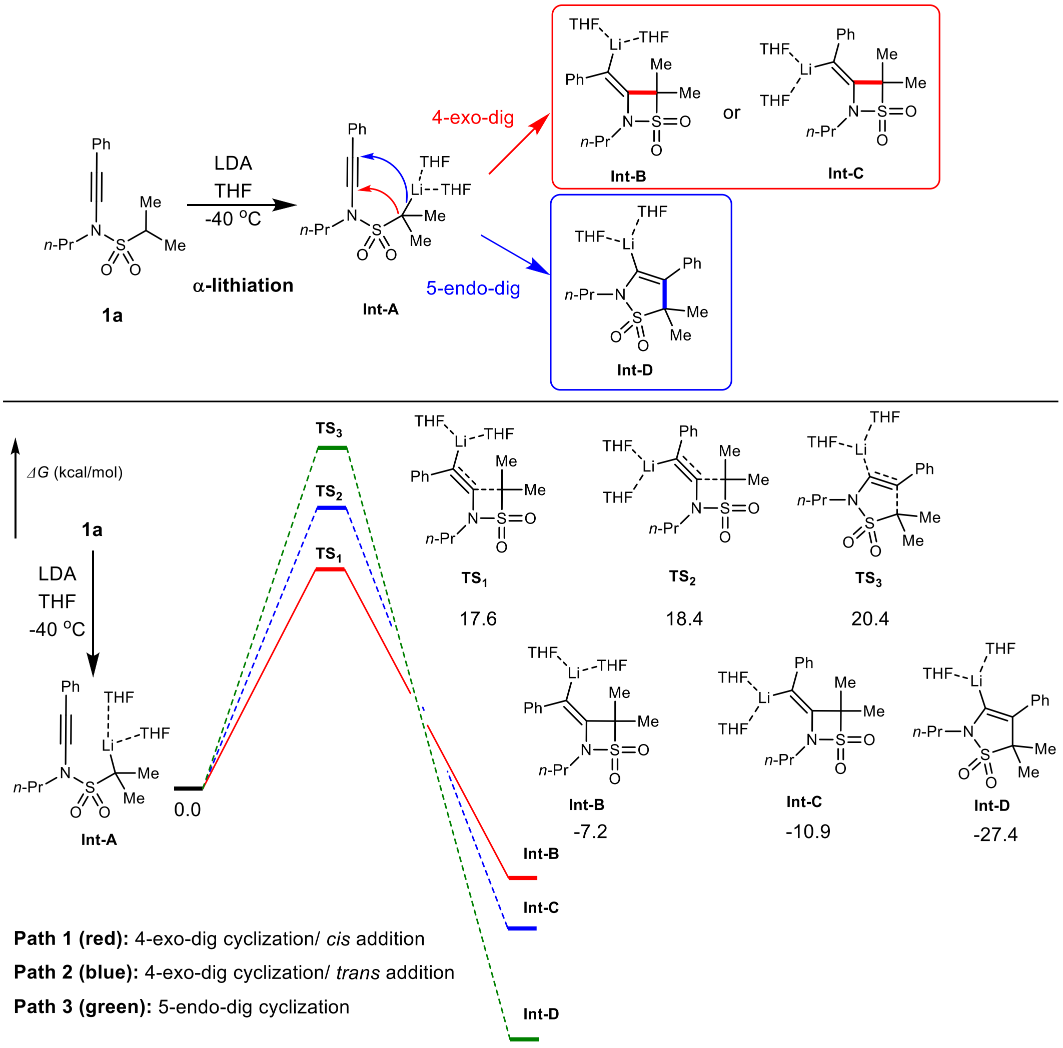

Supplementary Fig. 14. DFT calculations for the LDA-mediated cyclization in the skeleton reorganization. Free energy profiles (kcal/mol) of three possible cyclizations from **Int-A**. path 1 (red): 4-exo-dig cyclization/ *cis* addition leading to **Int-B** (via **TS$_1$**), path 2 (blue): 4-exo-dig cyclization/ *trans* addition

leading to **Int-C** (via **TS$_2$**), path 3 (green): 5-endo-dig cyclization leading to **Int-D** (via **TS$_3$**).

In the LDA/DMPU-mediated process, three similar paths may occur, resulting in **Int-F**, **Int-G** or **Int-H**. The DFT calculations showed that **Path 4** is more favored, in which **Int-E** transforms to **Int-F** via **TS$_4$** with a lower barrier of 22.5 kcal/mol (Supplementary Fig. 15).

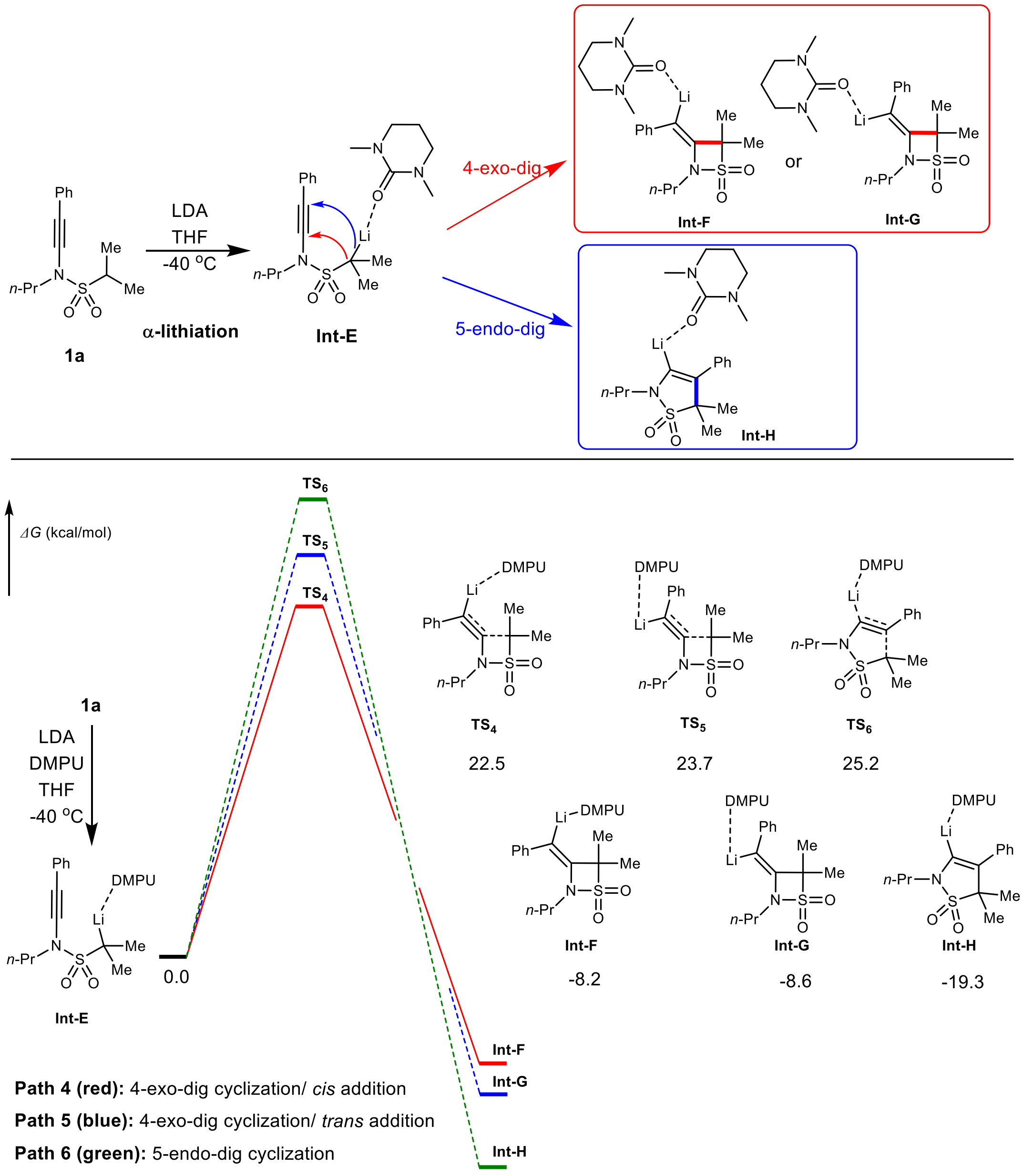

Supplementary Fig. 15. DFT calculations for the LDA/DMPU-mediated cyclization in the skeleton reorganization. Free energy profiles (kcal/mol) of three possible cyclizations from **Int-E**. path 4 (red): 4-exo-dig cyclization/ *cis* addition leading to **Int-F** (via **TS$_4$**), path 5 (blue): 4-exo-dig cyclization/ *trans* addition leading to **Int-G** (via **TS$_5$**), path 6 (green): 5-endo-dig cyclization leading to **Int-H** (via **TS$_6$**).

14. Copies of NMR Spectra

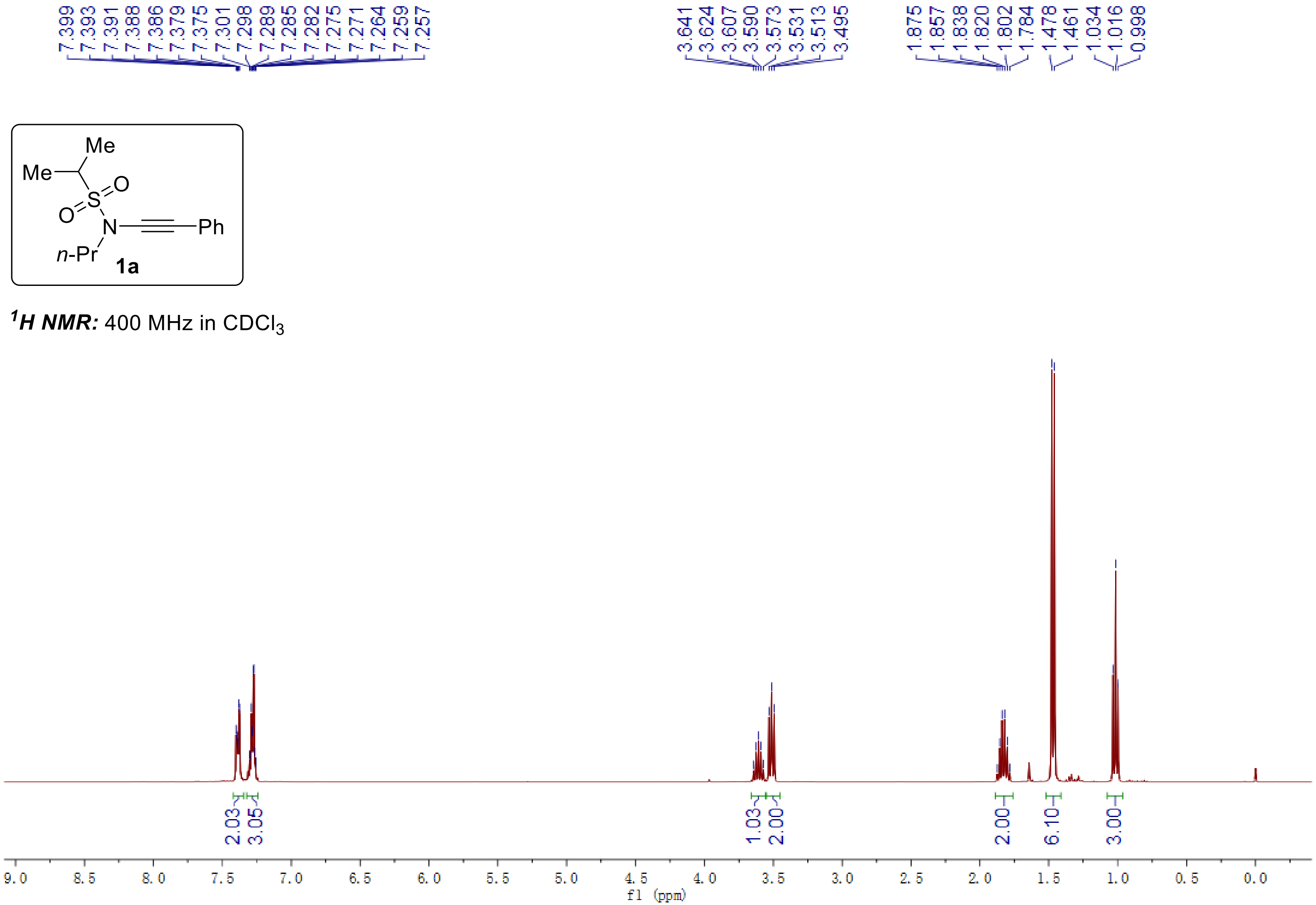

^{1}***H NMR:*** 400 MHz in $CDCl_3$

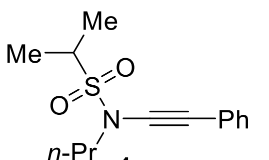

1a

^{13}C NMR: 100 MHz in $CDCl_3$

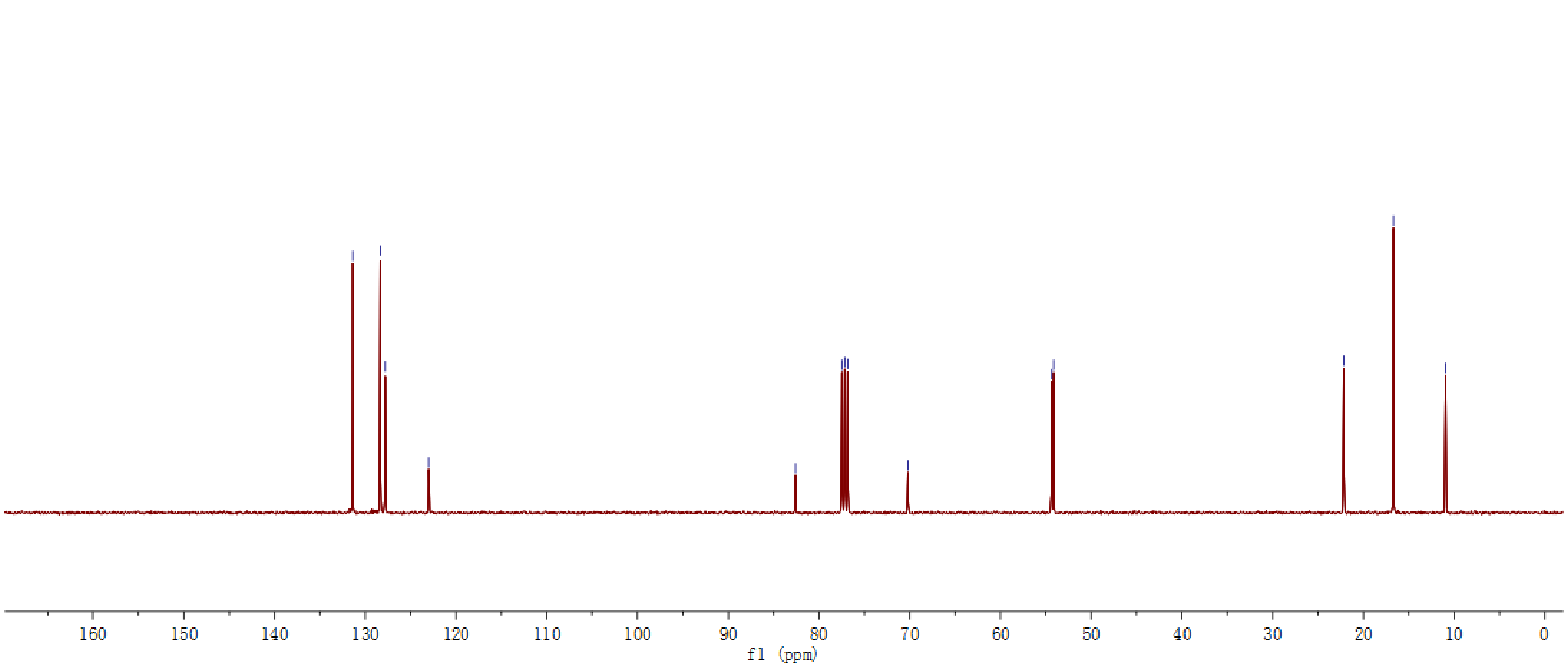

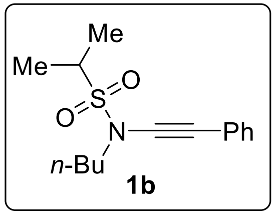

^{1}**H NMR:** 400 MHz in $CDCl_3$

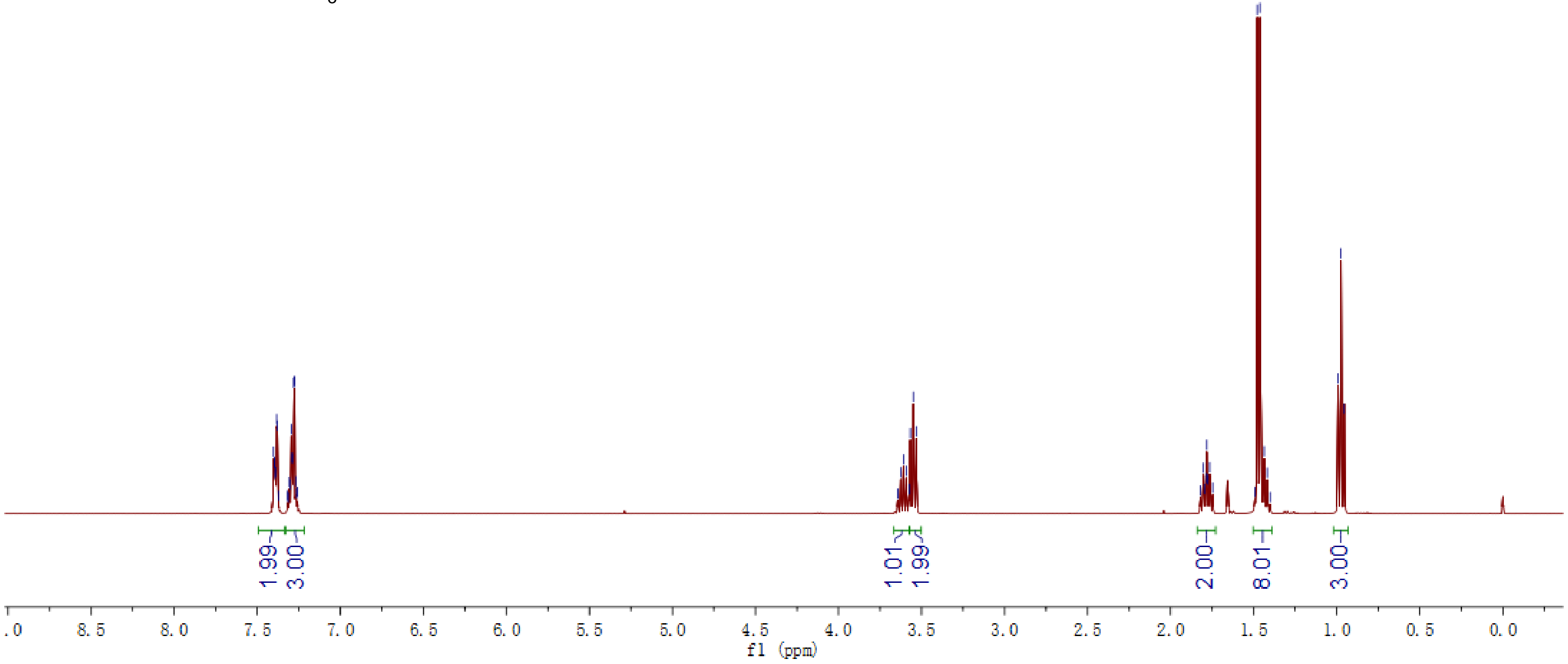

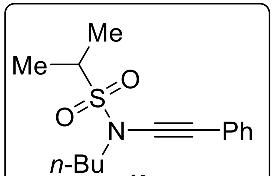

1b

^{13}C NMR: 100 MHz in $CDCl_3$

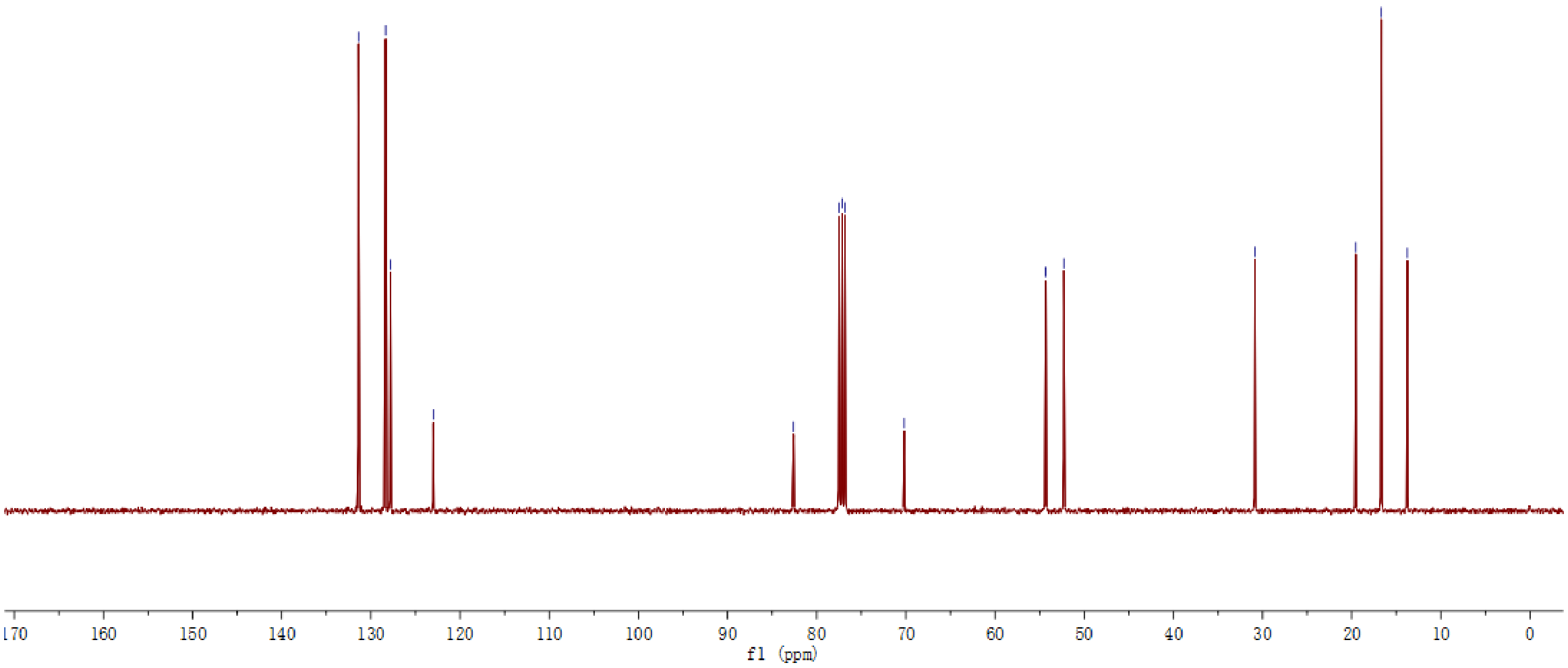

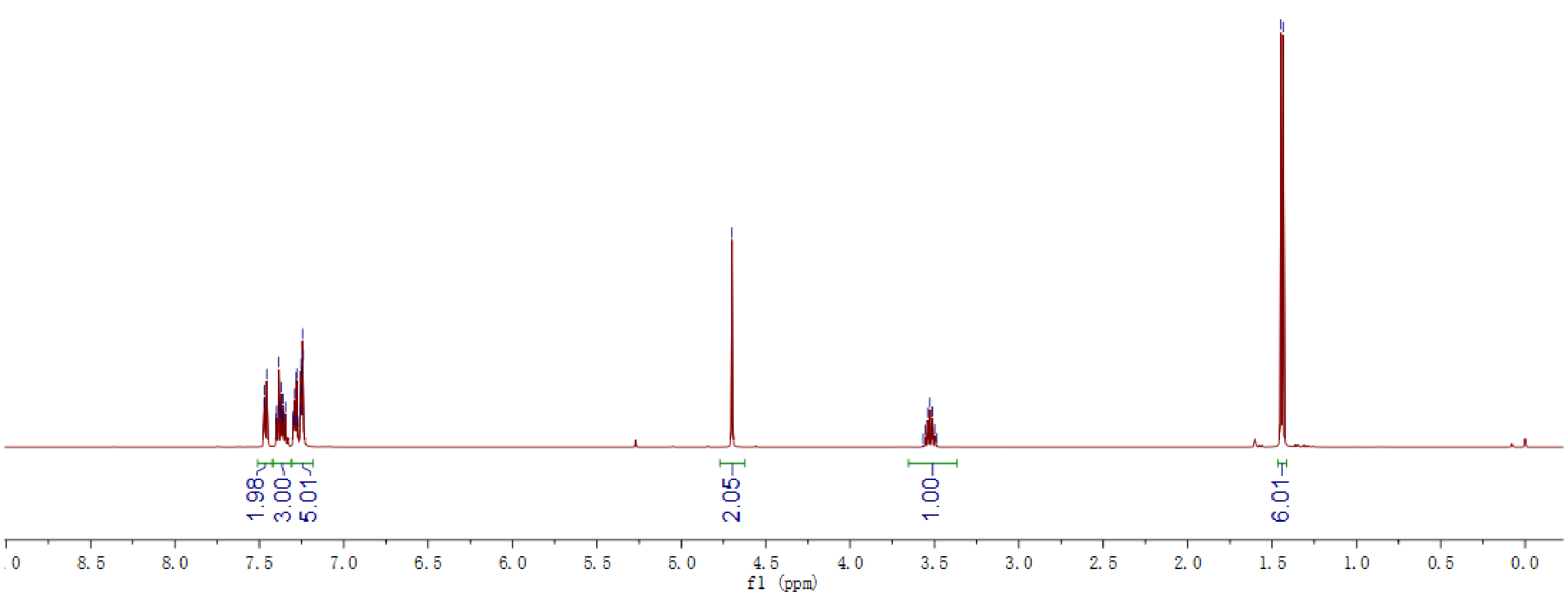

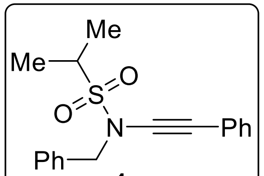

1c

¹H NMR: 500 MHz in $CDCl_3$

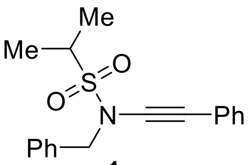

1c

[13]***C NMR:*** 125 MHz in $CDCl_3$

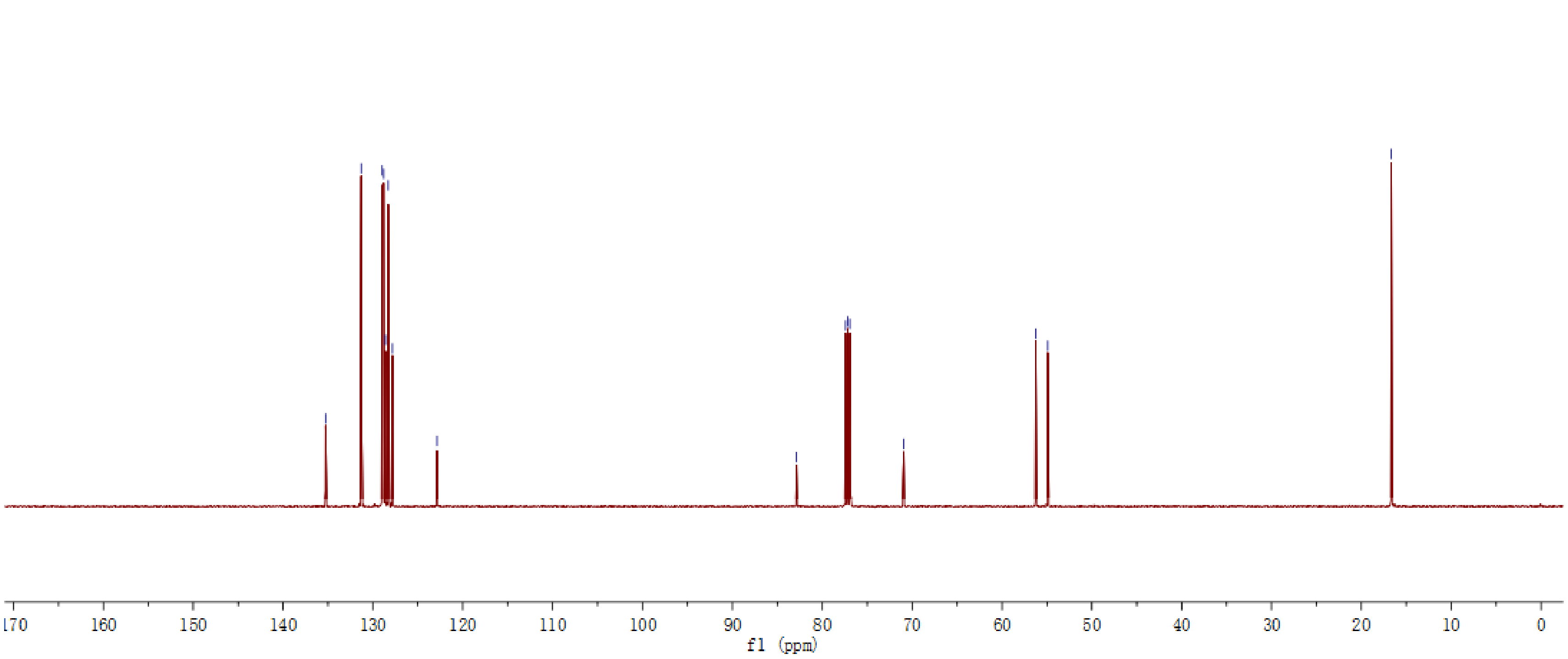

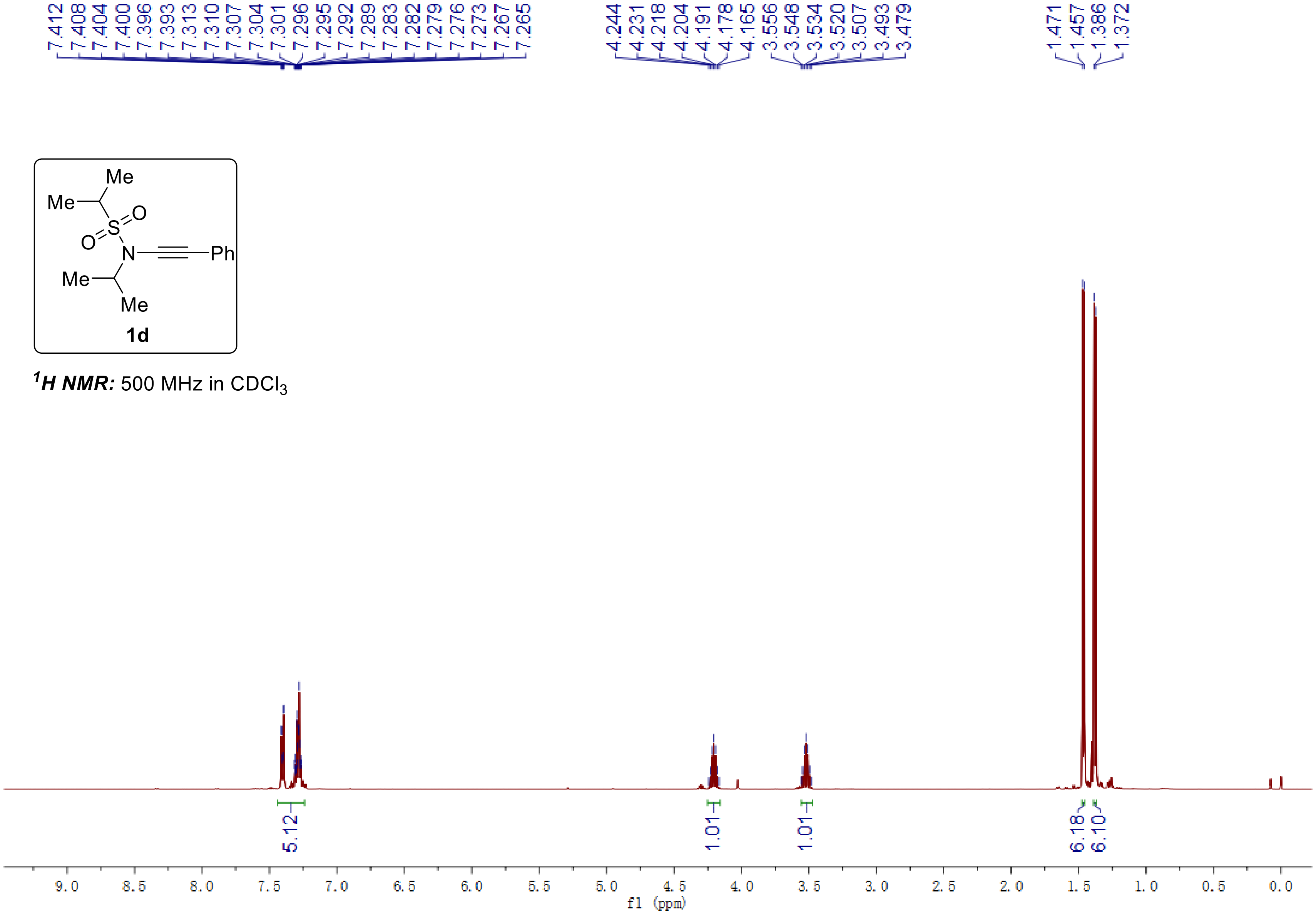

^{1}H NMR: 500 MHz in $CDCl_3$

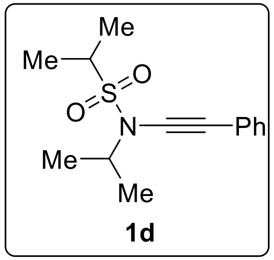
Me
Me
S
O
O
N
Ph
Me
Me
1d

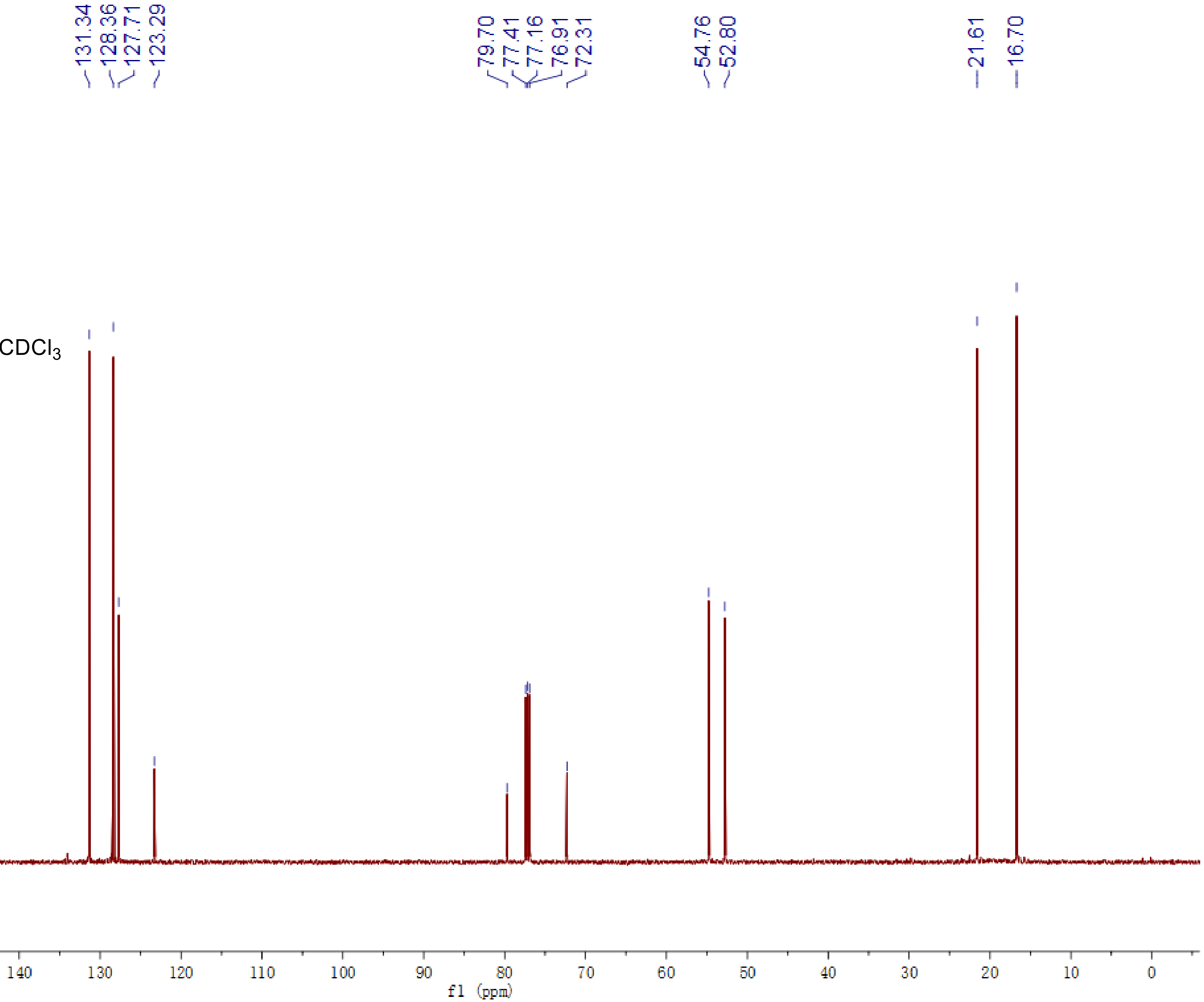
13C NMR: 125 MHz in CDCl3
131.34
128.36
127.71
123.29
79.70
77.41
77.16
76.91
72.31
54.76
52.80
21.61
16.70
170 160 150 140 130 120 110 100 90 80 70 60 50 40 30 20 10 0
f1 (ppm)

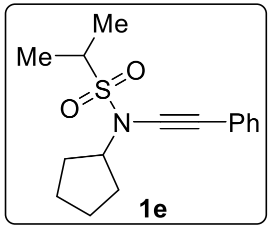

^{1}***H NMR:*** 400 MHz in $CDCl_3$

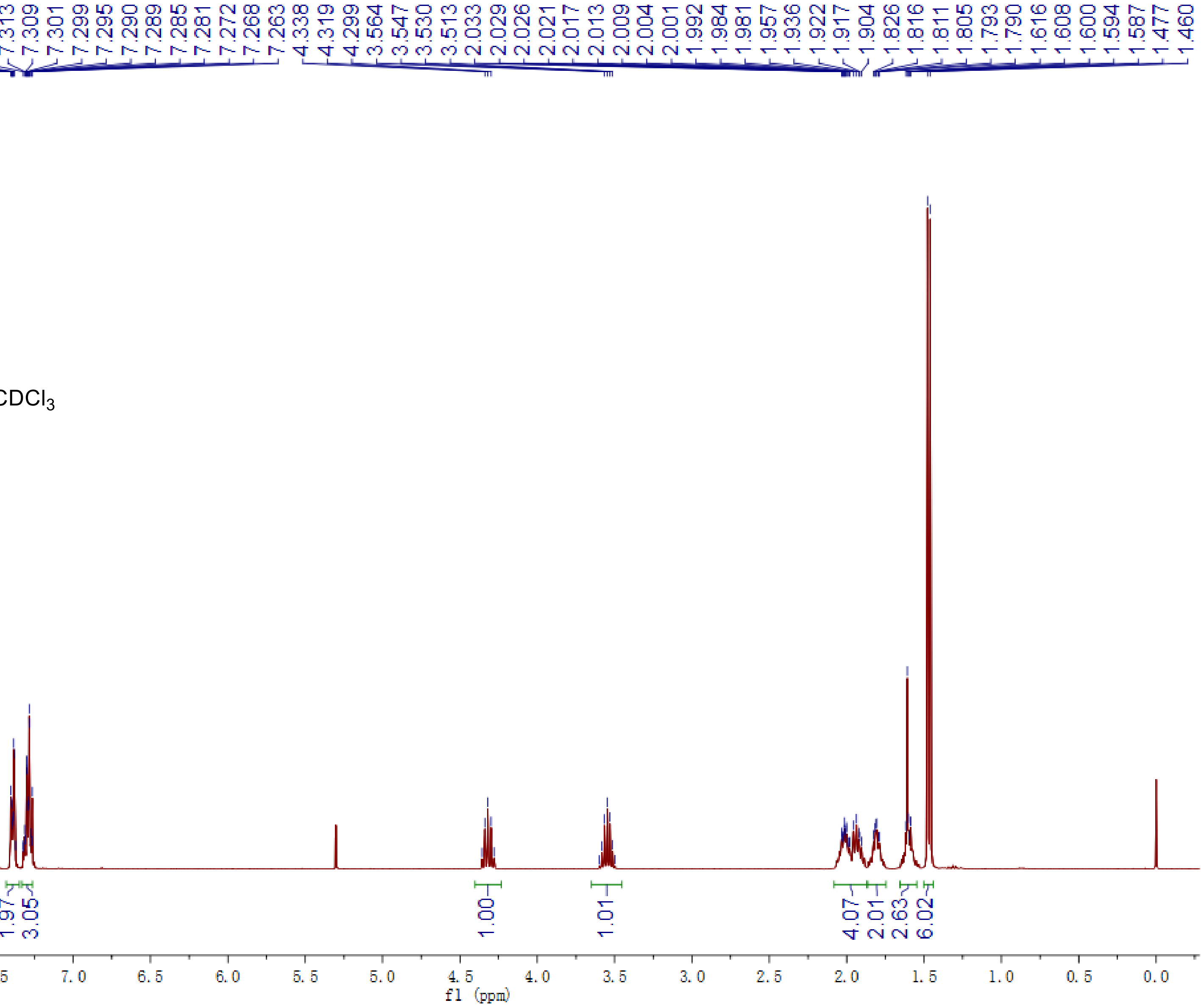

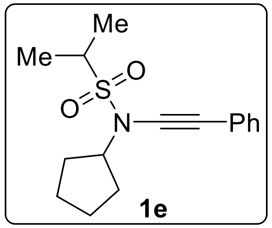

1e

^{13}C NMR: 100 MHz in $CDCl_3$

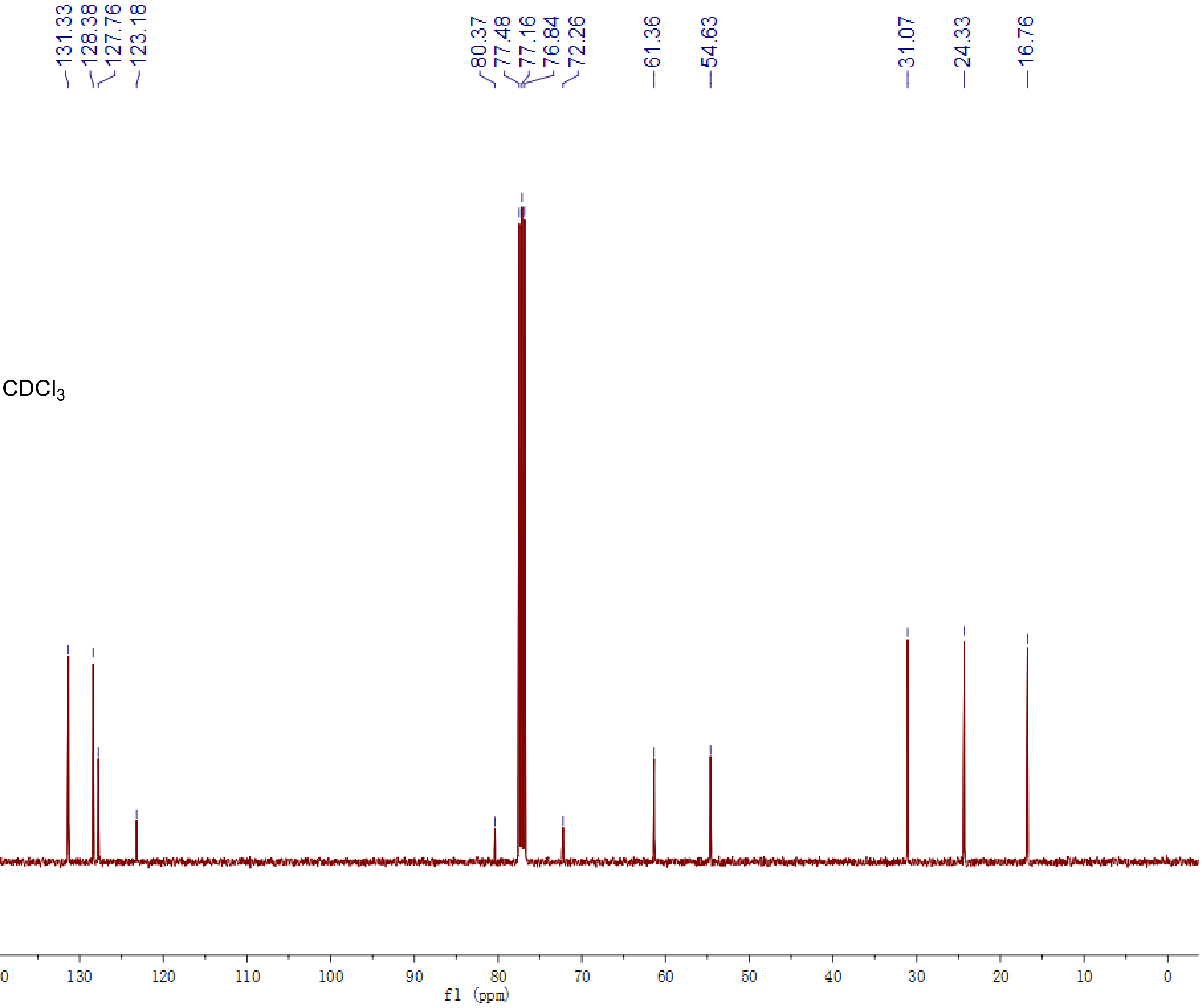

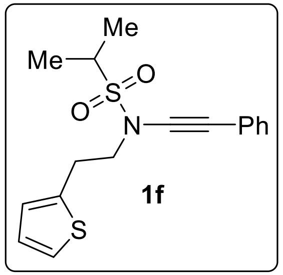

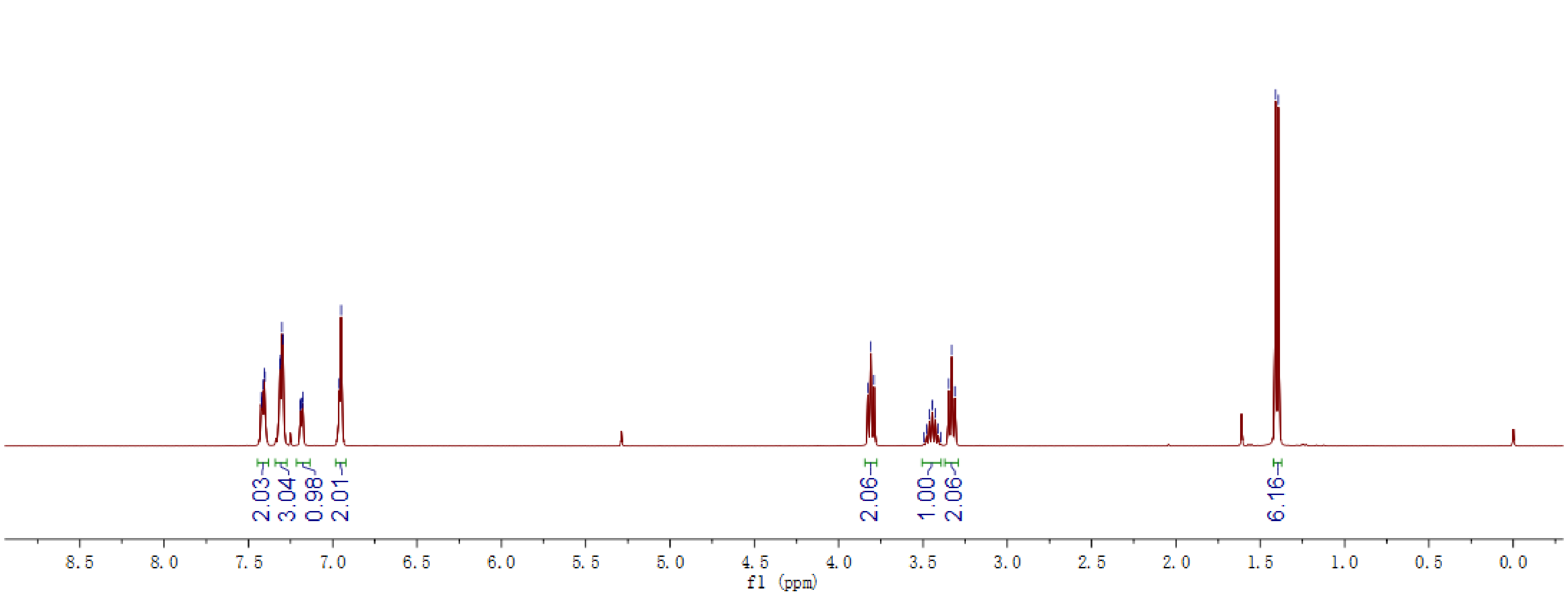

^{1}H NMR: 400 MHz in $CDCl_3$

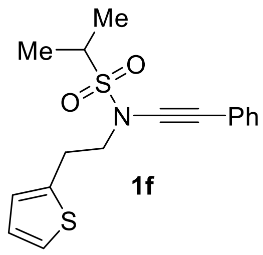

1f

^{13}C NMR: 100 MHz in $CDCl_3$

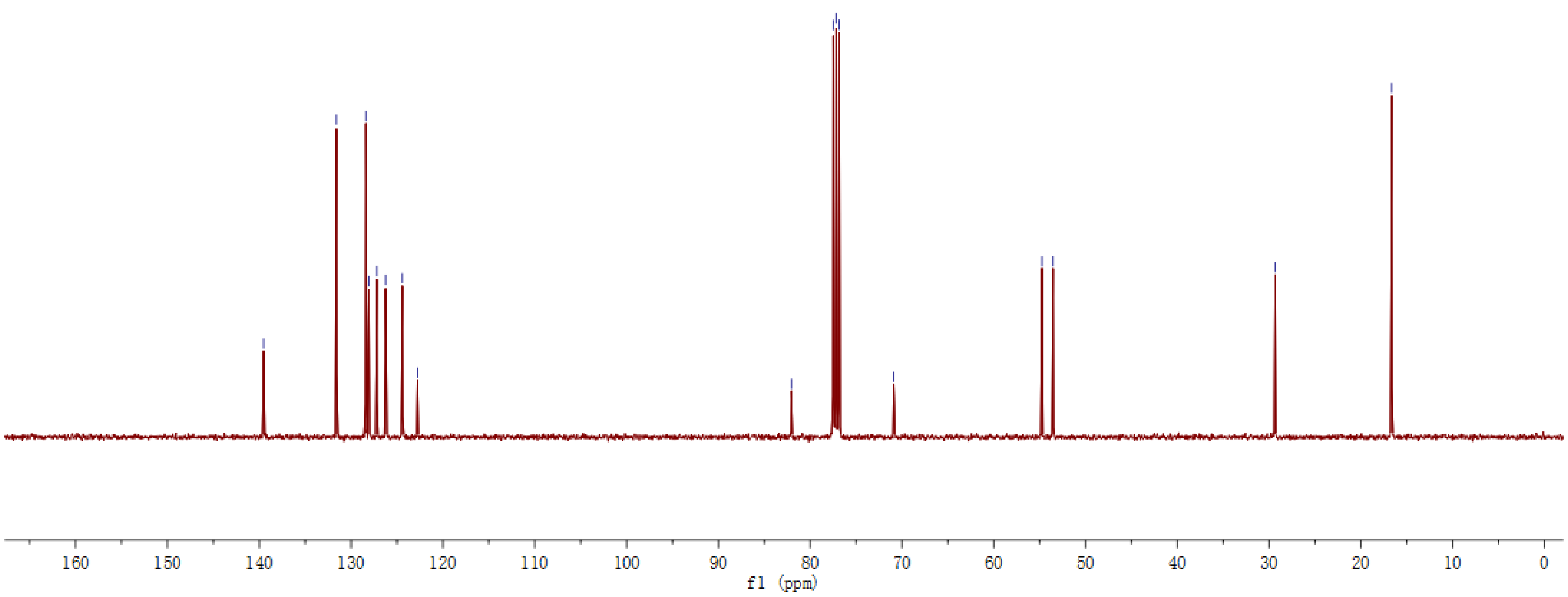

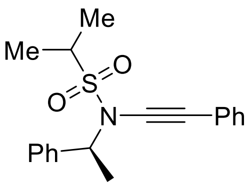

1g

^{1}H NMR: 400 MHz in $CDCl_3$

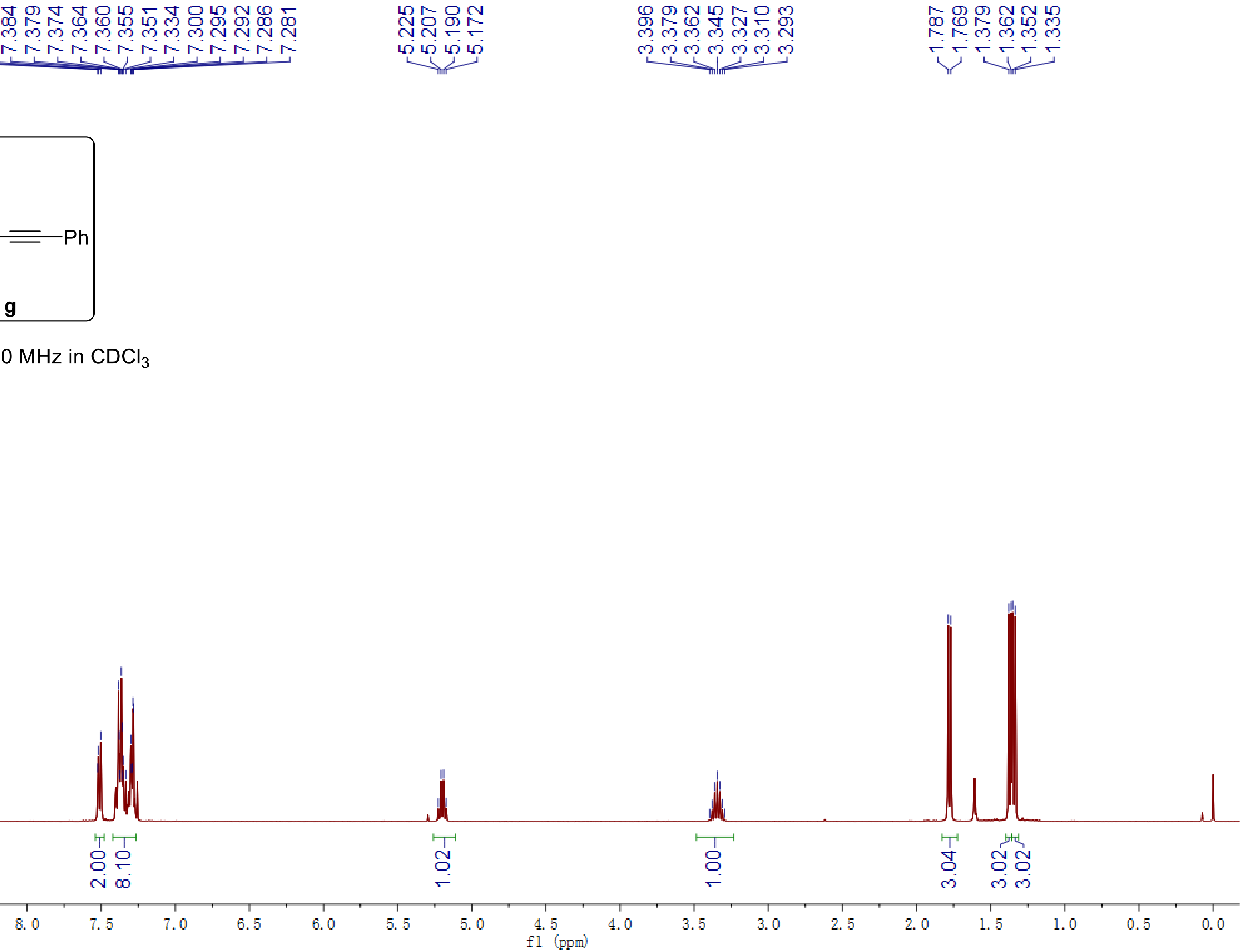

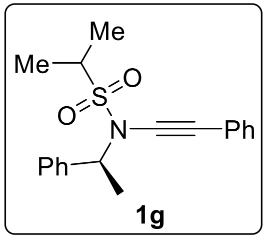

***13*C NMR:** 100 MHz in $CDCl_3$

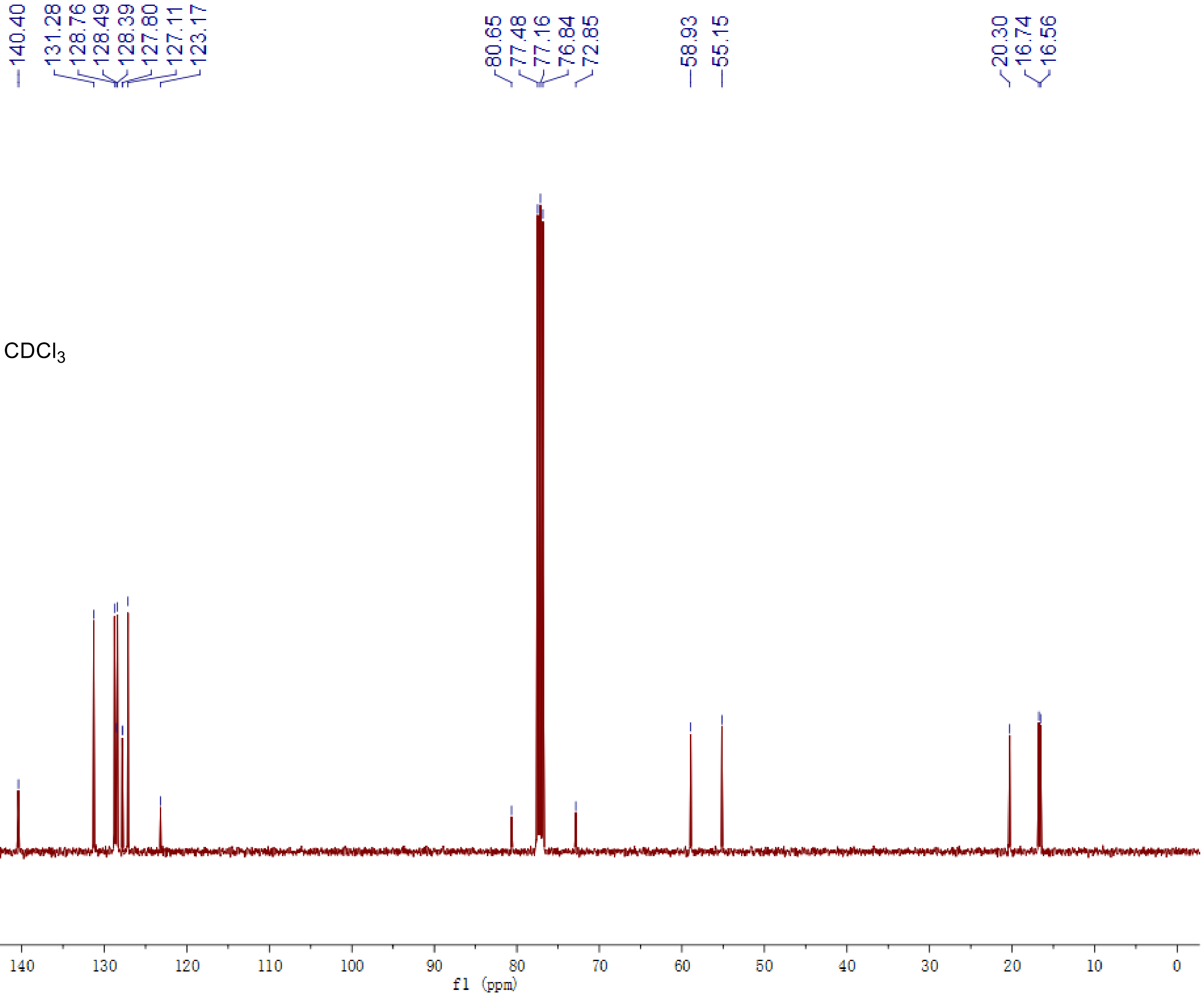

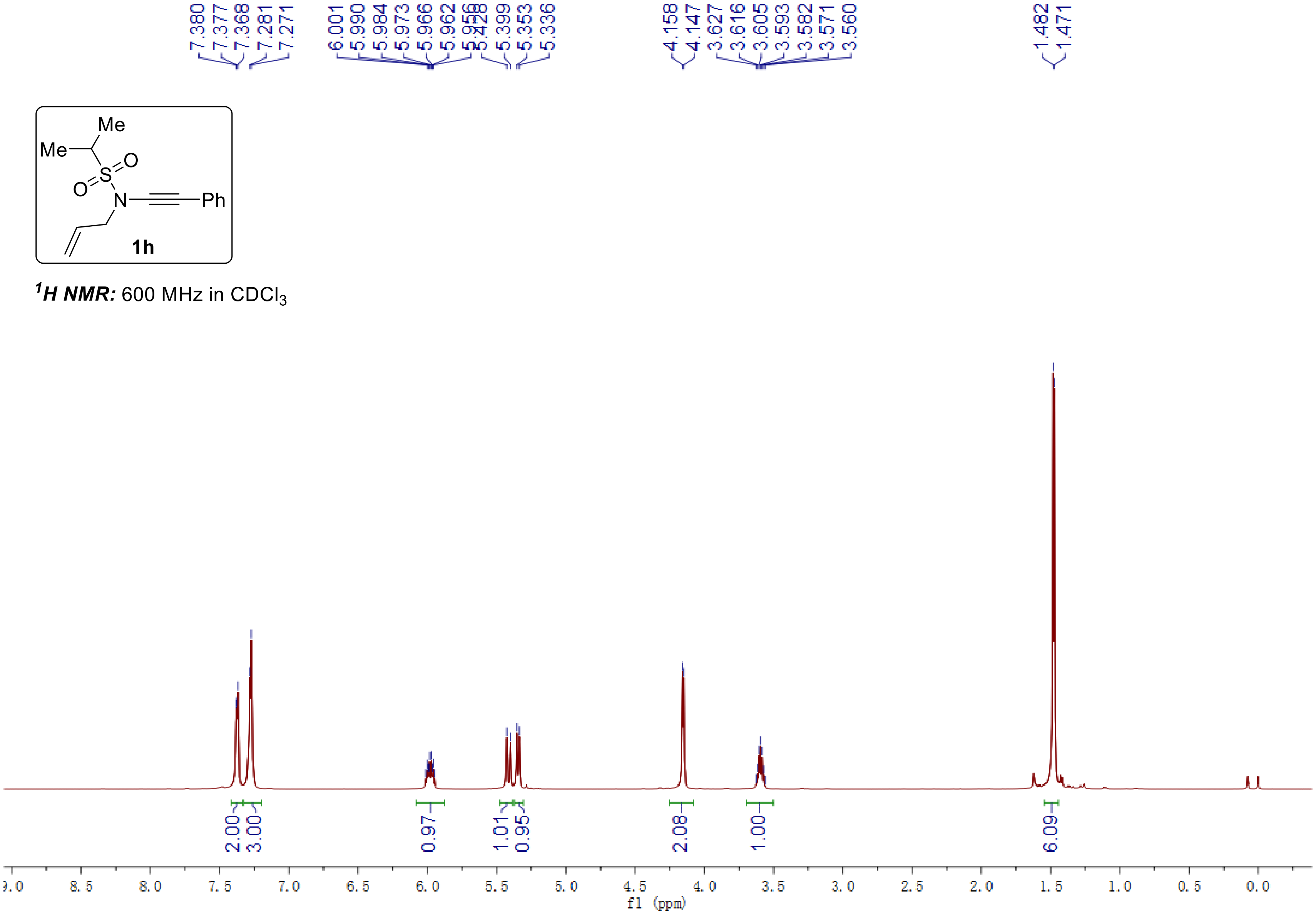

^{1}H NMR: 600 MHz in $CDCl_3$

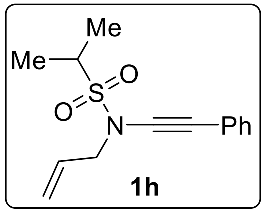

1h

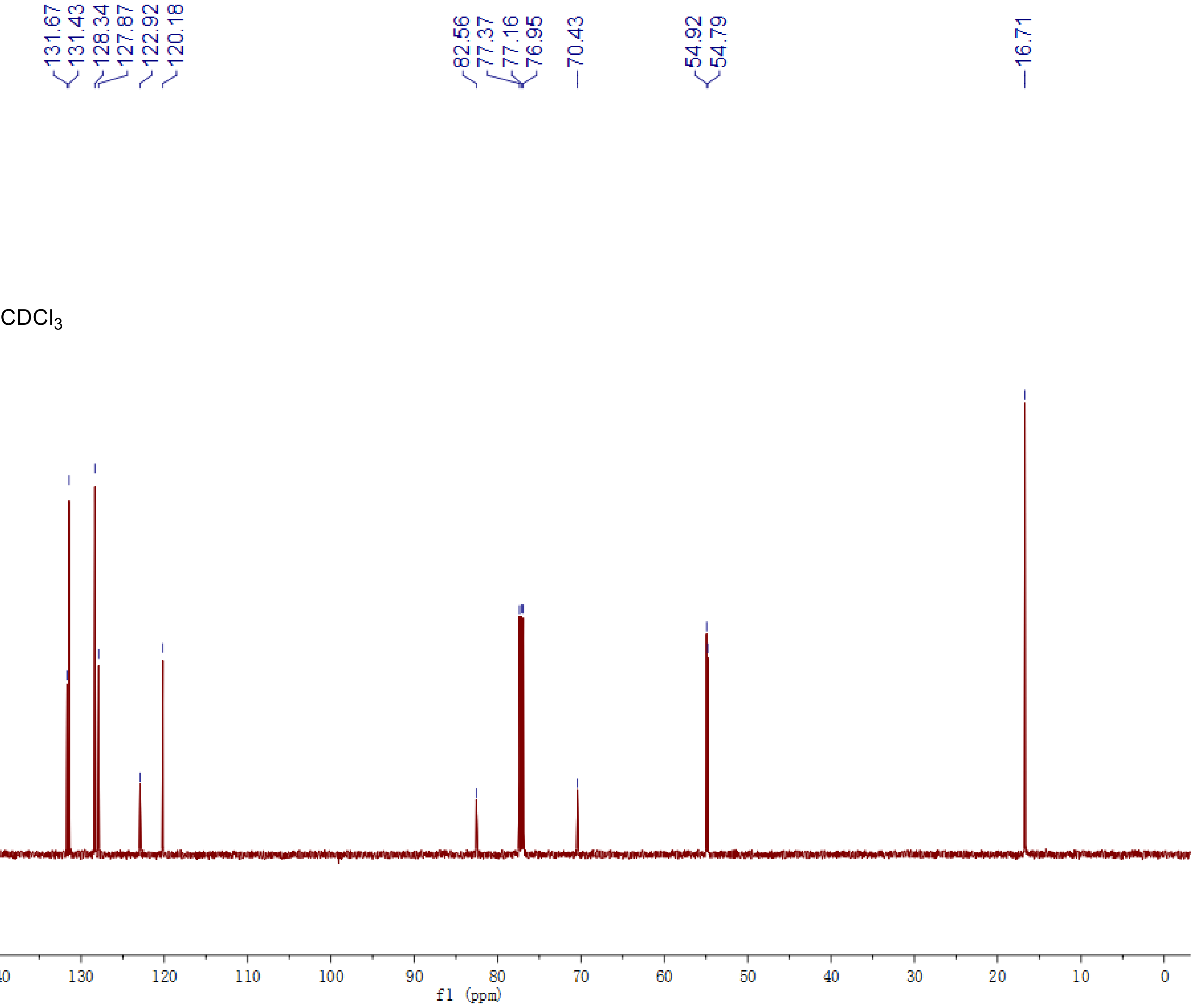

^{13}C NMR: 150 MHz in $CDCl_3$

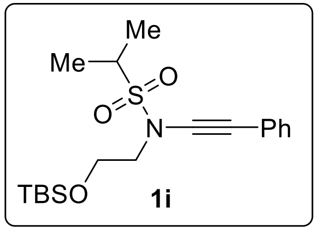

^{1}**H NMR:** 400 MHz in $CDCl_3$

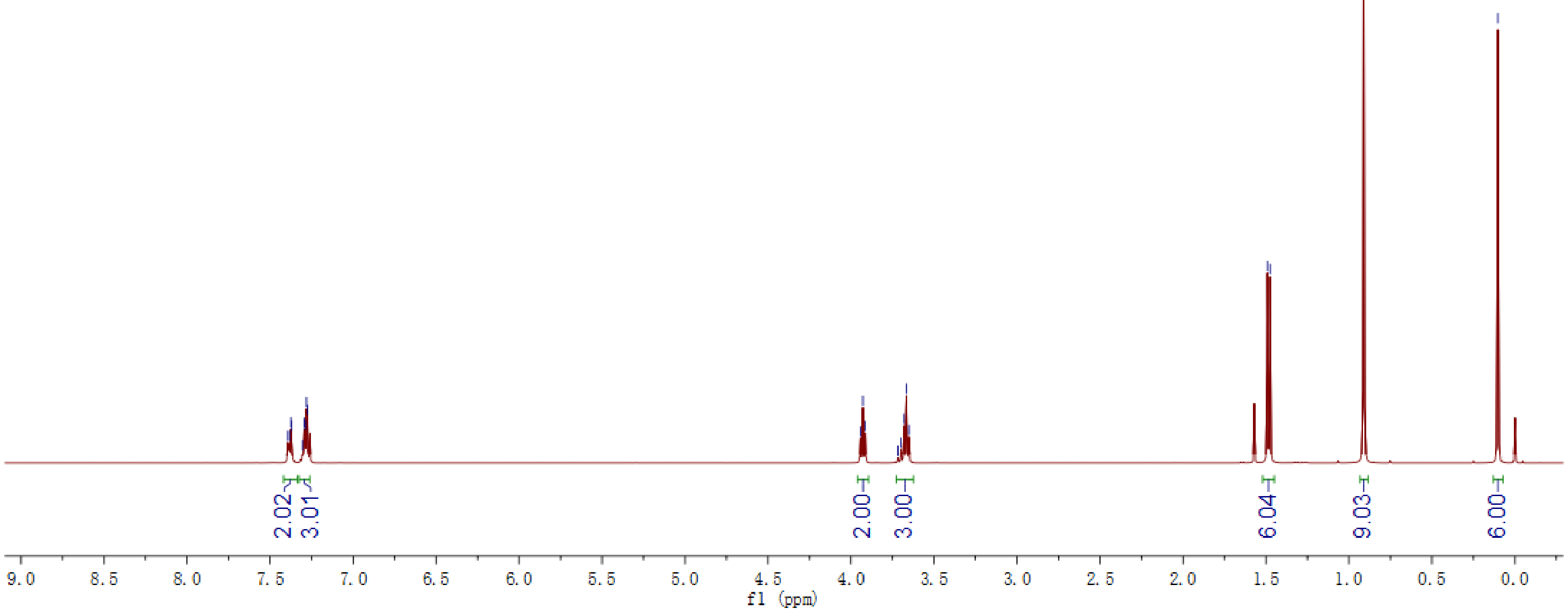

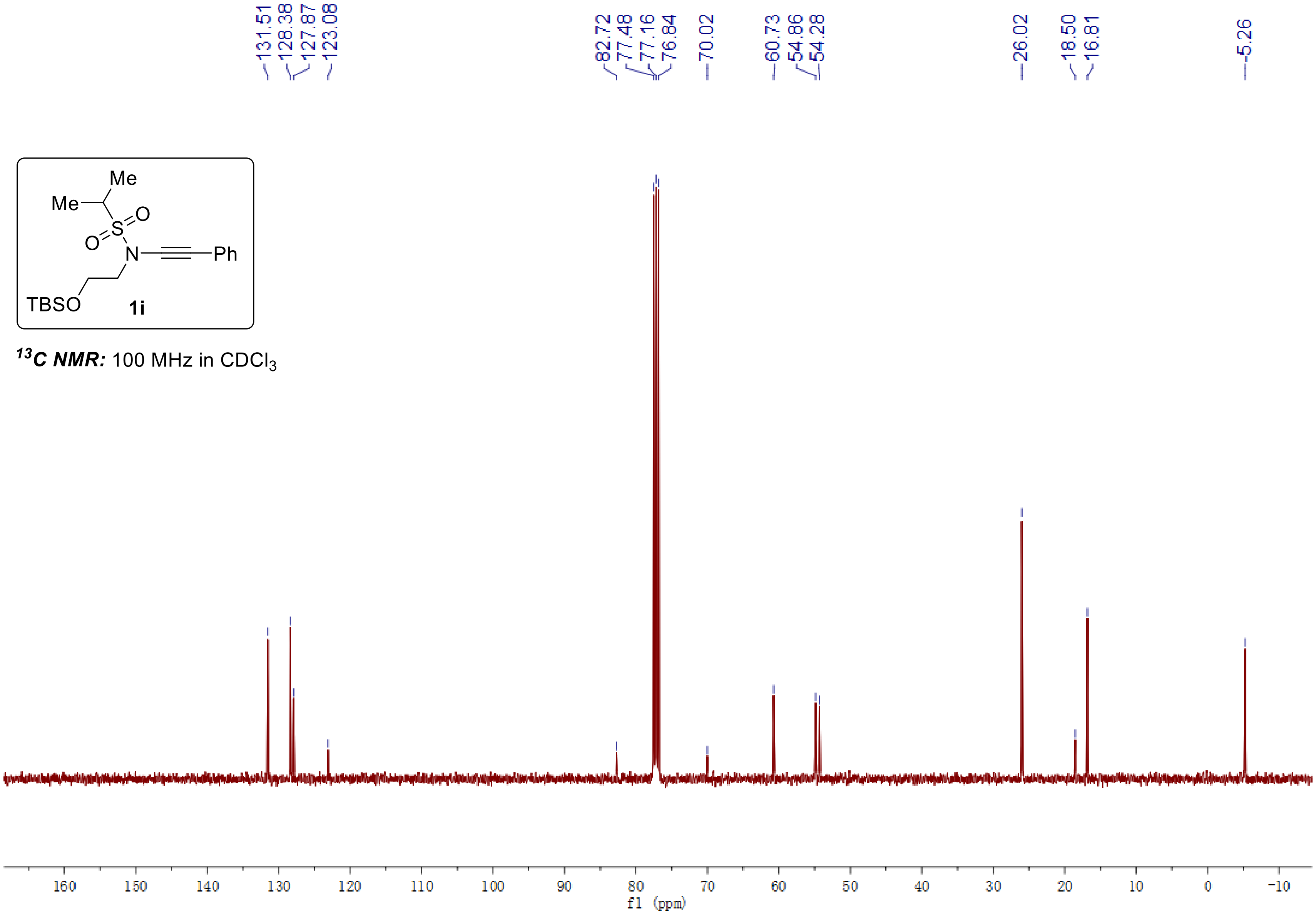
131.51
128.38
127.87
123.08
82.72
77.48
77.16
76.84
70.02
60.73
54.86
54.28
26.02
18.50
16.81
-5.26
Me
Me
O
S
O
N
Ph
TBSO
1i
^{13}C NMR: 100 MHz in $CDCl_3$
160
150
140
130
120
110
100
90
80
70
60
50
40
30
20
10
0
-10
f1 (ppm)

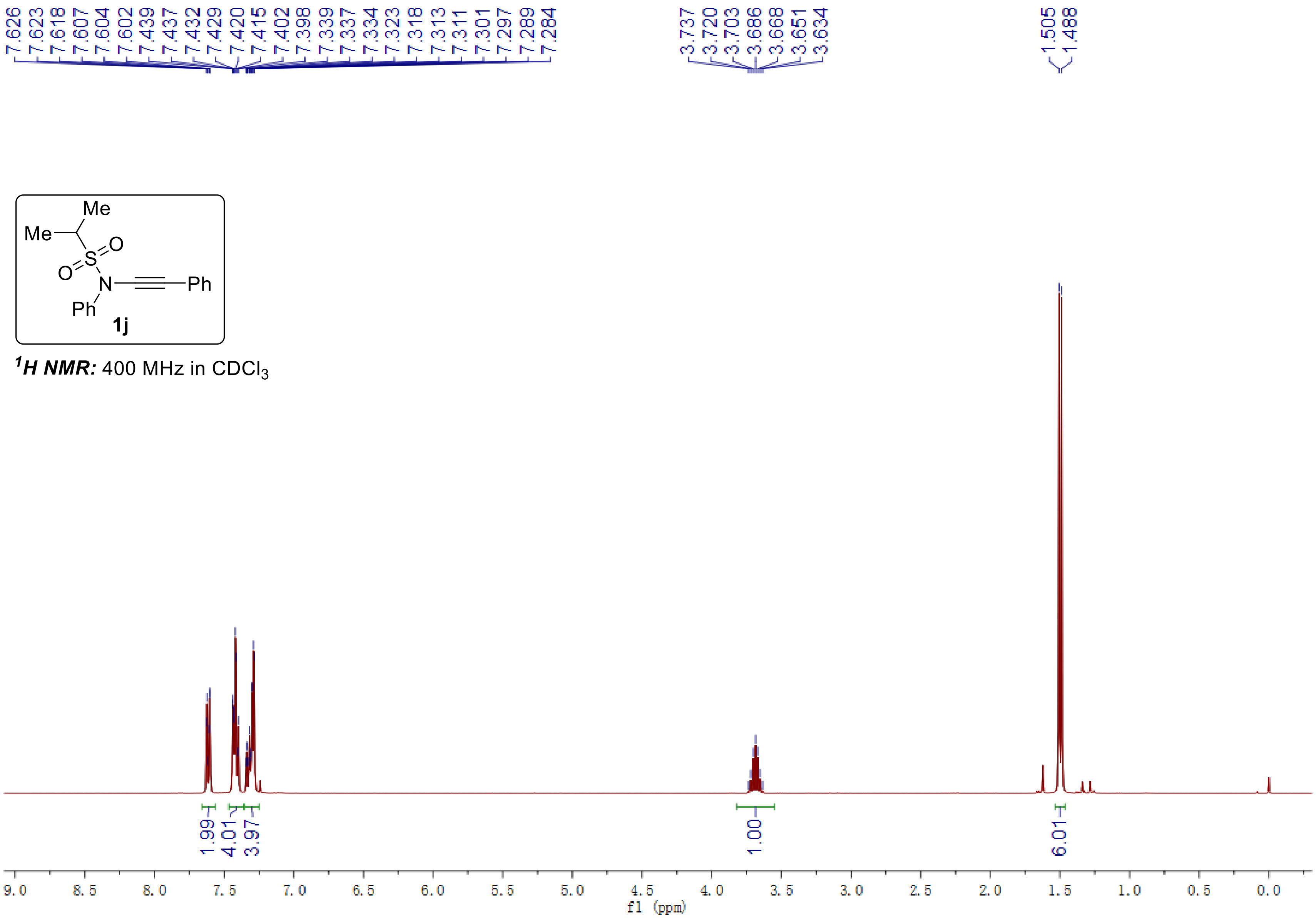

^{1}H NMR: 400 MHz in $CDCl_3$

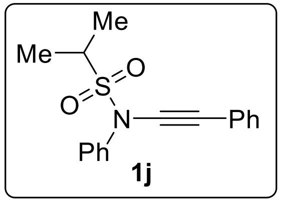

1j

^{13}C NMR: 100 MHz in $CDCl_3$

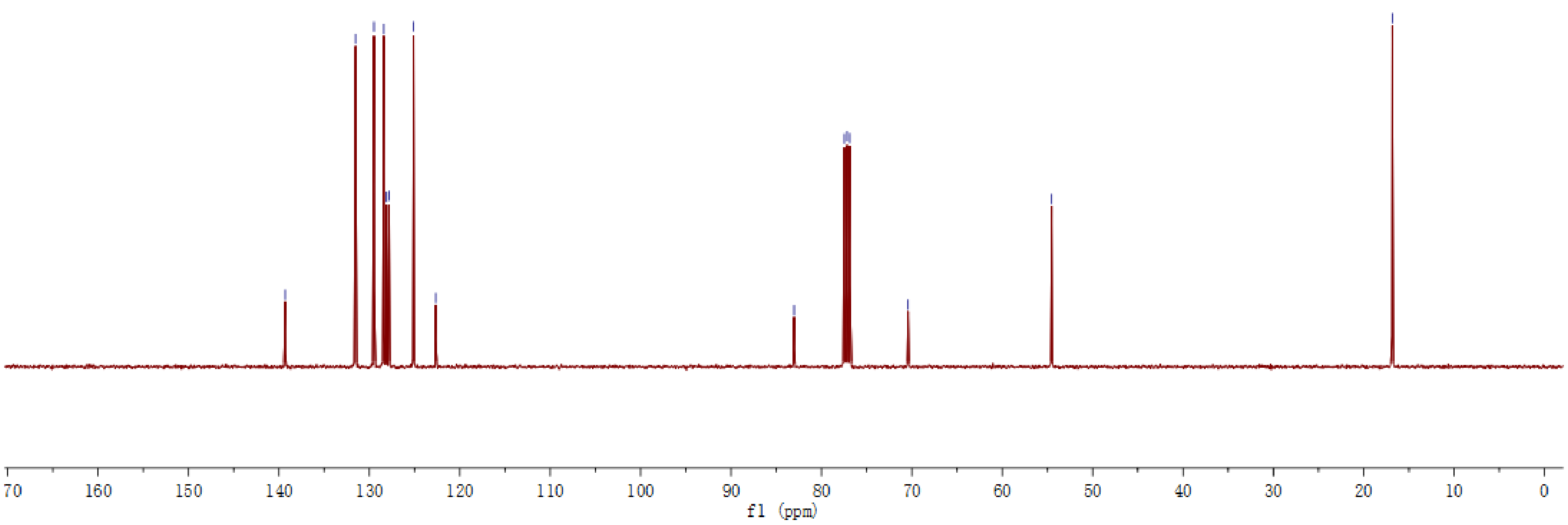

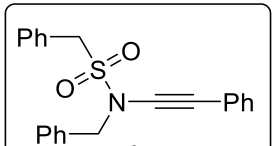

1m

^{1}H NMR: 400 MHz in $CDCl_3$

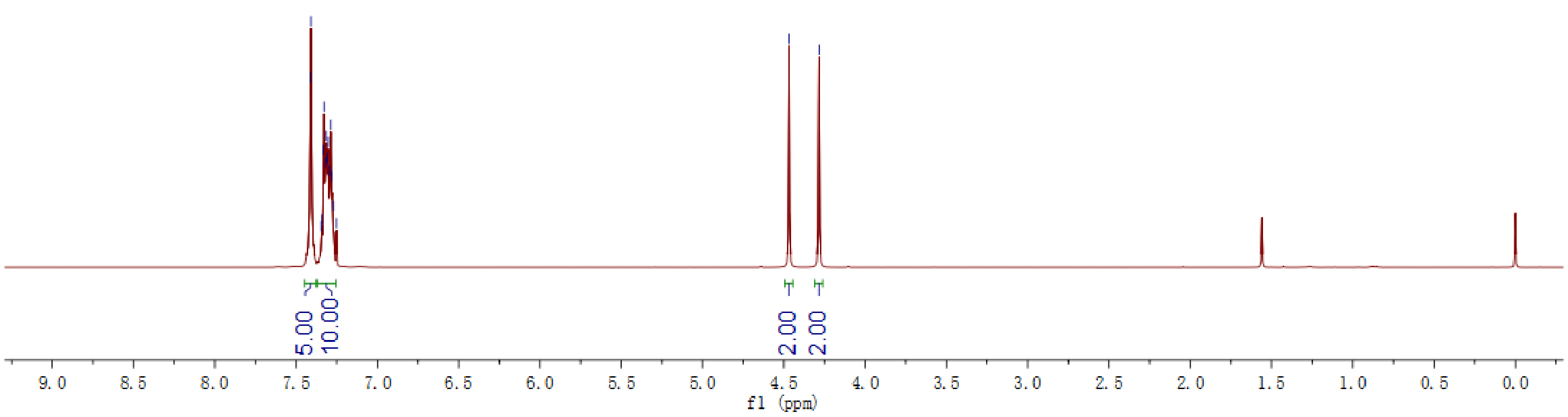

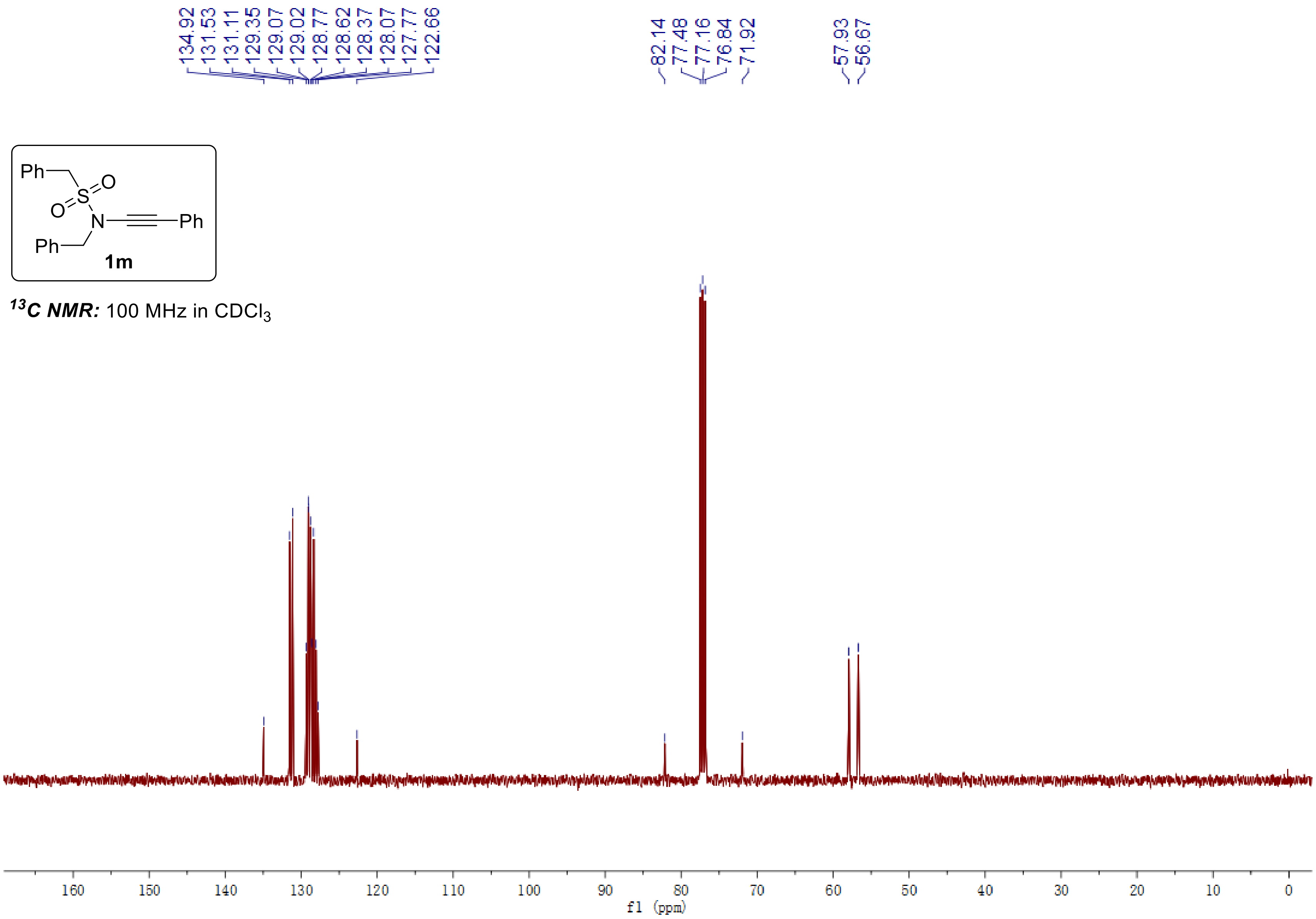

^{13}***C NMR:*** 100 MHz in $CDCl_3$

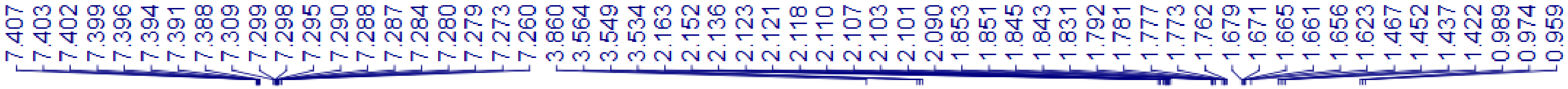

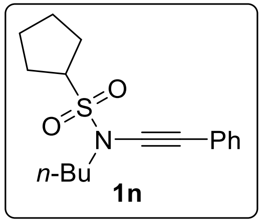

^{1}H NMR: 500 MHz in $CDCl_3$

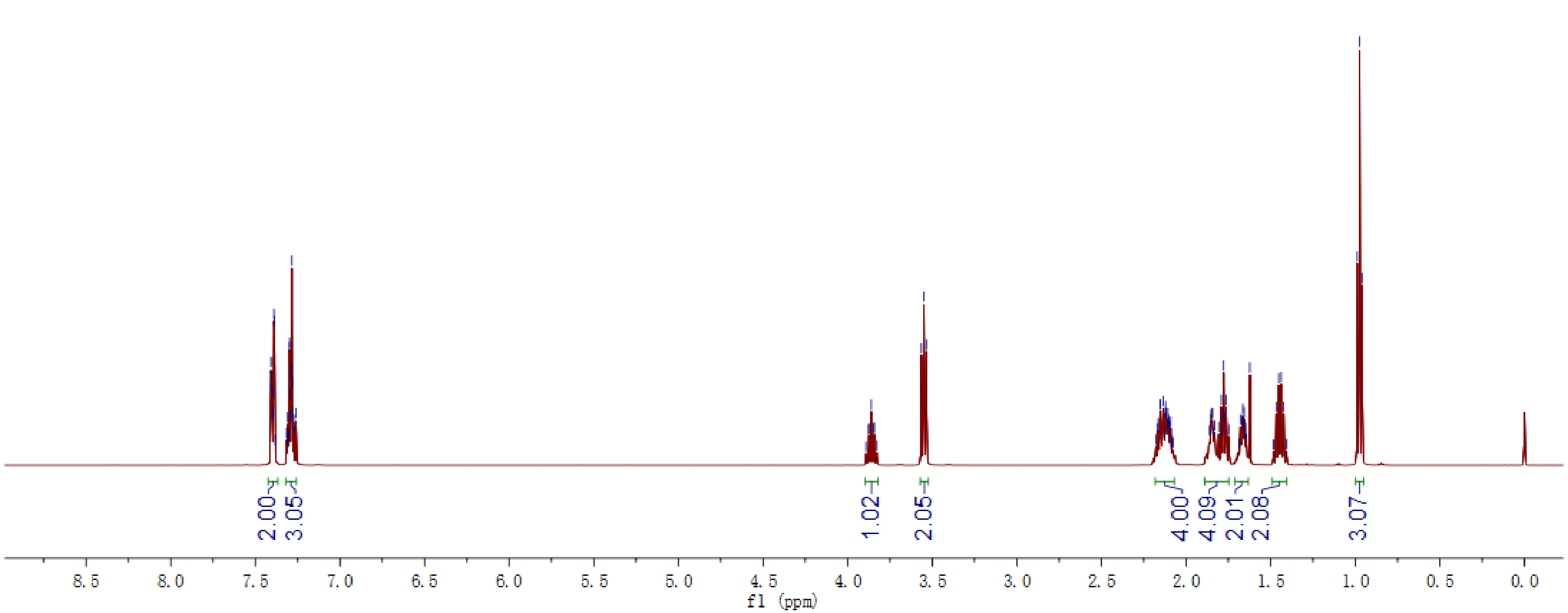

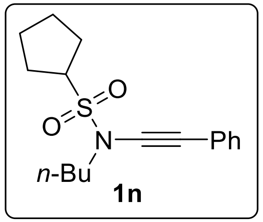

1n

^{13}C NMR: 125 MHz in $CDCl_3$

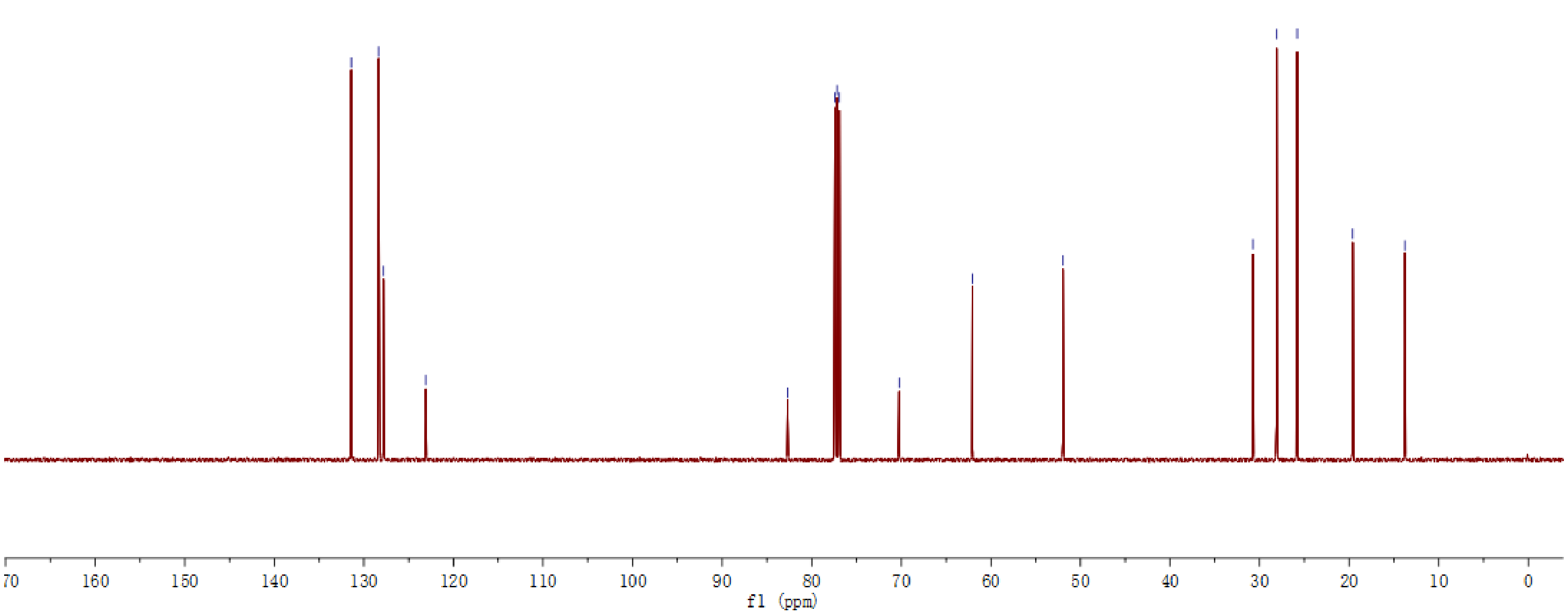

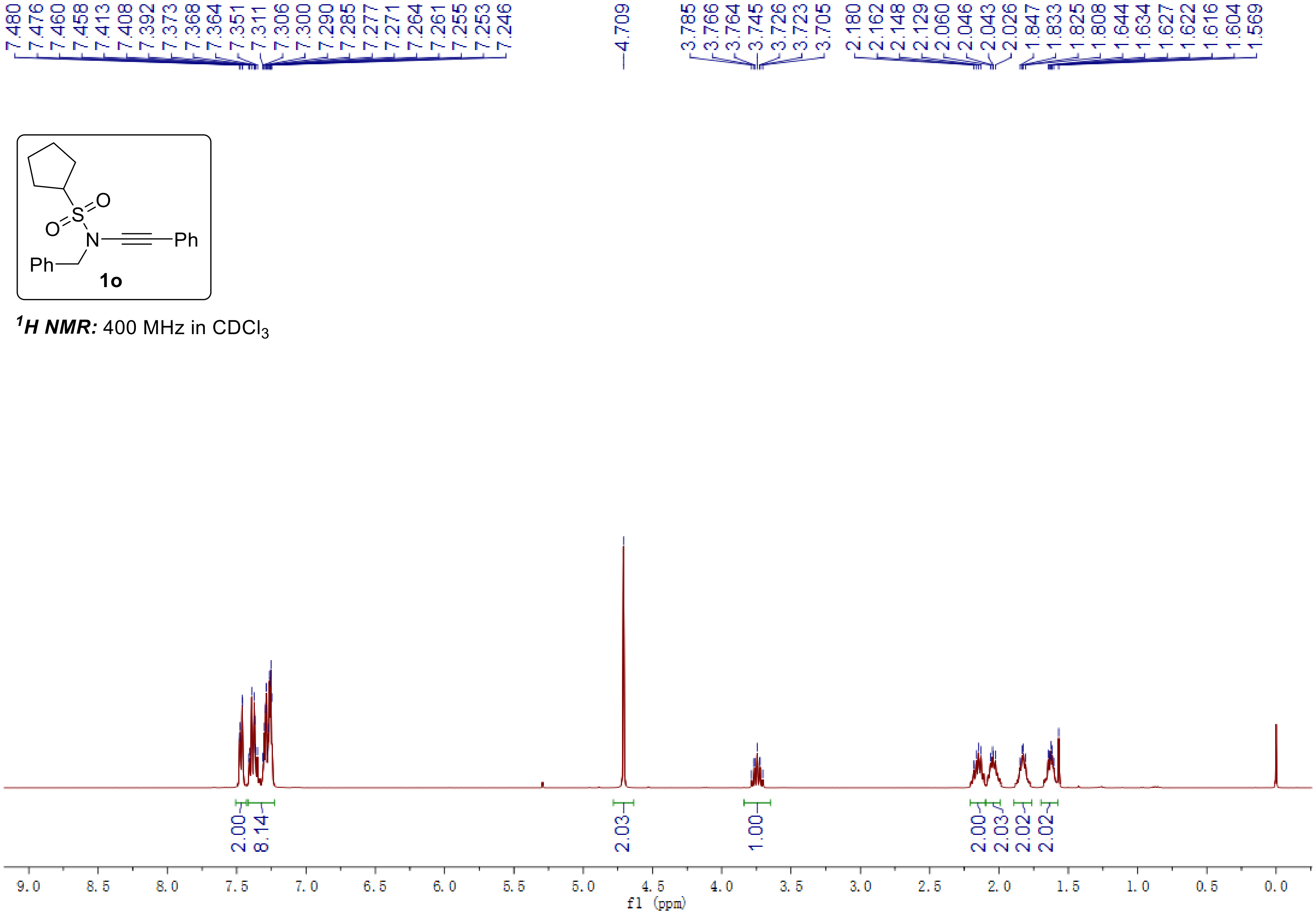

^{1}H NMR: 400 MHz in $CDCl_3$

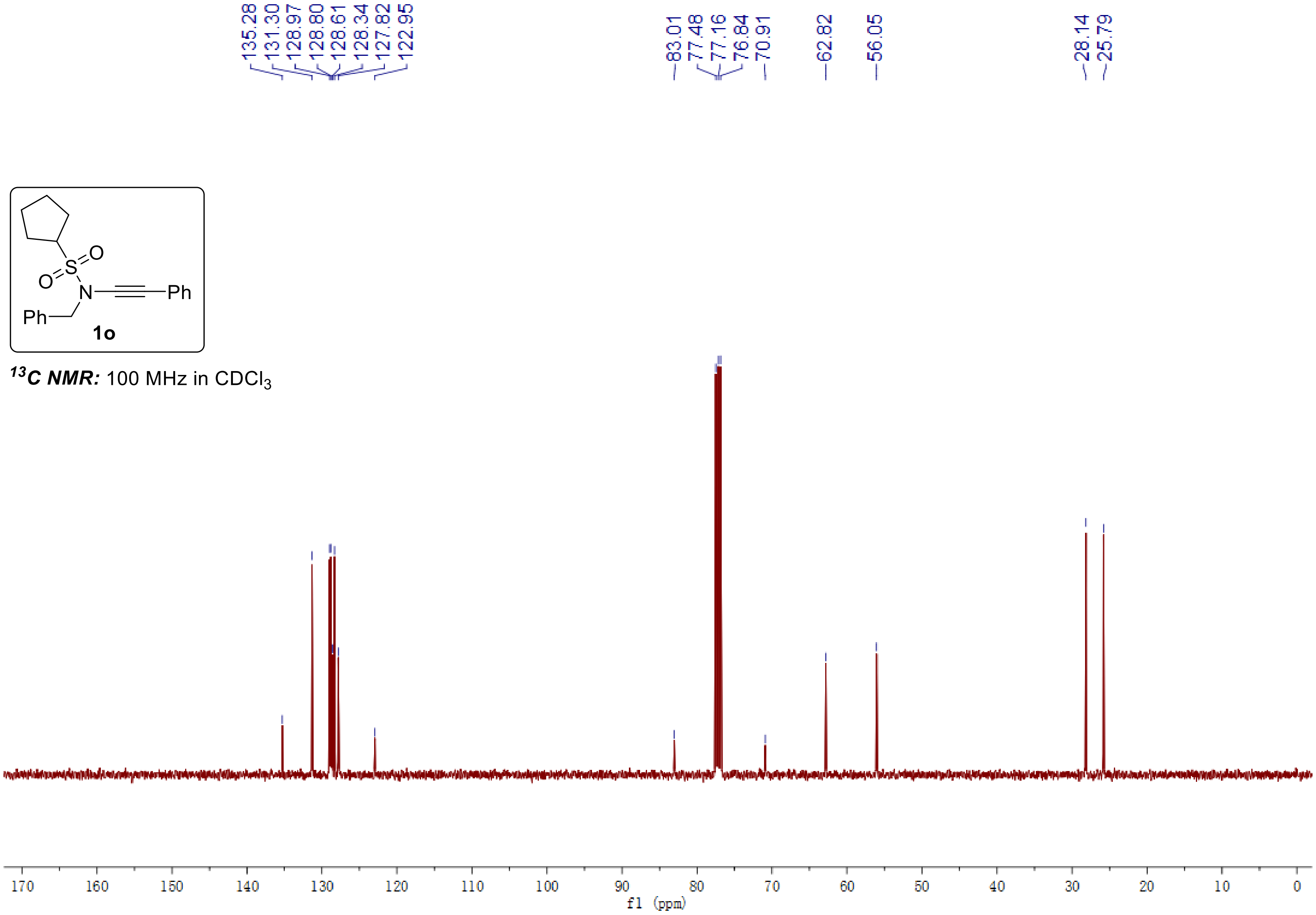

1o

13C NMR: 100 MHz in $CDCl_3$

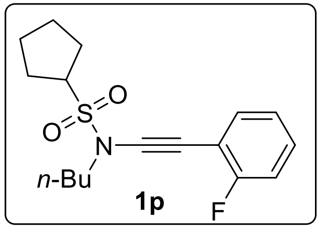

^{1}H NMR: 500 MHz in $CDCl_3$

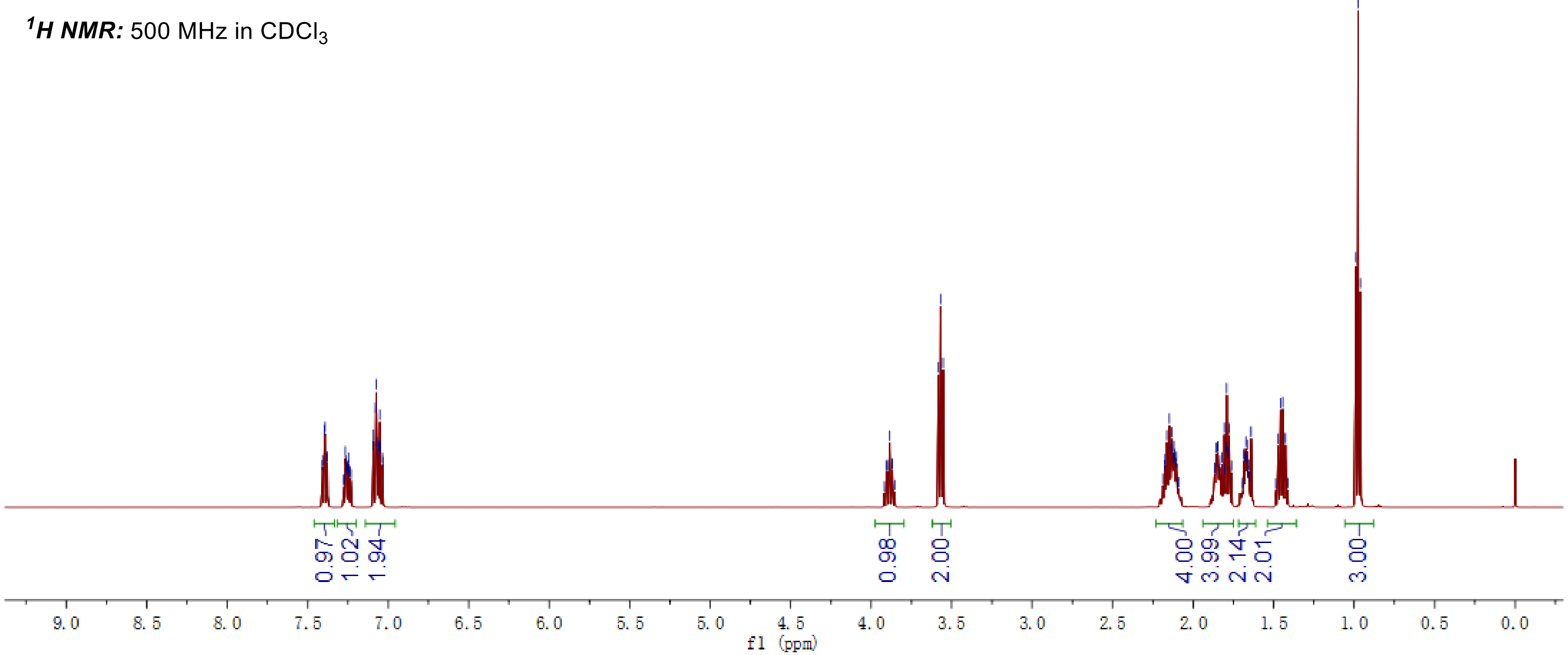

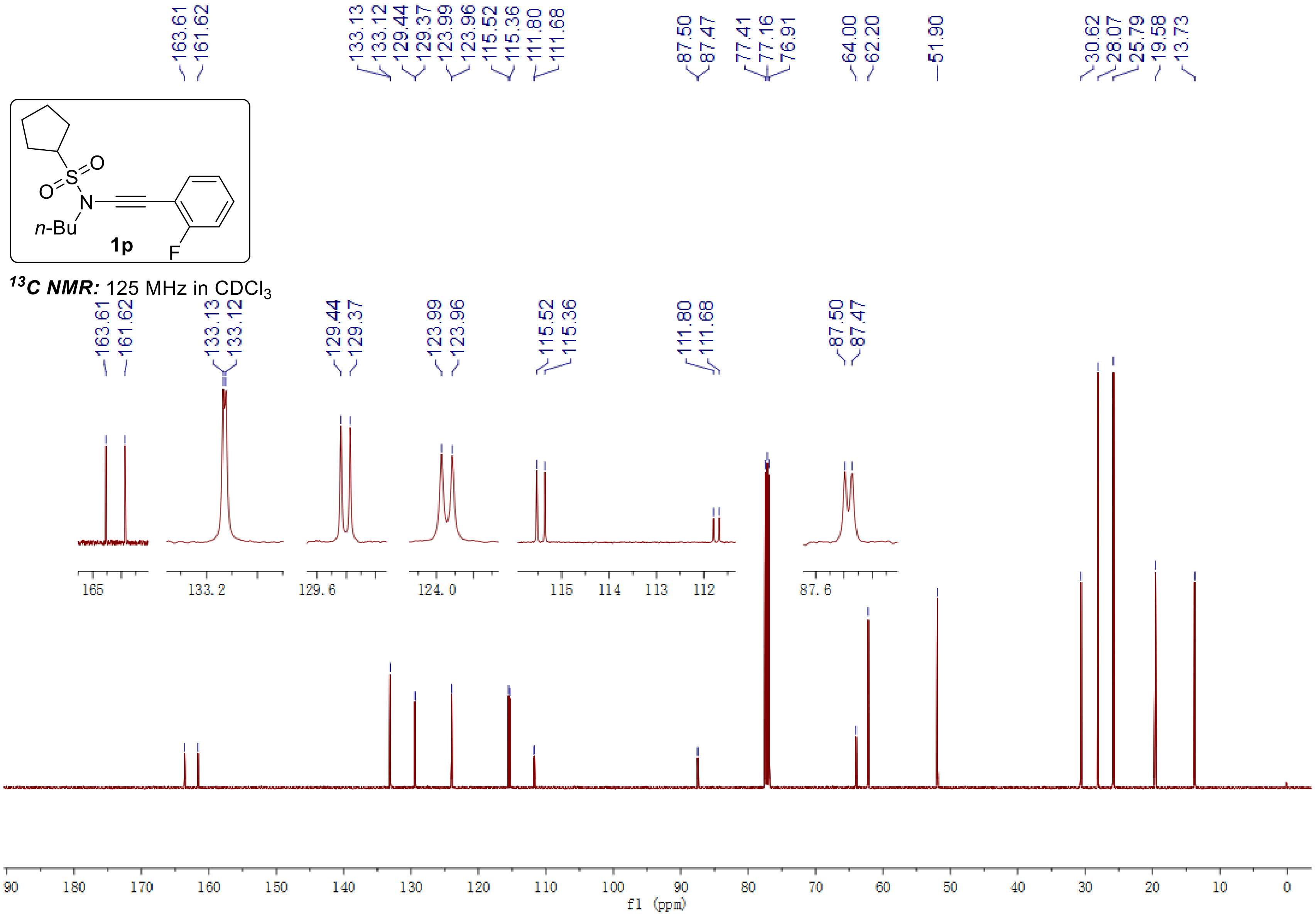

¹³C NMR: 125 MHz in $CDCl_3$

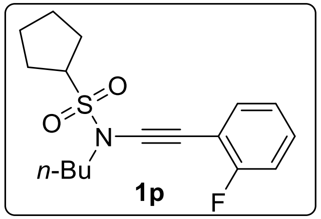

^{19}***F NMR:*** 376 MHz in $CDCl_3$

-111.102

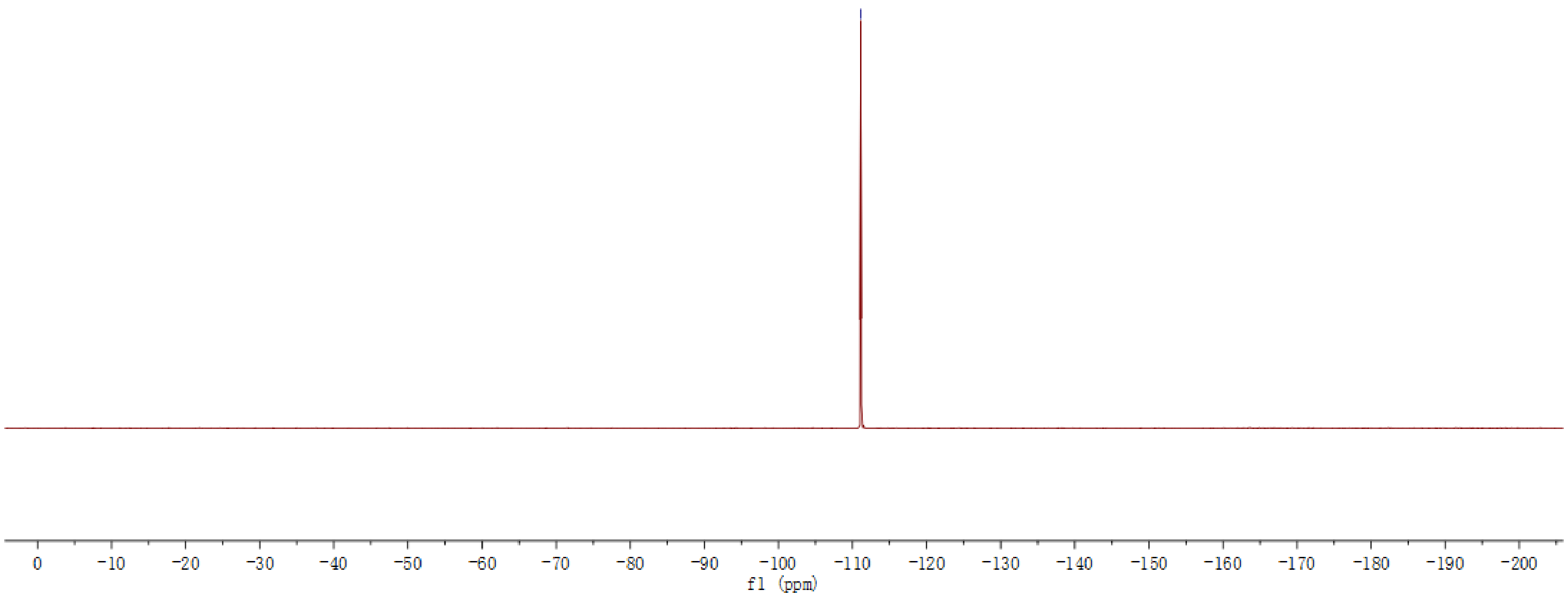

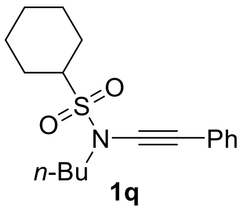

1q

^{1}H NMR: 400 MHz in $CDCl_3$

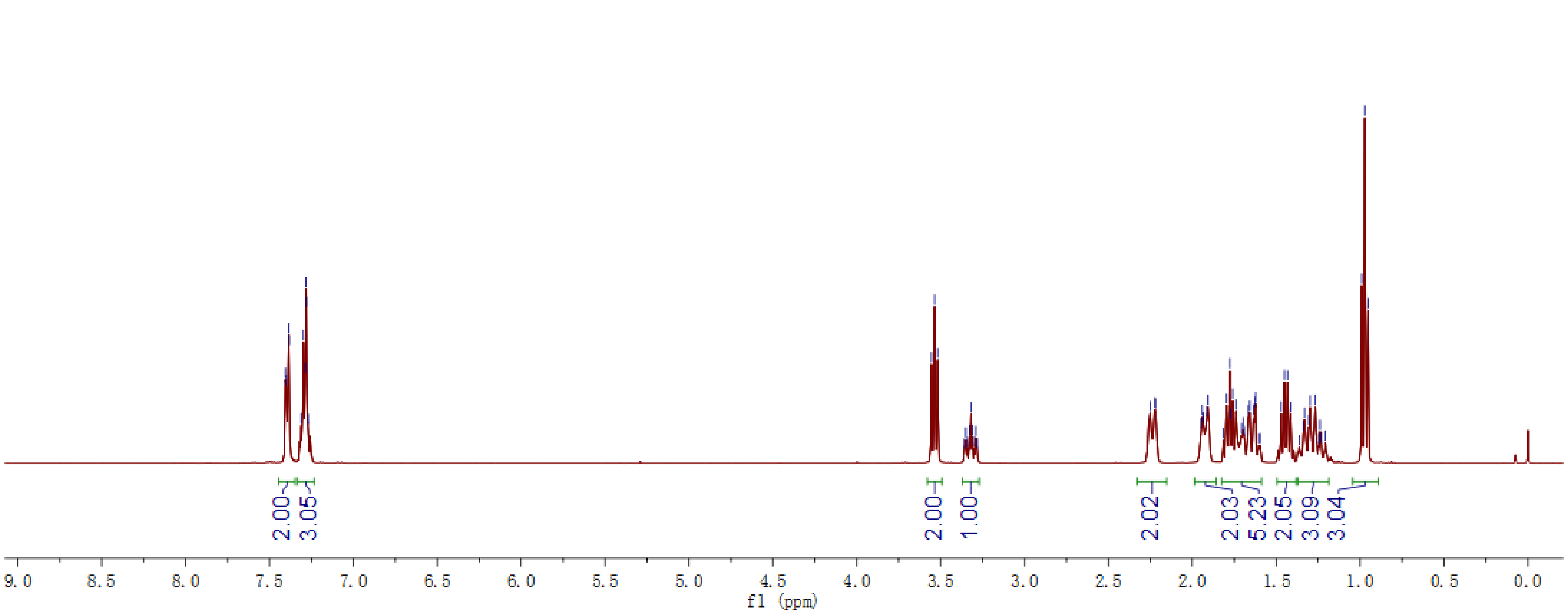

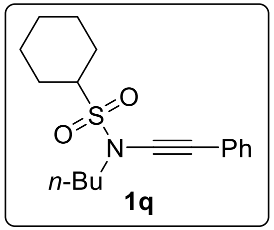

^{13}**C NMR:** 100 MHz in $CDCl_3$

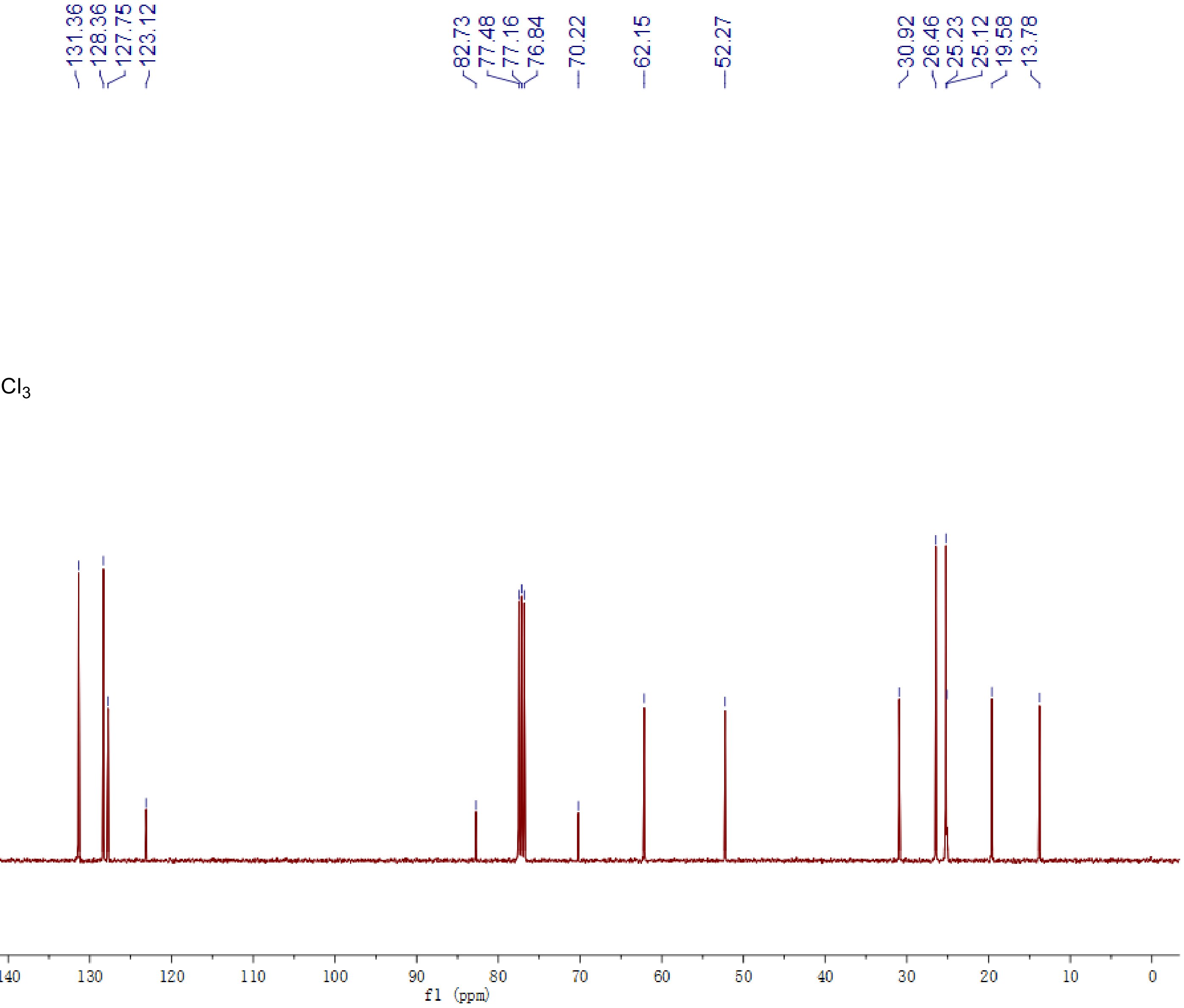

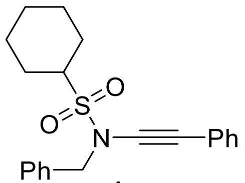

1r

^{1}H NMR: 400 MHz in $CDCl_3$

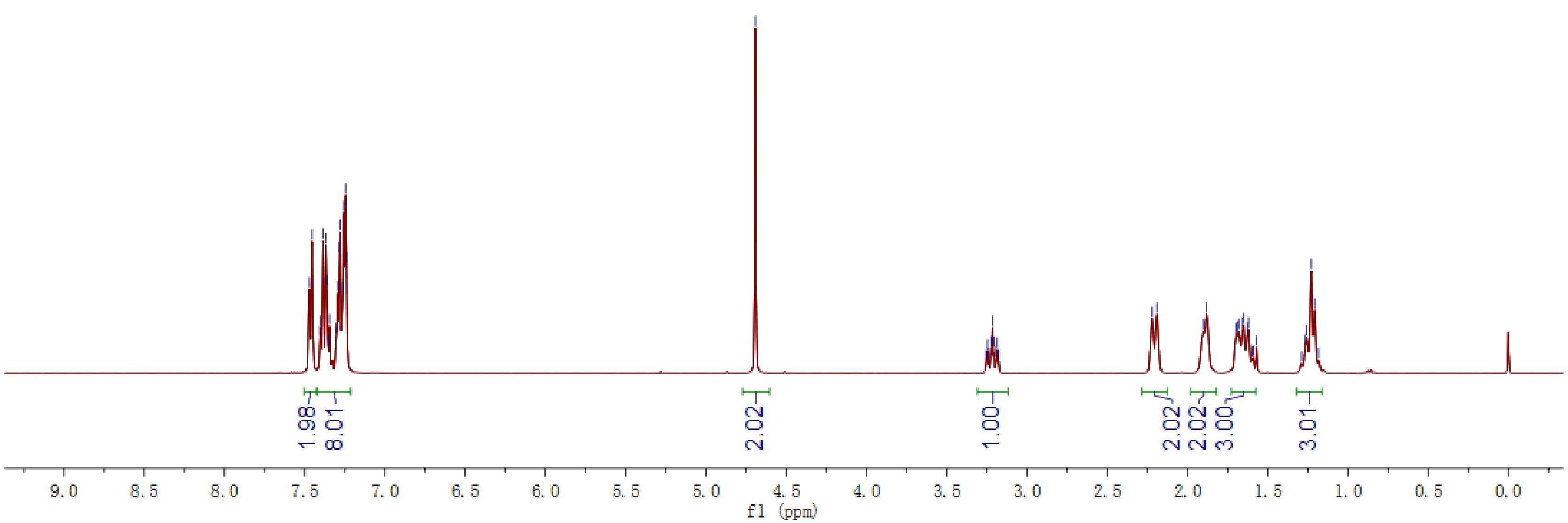

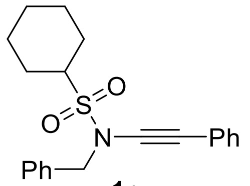

1r

^{13}C NMR: 100 MHz in $CDCl_3$

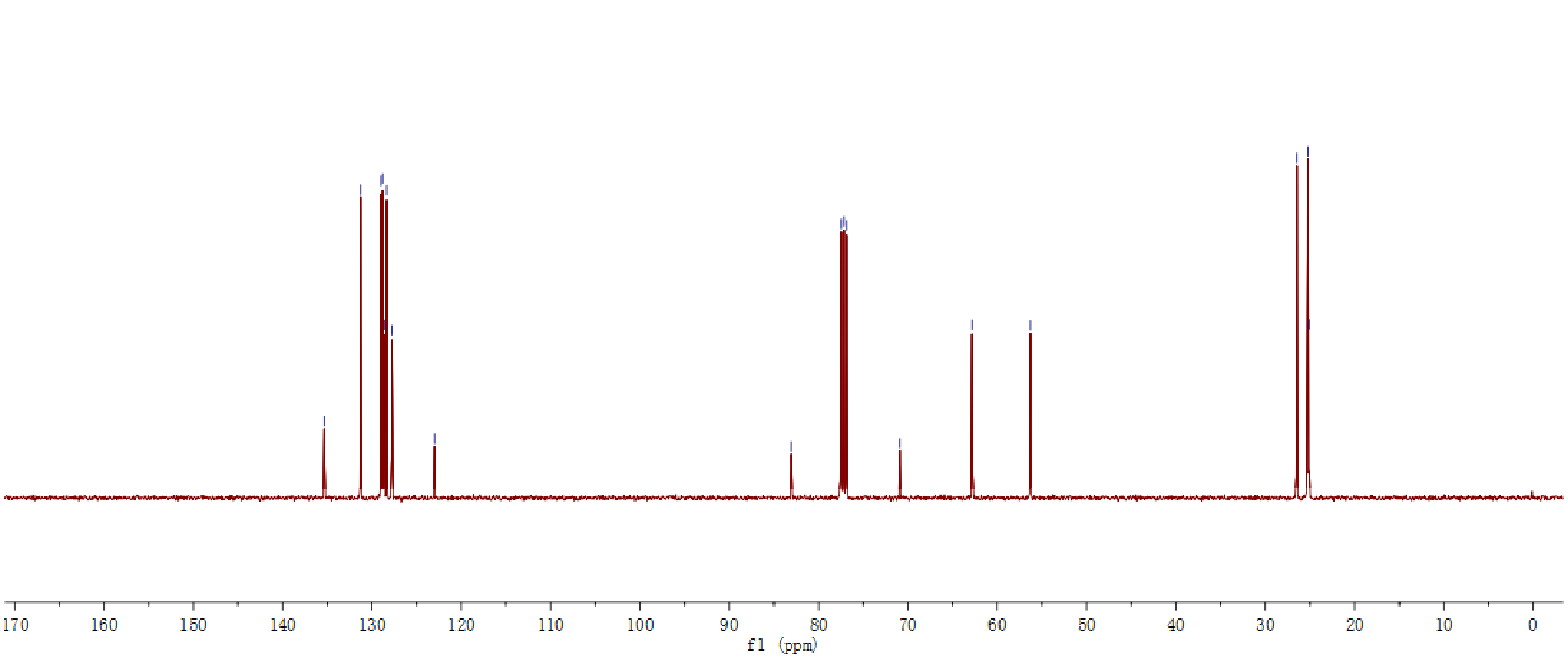

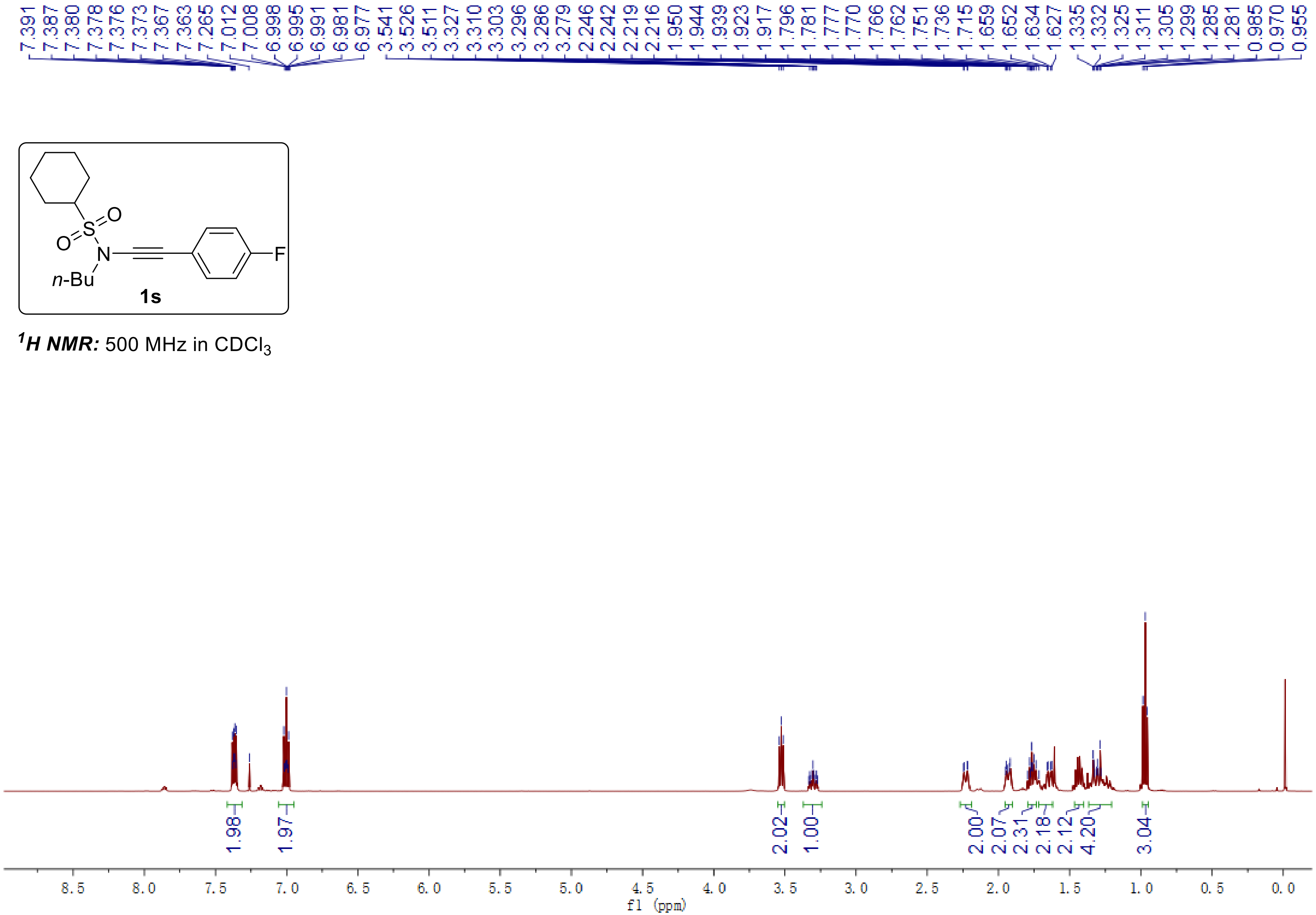

^{1}H NMR: 500 MHz in $CDCl_3$

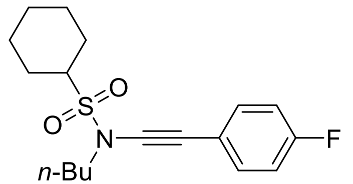

^{13}C NMR: 125 MHz in $CDCl_3$

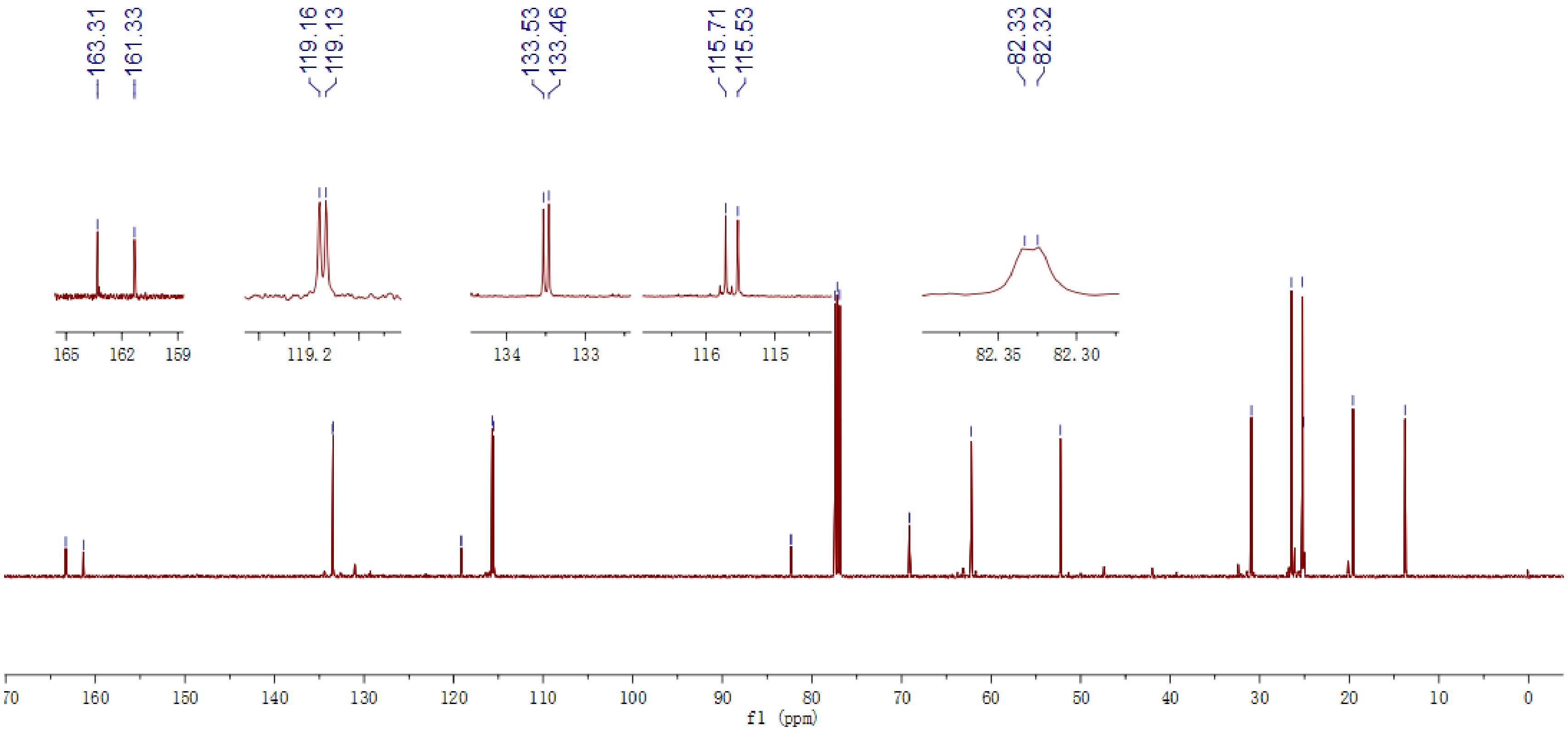

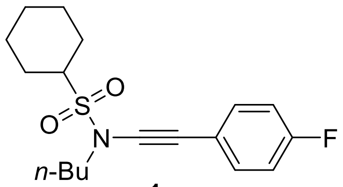

1s

^{19}F NMR: 376 MHz in $CDCl_3$

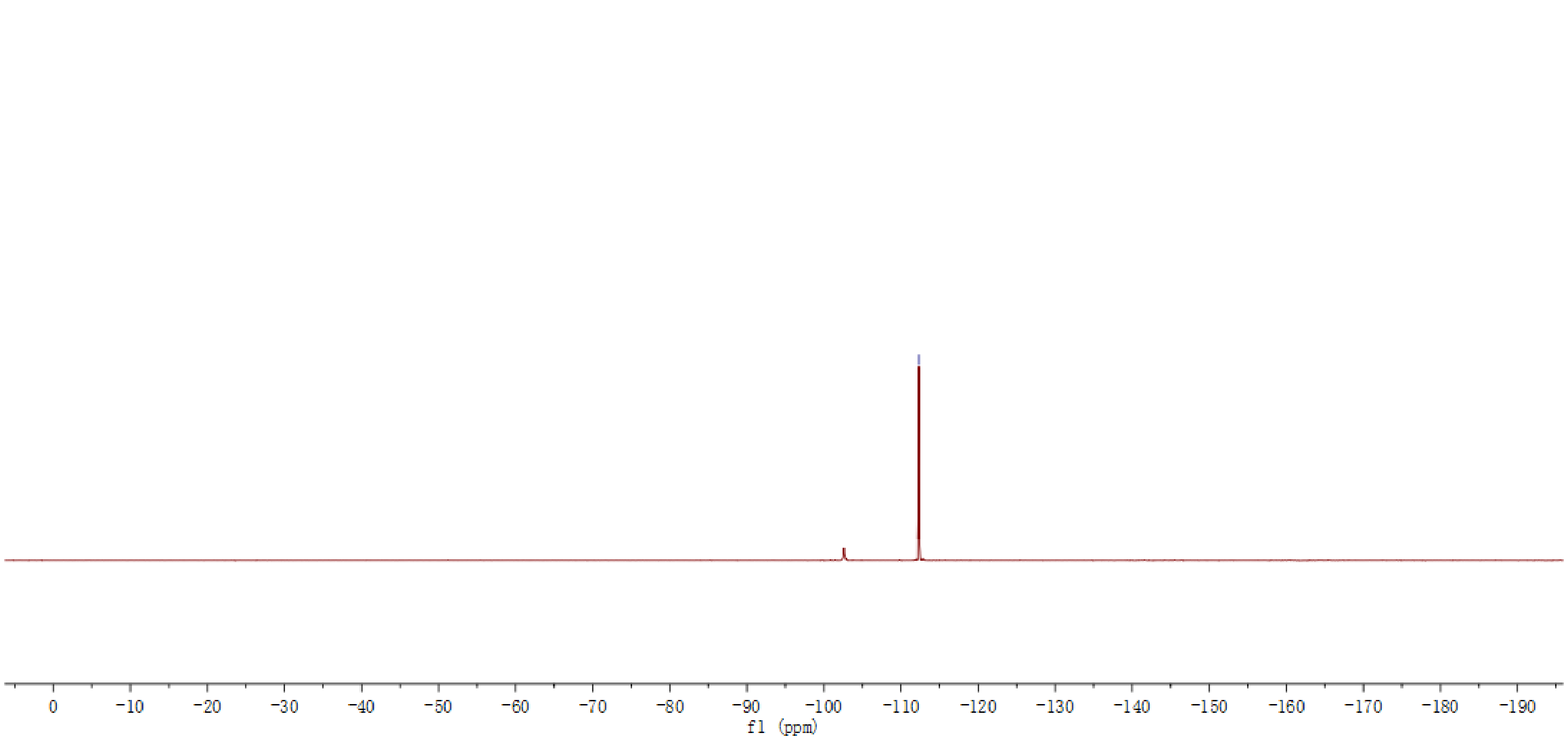

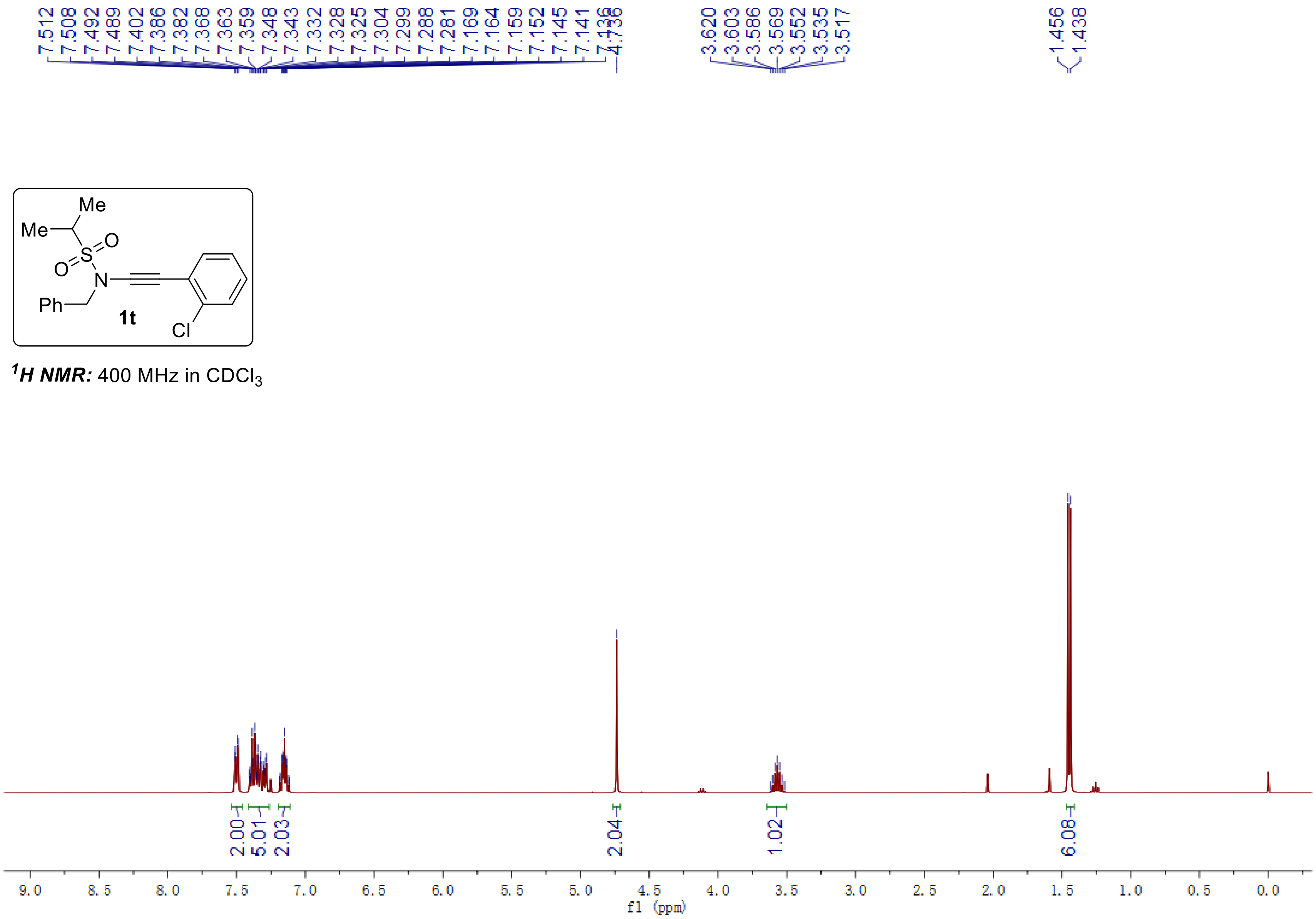

^{1}H NMR: 400 MHz in $CDCl_3$

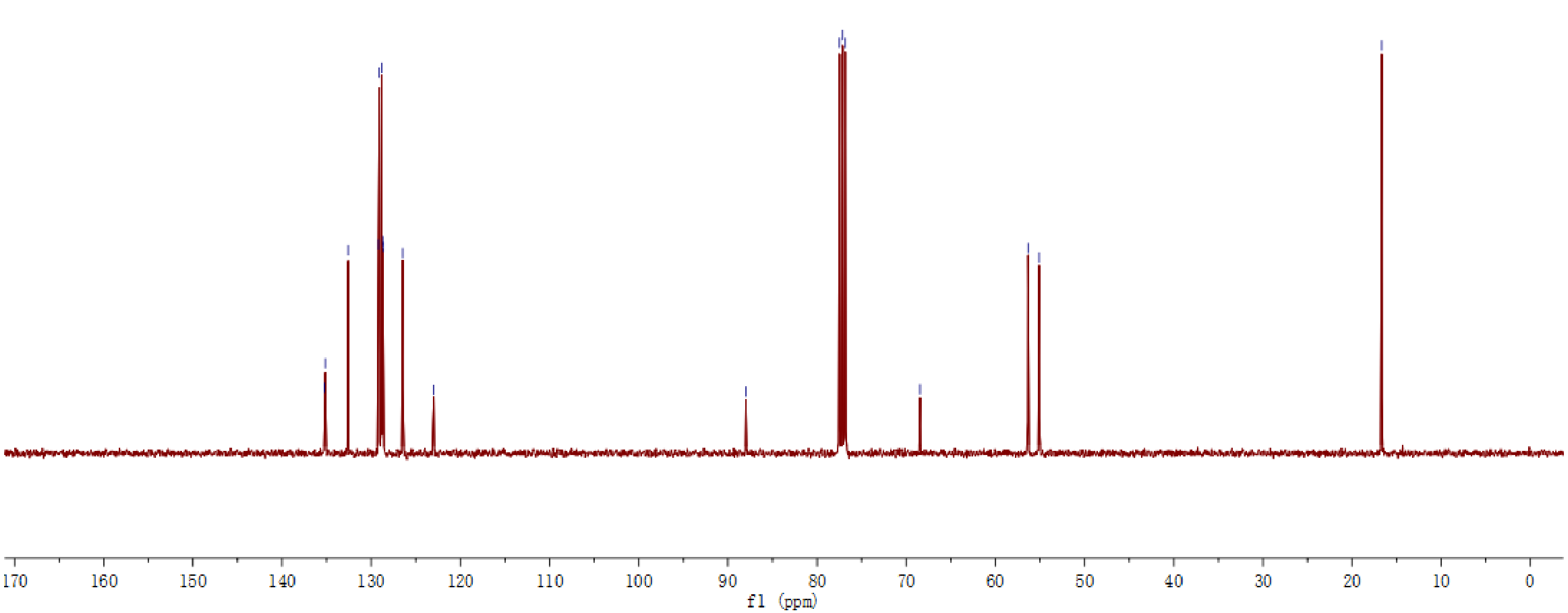

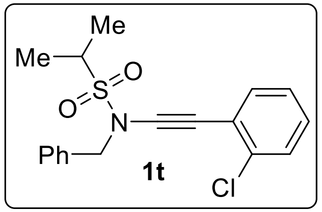

1t

^{13}C NMR: 100 MHz in $CDCl_3$

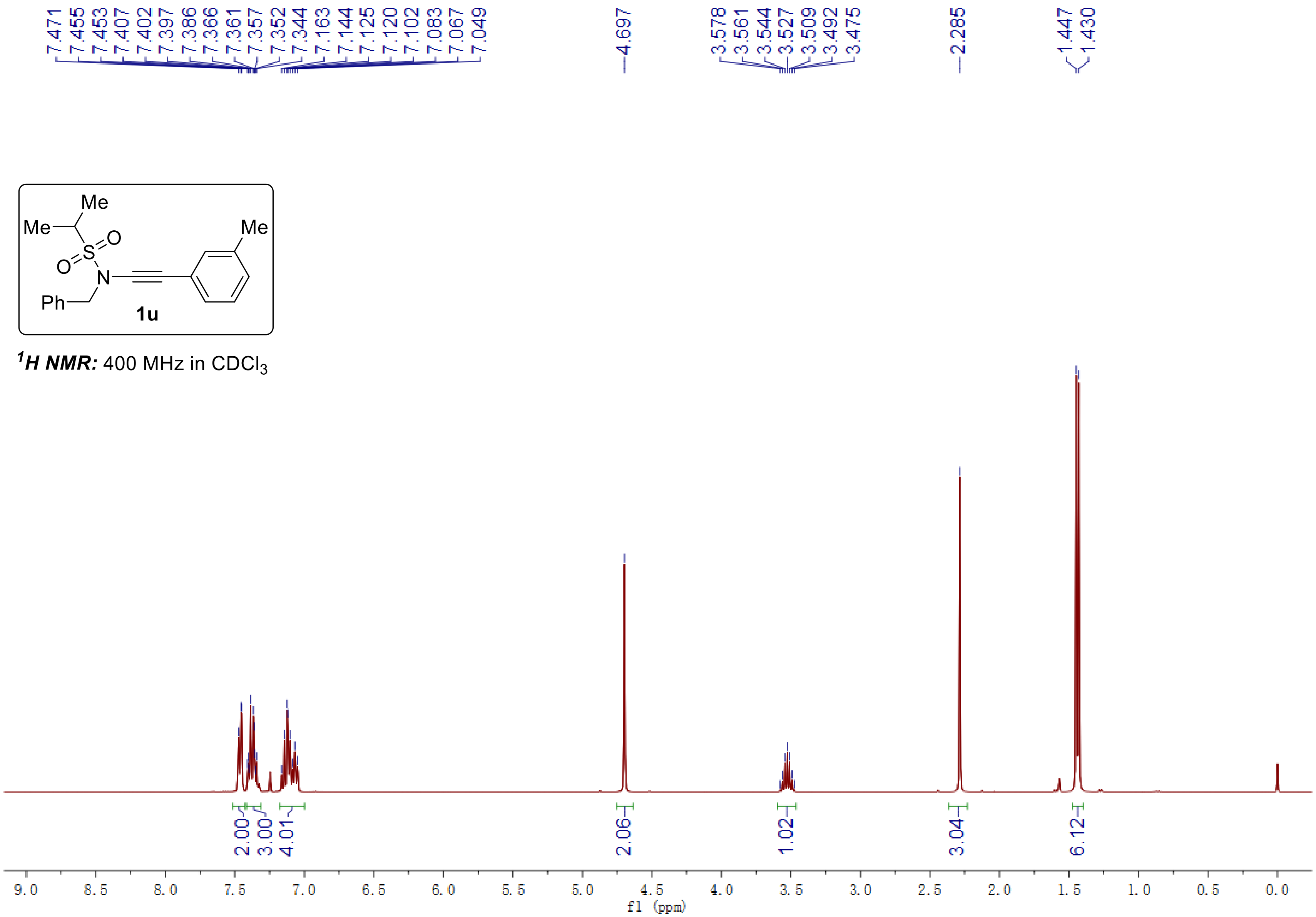
7.471 7.455 7.453 7.407 7.402 7.397 7.386 7.366 7.361 7.357 7.352 7.344 7.163 7.144 7.125 7.120 7.102 7.083 7.067 7.049
4.697
3.578 3.561 3.544 3.527 3.509 3.492 3.475
2.285
1.447 1.430
Me
Me
Me
O
S
O
N
Ph
1u
¹H NMR: 400 MHz in CDCl₃
2.00
3.00
4.01
2.06
1.02
3.04
6.12
9.0 8.5 8.0 7.5 7.0 6.5 6.0 5.5 5.0 4.5 4.0 3.5 3.0 2.5 2.0 1.5 1.0 0.5 0.0
f1 (ppm)

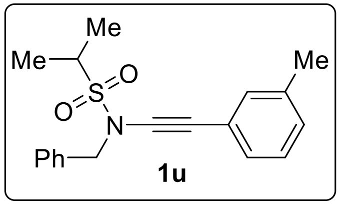

1u

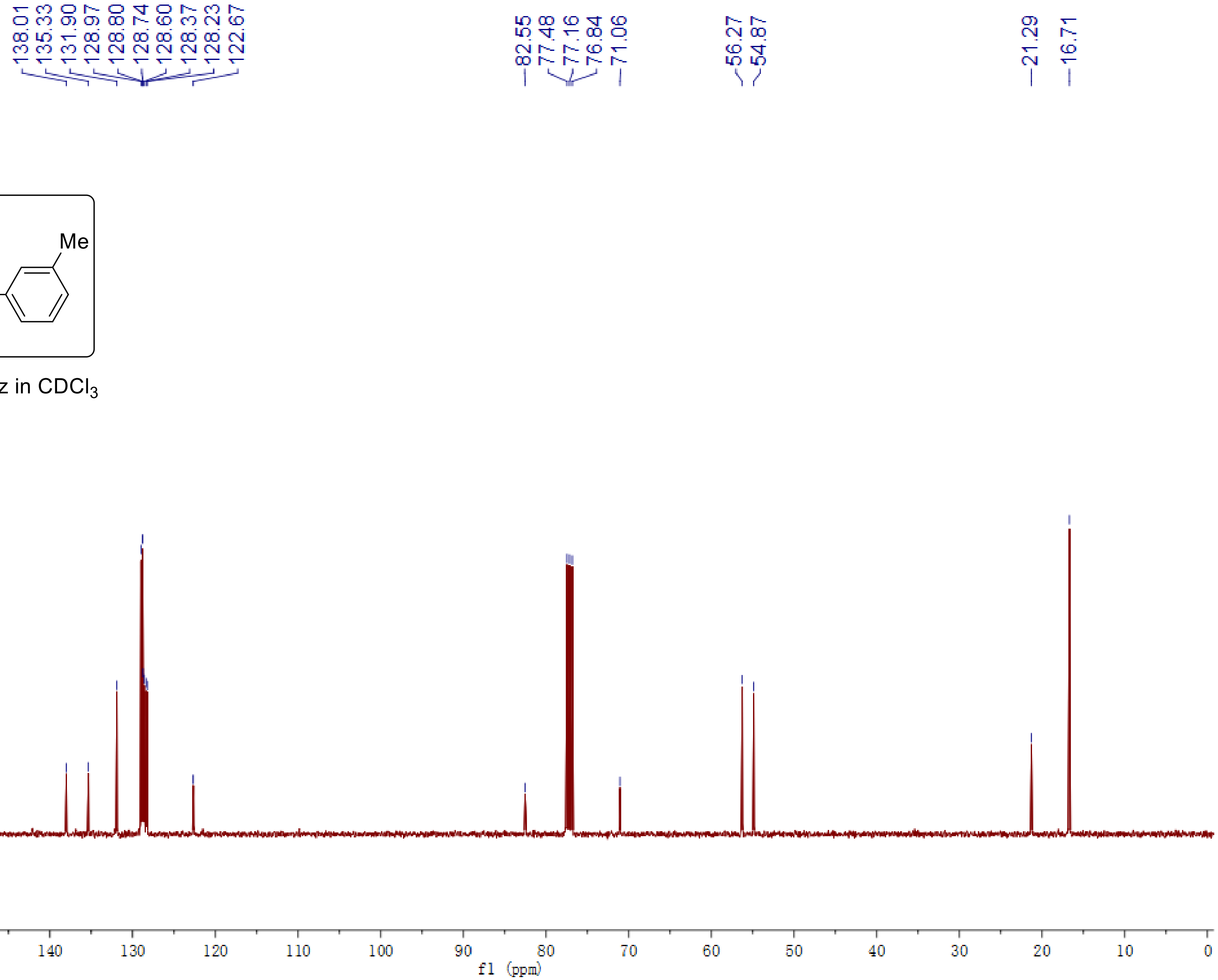

^{13}C NMR: 100 MHz in $CDCl_3$

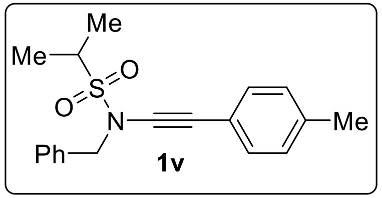

1v

¹H NMR: 400 MHz in $CDCl_3$

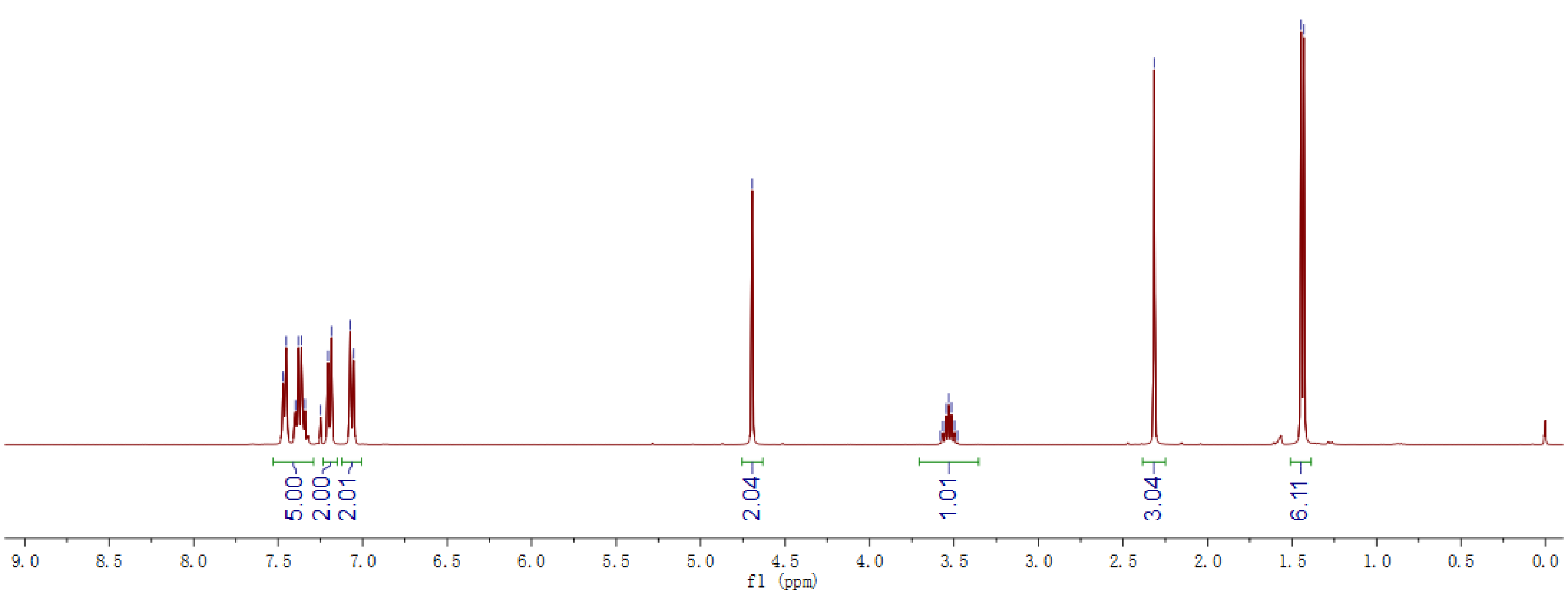

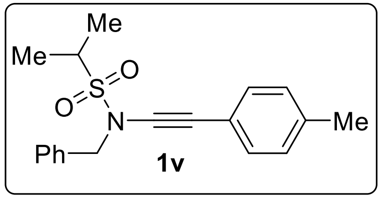

^{13}**C NMR:** 100 MHz in $CDCl_3$

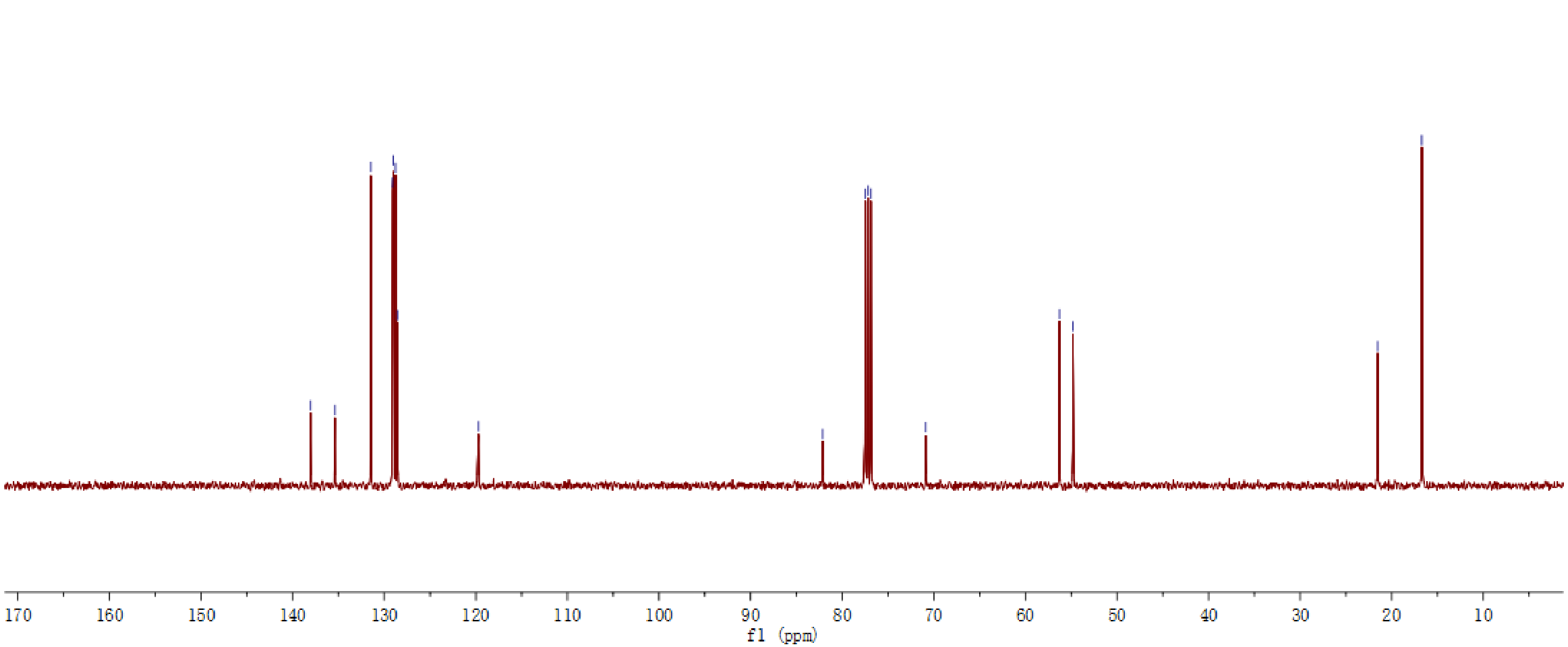

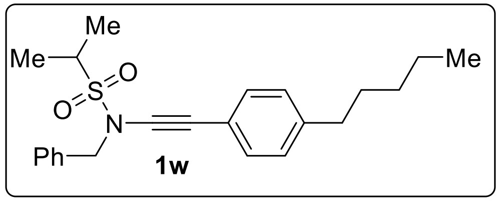

1w

^{1}H NMR: 400 MHz in $CDCl_3$

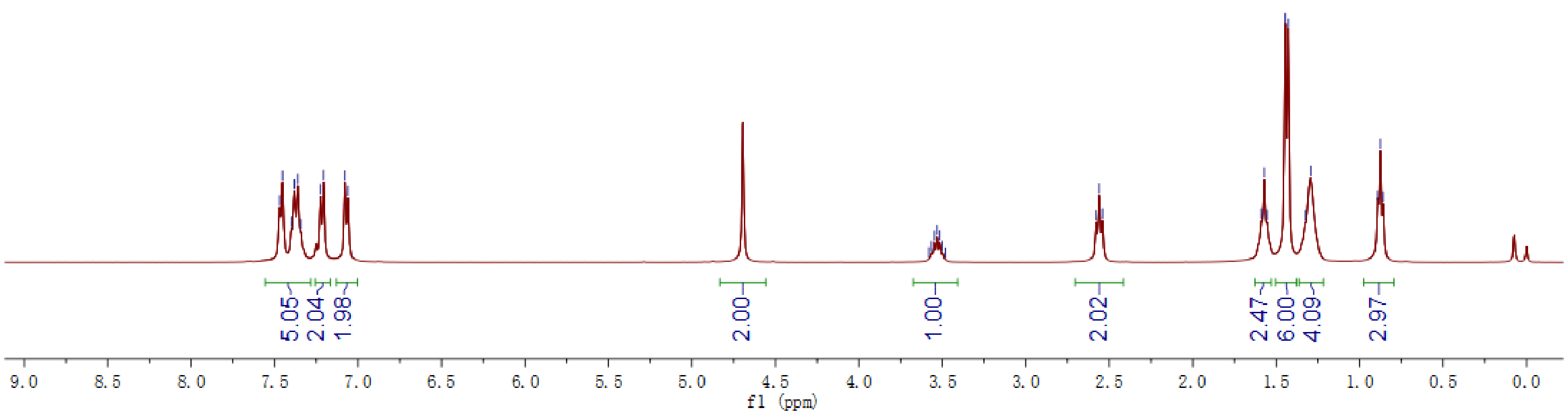

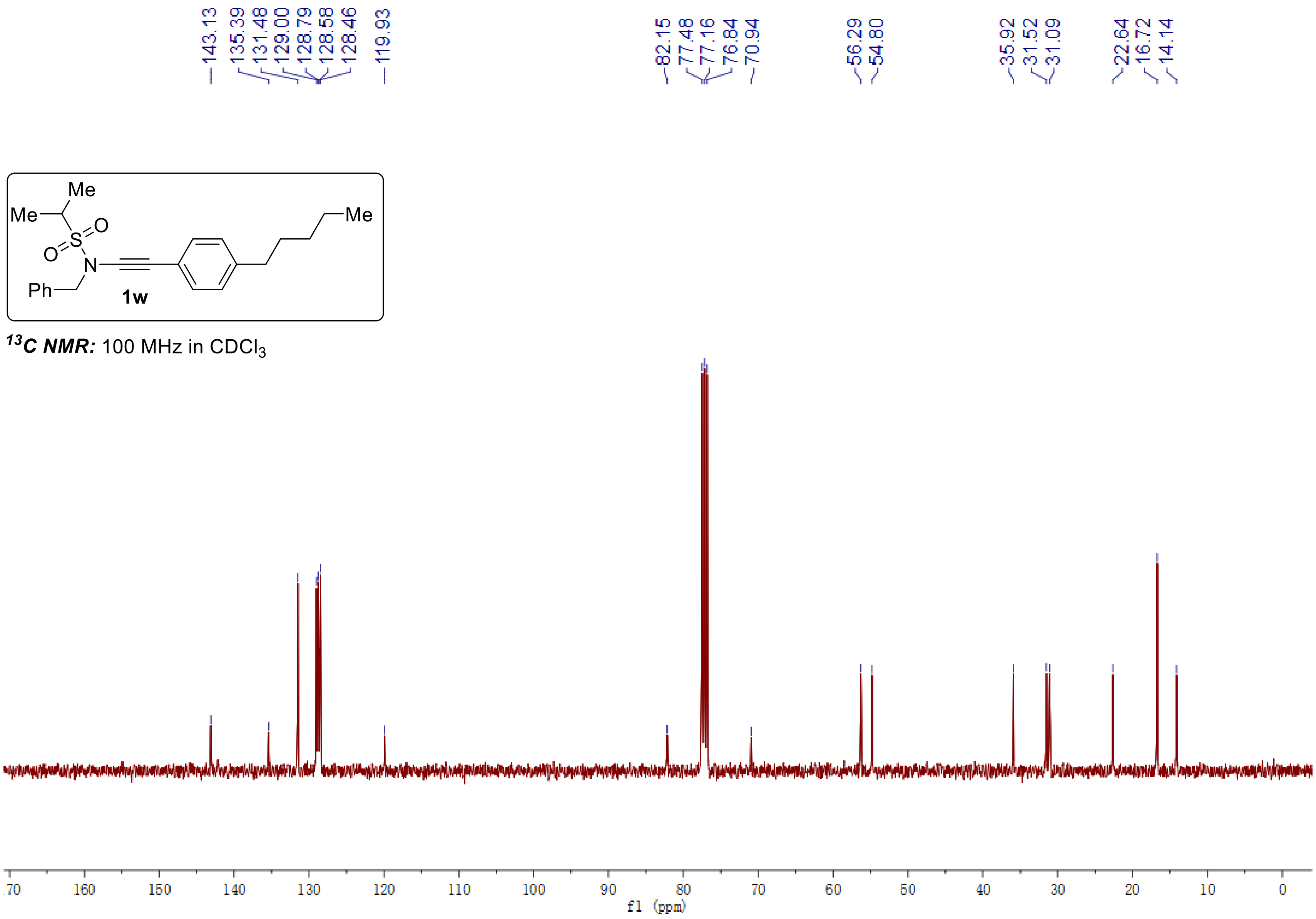
143.13
135.39
131.48
129.00
128.79
128.58
128.46
119.93
82.15
77.48
77.16
76.84
70.94
56.29
54.80
35.92
31.52
31.09
22.64
16.72
14.14
Me
Me
O
S
O
N
Ph
1w
Me
13C NMR: 100 MHz in CDCl3
170
160
150
140
130
120
110
100
90
80
70
60
50
40
30
20
10
0
f1 (ppm)

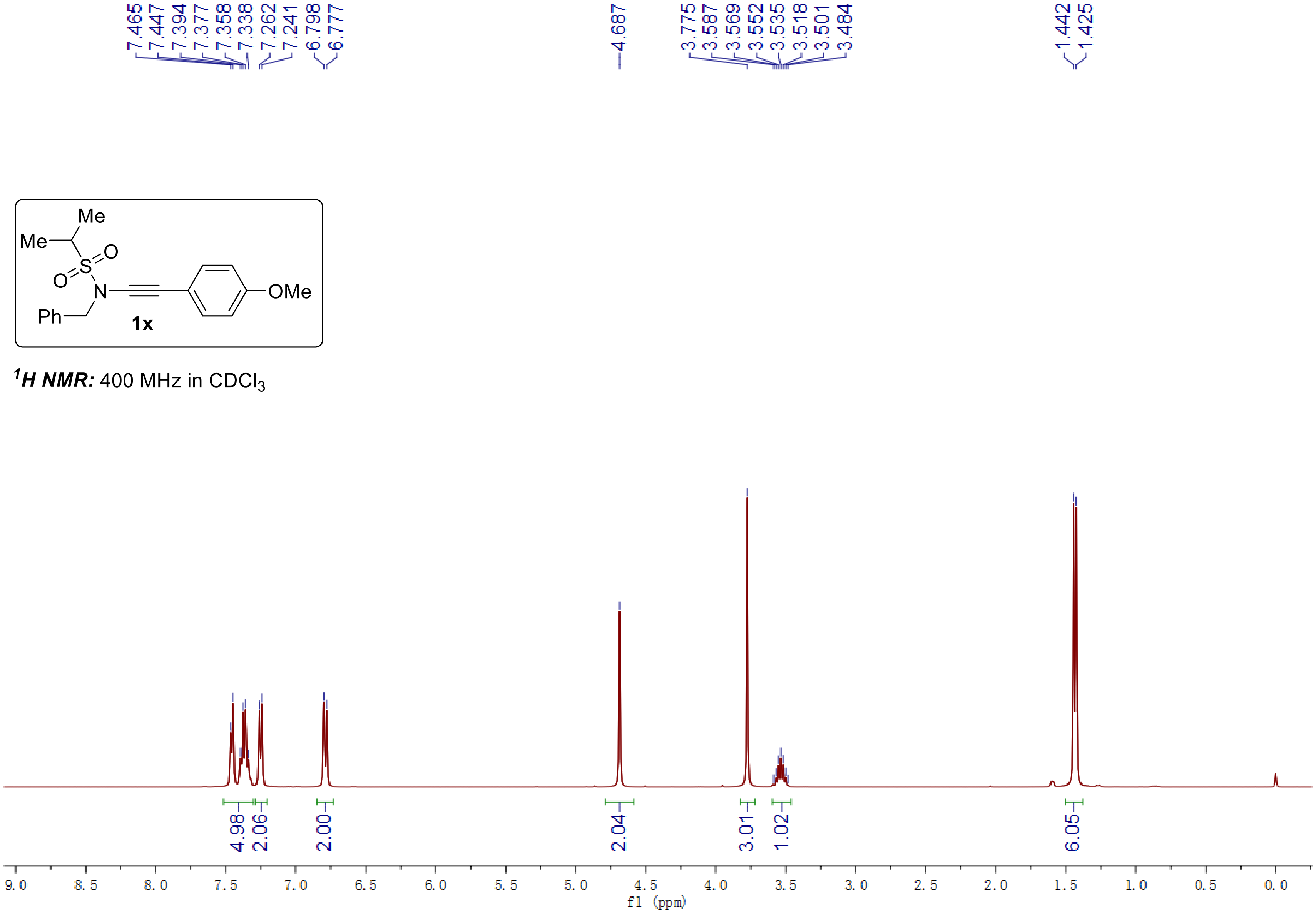

^{1}***H NMR:*** 400 MHz in $CDCl_3$

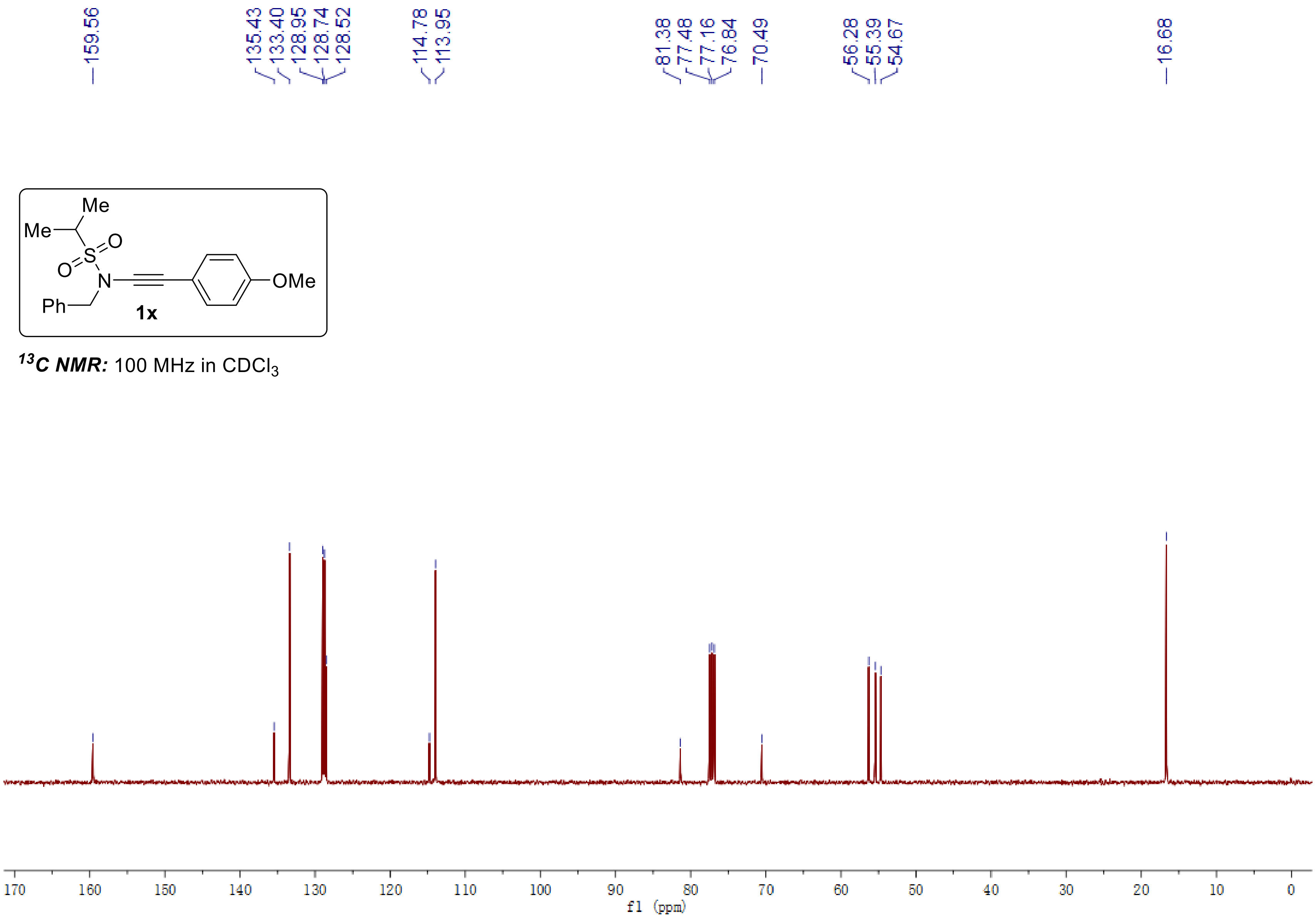

^{13}C NMR: 100 MHz in $CDCl_3$

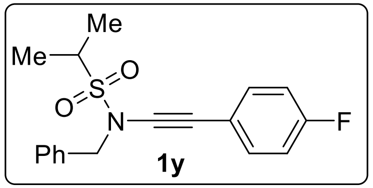

[1]*H NMR:* 400 MHz in $CDCl_3$

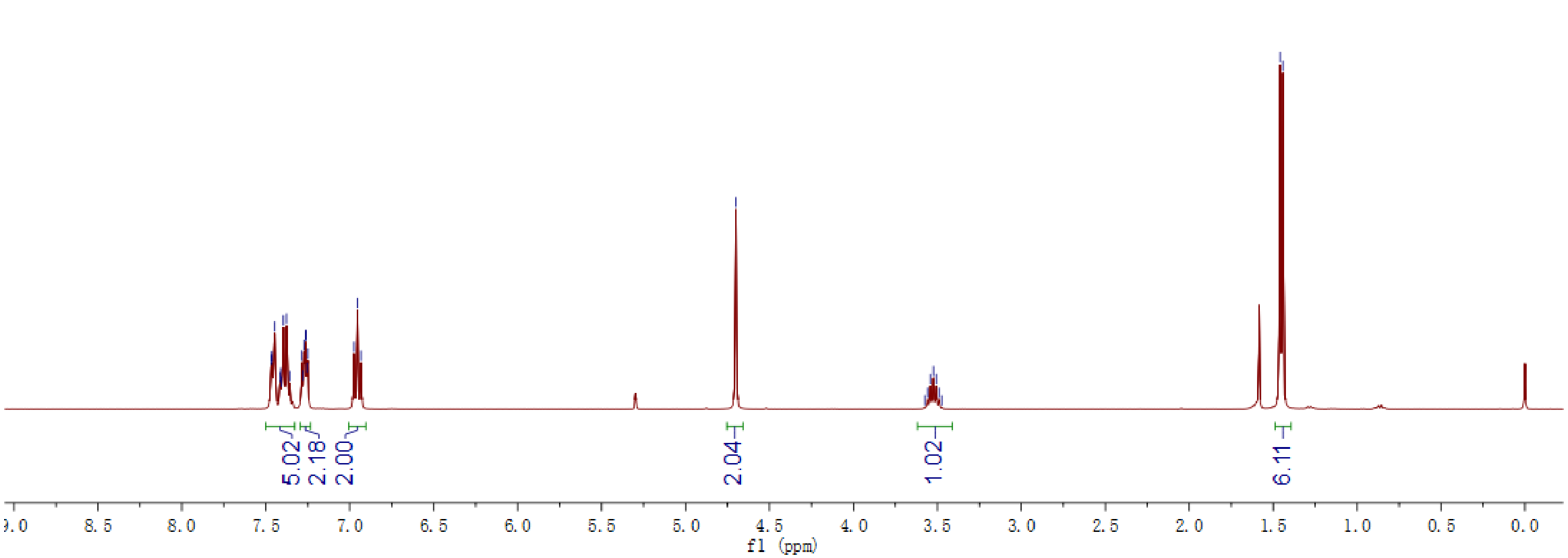

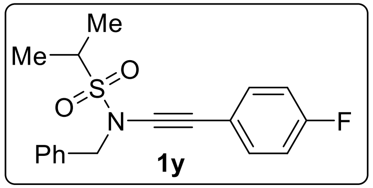

1y

^{13}**C NMR:** 100 MHz in $CDCl_3$

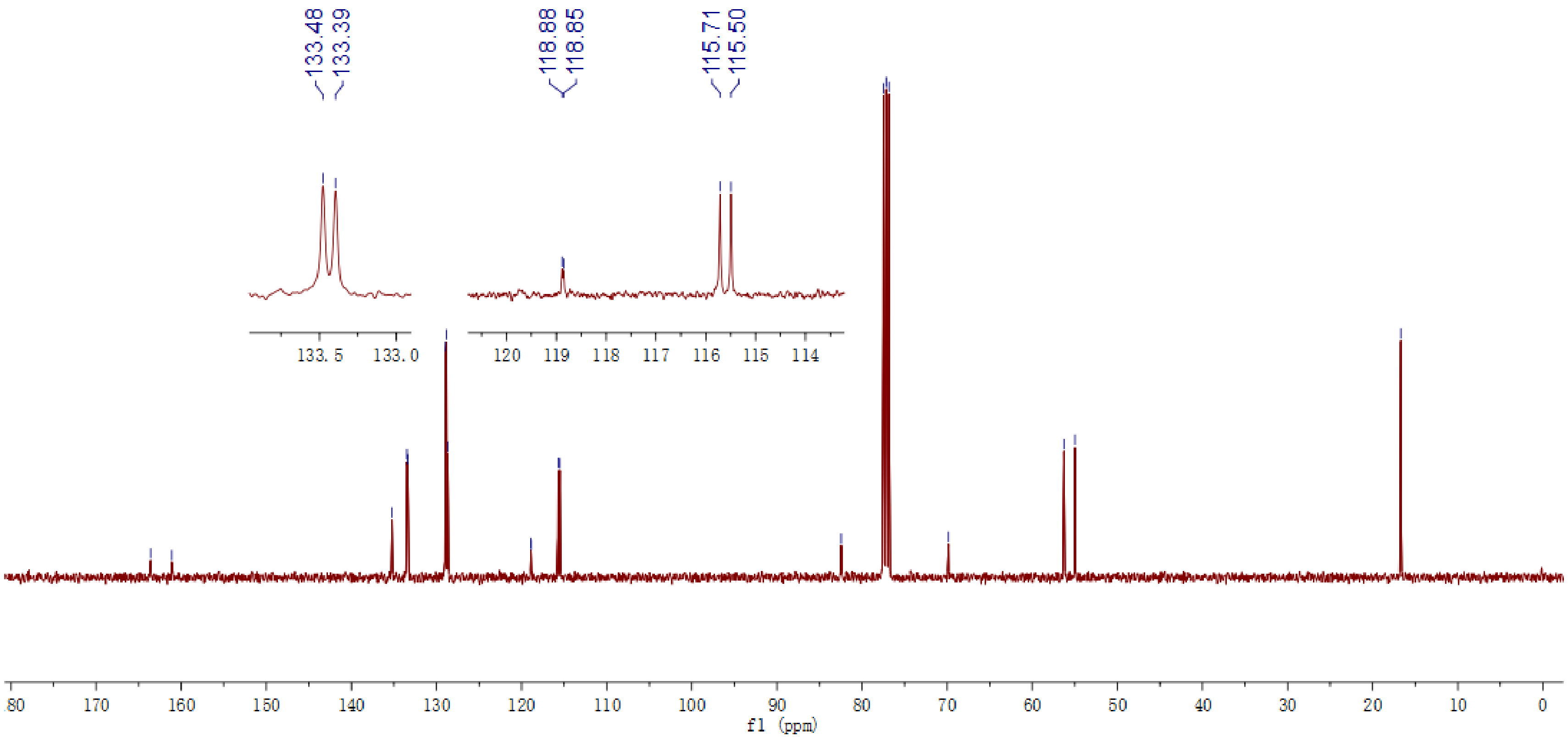

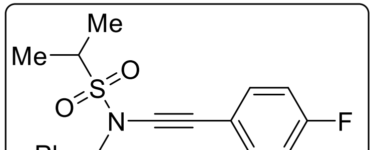

1y

^{19}F NMR: 376 MHz in $CDCl_3$

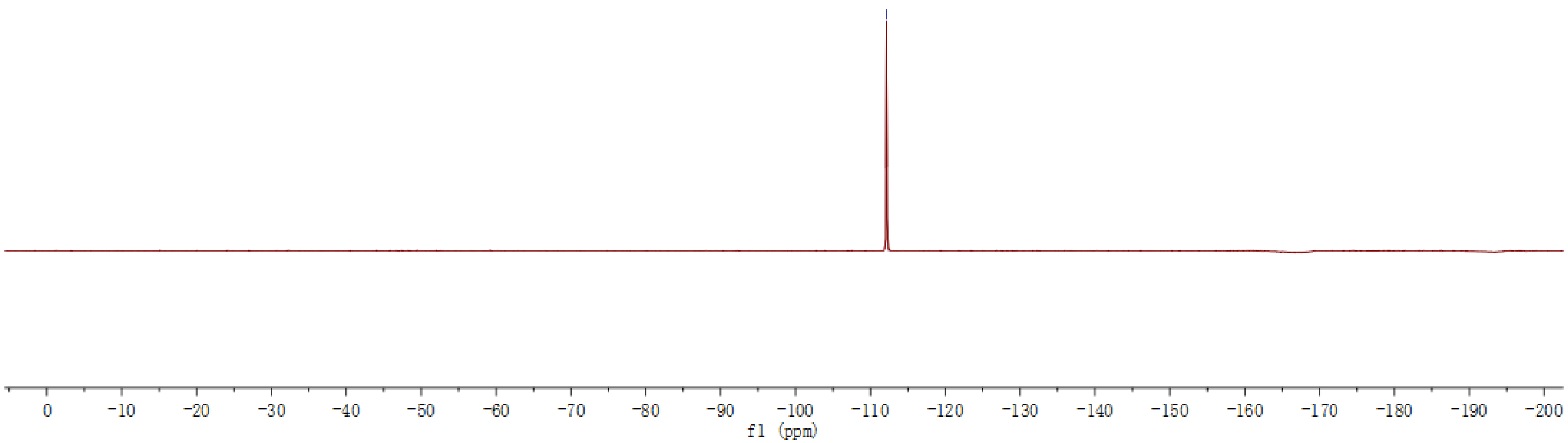

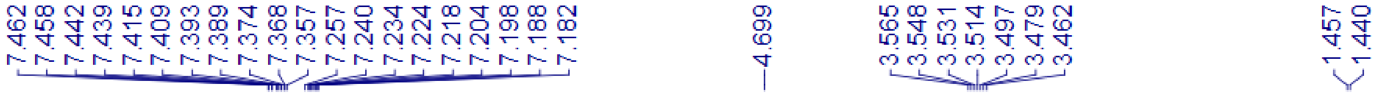

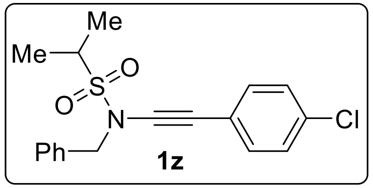

^{1}**_H NMR:_** 400 MHz in $CDCl_3$

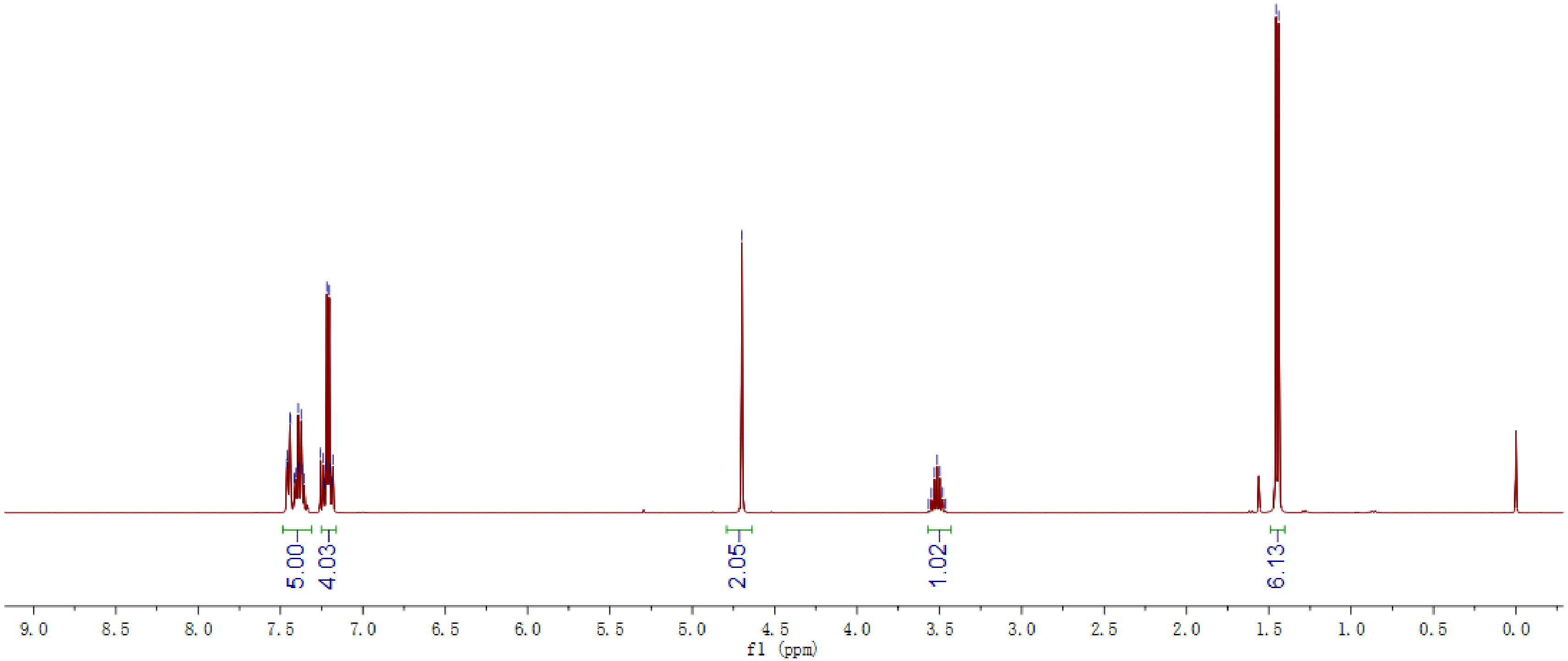

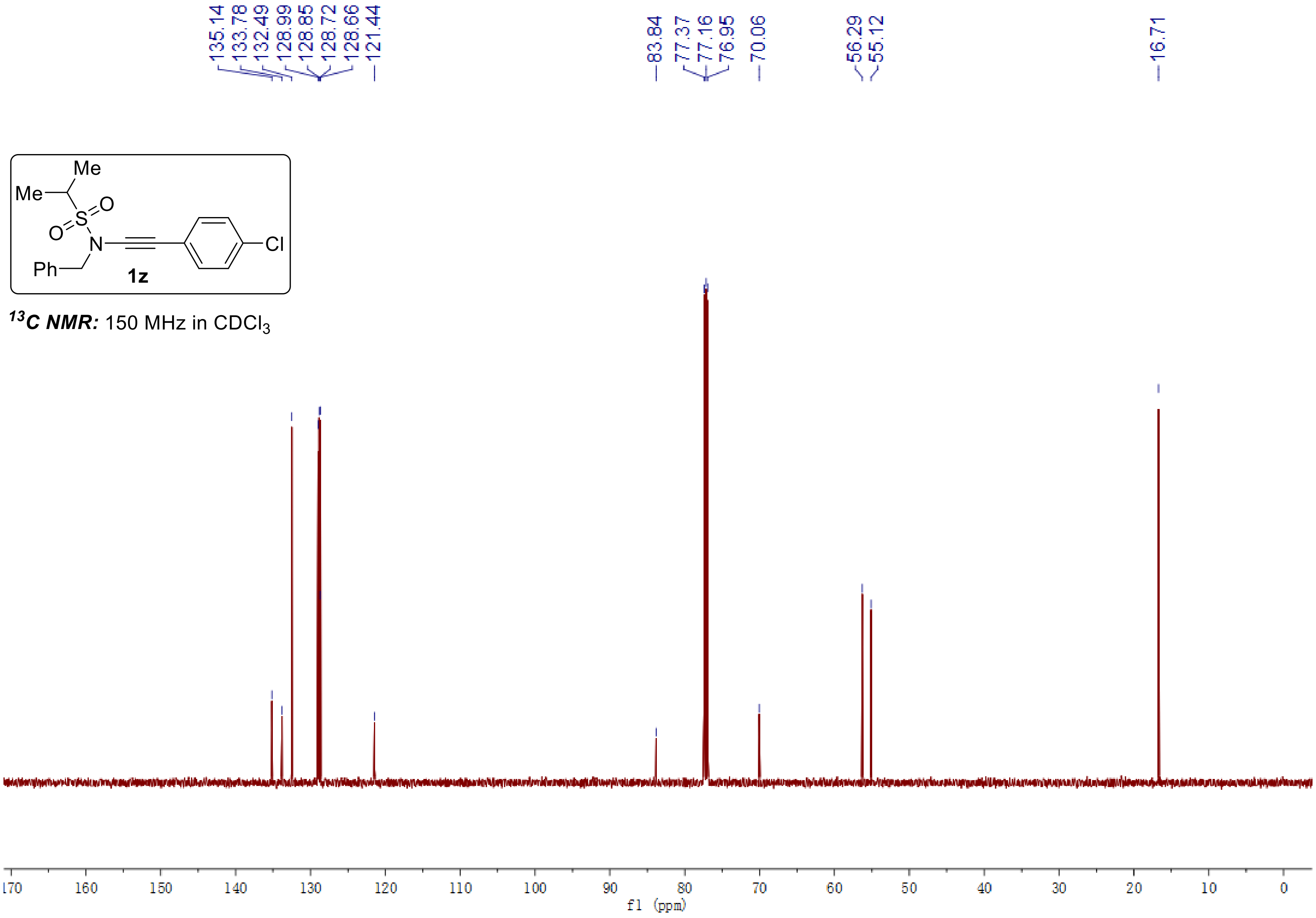

1z

^{13}C NMR: 150 MHz in $CDCl_3$

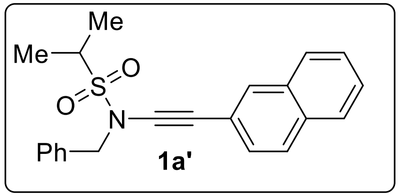

^{1}H NMR: 600 MHz in $CDCl_3$

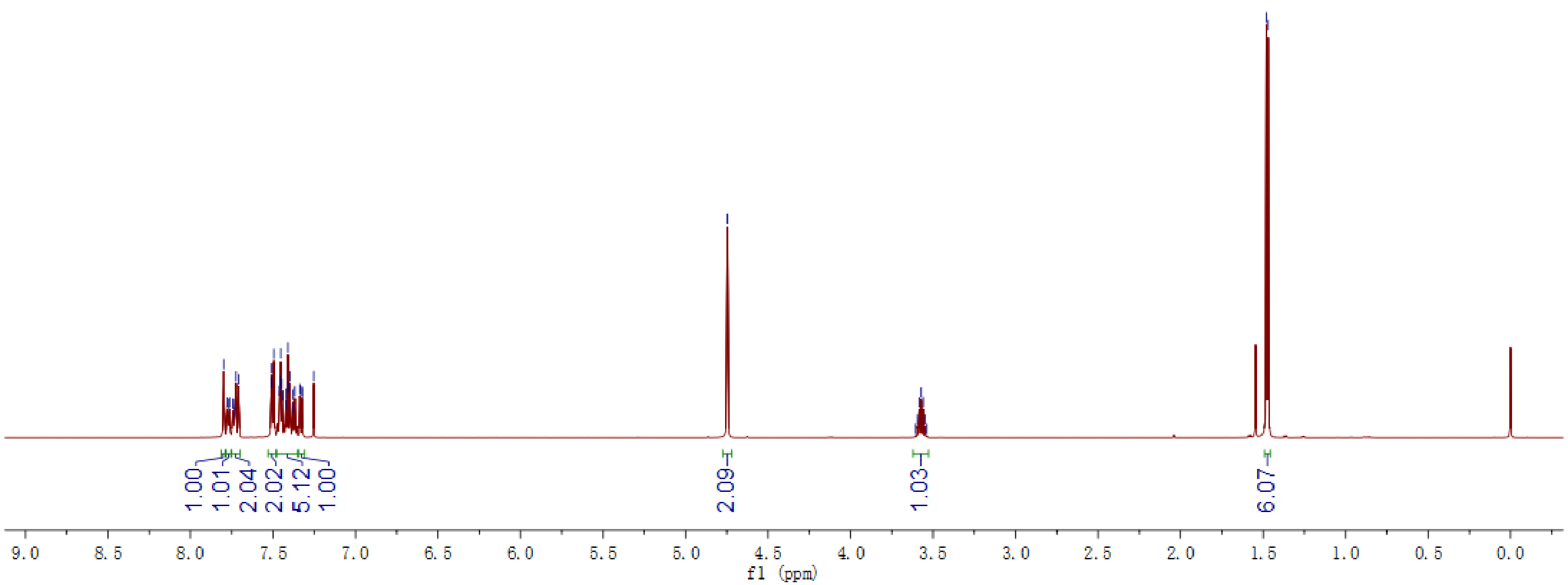

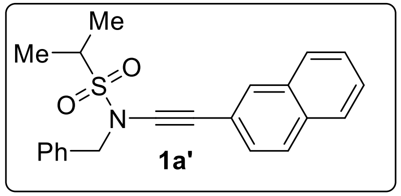

1a'

***13*C NMR:** 150 MHz in $CDCl_3$

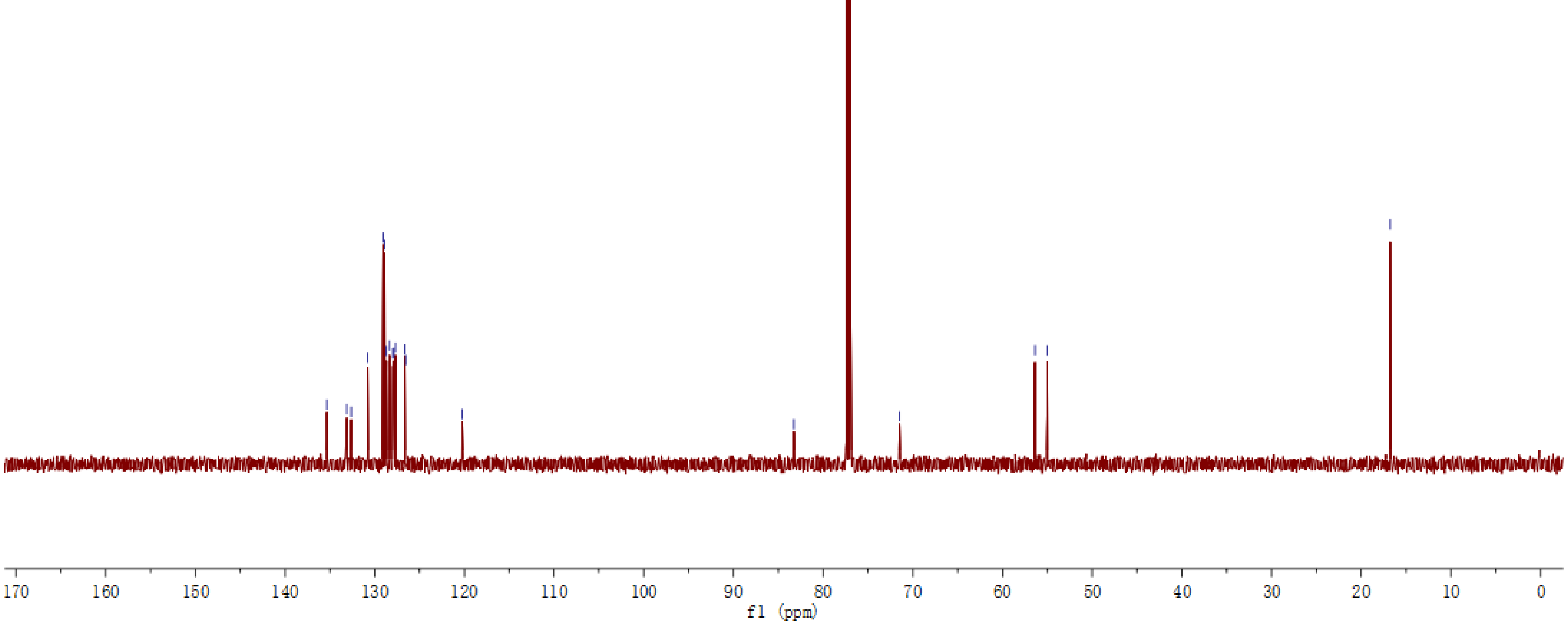

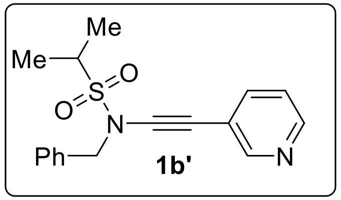

*[1]**H NMR:*** 600 MHz in $CDCl_3$

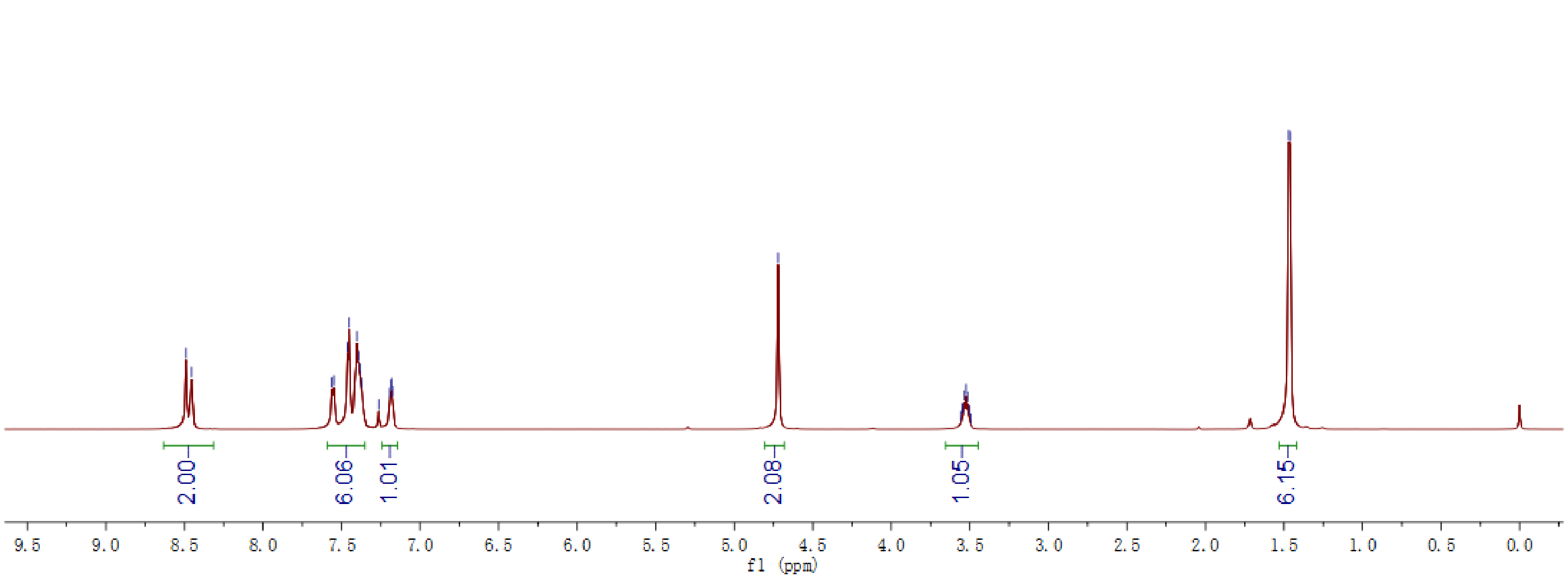

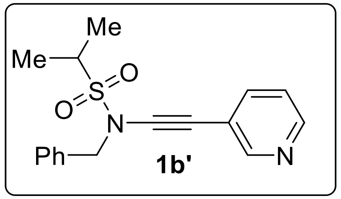

^{13}**C NMR:** 150 MHz in $CDCl_3$

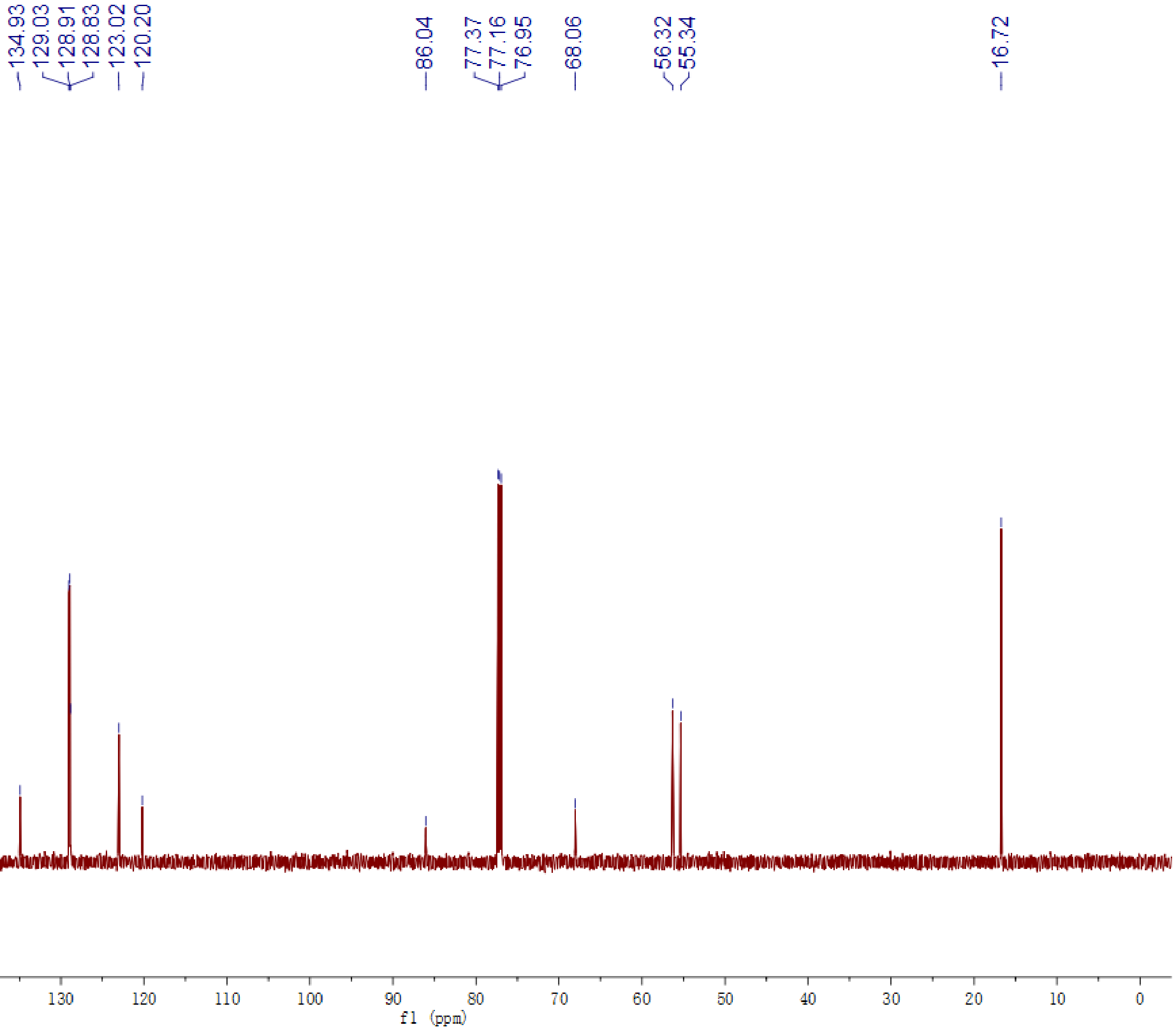

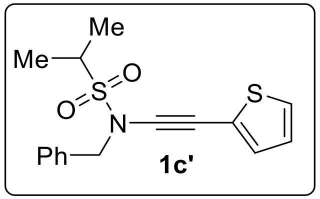

1c'

^{1}H NMR: 400 MHz in $CDCl_3$

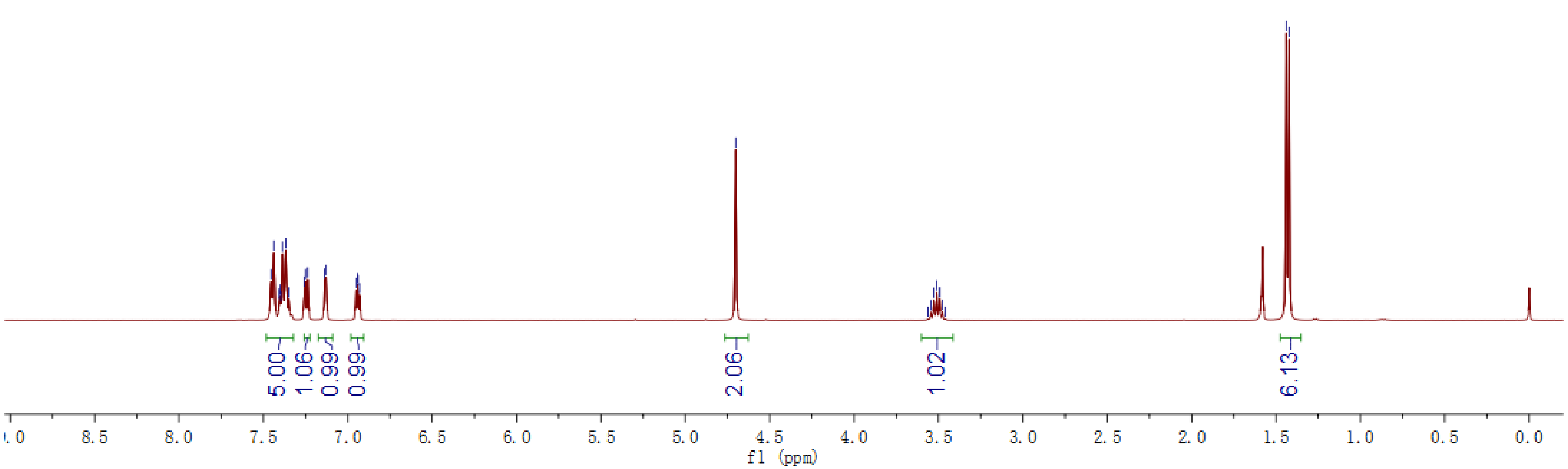

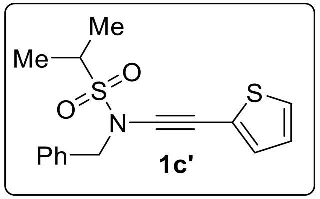

^{13}C ***NMR:*** 100 MHz in $CDCl_3$

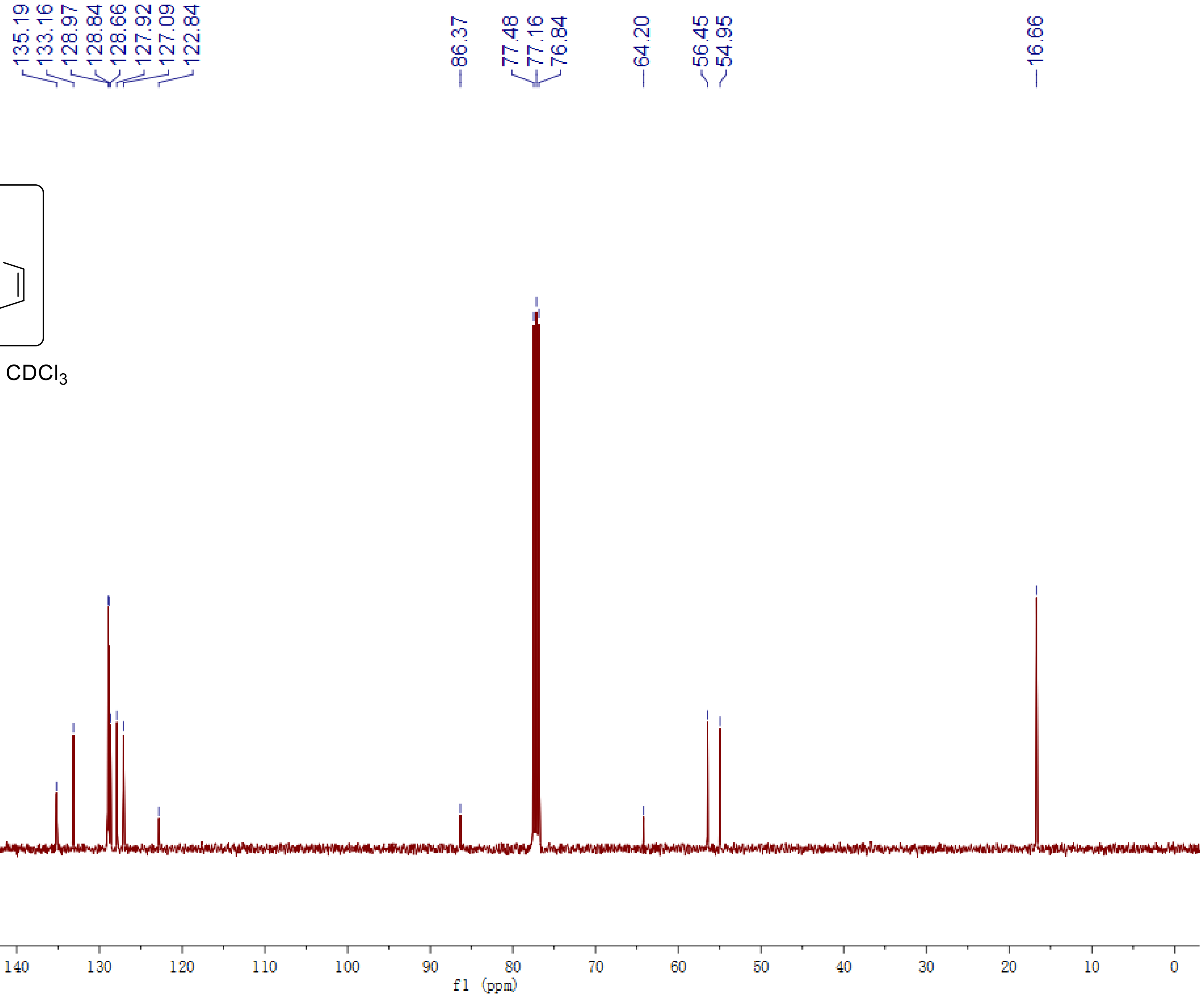

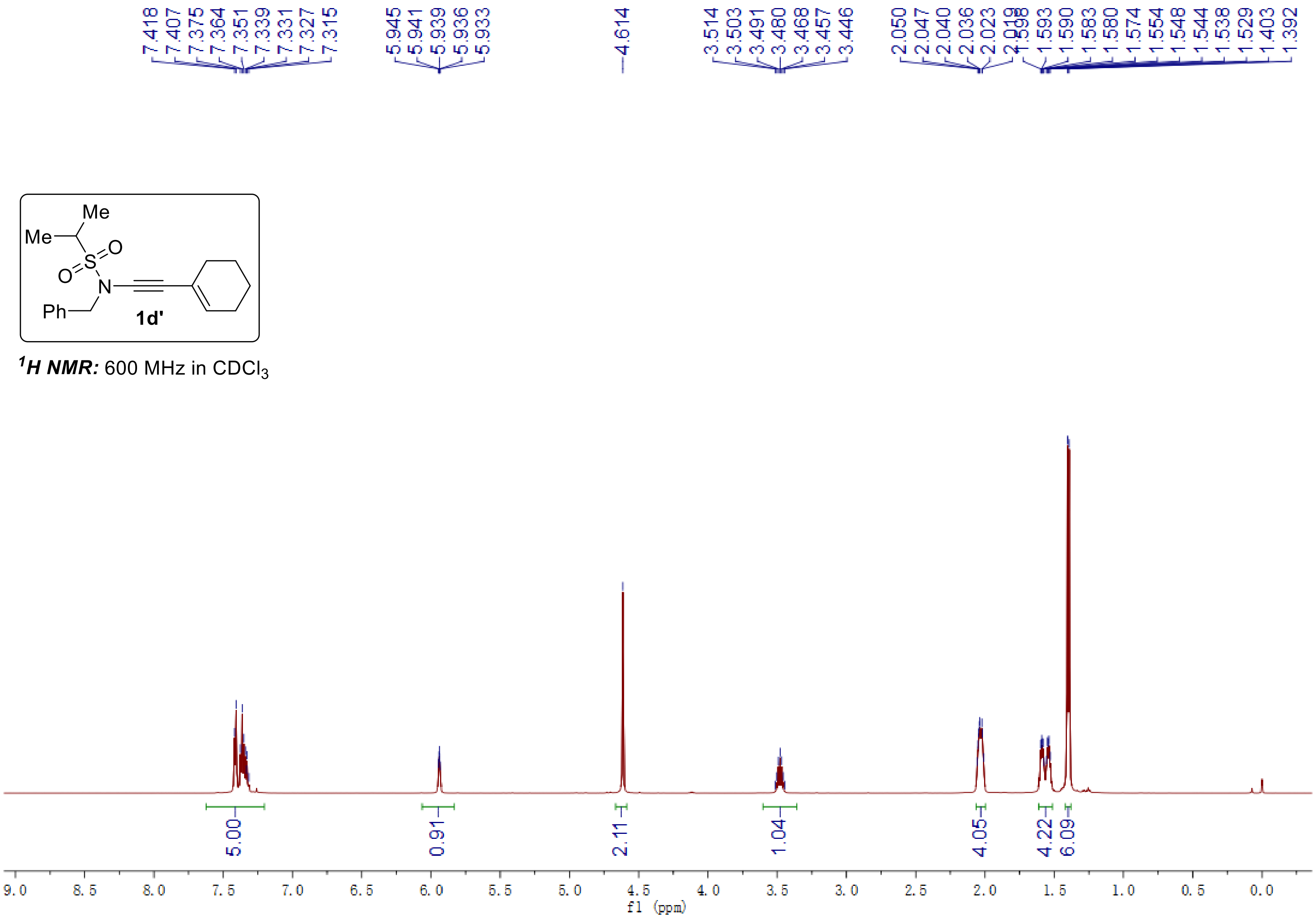

^{1}H NMR: 600 MHz in $CDCl_3$

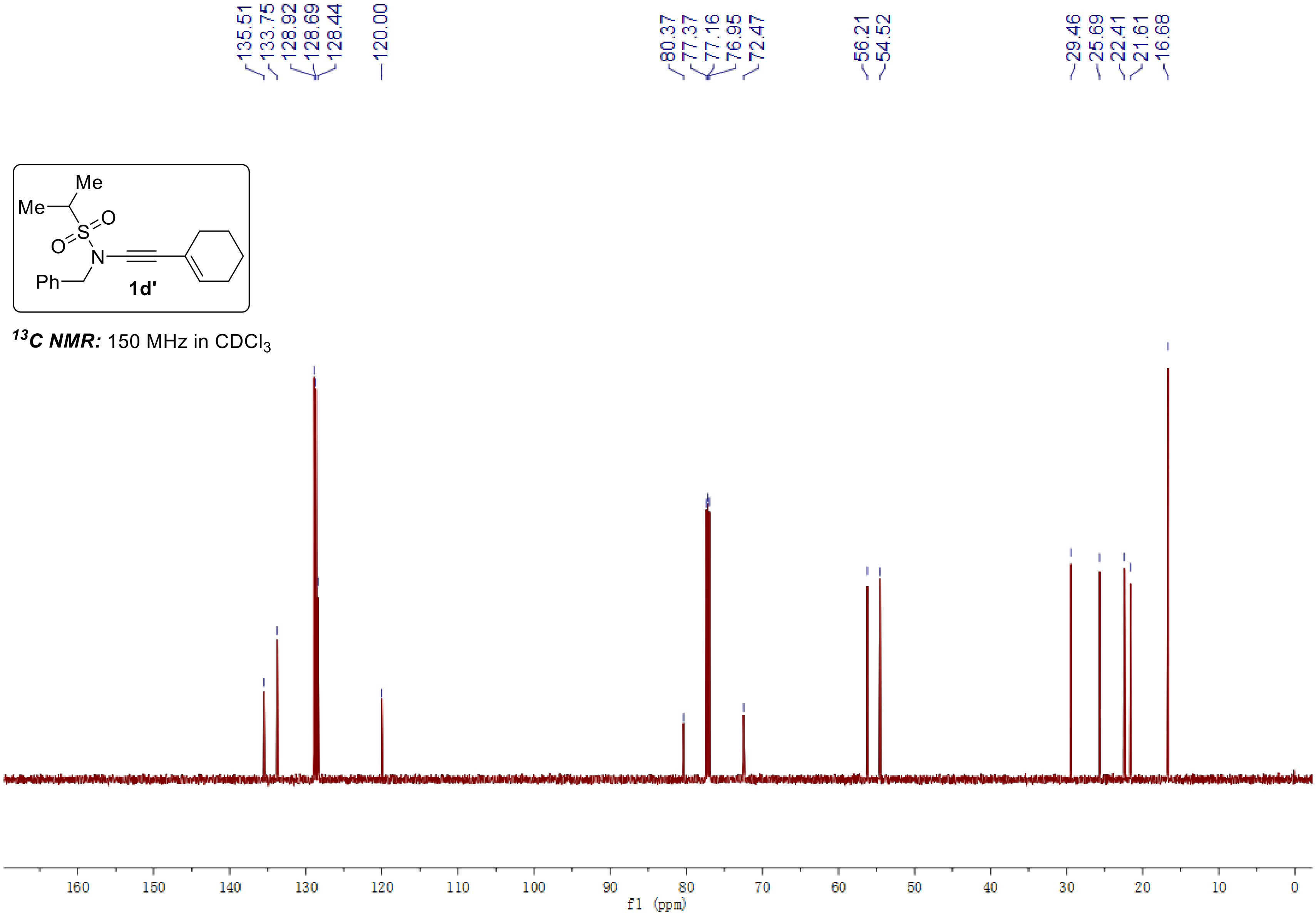
135.51
133.75
128.92
128.69
128.44
120.00
80.37
77.37
77.16
76.95
72.47
56.21
54.52
29.46
25.69
22.41
21.61
16.68
Me
Me
O
O
S
N
Ph
1d'
13C NMR: 150 MHz in CDCl3
160
150
140
130
120
110
100
90
80
70
60
50
40
30
20
10
0
f1 (ppm)

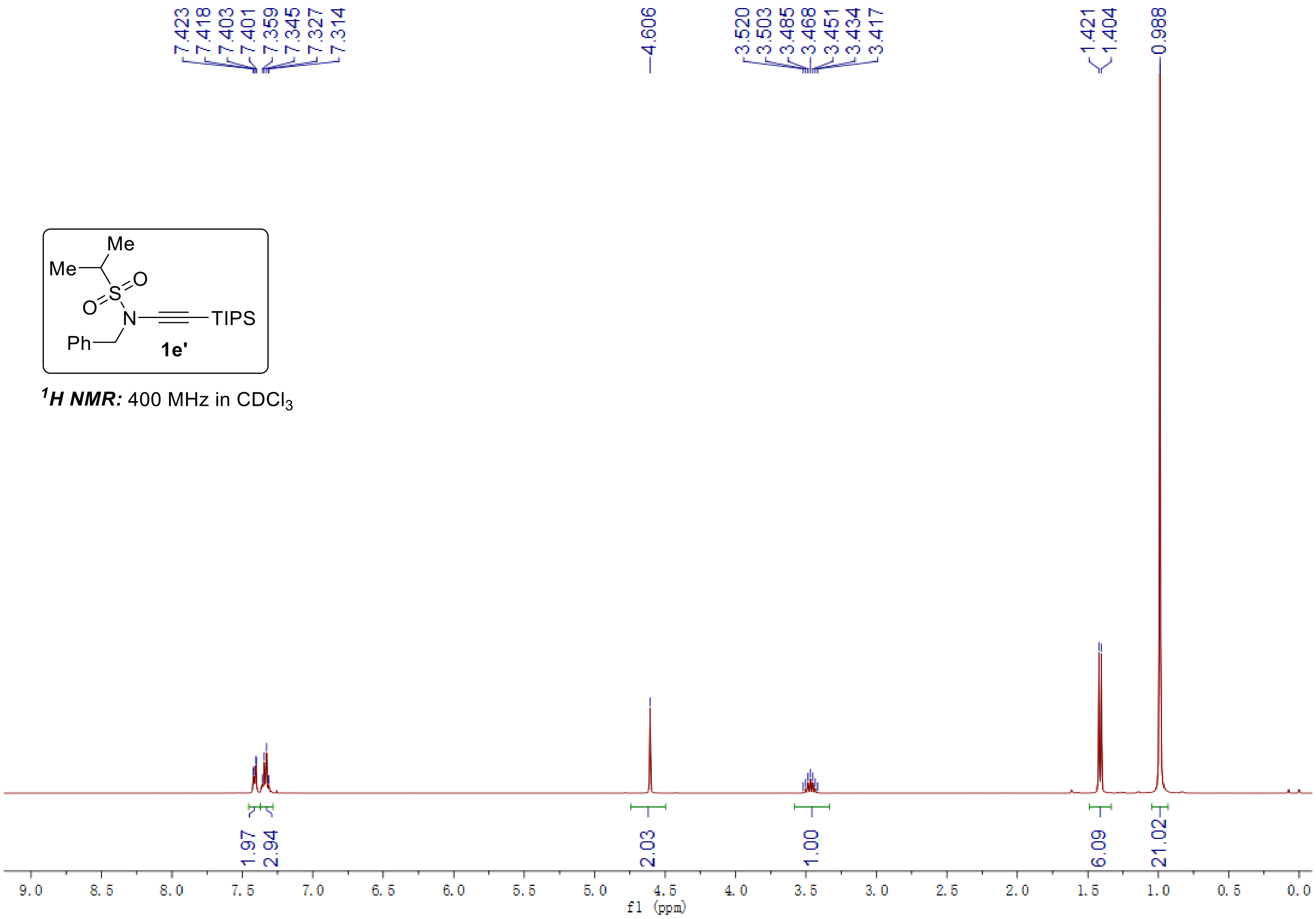

^{1}H NMR: 400 MHz in $CDCl_3$

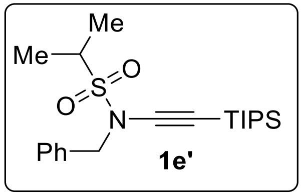

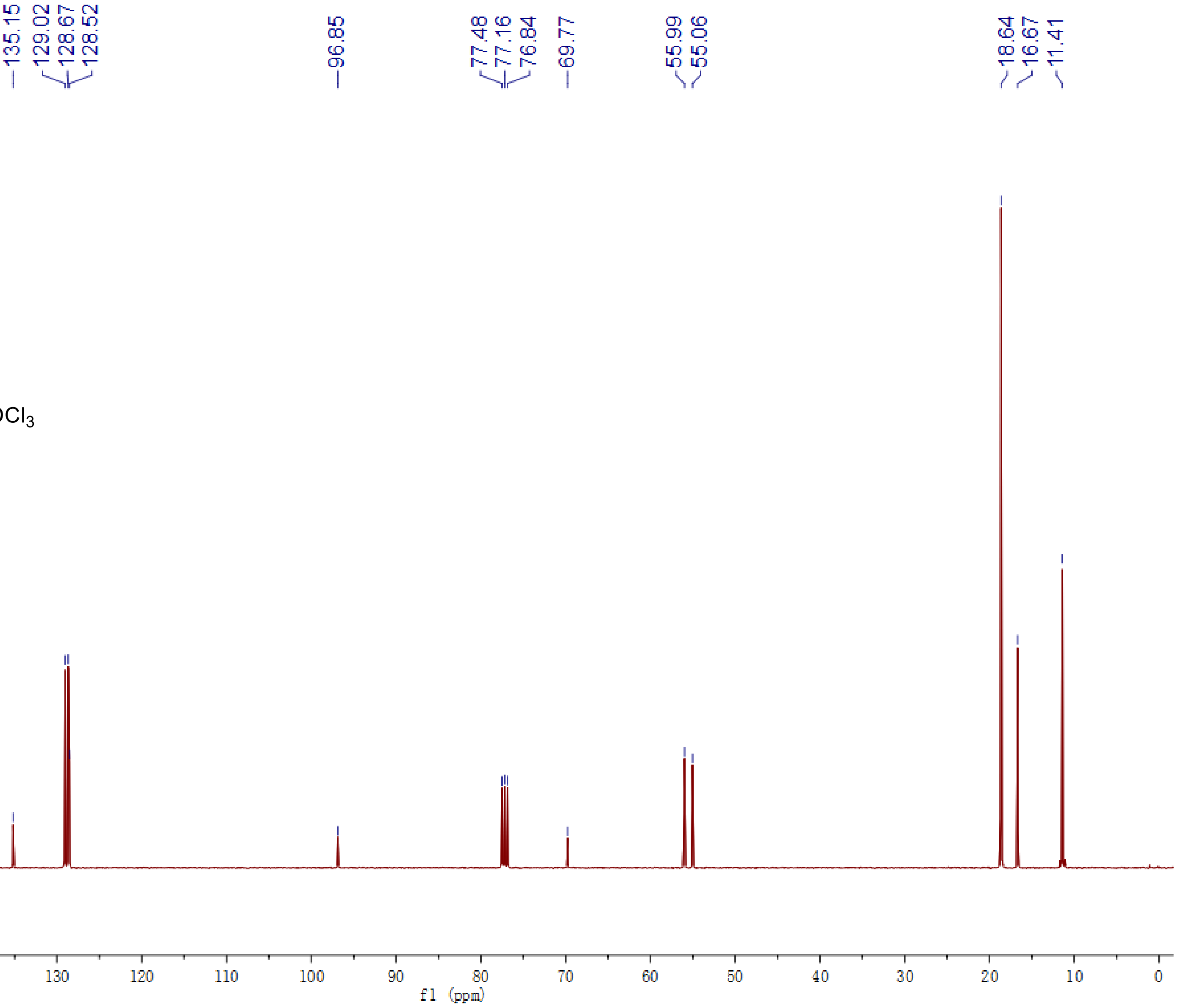

^{13}C NMR: 100 MHz in $CDCl_3$

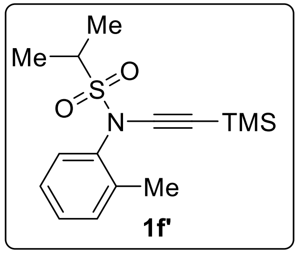

^{1}***H NMR:*** 500 MHz in $CDCl_3$

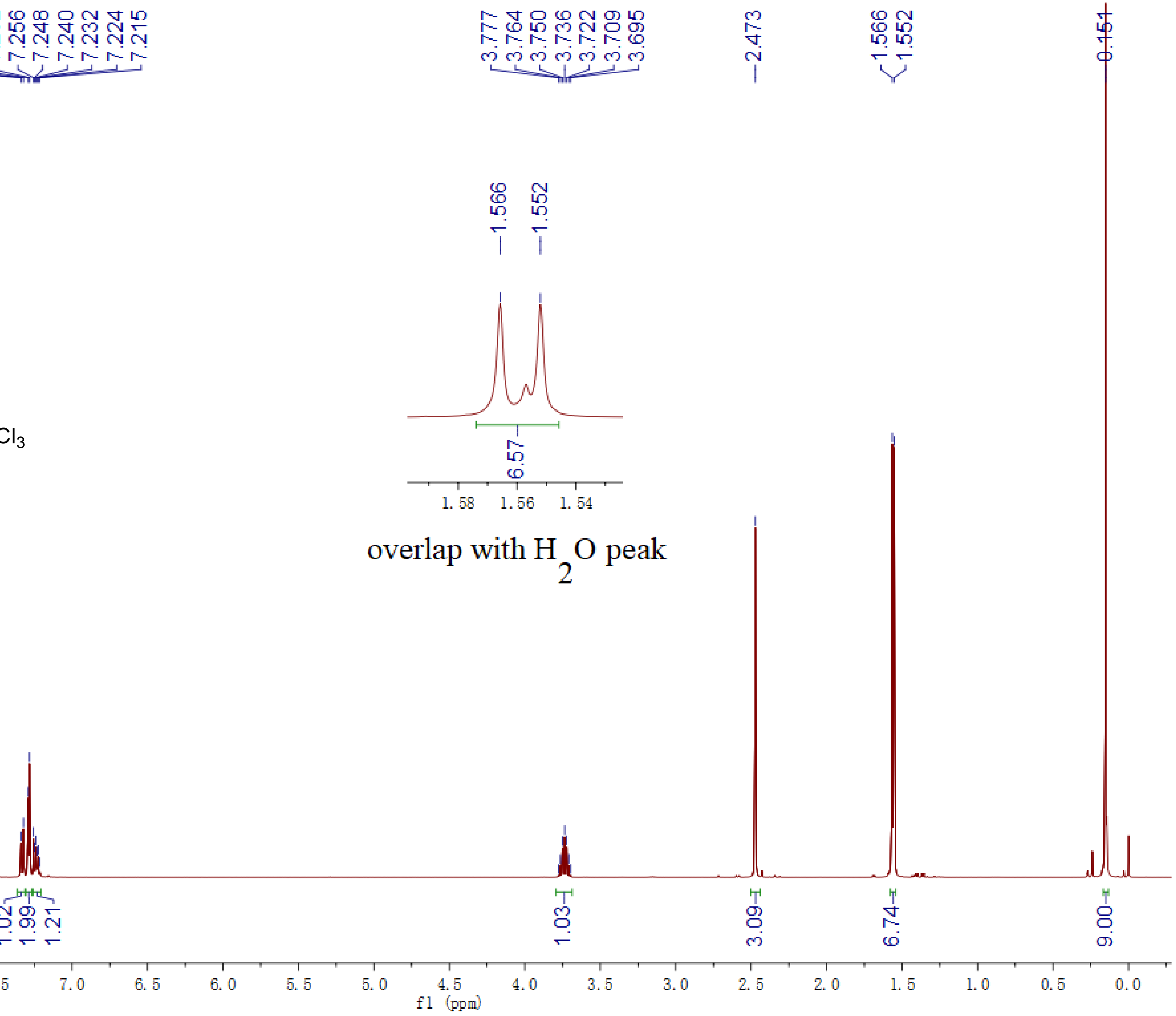

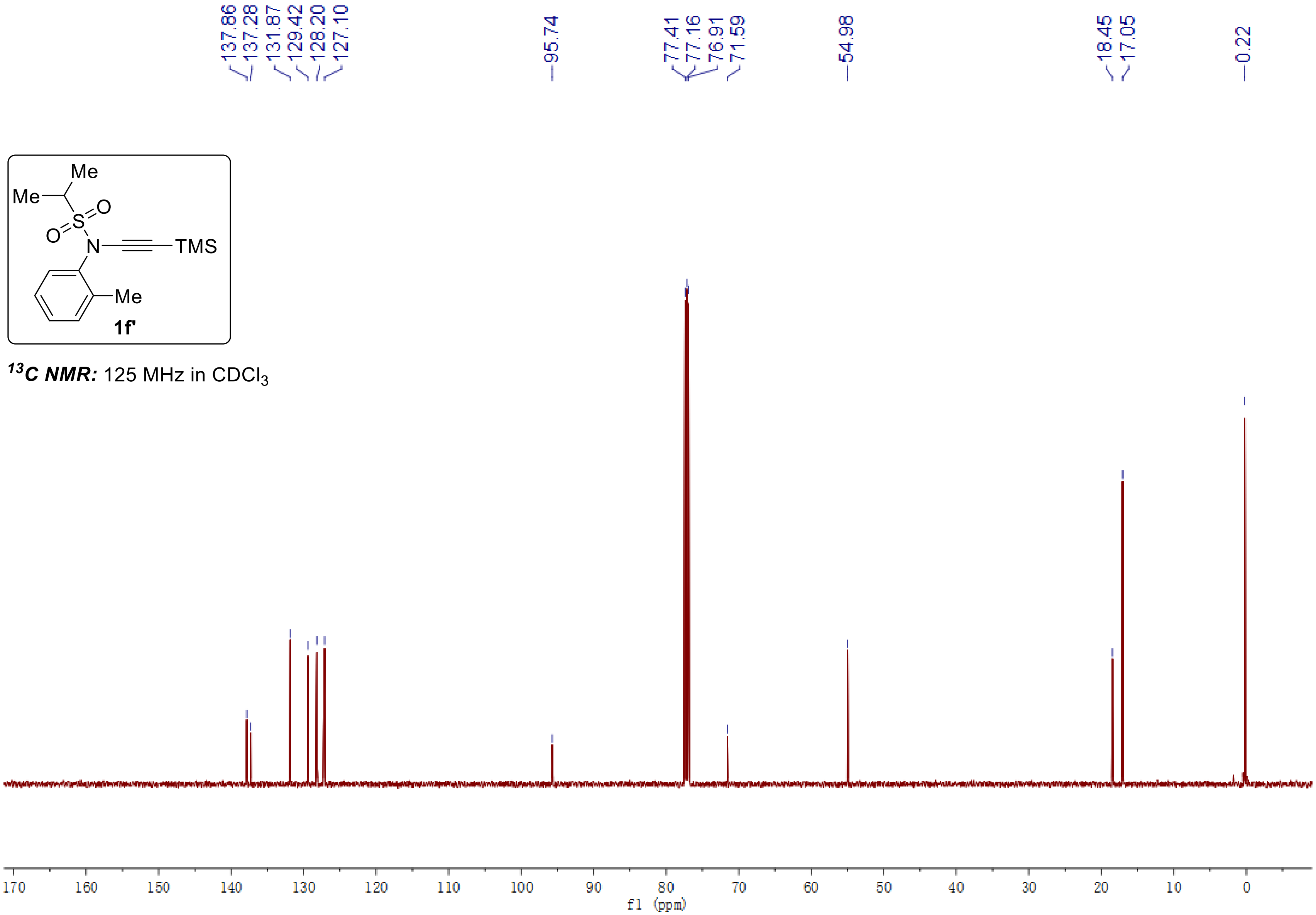
137.86
137.28
131.87
129.42
128.20
127.10
95.74
77.41
77.16
76.91
71.59
54.98
18.45
17.05
0.22
Me
Me
S
O
O
N
TMS
Me
1f'
^{13}C NMR: 125 MHz in $CDCl_3$
170
160
150
140
130
120
110
100
90
80
70
60
50
40
30
20
10
0
f1 (ppm)

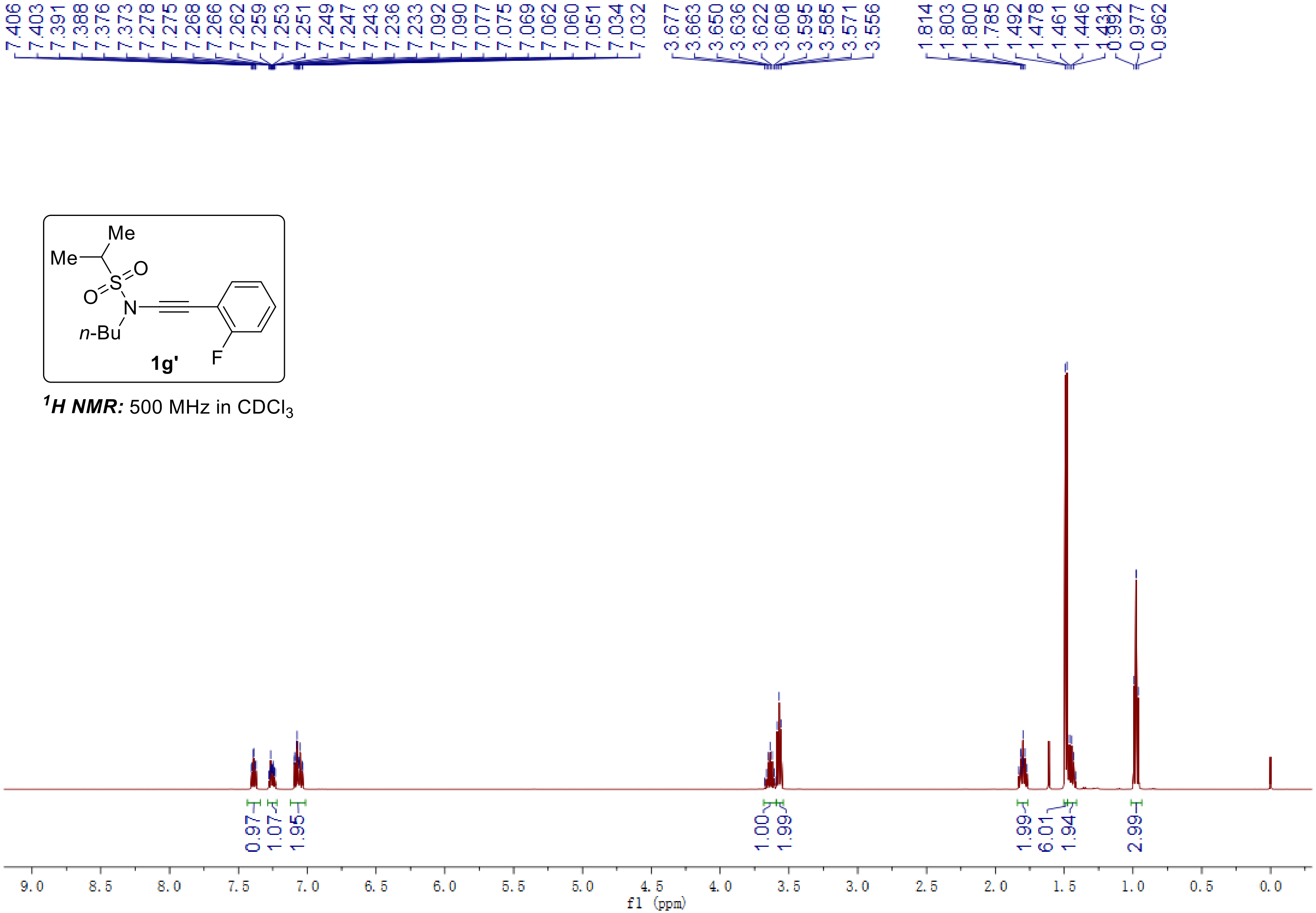

^{1}H NMR: 500 MHz in $CDCl_3$

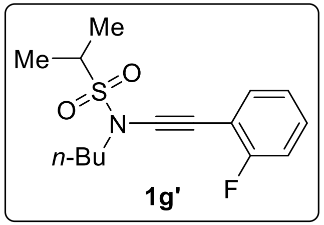

^{13}**_C NMR:_** 125 MHz in $CDCl_3$

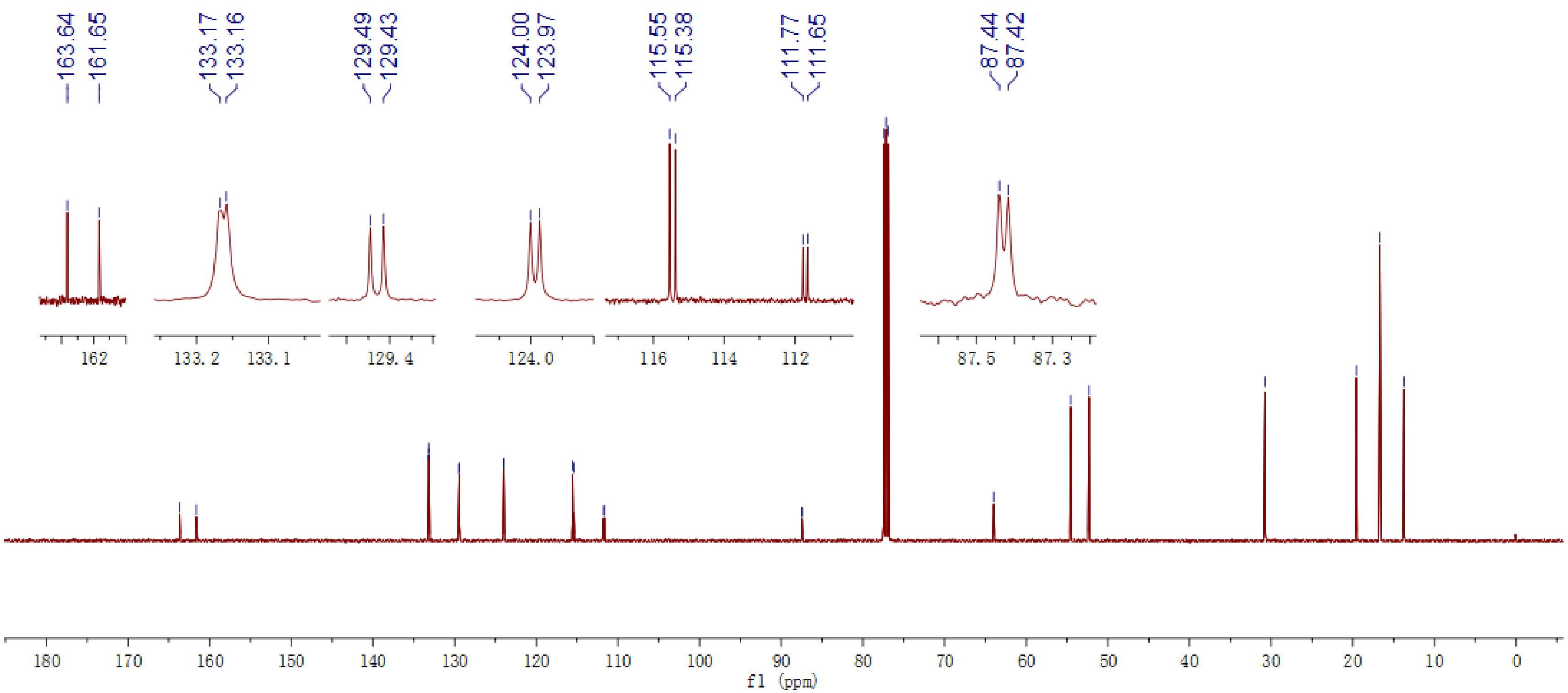

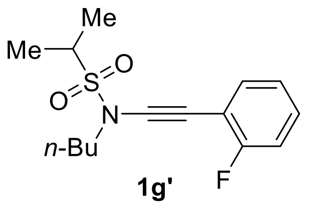
Me
Me
O
S
O
N
n-Bu
F

1g'

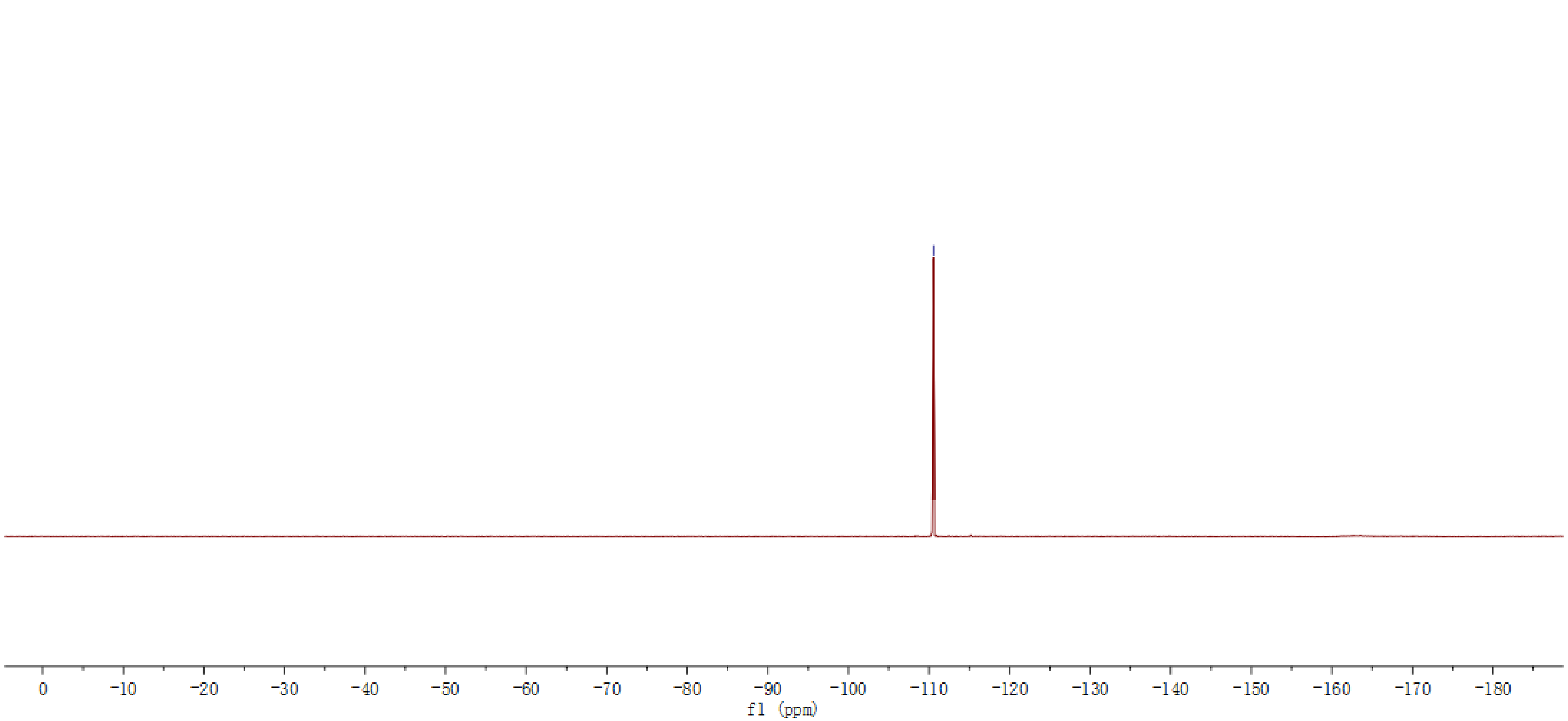
-110.551
0
-10
-20
-30
-40
-50
-60
-70
-80
-90
-100
-110
-120
-130
-140
-150
-160
-170
-180
f1 (ppm)

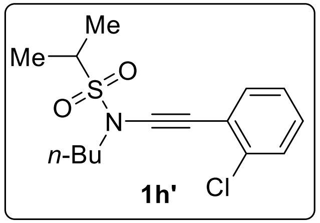

[1]*H NMR:* 500 MHz in $CDCl_3$

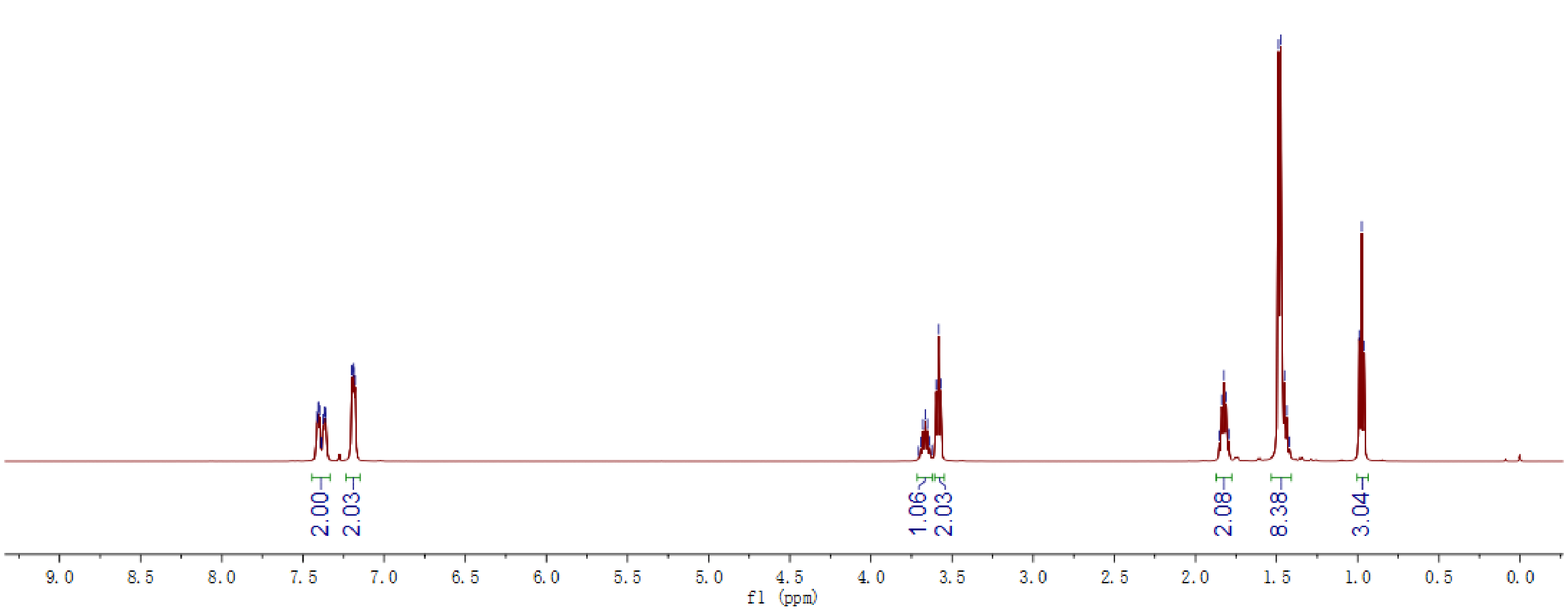

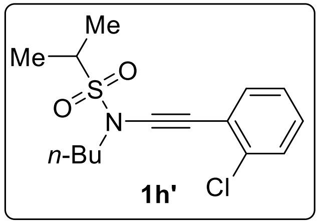

^{13}**C NMR:** 125 MHz in $CDCl_3$

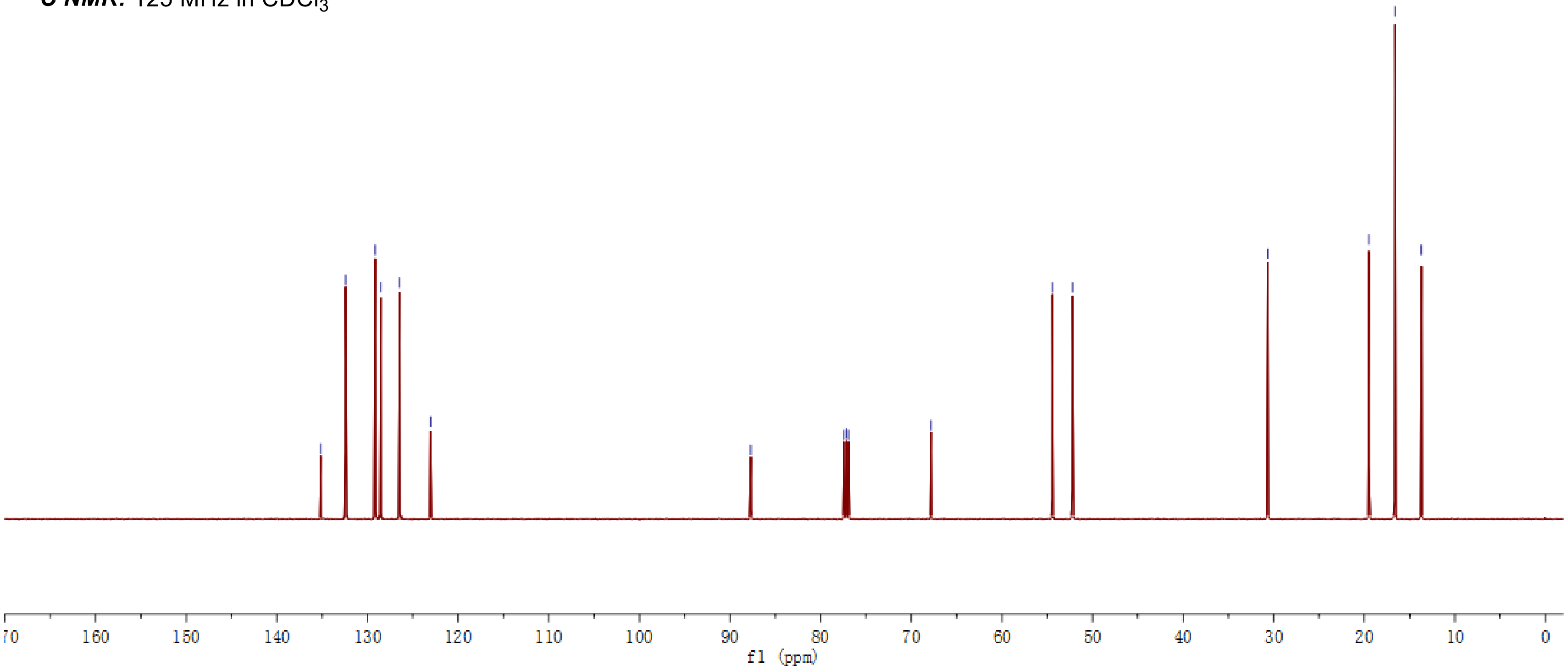

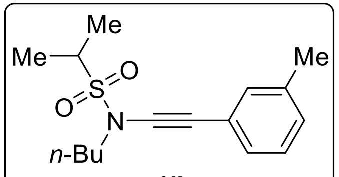

1i'

[1]***H NMR:*** 500 MHz in $CDCl_3$

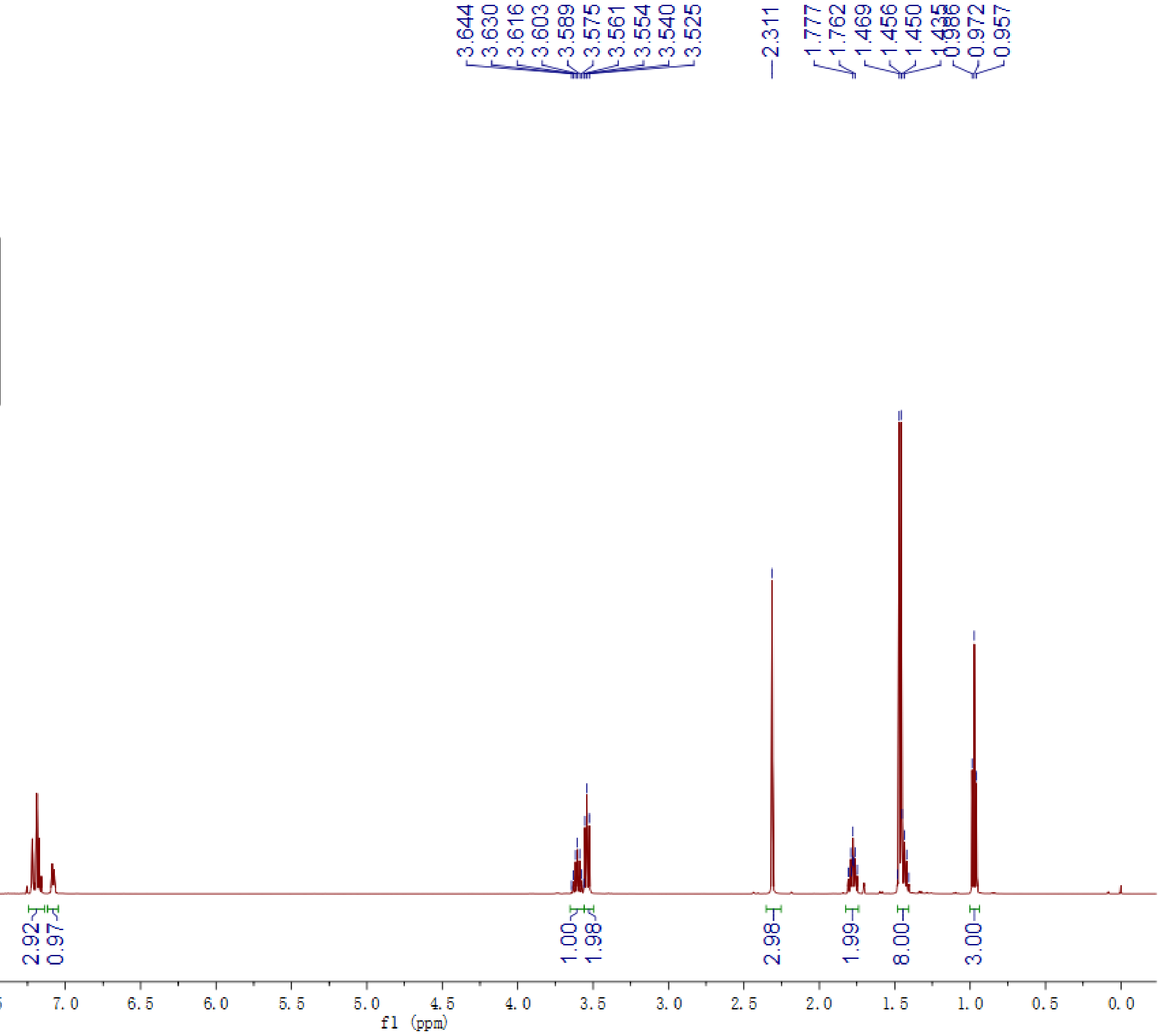

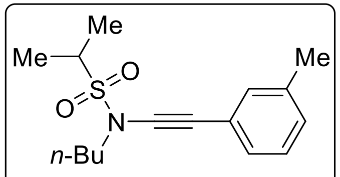

1i'

^{13}C NMR: 125 MHz in $CDCl_3$

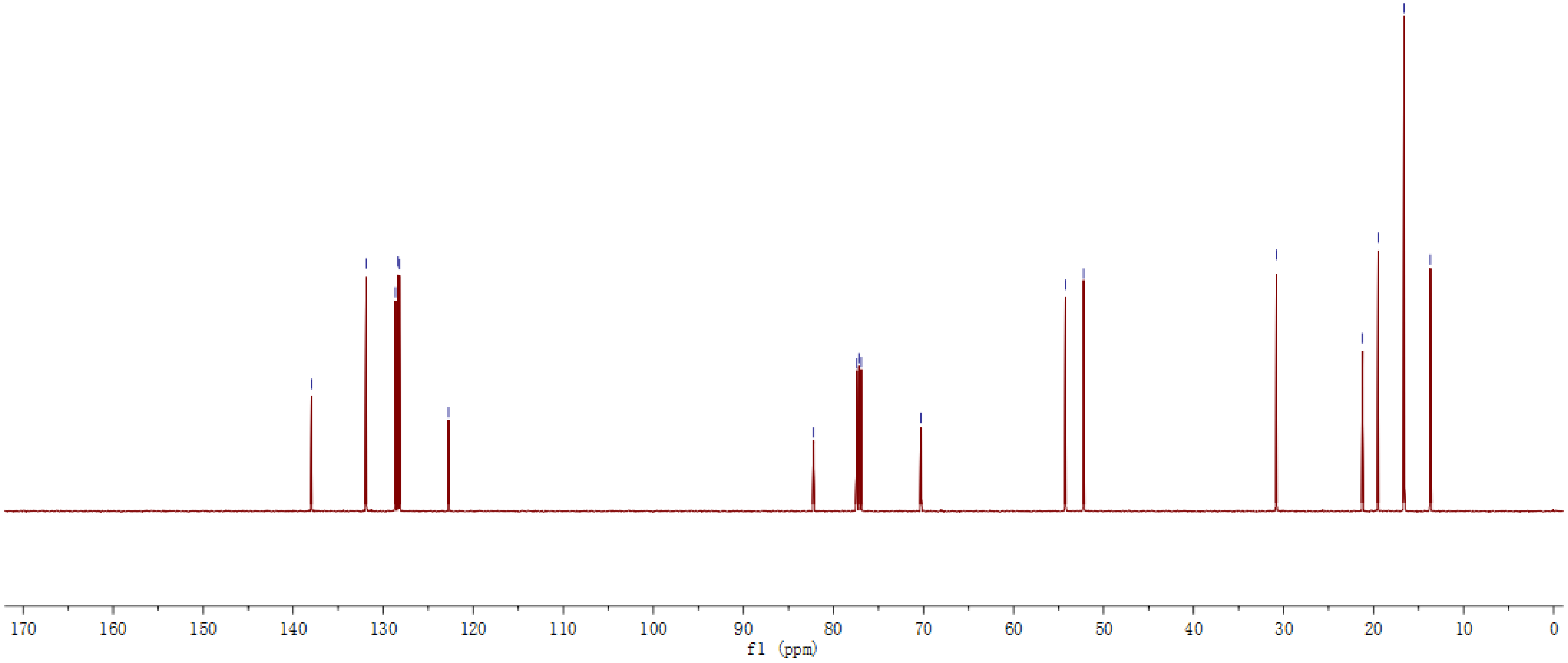

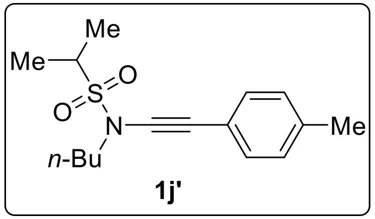

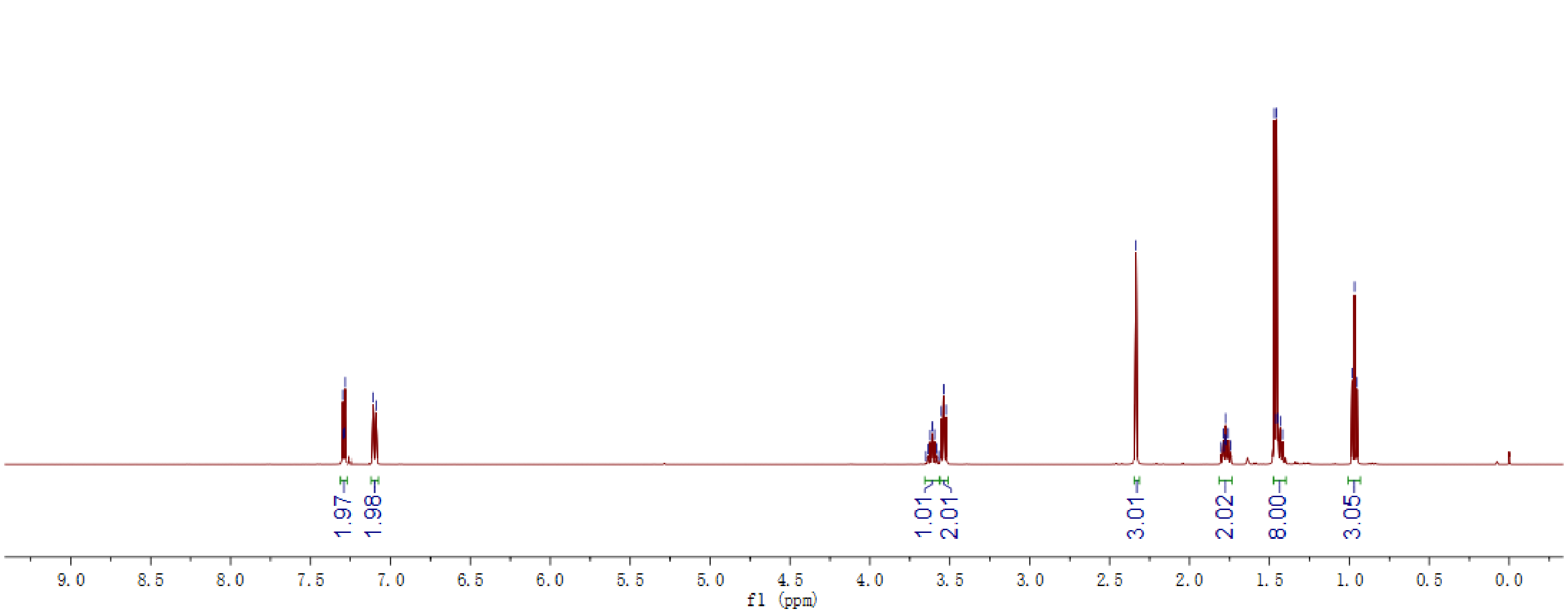

^{1}H NMR: 500 MHz in $CDCl_3$

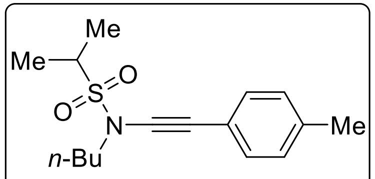

1j'

^{13}**_C NMR:_** 125 MHz in $CDCl_3$

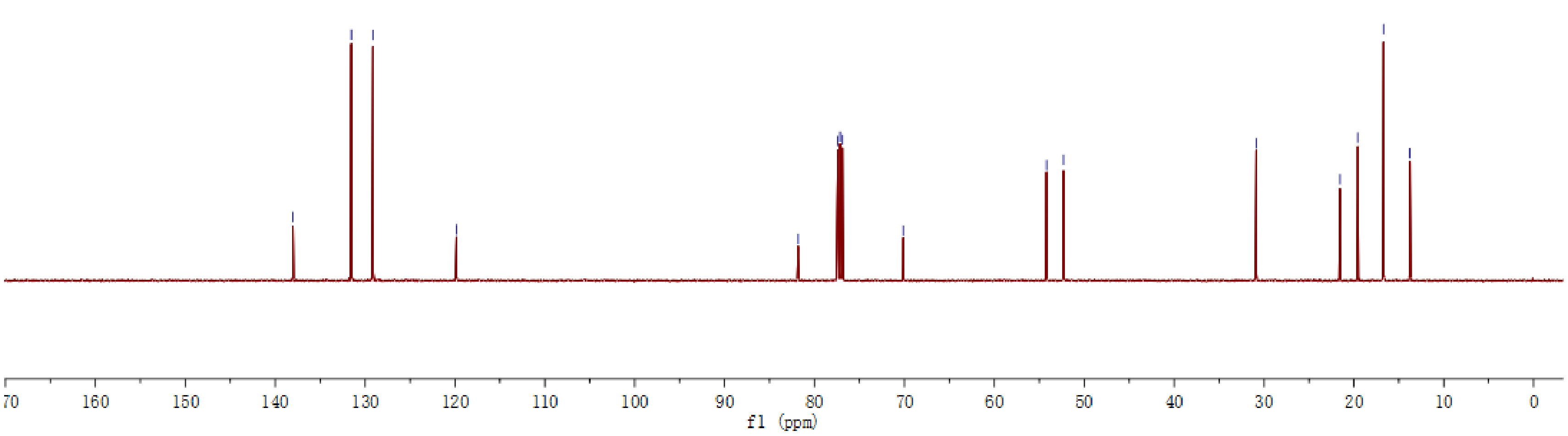

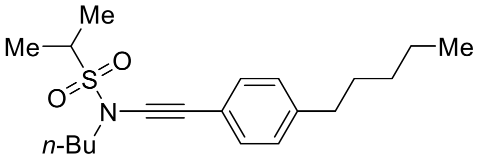

1k'

^{1}H NMR: 500 MHz in $CDCl_3$

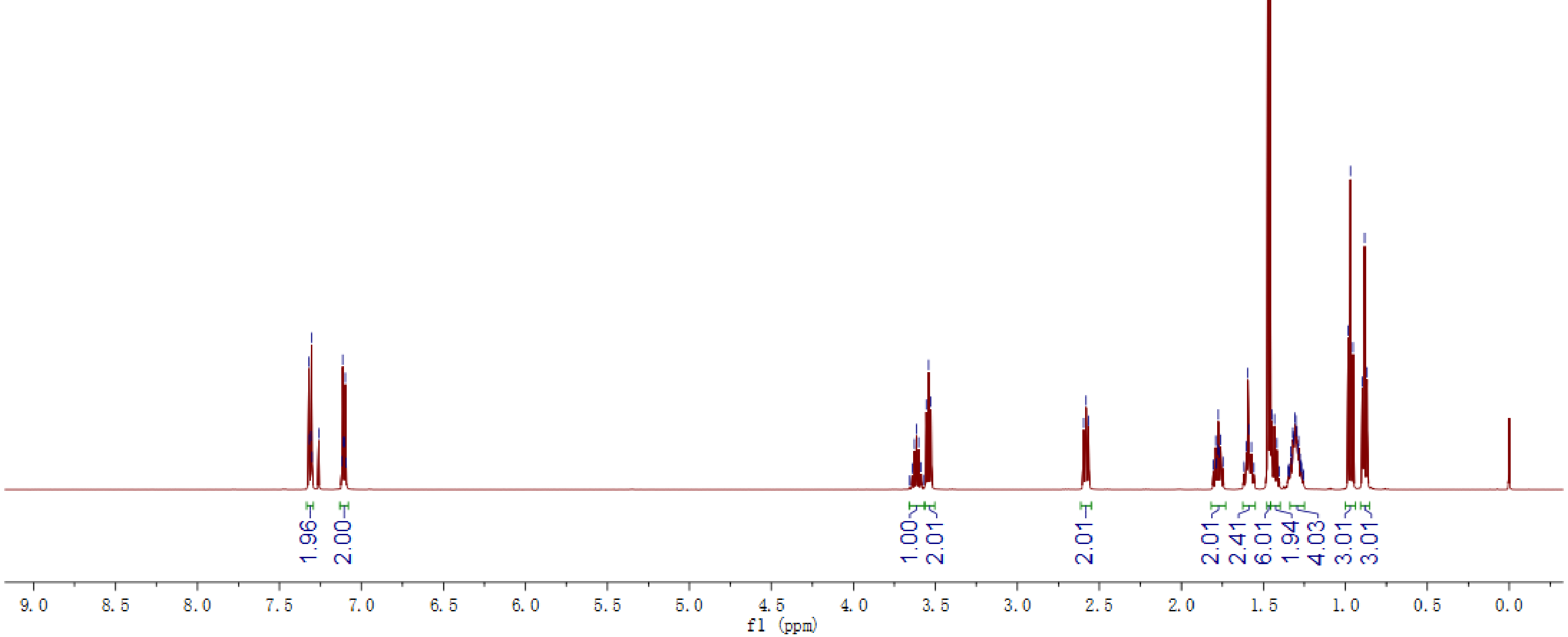

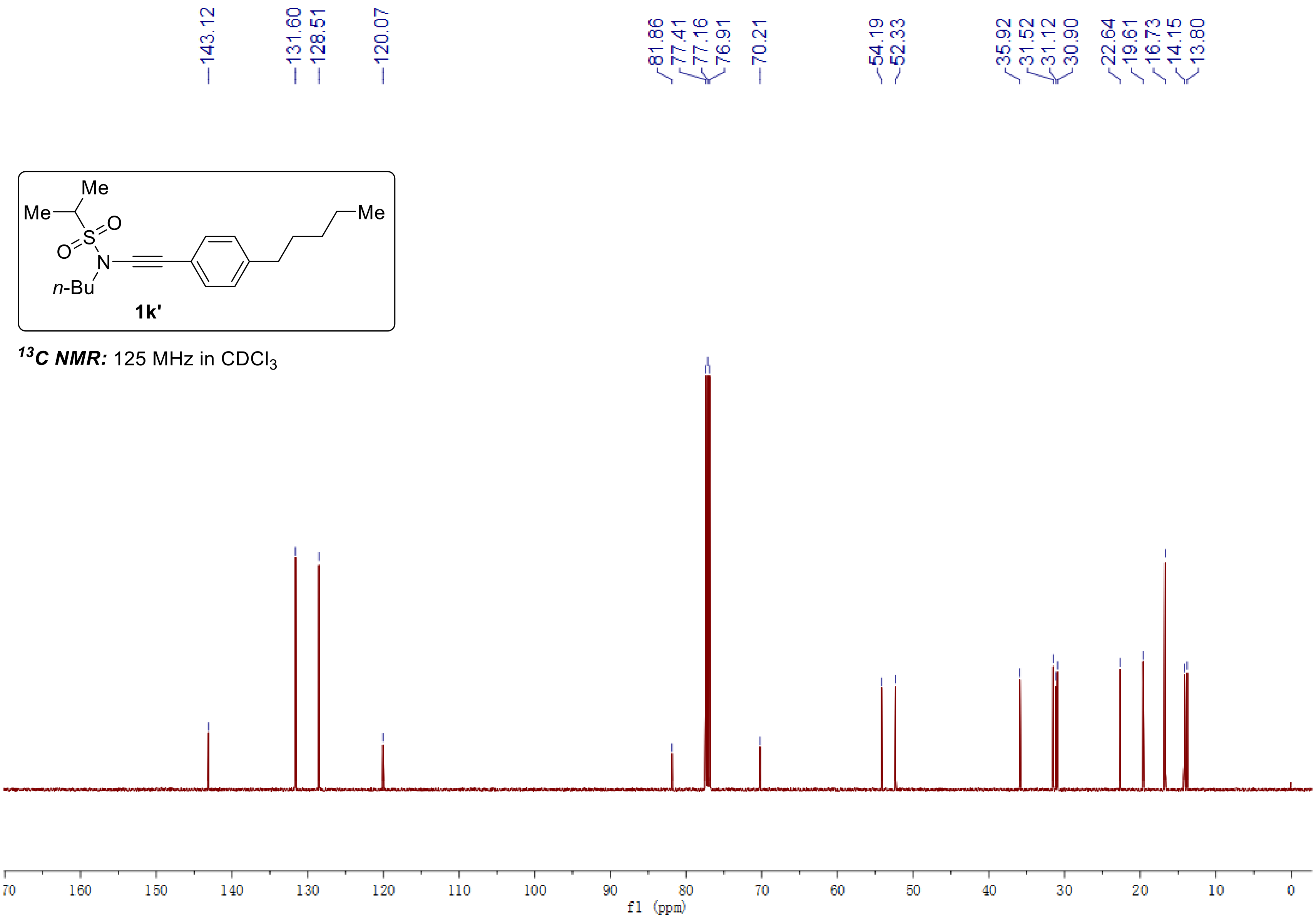

^{13}***C NMR:*** 125 MHz in $CDCl_3$

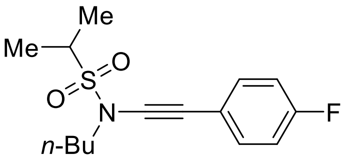

1l'

^{1}H NMR: 500 MHz in $CDCl_3$

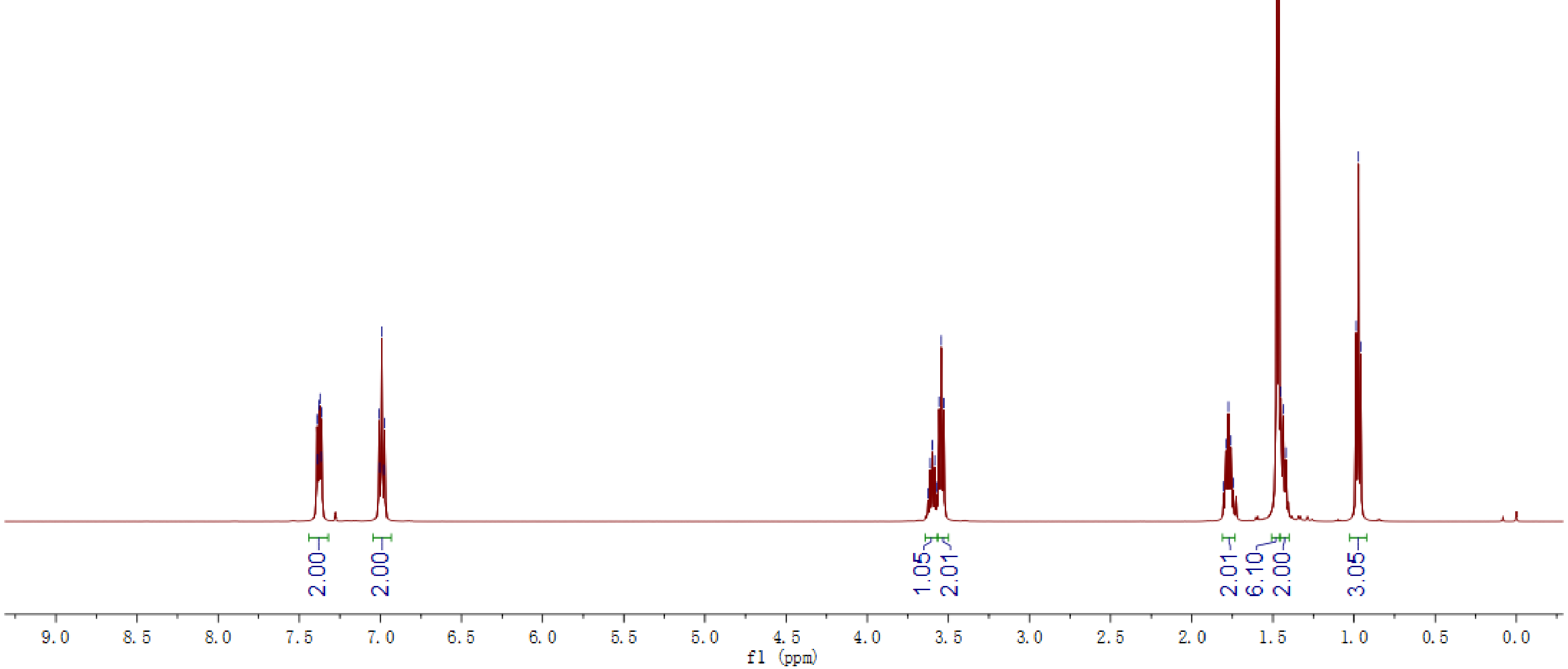

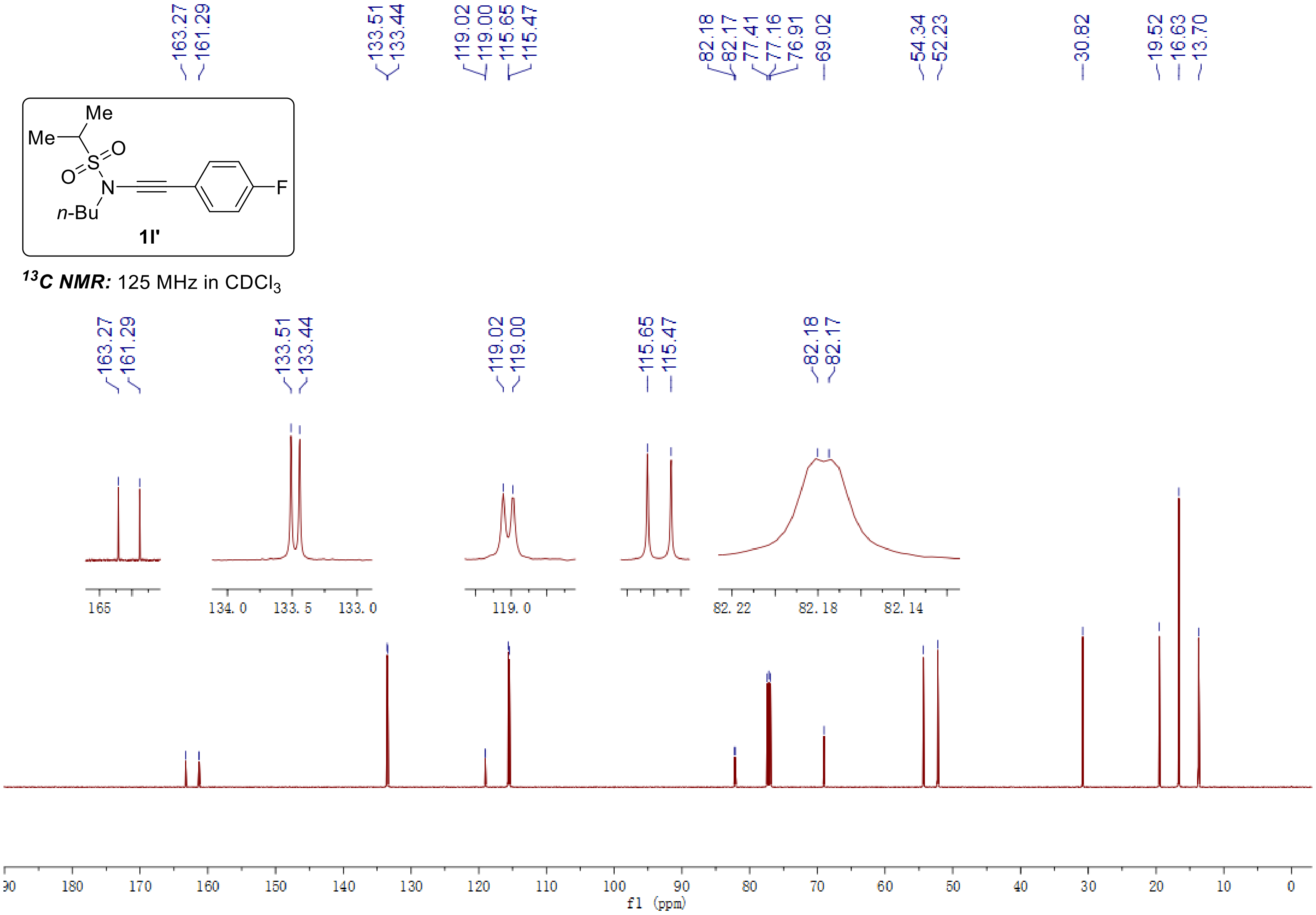
163.27
161.29
133.51
133.44
119.02
119.00
115.65
115.47
82.18
82.17
77.41
77.16
76.91
69.02
54.34
52.23
30.82
19.52
16.63
13.70
Me
Me
S
O
O
N
F
n-Bu
1l'
13C NMR: 125 MHz in CDCl3
165
134. 0
133. 5
133. 0
119. 0
82. 22
82. 18
82. 14
f1 (ppm)

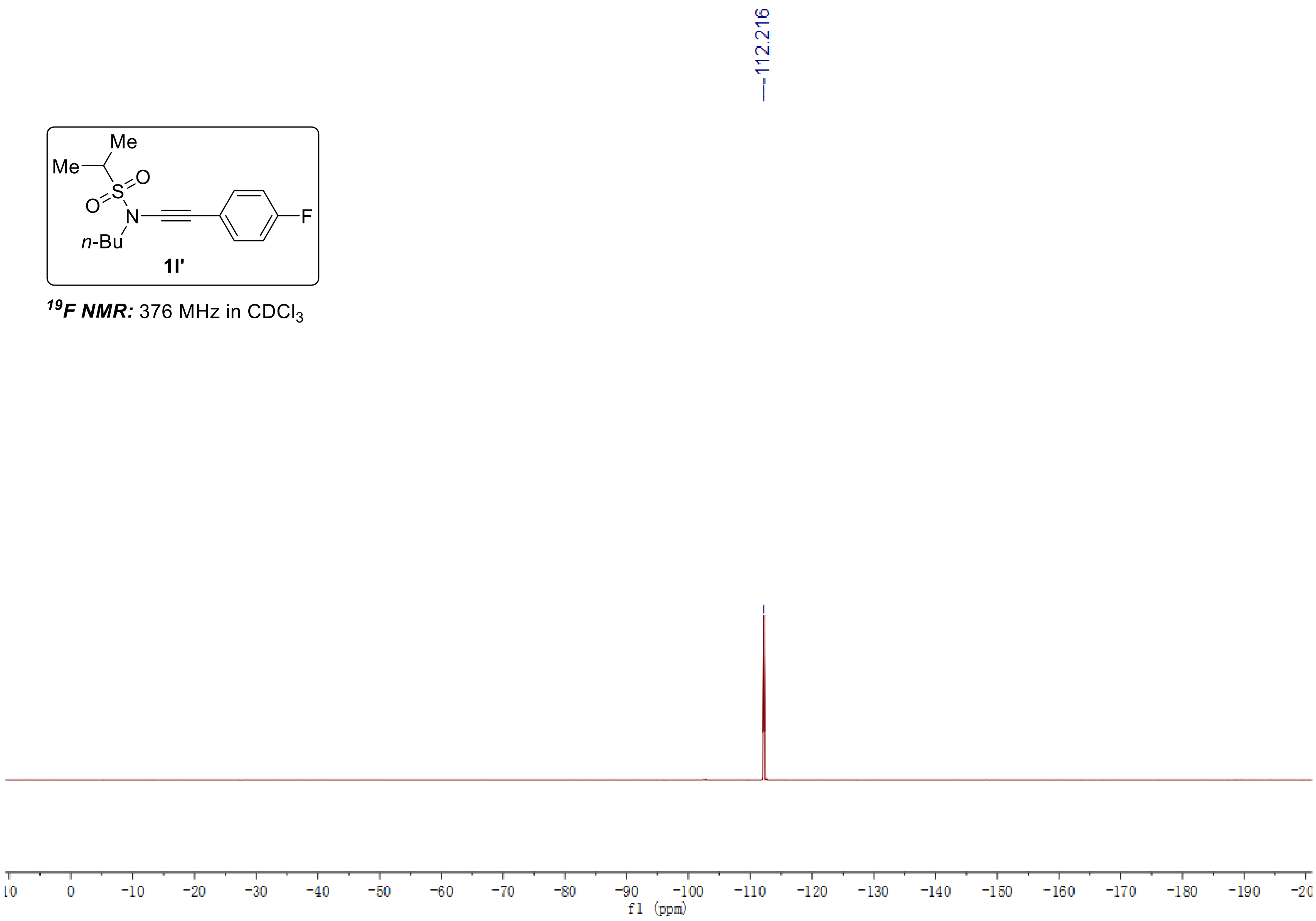
-112.216
Me
Me
O
S
O
N
F
n-Bu
1l'
19F NMR: 376 MHz in CDCl3
10
0
-10
-20
-30
-40
-50
-60
-70
-80
-90
-100
-110
-120
-130
-140
-150
-160
-170
-180
-190
-20
f1 (ppm)

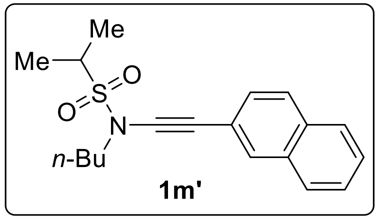

1m'

^{1}H NMR: 500 MHz in $CDCl_3$

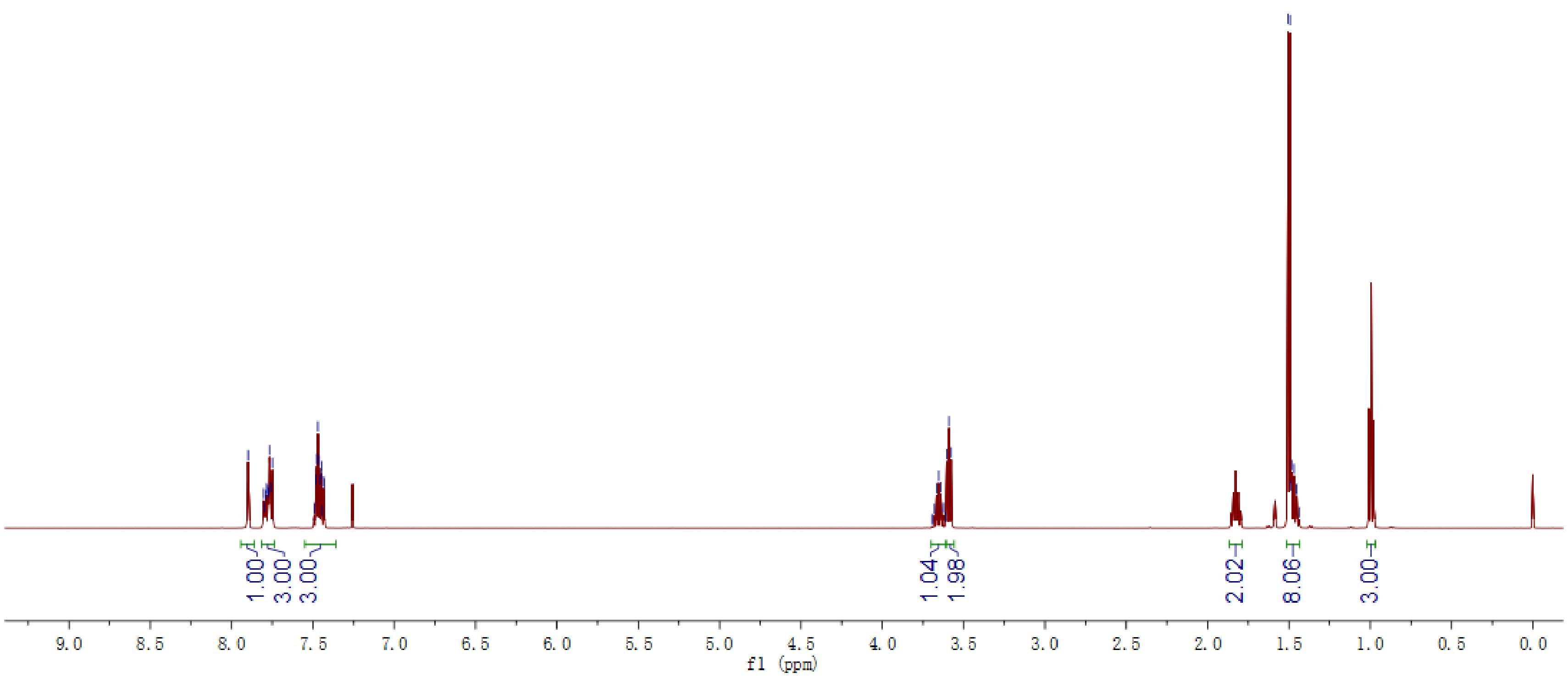

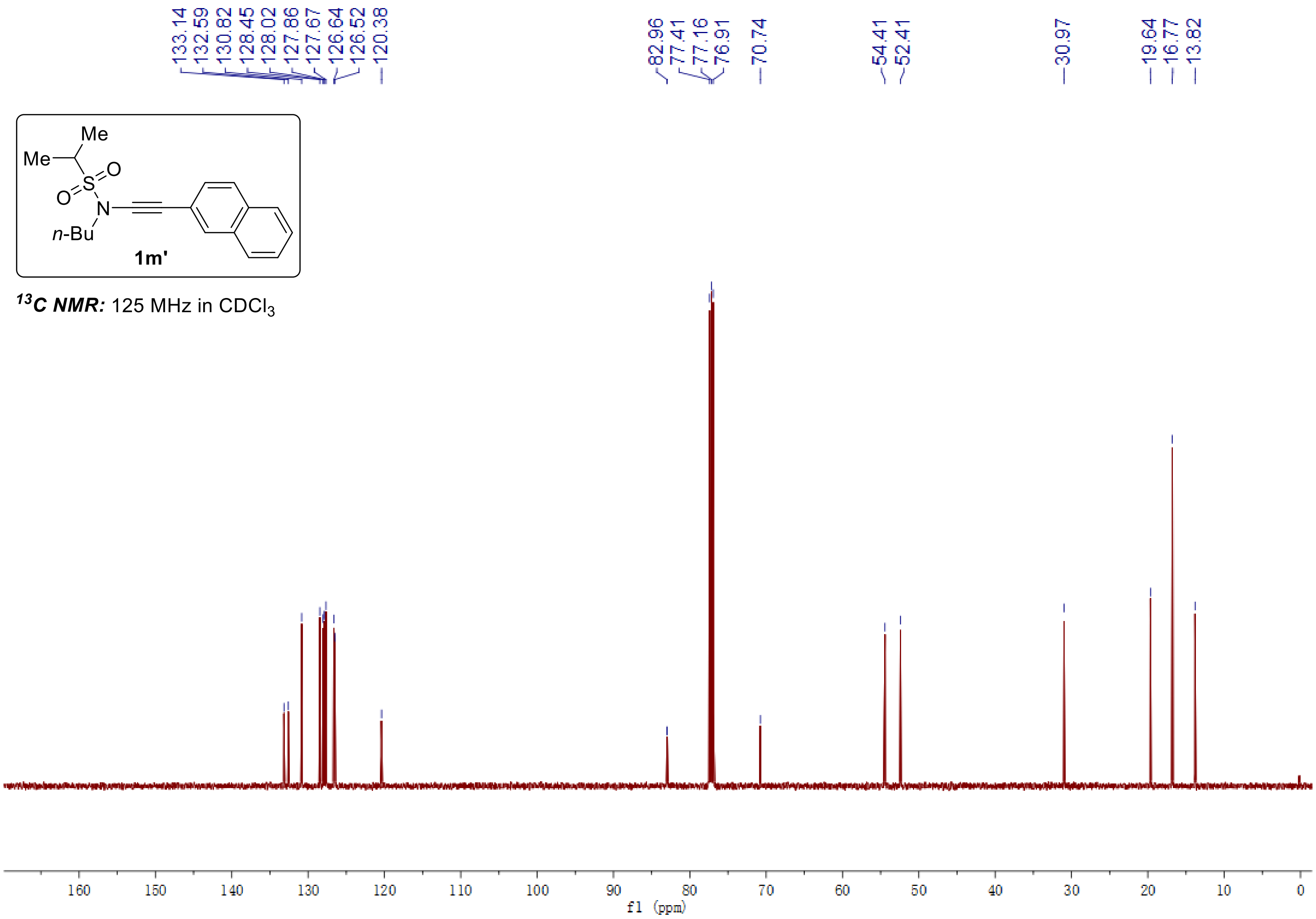
133.14
132.59
130.82
128.45
128.02
127.86
127.67
126.64
126.52
120.38
82.96
77.41
77.16
76.91
70.74
54.41
52.41
30.97
19.64
16.77
13.82
Me
Me
O
S
O
N
n-Bu
1m'
13C NMR: 125 MHz in CDCl3
f1 (ppm)

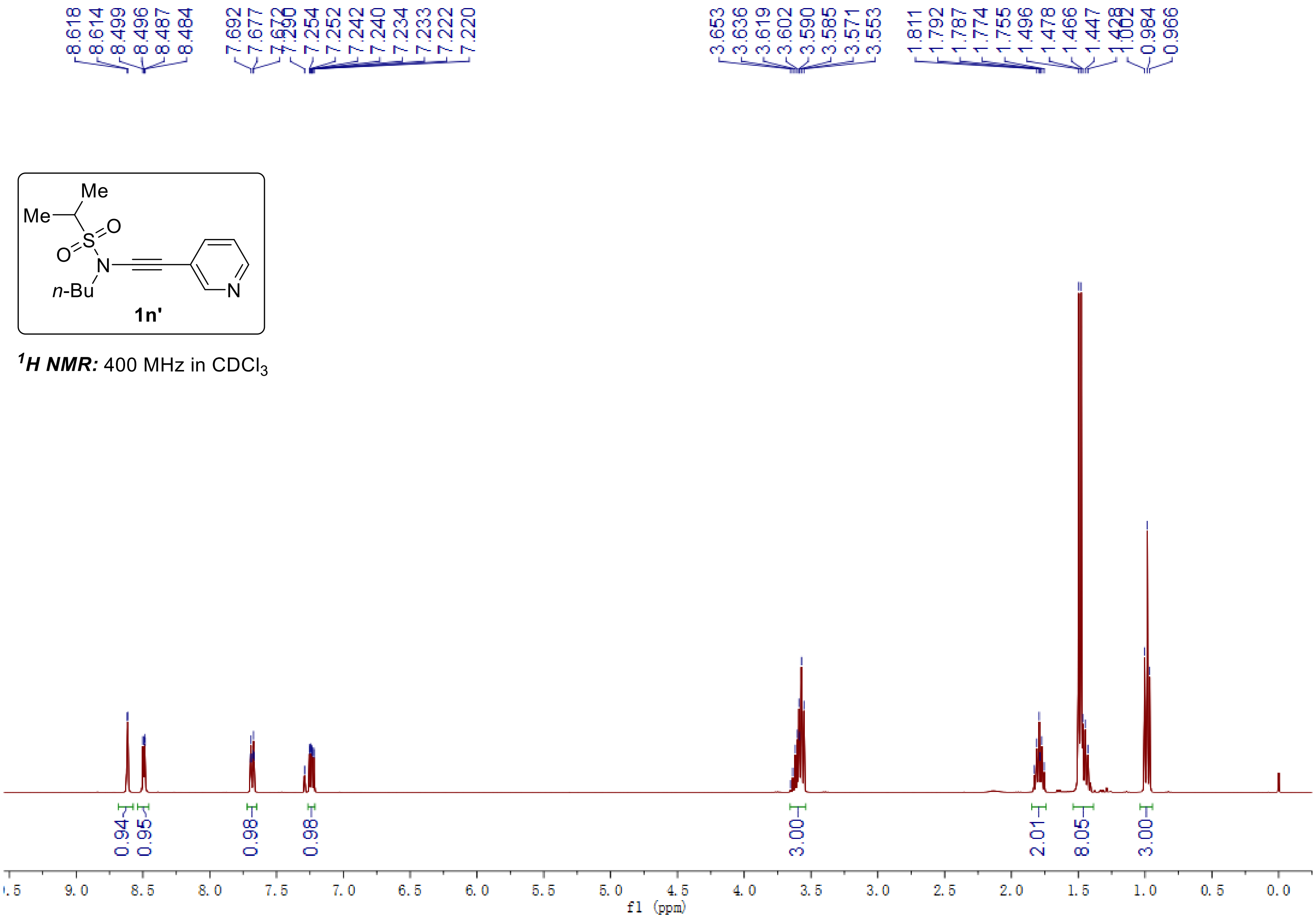

^{1}H NMR: 400 MHz in $CDCl_3$

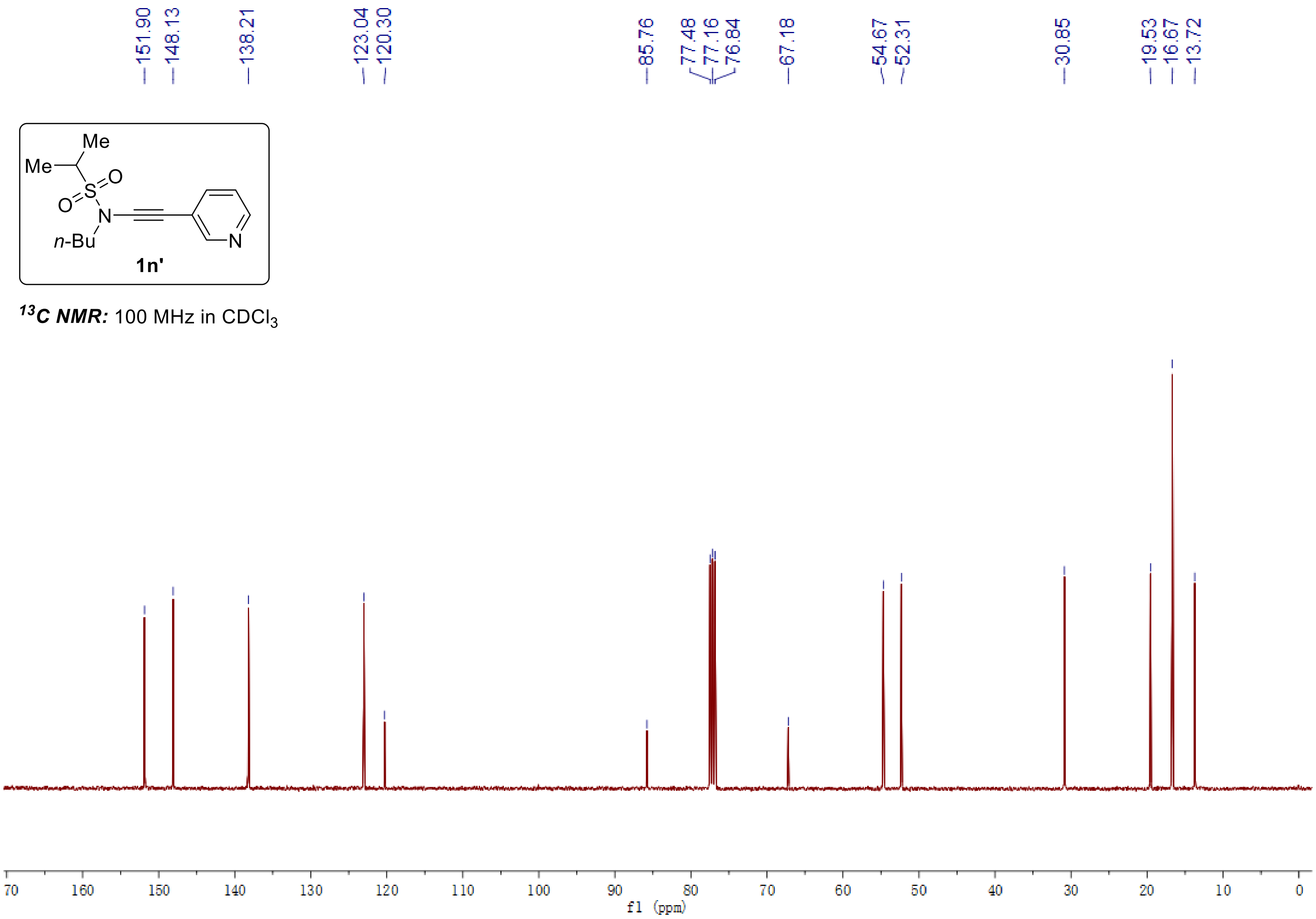
151.90
148.13
138.21
123.04
120.30
85.76
77.48
77.16
76.84
67.18
54.67
52.31
30.85
19.53
16.67
13.72
Me
Me
O
S
O
N
n-Bu
N
1n'
13C NMR: 100 MHz in CDCl3
170 160 150 140 130 120 110 100 90 80 70 60 50 40 30 20 10 0
f1 (ppm)

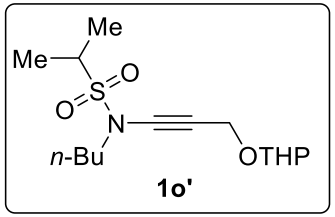

1H NMR: 400 MHz in $CDCl_3$

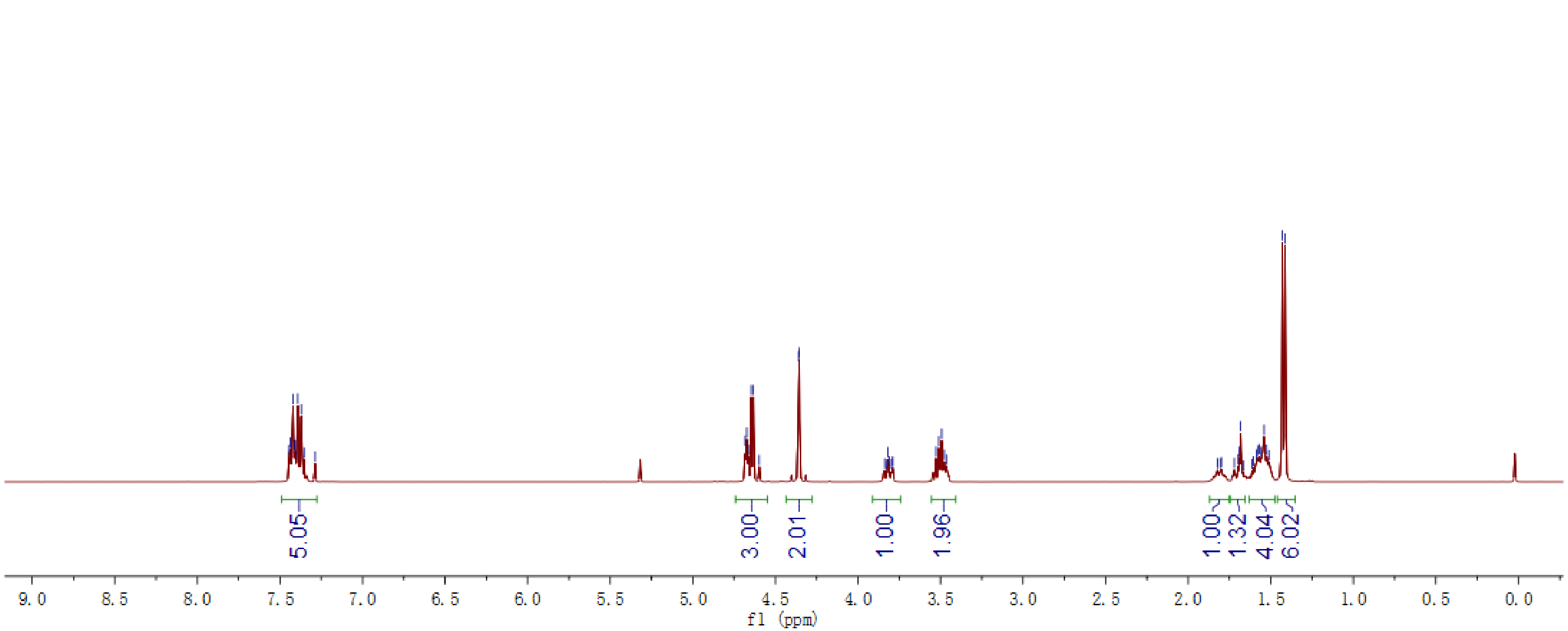

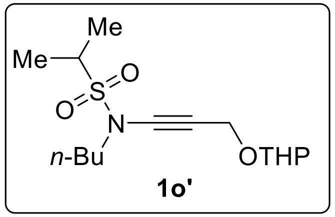

^{13}C NMR: 100 MHz in $CDCl_3$

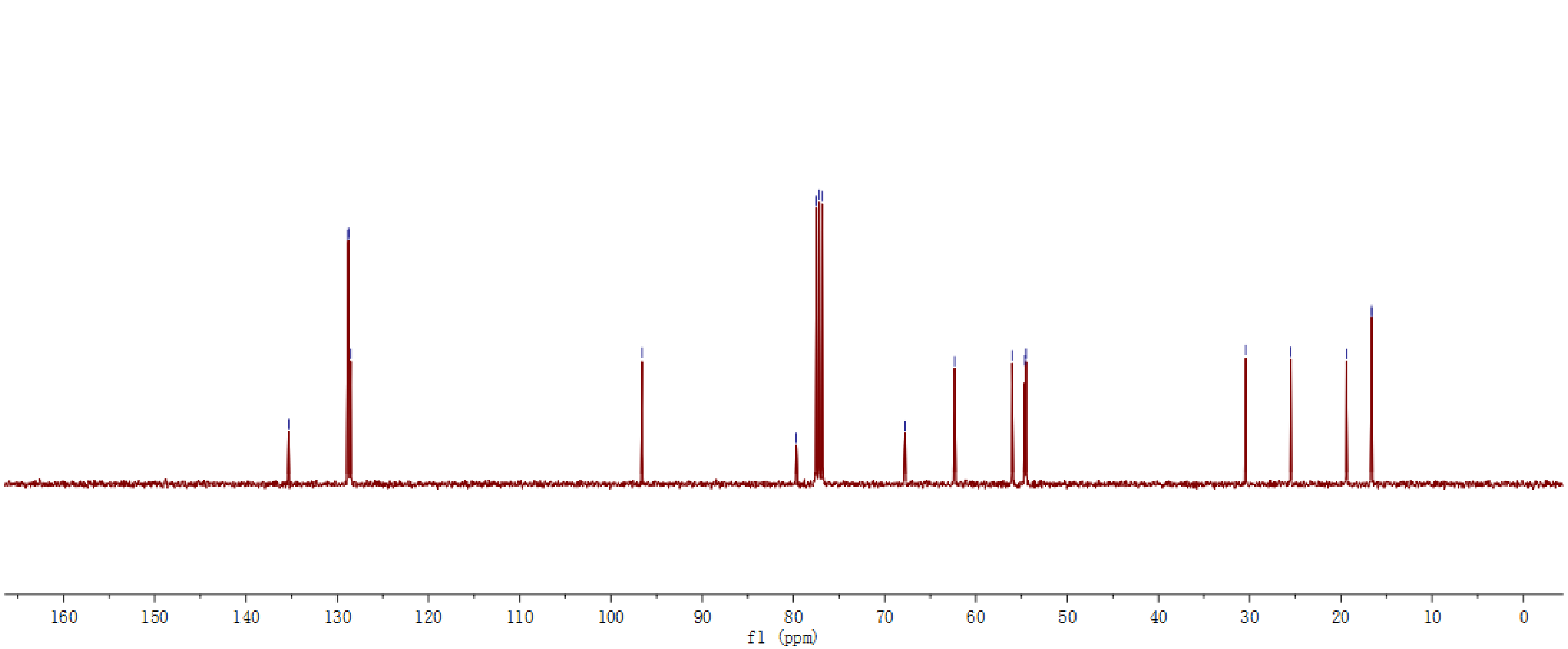

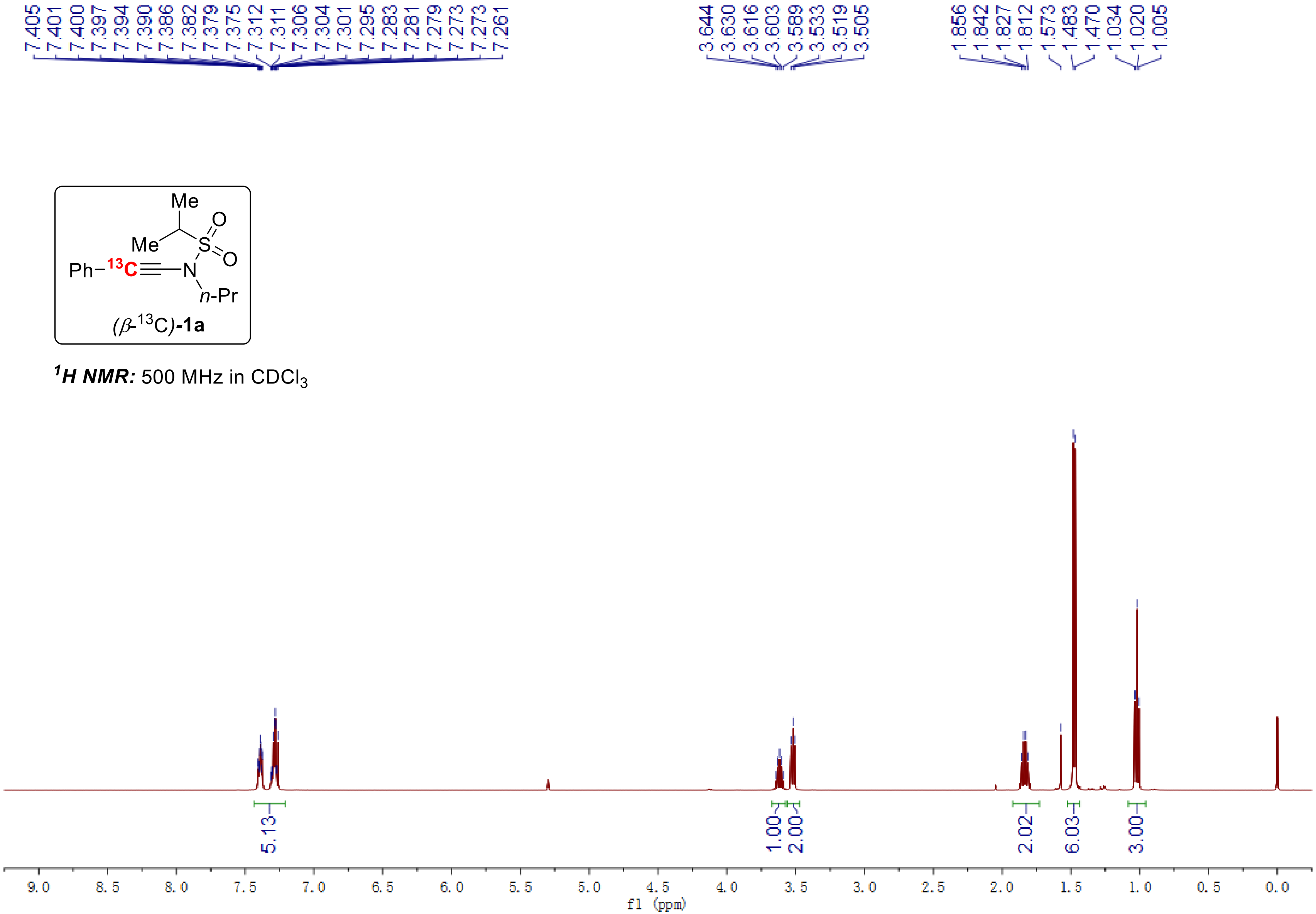

7.405
7.401
7.400
7.397
7.394
7.390
7.386
7.382
7.379
7.375
7.312
7.311
7.306
7.304
7.301
7.295
7.283
7.281
7.279
7.273
7.273
7.261
3.644
3.630
3.616
3.603
3.589
3.533
3.519
3.505
1.856
1.842
1.827
1.812
1.573
1.483
1.470
1.034
1.020
1.005
Me
O
Me
S
O
Ph
13C
N
n-Pr
(β-13C)-1a
1H NMR: 500 MHz in CDCl3
5.13
1.00
2.00
2.02
6.03
3.00
9.0
8.5
8.0
7.5
7.0
6.5
6.0
5.5
5.0
4.5
4.0
3.5
3.0
2.5
2.0
1.5
1.0
0.5
0.0
f1 (ppm)

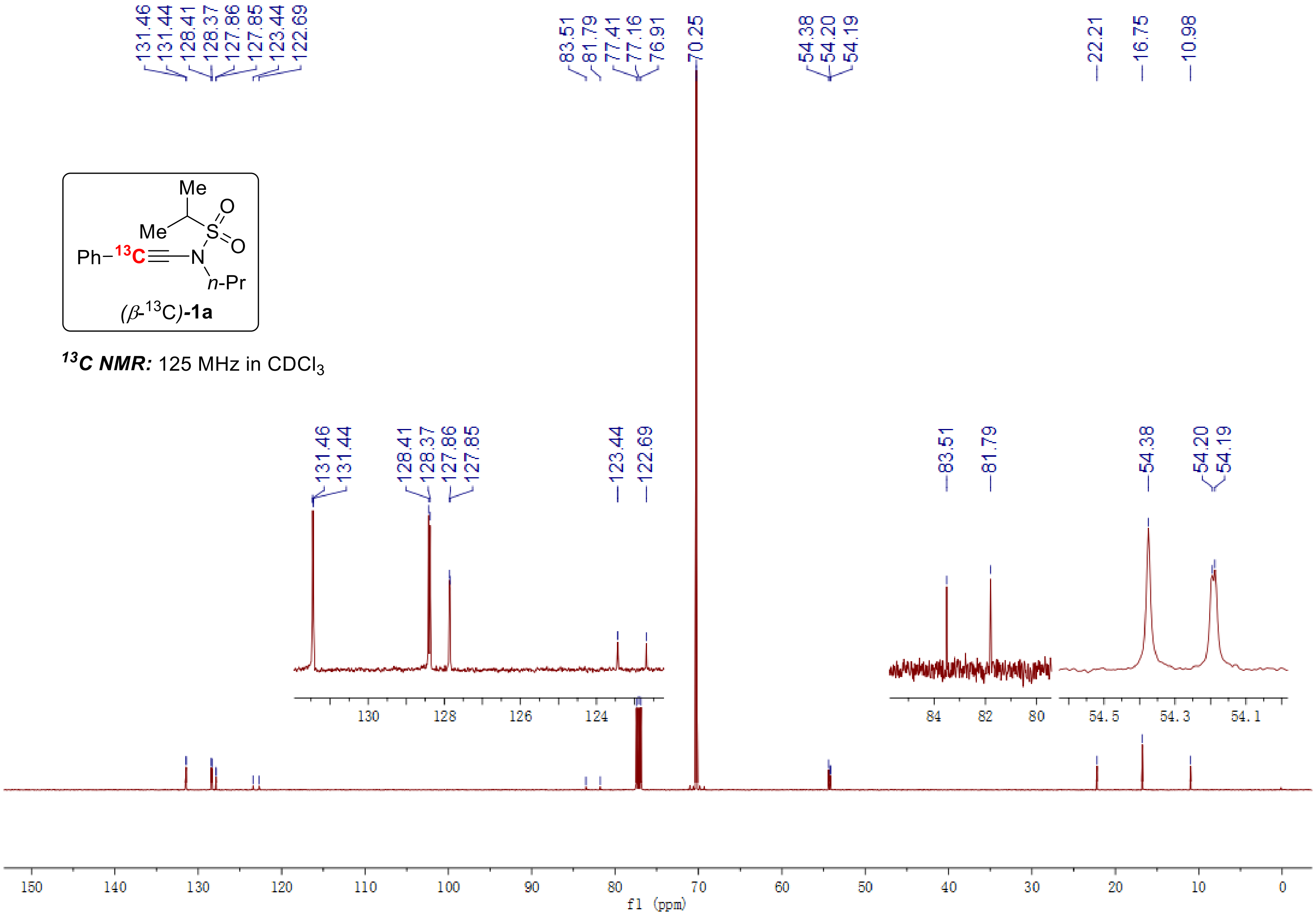
131.46
131.44
128.41
128.37
127.86
127.85
123.44
122.69
83.51
81.79
77.41
77.16
76.91
70.25
54.38
54.20
54.19
22.21
16.75
10.98
Me
Me
O
S
O
Ph−13C≡N
n-Pr
(β-13C)-1a
13C NMR: 125 MHz in CDCl3
f1 (ppm)

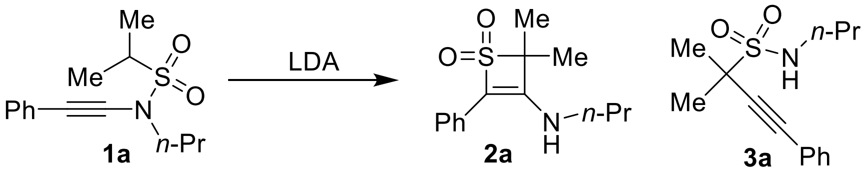

Crude ^{1}H NMR (500 MHz in $CDCl_3$): 2a: 3a = 5:1

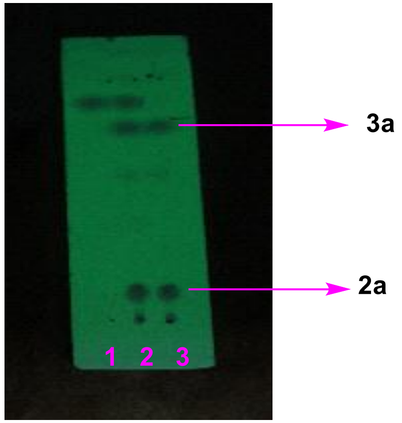

TLC: PE:EA = 3:1
Line 1: **1a**
Line 2: Mixture
Line 3: Reaction

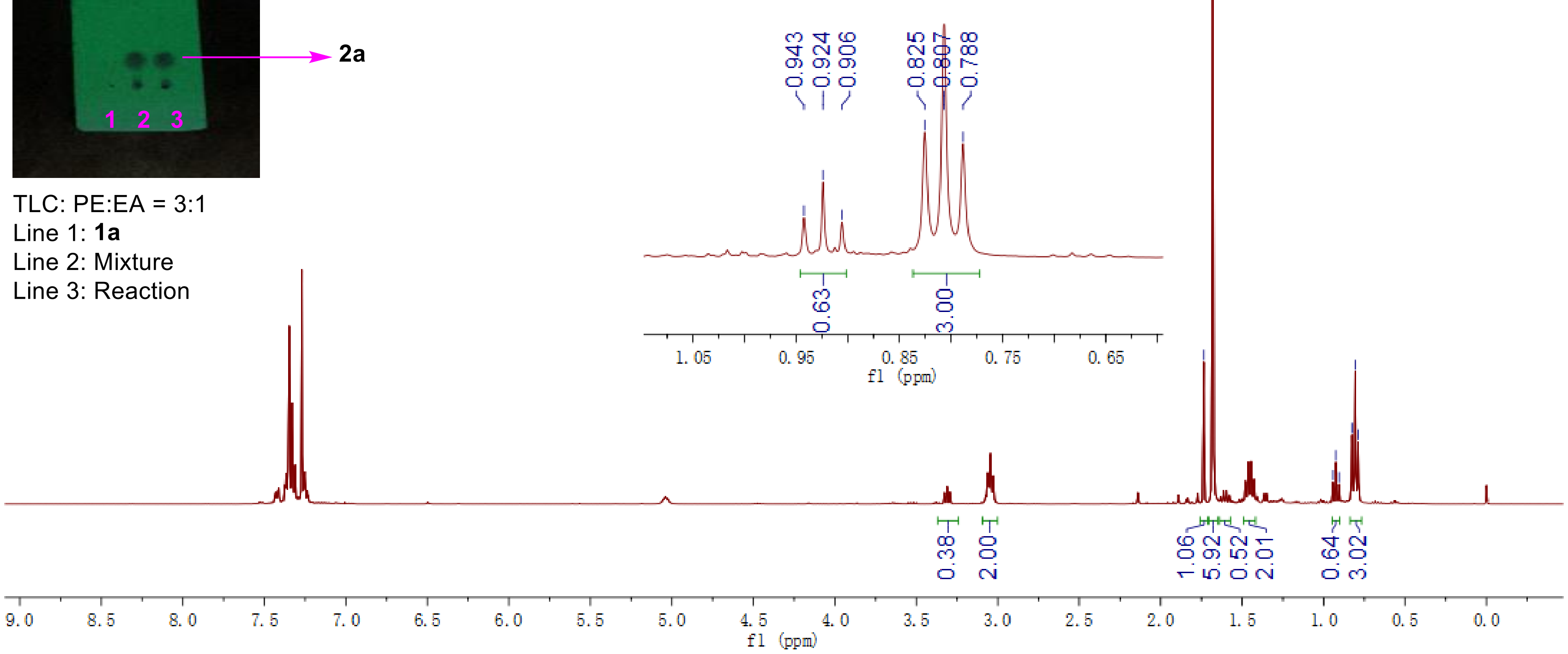

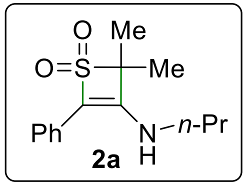

[1]*H NMR*: 400 MHz in CDCl3

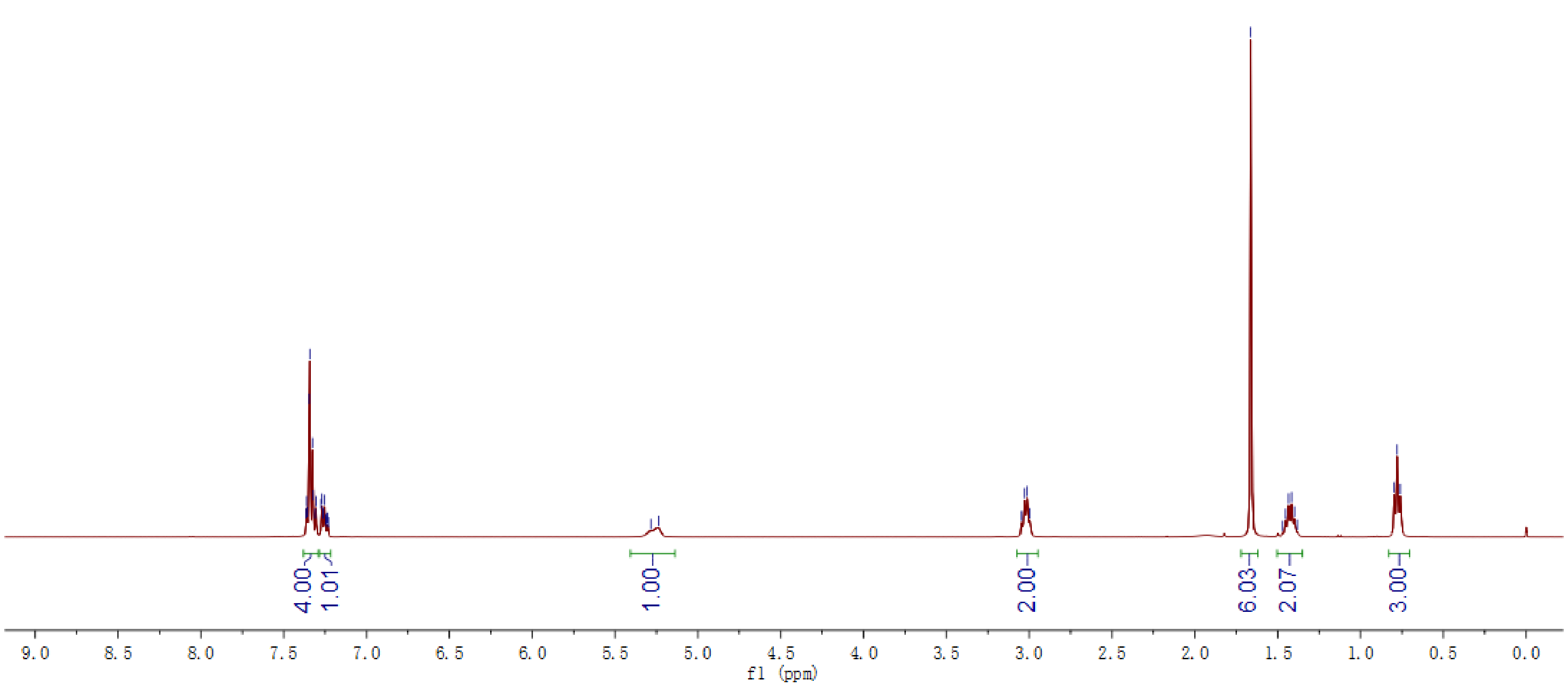

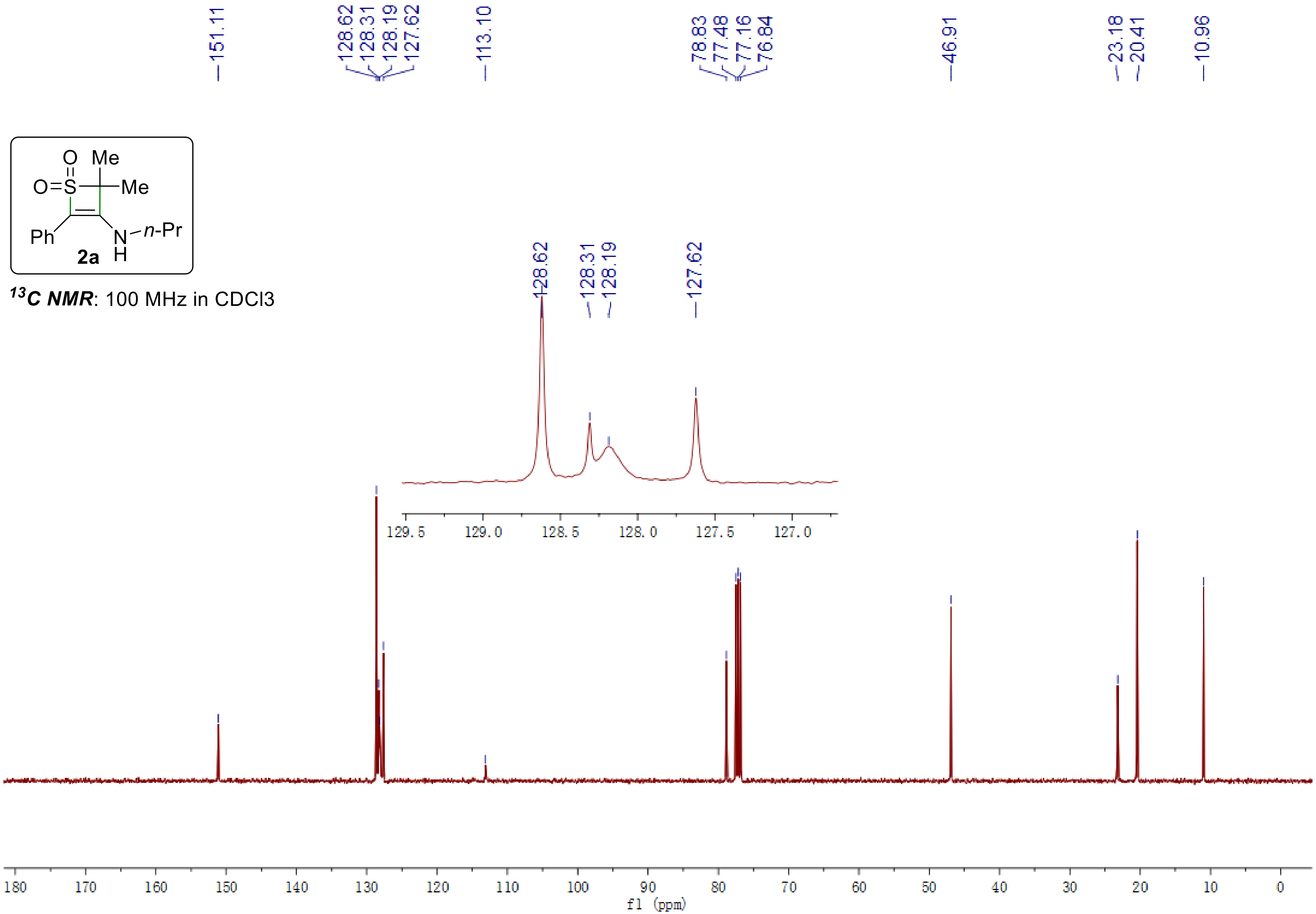
151.11
128.62
128.31
128.19
127.62
113.10
78.83
77.48
77.16
76.84
46.91
23.18
20.41
10.96
O
Me
O=S
Me
Ph
N
n-Pr
H
2a
13C NMR: 100 MHz in CDCl3
128.62
128.31
128.19
127.62
129.5
129.0
128.5
128.0
127.5
127.0
180
170
160
150
140
130
120
110
100
90
80
70
60
50
40
30
20
10
0
f1 (ppm)

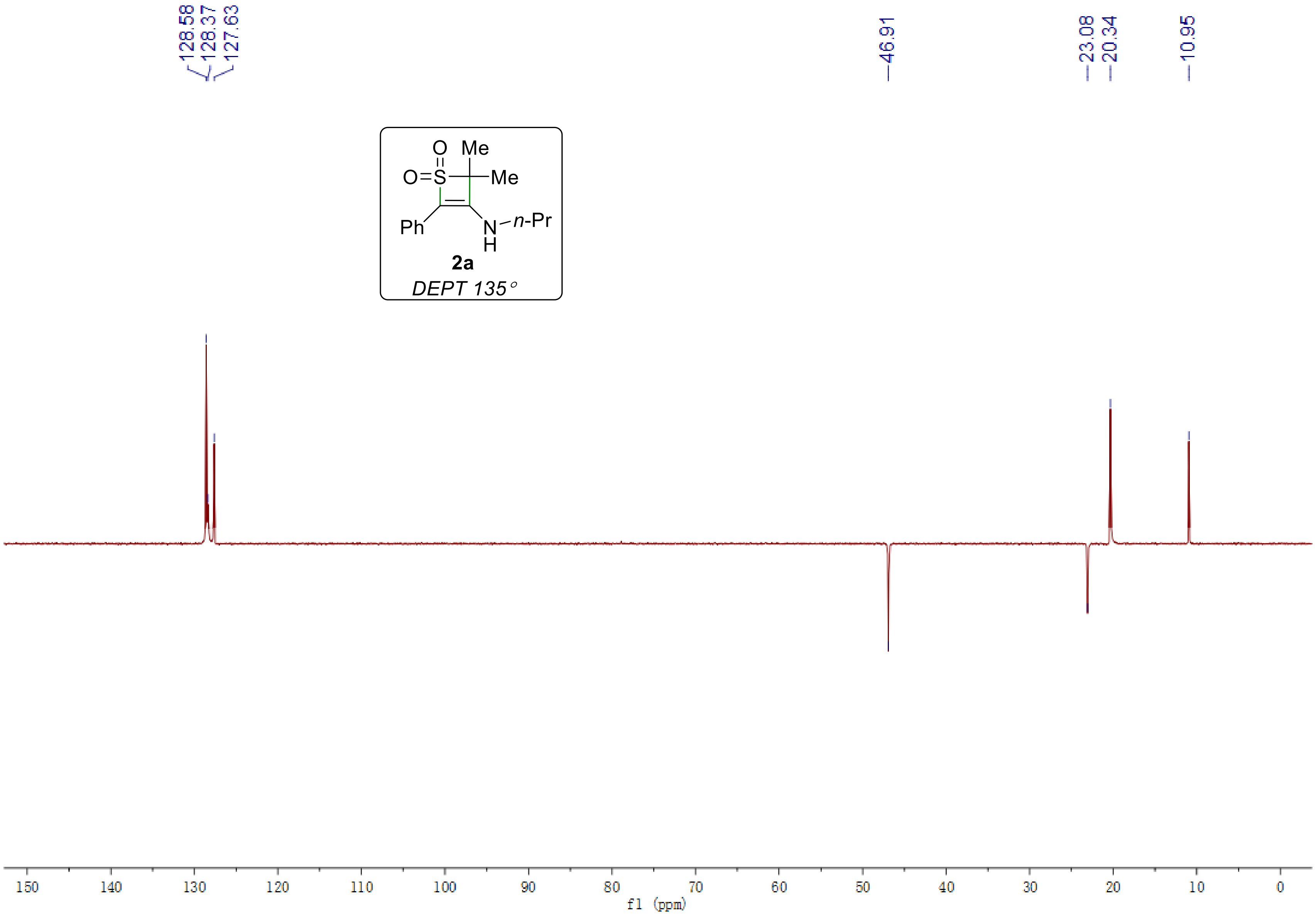
128.58
128.37
127.63
46.91
23.08
20.34
10.95
O
Me
O=S
Me
Ph
N
n-Pr
H
2a
DEPT 135°
150
140
130
120
110
100
90
80
70
60
50
40
30
20
10
0
f1 (ppm)

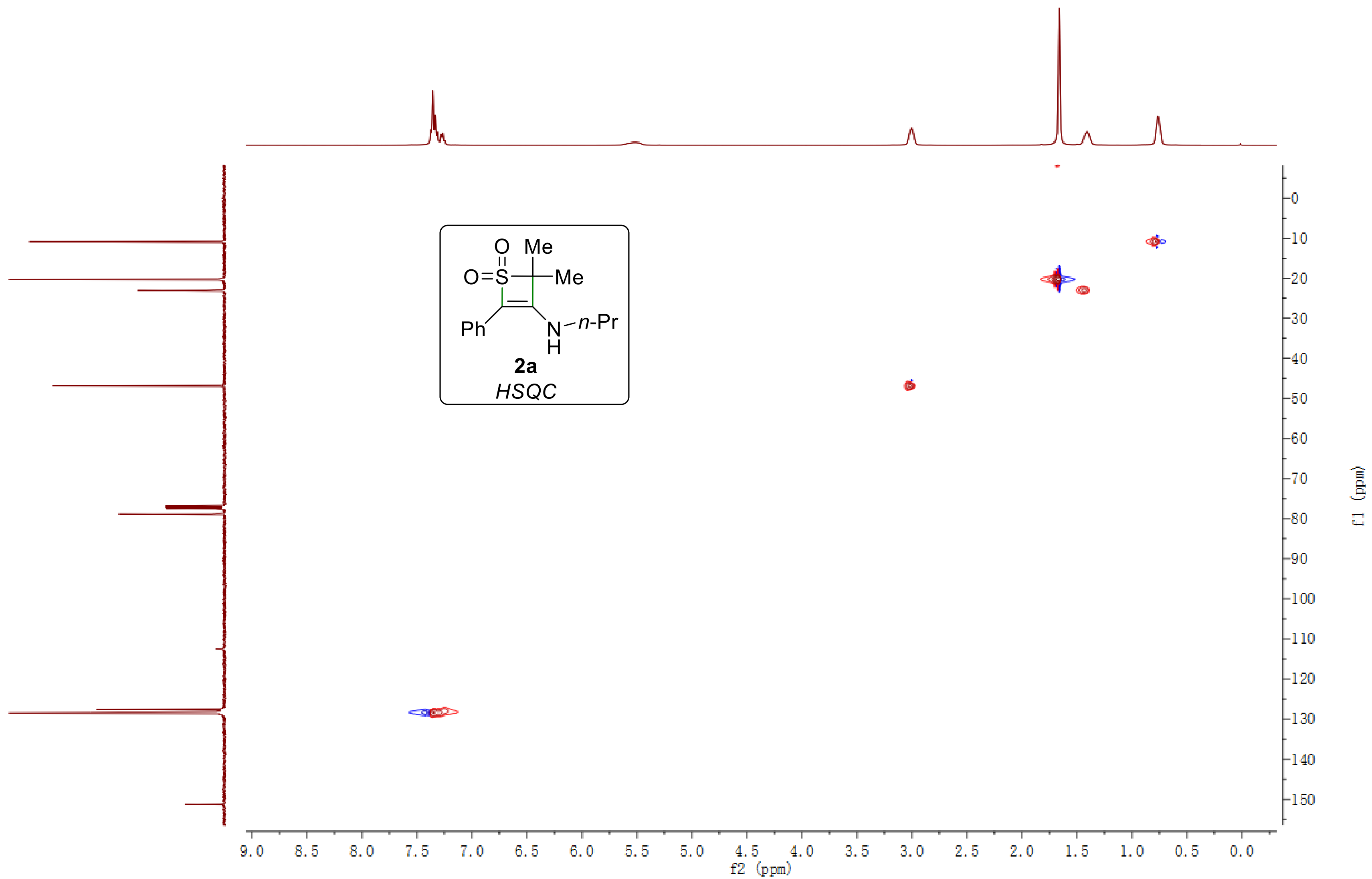

O
Me
O=S
Me
Ph
N
n-Pr
H
2a
HSQC
9.0 8.5 8.0 7.5 7.0 6.5 6.0 5.5 5.0 4.5 4.0 3.5 3.0 2.5 2.0 1.5 1.0 0.5 0.0
f2 (ppm)
0 10 20 30 40 50 60 70 80 90 100 110 120 130 140 150
f1 (ppm)

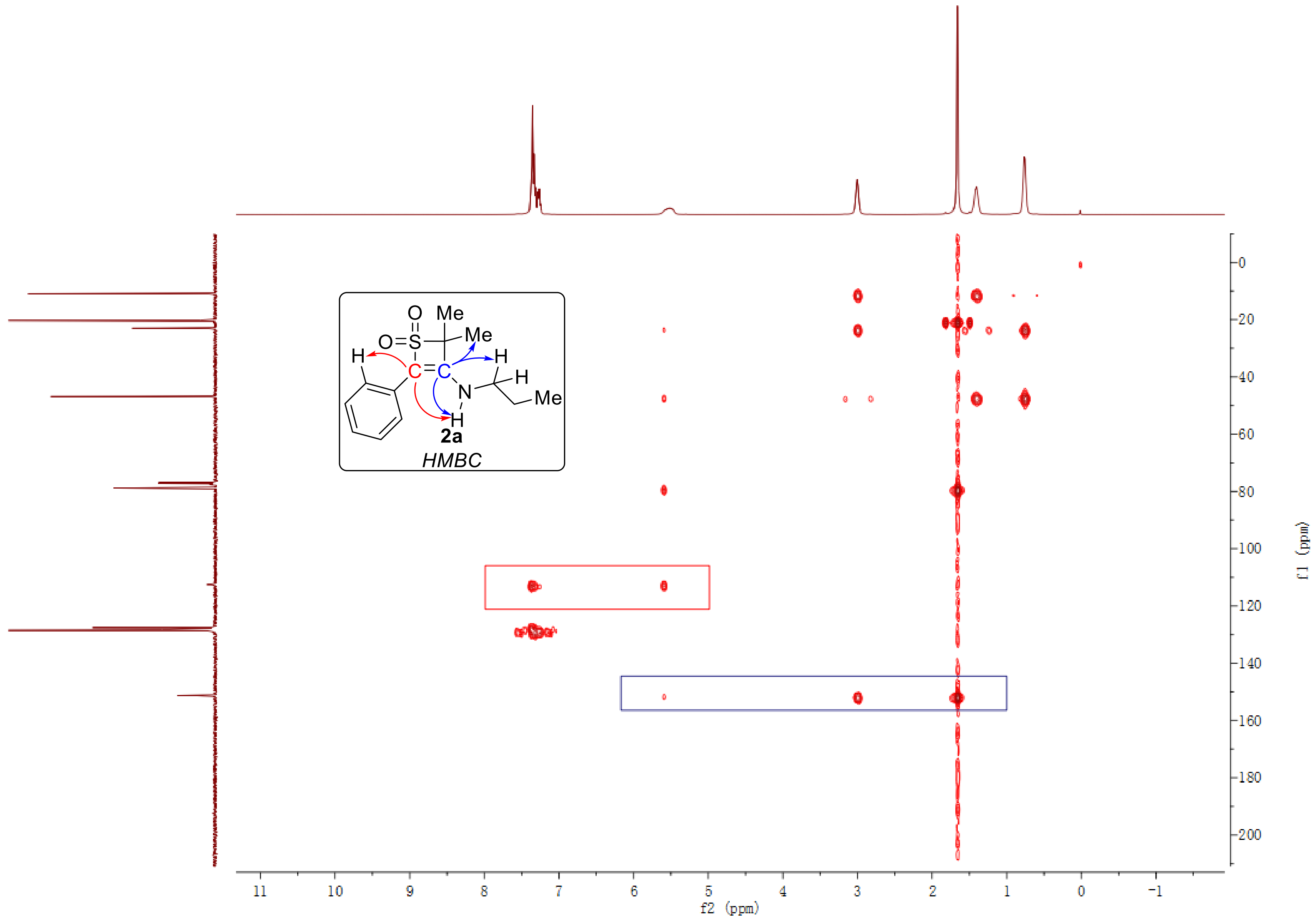

O
Me
O=S
Me
H
C=C
H
N
H
Me
H
2a
HMBC
f2 (ppm)
f1 (ppm)

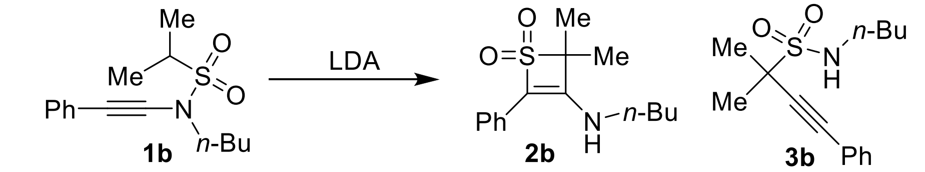

Crude ^{1}H NMR (500 MHz in $CDCl_3$): 2b: 3b = 4:1

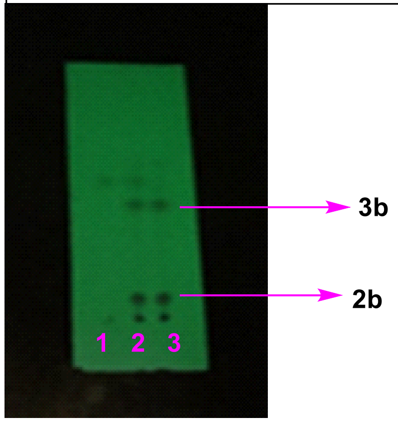

TLC: PE:EA = 3:1
Line 1: **1b**
Line 2: Mixture
Line 3: Reaction

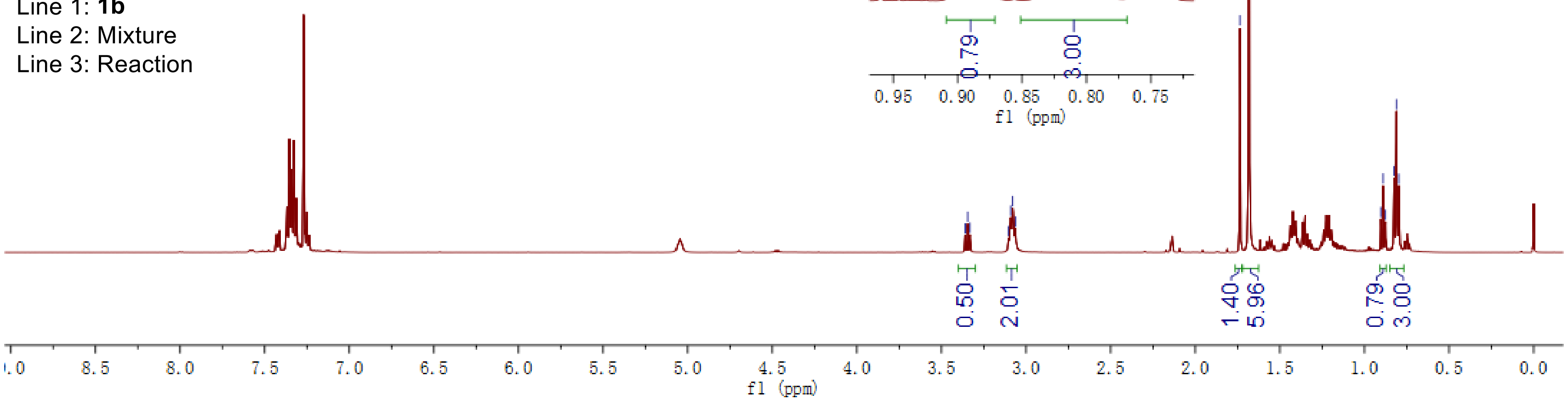

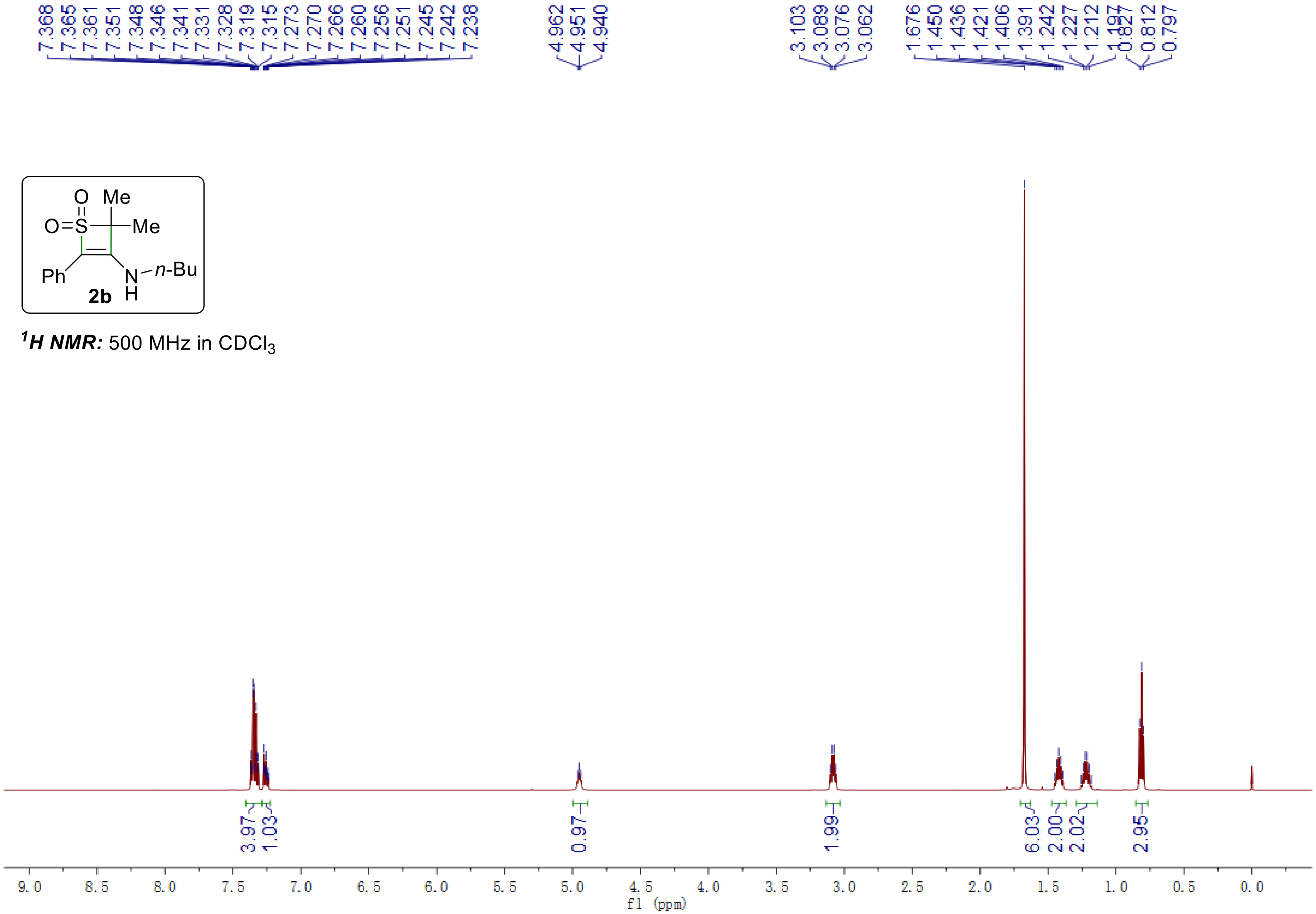

7.368
7.365
7.361
7.351
7.348
7.346
7.341
7.331
7.328
7.319
7.315
7.273
7.270
7.266
7.260
7.256
7.251
7.245
7.242
7.238
4.962
4.951
4.940
3.103
3.089
3.076
3.062
1.676
1.450
1.436
1.421
1.406
1.391
1.242
1.227
1.212
1.197
0.827
0.812
0.797
2b
1H NMR: 500 MHz in CDCl3
3.97
1.03
0.97
1.99
6.03
2.00
2.02
2.95
9.0
8.5
8.0
7.5
7.0
6.5
6.0
5.5
5.0
4.5
4.0
3.5
3.0
2.5
2.0
1.5
1.0
0.5
0.0
f1 (ppm)

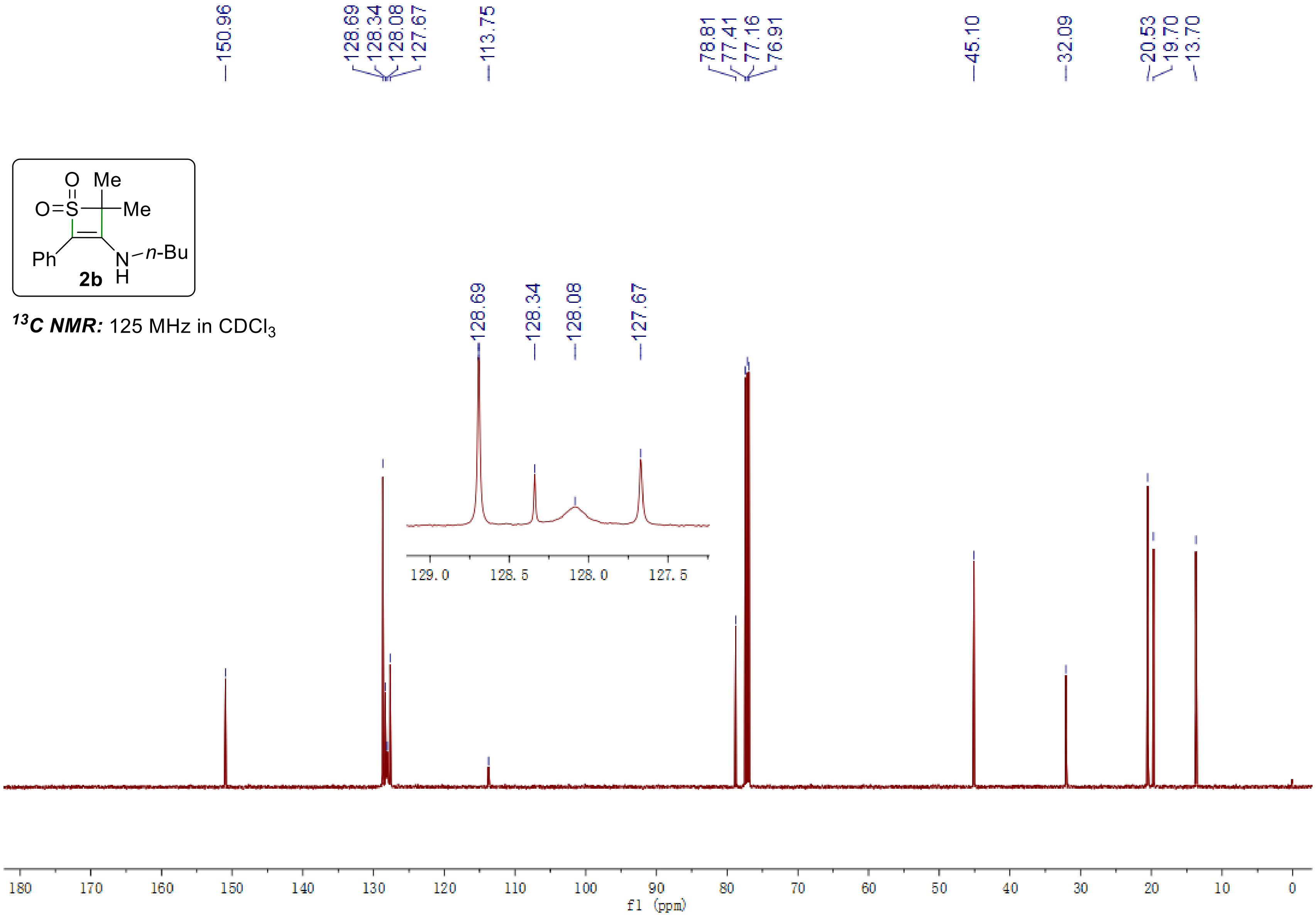
150.96
128.69
128.34
128.08
127.67
113.75
78.81
77.41
77.16
76.91
45.10
32.09
20.53
19.70
13.70
O
Me
O=S
Me
Ph
N
n-Bu
H
2b
13C NMR: 125 MHz in CDCl3
129.0
128.5
128.0
127.5
180
170
160
150
140
130
120
110
100
90
80
70
60
50
40
30
20
10
0
f1 (ppm)

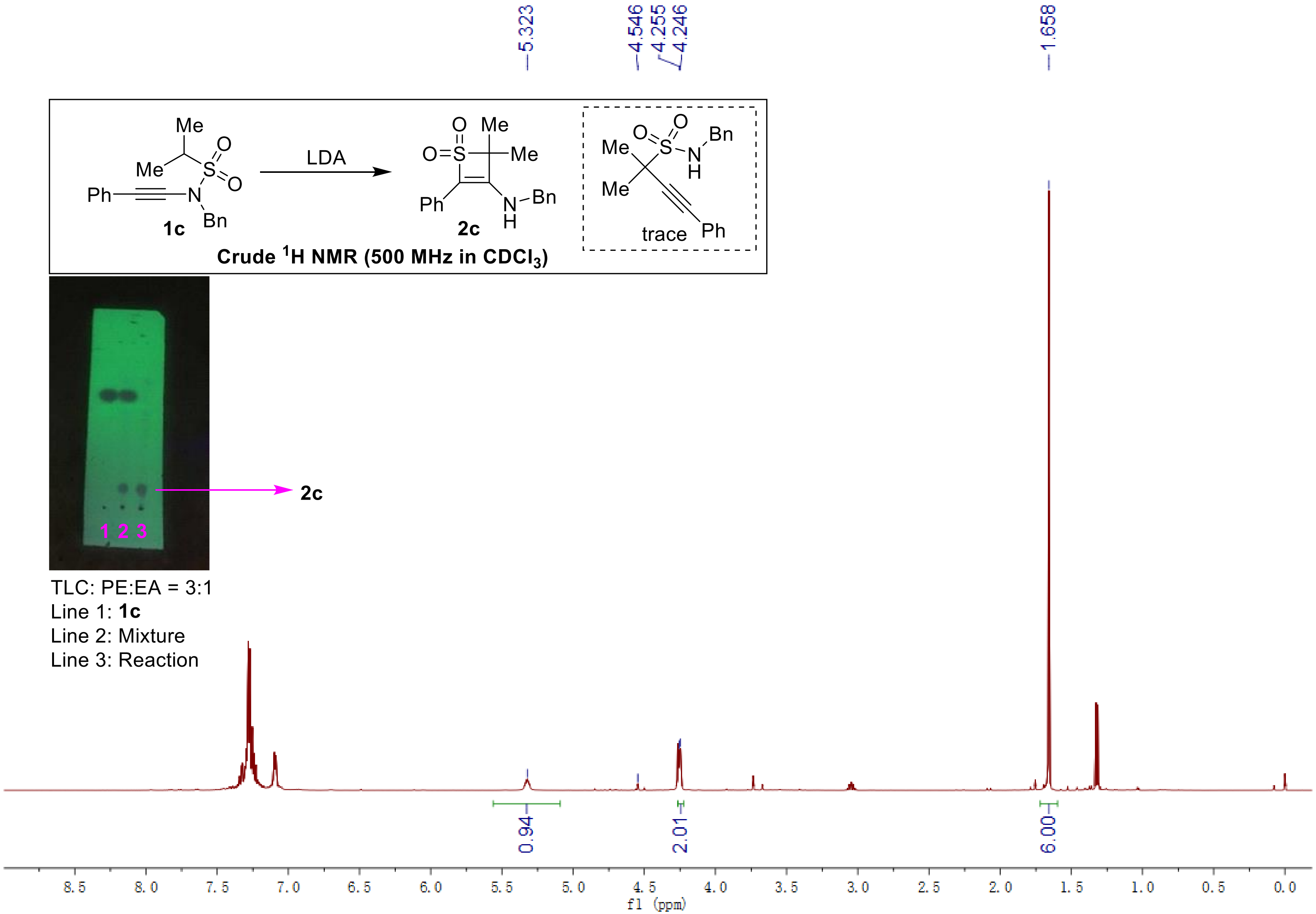

5.323
4.546
4.255
4.246
1.658
Me
O
S
O
Me
Ph
N
1c
Bn
LDA
O
Me
O=S
Me
Ph
N-Bn
H
2c
Me
O
S
O
Bn
N
H
Me
trace
Ph
Crude 1H NMR (500 MHz in CDCl3)
2c
1 2 3
TLC: PE:EA = 3:1
Line 1: 1c
Line 2: Mixture
Line 3: Reaction
0.94
2.01
6.00
8.5
8.0
7.5
7.0
6.5
6.0
5.5
5.0
4.5
4.0
3.5
3.0
2.5
2.0
1.5
1.0
0.5
0.0
f1 (ppm)

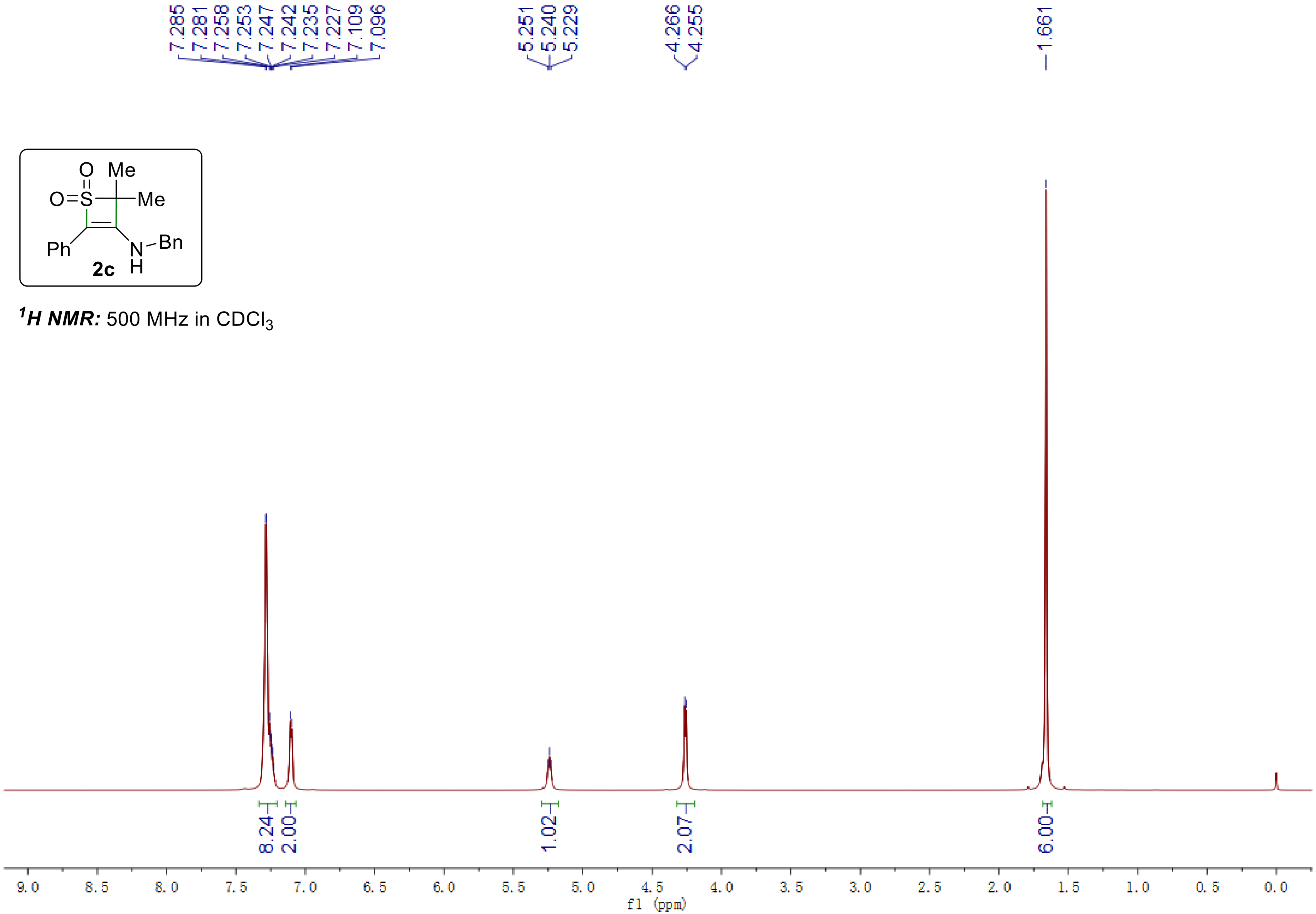
7.285
7.281
7.258
7.253
7.247
7.242
7.235
7.227
7.109
7.096
5.251
5.240
5.229
4.266
4.255
1.661
O
O=S
Me
Me
Ph
N–Bn
H
2c
¹H NMR: 500 MHz in $CDCl_3$
8.24
2.00
1.02
2.07
6.00
9.0
8.5
8.0
7.5
7.0
6.5
6.0
5.5
5.0
4.5
4.0
3.5
3.0
2.5
2.0
1.5
1.0
0.5
0.0
f1 (ppm)

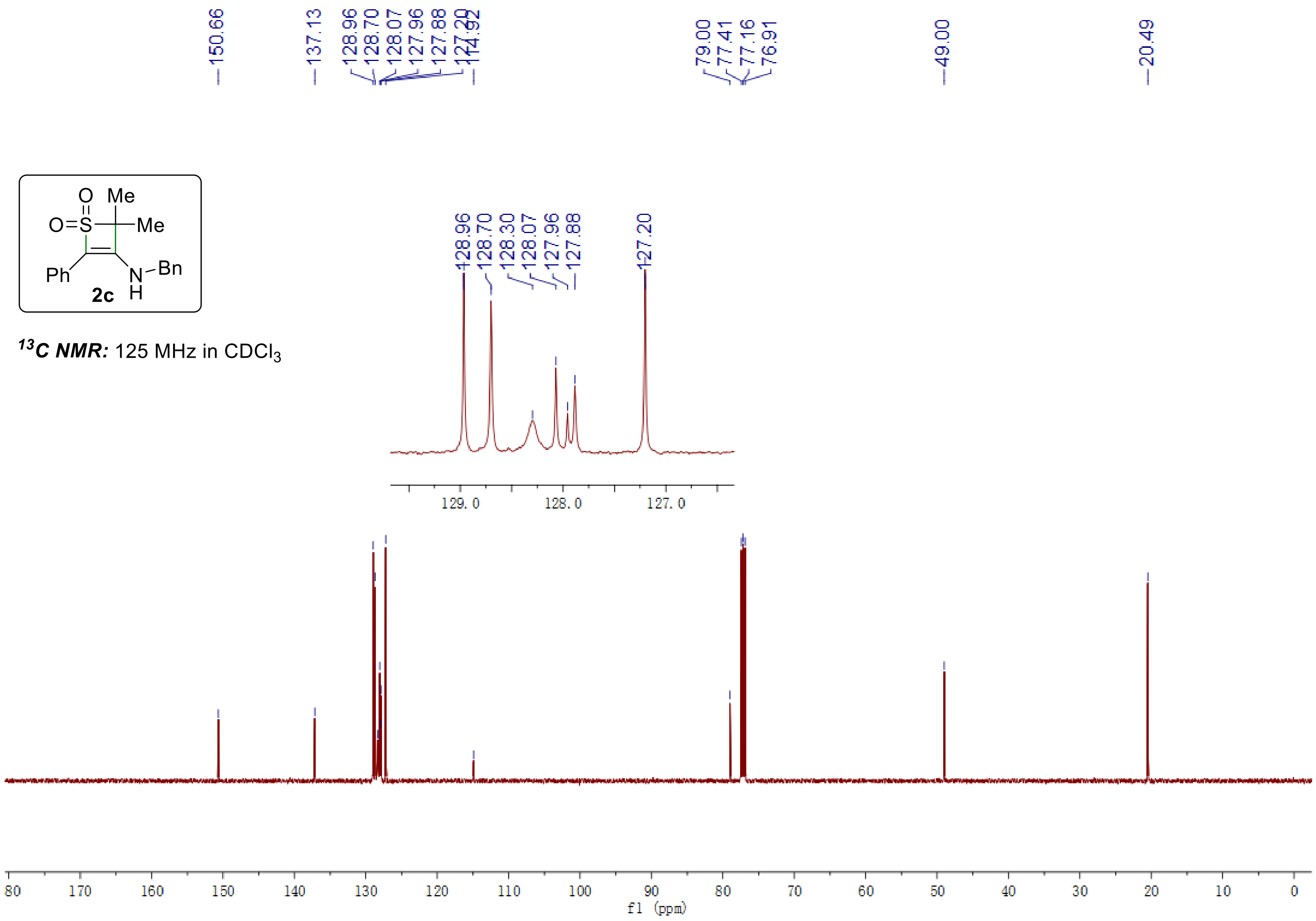

2c

^{13}C NMR: 125 MHz in $CDCl_3$

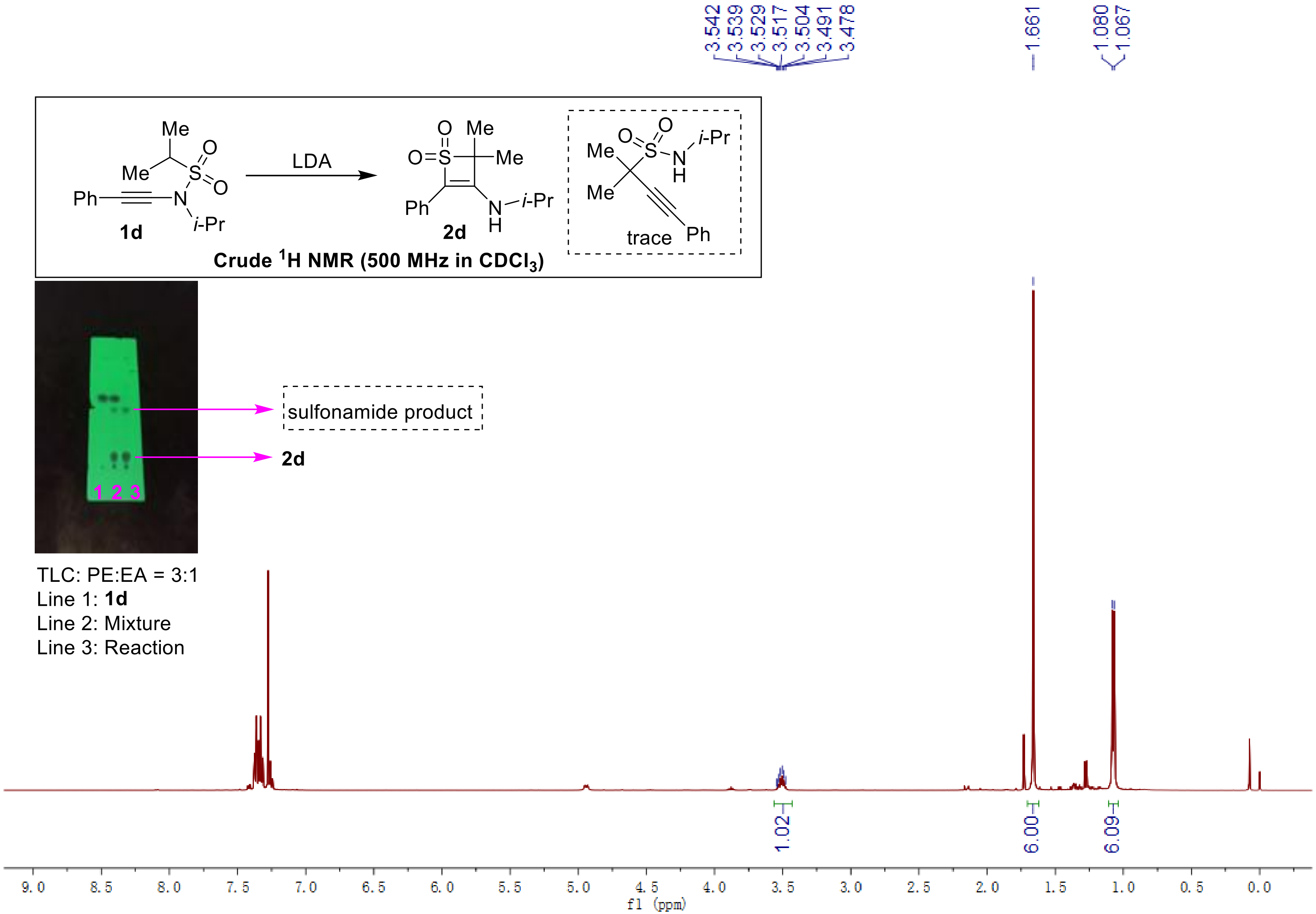
3.542
3.539
3.529
3.517
3.504
3.491
3.478
1.661
1.080
1.067
Me
Me
Ph
1d
i-Pr
LDA
O
Me
O=S
Me
Ph
N
i-Pr
H
2d
Me
O
O
S
N
H
i-Pr
Me
trace
Ph
Crude ¹H NMR (500 MHz in CDCl3)
sulfonamide product
2d
1 2 3
TLC: PE:EA = 3:1
Line 1: 1d
Line 2: Mixture
Line 3: Reaction
1.02
6.00
6.09
9.0
8.5
8.0
7.5
7.0
6.5
6.0
5.5
5.0
4.5
4.0
3.5
3.0
2.5
2.0
1.5
1.0
0.5
0.0
f1 (ppm)

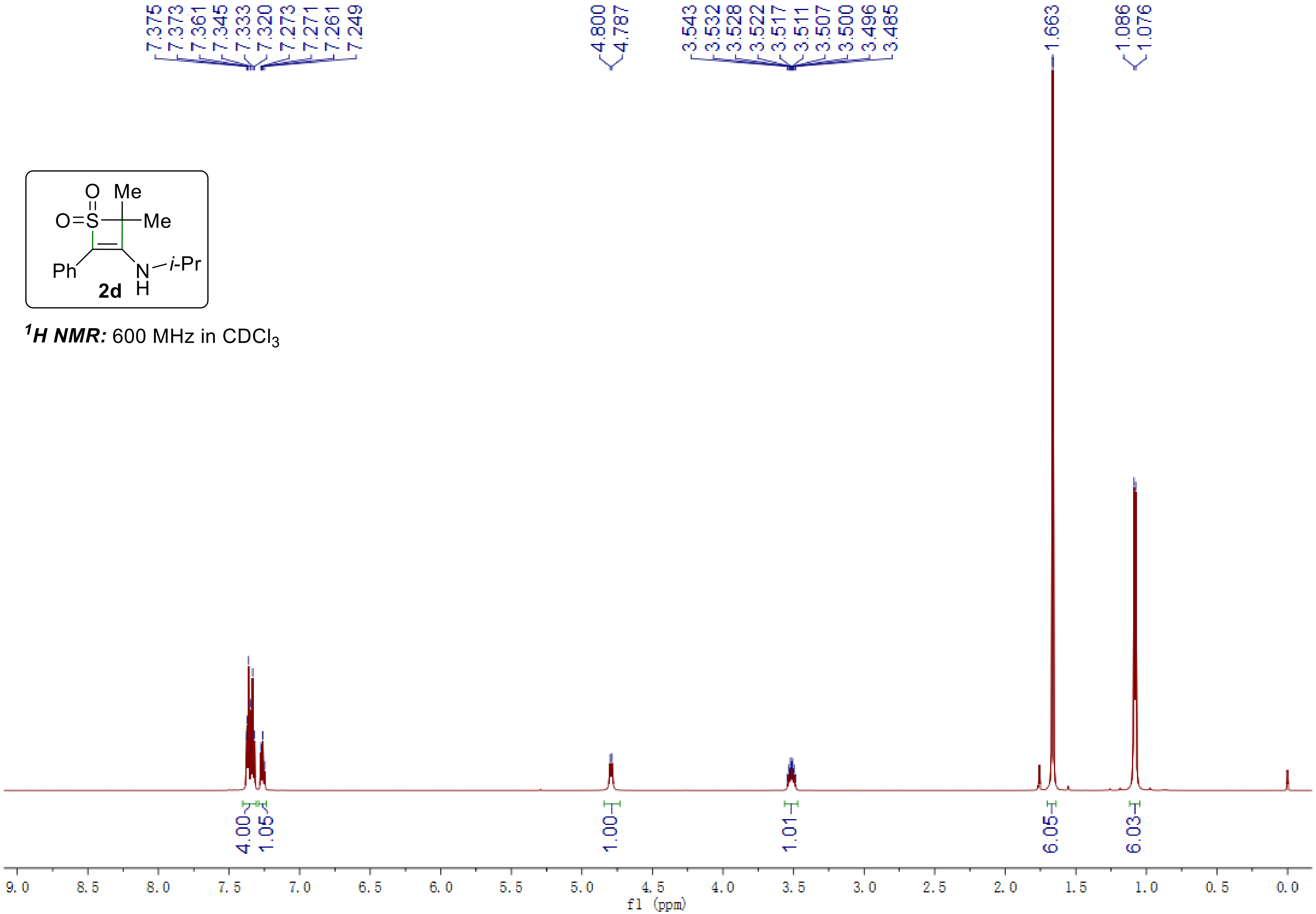
7.375
7.373
7.361
7.345
7.333
7.320
7.273
7.271
7.261
7.249
4.800
4.787
3.543
3.532
3.528
3.522
3.517
3.511
3.507
3.500
3.496
3.485
1.663
1.086
1.076
O
Me
O=S
Me
Ph
N
i-Pr
H
2d
1H NMR: 600 MHz in CDCl3
4.00
1.05
1.00
1.01
6.05
6.03
9.0 8.5 8.0 7.5 7.0 6.5 6.0 5.5 5.0 4.5 4.0 3.5 3.0 2.5 2.0 1.5 1.0 0.5 0.0
f1 (ppm)

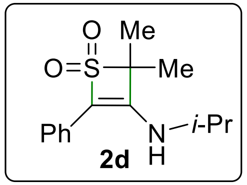

^{13}**C NMR:** 125 MHz in $CDCl_3$

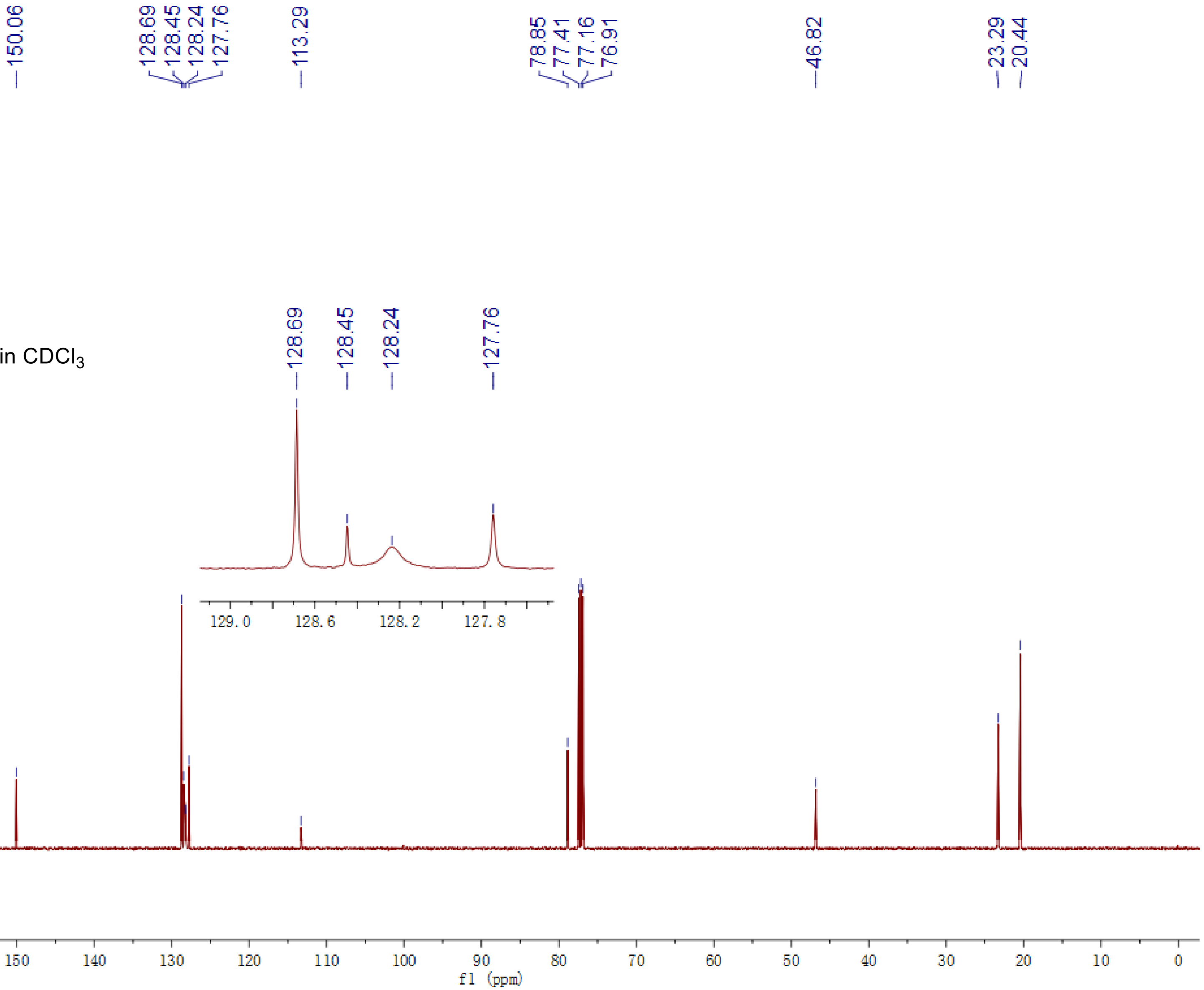

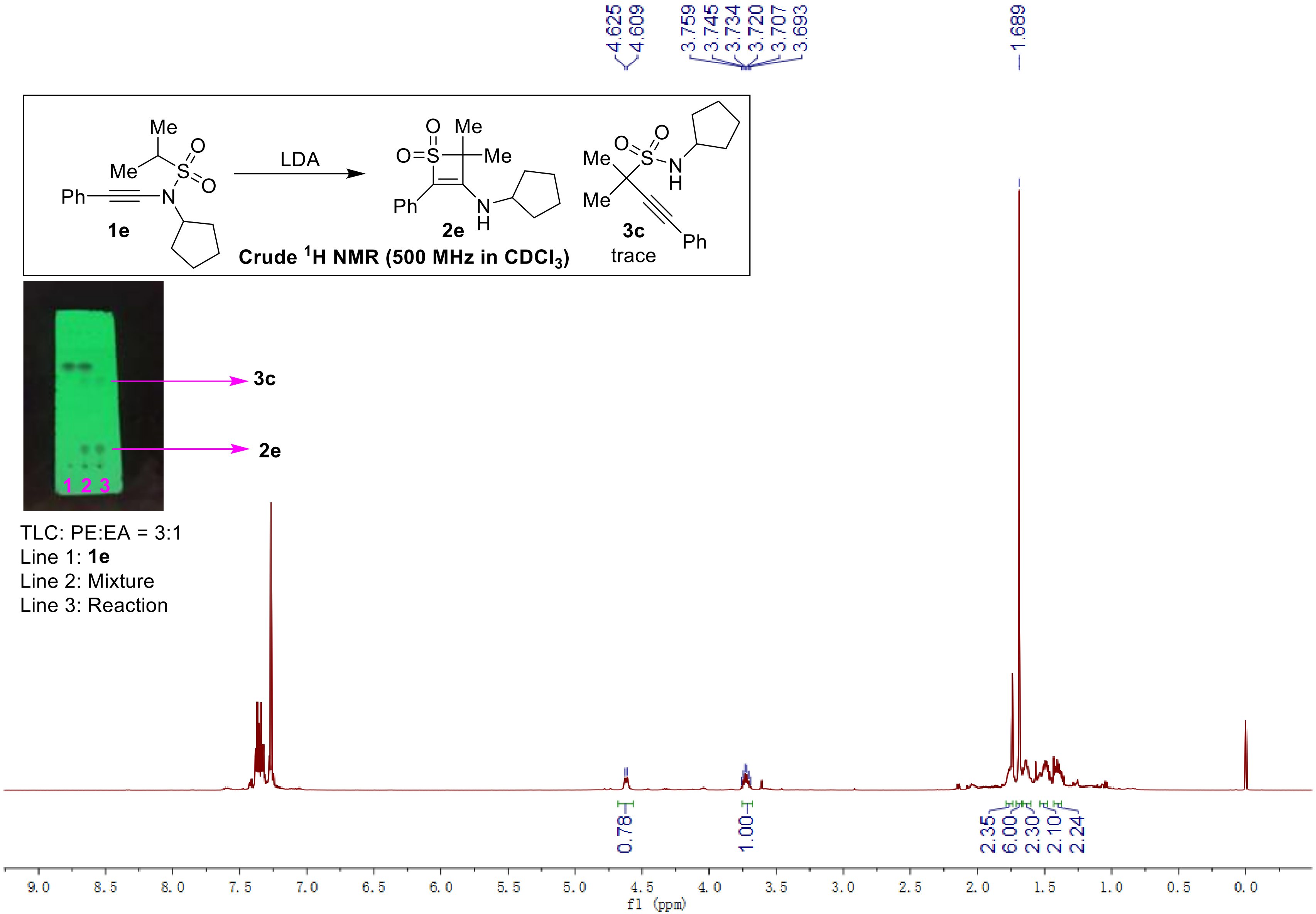
4.625
4.609
3.759
3.745
3.734
3.720
3.707
3.693
1.689
Me
Me
O
S
O
Ph
N
1e
LDA
O Me
O=S
Me
Ph
N
H
2e
Crude 1H NMR (500 MHz in CDCl3)
Me
O
S
O
N
H
Me
3c
trace
Ph
1 2 3
3c
2e
TLC: PE:EA = 3:1
Line 1: 1e
Line 2: Mixture
Line 3: Reaction
0.78
1.00
2.35
6.00
2.30
2.10
2.24
9.0
8.5
8.0
7.5
7.0
6.5
6.0
5.5
5.0
4.5
4.0
3.5
3.0
2.5
2.0
1.5
1.0
0.5
0.0
f1 (ppm)

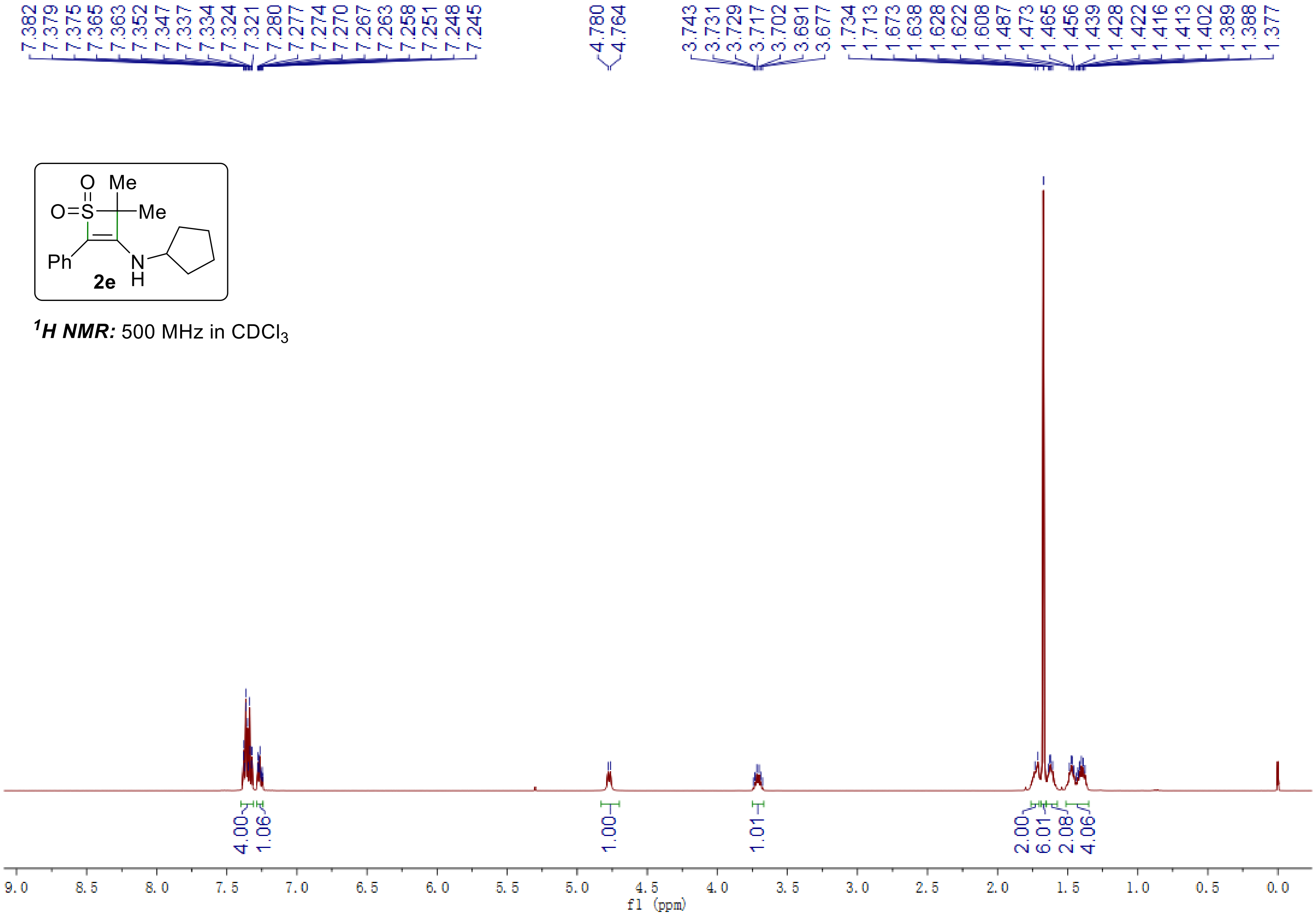
7.382 7.379 7.375 7.365 7.363 7.352 7.347 7.337 7.334 7.324 7.321 7.280 7.277 7.274 7.270 7.267 7.263 7.258 7.251 7.248 7.245
4.780 4.764
3.743 3.731 3.729 3.717 3.702 3.691 3.677
1.734 1.713 1.673 1.638 1.628 1.622 1.608 1.487 1.473 1.465 1.456 1.439 1.428 1.422 1.416 1.413 1.402 1.389 1.388 1.377
O
Me
O=S
Me
Ph
N
H
2e
1H NMR: 500 MHz in CDCl3
4.00
1.06
1.00
1.01
2.00
6.01
2.08
4.06
9.0 8.5 8.0 7.5 7.0 6.5 6.0 5.5 5.0 4.5 4.0 3.5 3.0 2.5 2.0 1.5 1.0 0.5 0.0
f1 (ppm)

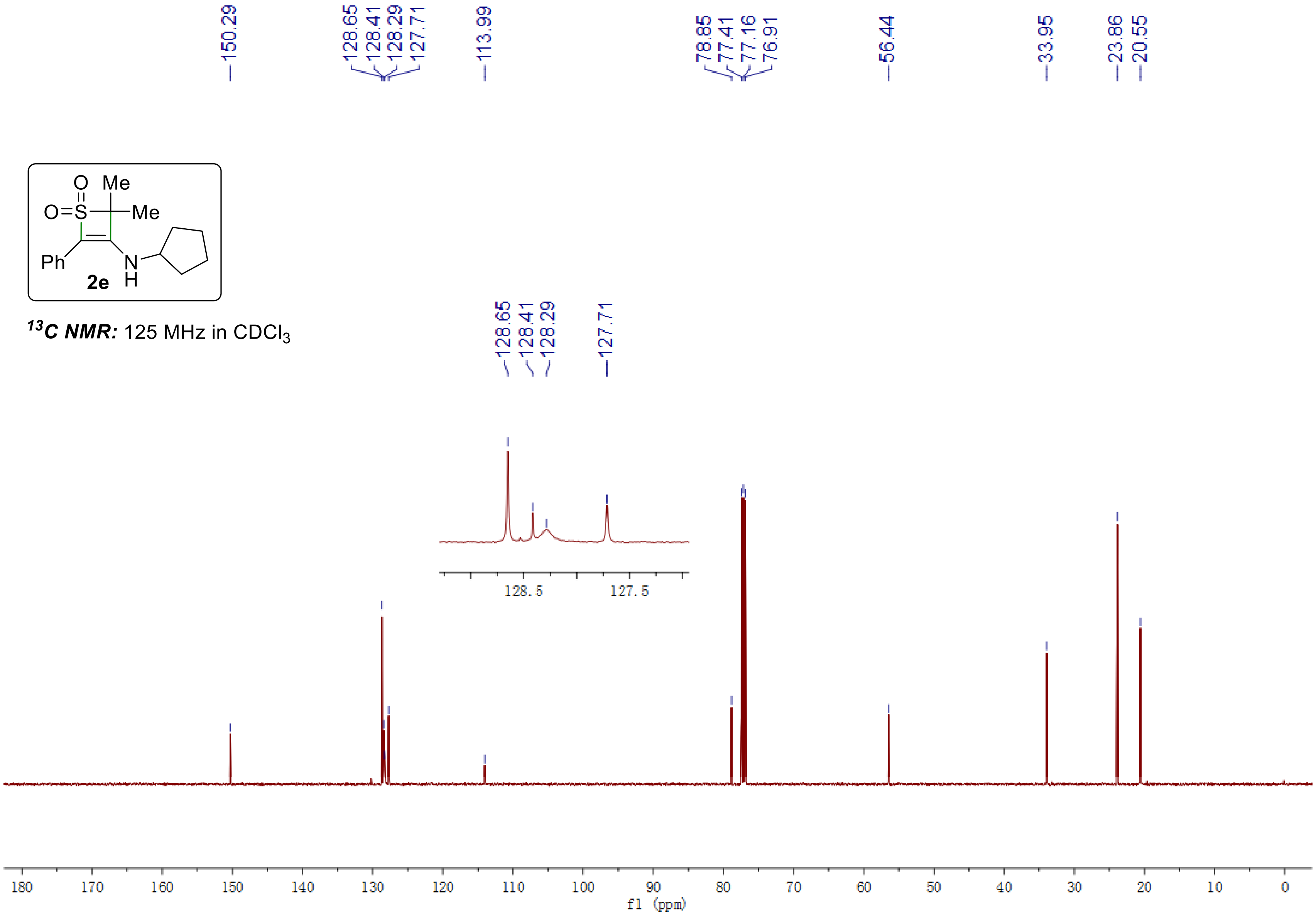
150.29
128.65
128.41
128.29
127.71
113.99
78.85
77.41
77.16
76.91
56.44
33.95
23.86
20.55
O
Me
O=S
Me
Ph
N
H
2e
13C NMR: 125 MHz in CDCl3
128.65
128.41
128.29
127.71
128.5
127.5
180
170
160
150
140
130
120
110
100
90
80
70
60
50
40
30
20
10
0
f1 (ppm)

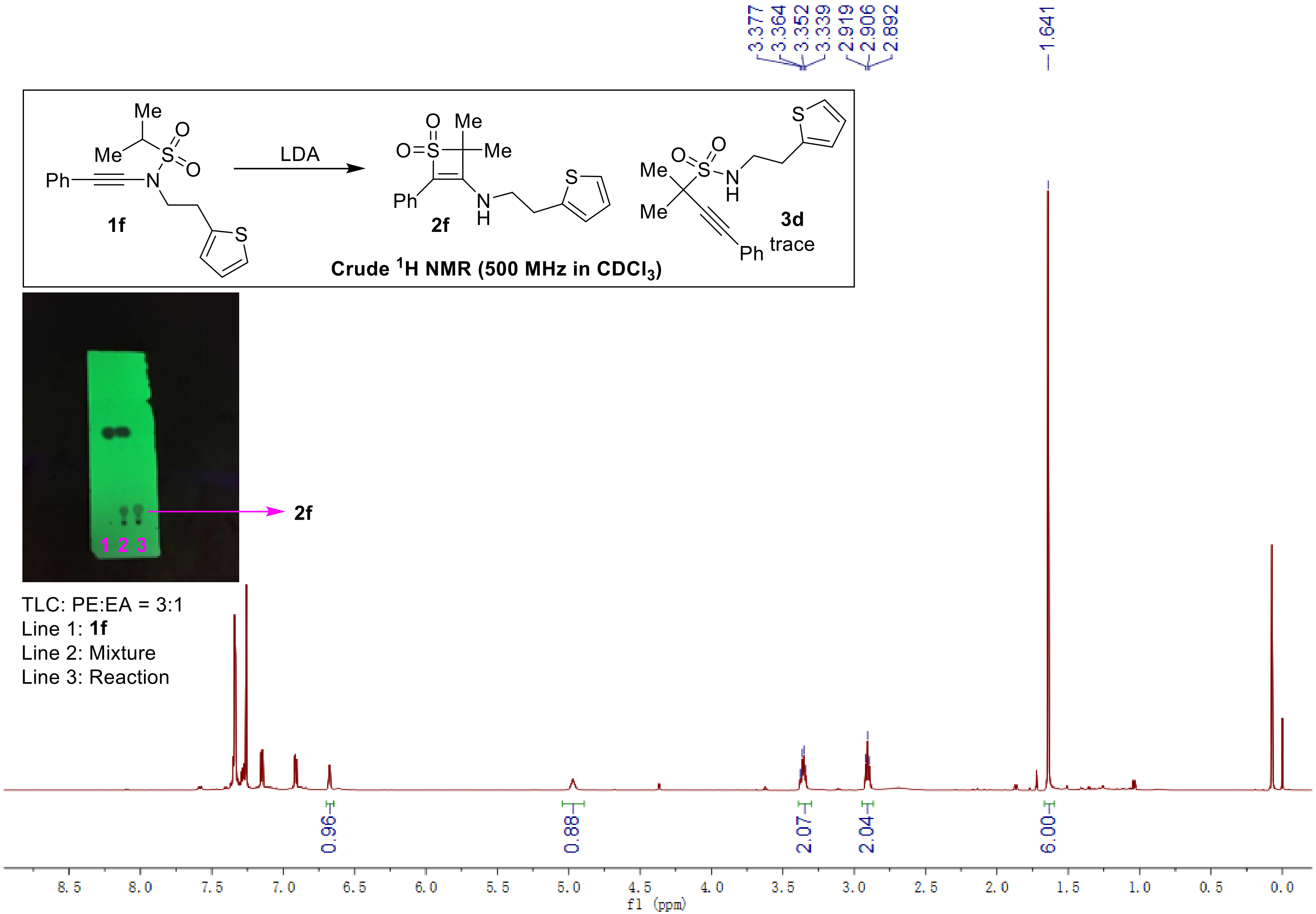

1f
LDA
2f
3d
trace
Crude ¹H NMR (500 MHz in CDCl₃)
1 2 3
2f
TLC: PE:EA = 3:1
Line 1: 1f
Line 2: Mixture
Line 3: Reaction
3.377
3.364
3.352
3.339
2.919
2.906
2.892
1.641
0.96
0.88
2.07
2.04
6.00
8.5
8.0
7.5
7.0
6.5
6.0
5.5
5.0
4.5
4.0
3.5
3.0
2.5
2.0
1.5
1.0
0.5
0.0
f1 (ppm)

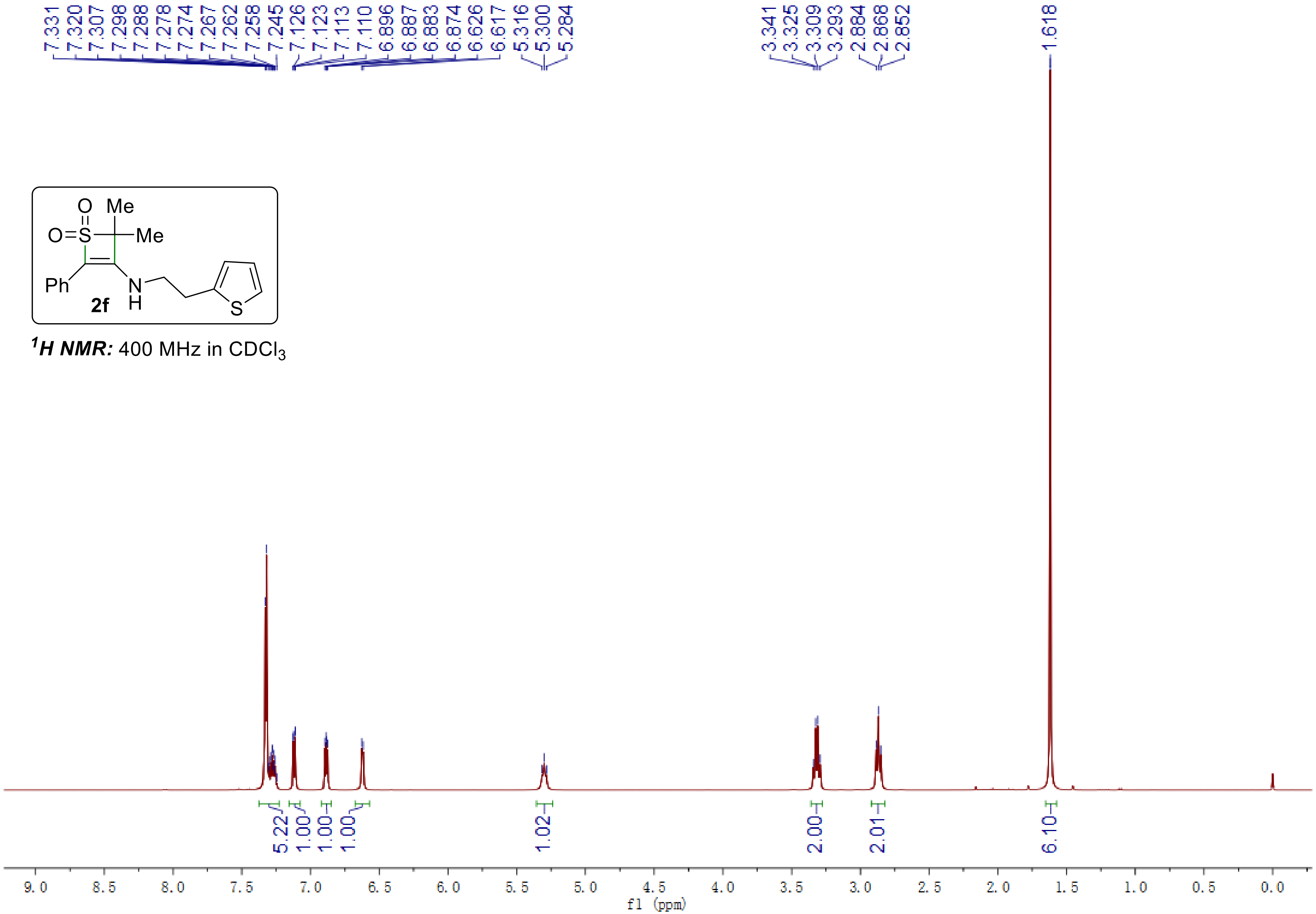

7.331 7.320 7.307 7.298 7.288 7.278 7.274 7.267 7.262 7.258 7.245 7.126 7.123 7.113 7.110 6.896 6.887 6.883 6.874 6.626 6.617 5.316 5.300 5.284
3.341 3.325 3.309 3.293 2.884 2.868 2.852
1.618
O Me
O=S
Me
Ph
N
H
S
2f
^{1}H NMR: 400 MHz in $CDCl_3$
5.22 1.00 1.00 1.00 1.02 2.00 2.01 6.10
9.0 8.5 8.0 7.5 7.0 6.5 6.0 5.5 5.0 4.5 4.0 3.5 3.0 2.5 2.0 1.5 1.0 0.5 0.0
f1 (ppm)

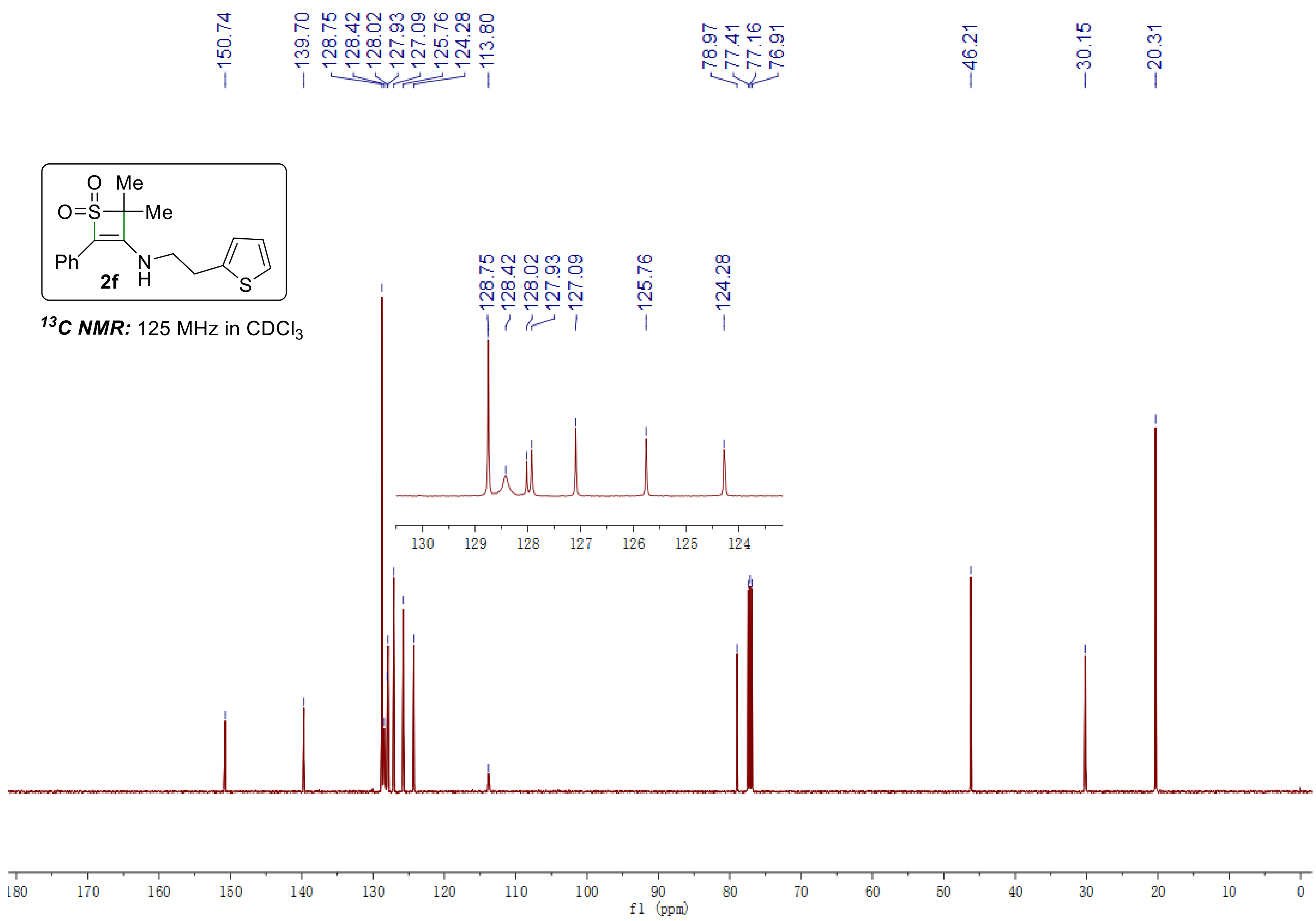
150.74
139.70
128.75
128.42
128.02
127.93
127.09
125.76
124.28
113.80
78.97
77.41
77.16
76.91
46.21
30.15
20.31
O
Me
O=S
Me
Ph
N
H
S
2f
^{13}C NMR: 125 MHz in $CDCl_3$
f1 (ppm)

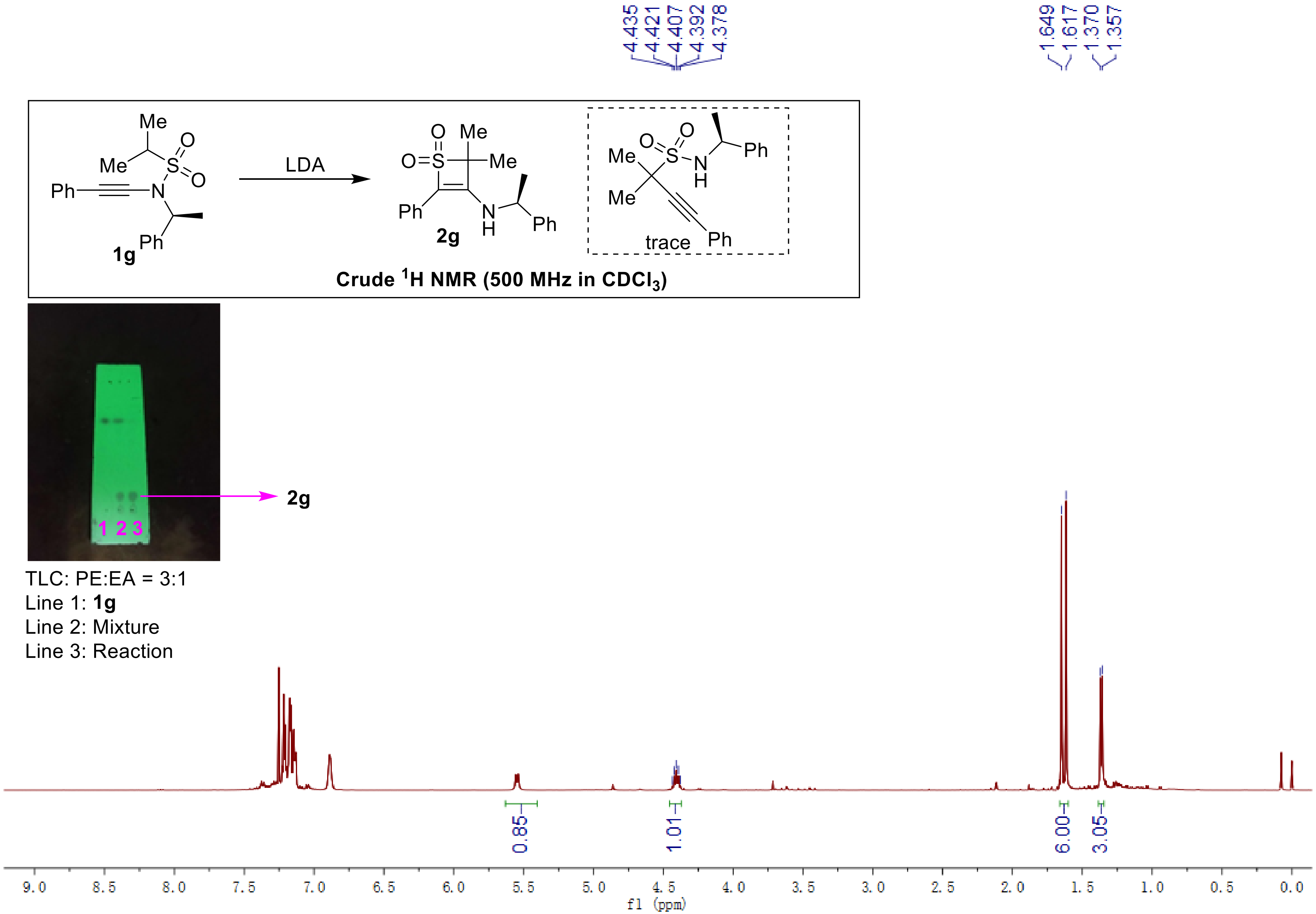

Me
Me
Ph
N
1g
Ph
LDA
O
Me
O=S
Me
Ph
2g
N
H
Ph
Me
O
O
S
N
H
Ph
Me
trace
Ph
Crude ^{1}H NMR (500 MHz in CDCl$_3$)
1 2 3
2g
TLC: PE:EA = 3:1
Line 1: 1g
Line 2: Mixture
Line 3: Reaction
4.435
4.421
4.407
4.392
4.378
1.649
1.617
1.370
1.357
0.85
1.01
6.00
3.05
9.0 8.5 8.0 7.5 7.0 6.5 6.0 5.5 5.0 4.5 4.0 3.5 3.0 2.5 2.0 1.5 1.0 0.5 0.0
f1 (ppm)

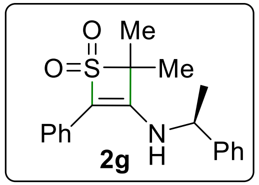

2g

[1]***H NMR:*** 500 MHz in $CDCl_3$

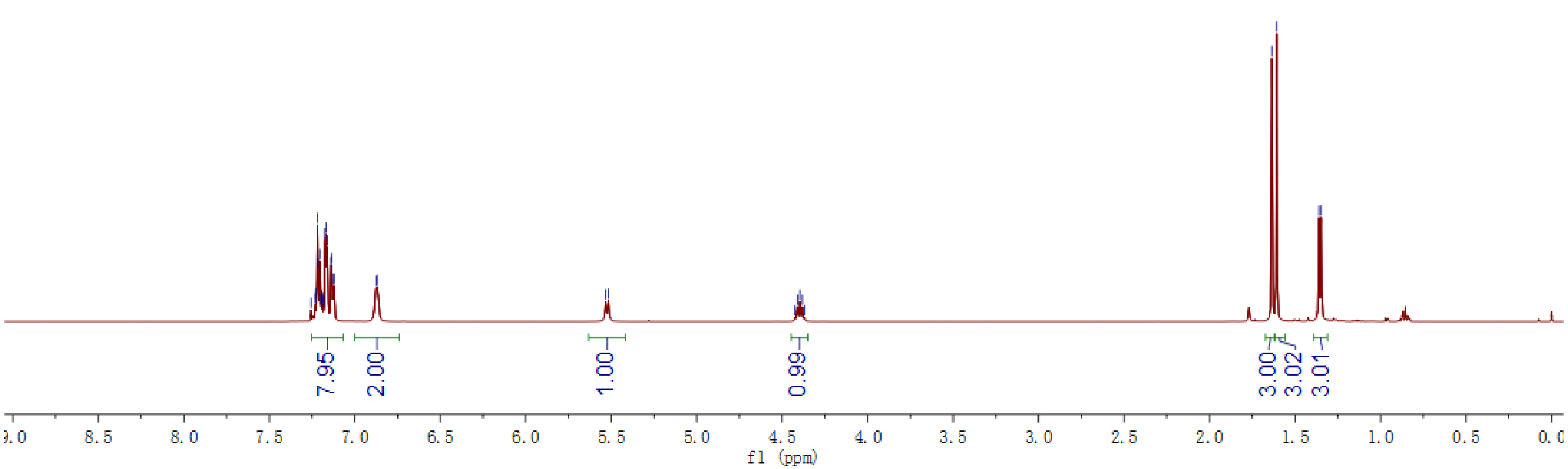

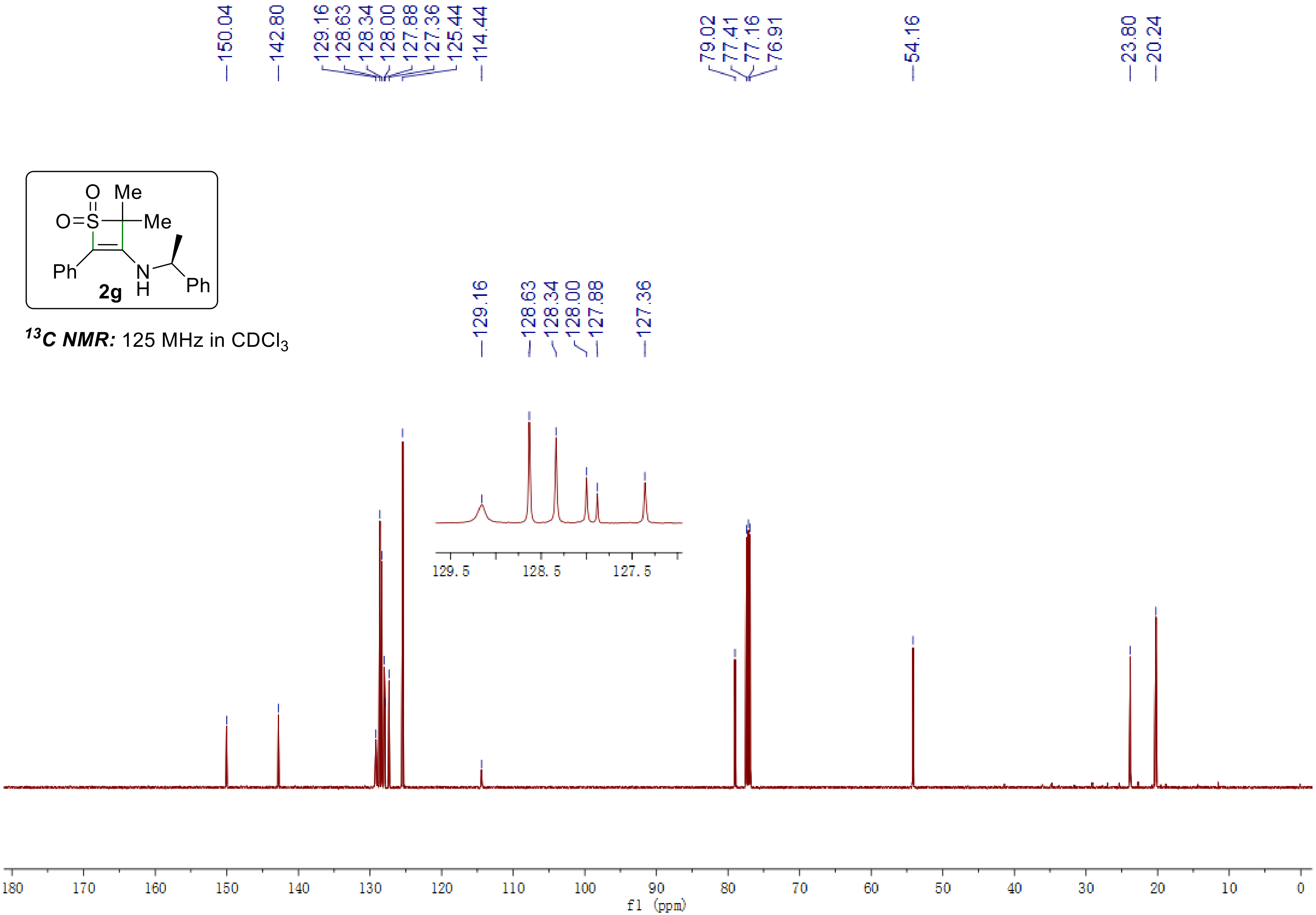
150.04
142.80
129.16
128.63
128.34
128.00
127.88
127.36
125.44
114.44
79.02
77.41
77.16
76.91
54.16
23.80
20.24
O
Me
O=S
Me
Ph
N
H
Ph
2g
13C NMR: 125 MHz in CDCl3
129.16
128.63
128.34
128.00
127.88
127.36
129.5
128.5
127.5
180
170
160
150
140
130
120
110
100
90
80
70
60
50
40
30
20
10
0
f1 (ppm)

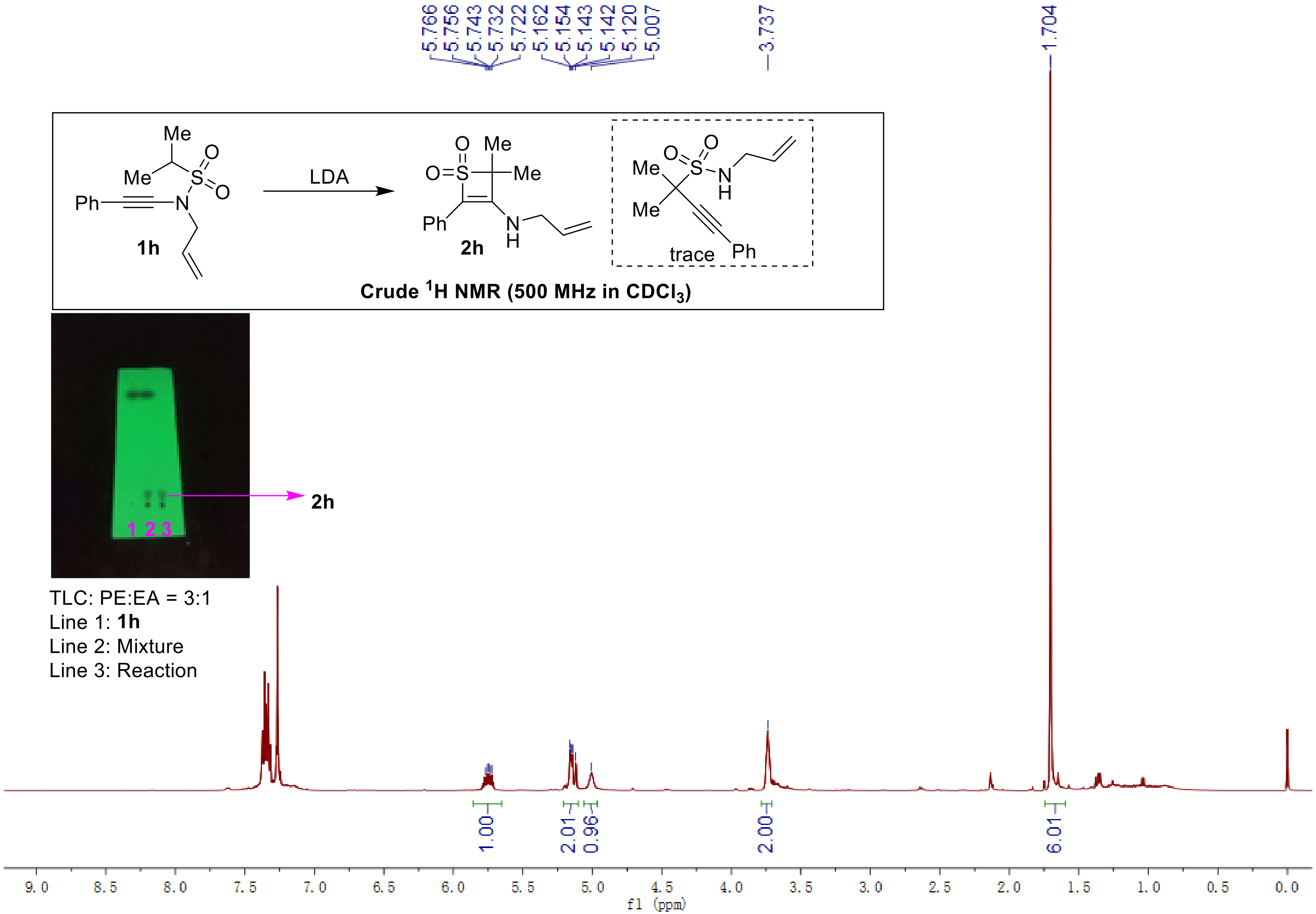
5.766
5.756
5.743
5.732
5.722
5.162
5.154
5.143
5.142
5.120
5.007
3.737
1.704
Me
O
S
O
Me
Ph
N
1h
LDA
O Me
O=S
Me
Ph
N
H
2h
Me
O
S
O
N
H
Me
trace
Ph
Crude ¹H NMR (500 MHz in CDCl₃)
2h
1 2 3
TLC: PE:EA = 3:1
Line 1: 1h
Line 2: Mixture
Line 3: Reaction
1.00
2.01
0.96
2.00
6.01
9.0
8.5
8.0
7.5
7.0
6.5
6.0
5.5
5.0
4.5
4.0
3.5
3.0
2.5
2.0
1.5
1.0
0.5
0.0
f1 (ppm)

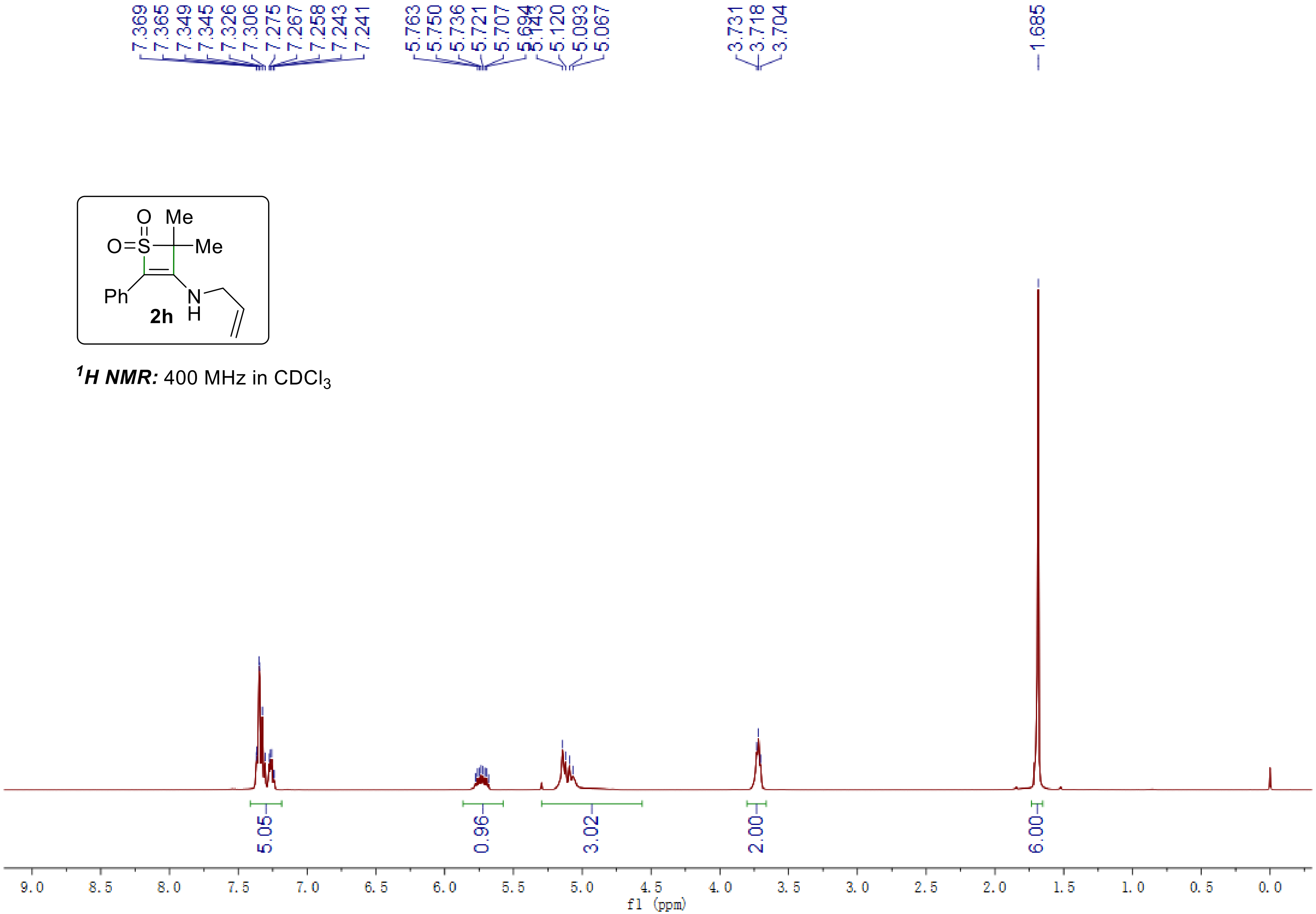
7.369
7.365
7.349
7.345
7.326
7.306
7.275
7.267
7.258
7.243
7.241
5.763
5.750
5.736
5.721
5.707
5.694
5.143
5.120
5.093
5.067
3.731
3.718
3.704
1.685
O
Me
O=S
Me
Ph
N
H
2h
1H NMR: 400 MHz in CDCl3
5.05
0.96
3.02
2.00
6.00
9.0
8.5
8.0
7.5
7.0
6.5
6.0
5.5
5.0
4.5
4.0
3.5
3.0
2.5
2.0
1.5
1.0
0.5
0.0
f1 (ppm)

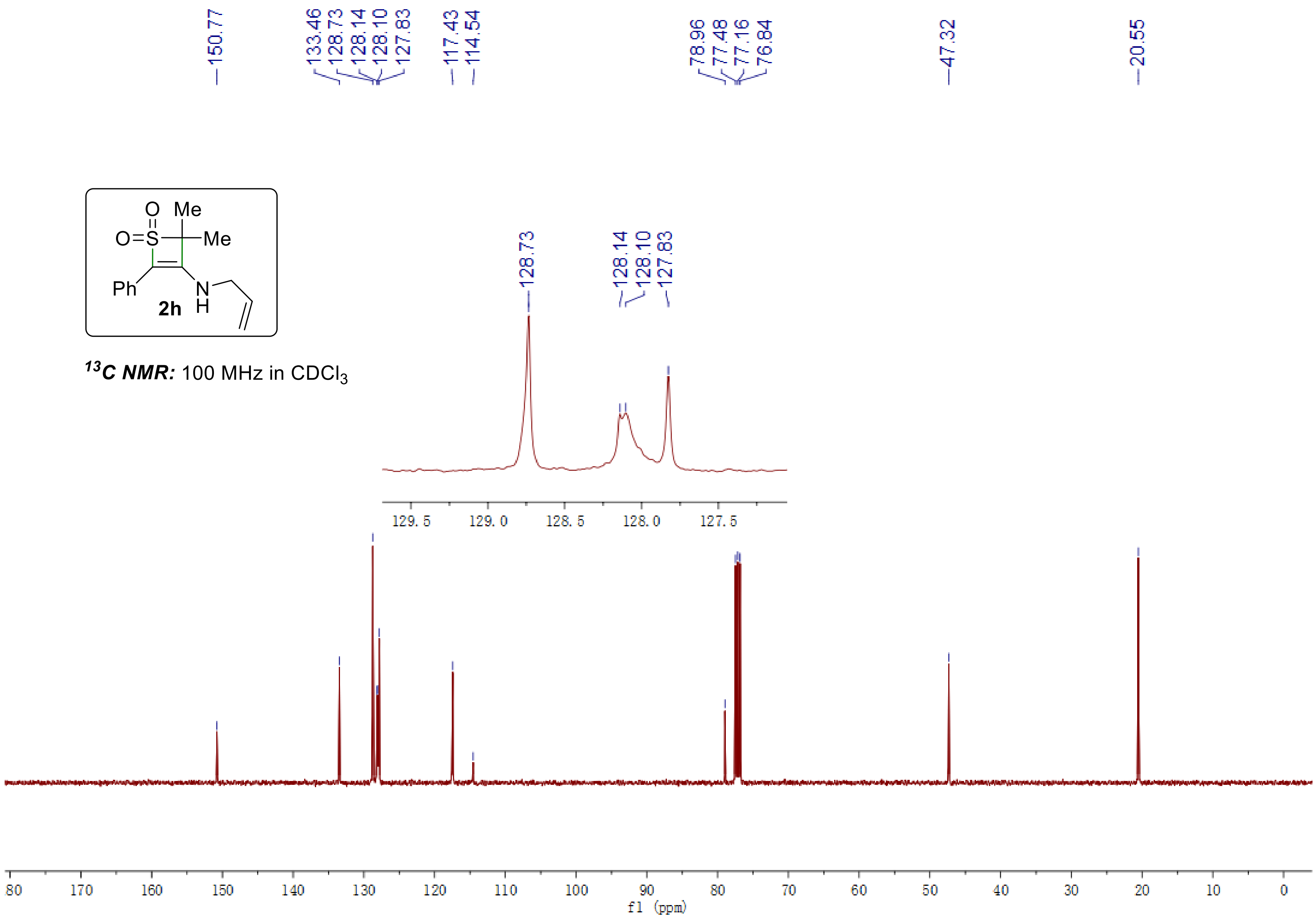
150.77
133.46
128.73
128.14
128.10
127.83
117.43
114.54
78.96
77.48
77.16
76.84
47.32
20.55
O
Me
O=S
Me
Ph
N
H
2h
13C NMR: 100 MHz in CDCl3
128.73
128.14
128.10
127.83
129.5
129.0
128.5
128.0
127.5
180
170
160
150
140
130
120
110
100
90
80
70
60
50
40
30
20
10
0
f1 (ppm)

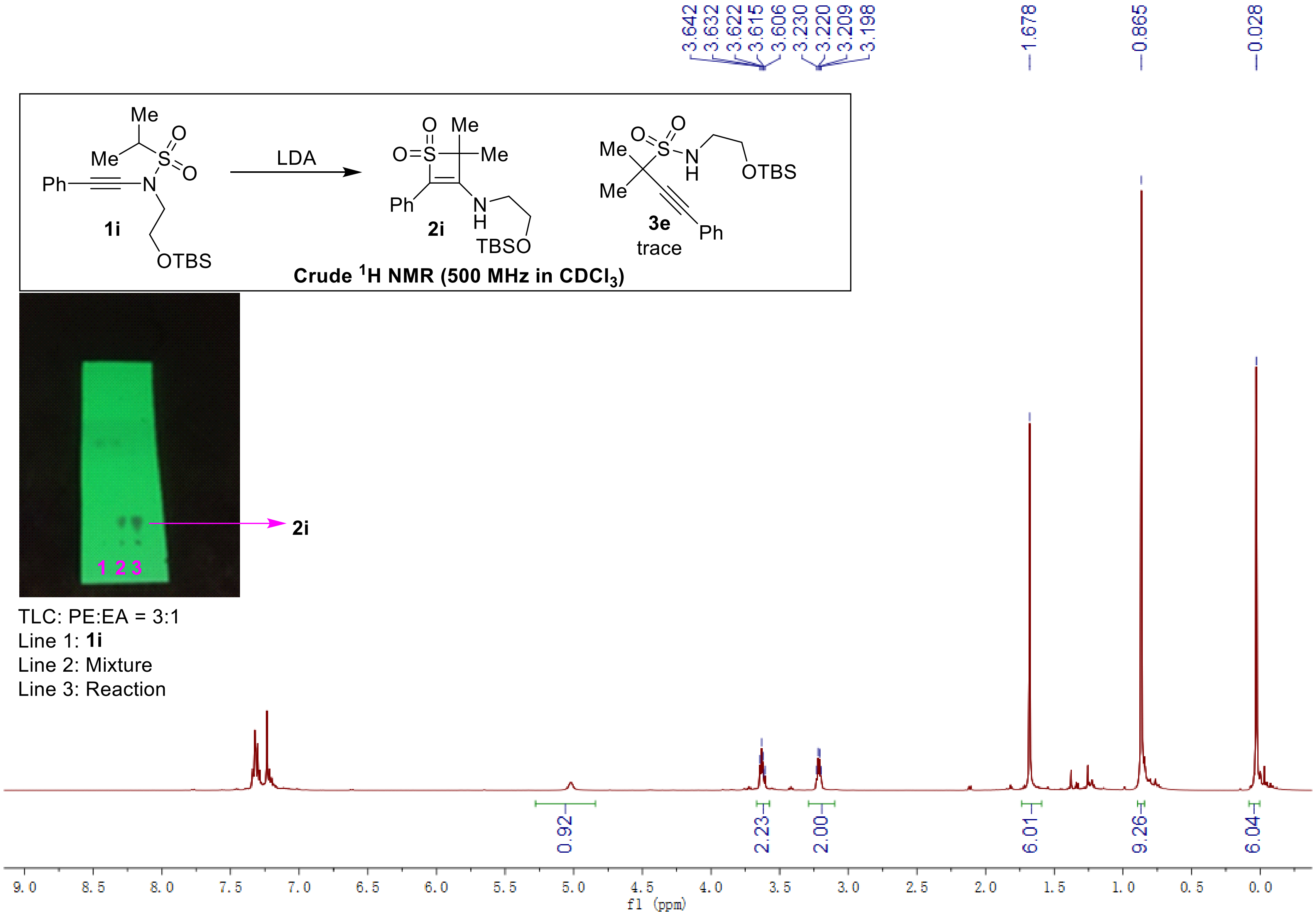
Me
O
Me
S
O
Ph
N
1i
OTBS
LDA
O Me
O=S
Me
Ph
N
H
2i
TBSO
O
Me
O
S
N
H
OTBS
Me
3e
trace
Ph
Crude 1H NMR (500 MHz in CDCl3)
2i
1 2 3
TLC: PE:EA = 3:1
Line 1: 1i
Line 2: Mixture
Line 3: Reaction
3.642
3.632
3.622
3.615
3.606
3.230
3.220
3.209
3.198
1.678
0.865
0.028
0.92
2.23
2.00
6.01
9.26
6.04
9.0
8.5
8.0
7.5
7.0
6.5
6.0
5.5
5.0
4.5
4.0
3.5
3.0
2.5
2.0
1.5
1.0
0.5
0.0
f1 (ppm)

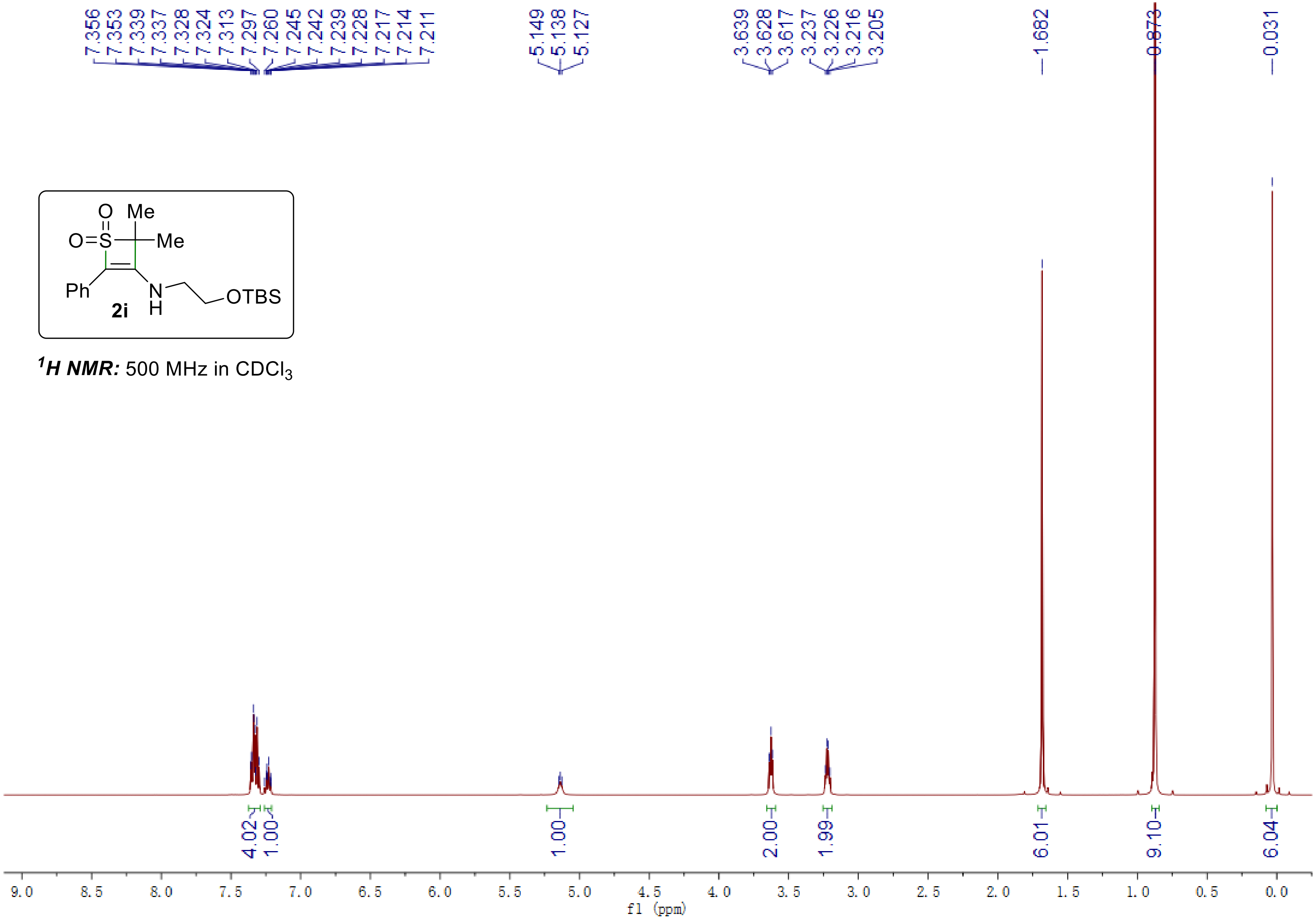
7.356 7.353 7.339 7.337 7.328 7.324 7.313 7.297 7.260 7.245 7.242 7.239 7.228 7.217 7.214 7.211
5.149 5.138 5.127
3.639 3.628 3.617 3.237 3.226 3.216 3.205
1.682
0.873
-0.031
O Me
O=S
Me
Ph
N
H
OTBS
2i
^{1}H NMR: 500 MHz in CDCl$_3$
4.02
1.00
1.00
2.00
1.99
6.01
9.10
6.04
9.0 8.5 8.0 7.5 7.0 6.5 6.0 5.5 5.0 4.5 4.0 3.5 3.0 2.5 2.0 1.5 1.0 0.5 0.0
f1 (ppm)

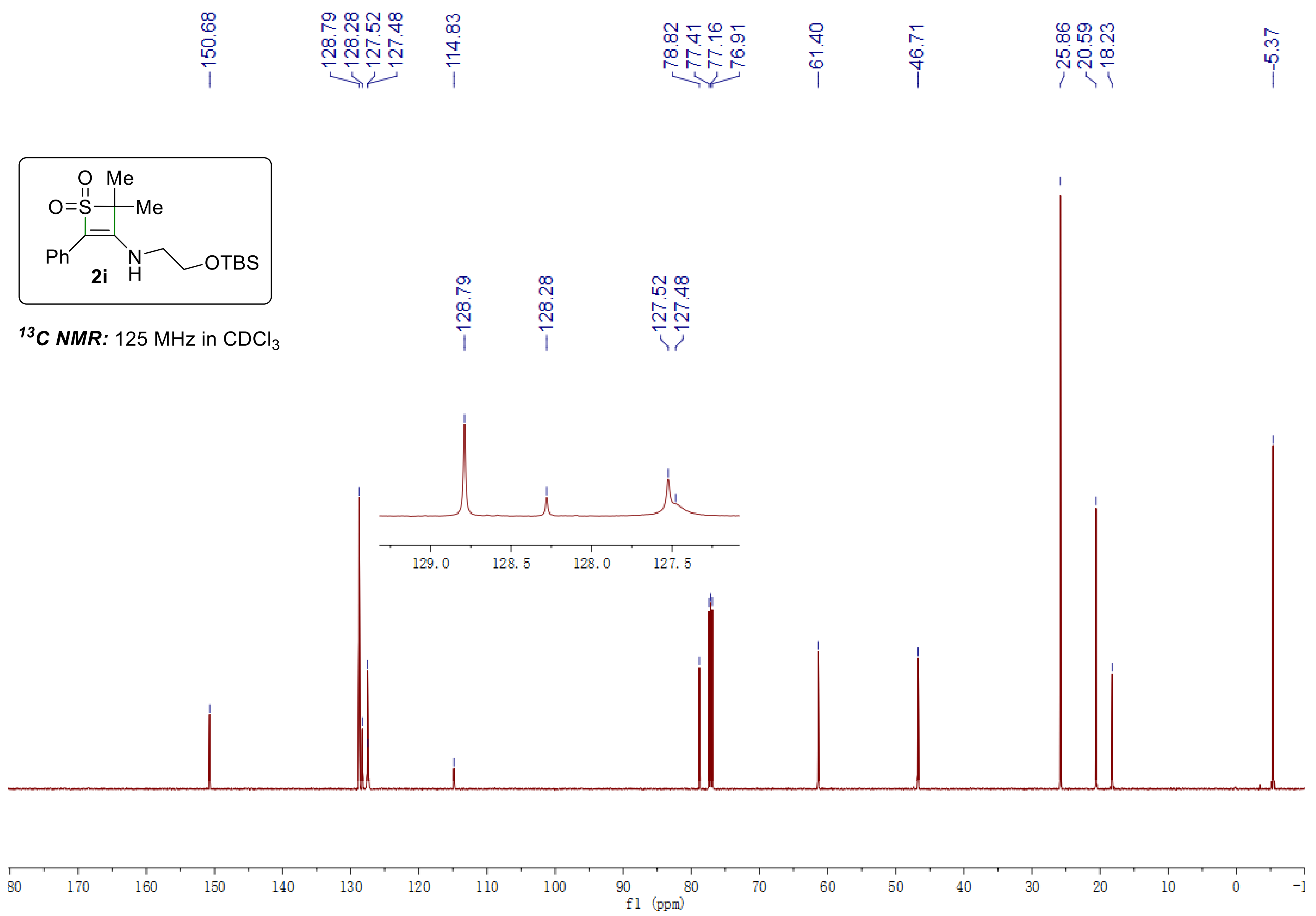
2i
O
Me
O=S
Me
Ph
N
H
OTBS
^{13}C NMR: 125 MHz in CDCl$_3$
150.68
128.79
128.28
127.52
127.48
114.83
78.82
77.41
77.16
76.91
61.40
46.71
25.86
20.59
18.23
-5.37
129.0
128.5
128.0
127.5
f1 (ppm)

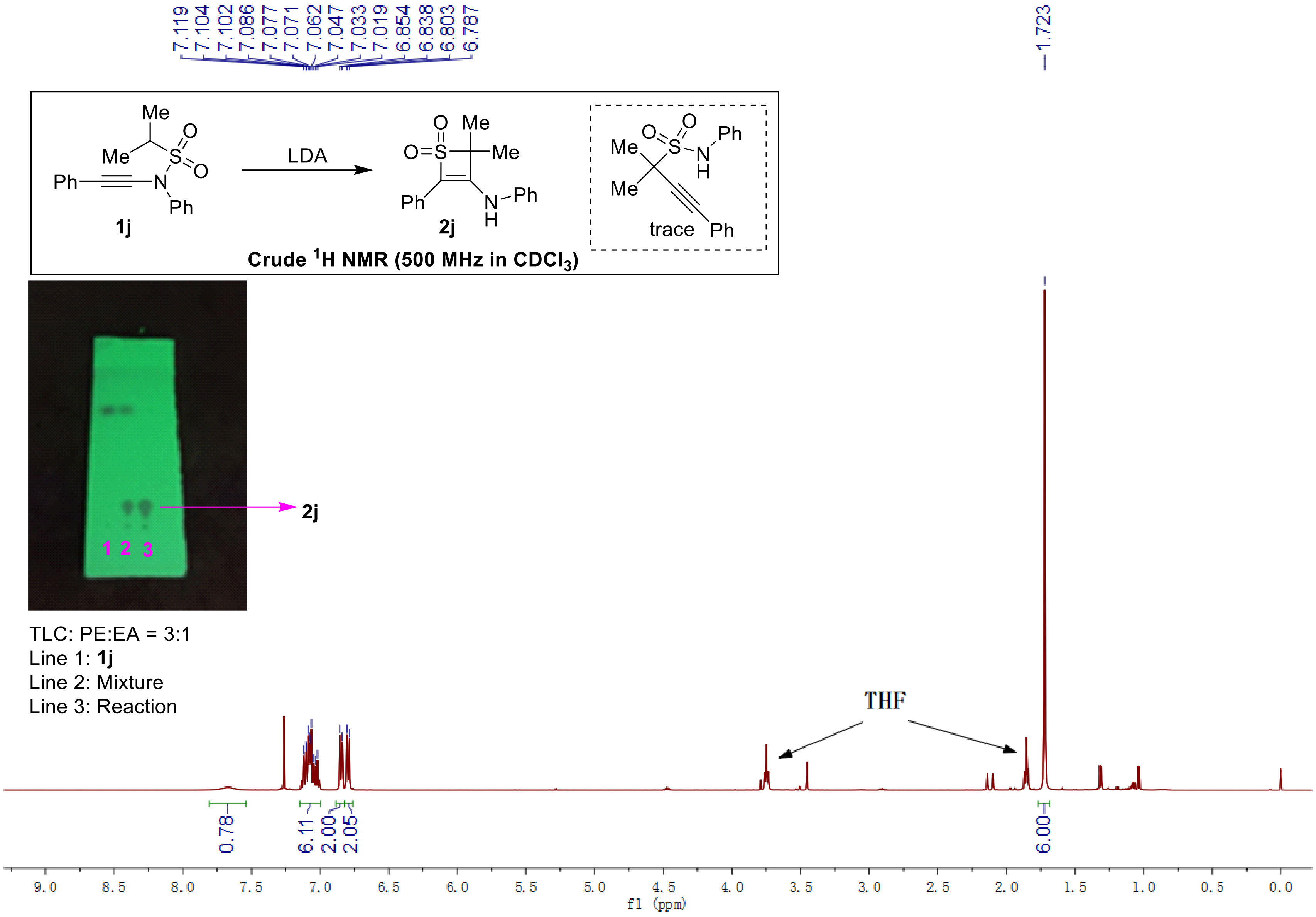
7.119
7.104
7.102
7.086
7.077
7.071
7.062
7.047
7.033
7.019
6.854
6.838
6.803
6.787
1.723
LDA
1j
2j
trace
Crude 1H NMR (500 MHz in CDCl3)
2j
1 2 3
TLC: PE:EA = 3:1
Line 1: 1j
Line 2: Mixture
Line 3: Reaction
THF
0.78
6.11
2.00
2.05
6.00
f1 (ppm)

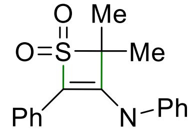

2j

^{1}H NMR: 400 MHz in $CDCl_3$

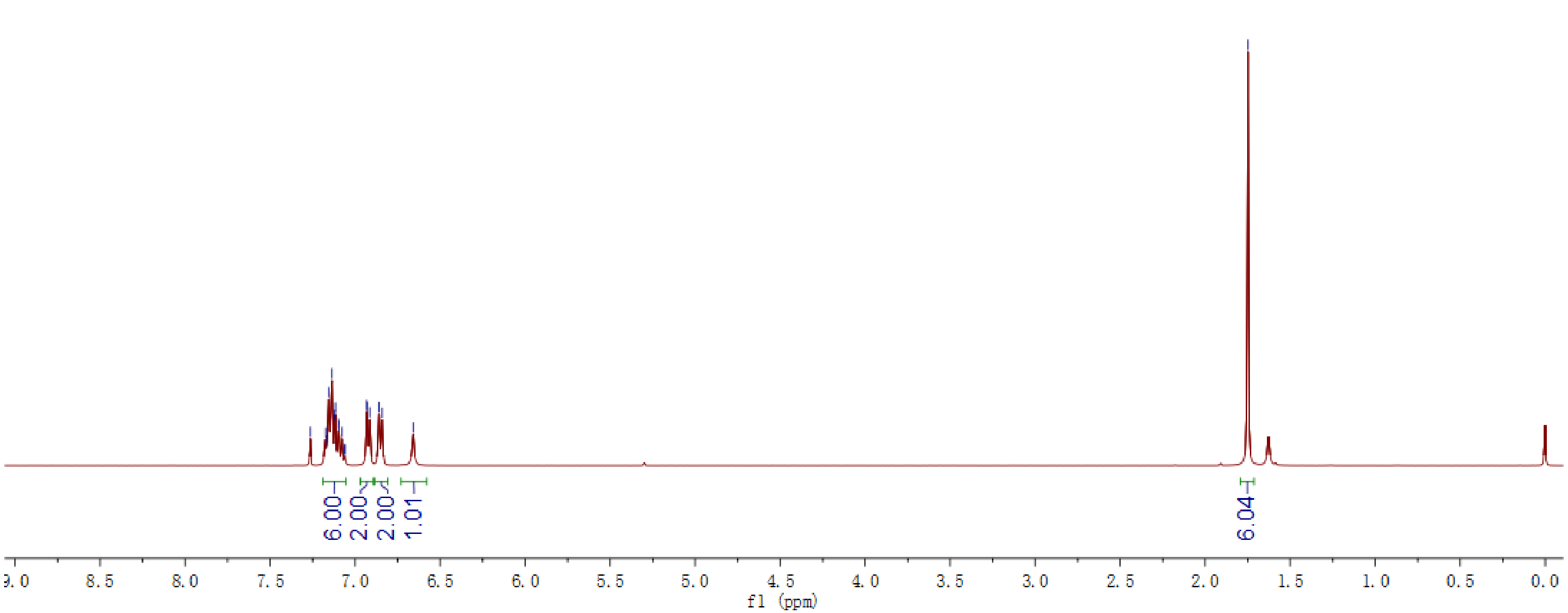

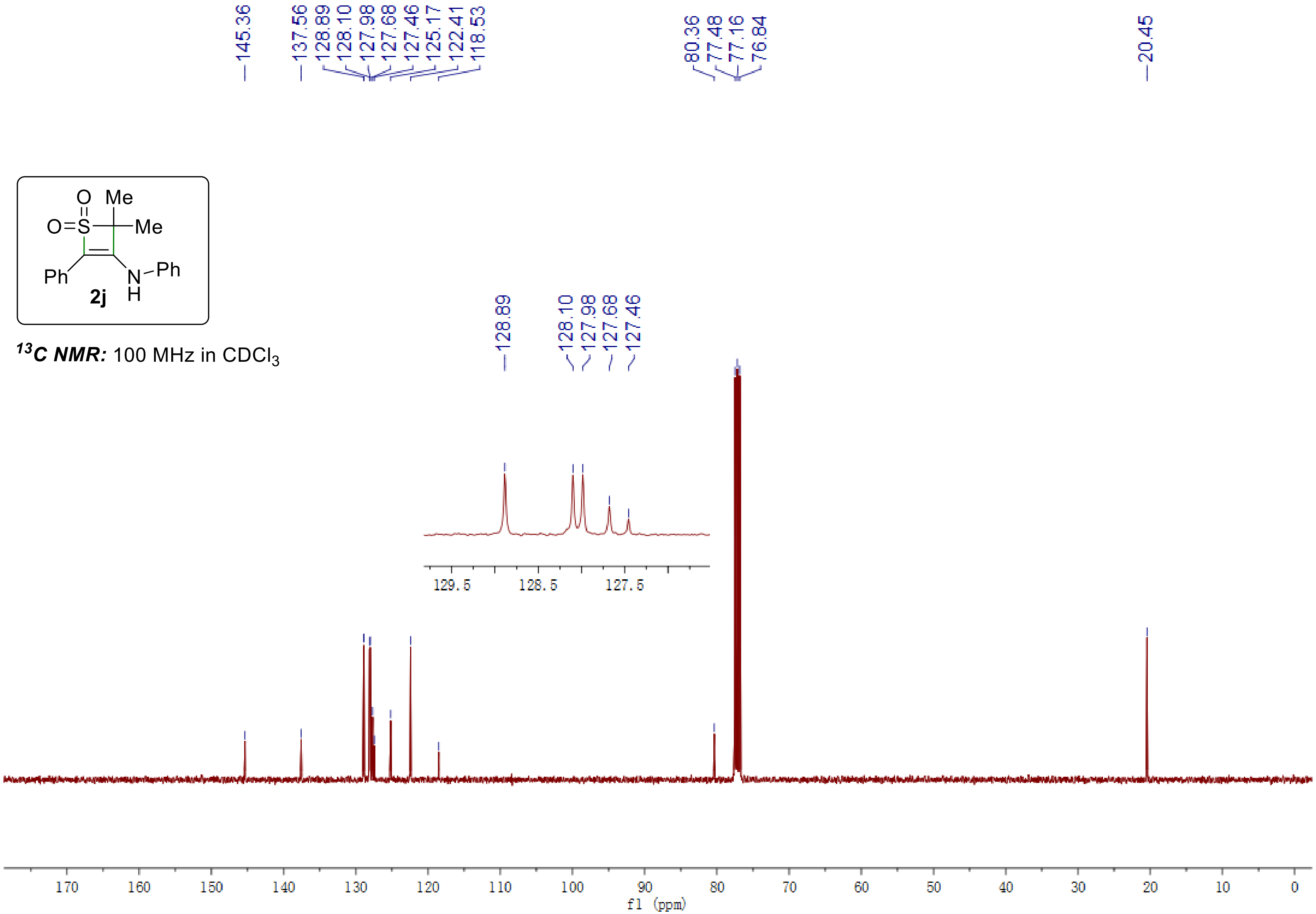
145.36
137.56
128.89
128.10
127.98
127.68
127.46
125.17
122.41
118.53
80.36
77.48
77.16
76.84
20.45
O
Me
O=S
Me
Ph
N
Ph
H
2j
13C NMR: 100 MHz in CDCl3
128.89
128.10
127.98
127.68
127.46
129.5
128.5
127.5
170
160
150
140
130
120
110
100
90
80
70
60
50
40
30
20
10
0
f1 (ppm)

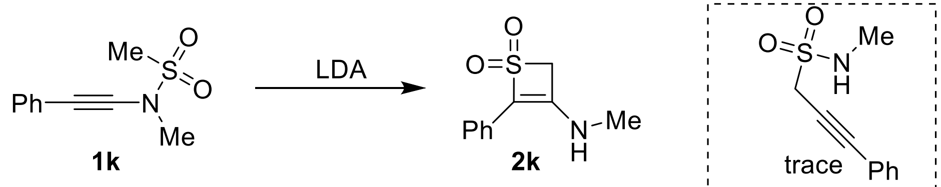

Crude ^{1}H NMR (500 MHz in DMSO-d_6)

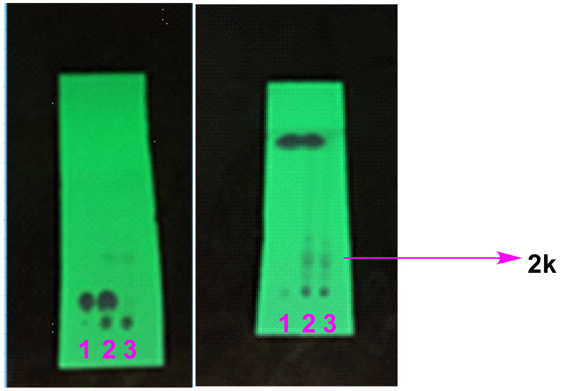

TLC: left, PE:EA = 20:1; right, PE:EA = 1:1
Line 1: **1k**
Line 2: Mixture
Line 3: Reaction

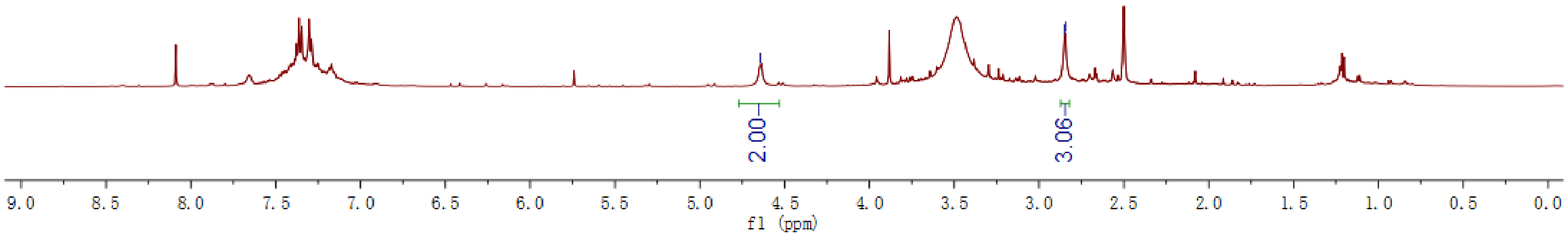

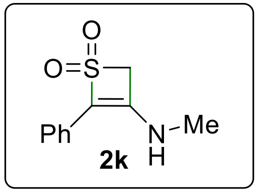

^{1}H NMR: 500 MHz in DMSO-d_6

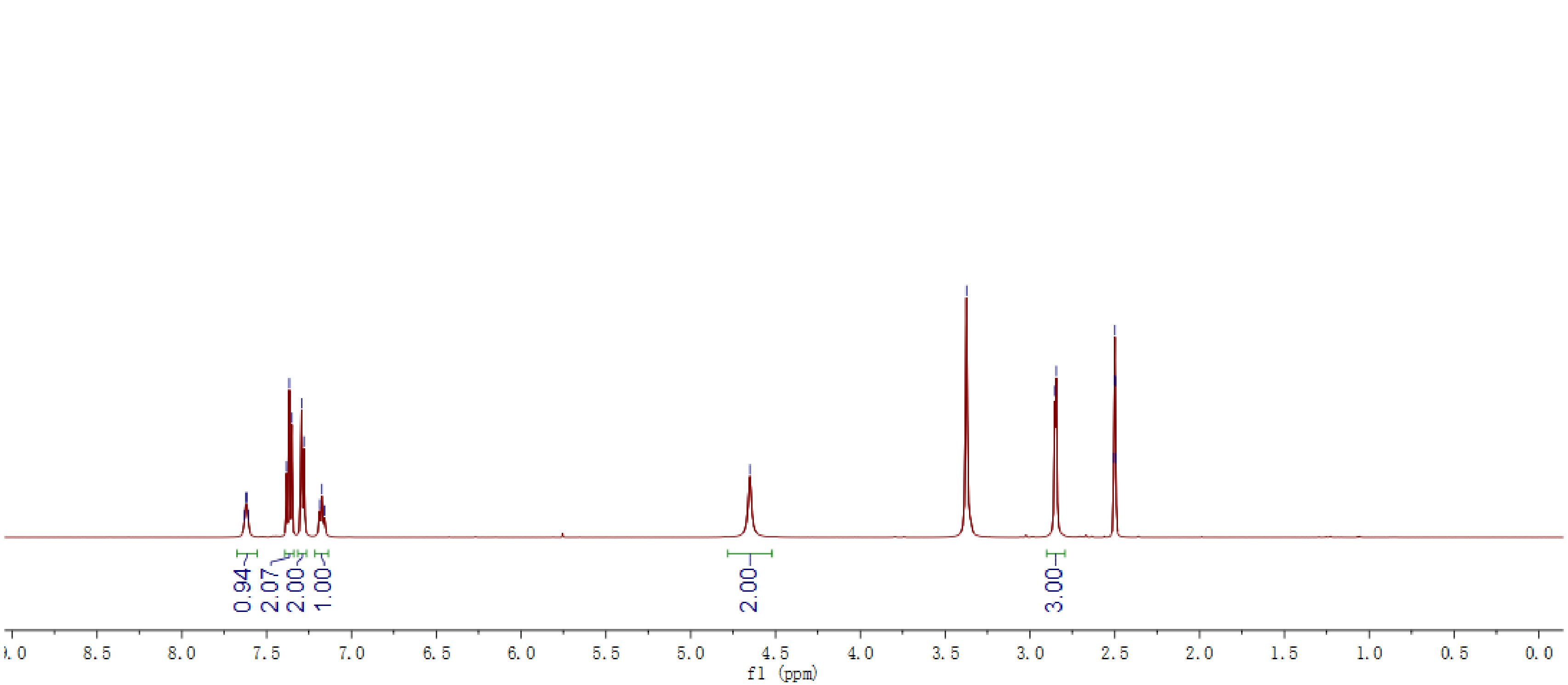

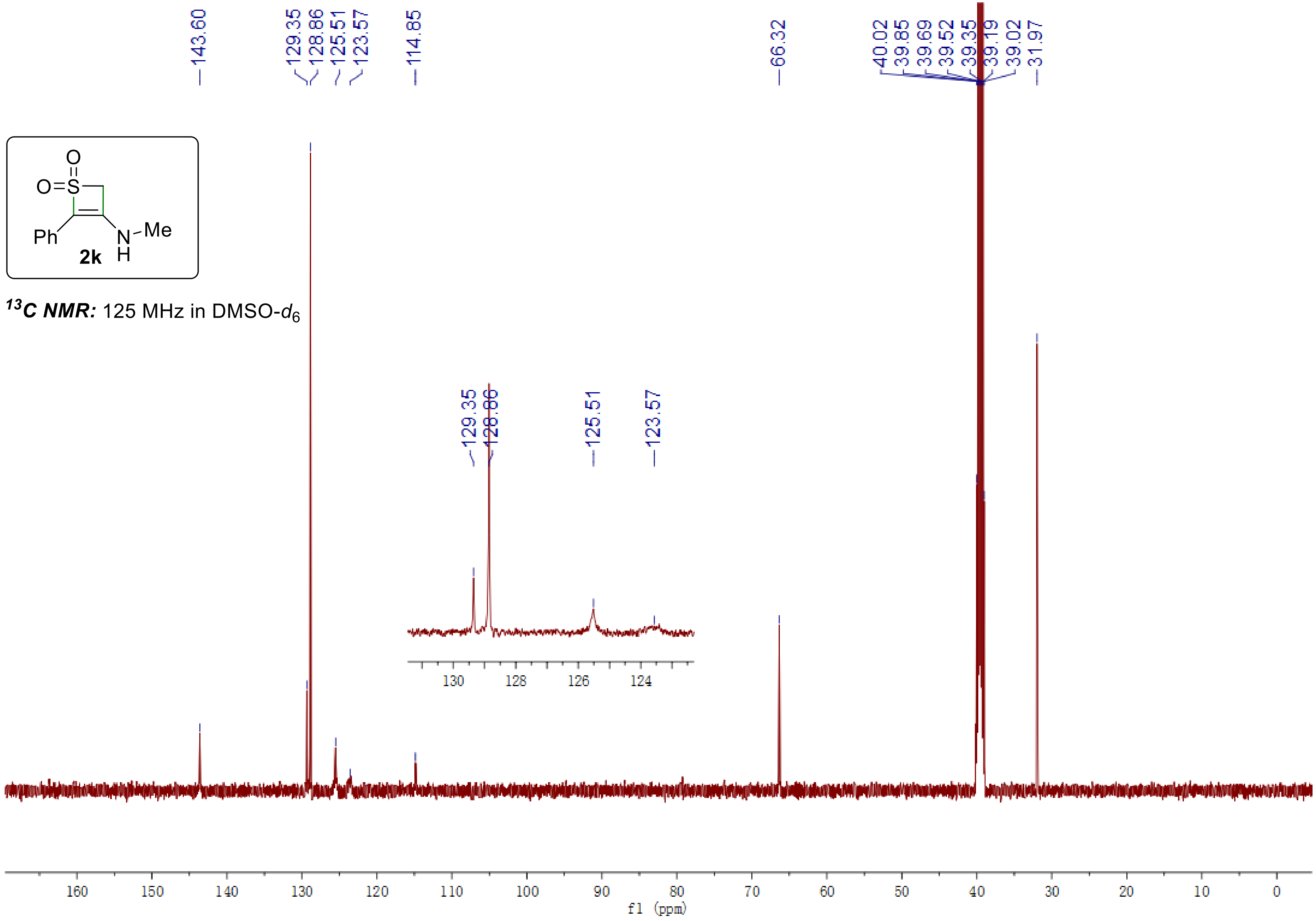
143.60
129.35
128.86
125.51
123.57
114.85
66.32
40.02
39.85
39.69
39.52
39.35
39.19
39.02
31.97
2k
^{13}C NMR: 125 MHz in DMSO-d_6
f1 (ppm)

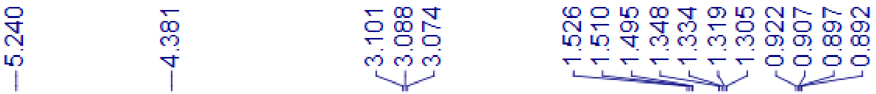

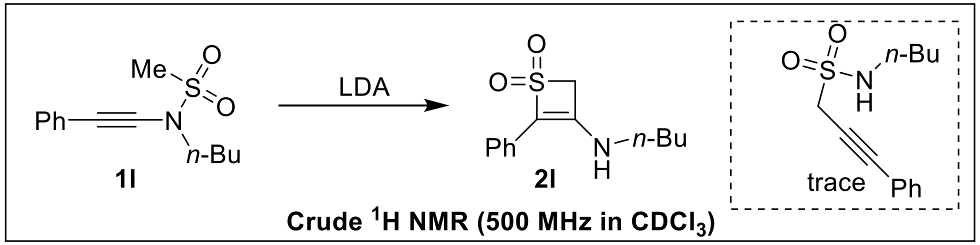

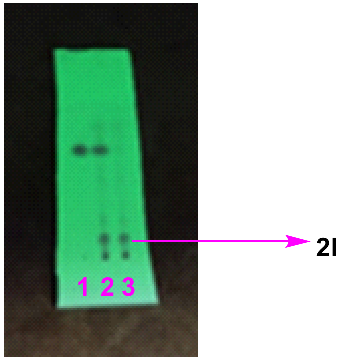

TLC:PE:EA = 3:1;
Line 1: **1l**
Line 2: Mixture
Line 3: Reaction

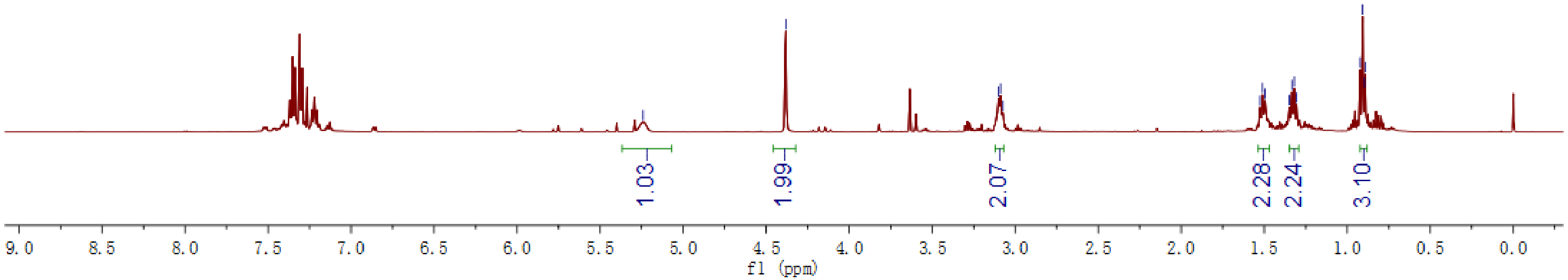

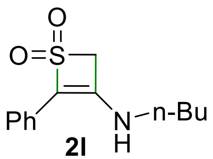

2l

^{1}H NMR: 600 MHz in $CDCl_3$

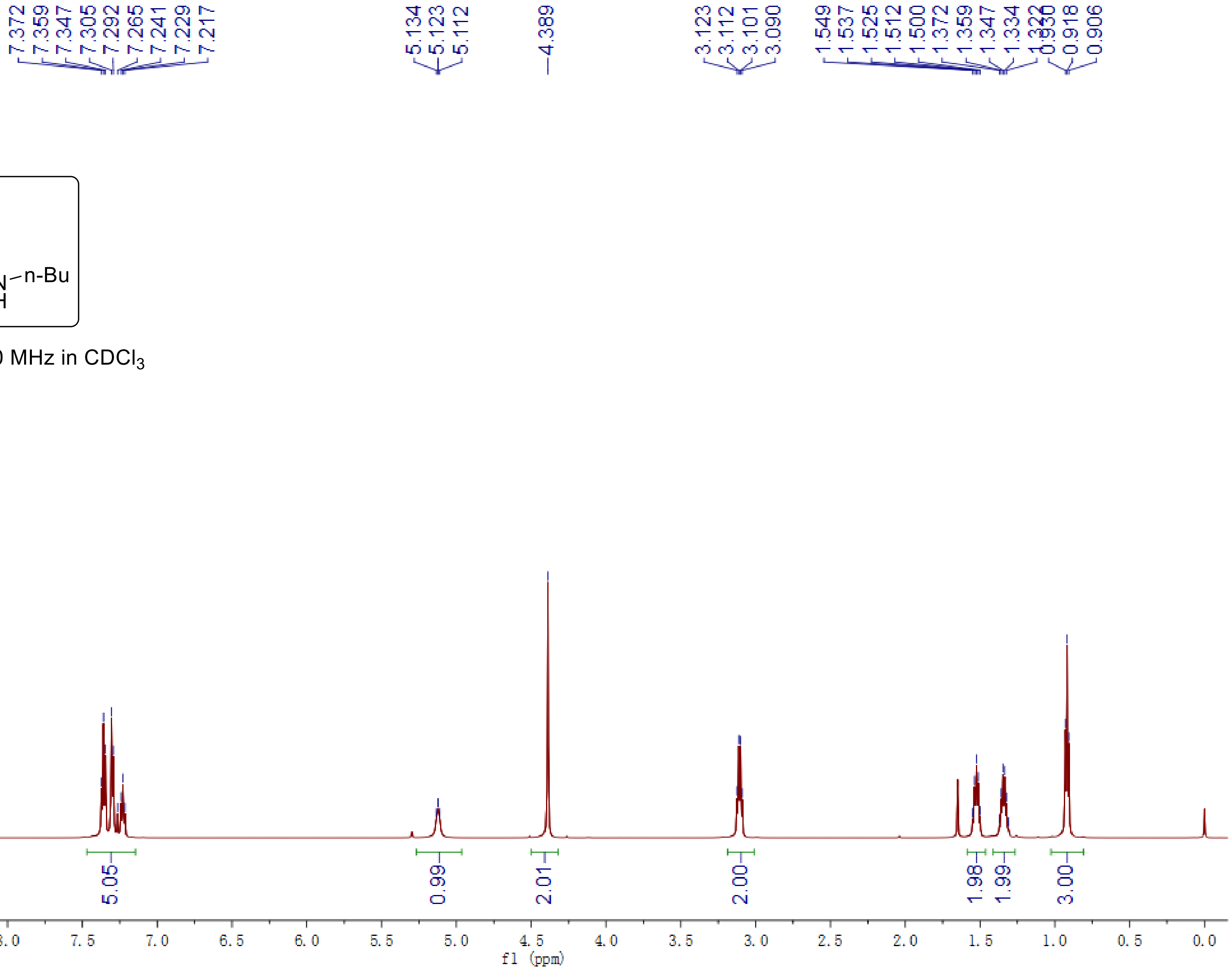

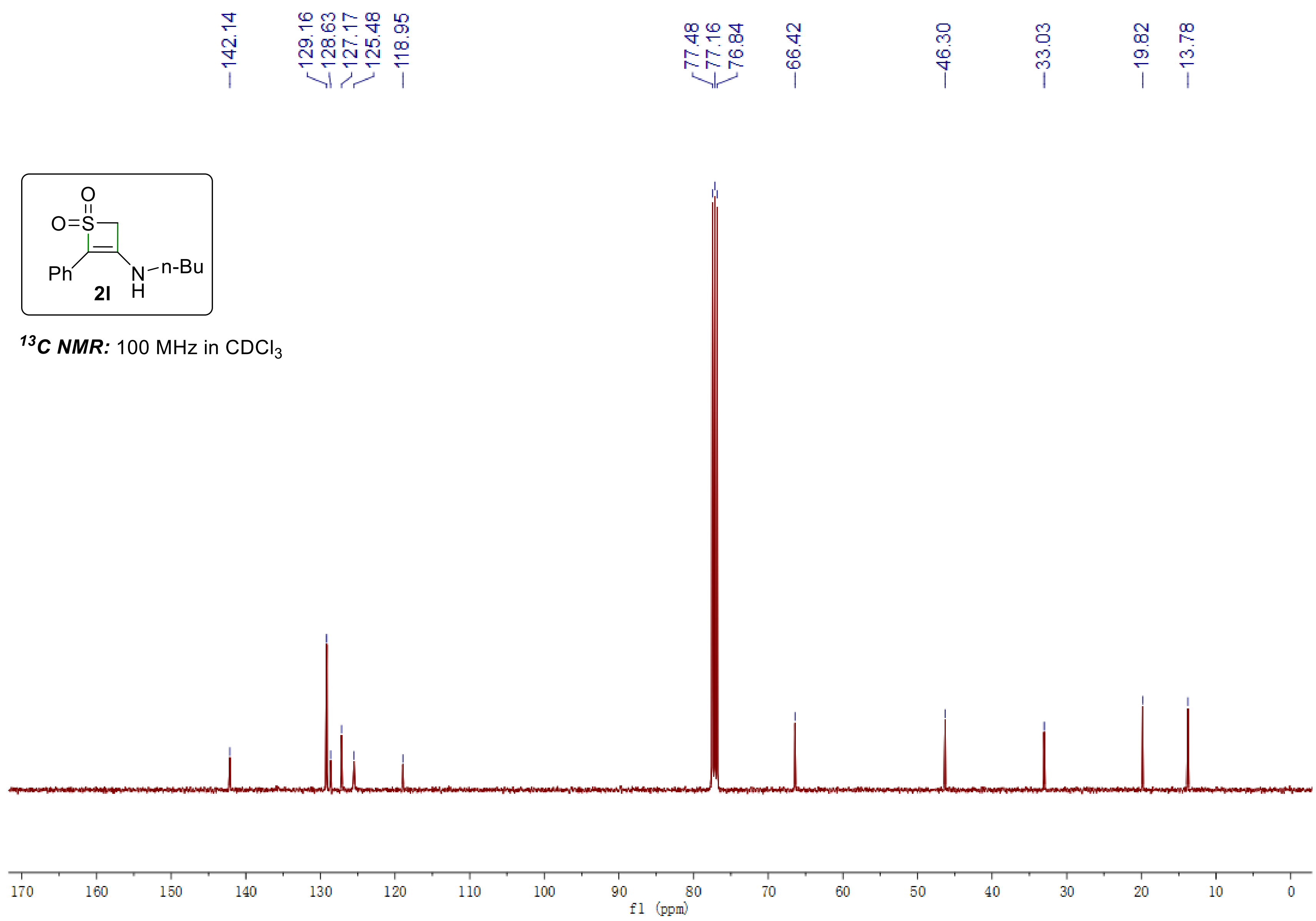
142.14
129.16
128.63
127.17
125.48
118.95
77.48
77.16
76.84
66.42
46.30
33.03
19.82
13.78
O=S
Ph
N
H
n-Bu
2l
^{13}C NMR: 100 MHz in $CDCl_3$
170 160 150 140 130 120 110 100 90 80 70 60 50 40 30 20 10 0
f1 (ppm)

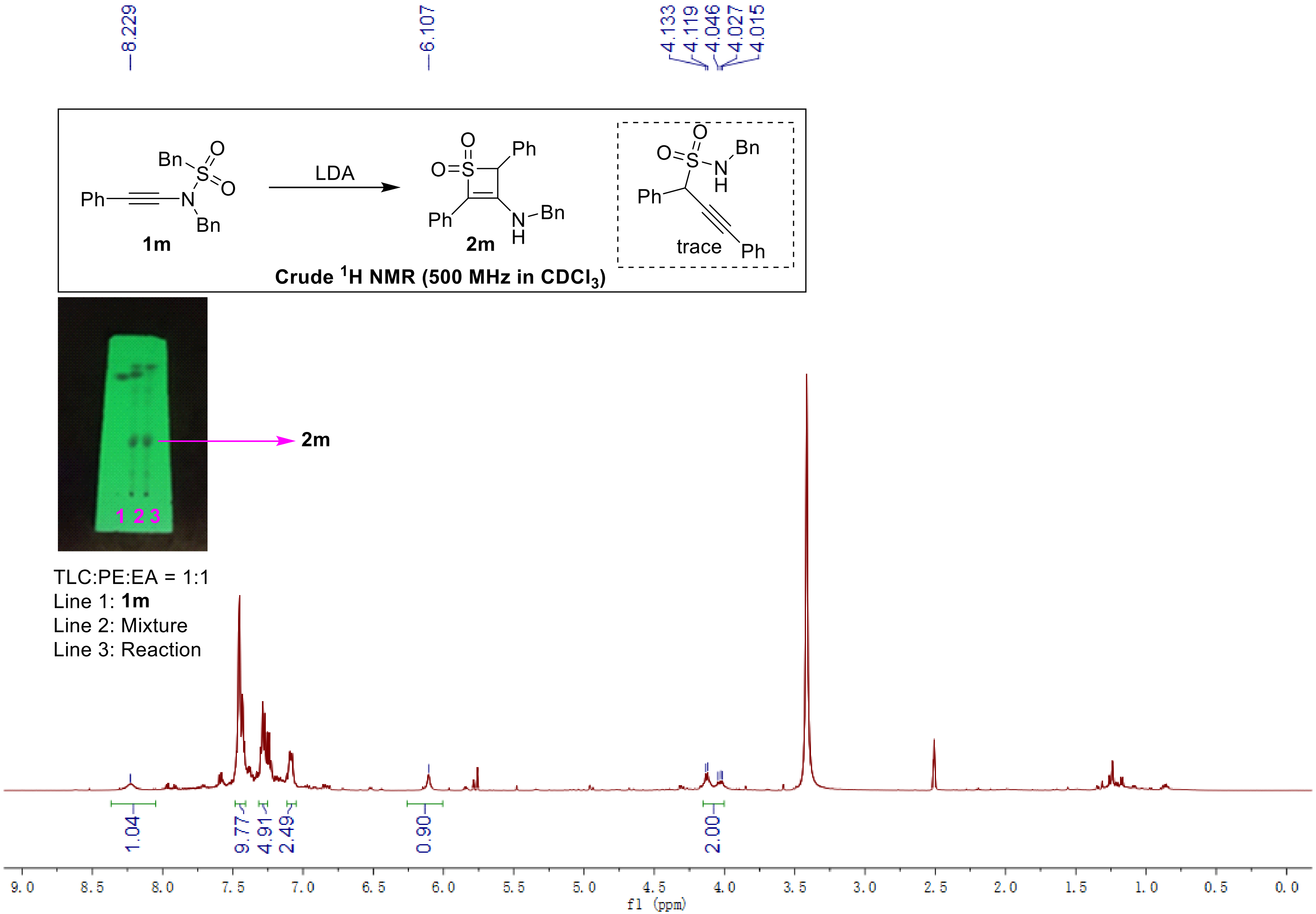
8.229
6.107
4.133
4.119
4.046
4.027
4.015
Ph
Bn
O
S
O
N
Bn
1m
LDA
O
O=S
Ph
Ph
N–Bn
H
2m
O
O=S
N
H
Bn
Ph
trace
Ph
Crude 1H NMR (500 MHz in CDCl3)
2m
1 2 3
TLC:PE:EA = 1:1
Line 1: 1m
Line 2: Mixture
Line 3: Reaction
1.04
9.77
4.91
2.49
0.90
2.00
9.0 8.5 8.0 7.5 7.0 6.5 6.0 5.5 5.0 4.5 4.0 3.5 3.0 2.5 2.0 1.5 1.0 0.5 0.0
f1 (ppm)

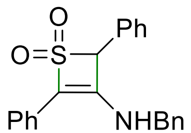

2m

^{1}H NMR: 400 MHz in DMSO-d_6

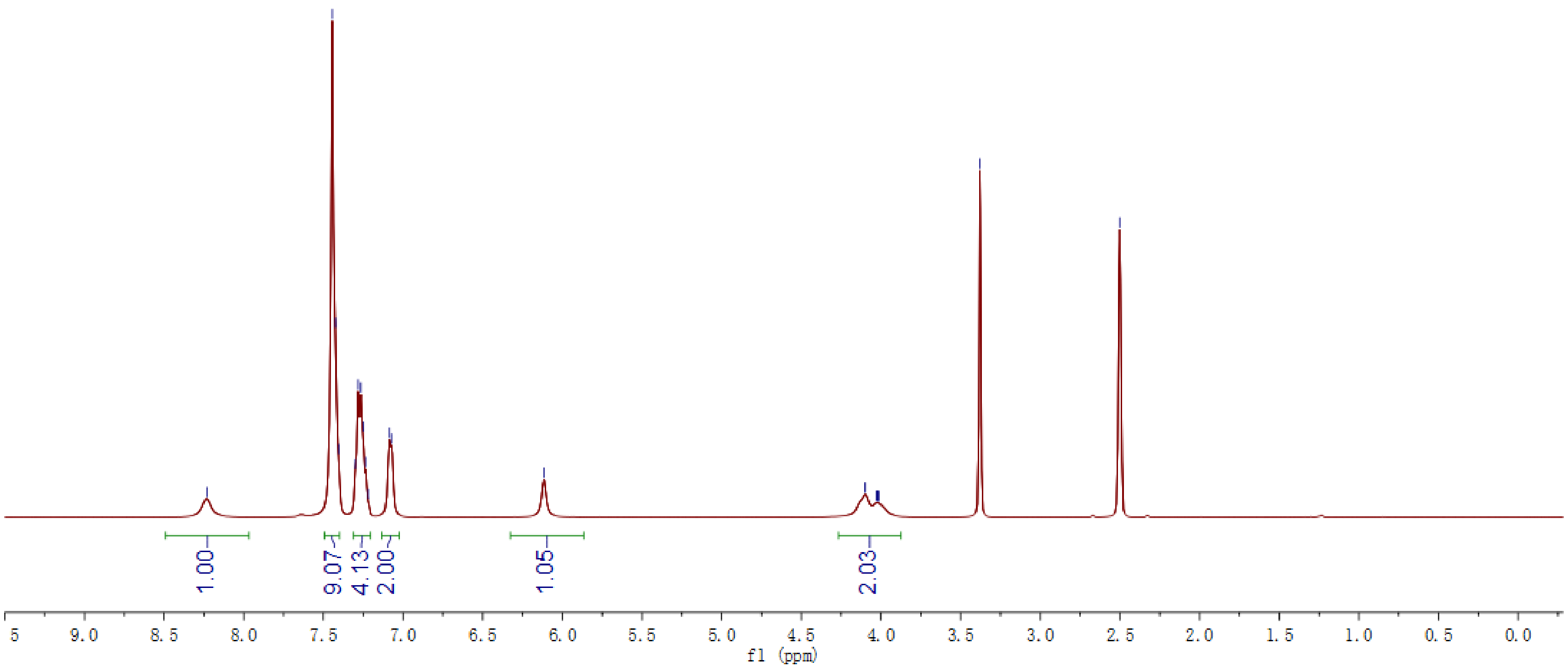

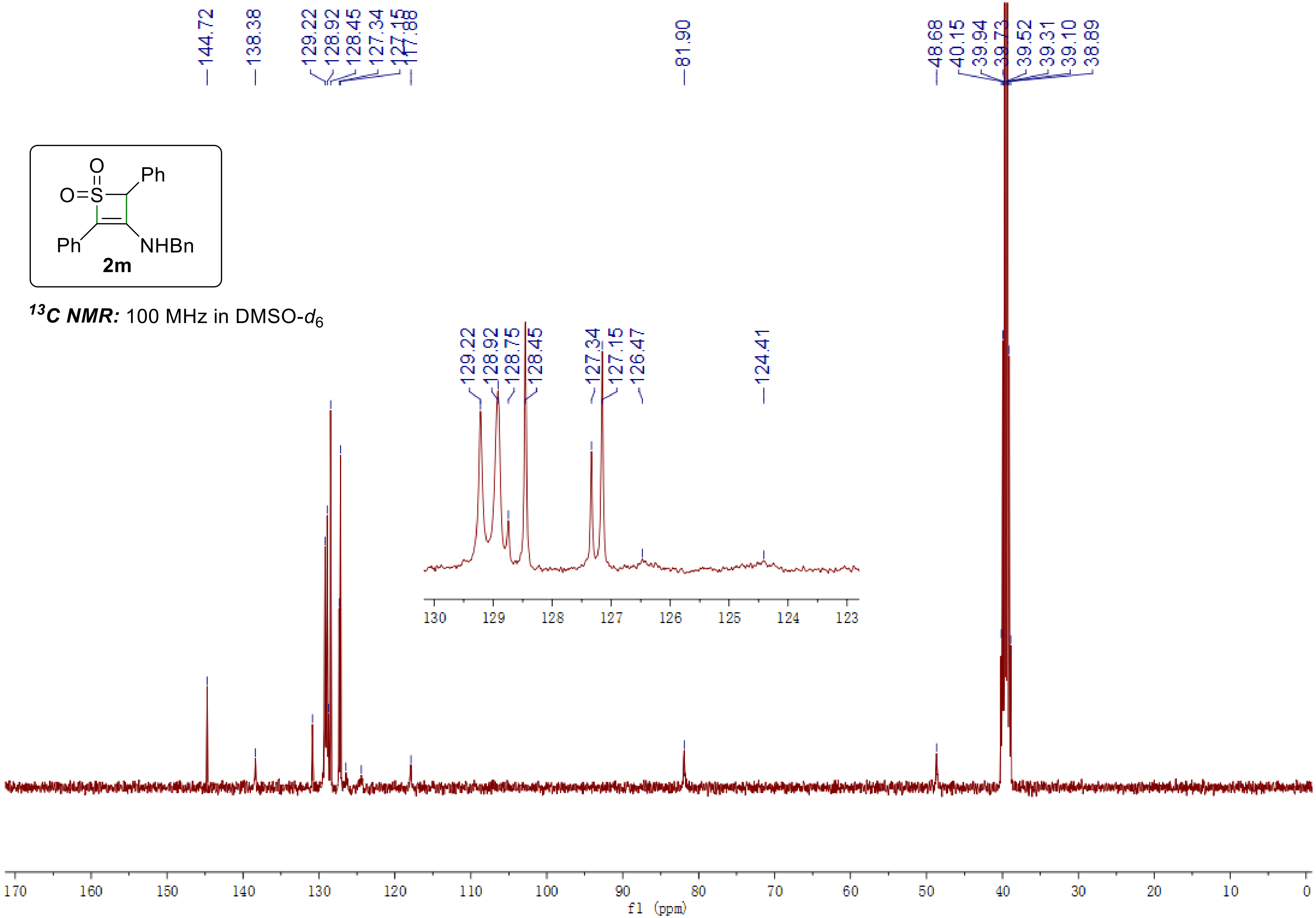
144.72
138.38
129.22
128.92
128.45
127.34
127.15
117.88
81.90
48.68
40.15
39.94
39.73
39.52
39.31
39.10
38.89
O
O=S
Ph
Ph
NHBn
2m
13C NMR: 100 MHz in DMSO-d6
129.22
128.92
128.75
128.45
127.34
127.15
126.47
124.41
130 129 128 127 126 125 124 123
170 160 150 140 130 120 110 100 90 80 70 60 50 40 30 20 10 0
f1 (ppm)

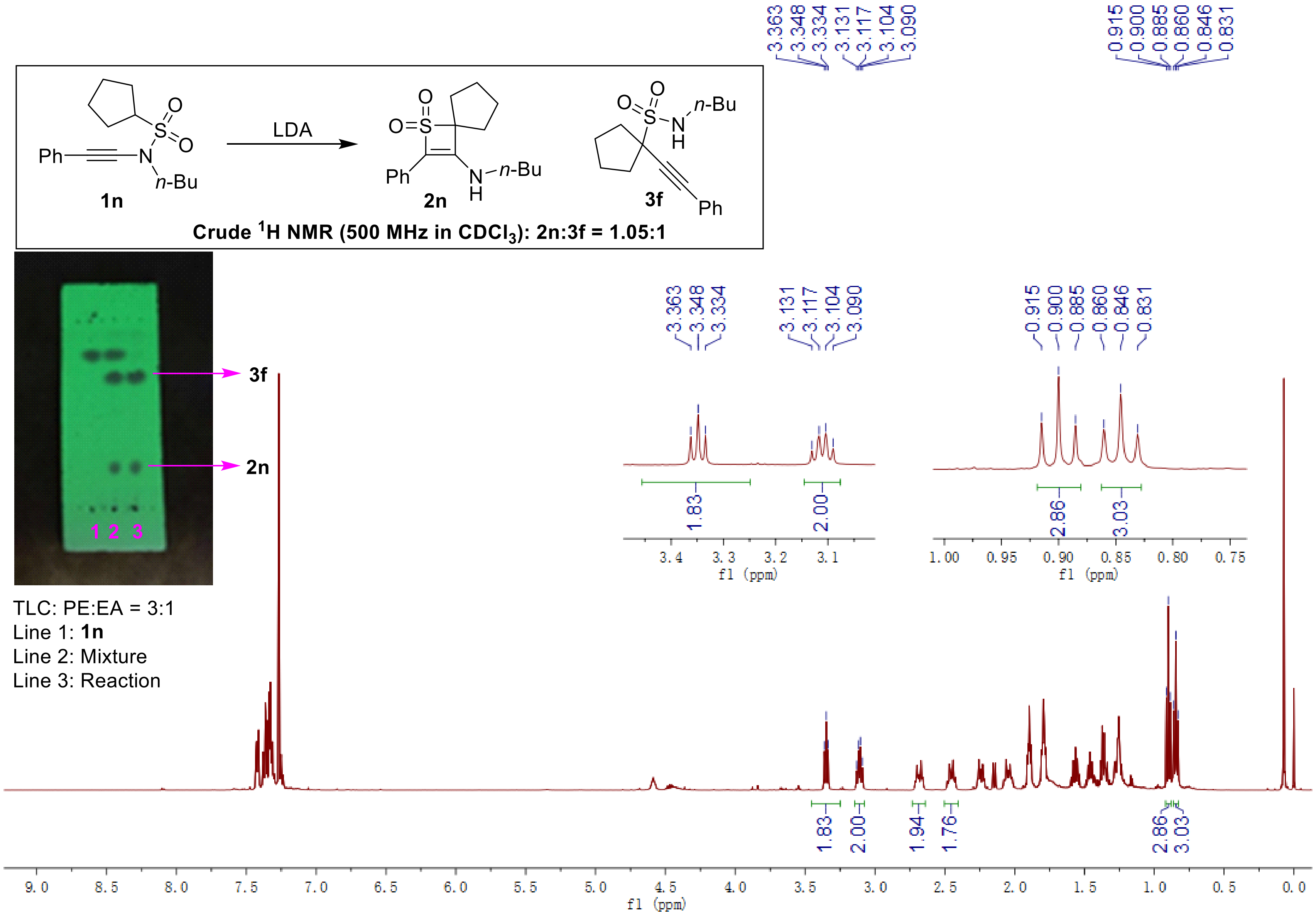

Crude ^{1}H NMR (500 MHz in $CDCl_3$): 2n:3f = 1.05:1

TLC: PE:EA = 3:1
Line 1: **1n**
Line 2: Mixture
Line 3: Reaction

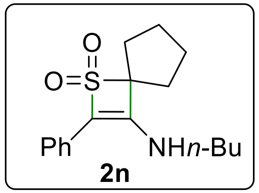

^{1}H NMR: 500 MHz in $CDCl_3$

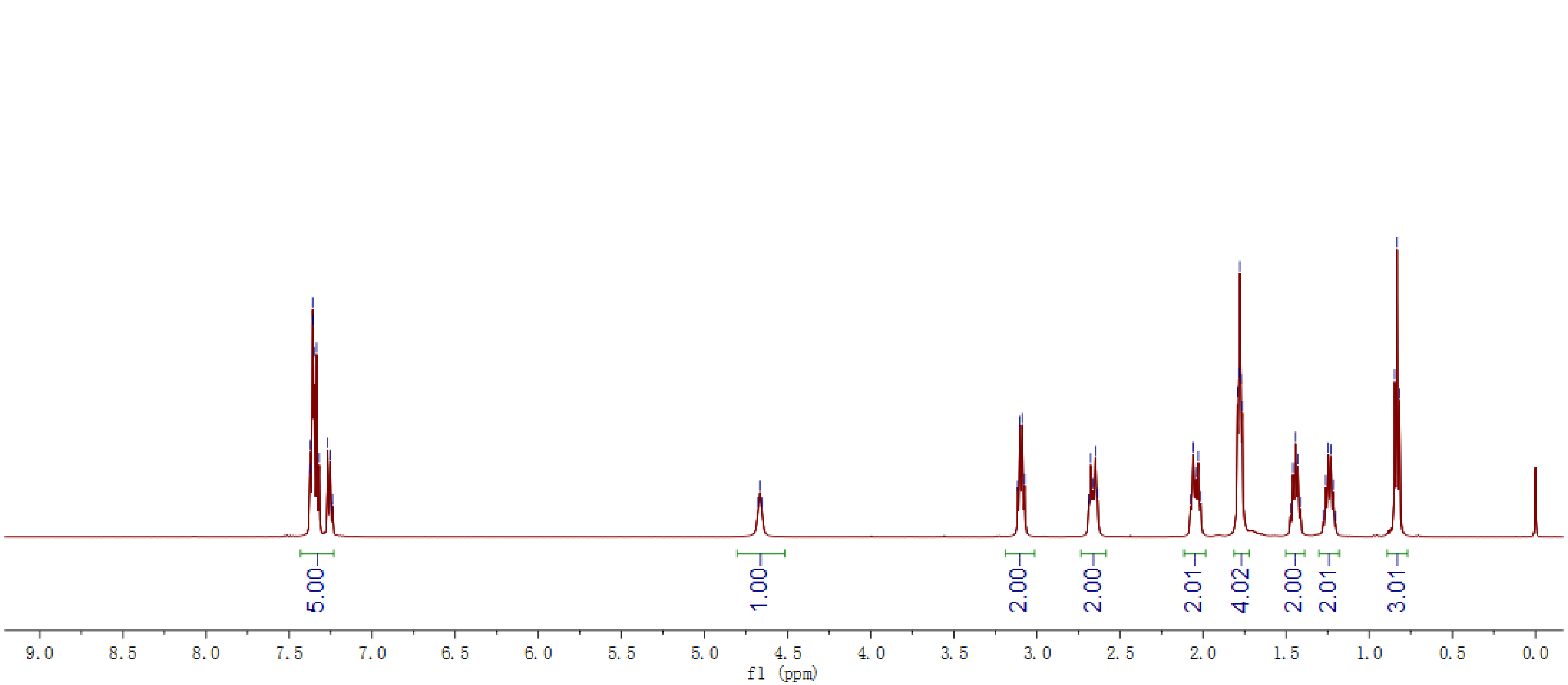

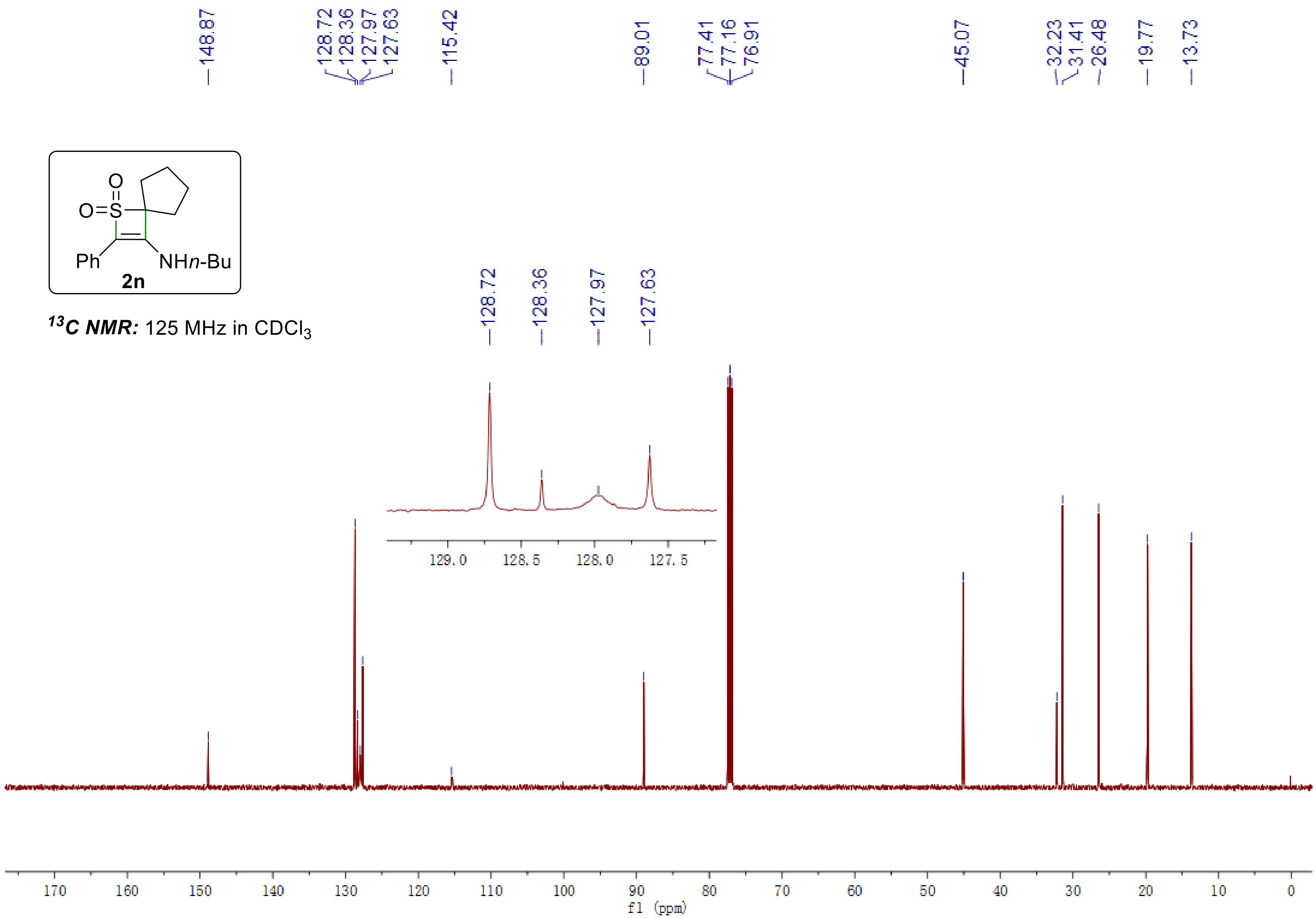

148.87
128.72
128.36
127.97
127.63
115.42
89.01
77.41
77.16
76.91
45.07
32.23
31.41
26.48
19.77
13.73
O
O=S
Ph
NHn-Bu
2n
13C NMR: 125 MHz in CDCl3
128.72
128.36
127.97
127.63
129.0
128.5
128.0
127.5
170
160
150
140
130
120
110
100
90
80
70
60
50
40
30
20
10
0
f1 (ppm)

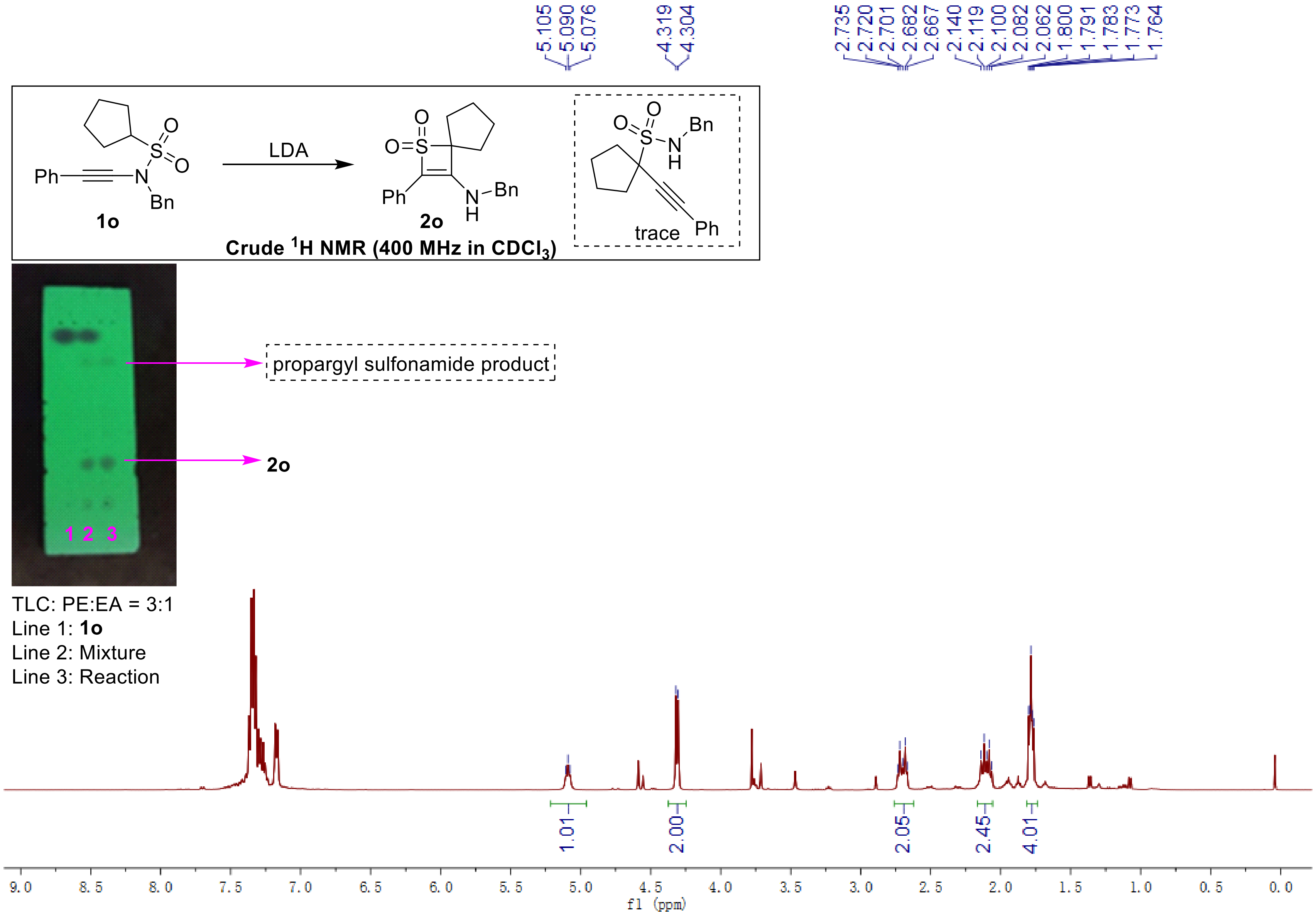
LDA
1o
2o
trace
Crude ¹H NMR (400 MHz in CDCl₃)
propargyl sulfonamide product
2o
1 2 3
TLC: PE:EA = 3:1
Line 1: 1o
Line 2: Mixture
Line 3: Reaction
5.105 5.090 5.076 4.319 4.304 2.735 2.720 2.701 2.682 2.667 2.140 2.119 2.100 2.082 2.062 1.800 1.791 1.783 1.773 1.764
1.01 2.00 2.05 2.45 4.01
f1 (ppm)

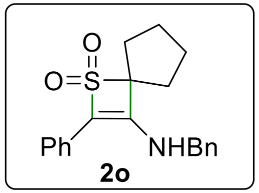

^{1}H NMR: 500 MHz in $CDCl_3$

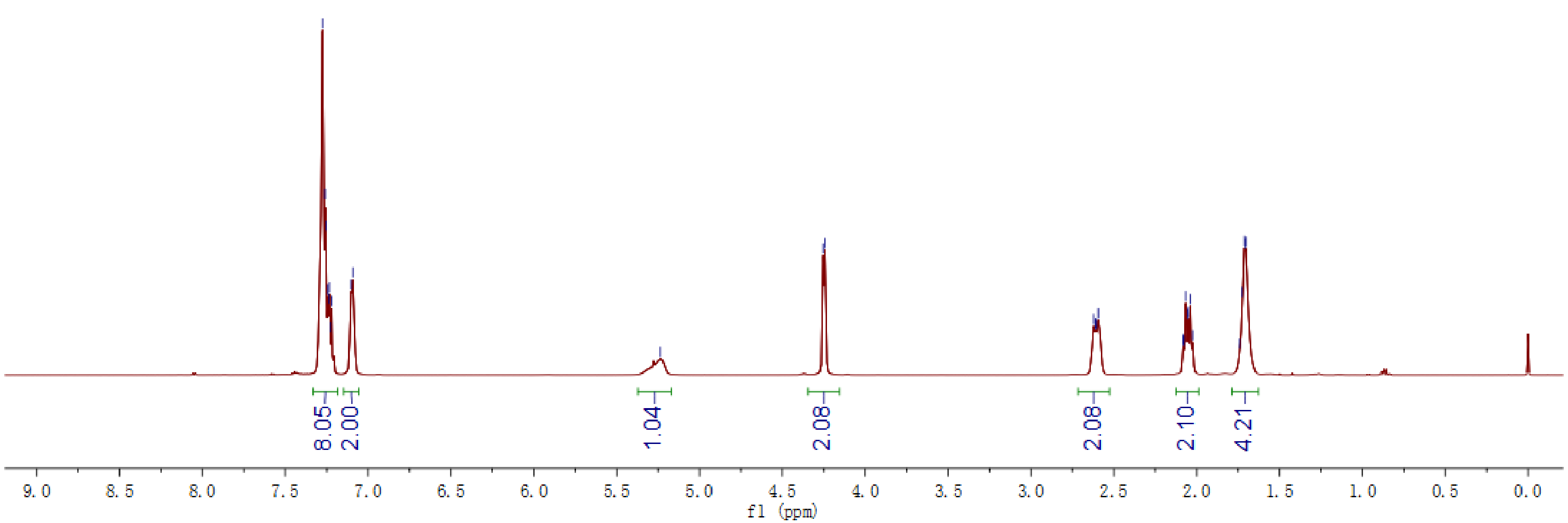

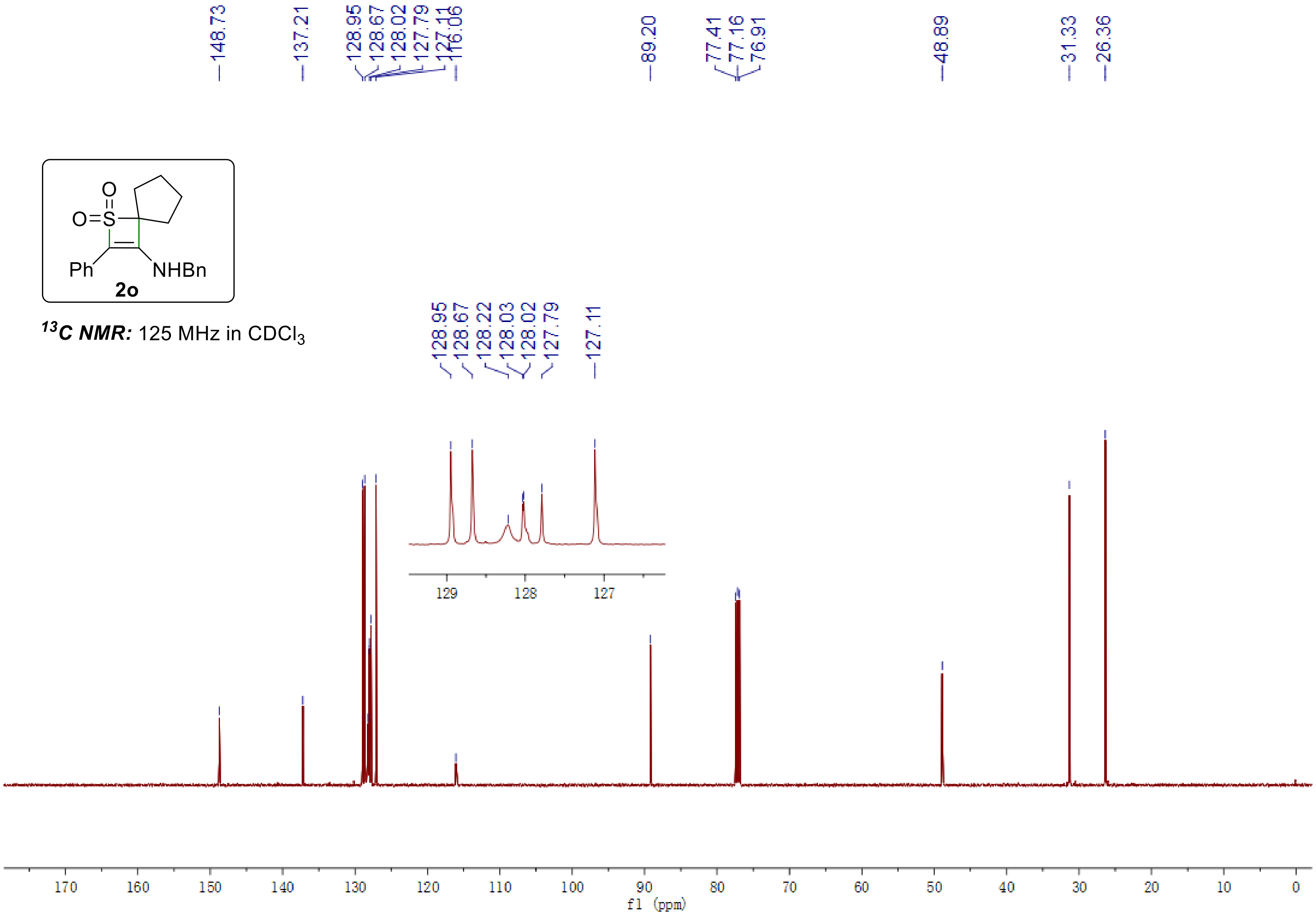
148.73
137.21
128.95
128.67
128.02
127.79
127.11
116.06
89.20
77.41
77.16
76.91
48.89
31.33
26.36
O=S
O
Ph
NHBn
2o
13C NMR: 125 MHz in CDCl3
128.95
128.67
128.22
128.03
128.02
127.79
127.11
129
128
127
170 160 150 140 130 120 110 100 90 80 70 60 50 40 30 20 10 0
f1 (ppm)

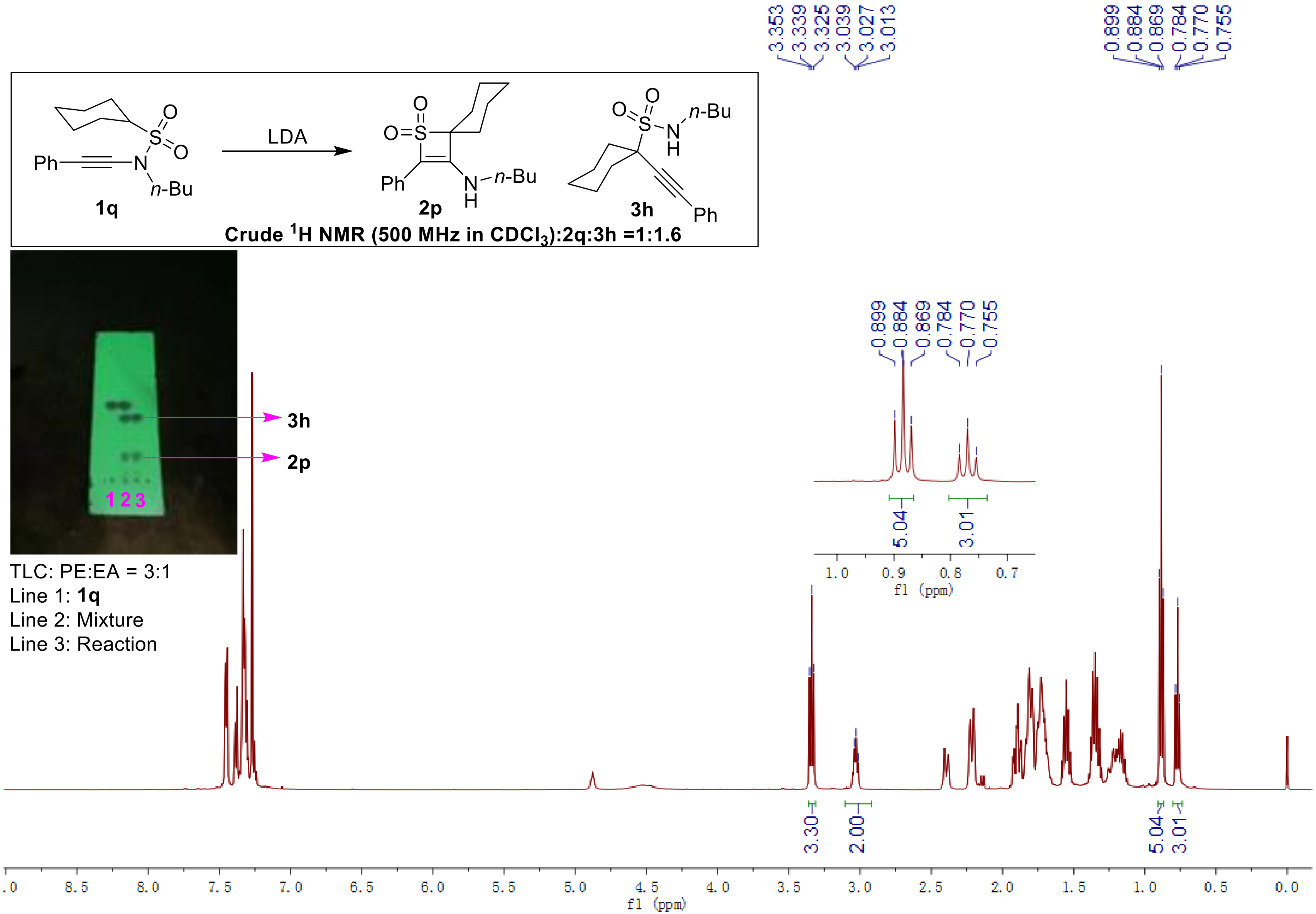
LDA
Ph
N
n-Bu
1q
2p
H
3h
Crude ^{1}H NMR (500 MHz in CDCl$_3$):2q:3h =1:1.6
3h
2p
1 2 3
TLC: PE:EA = 3:1
Line 1: **1q**
Line 2: Mixture
Line 3: Reaction
3.353
3.339
3.325
3.039
3.027
3.013
0.899
0.884
0.869
0.784
0.770
0.755
5.04
3.01
3.30
2.00
f1 (ppm)

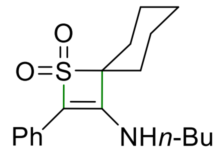

^{1}H NMR: 400 MHz in $CDCl_3$

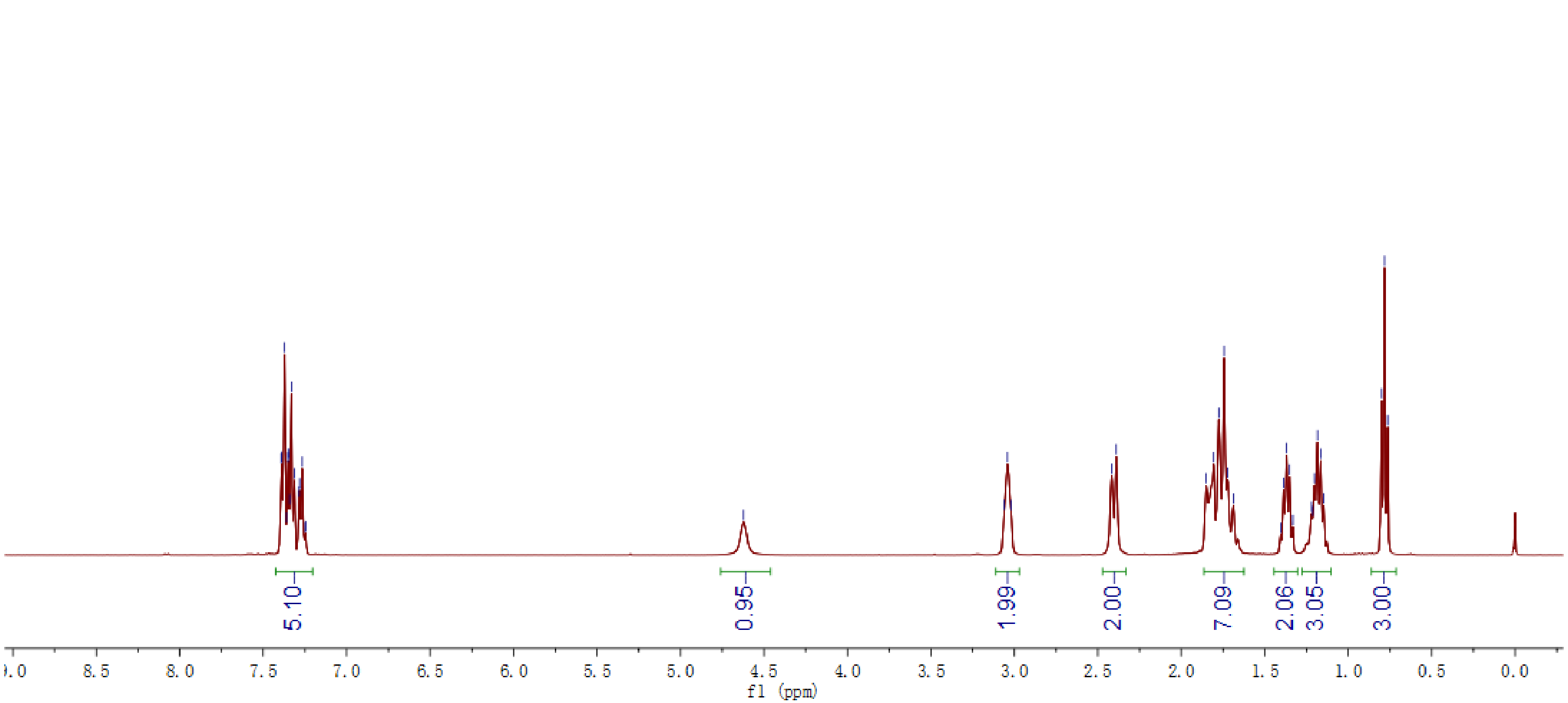

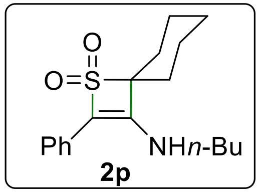

^{13}C NMR: 100 MHz in $CDCl_3$

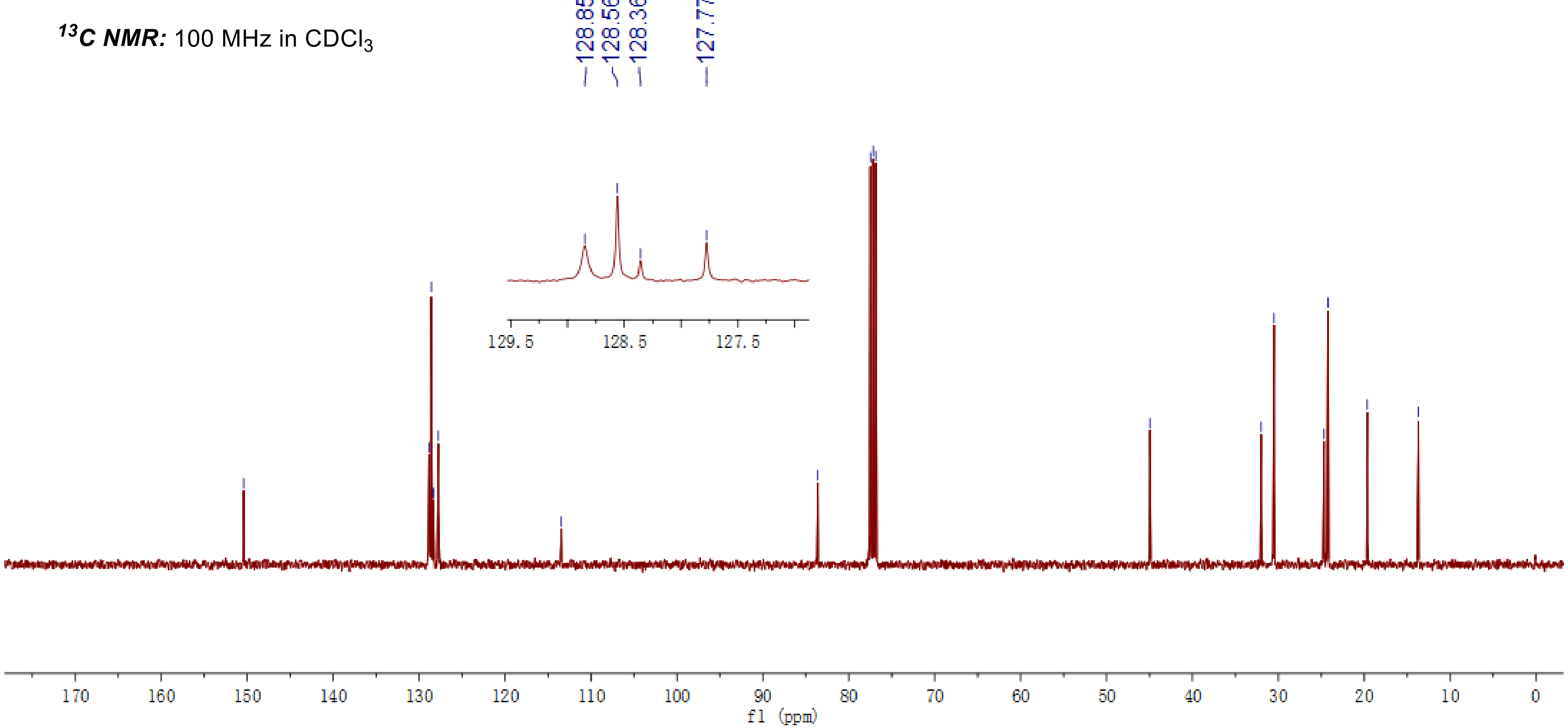

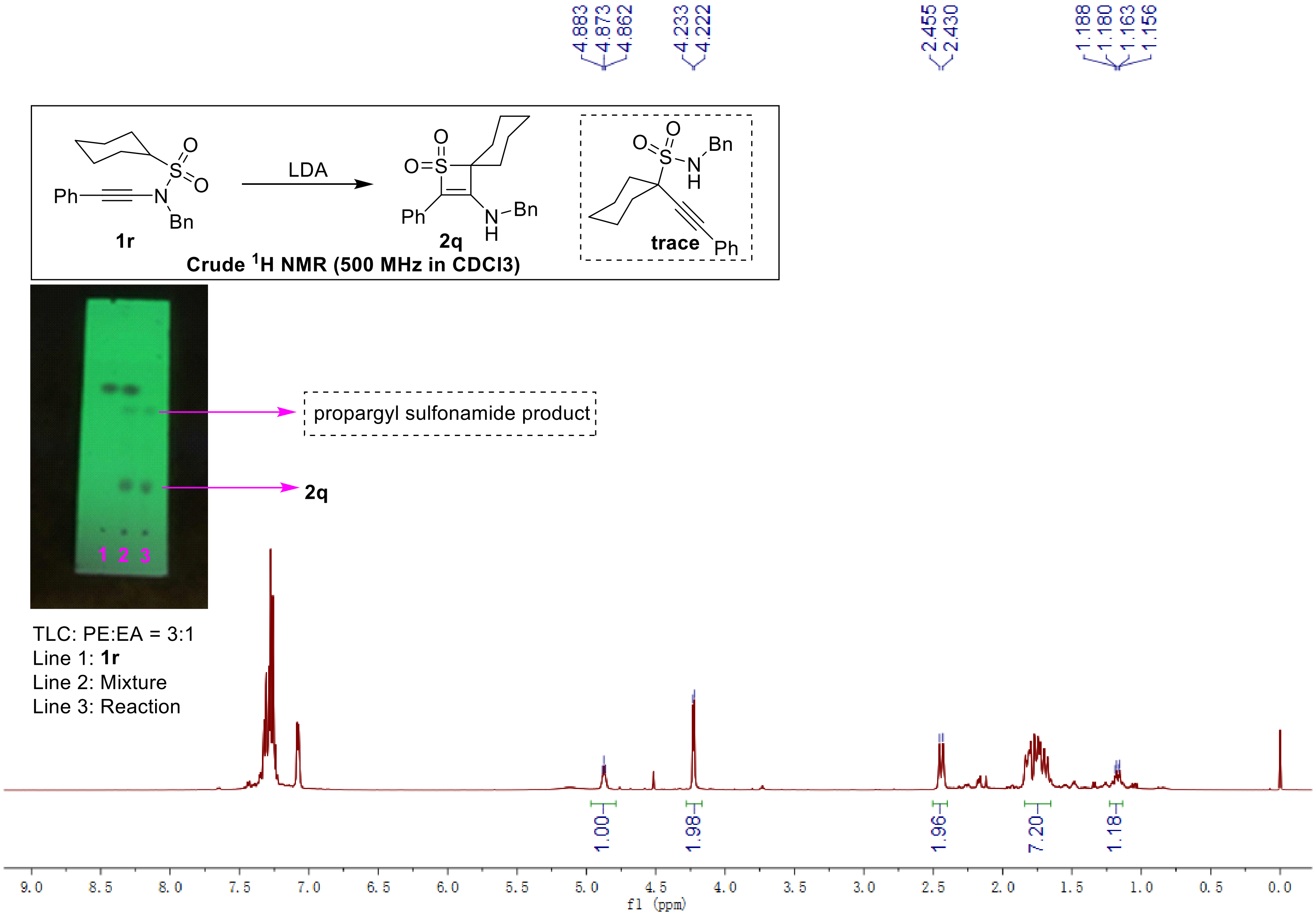

4.883
4.873
4.862
4.233
4.222
2.455
2.430
1.188
1.180
1.163
1.156
Ph
N
Bn
O
S
O
1r
LDA
O
O=S
Ph
N-Bn
H
2q
O
O
S
N
Bn
H
trace
Ph
Crude 1H NMR (500 MHz in CDCl3)
propargyl sulfonamide product
2q
1 2 3
TLC: PE:EA = 3:1
Line 1: 1r
Line 2: Mixture
Line 3: Reaction
1.00
1.98
1.96
7.20
1.18
9.0 8.5 8.0 7.5 7.0 6.5 6.0 5.5 5.0 4.5 4.0 3.5 3.0 2.5 2.0 1.5 1.0 0.5 0.0
f1 (ppm)

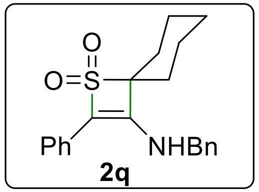

^{1}***H NMR:*** 500 MHz in $CDCl_3$

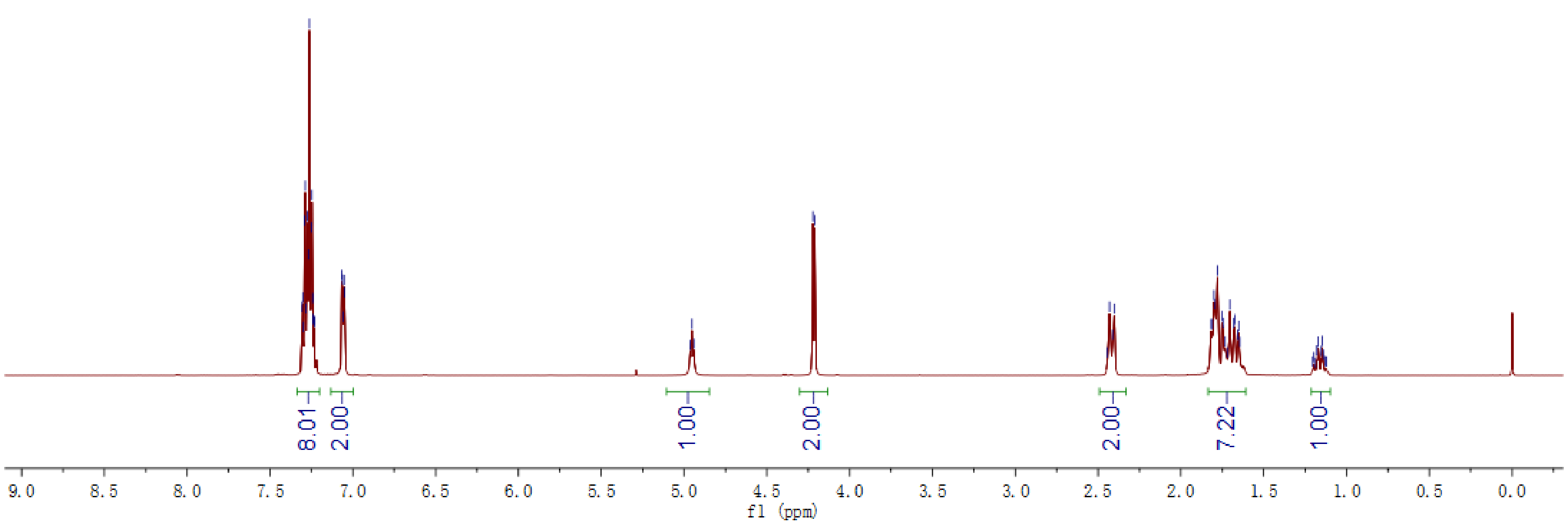

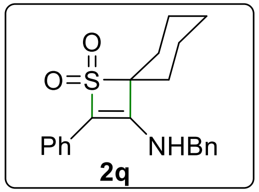

^{13}C NMR: 150 MHz in $CDCl_3$

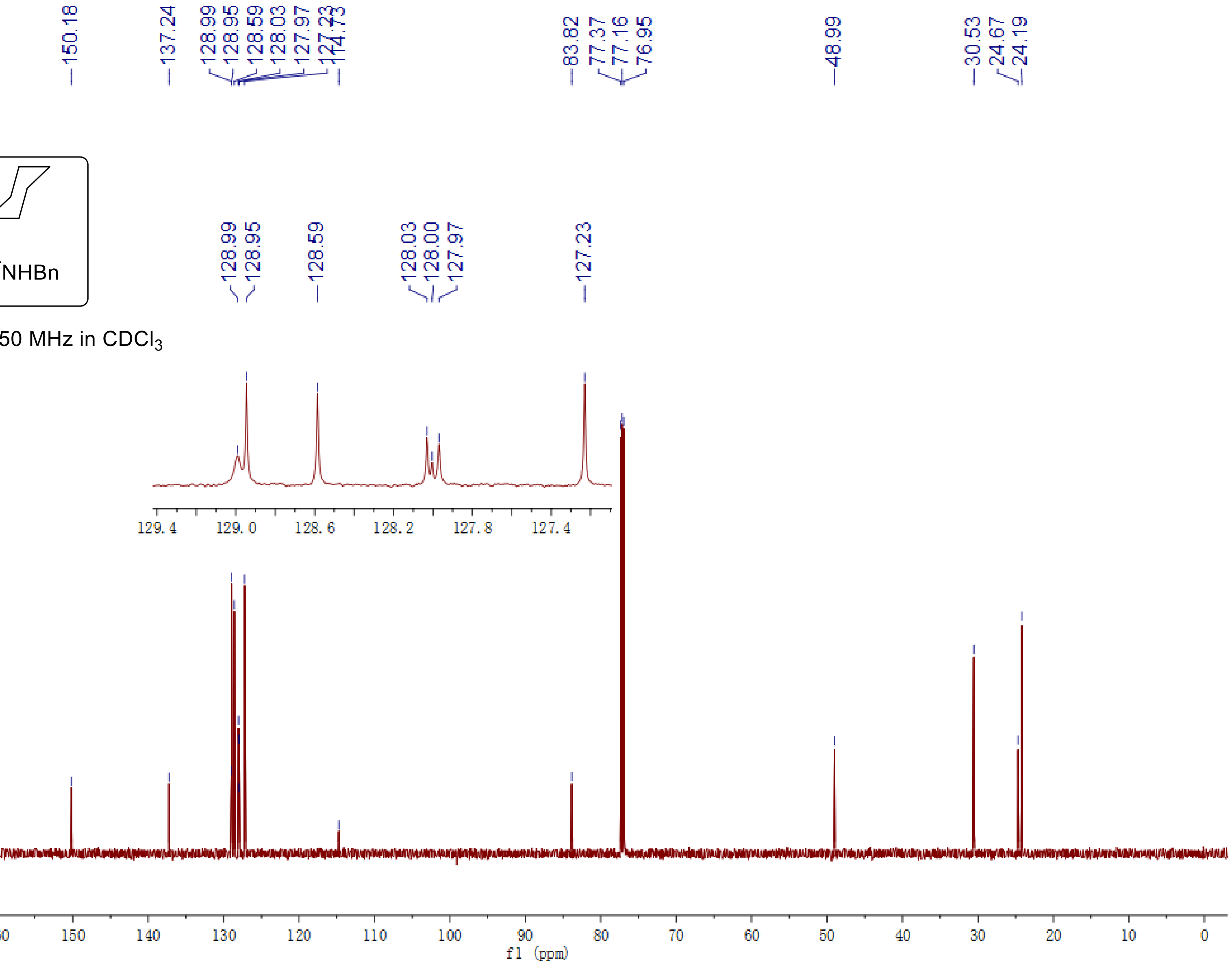

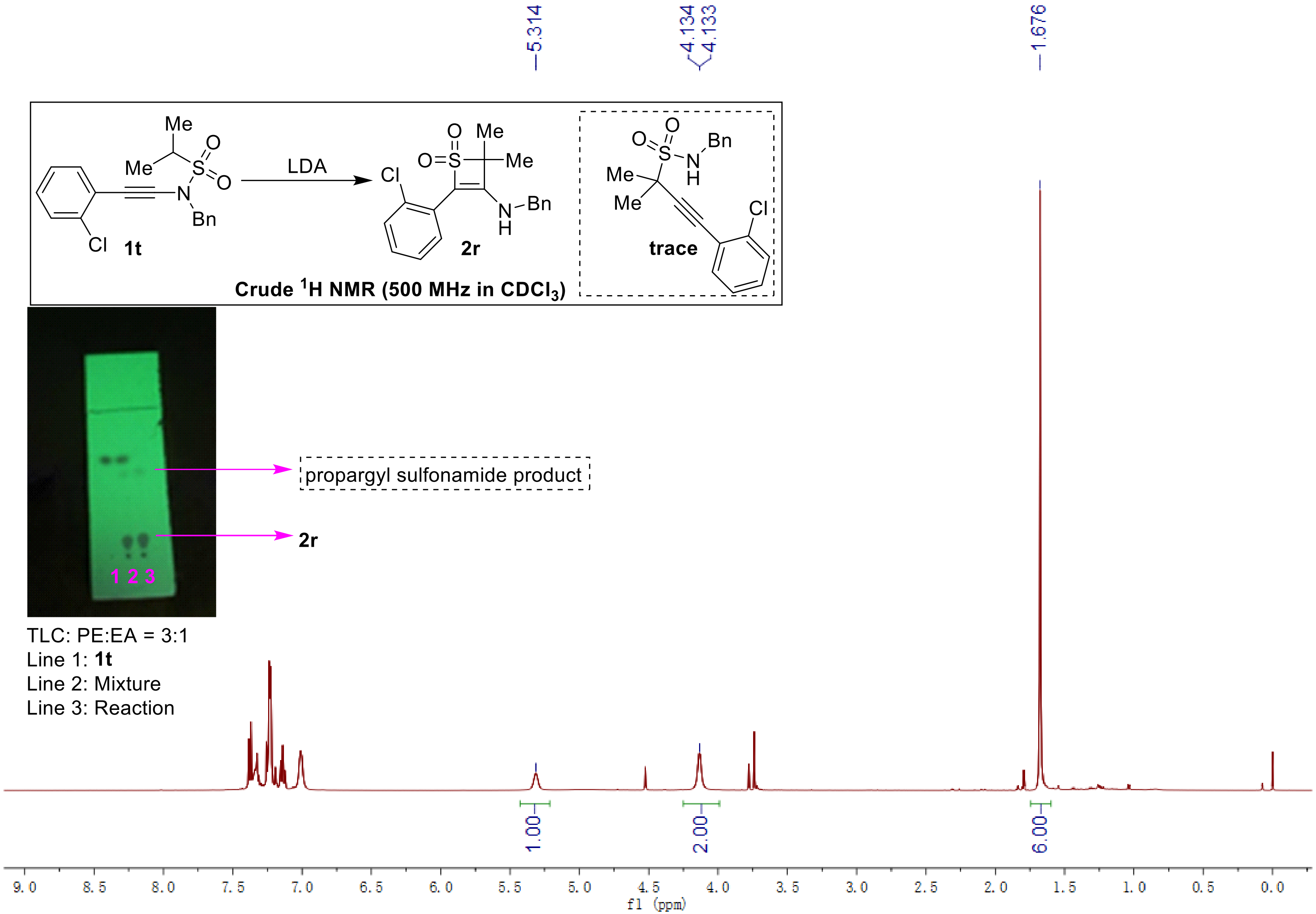
LDA
1t
2r
trace
Crude 1H NMR (500 MHz in CDCl3)
propargyl sulfonamide product
2r
1 2 3
TLC: PE:EA = 3:1
Line 1: 1t
Line 2: Mixture
Line 3: Reaction
5.314
4.134
4.133
1.676
1.00
2.00
6.00
9.0 8.5 8.0 7.5 7.0 6.5 6.0 5.5 5.0 4.5 4.0 3.5 3.0 2.5 2.0 1.5 1.0 0.5 0.0
f1 (ppm)

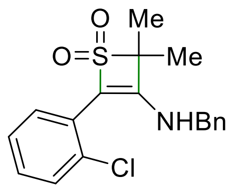

2r

^{1}H NMR: 500 MHz in $CDCl_3$

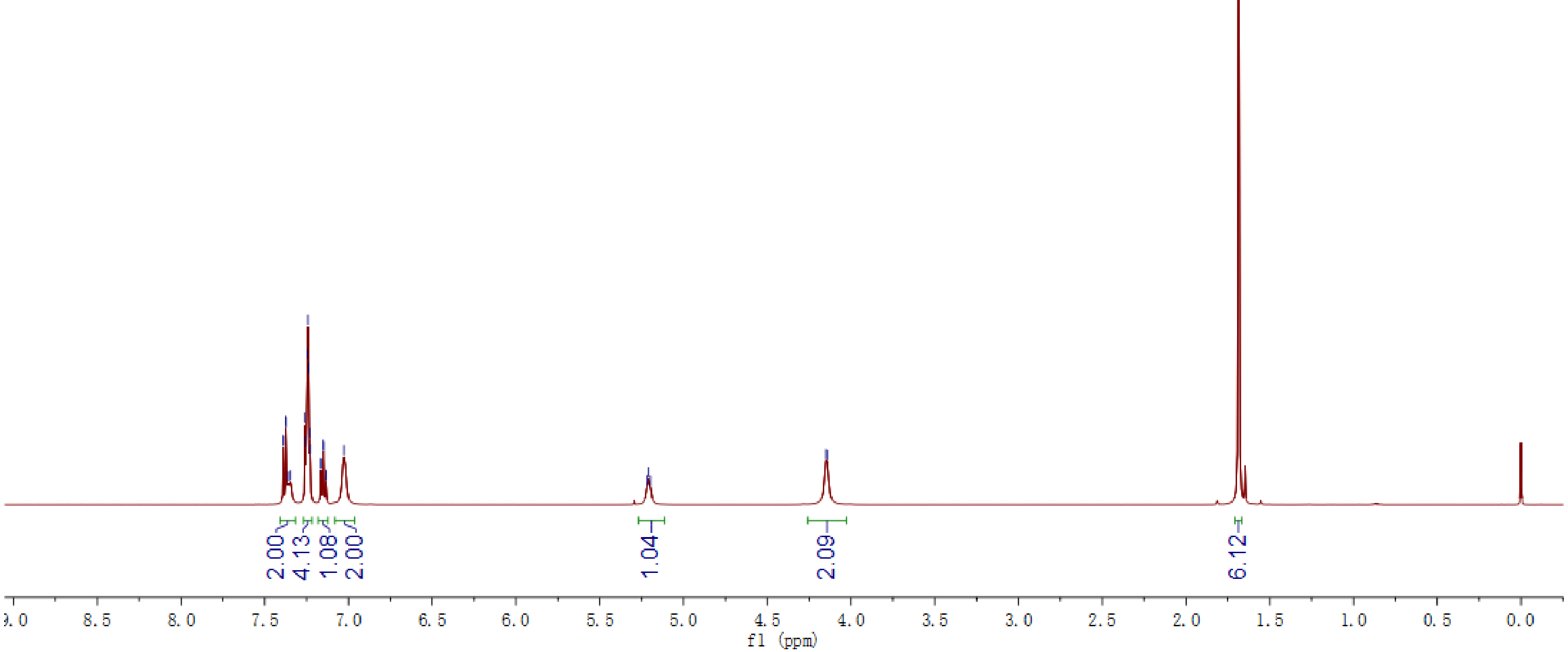

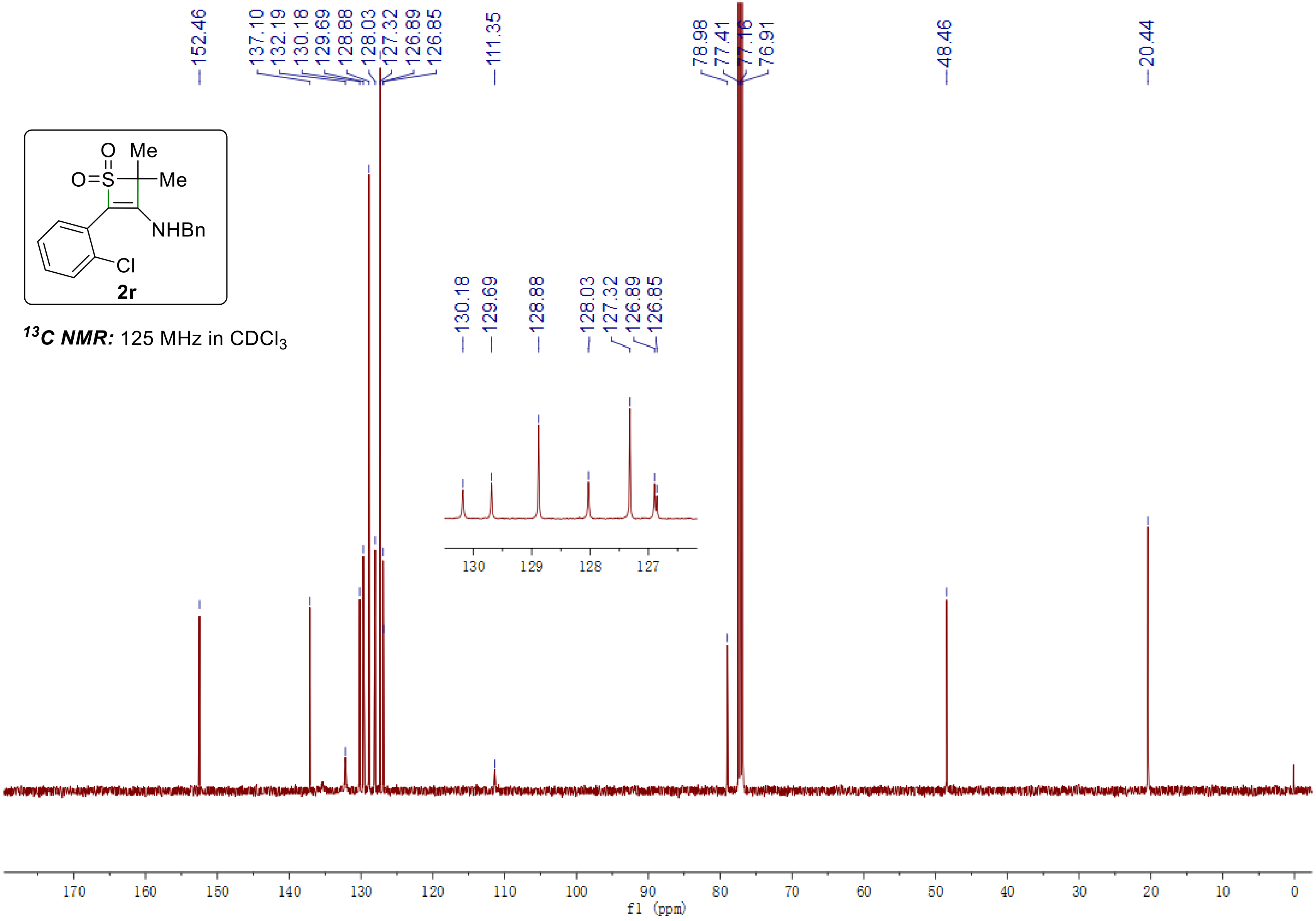

^{13}*C NMR:* 125 MHz in $CDCl_3$

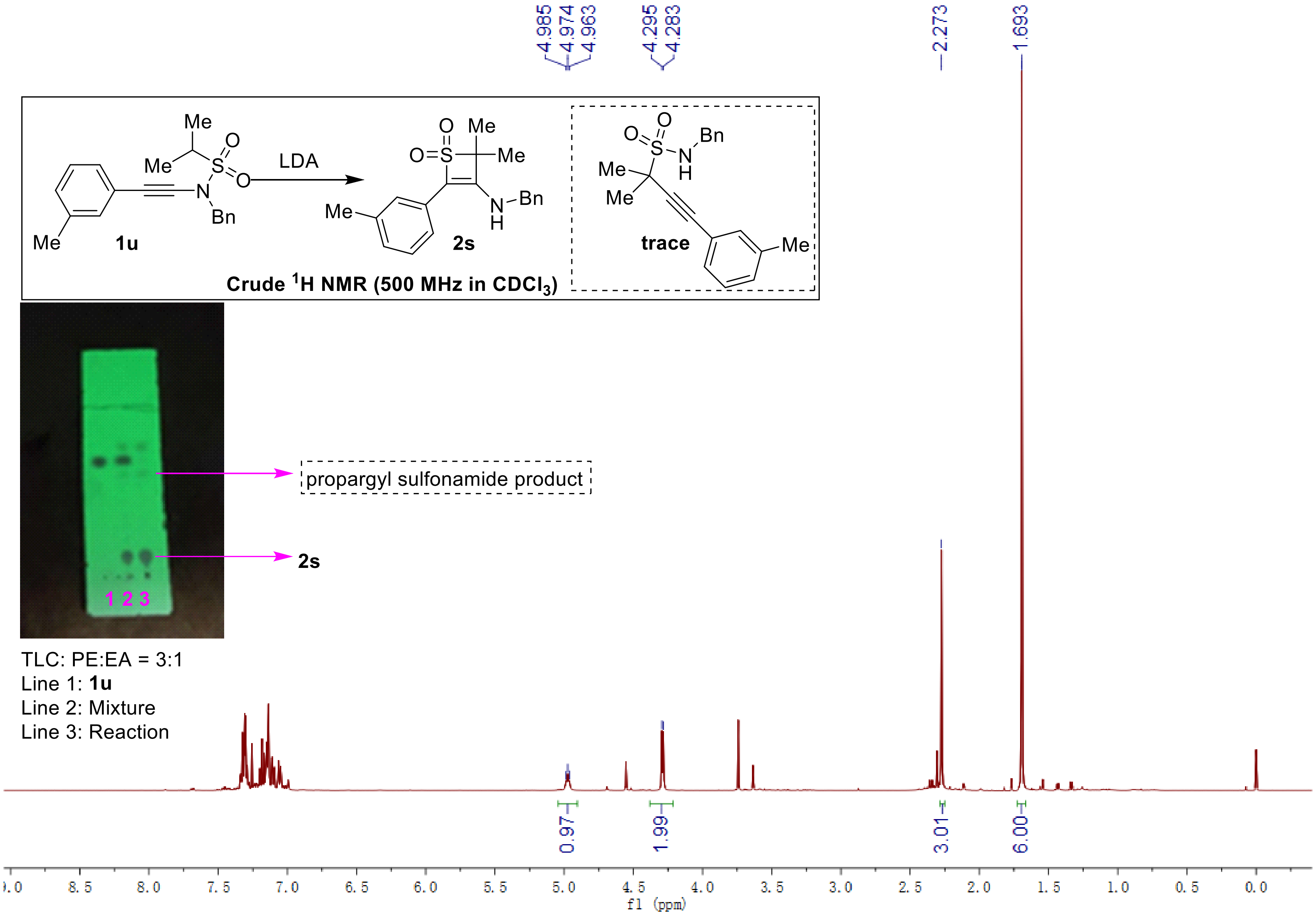

LDA
1u
2s
trace
Crude 1H NMR (500 MHz in CDCl3)
propargyl sulfonamide product
2s
1 2 3
TLC: PE:EA = 3:1
Line 1: 1u
Line 2: Mixture
Line 3: Reaction
4.985
4.974
4.963
4.295
4.283
2.273
1.693
0.97
1.99
3.01
6.00
f1 (ppm)

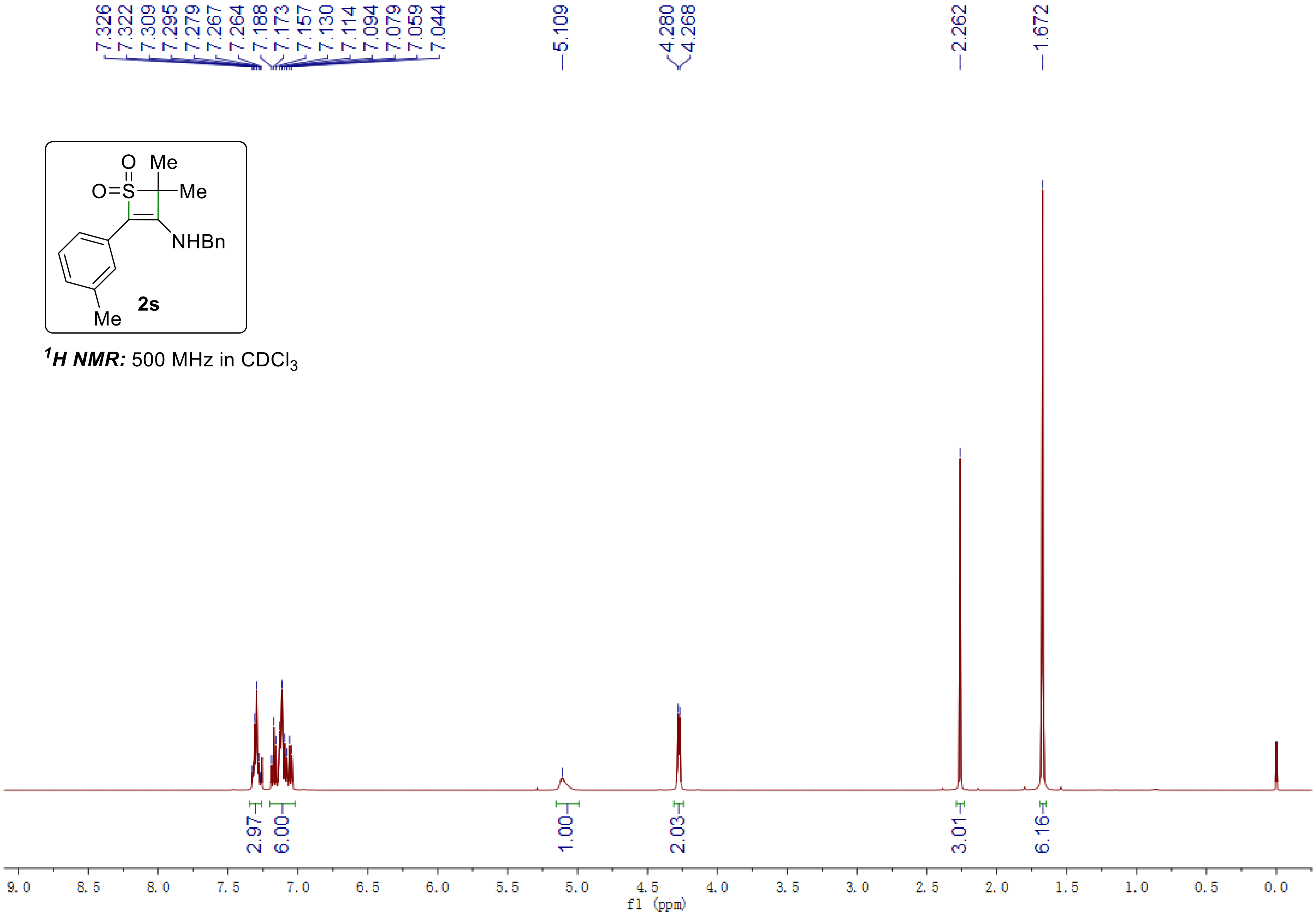

^{1}H NMR: 500 MHz in $CDCl_3$

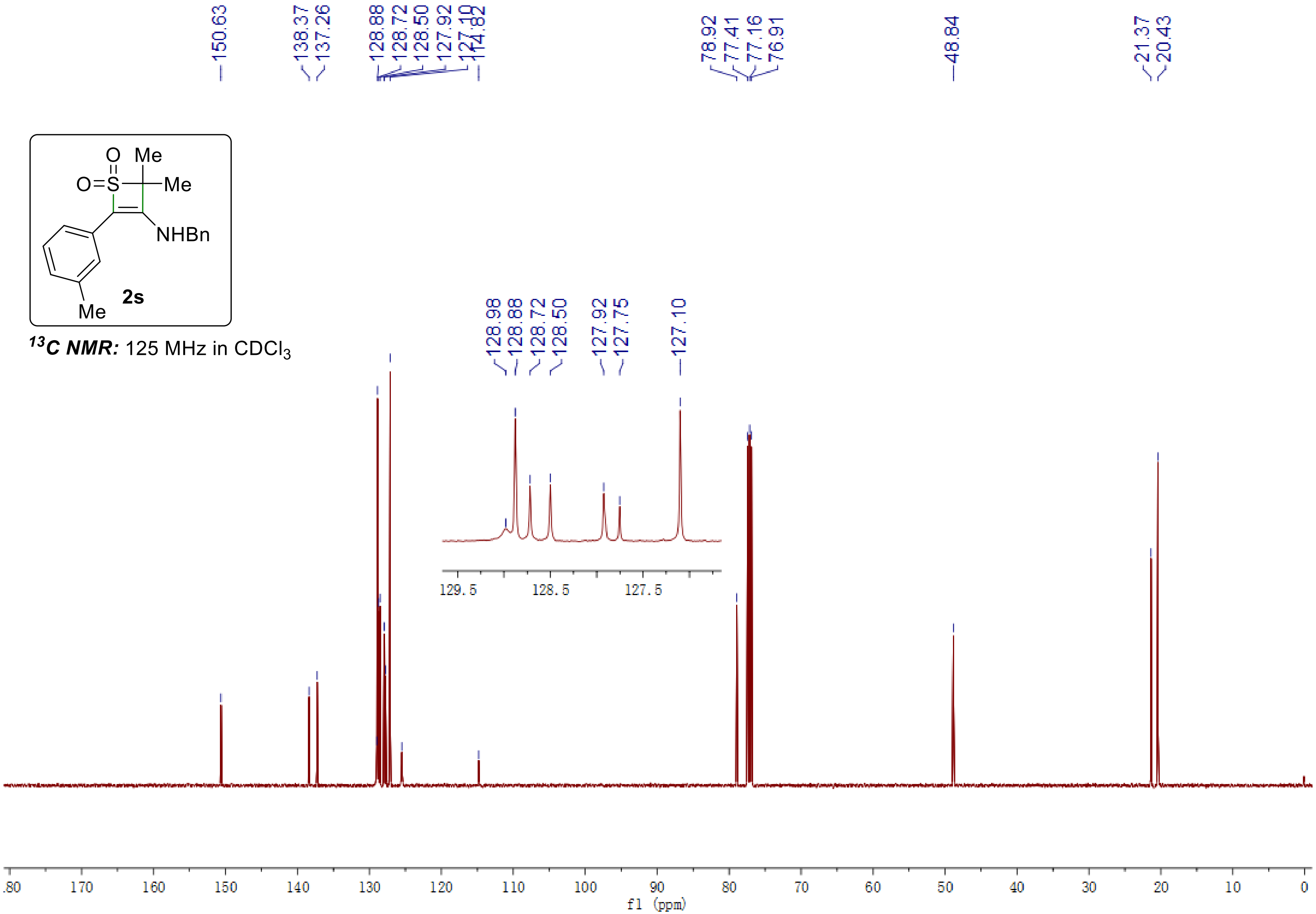

13C NMR: 125 MHz in $CDCl_3$

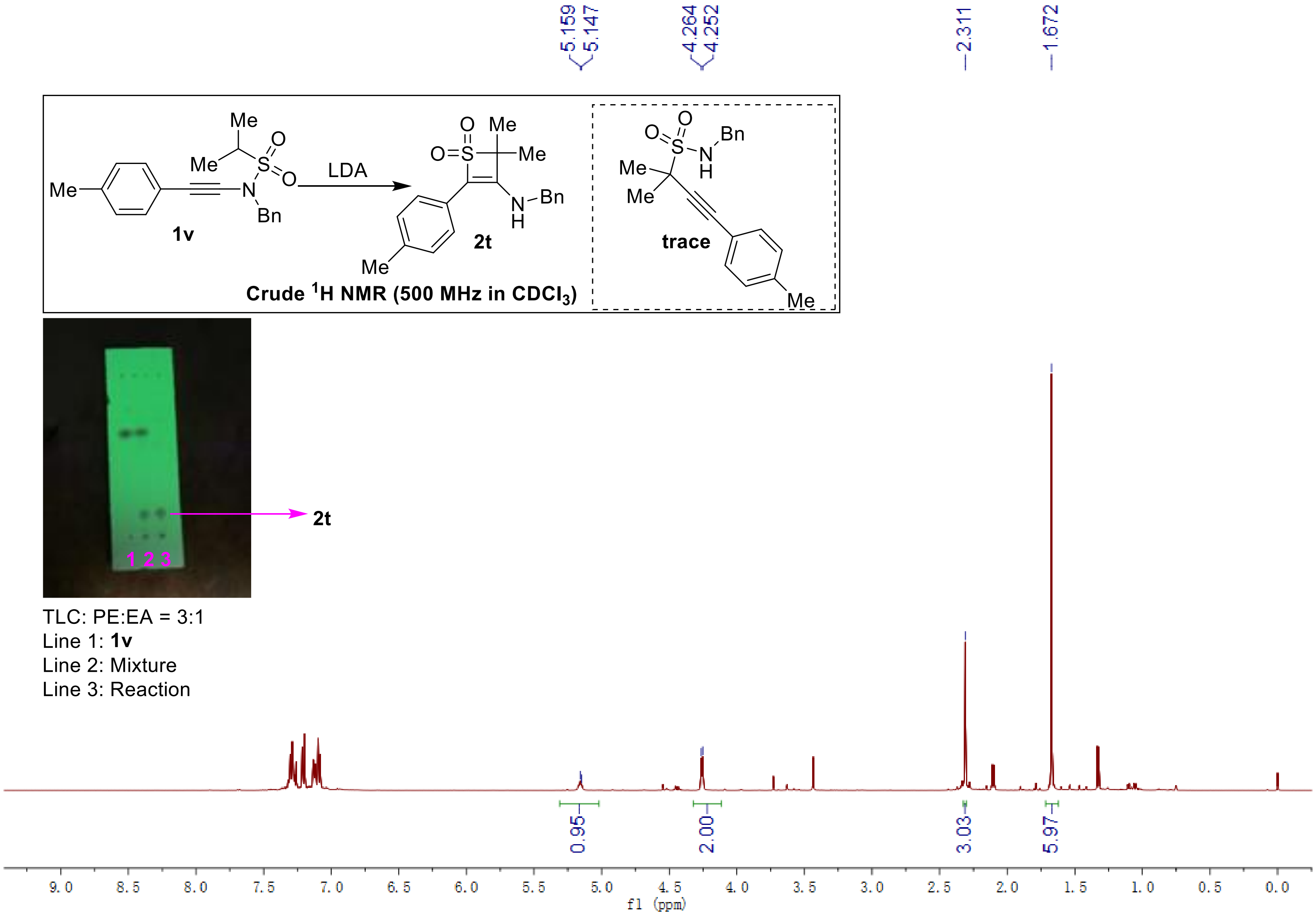
1v
LDA
2t
trace
Crude 1H NMR (500 MHz in CDCl3)
1 2 3
2t
TLC: PE:EA = 3:1
Line 1: 1v
Line 2: Mixture
Line 3: Reaction
5.159
5.147
4.264
4.252
2.311
1.672
0.95
2.00
3.03
5.97
f1 (ppm)

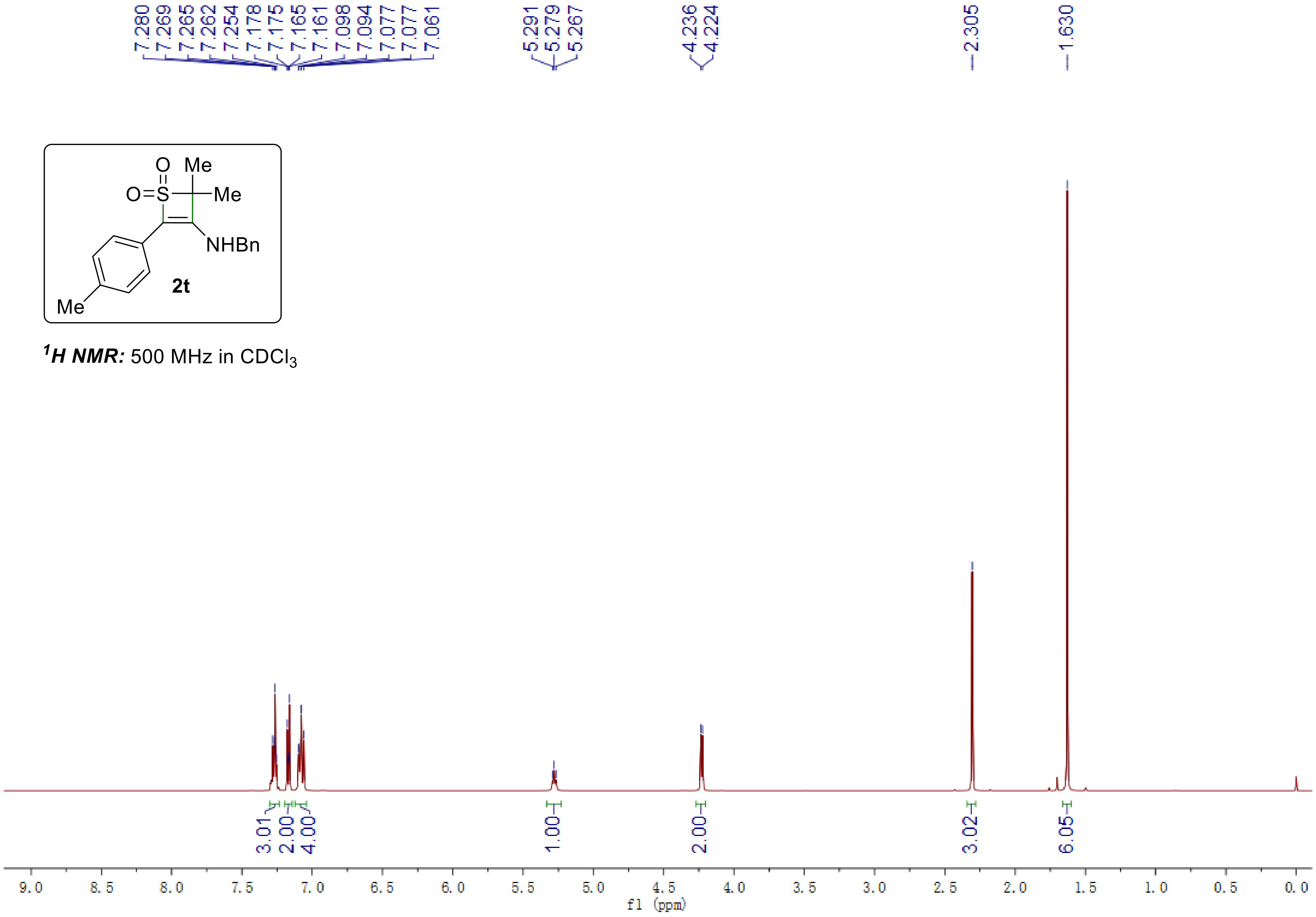

7.280
7.269
7.265
7.262
7.254
7.178
7.175
7.165
7.161
7.098
7.094
7.077
7.077
7.061
5.291
5.279
5.267
4.236
4.224
2.305
1.630
O
Me
O=S
Me
NHBn
2t
Me
^{1}H NMR: 500 MHz in CDCl$_3$
3.01
2.00
4.00
1.00
2.00
3.02
6.05
9.0
8.5
8.0
7.5
7.0
6.5
6.0
5.5
5.0
4.5
4.0
3.5
3.0
2.5
2.0
1.5
1.0
0.5
0.0
f1 (ppm)

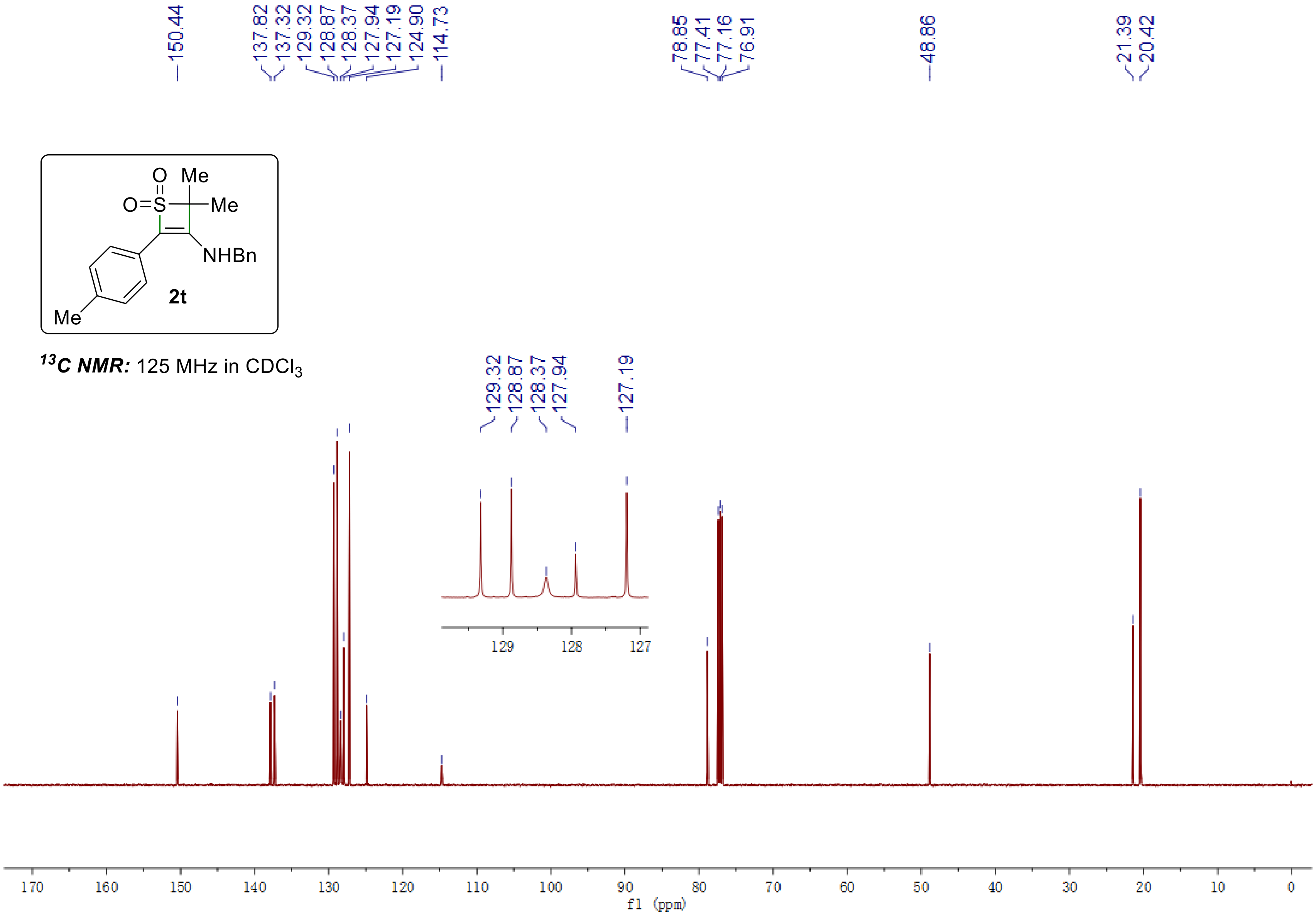

150.44
137.82
137.32
129.32
128.87
128.37
127.94
127.19
124.90
114.73
78.85
77.41
77.16
76.91
48.86
21.39
20.42
O
Me
O=S
Me
NHBn
Me
2t
^{13}C NMR: 125 MHz in CDCl$_3$
129.32
128.87
128.37
127.94
127.19
129
128
127
170
160
150
140
130
120
110
100
90
80
70
60
50
40
30
20
10
0
f1 (ppm)

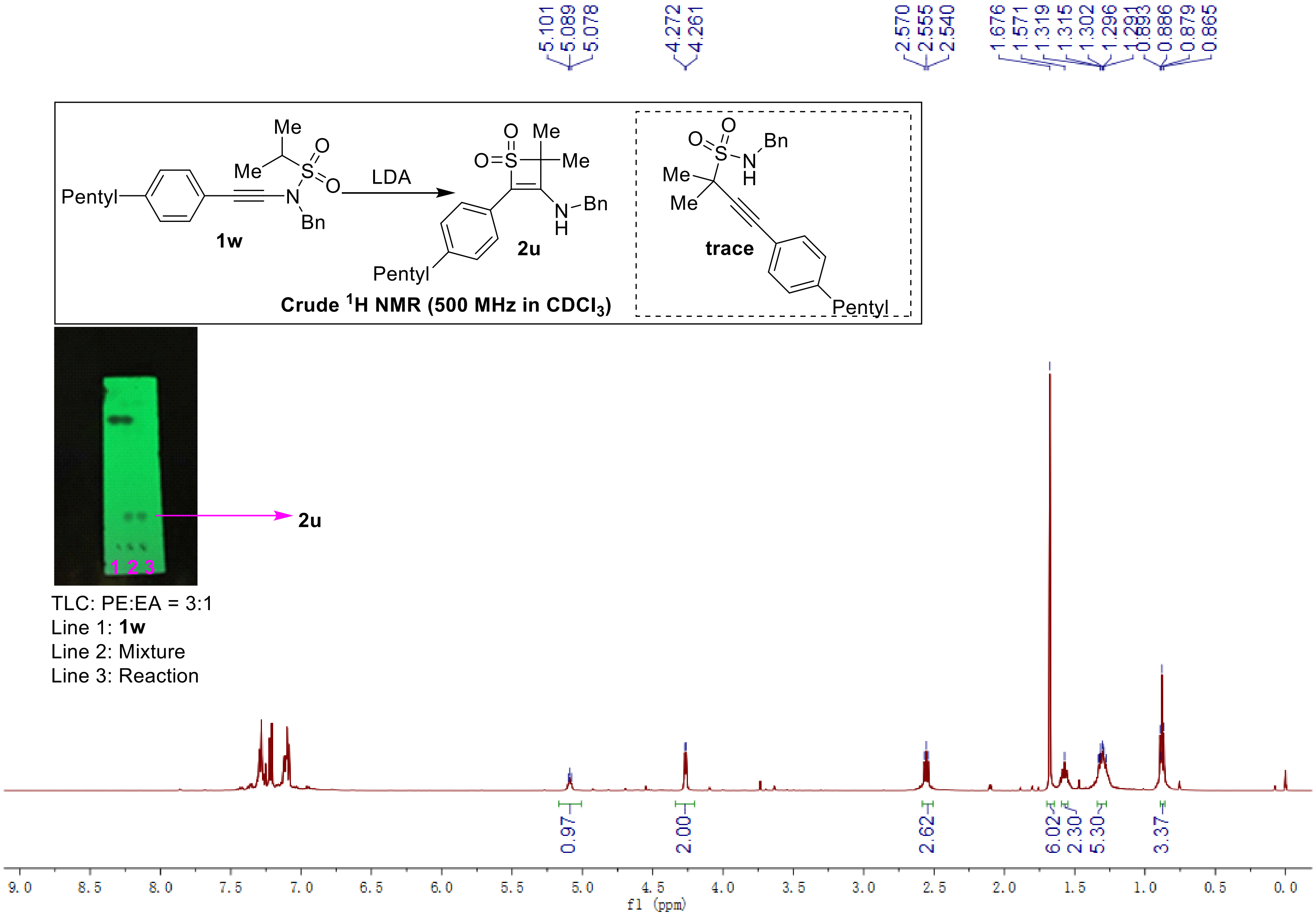
5.101
5.089
5.078
4.272
4.261
2.570
2.555
2.540
1.676
1.571
1.319
1.315
1.302
1.296
1.291
0.893
0.886
0.879
0.865
Pentyl
Me
Me
O
S
O
N
Bn
1w
LDA
O
O=S
Me
Me
N–Bn
H
2u
Pentyl
Crude ¹H NMR (500 MHz in CDCl3)
O
O
S
N
Bn
H
Me
Me
trace
Pentyl
1 2 3
2u
TLC: PE:EA = 3:1
Line 1: 1w
Line 2: Mixture
Line 3: Reaction
0.97
2.00
2.62
6.02
2.30
5.30
3.37
9.0
8.5
8.0
7.5
7.0
6.5
6.0
5.5
5.0
4.5
4.0
3.5
3.0
2.5
2.0
1.5
1.0
0.5
0.0
f1 (ppm)

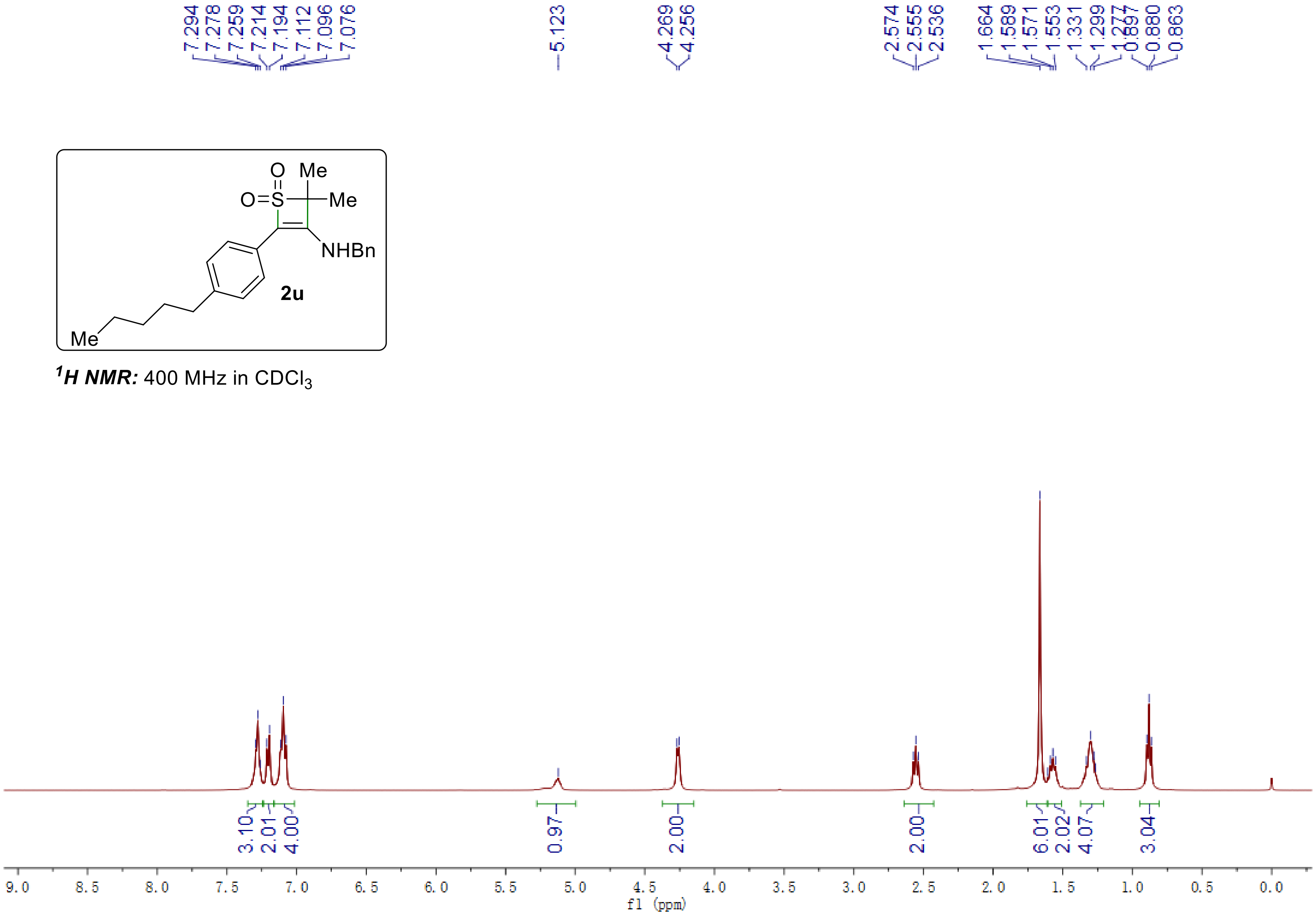

^{1}H NMR: 400 MHz in $CDCl_3$

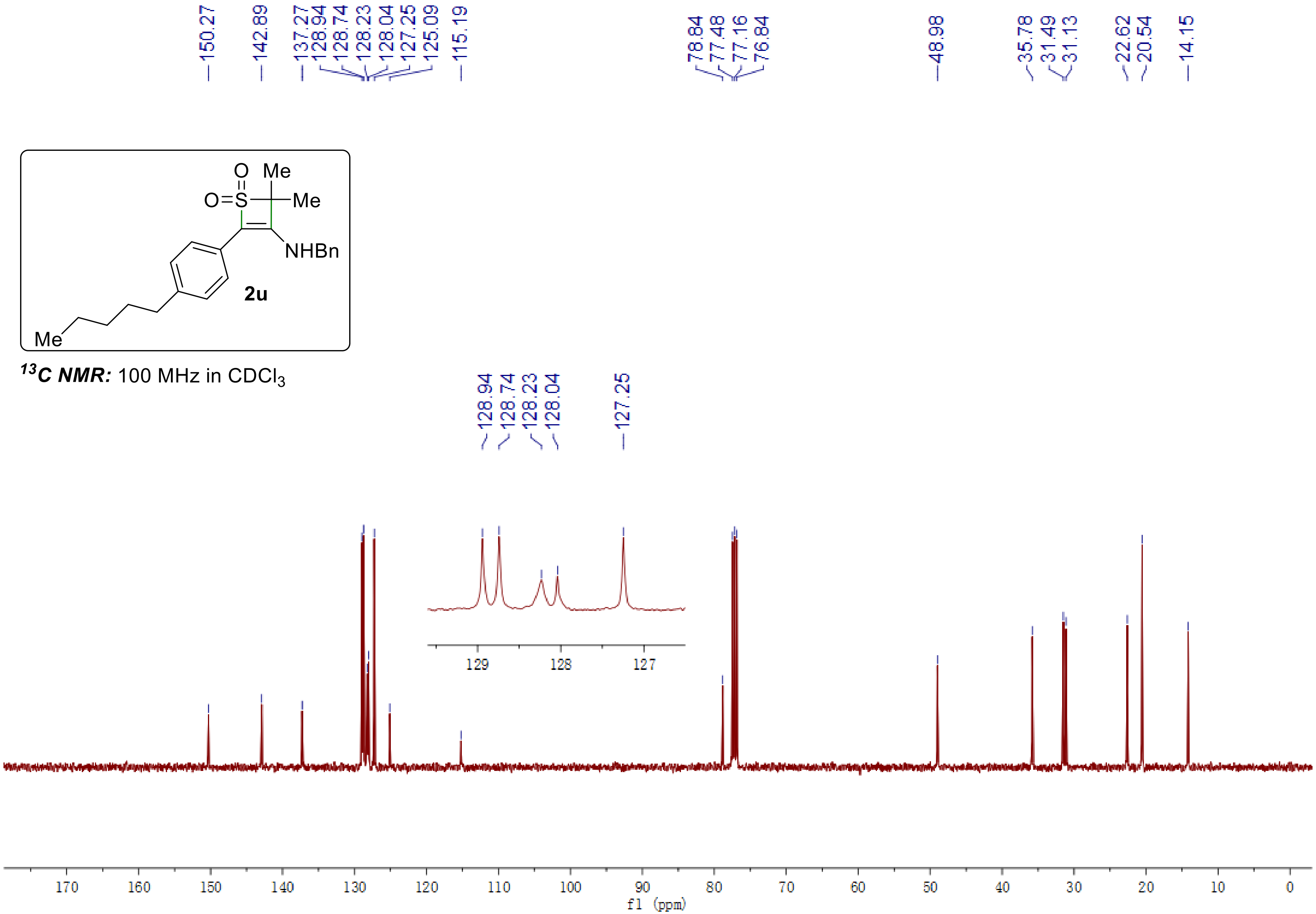
150.27
142.89
137.27
128.94
128.74
128.23
128.04
127.25
125.09
115.19
78.84
77.48
77.16
76.84
48.98
35.78
31.49
31.13
22.62
20.54
14.15
O
Me
O=S
Me
NHBn
2u
Me
^{13}C NMR: 100 MHz in CDCl$_3$
129
128
127
170
160
150
140
130
120
110
100
90
80
70
60
50
40
30
20
10
0
f1 (ppm)

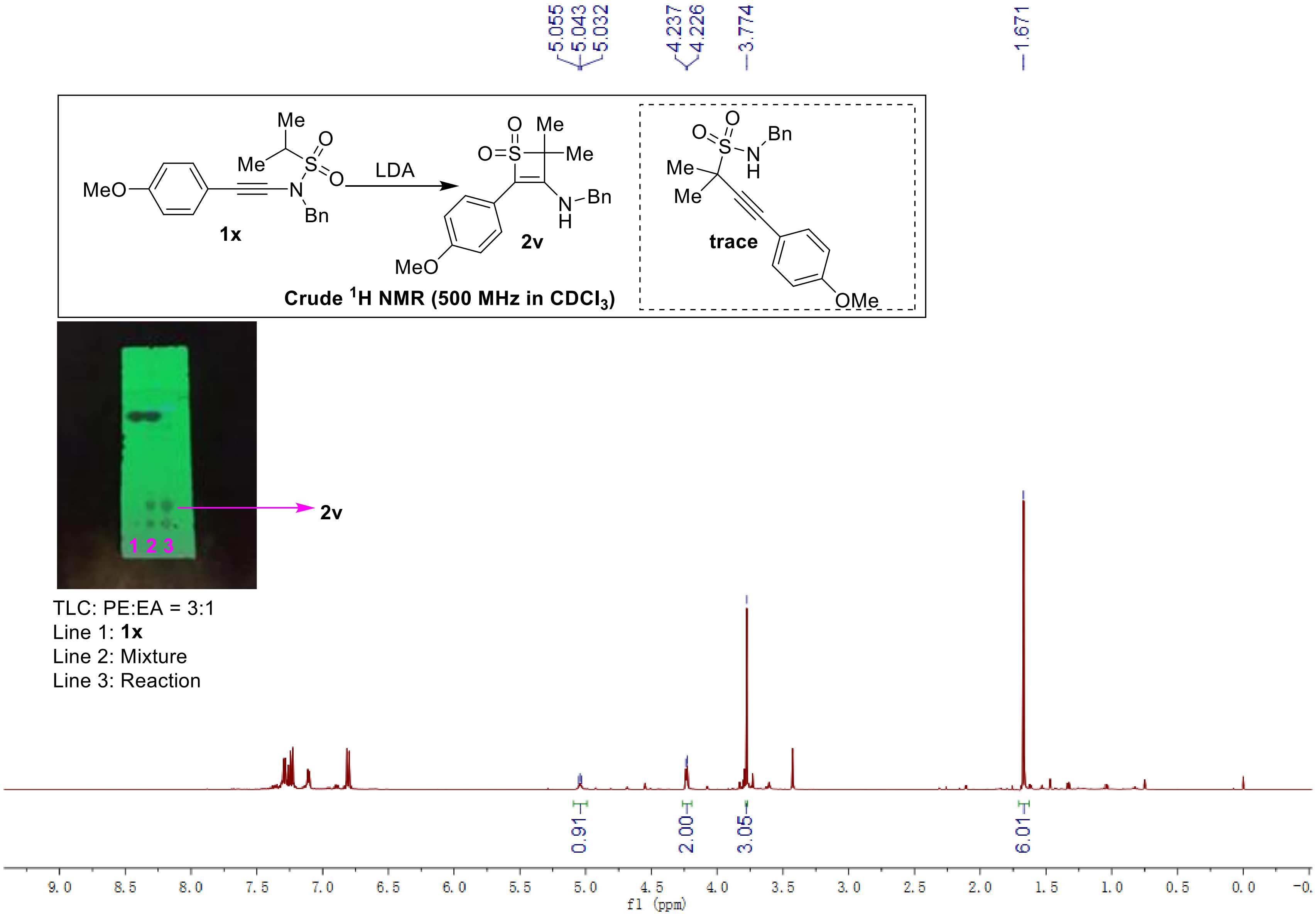

5.055
5.043
5.032
4.237
4.226
3.774
1.671
MeO
Me
Me
O
S
O
N
Bn
1x
LDA
O
O=S
Me
Me
N
H
Bn
MeO
2v
O
O
S
N
H
Bn
Me
Me
trace
OMe
Crude ¹H NMR (500 MHz in CDCl3)
1 2 3
2v
TLC: PE:EA = 3:1
Line 1: 1x
Line 2: Mixture
Line 3: Reaction
0.91
2.00
3.05
6.01
9.0 8.5 8.0 7.5 7.0 6.5 6.0 5.5 5.0 4.5 4.0 3.5 3.0 2.5 2.0 1.5 1.0 0.5 0.0 -0.
f1 (ppm)

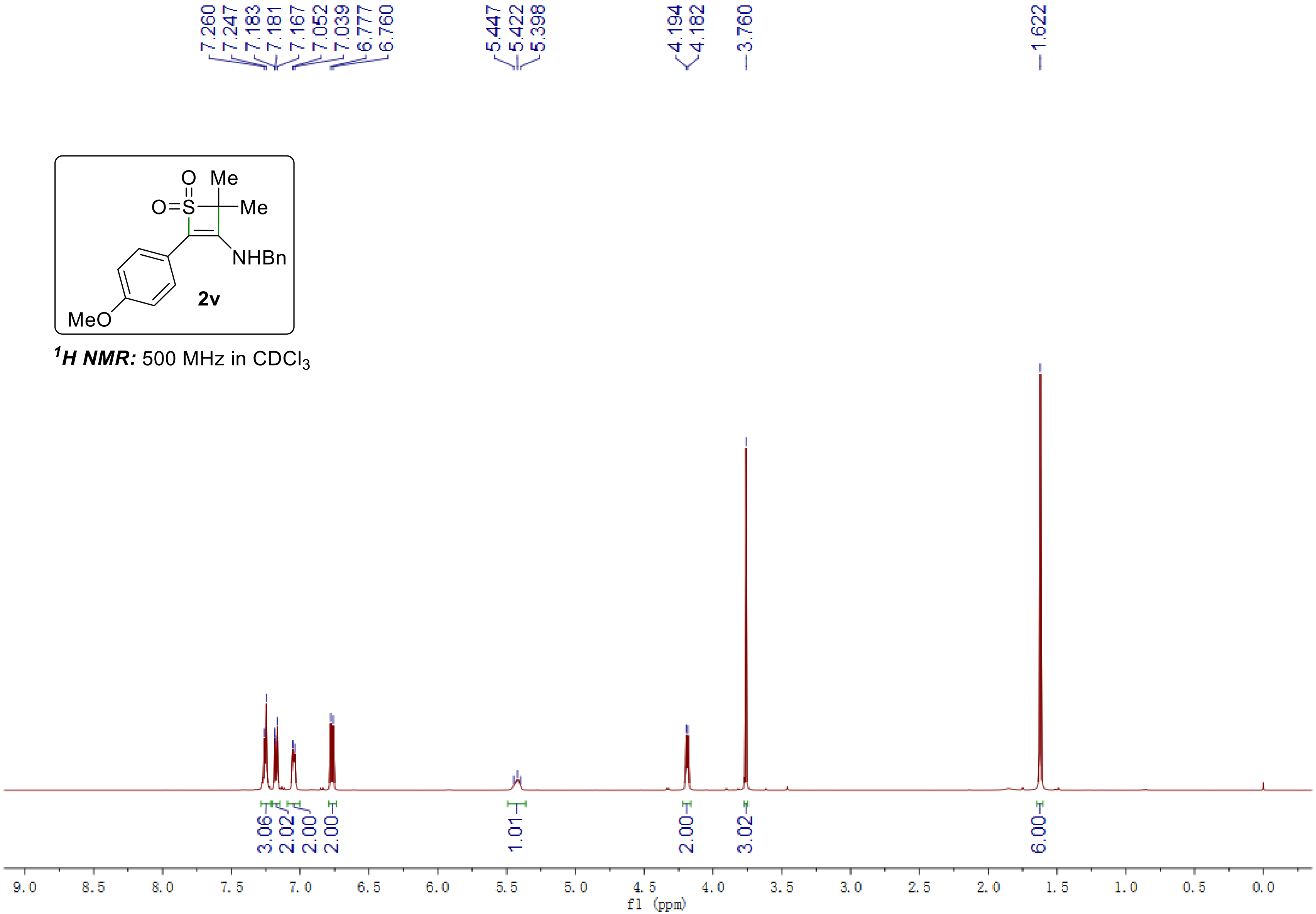
7.260
7.247
7.183
7.181
7.167
7.052
7.039
6.777
6.760
5.447
5.422
5.398
4.194
4.182
3.760
1.622
O
O=S
Me
Me
NHBn
MeO
2v
1H NMR: 500 MHz in CDCl3
3.06
2.02
2.00
2.00
1.01
2.00
3.02
6.00
9.0
8.5
8.0
7.5
7.0
6.5
6.0
5.5
5.0
4.5
4.0
3.5
3.0
2.5
2.0
1.5
1.0
0.5
0.0
f1 (ppm)

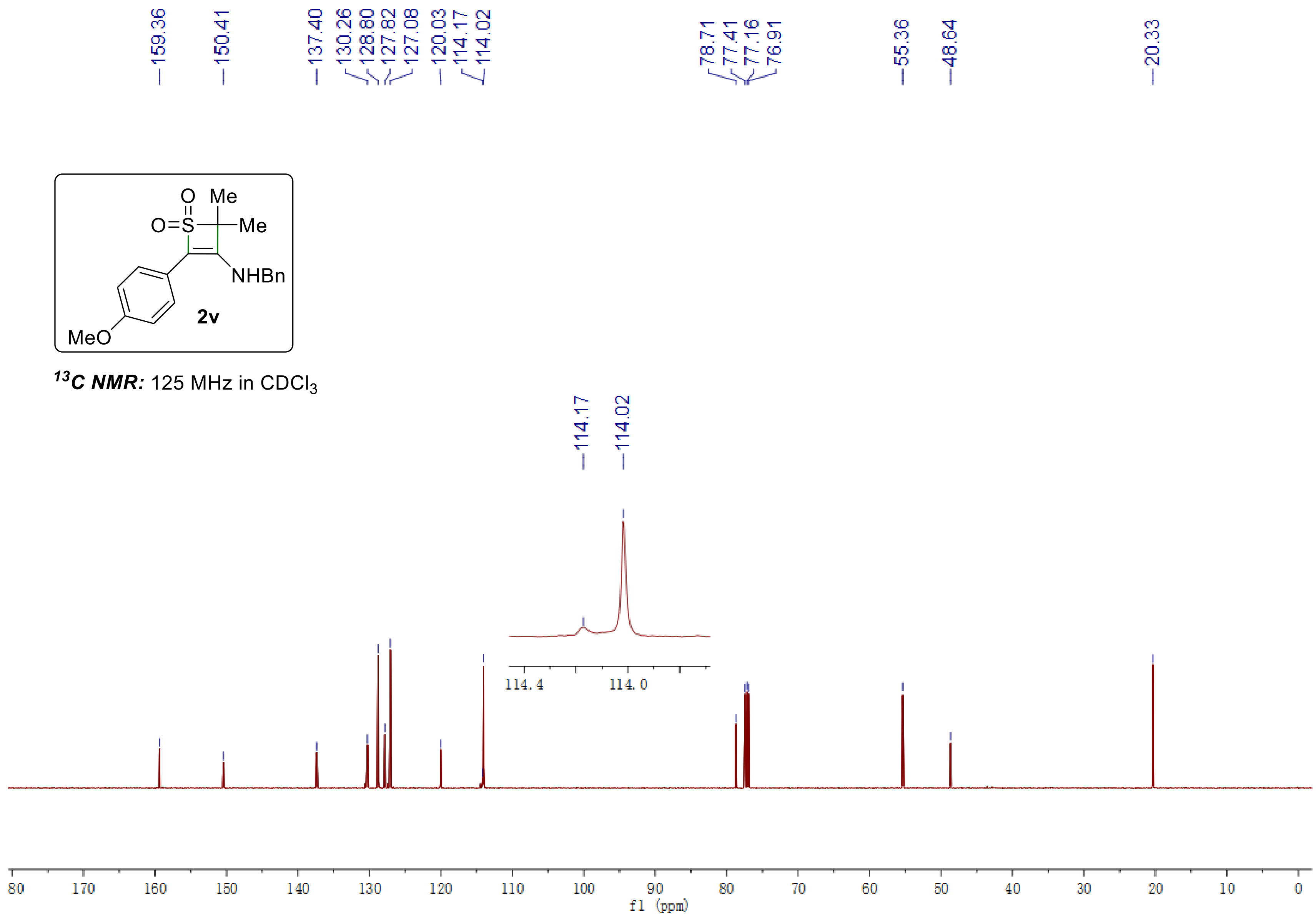
159.36
150.41
137.40
130.26
128.80
127.82
127.08
120.03
114.17
114.02
78.71
77.41
77.16
76.91
55.36
48.64
20.33
O
Me
O=S
Me
NHBn
2v
MeO
13C NMR: 125 MHz in CDCl3
114.17
114.02
114.4
114.0
f1 (ppm)

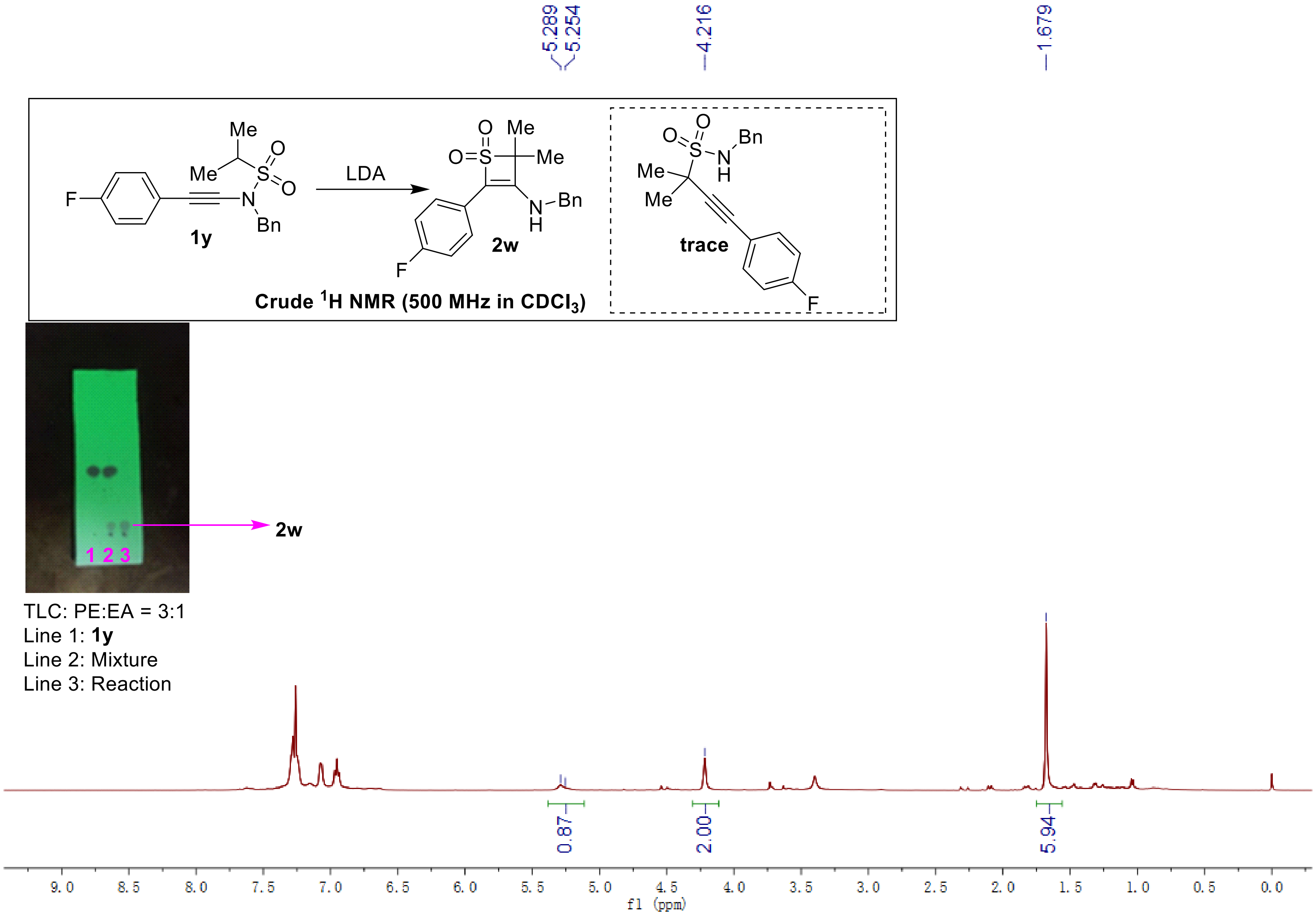

5.289
5.254
4.216
1.679
1y
LDA
2w
trace
Crude ¹H NMR (500 MHz in CDCl₃)
2w
1 2 3
TLC: PE:EA = 3:1
Line 1: 1y
Line 2: Mixture
Line 3: Reaction
0.87
2.00
5.94
9.0 8.5 8.0 7.5 7.0 6.5 6.0 5.5 5.0 4.5 4.0 3.5 3.0 2.5 2.0 1.5 1.0 0.5 0.0
f1 (ppm)

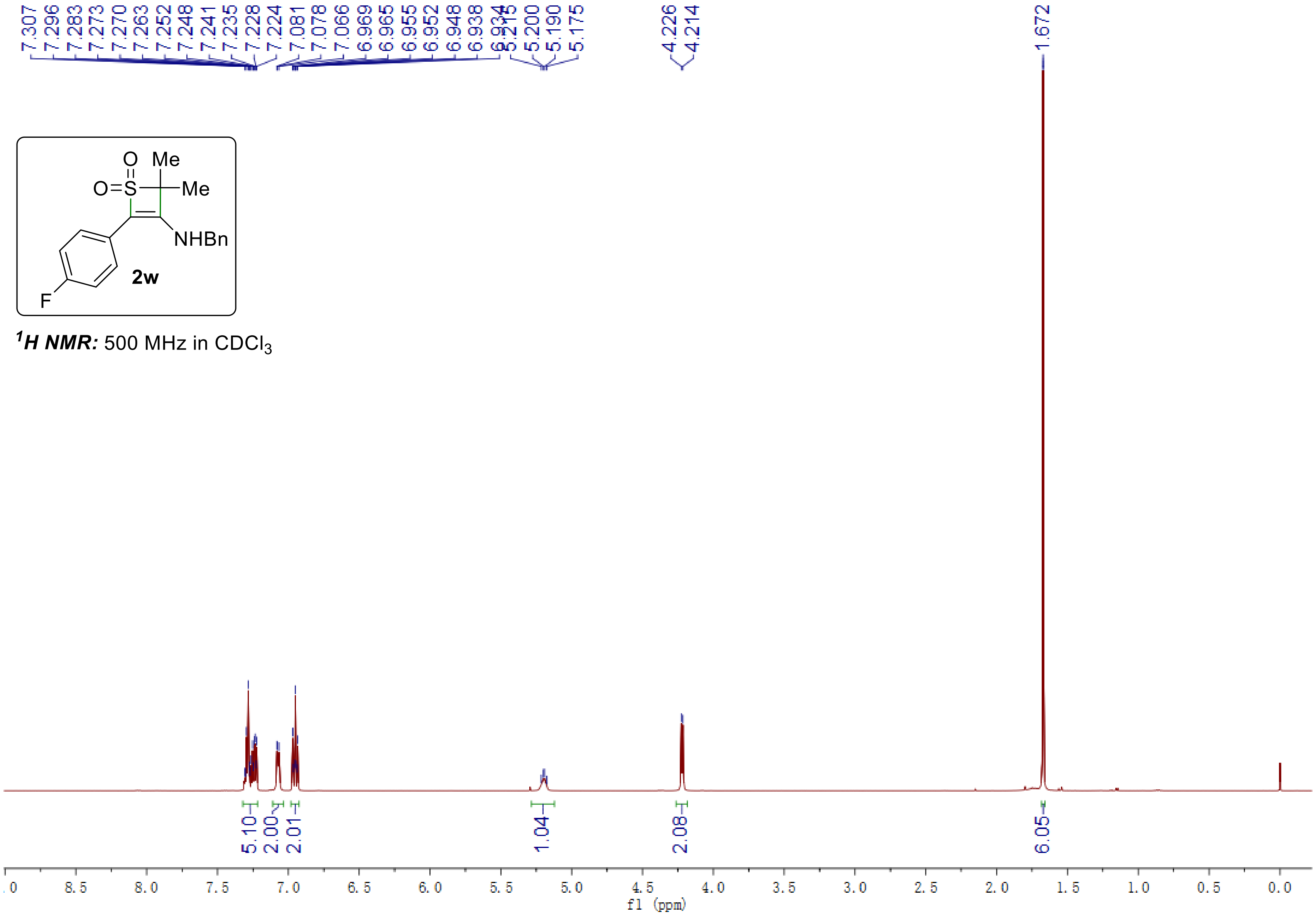

^{1}H NMR: 500 MHz in $CDCl_3$

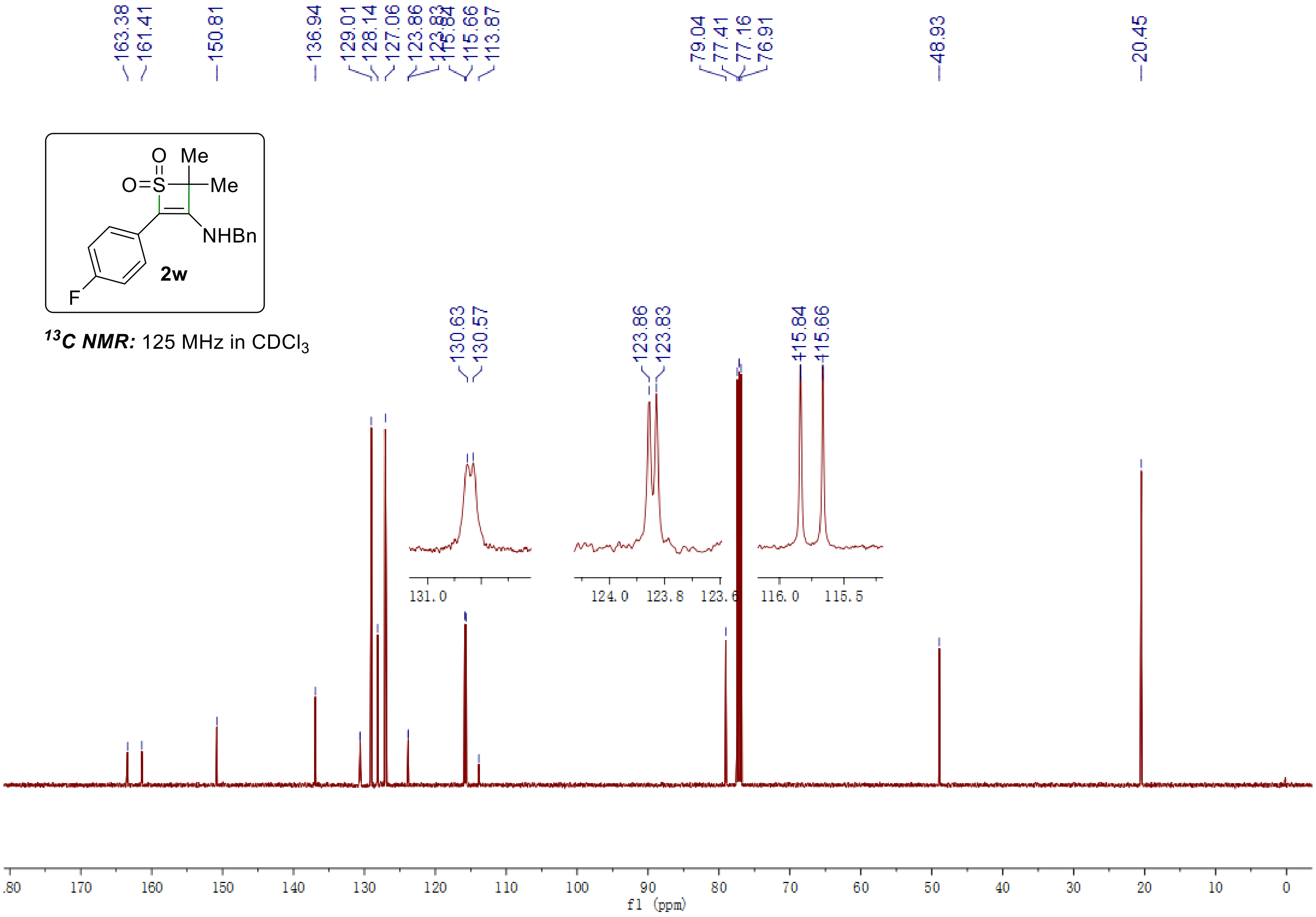

163.38
161.41
150.81
136.94
129.01
128.14
127.06
123.86
123.83
115.84
115.66
113.87
79.04
77.41
77.16
76.91
48.93
20.45
2w
NHBn
F
Me
13C NMR: 125 MHz in CDCl3
130.63
130.57
123.86
123.83
115.84
115.66
f1 (ppm)

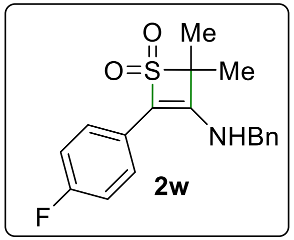

^{19}F NMR: 376 MHz in $CDCl_3$

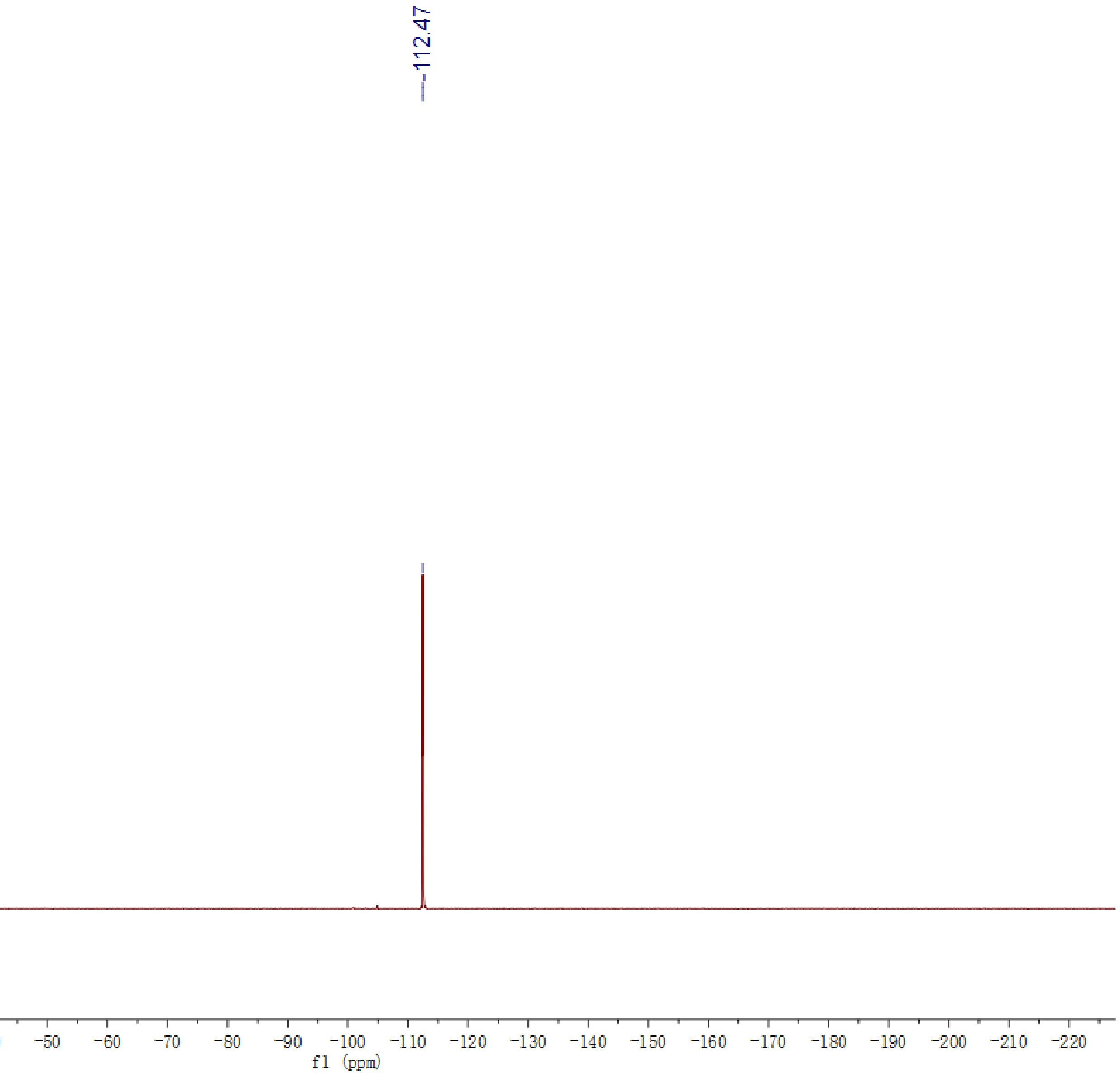

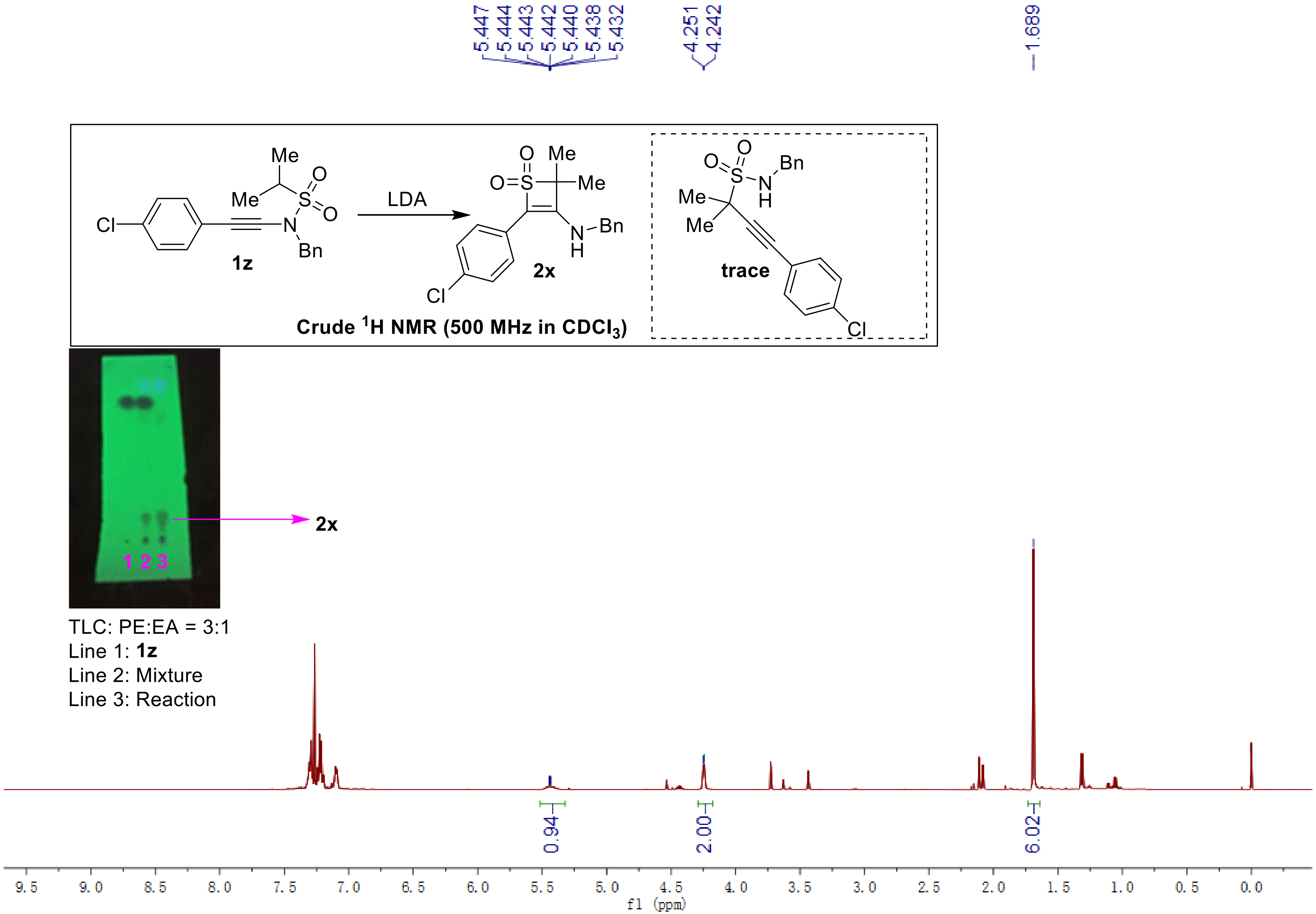
5.447
5.444
5.443
5.442
5.440
5.438
5.432
4.251
4.242
1.689
Cl
Me
Me
S
O
O
N
Bn
1z
LDA
O
Me
O=S
Me
N
Bn
H
Cl
2x
O
O
S
Bn
N
H
Me
Me
trace
Cl
Crude ¹H NMR (500 MHz in CDCl3)
1 2 3
2x
TLC: PE:EA = 3:1
Line 1: 1z
Line 2: Mixture
Line 3: Reaction
0.94
2.00
6.02
9.5 9.0 8.5 8.0 7.5 7.0 6.5 6.0 5.5 5.0 4.5 4.0 3.5 3.0 2.5 2.0 1.5 1.0 0.5 0.0
f1 (ppm)

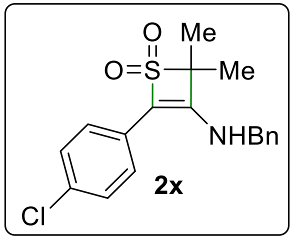

^{1}H NMR: 600 MHz in $CDCl_3$

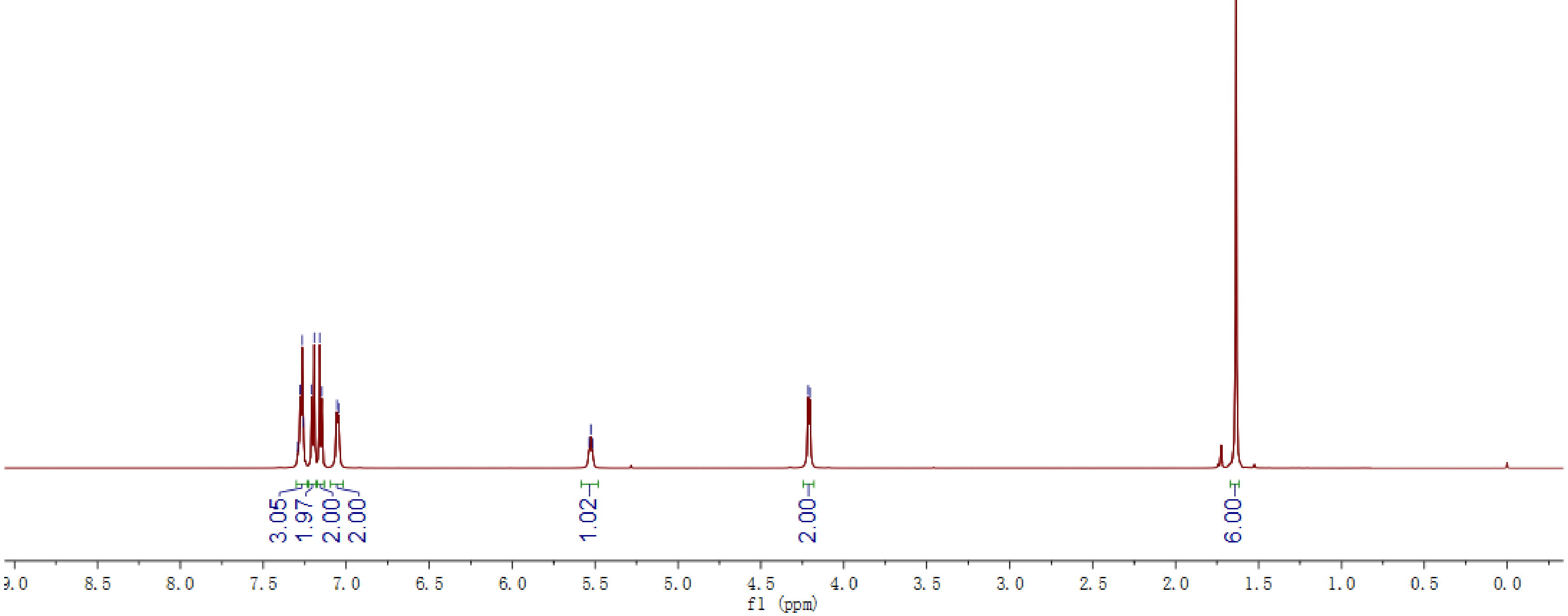

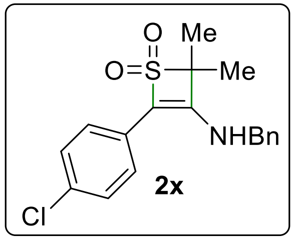

^{13}***C NMR:*** 150 MHz in $CDCl_3$

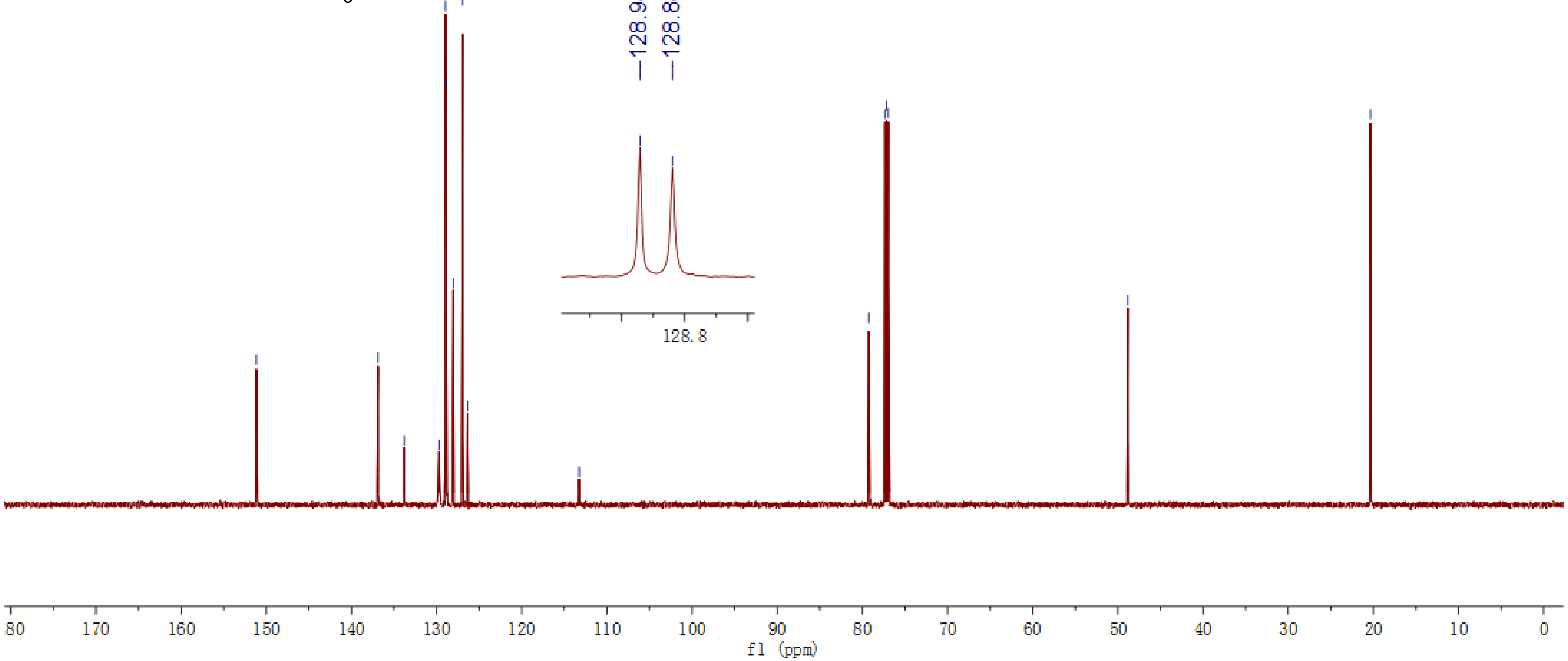

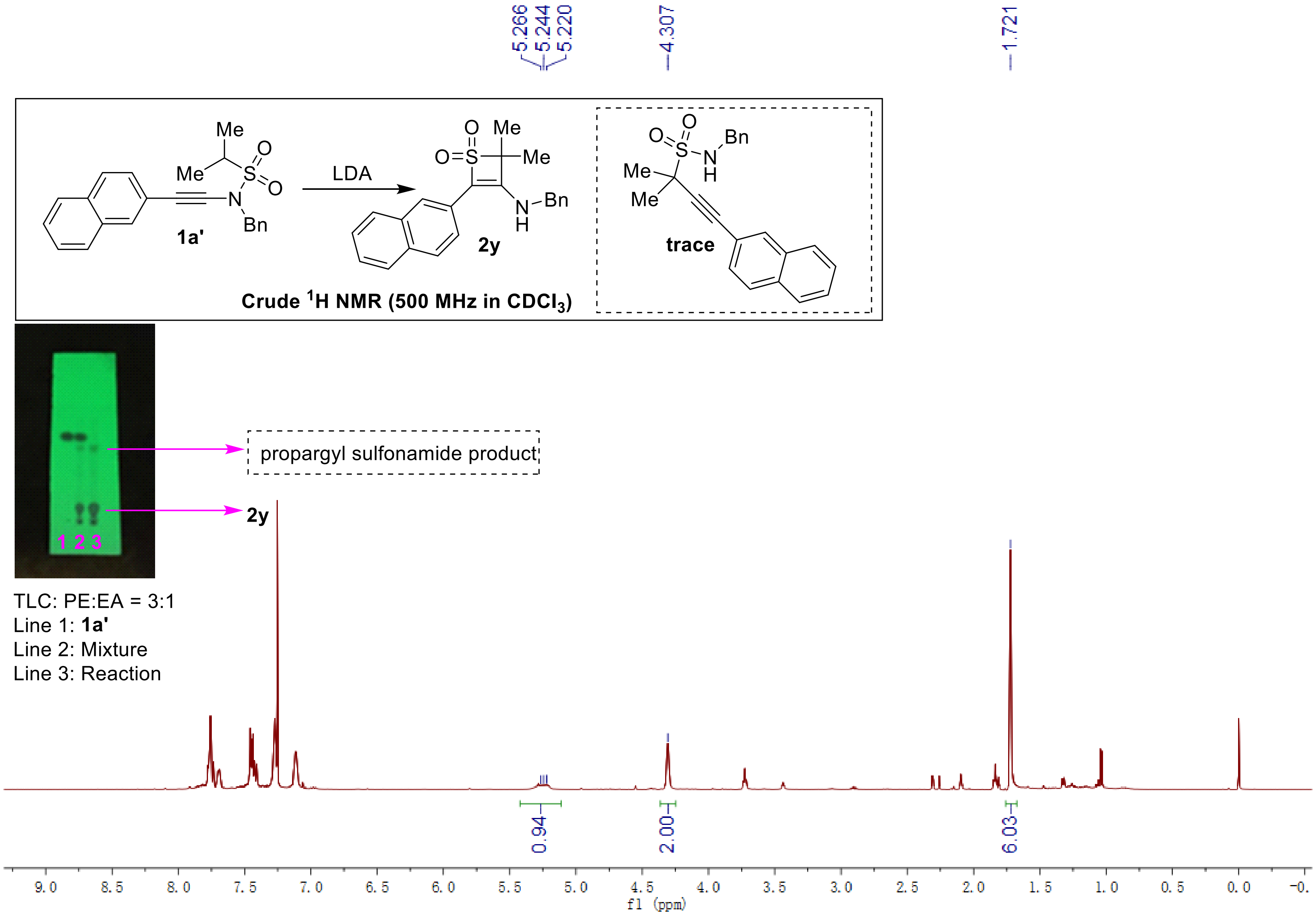

5.266
5.244
5.220
4.307
1.721
Me
Me
O
S
O
N
Bn
1a'
LDA
O
Me
O=S
Me
N
Bn
H
2y
O
O
S
N
Bn
H
Me
Me
trace
Crude ¹H NMR (500 MHz in CDCl3)
1 2 3
propargyl sulfonamide product
2y
TLC: PE:EA = 3:1
Line 1: 1a'
Line 2: Mixture
Line 3: Reaction
0.94
2.00
6.03
9.0 8.5 8.0 7.5 7.0 6.5 6.0 5.5 5.0 4.5 4.0 3.5 3.0 2.5 2.0 1.5 1.0 0.5 0.0 -0.
f1 (ppm)

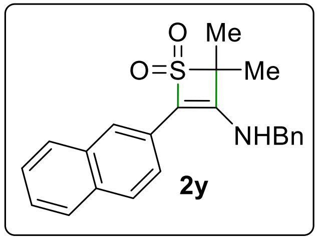

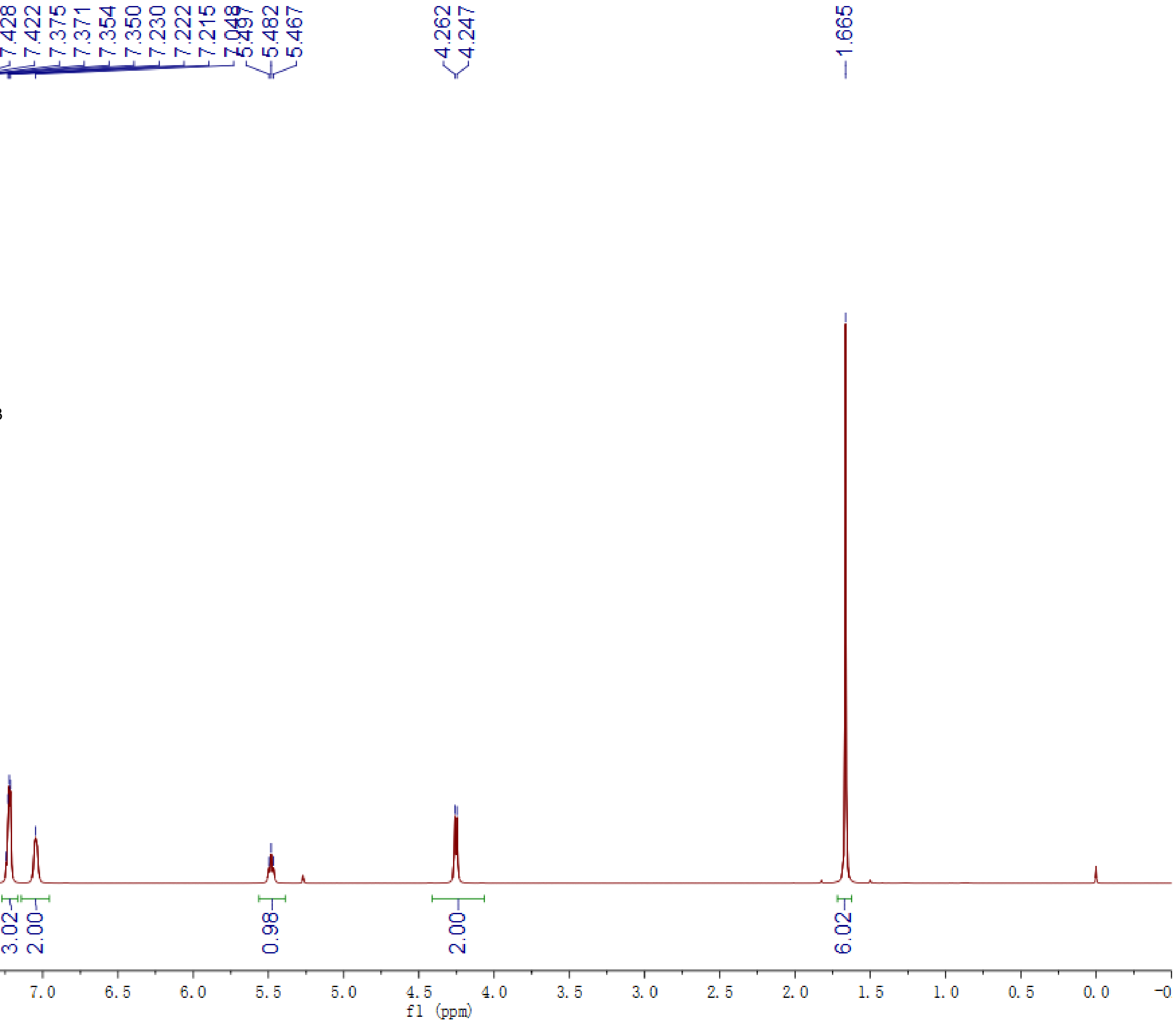

[1]***H NMR:*** 400 MHz in $CDCl_3$

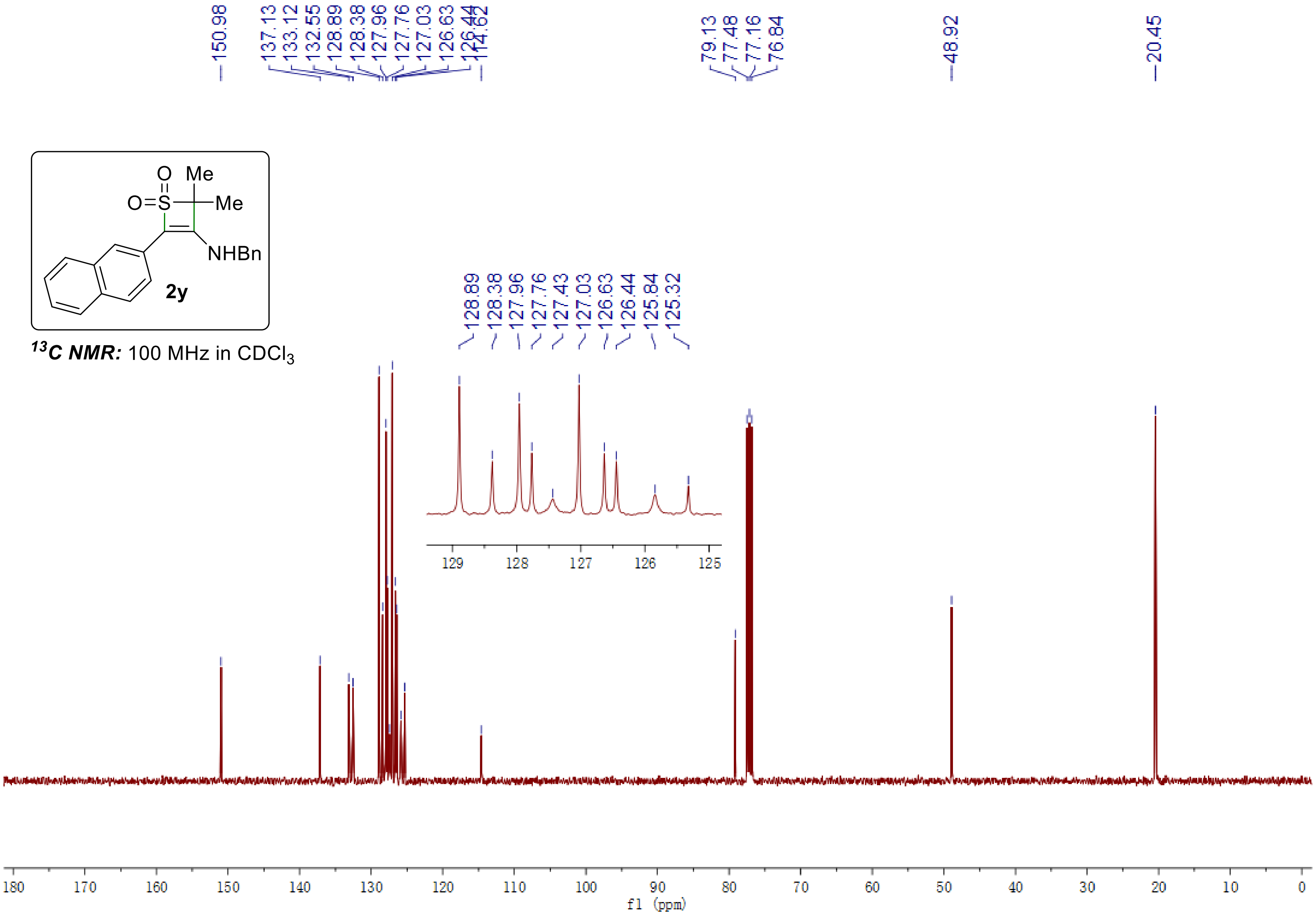
150.98
137.13
133.12
132.55
128.89
128.38
127.96
127.76
127.03
126.63
126.44
114.62
79.13
77.48
77.16
76.84
48.92
20.45
O
Me
O=S
Me
NHBn
2y
^{13}C NMR: 100 MHz in $CDCl_3$
128.89
128.38
127.96
127.76
127.43
127.03
126.63
126.44
125.84
125.32
129
128
127
126
125
180
170
160
150
140
130
120
110
100
90
80
70
60
50
40
30
20
10
0
f1 (ppm)

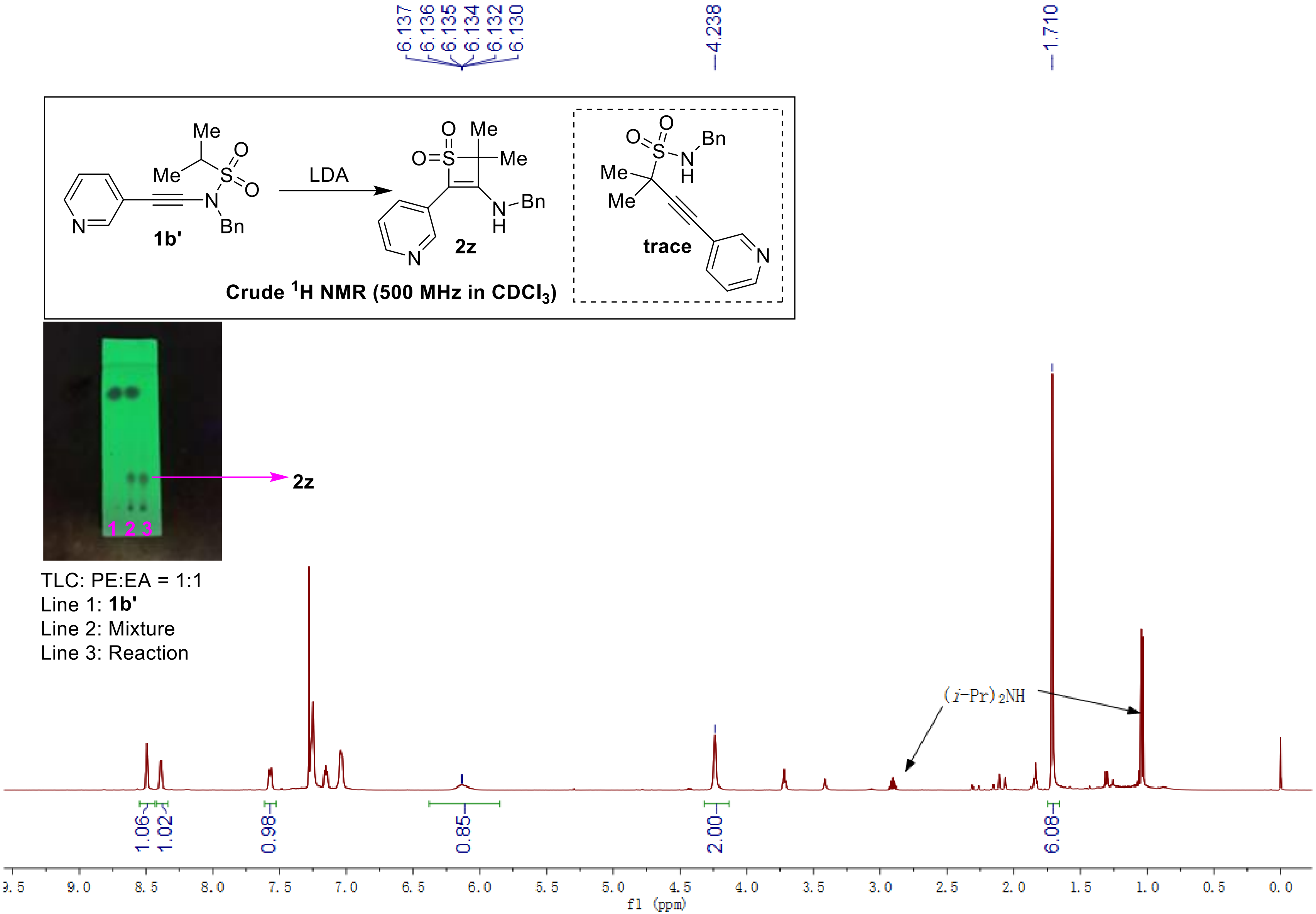
6.137
6.136
6.135
6.134
6.132
6.130
4.238
1.710
Me
Me
O
S
O
N
Bn
N
1b'
LDA
O
Me
O=S
Me
N-Bn
H
N
2z
O
O
S
Bn
N
H
Me
Me
trace
N
Crude 1H NMR (500 MHz in CDCl3)
2z
1 2 3
TLC: PE:EA = 1:1
Line 1: 1b'
Line 2: Mixture
Line 3: Reaction
(i-Pr)2NH
1.06
1.02
0.98
0.85
2.00
6.08
9.5
9.0
8.5
8.0
7.5
7.0
6.5
6.0
5.5
5.0
4.5
4.0
3.5
3.0
2.5
2.0
1.5
1.0
0.5
0.0
f1 (ppm)

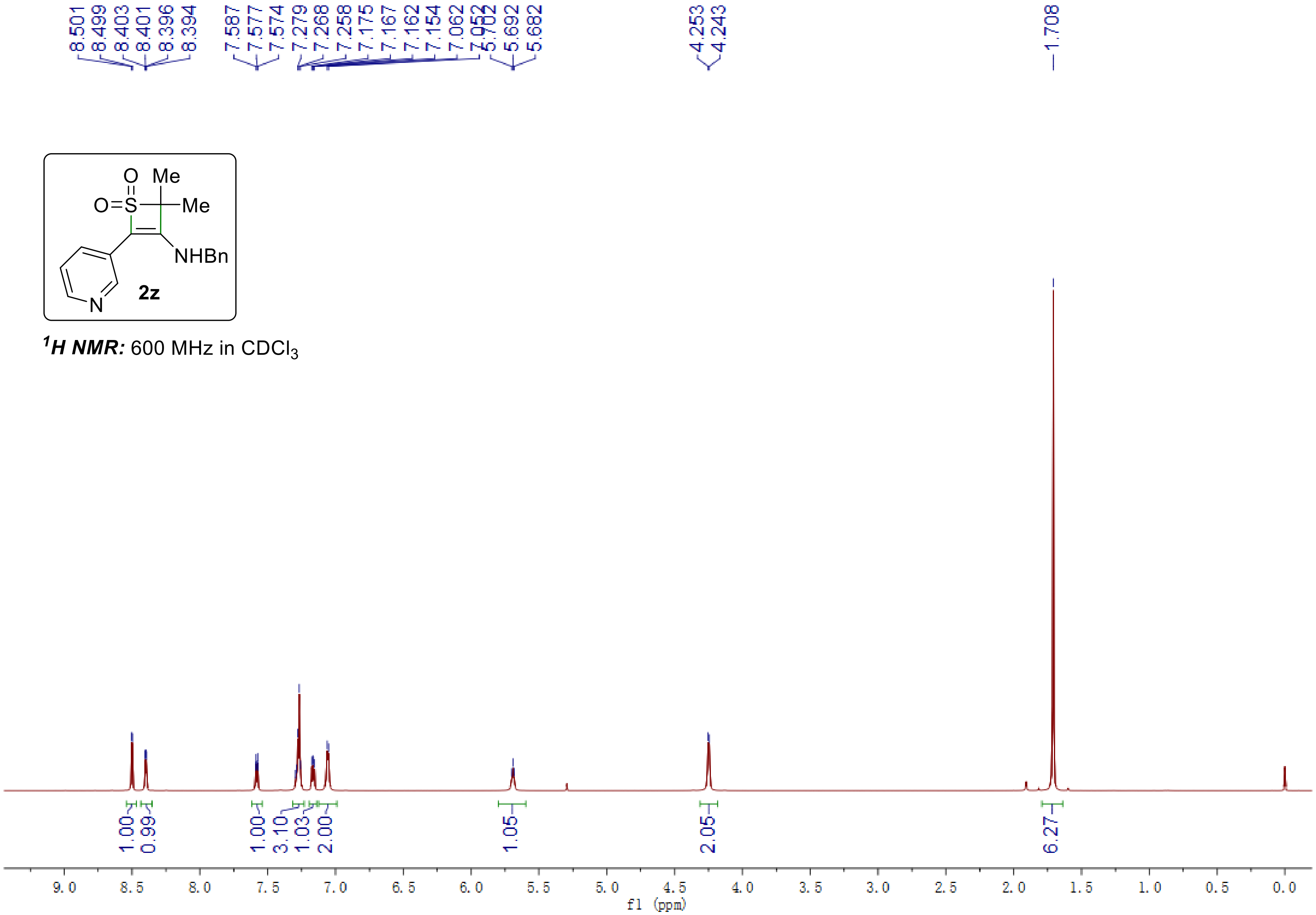

^{1}H NMR: 600 MHz in $CDCl_3$

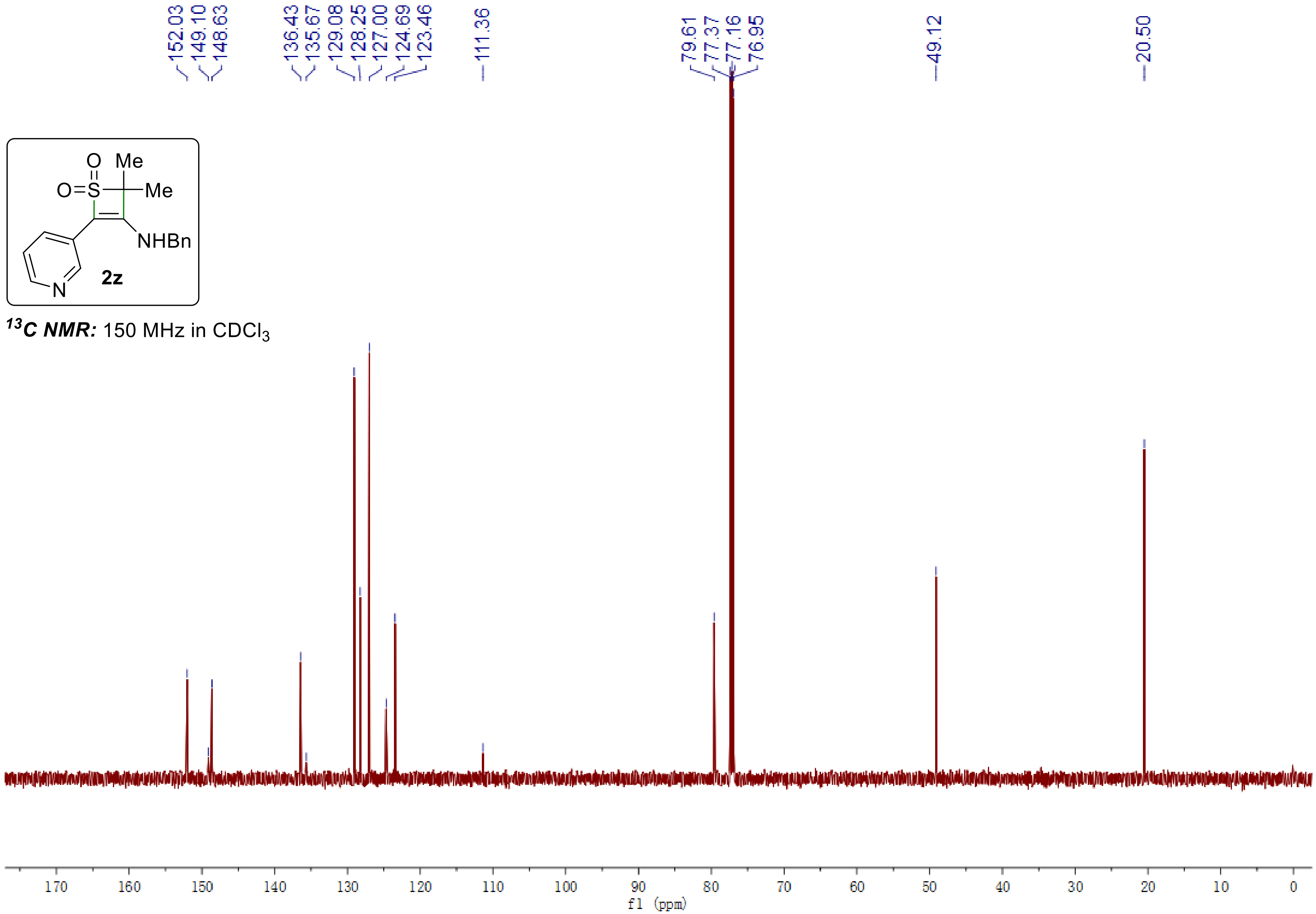
152.03
149.10
148.63
136.43
135.67
129.08
128.25
127.00
124.69
123.46
111.36
79.61
77.37
77.16
76.95
49.12
20.50
O
Me
O=S
Me
NHBn
N
2z
13C NMR: 150 MHz in CDCl3
170
160
150
140
130
120
110
100
90
80
70
60
50
40
30
20
10
0
f1 (ppm)

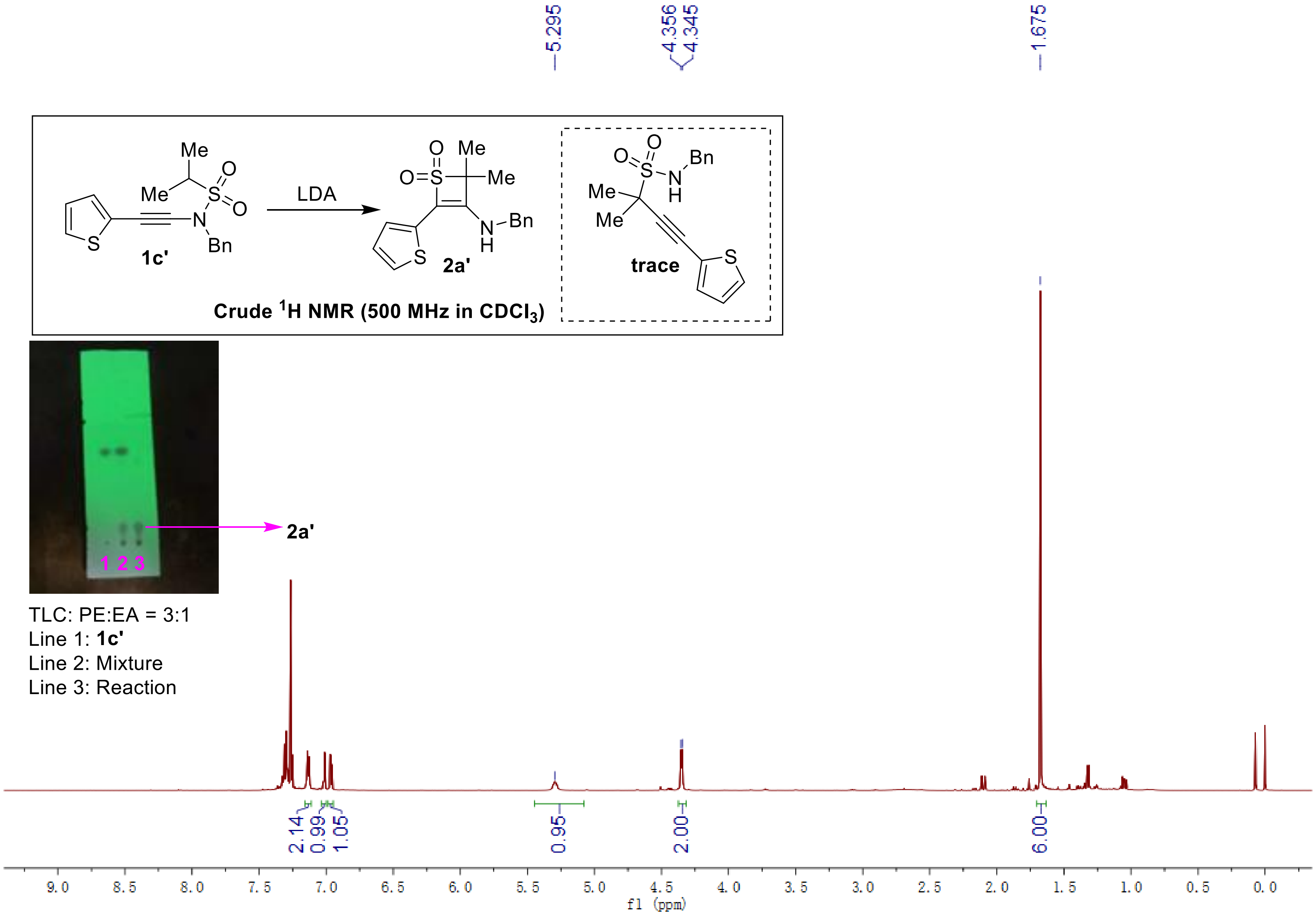
5.295
4.356
4.345
1.675
Me
Me
O
S
O
N
Bn
S
1c'
LDA
O
O=S
Me
Me
N-Bn
H
S
2a'
O
O
S
N
Bn
H
Me
Me
trace
S
Crude 1H NMR (500 MHz in CDCl3)
1 2 3
2a'
TLC: PE:EA = 3:1
Line 1: 1c'
Line 2: Mixture
Line 3: Reaction
2.14
0.99
1.05
0.95
2.00
6.00
9.0
8.5
8.0
7.5
7.0
6.5
6.0
5.5
5.0
4.5
4.0
3.5
3.0
2.5
2.0
1.5
1.0
0.5
0.0
f1 (ppm)

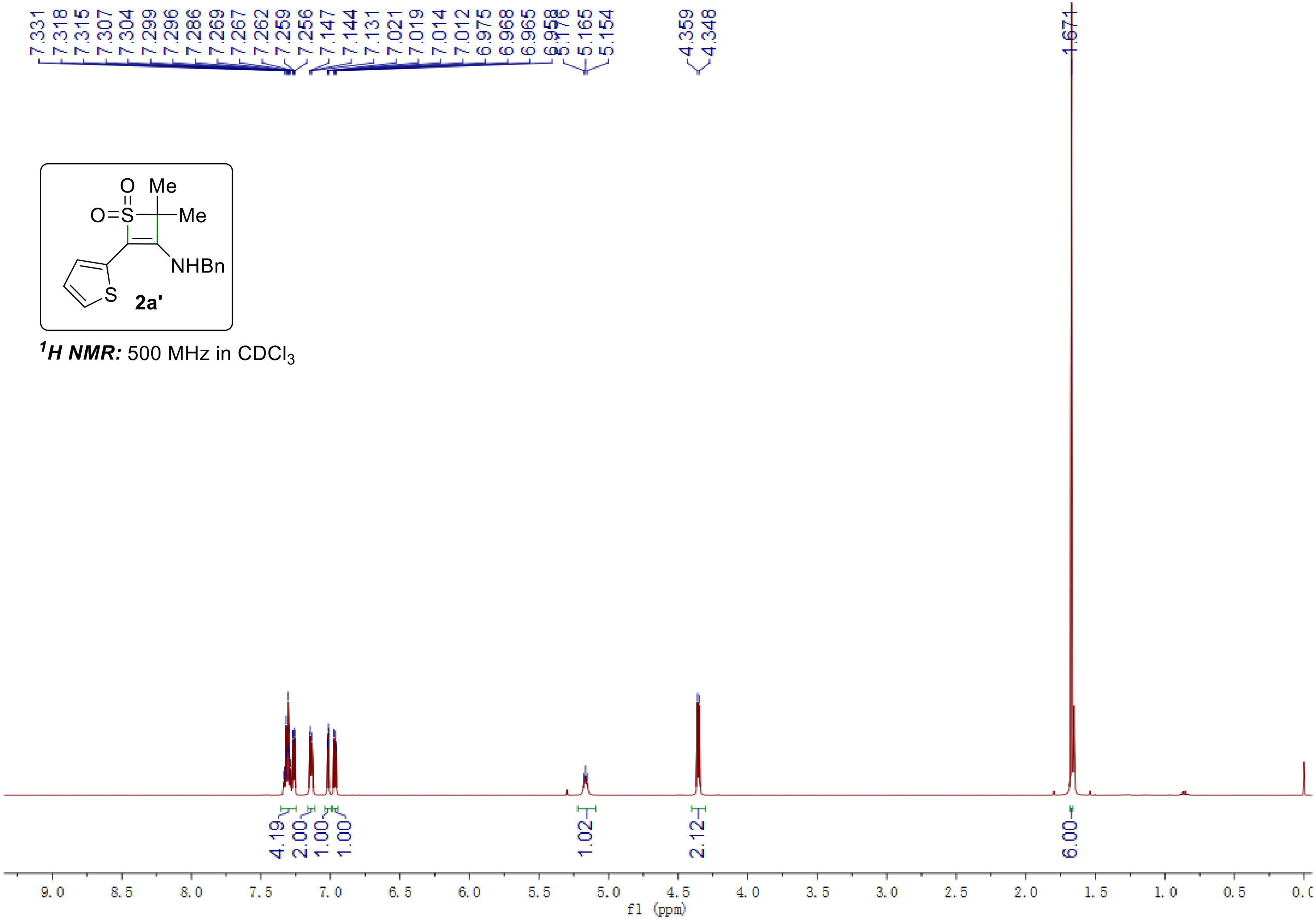

7.331
7.318
7.315
7.307
7.304
7.299
7.296
7.286
7.269
7.267
7.262
7.259
7.256
7.147
7.144
7.131
7.021
7.019
7.014
7.012
6.975
6.968
6.965
6.958
5.176
5.165
5.154
4.359
4.348
1.671
O
Me
O=S
Me
NHBn
S
2a'
1H NMR: 500 MHz in CDCl3
4.19
2.00
1.00
1.00
1.02
2.12
6.00
9.0
8.5
8.0
7.5
7.0
6.5
6.0
5.5
5.0
4.5
4.0
3.5
3.0
2.5
2.0
1.5
1.0
0.5
f1 (ppm)

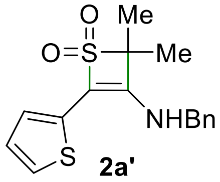

2a'

13C NMR: 125 MHz in $CDCl_3$

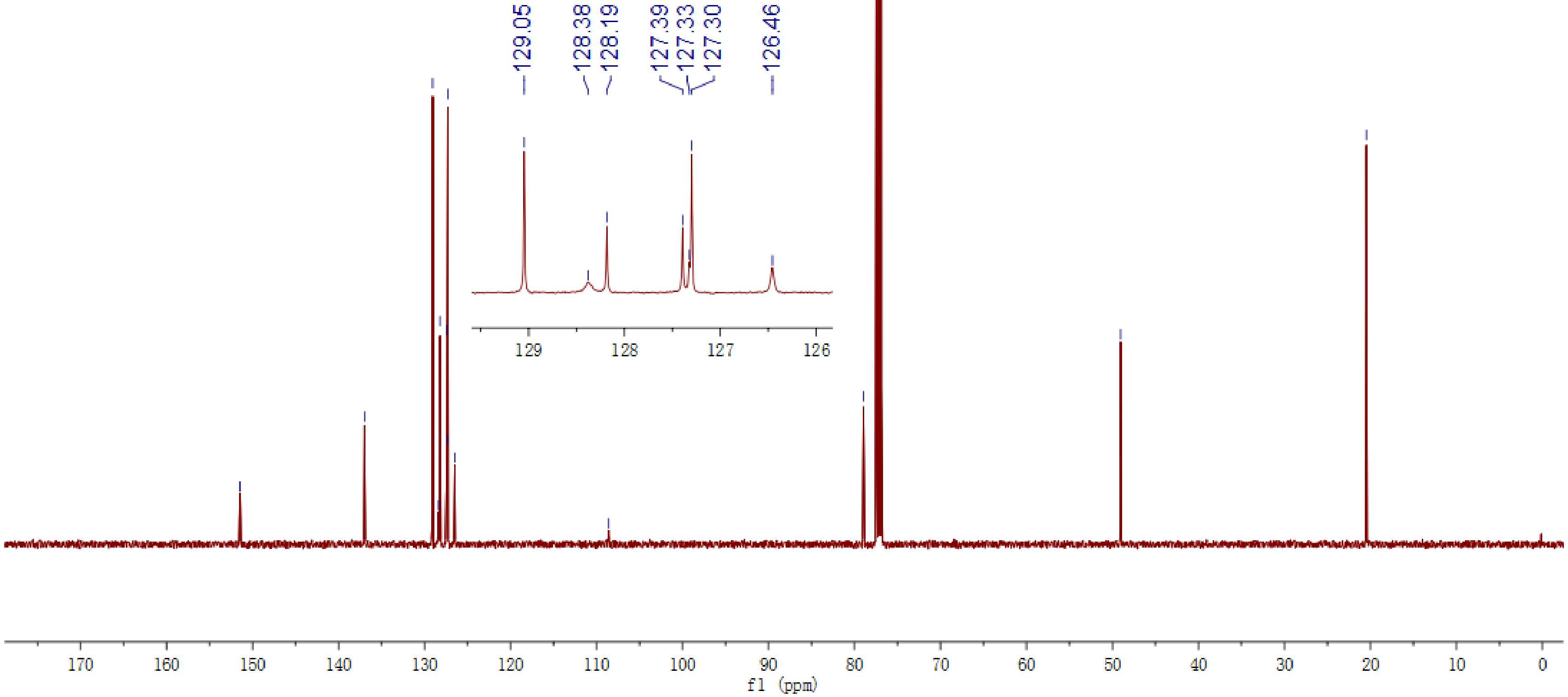

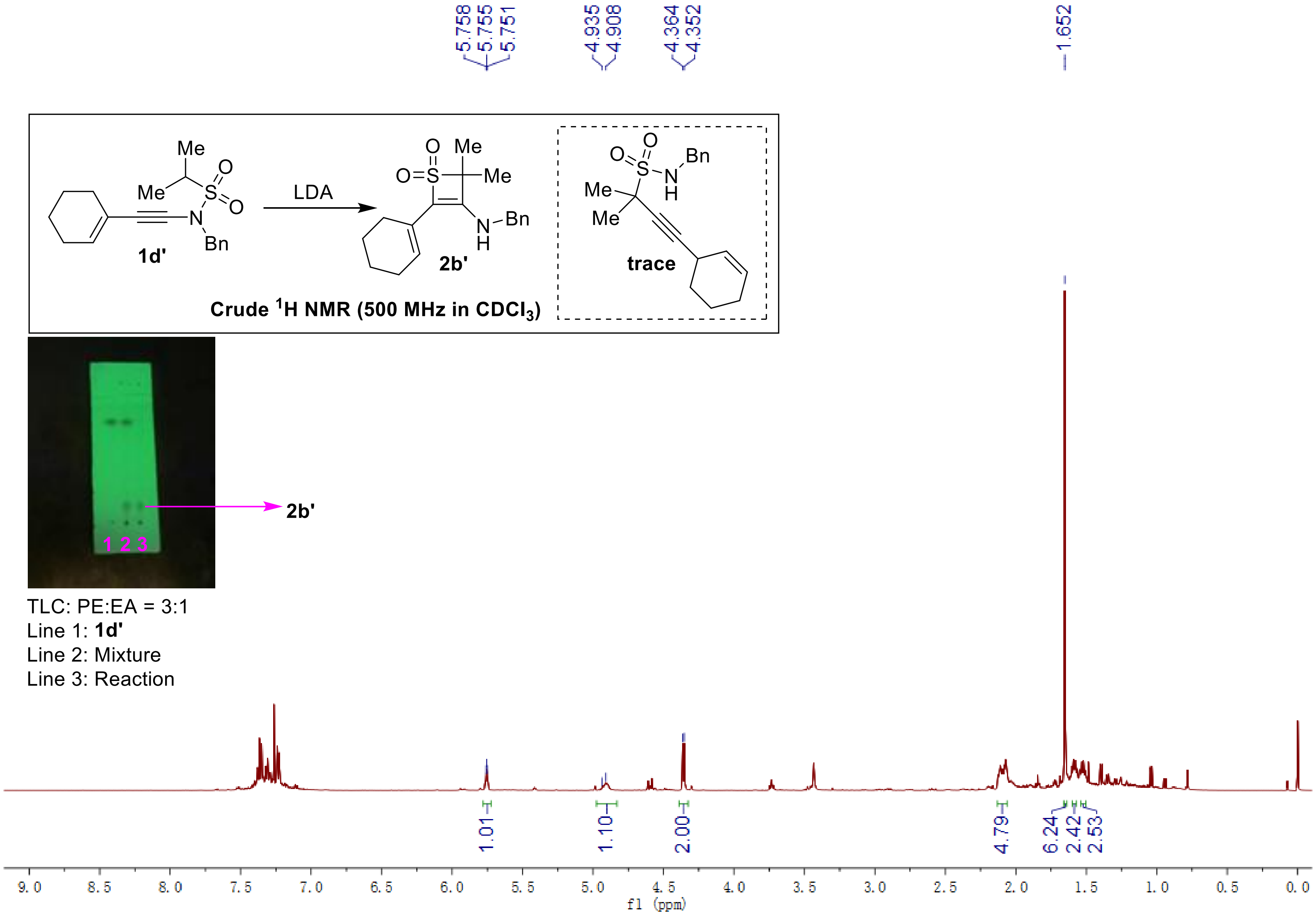

5.758
5.755
5.751
4.935
4.908
4.364
4.352
1.652
Me
Me
O
S
O
N
Bn
1d'
LDA
O
O=S
Me
Me
N-Bn
H
2b'
O
O
S
N
Bn
H
Me
Me
trace
Crude 1H NMR (500 MHz in CDCl3)
1 2 3
2b'
TLC: PE:EA = 3:1
Line 1: 1d'
Line 2: Mixture
Line 3: Reaction
1.01
1.10
2.00
4.79
6.24
2.42
2.53
9.0
8.5
8.0
7.5
7.0
6.5
6.0
5.5
5.0
4.5
4.0
3.5
3.0
2.5
2.0
1.5
1.0
0.5
0.0
f1 (ppm)

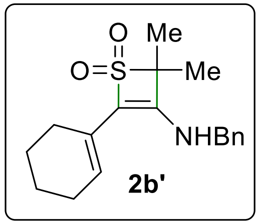

^{1}H NMR: 400 MHz in $CDCl_3$

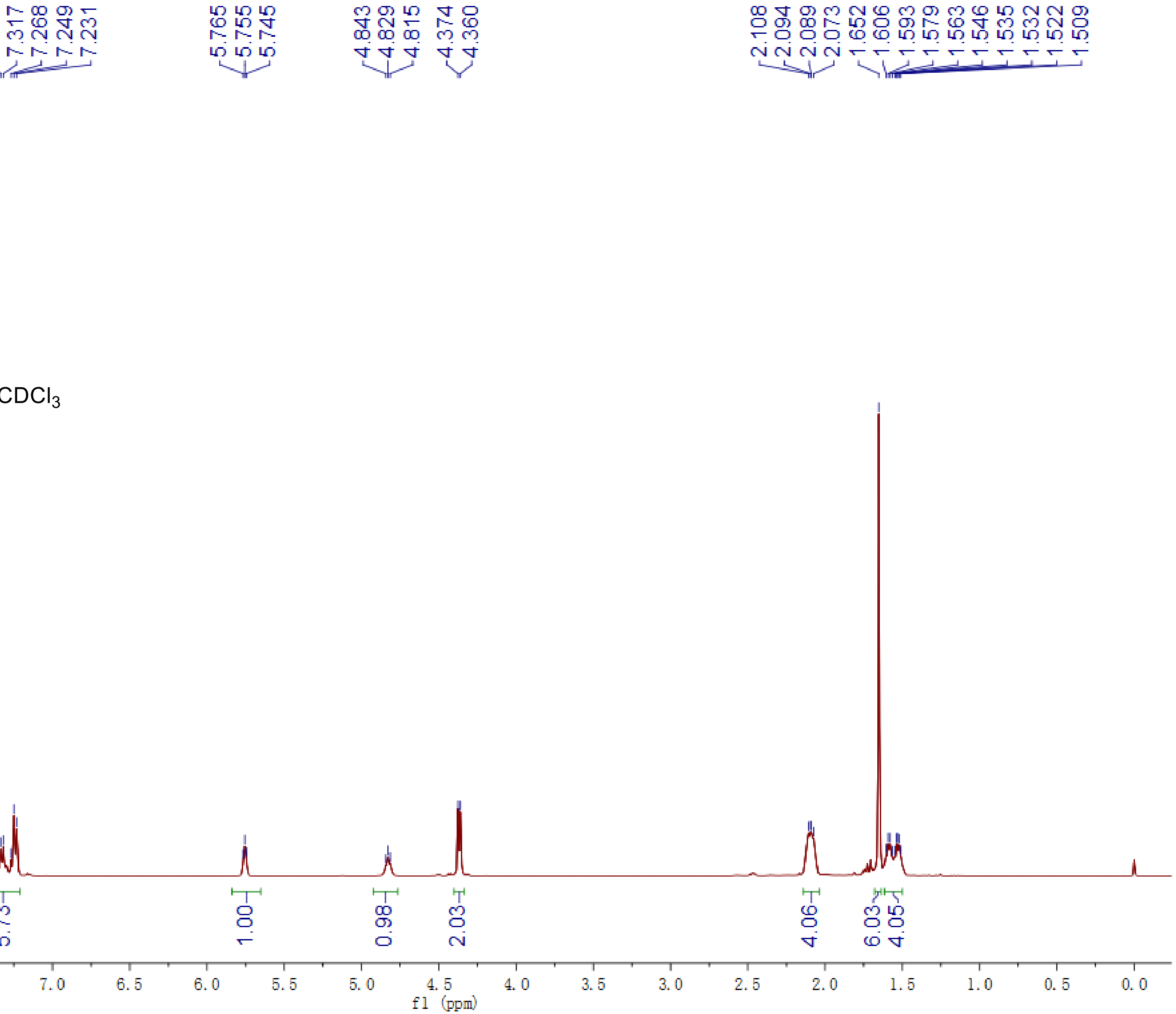

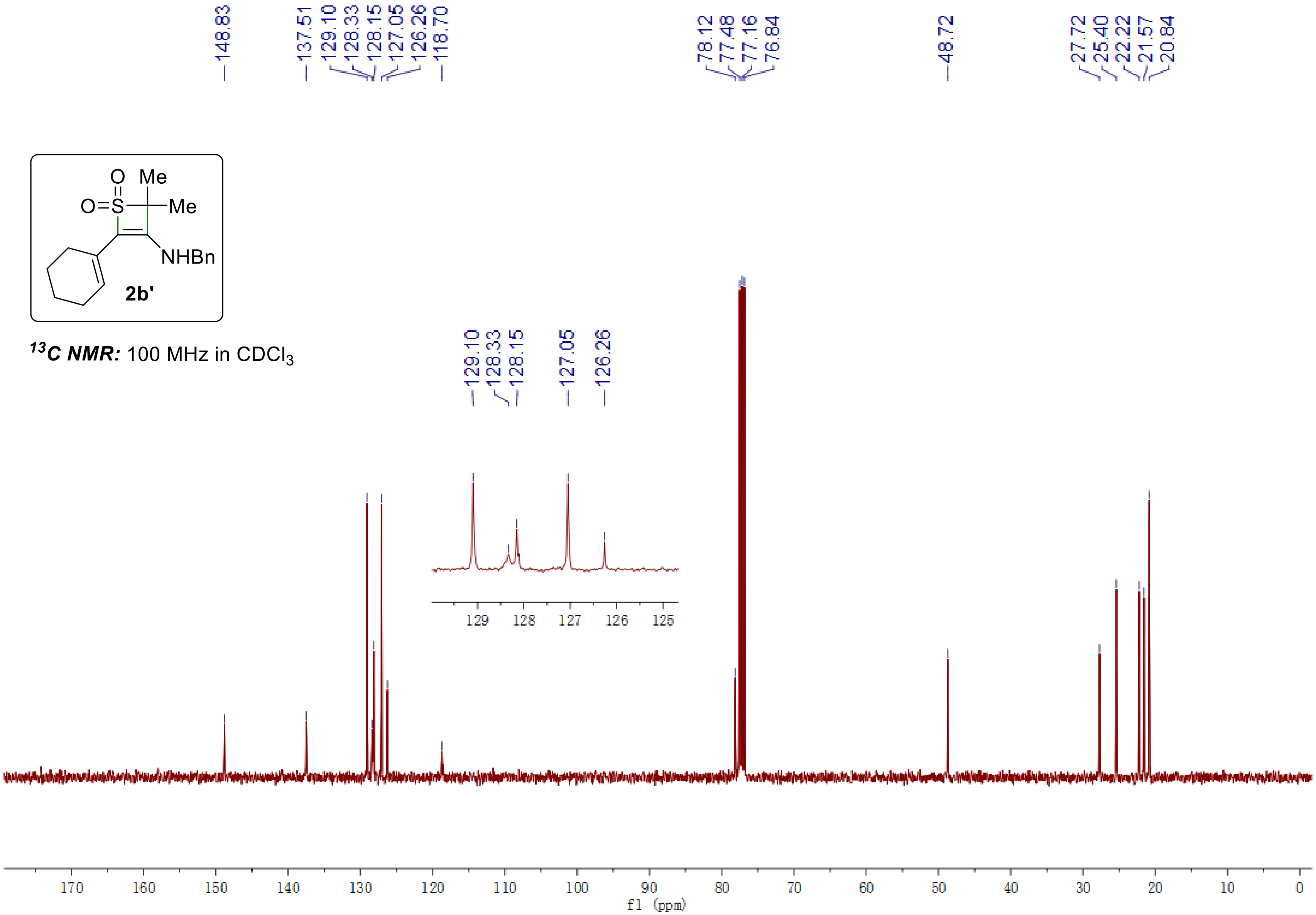
148.83
137.51
129.10
128.33
128.15
127.05
126.26
118.70
78.12
77.48
77.16
76.84
48.72
27.72
25.40
22.22
21.57
20.84
O
Me
O=S
Me
NHBn
2b'
^{13}C NMR: 100 MHz in $CDCl_3$
f1 (ppm)

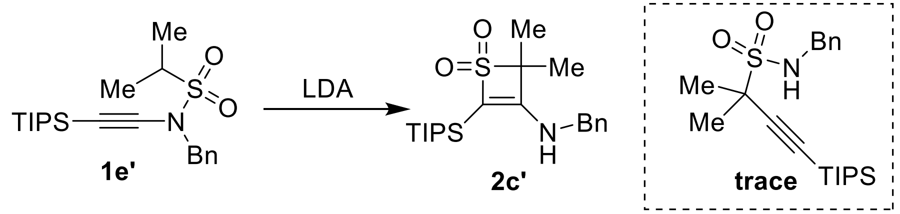

Crude ^{1}H NMR (500 MHz in $CDCl_3$)

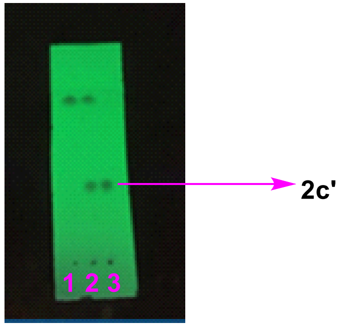

TLC: PE:EA = 3:1
Line 1: **1e'**
Line 2: Mixture
Line 3: Reaction

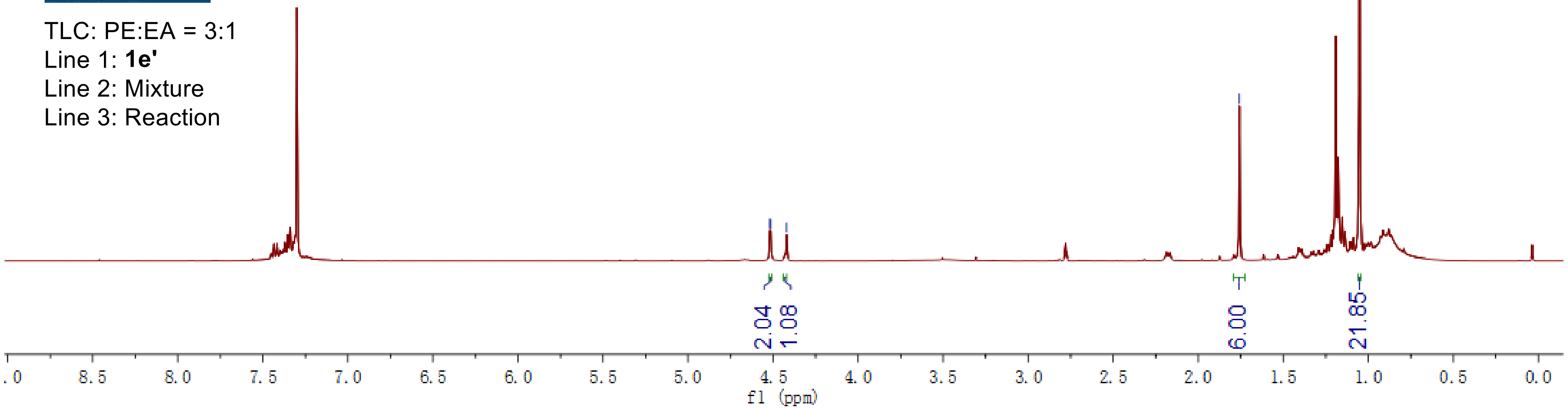

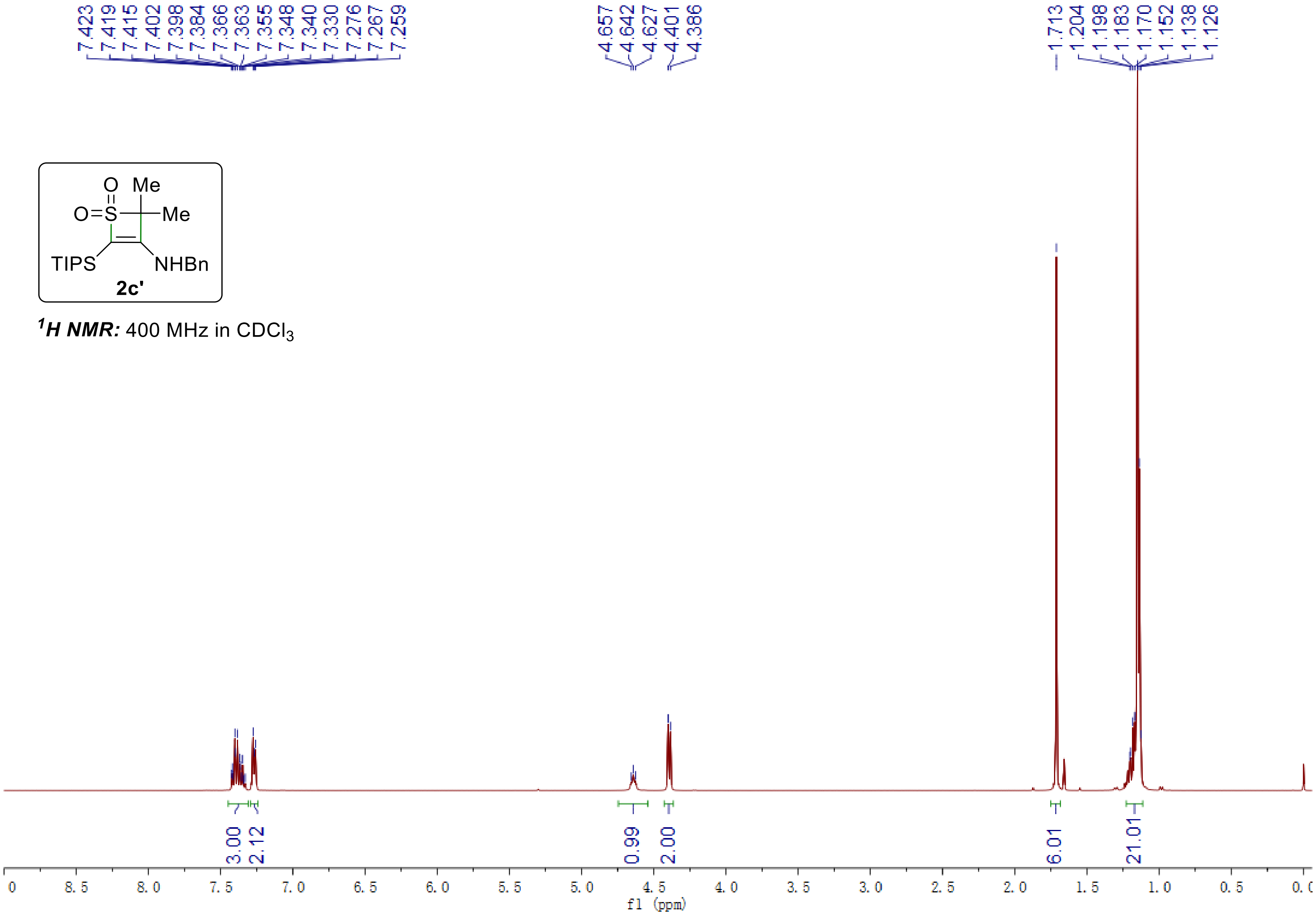

^{1}***H NMR:*** 400 MHz in $CDCl_3$

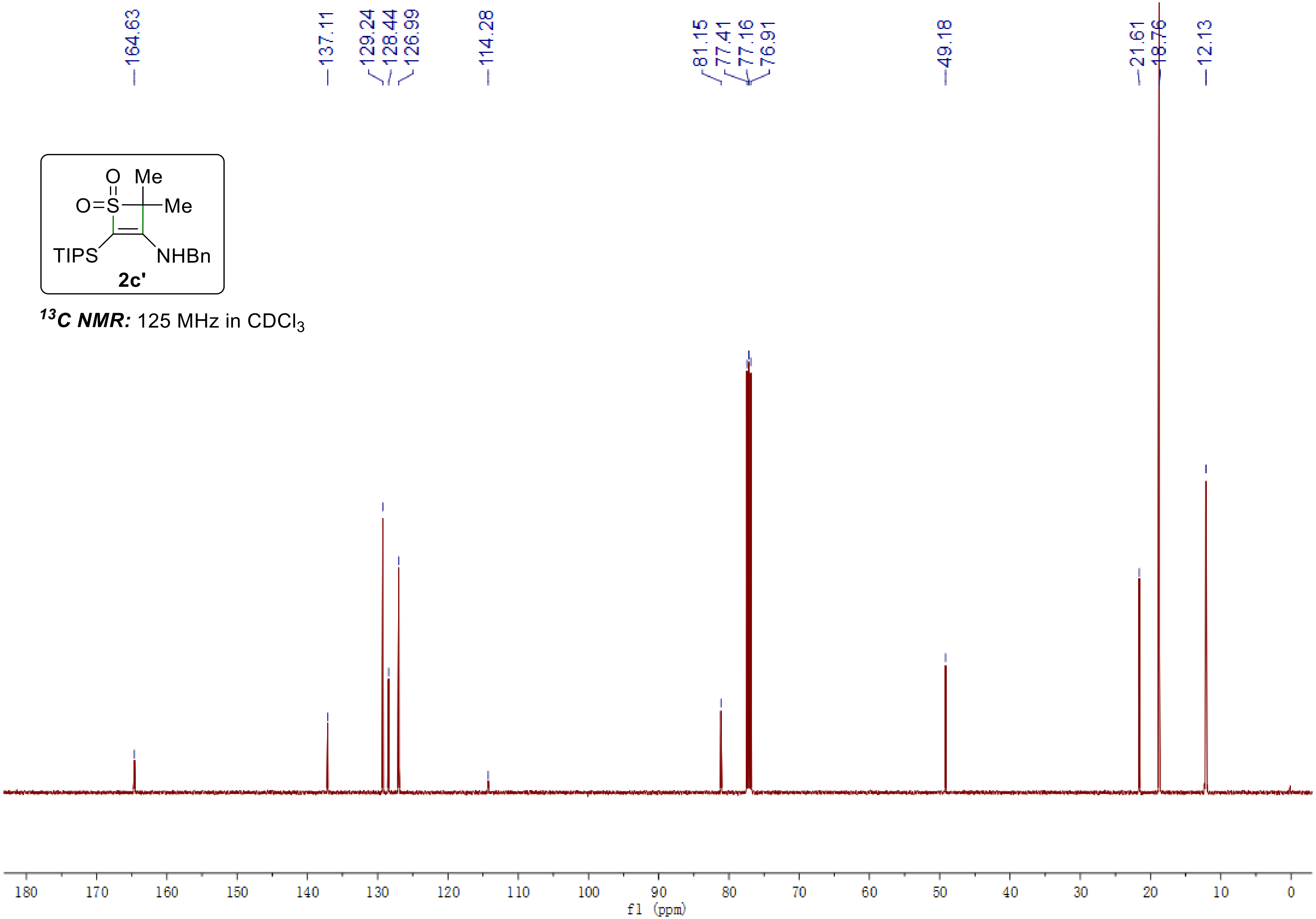

13C NMR: 125 MHz in $CDCl_3$

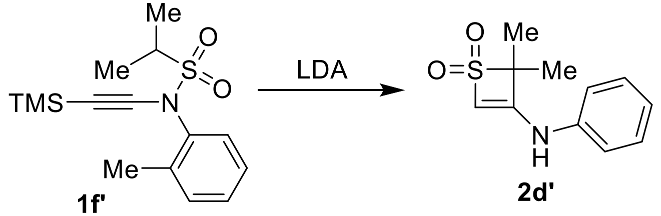

Crude ^{1}H NMR (500 MHz in $CDCl_3$)

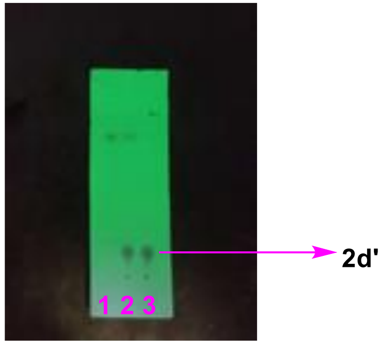

TLC: PE:EA = 3:1
Line 1: **1f'**
Line 2: Mixture
Line 3: Reaction

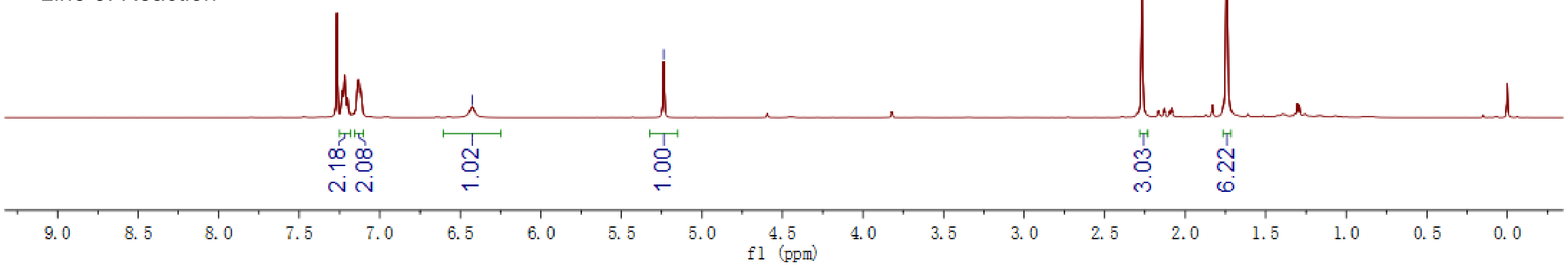

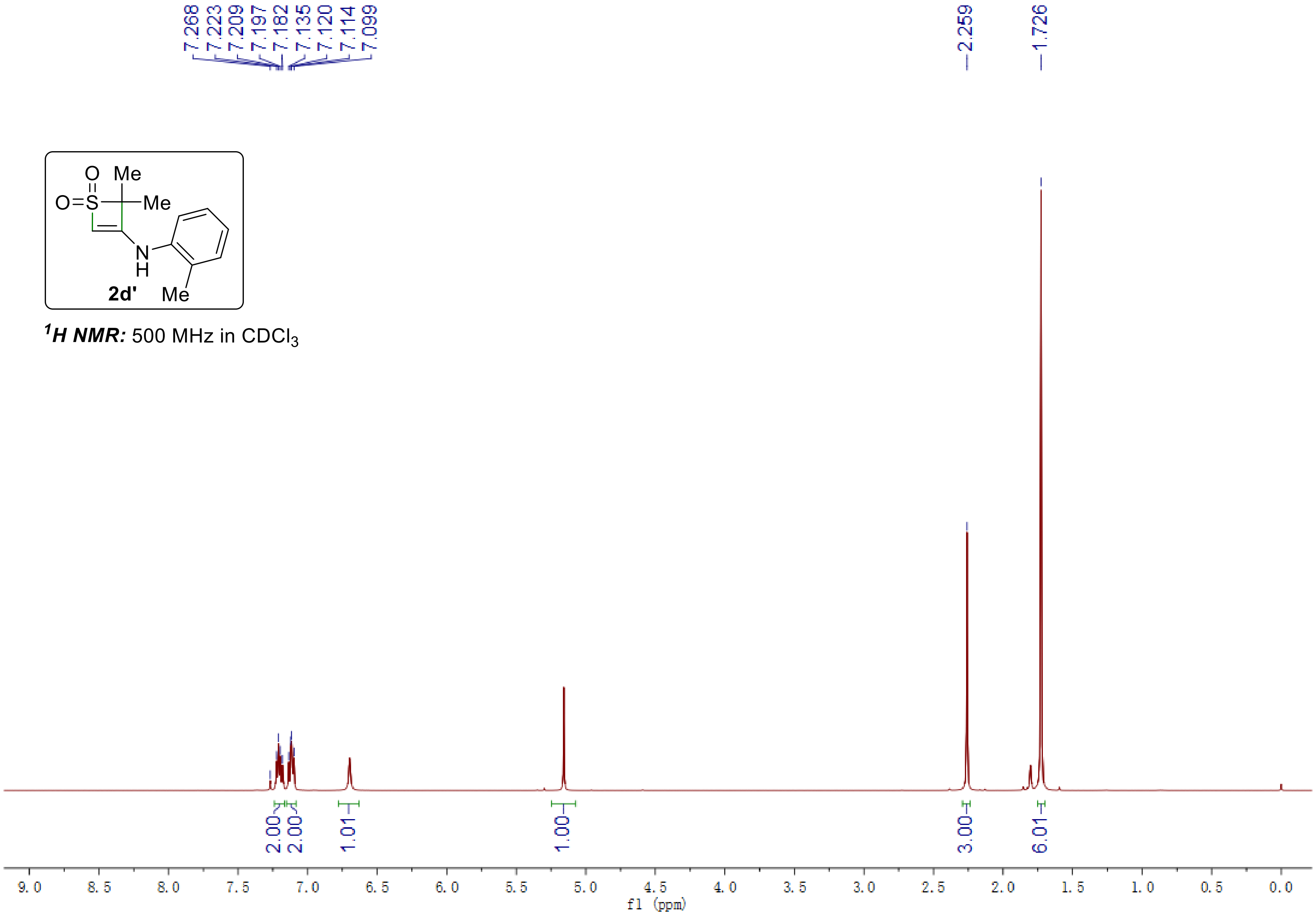
7.268
7.223
7.209
7.197
7.182
7.135
7.120
7.114
7.099
2.259
1.726
O
Me
O=S
Me
N
H
2d'
Me
1H NMR: 500 MHz in CDCl3
2.00
2.00
1.01
1.00
3.00
6.01
9.0
8.5
8.0
7.5
7.0
6.5
6.0
5.5
5.0
4.5
4.0
3.5
3.0
2.5
2.0
1.5
1.0
0.5
0.0
f1 (ppm)

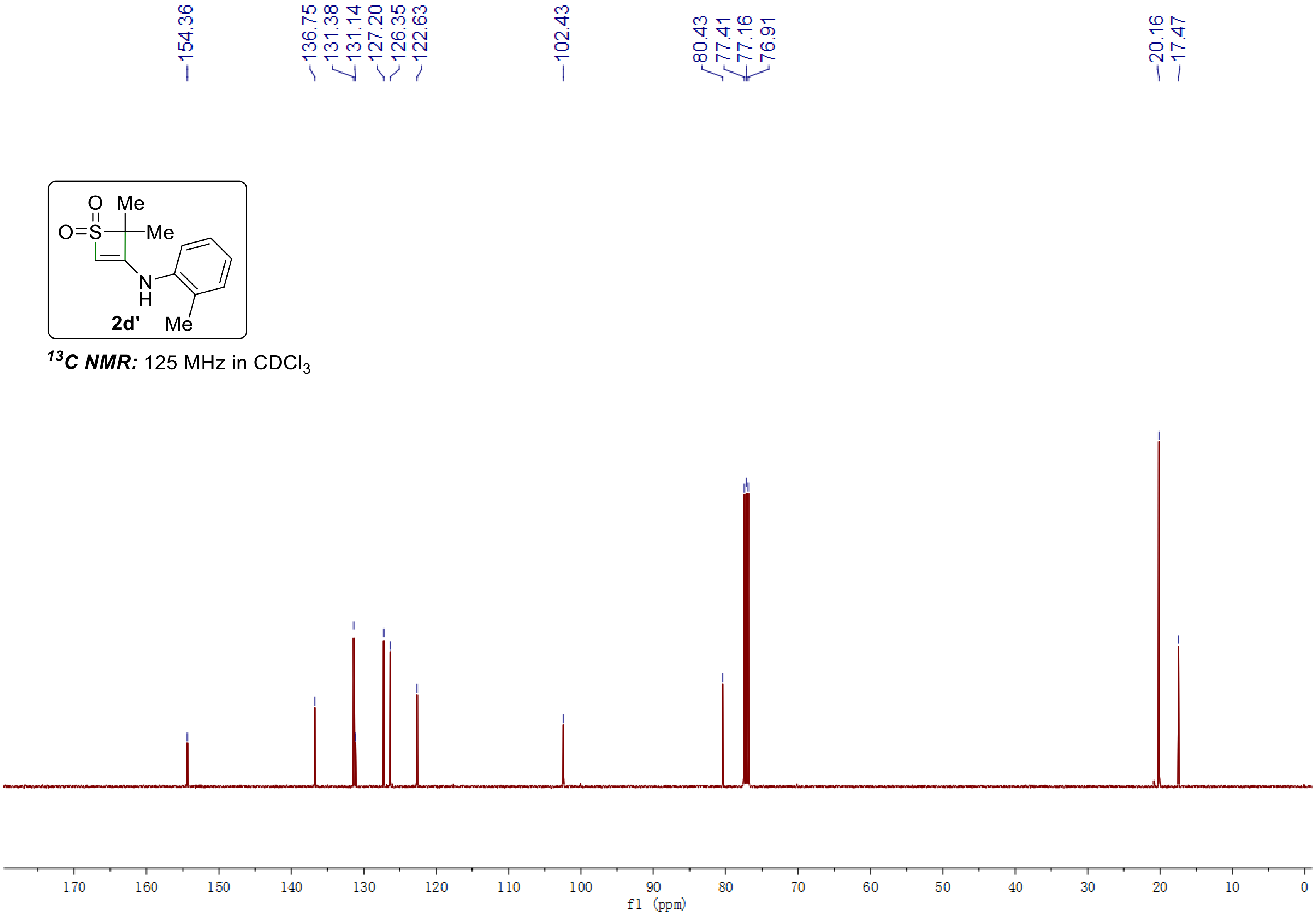

^{13}C NMR: 125 MHz in $CDCl_3$

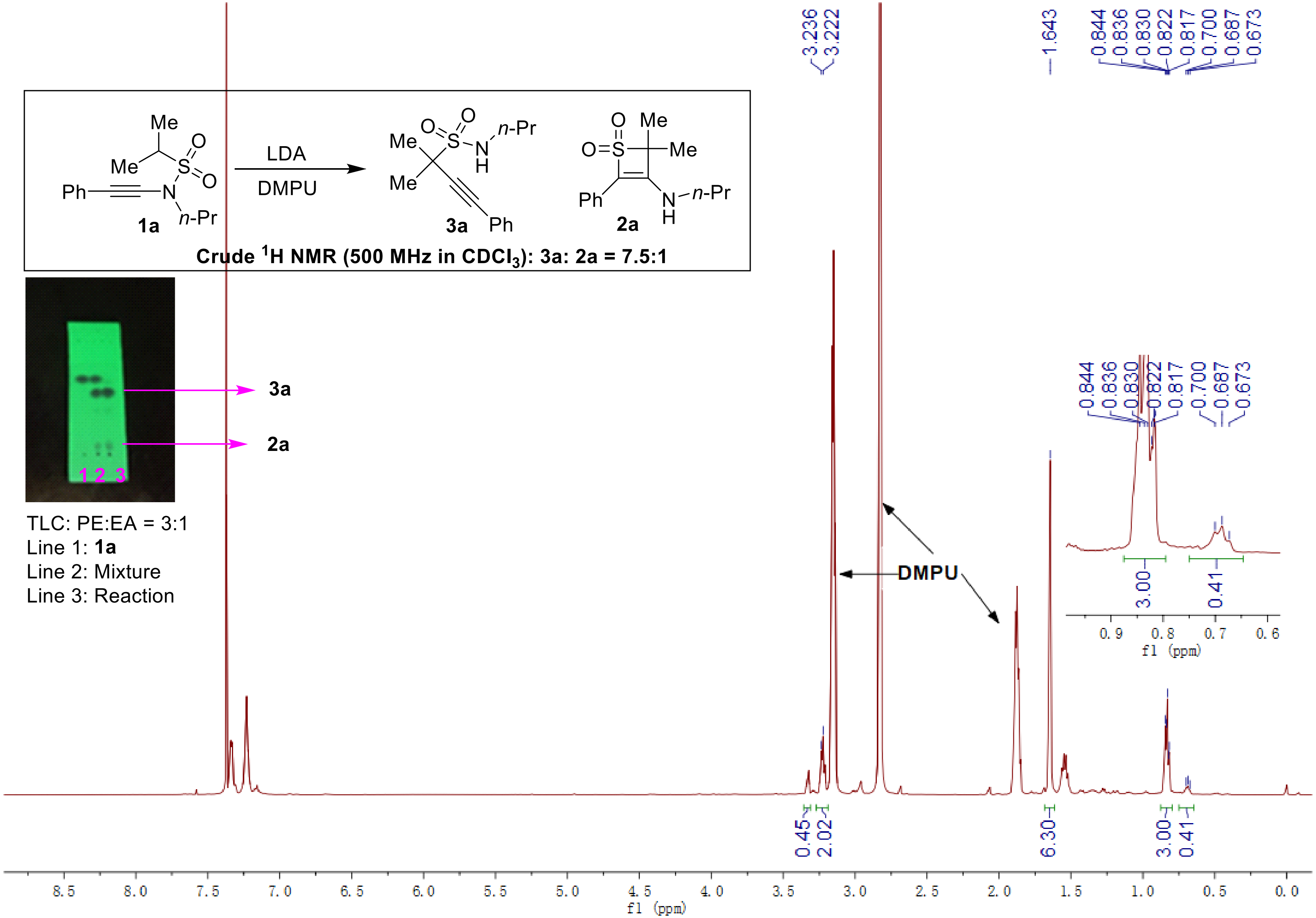
Ph
Me
Me
1a
n-Pr
LDA
DMPU
3a
2a
Crude ¹H NMR (500 MHz in CDCl₃): 3a: 2a = 7.5:1
3a
2a
1 2 3
TLC: PE:EA = 3:1
Line 1: 1a
Line 2: Mixture
Line 3: Reaction
DMPU
3.236
3.222
1.643
0.844
0.836
0.830
0.822
0.817
0.700
0.687
0.673
0.45
2.02
6.30
3.00
0.41
f1 (ppm)

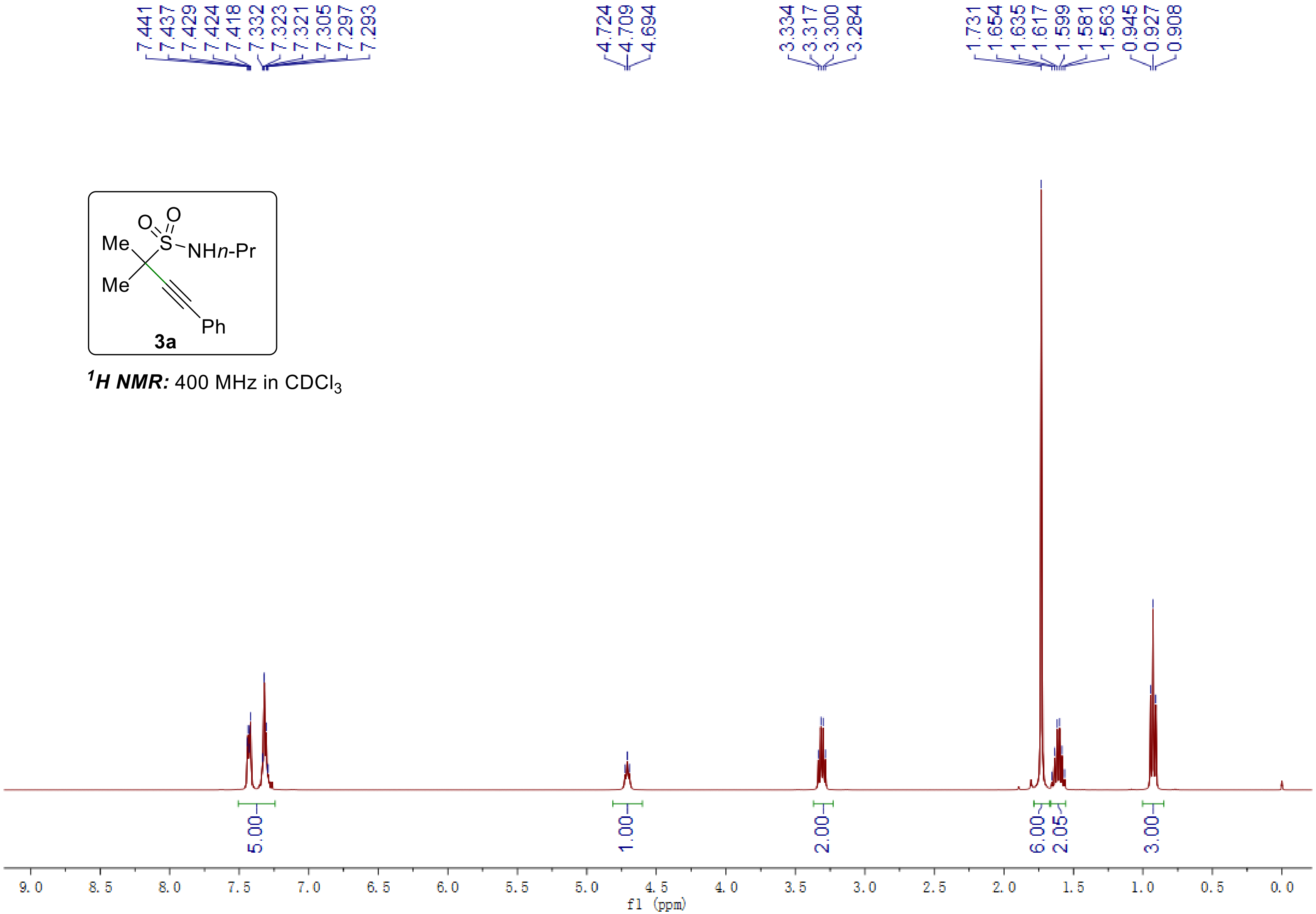
7.441
7.437
7.429
7.424
7.418
7.332
7.323
7.321
7.305
7.297
7.293
4.724
4.709
4.694
3.334
3.317
3.300
3.284
1.731
1.654
1.635
1.617
1.599
1.581
1.563
0.945
0.927
0.908
Me
Me
O
O
S
NHn-Pr
Ph
3a
1H NMR: 400 MHz in CDCl3
5.00
1.00
2.00
6.00
2.05
3.00
9.0
8.5
8.0
7.5
7.0
6.5
6.0
5.5
5.0
4.5
4.0
3.5
3.0
2.5
2.0
1.5
1.0
0.5
0.0
f1 (ppm)

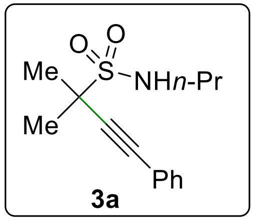

***13*C NMR:** 100 MHz in $CDCl_3$

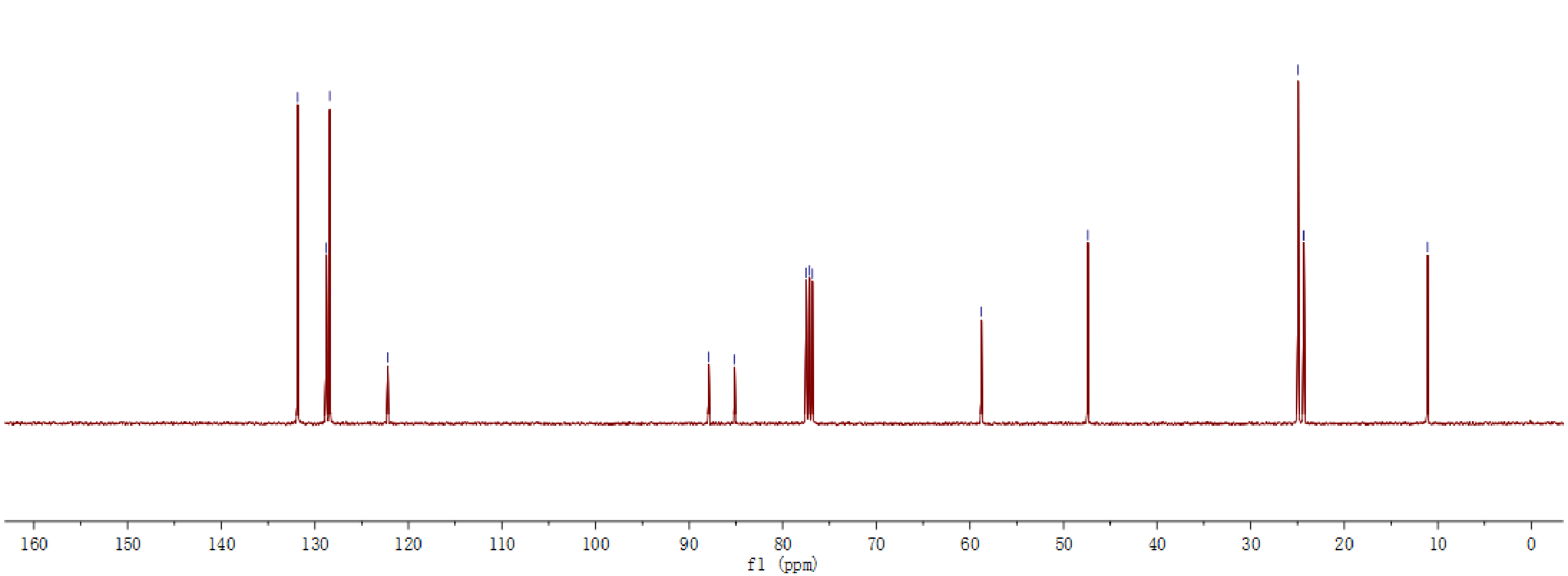

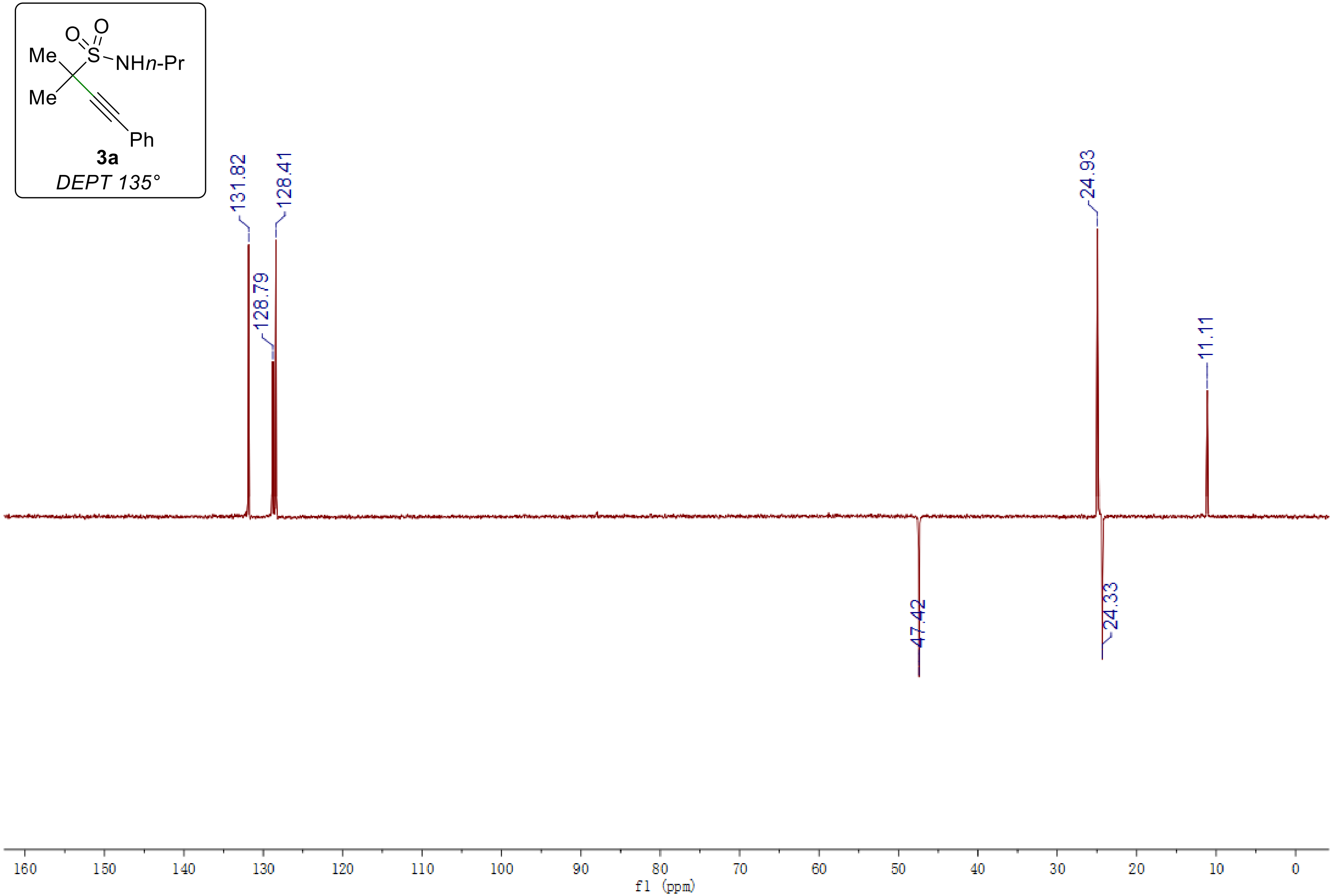

O
O
Me
S
NH*n*-Pr
Me
Ph
3a
DEPT 135°
131.82
128.79
128.41
24.93
11.11
47.42
24.33
160
150
140
130
120
110
100
90
80
70
60
50
40
30
20
10
0
f1 (ppm)

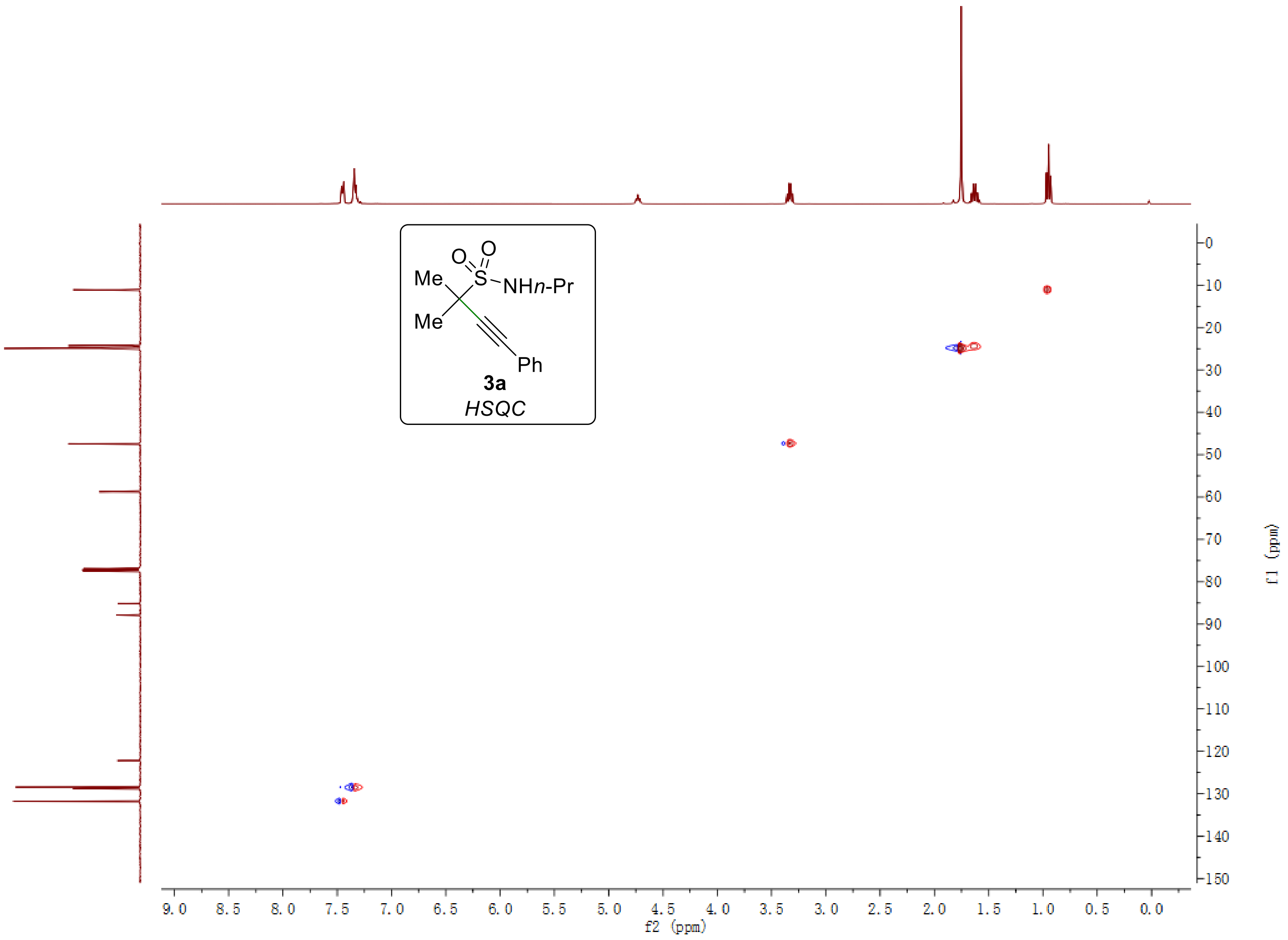

O
O
Me
S
NHn-Pr
Me
Ph
3a
HSQC
f2 (ppm)
f1 (ppm)

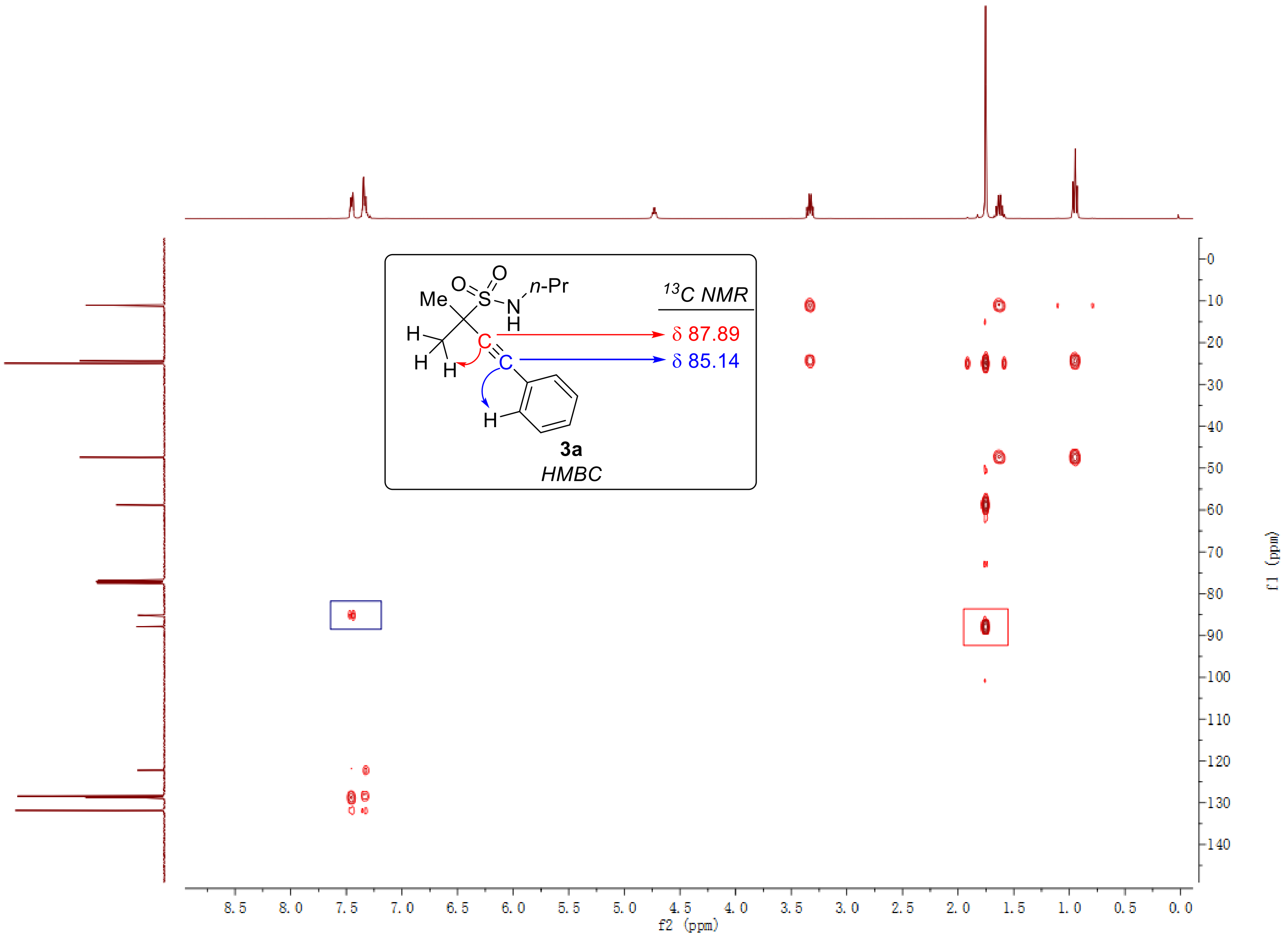
Me
O
O
S
N
H
n-Pr
H
H
H
C
C
H
13C NMR
δ 87.89
δ 85.14
3a
HMBC
f2 (ppm)
f1 (ppm)

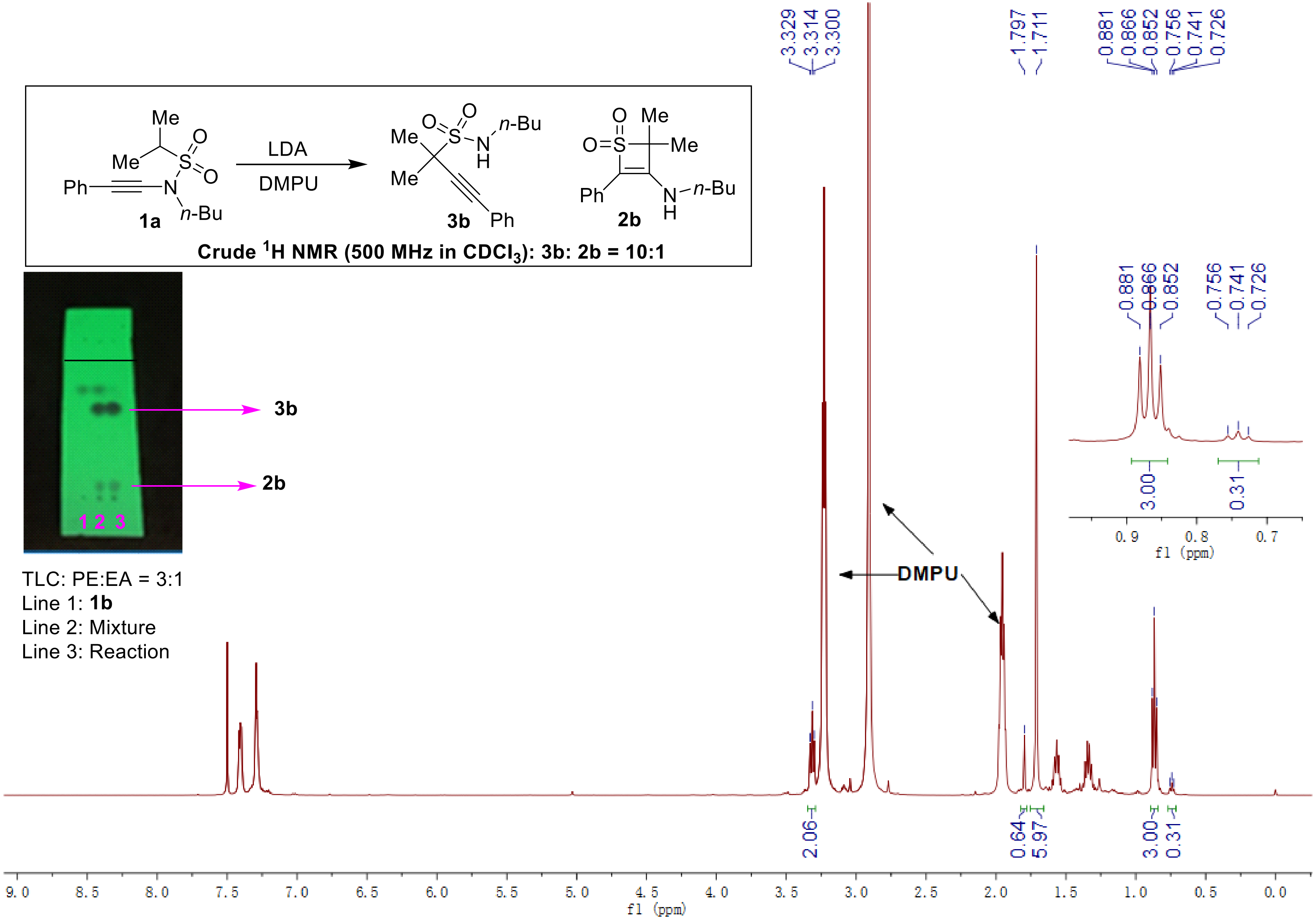

Me
O
Me
S
O
Ph
N
1a
n-Bu
LDA
DMPU
O
O
Me
S
n-Bu
N
H
Me
3b
Ph
O
Me
O=S
Me
Ph
N
n-Bu
H
2b
Crude 1H NMR (500 MHz in CDCl3): 3b: 2b = 10:1
1 2 3
3b
2b
TLC: PE:EA = 3:1
Line 1: 1b
Line 2: Mixture
Line 3: Reaction
DMPU
3.329
3.314
3.300
1.797
1.711
0.881
0.866
0.852
0.756
0.741
0.726
3.00
0.31
0.9
0.8
0.7
f1 (ppm)
2.06
0.64
5.97
3.00
0.31
9.0
8.5
8.0
7.5
7.0
6.5
6.0
5.5
5.0
4.5
4.0
3.5
3.0
2.5
2.0
1.5
1.0
0.5
0.0
f1 (ppm)

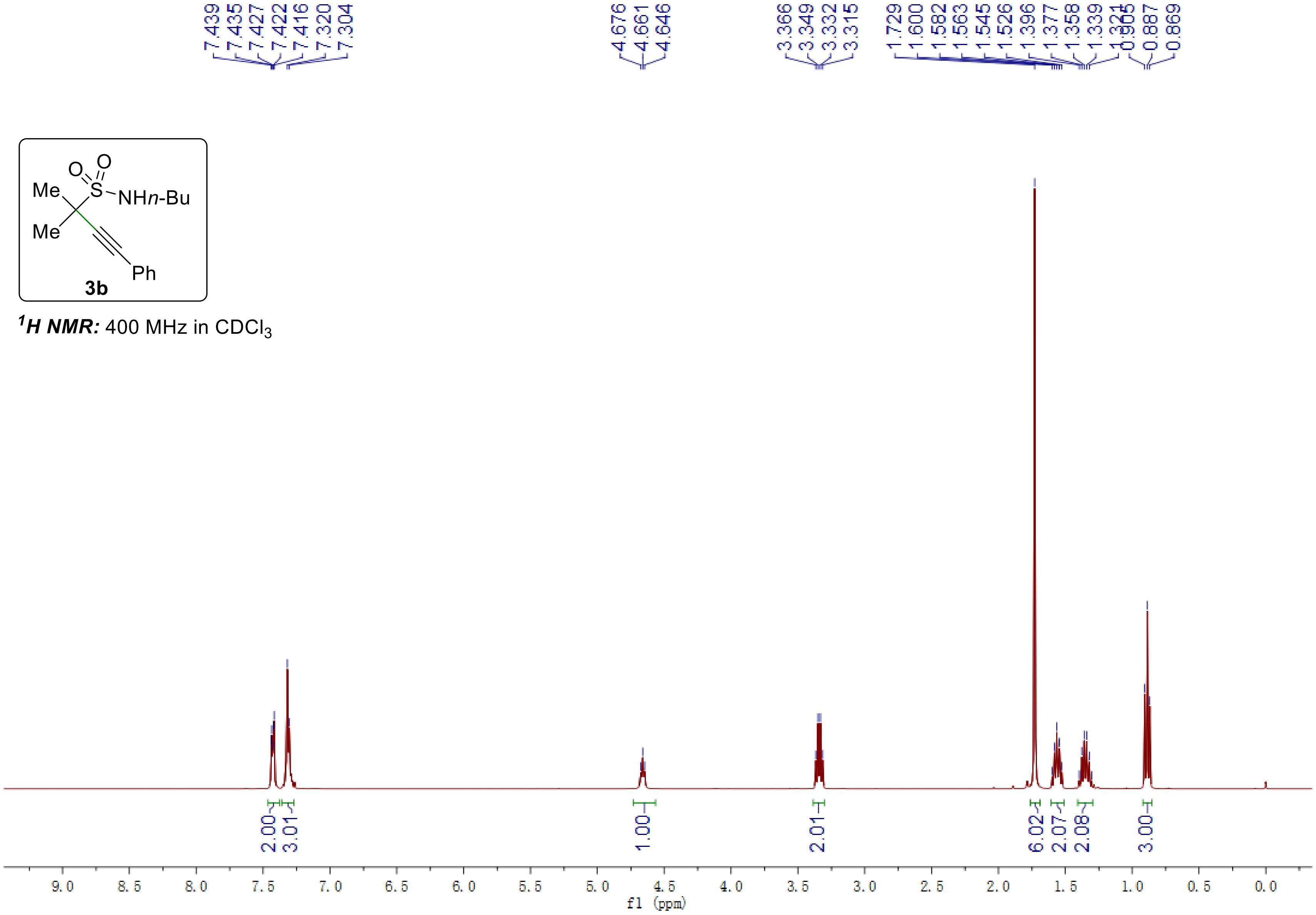

3b

^{1}H NMR: 400 MHz in $CDCl_3$

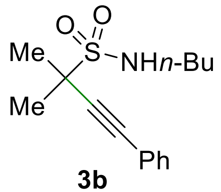

3b

13C NMR: 100 MHz in $CDCl_3$

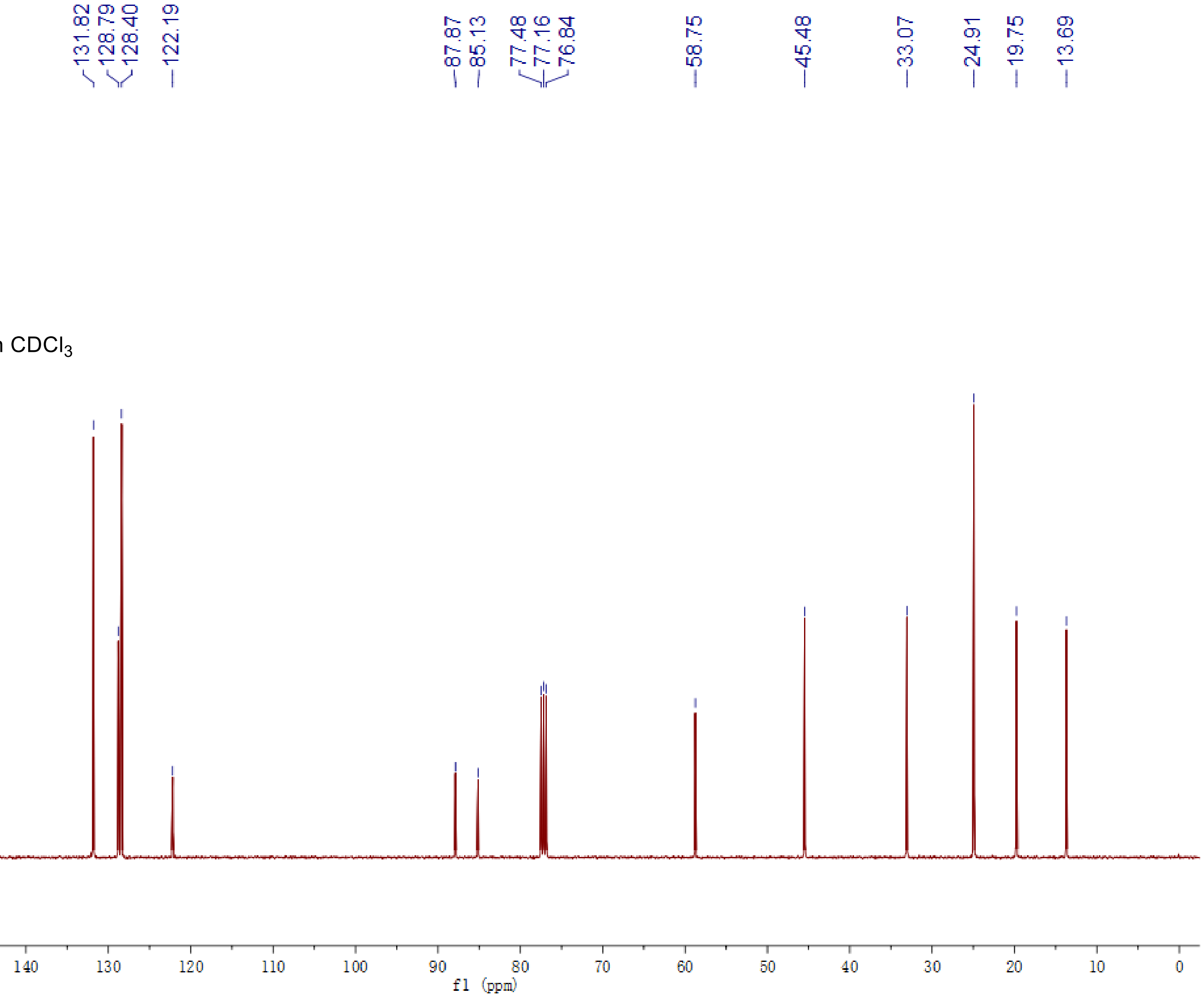

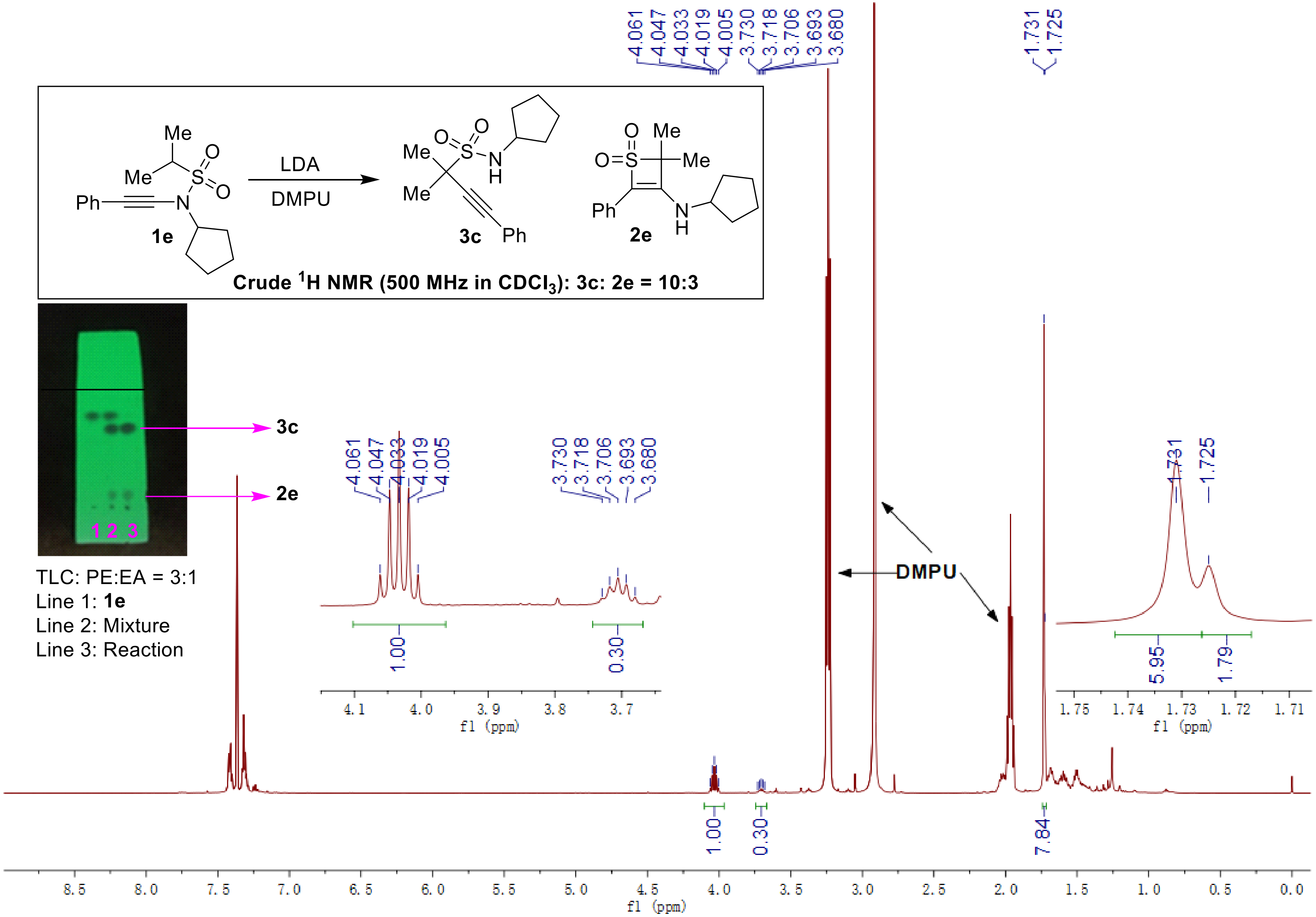
LDA
DMPU
1e
3c
2e
Crude ^{1}H NMR (500 MHz in $CDCl_3$): 3c: 2e = 10:3
TLC: PE:EA = 3:1
Line 1: **1e**
Line 2: Mixture
Line 3: Reaction
DMPU
f1 (ppm)

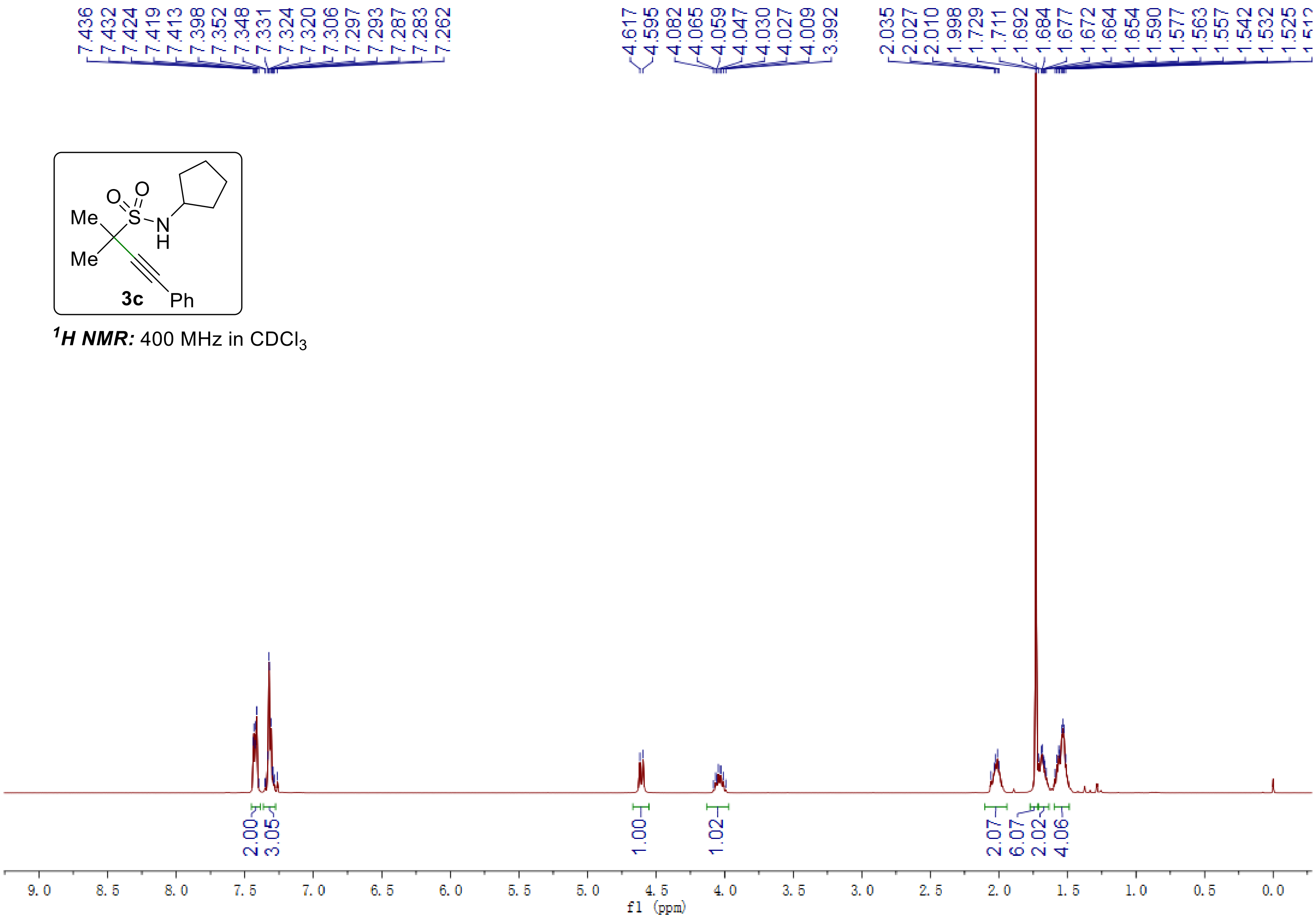

^{1}H NMR: 400 MHz in $CDCl_3$

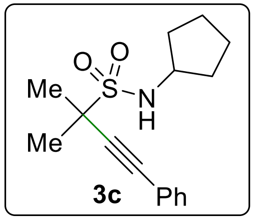

^{13}***C NMR:*** 100 MHz in $CDCl_3$

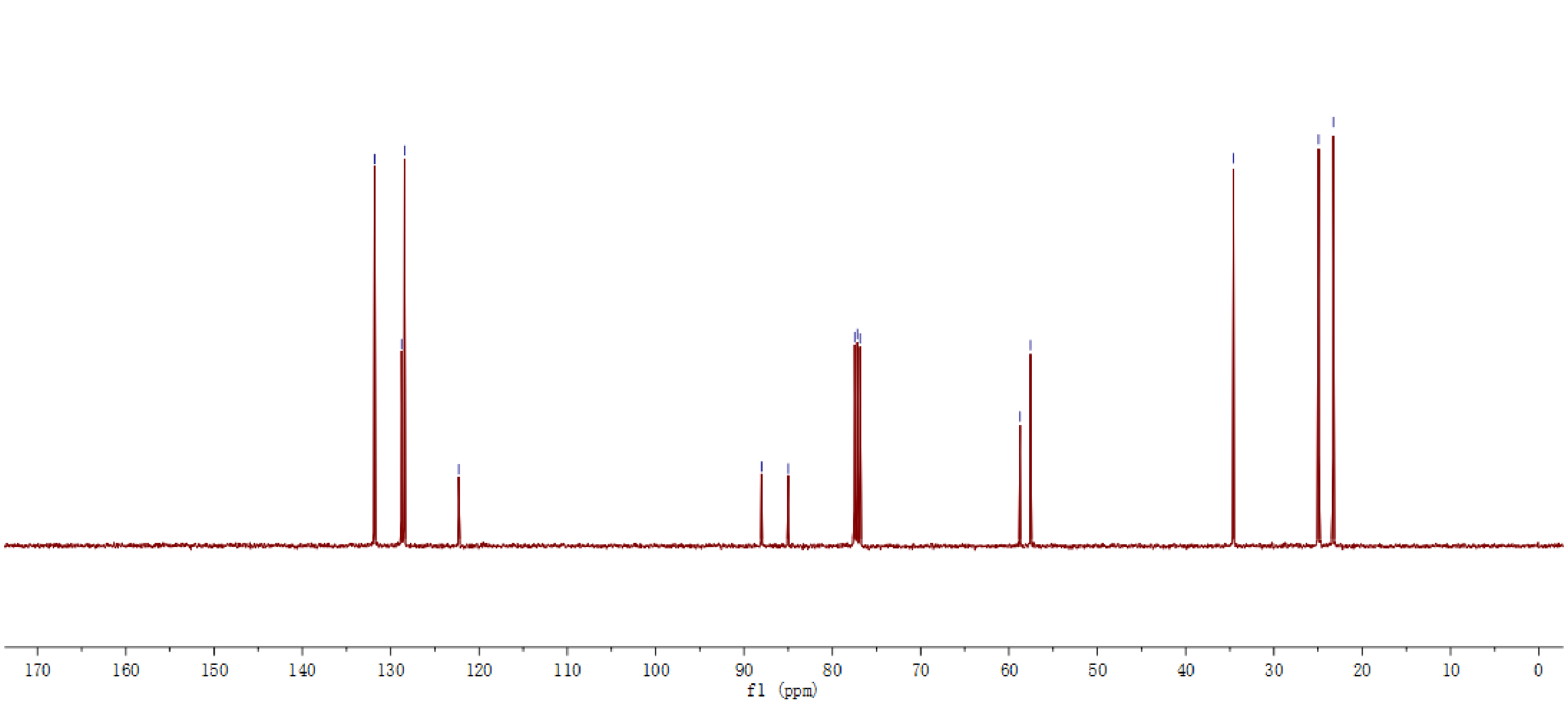

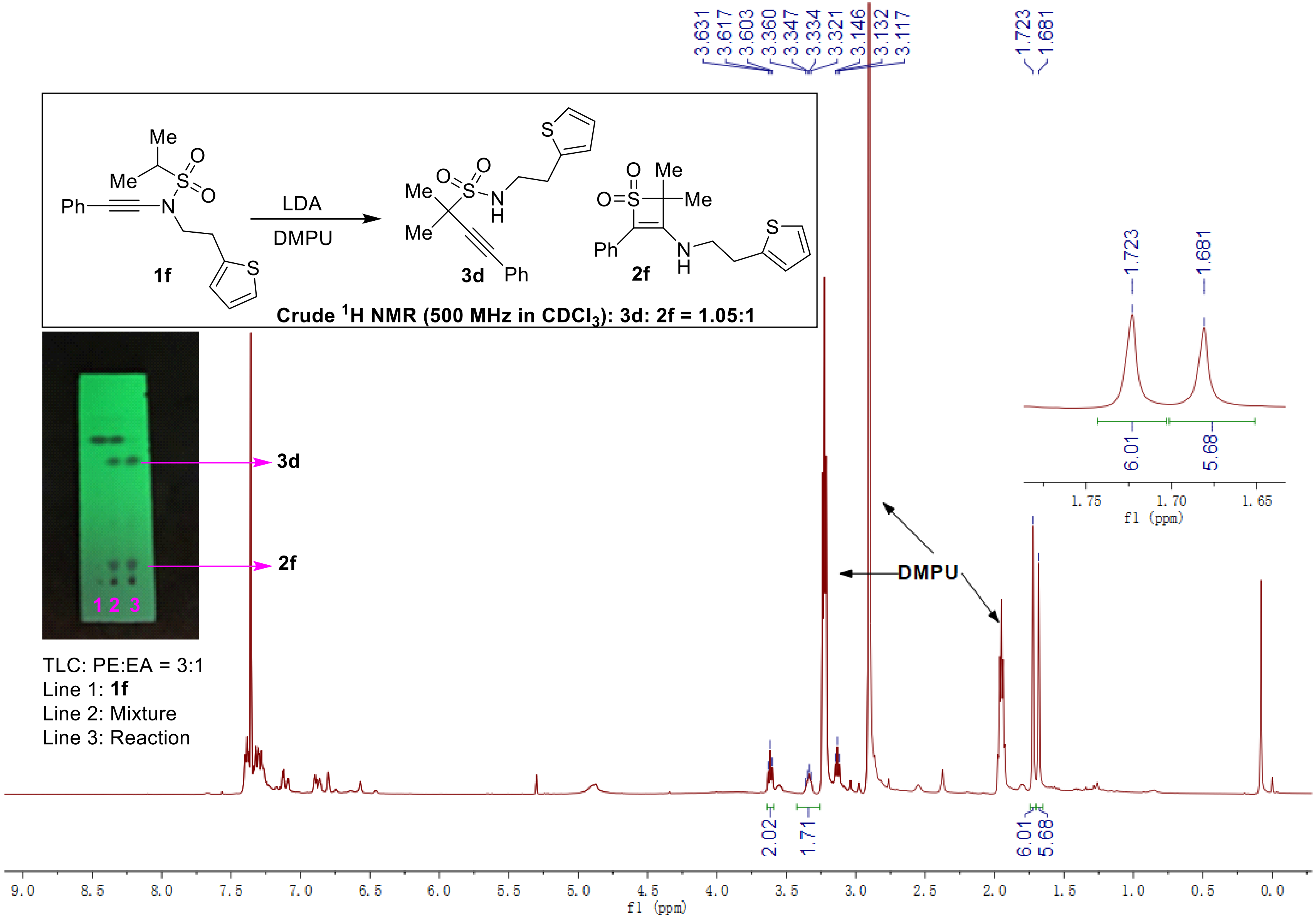
LDA
DMPU
1f
3d
2f
Crude ¹H NMR (500 MHz in CDCl3): 3d: 2f = 1.05:1
TLC: PE:EA = 3:1
Line 1: 1f
Line 2: Mixture
Line 3: Reaction
DMPU
3.631 3.617 3.603 3.360 3.347 3.334 3.321 3.146 3.132 3.117
1.723 1.681
2.02 1.71 6.01 5.68
f1 (ppm)

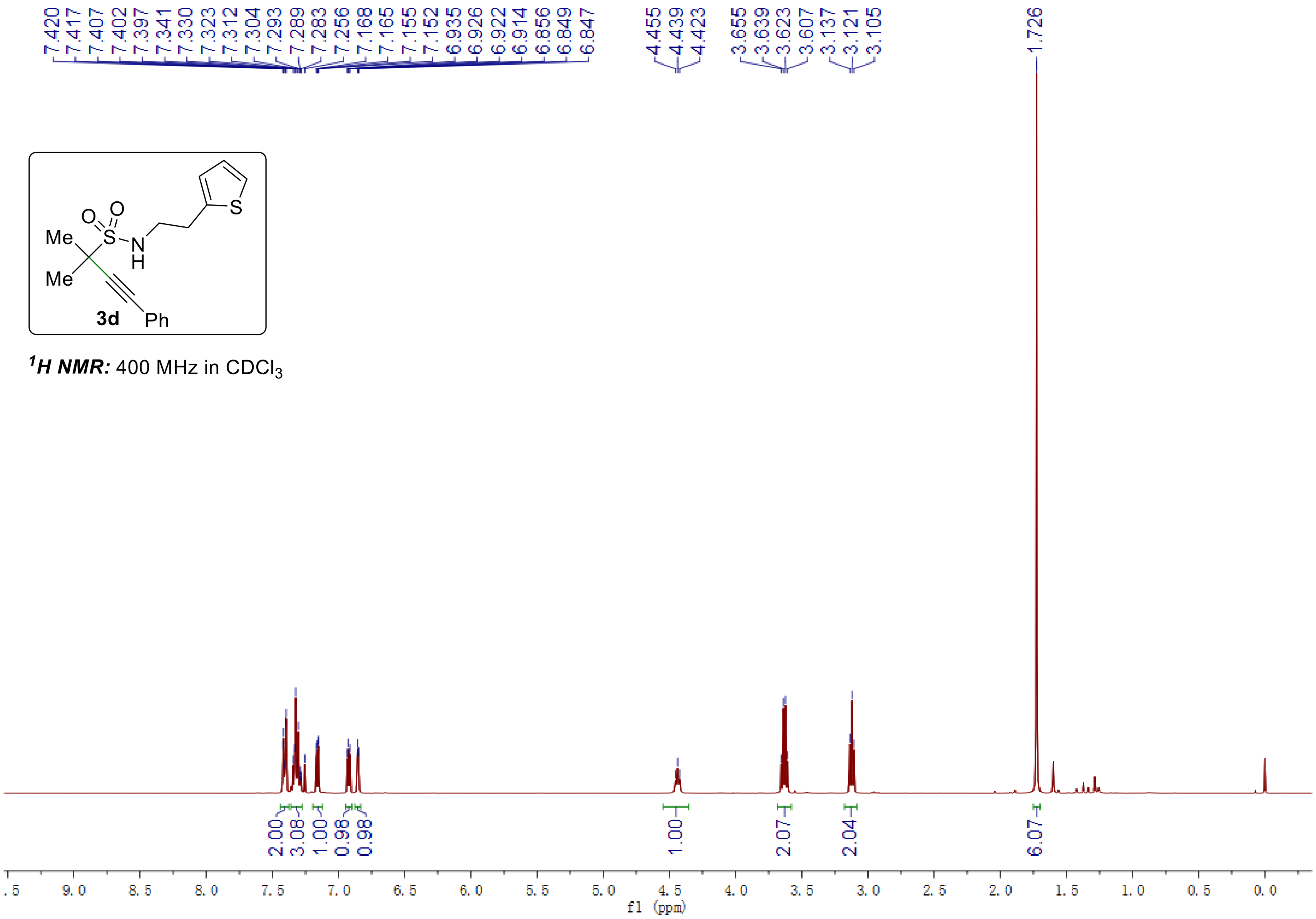

[1]*H NMR:* 400 MHz in $CDCl_3$

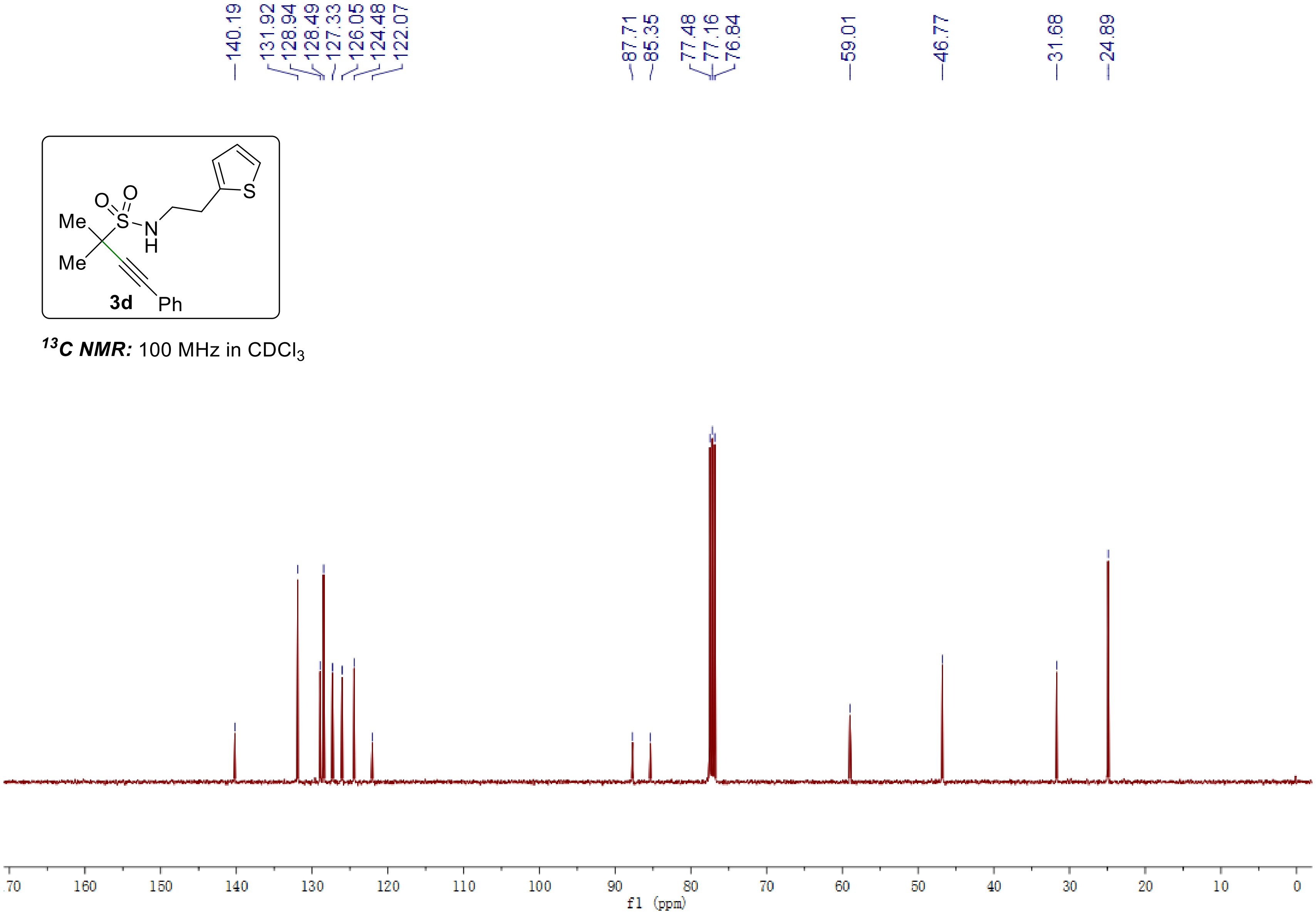

^{13}***C NMR:*** 100 MHz in $CDCl_3$

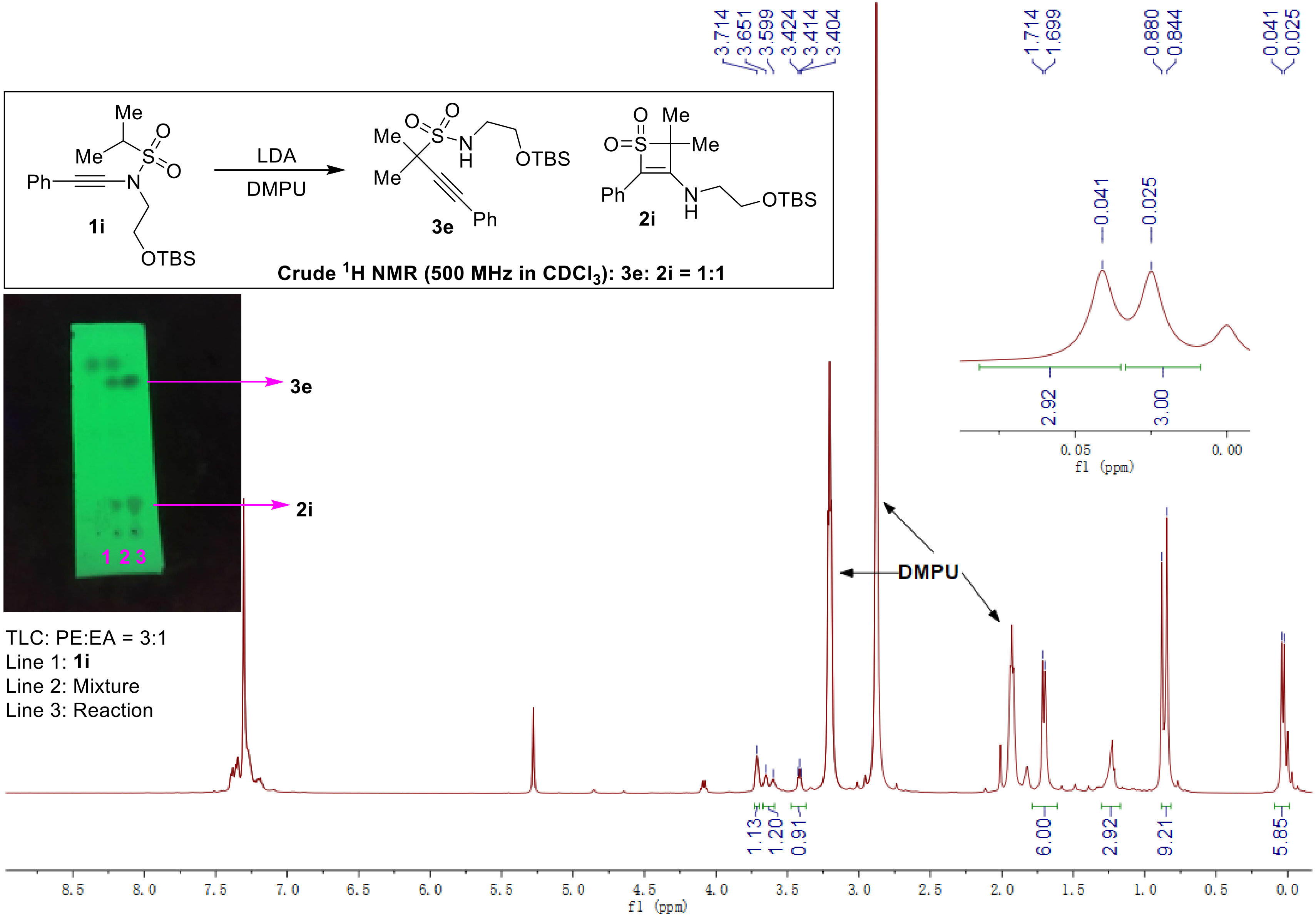
Ph
Me
Me
S
O
O
N
OTBS
1i
LDA
DMPU
Me
Me
O
O
S
N
H
OTBS
Ph
3e
O
Me
O=S
Me
Ph
N
H
OTBS
2i
Crude ¹H NMR (500 MHz in CDCl₃): 3e: 2i = 1:1
3e
2i
1 2 3
TLC: PE:EA = 3:1
Line 1: 1i
Line 2: Mixture
Line 3: Reaction
DMPU
3.714
3.651
3.599
3.424
3.414
3.404
1.714
1.699
0.880
0.844
0.041
0.025
1.13
1.20
0.91
6.00
2.92
9.21
5.85
2.92
3.00
0.05
0.00
f1 (ppm)
8.5
8.0
7.5
7.0
6.5
6.0
5.5
5.0
4.5
4.0
3.5
3.0
2.5
2.0
1.5
1.0
0.5
0.0
f1 (ppm)

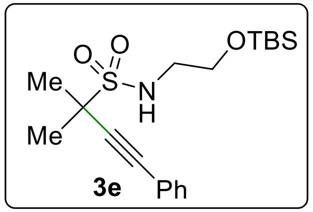

^{1}H NMR: 400 MHz in $CDCl_3$

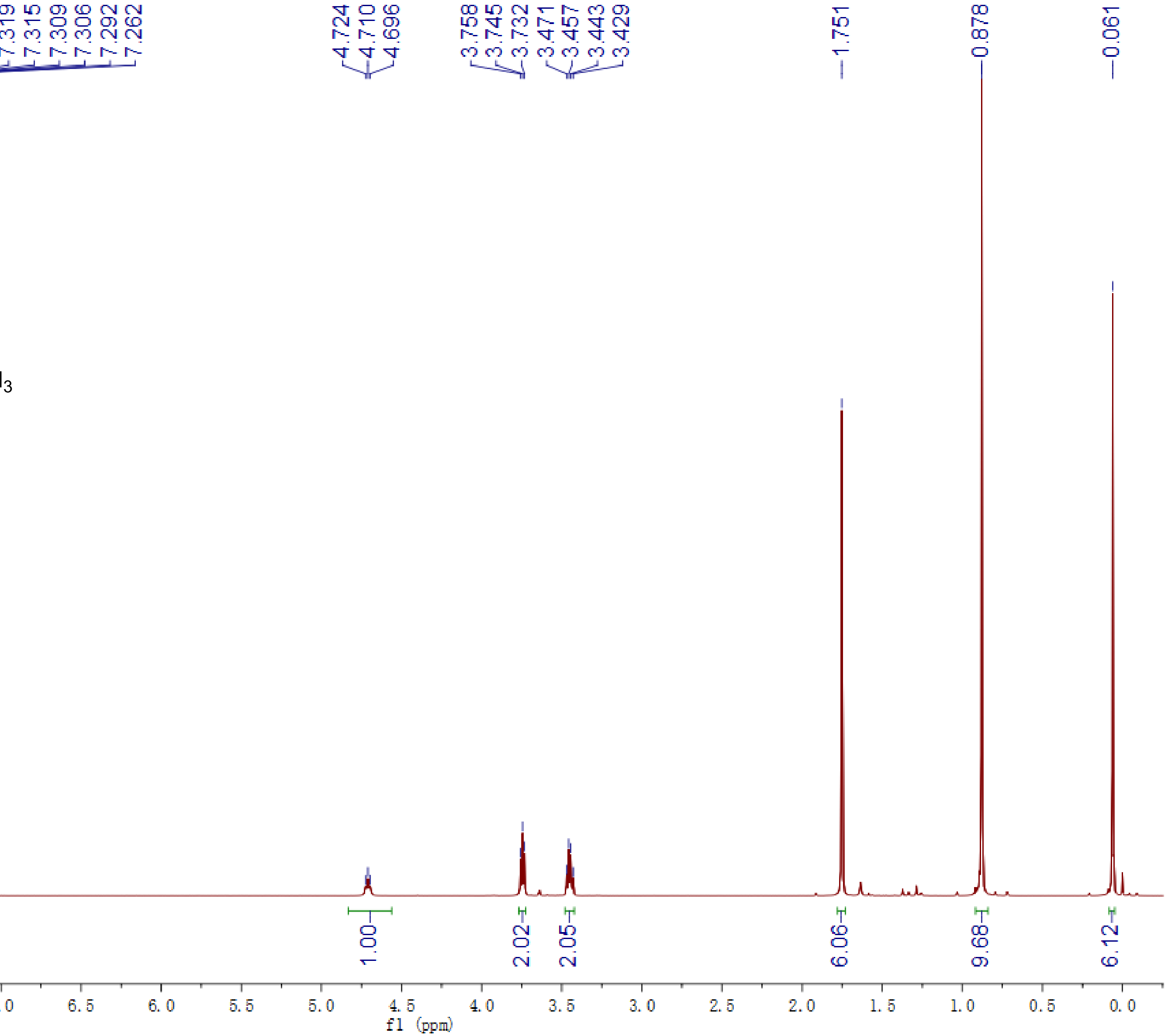

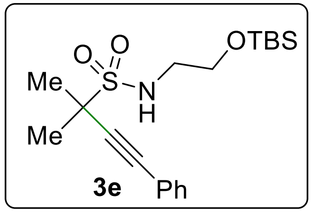

^{13}***C NMR:*** 100 MHz in $CDCl_3$

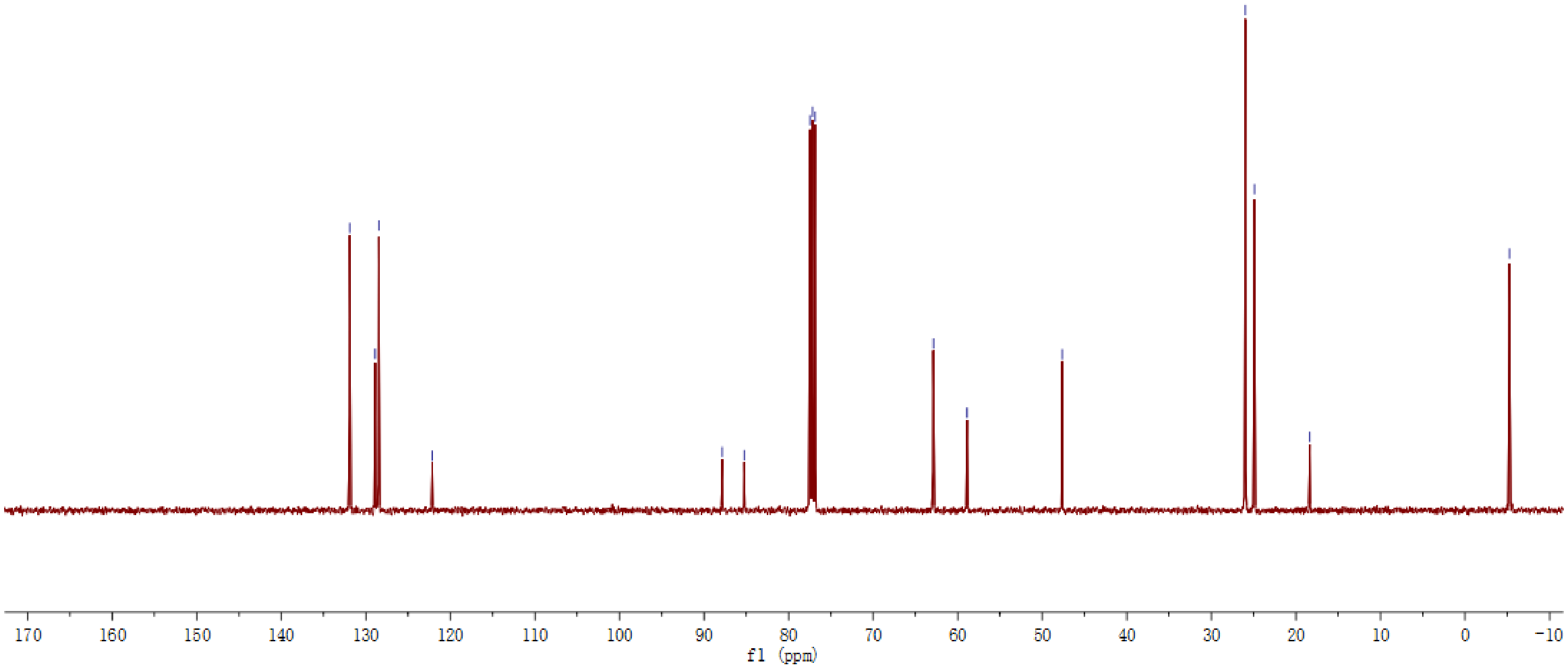

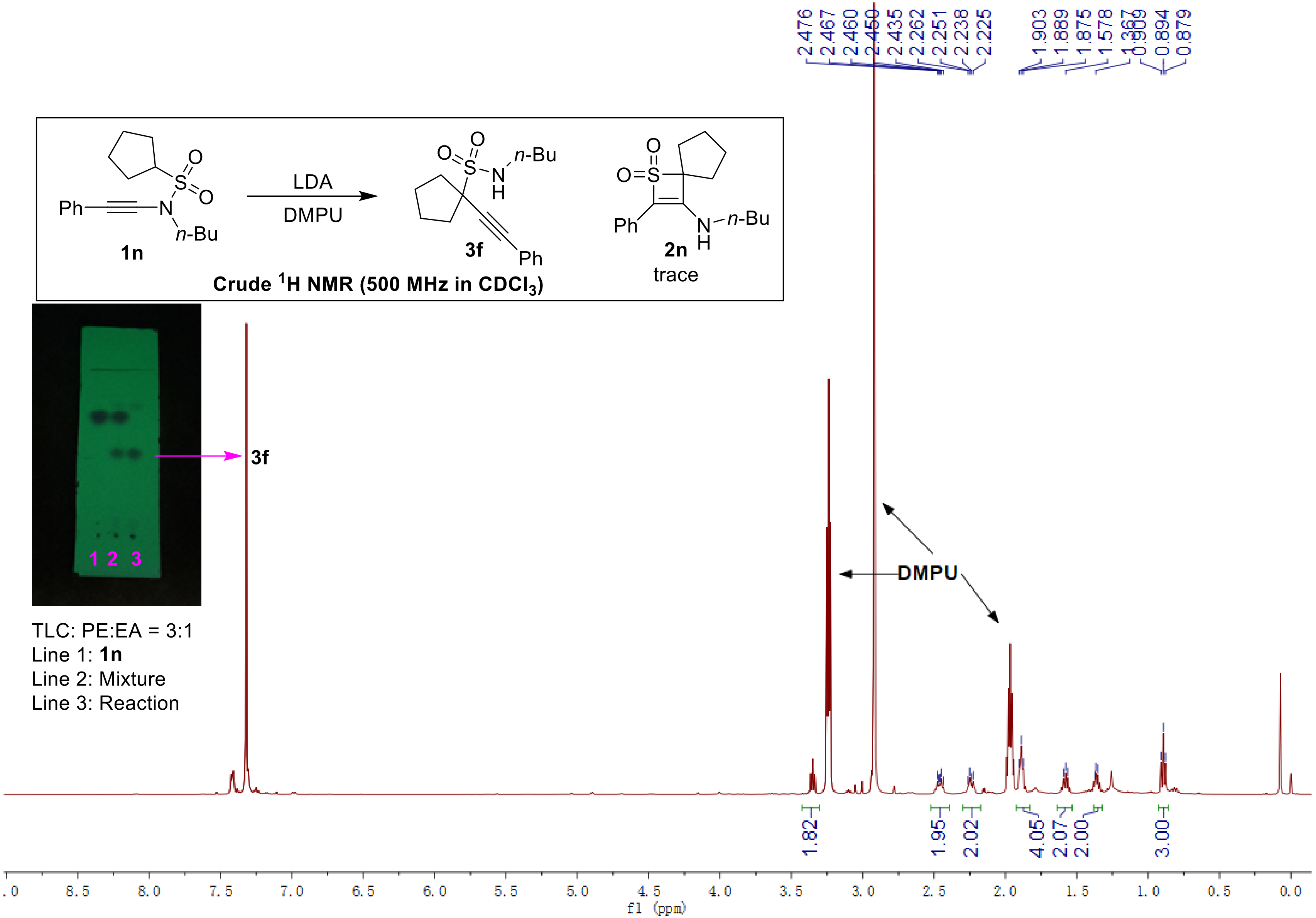
LDA
DMPU
1n
3f
2n
trace
Crude ¹H NMR (500 MHz in CDCl₃)
TLC: PE:EA = 3:1
Line 1: 1n
Line 2: Mixture
Line 3: Reaction
DMPU
f1 (ppm)

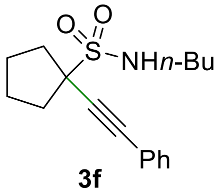

3f

^{1}H NMR: 500 MHz in $CDCl_3$

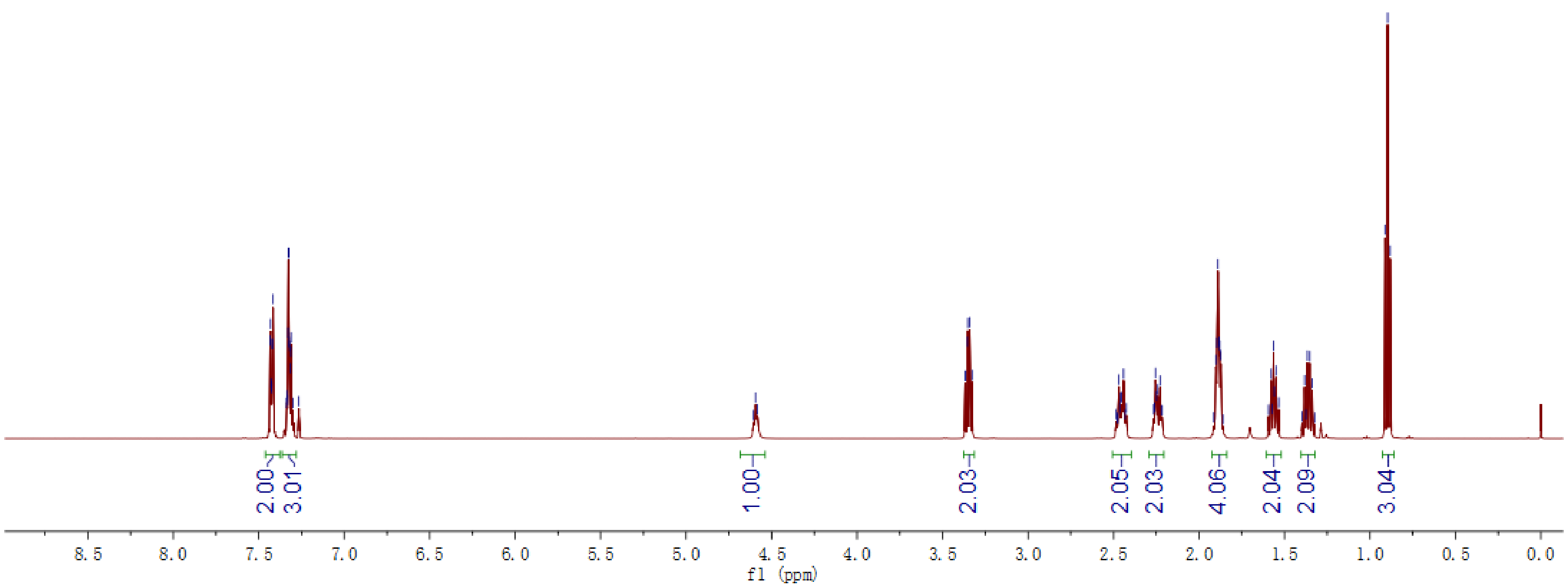

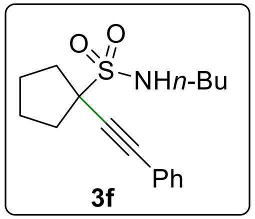

^{13}C NMR: 125 MHz in $CDCl_3$

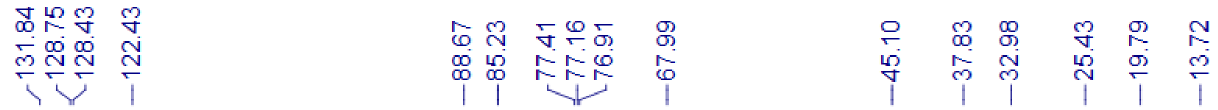

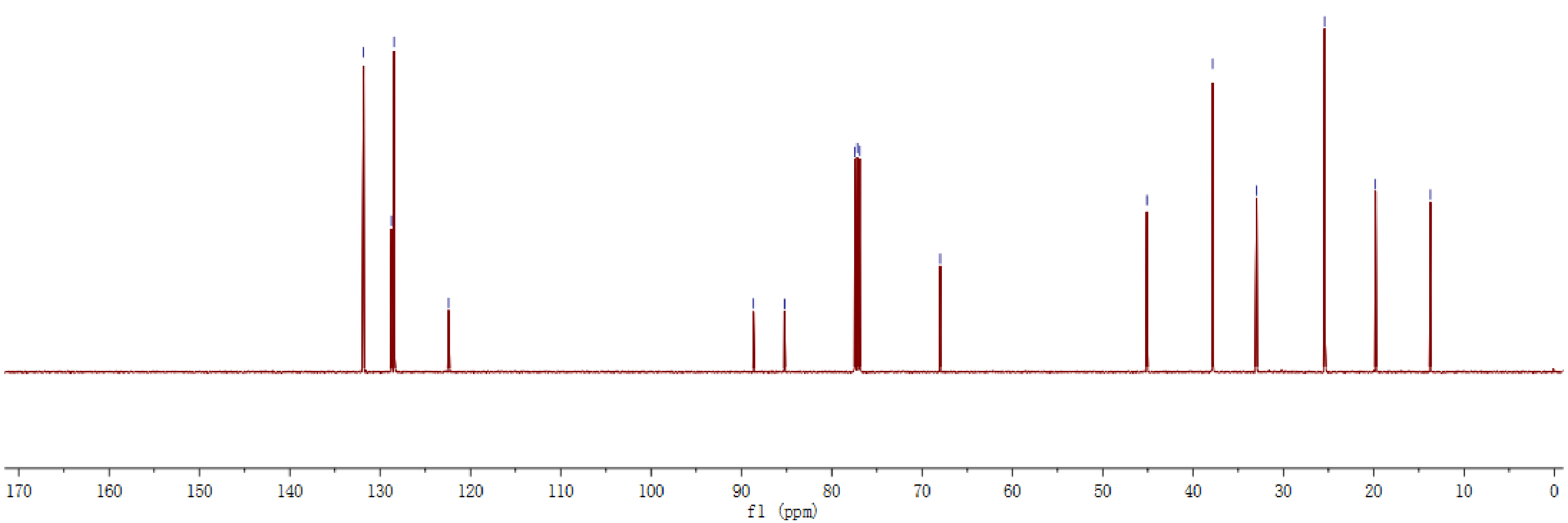

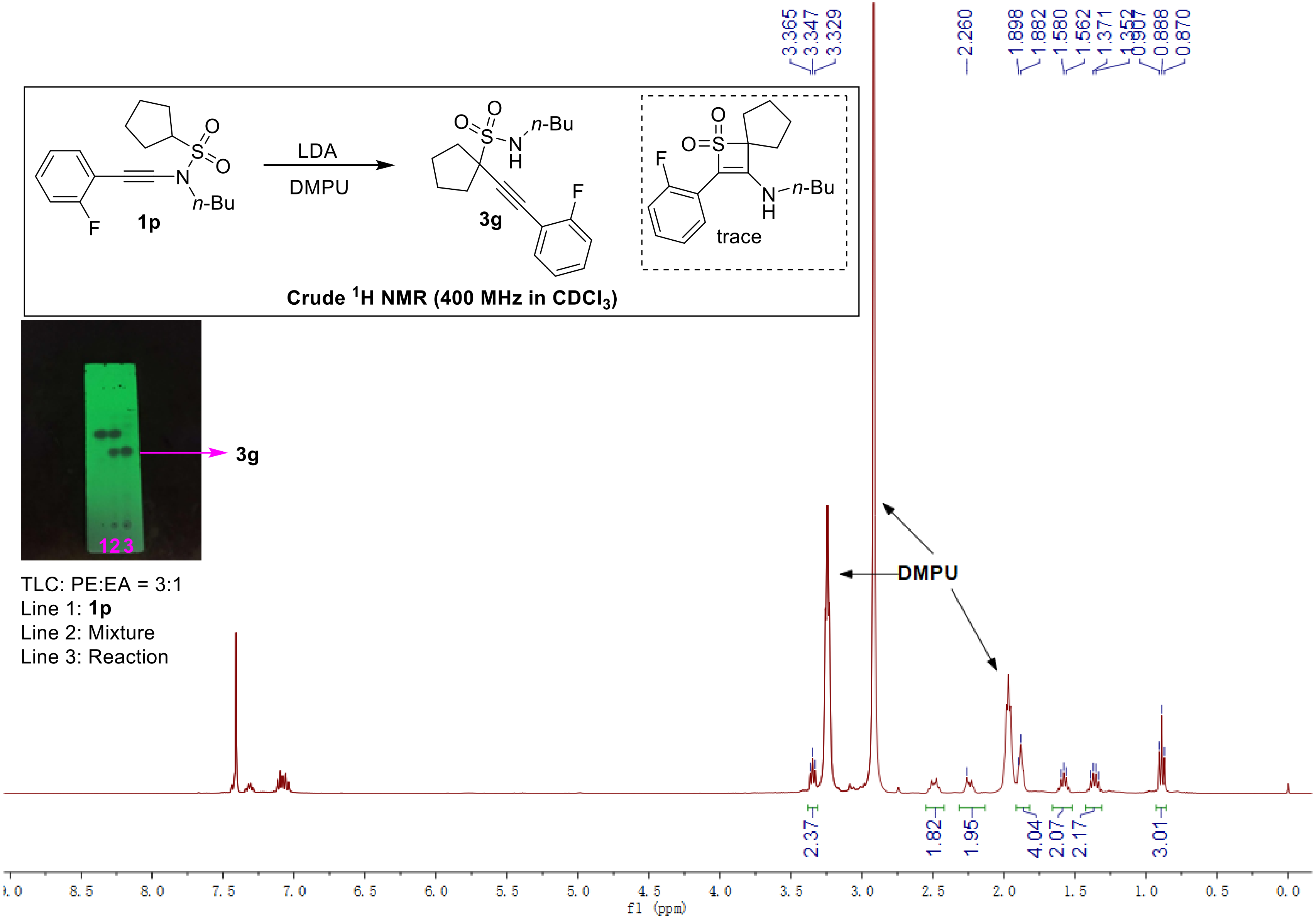
LDA
DMPU
1p
3g
n-Bu
trace
Crude ¹H NMR (400 MHz in CDCl₃)
3g
123
TLC: PE:EA = 3:1
Line 1: 1p
Line 2: Mixture
Line 3: Reaction
DMPU
3.365
3.347
3.329
2.260
1.898
1.882
1.580
1.562
1.371
1.352
0.907
0.888
0.870
2.37
1.82
1.95
4.04
2.07
2.17
3.01
f1 (ppm)

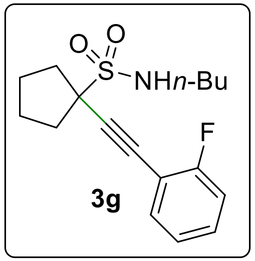

1H NMR: 500 MHz in $CDCl_3$

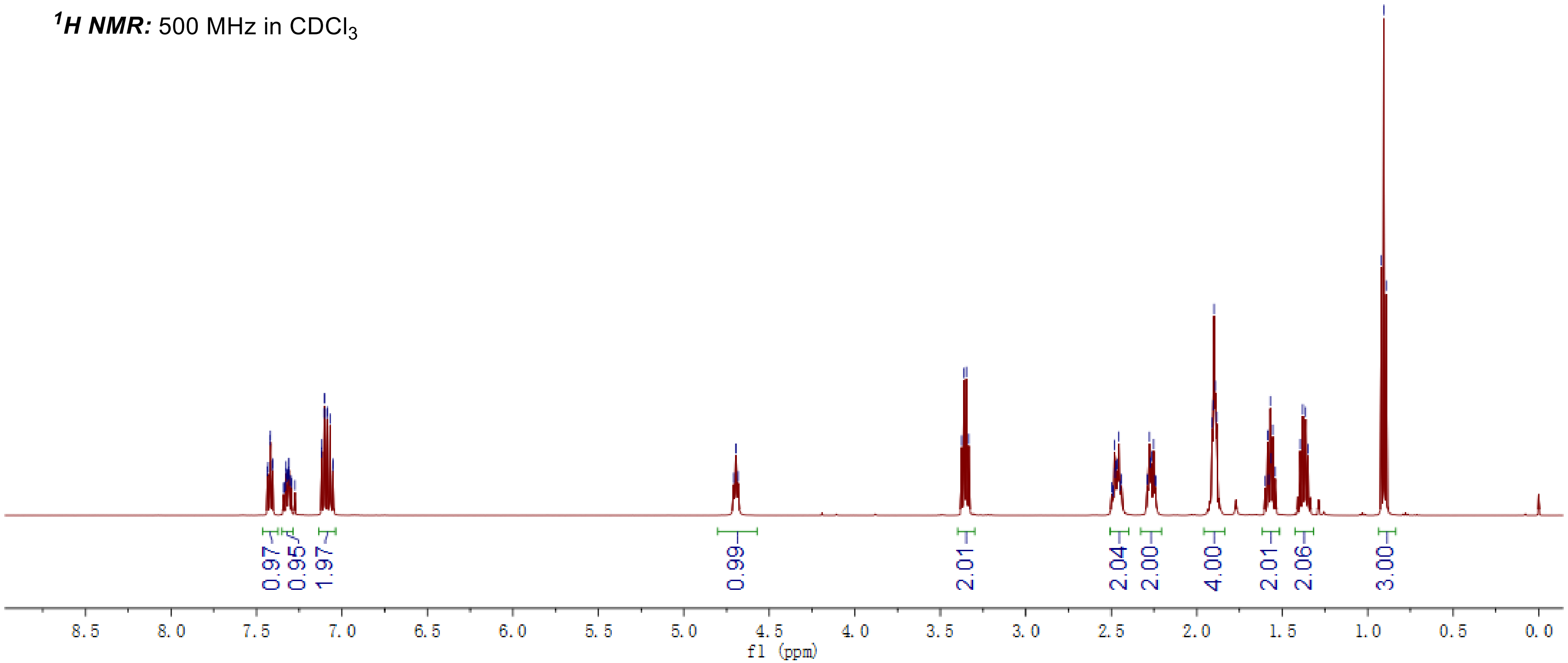

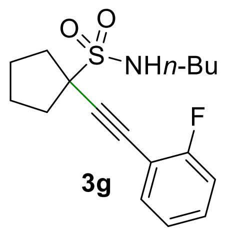

3g

^{13}***C NMR:*** 125 MHz in $CDCl_3$

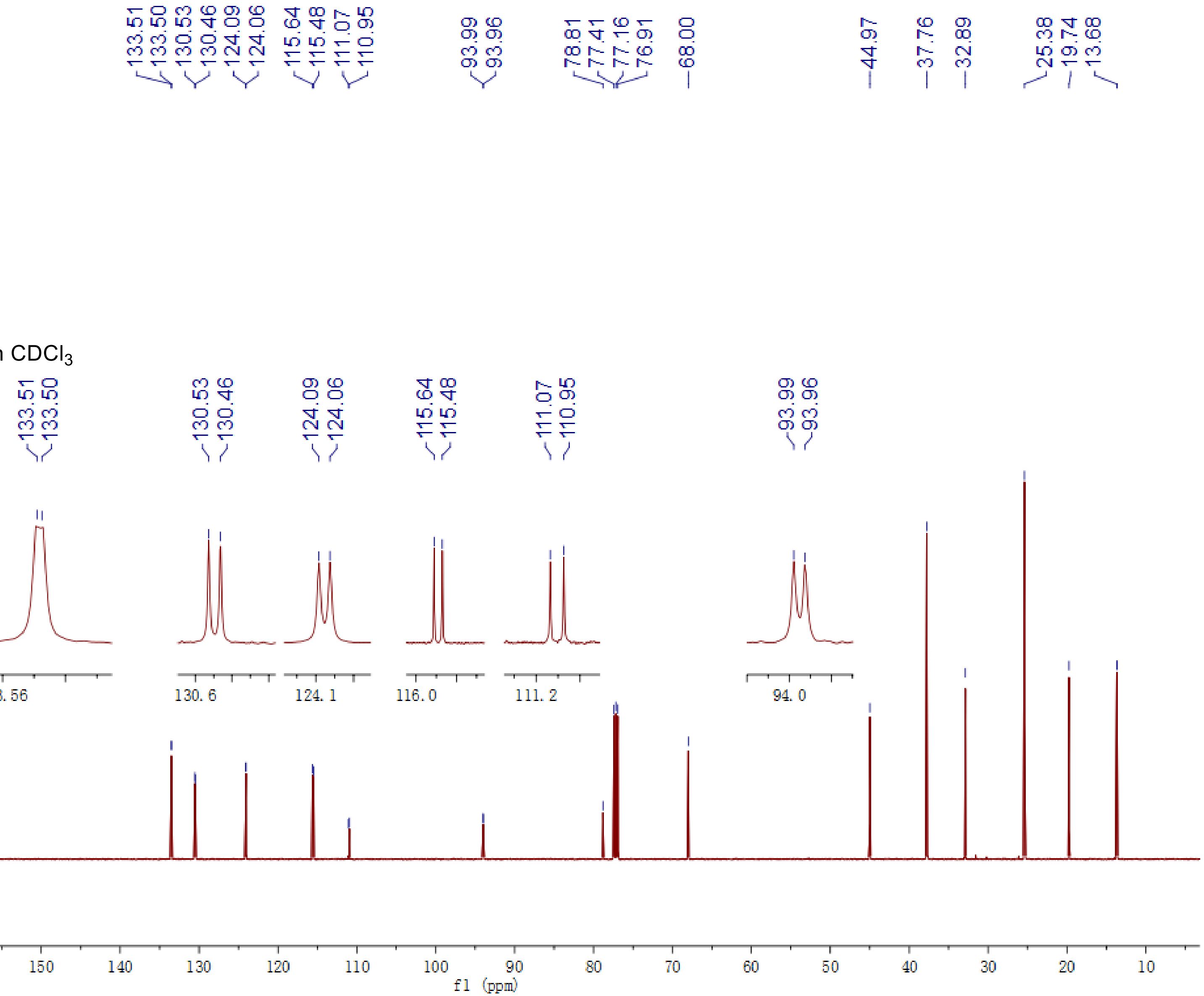

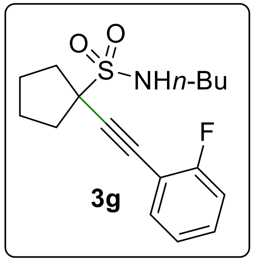

^{19}F NMR: 376 MHz in $CDCl_3$

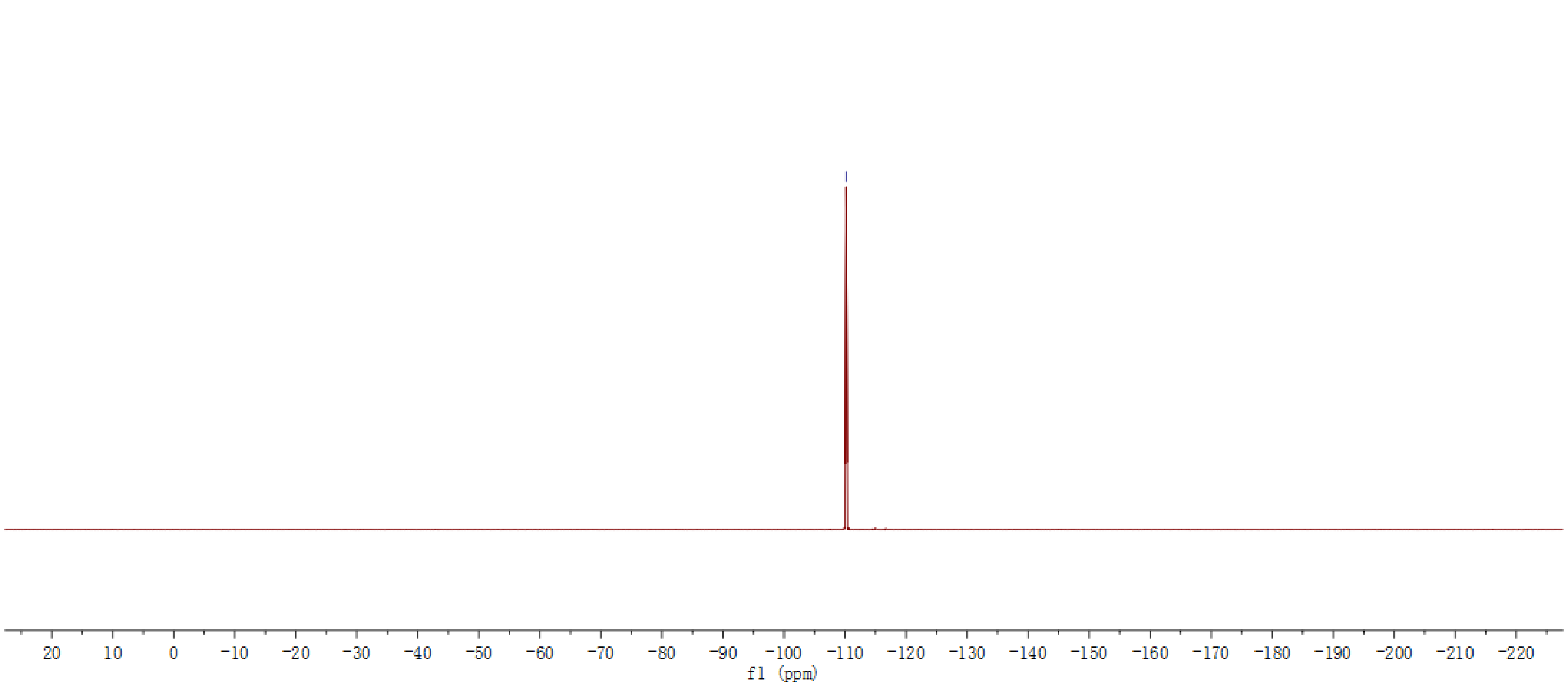

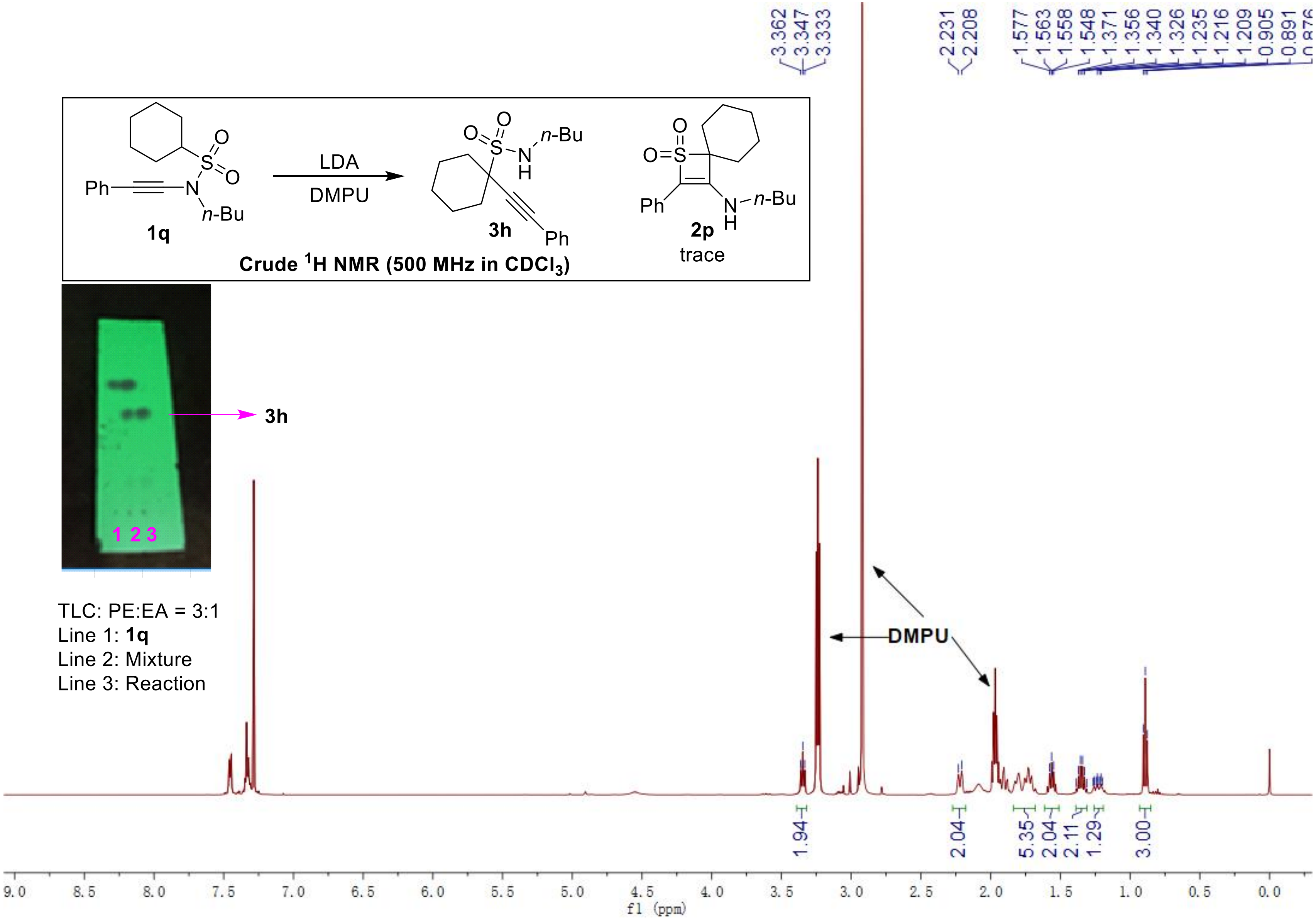

LDA
DMPU
1q
3h
2p
trace
Crude ¹H NMR (500 MHz in CDCl3)
3h
1 2 3
TLC: PE:EA = 3:1
Line 1: 1q
Line 2: Mixture
Line 3: Reaction
DMPU
3.362
3.347
3.333
2.231
2.208
1.577
1.563
1.558
1.548
1.371
1.356
1.340
1.326
1.235
1.216
1.209
0.905
0.891
1.94
2.04
5.35
2.04
2.11
1.29
3.00
f1 (ppm)

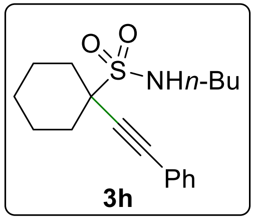

^{1}H NMR: 400 MHz in $CDCl_3$

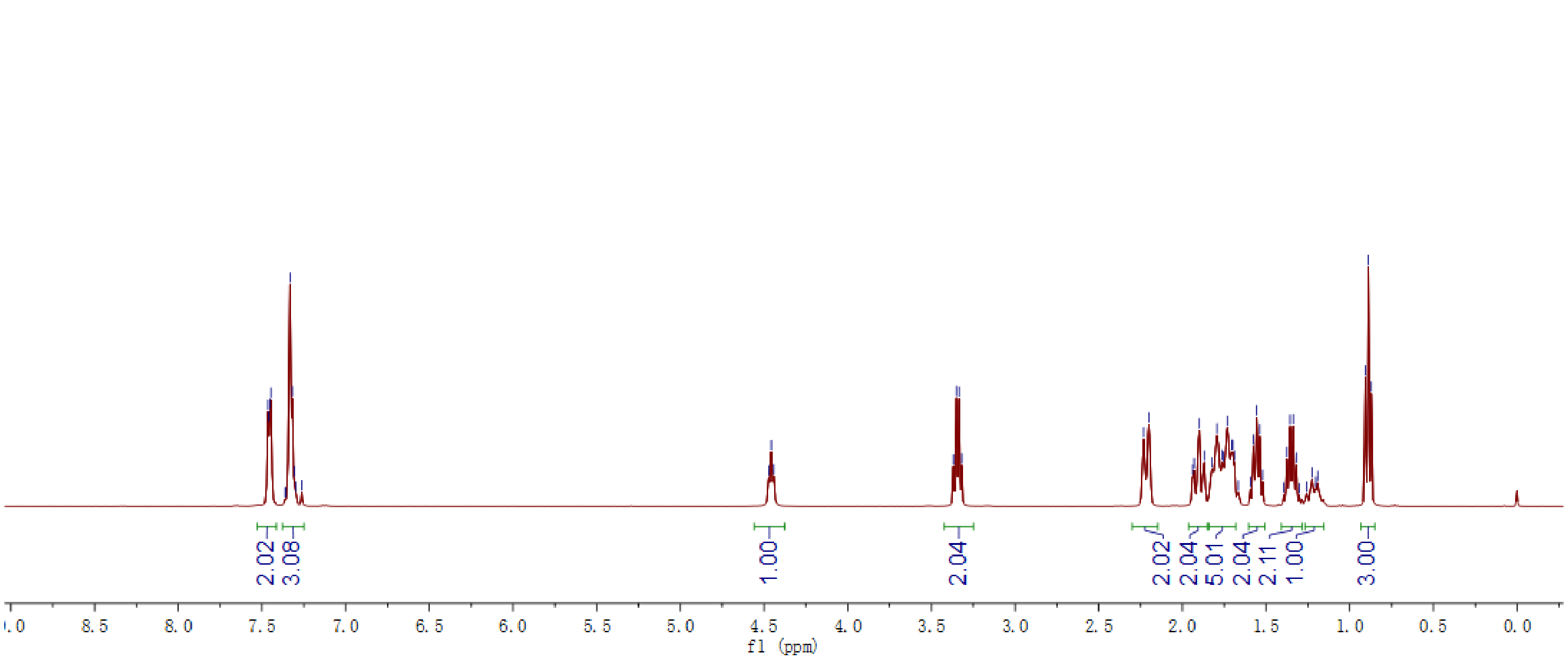

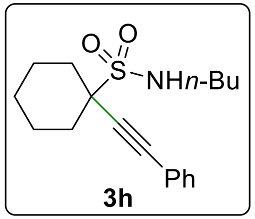

^{13}C NMR: 100 MHz in $CDCl_3$

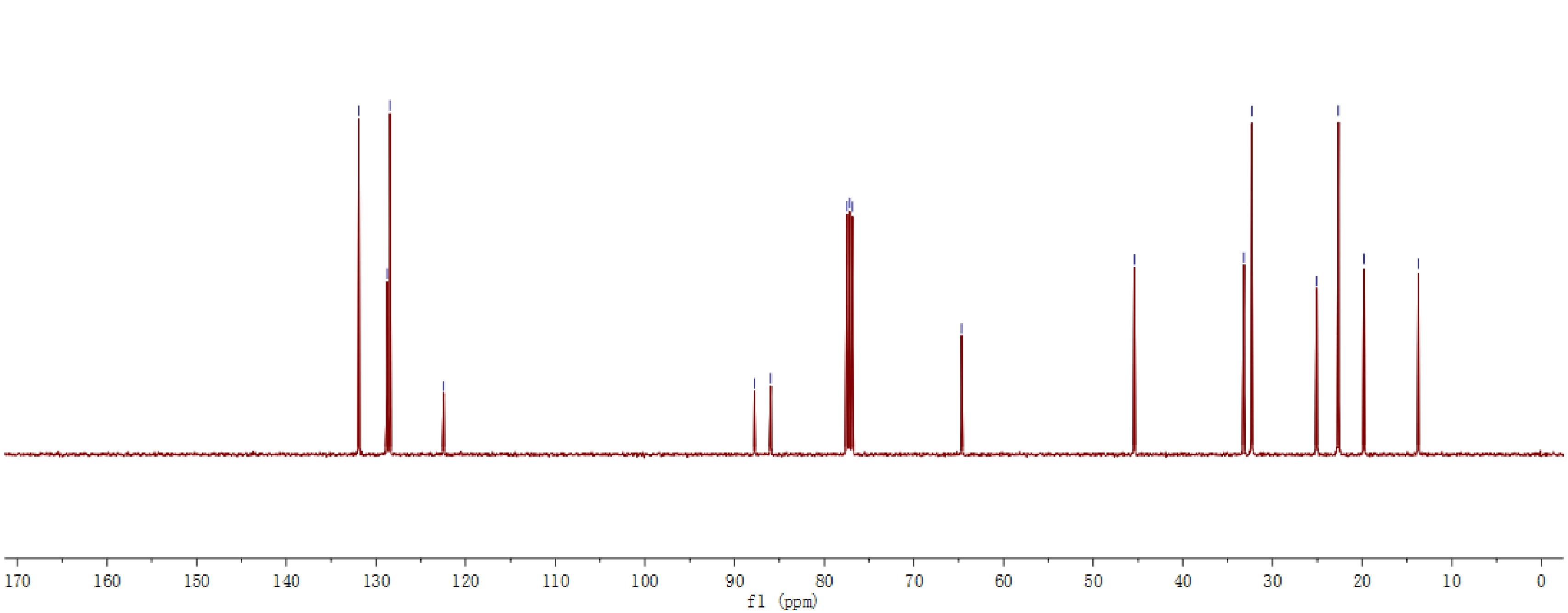

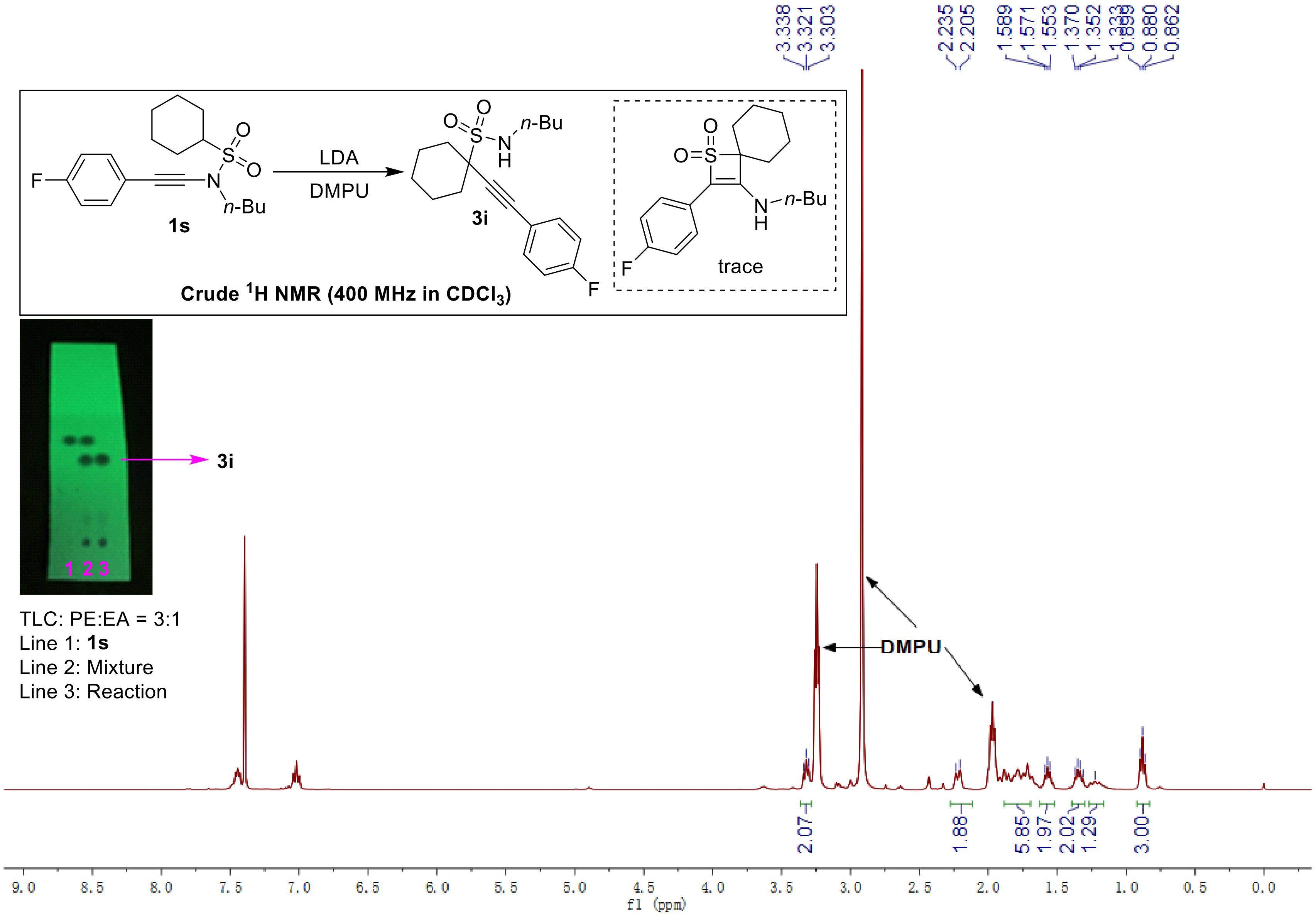
LDA
DMPU
1s
3i
n-Bu
trace
Crude ^{1}H NMR (400 MHz in CDCl$_3$)
3i
1 2 3
TLC: PE:EA = 3:1
Line 1: **1s**
Line 2: Mixture
Line 3: Reaction
DMPU
3.338
3.321
3.303
2.235
2.205
1.589
1.571
1.553
1.370
1.352
1.333
0.899
0.880
0.862
2.07
1.88
5.85
1.97
2.02
1.29
3.00
9.0 8.5 8.0 7.5 7.0 6.5 6.0 5.5 5.0 4.5 4.0 3.5 3.0 2.5 2.0 1.5 1.0 0.5 0.0
f1 (ppm)

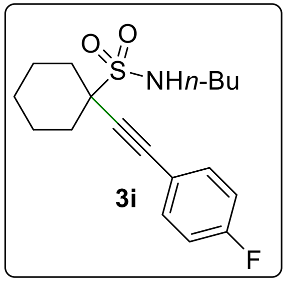

3i

^{1}***H NMR:*** 500 MHz in $CDCl_3$

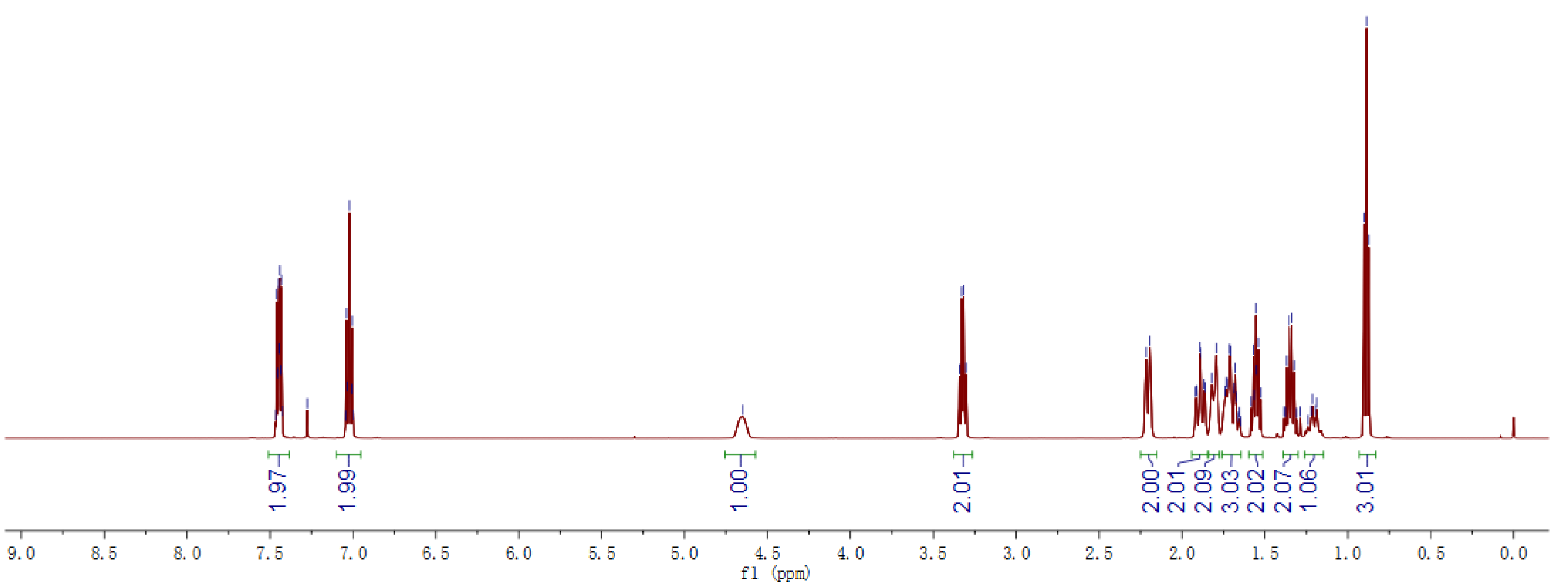

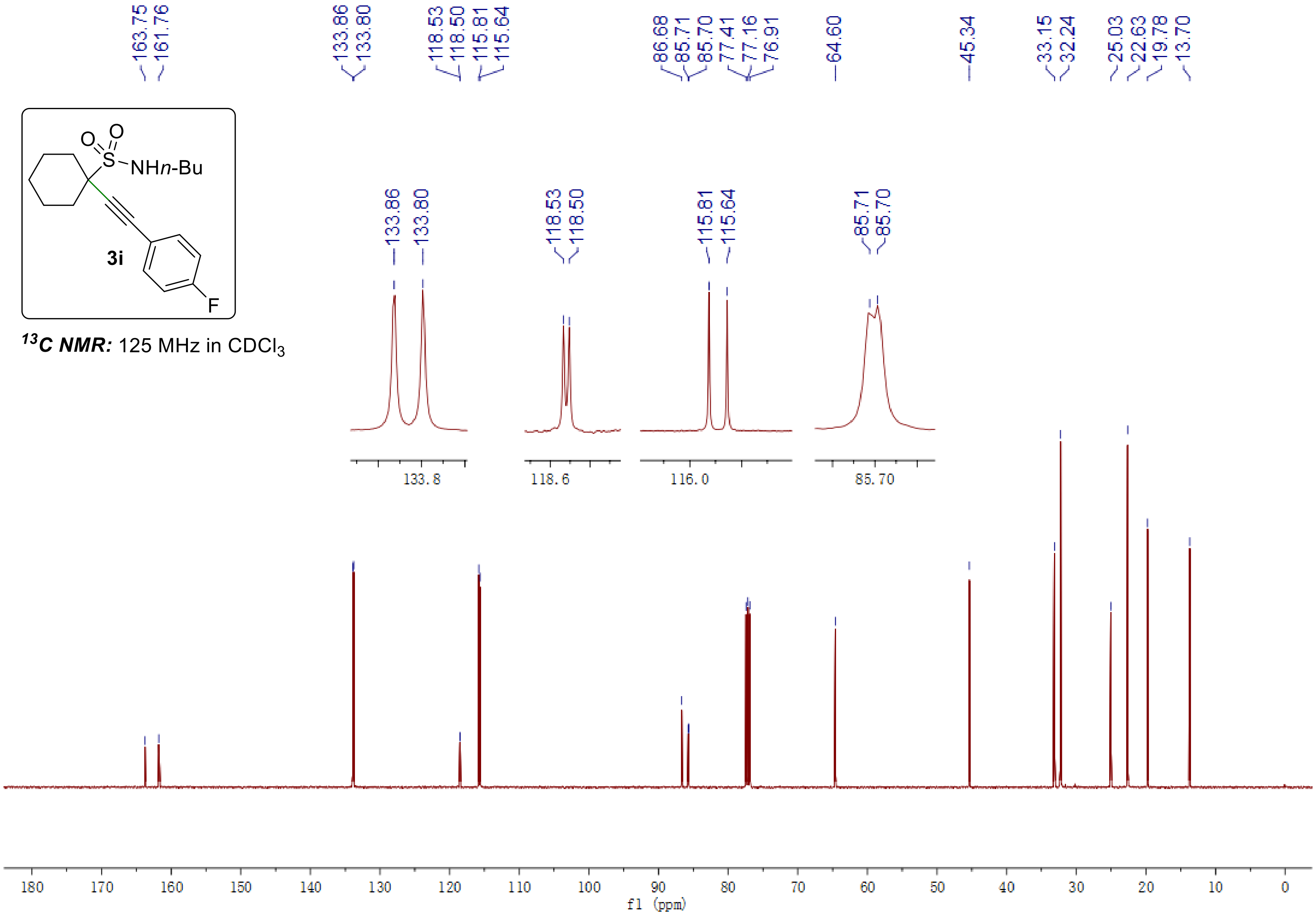
163.75
161.76
133.86
133.80
118.53
118.50
115.81
115.64
86.68
85.71
85.70
77.41
77.16
76.91
64.60
45.34
33.15
32.24
25.03
22.63
19.78
13.70
O
O
S
NHn-Bu
3i
F
^{13}C NMR: 125 MHz in $CDCl_3$
133.8
118.6
116.0
85.70
180
170
160
150
140
130
120
110
100
90
80
70
60
50
40
30
20
10
0
f1 (ppm)

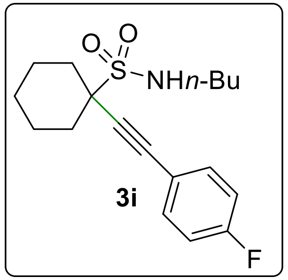

^{19}F NMR: 376 MHz in $CDCl_3$

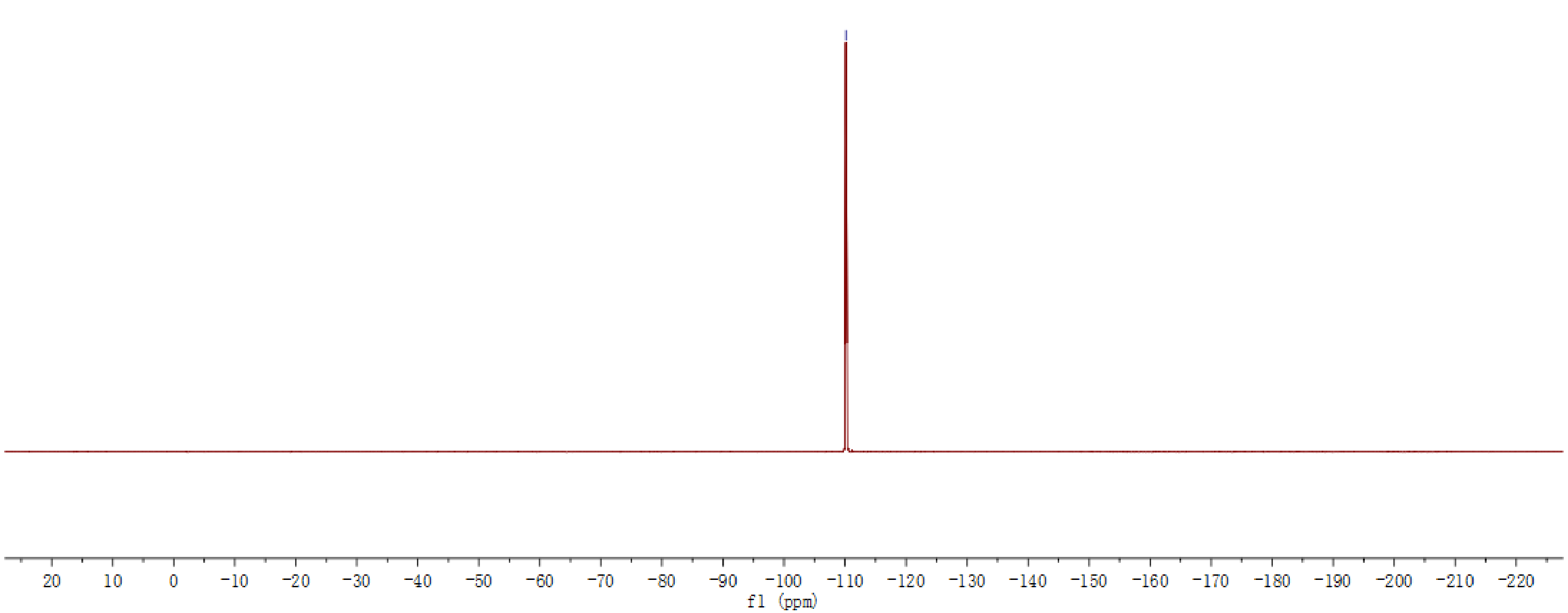

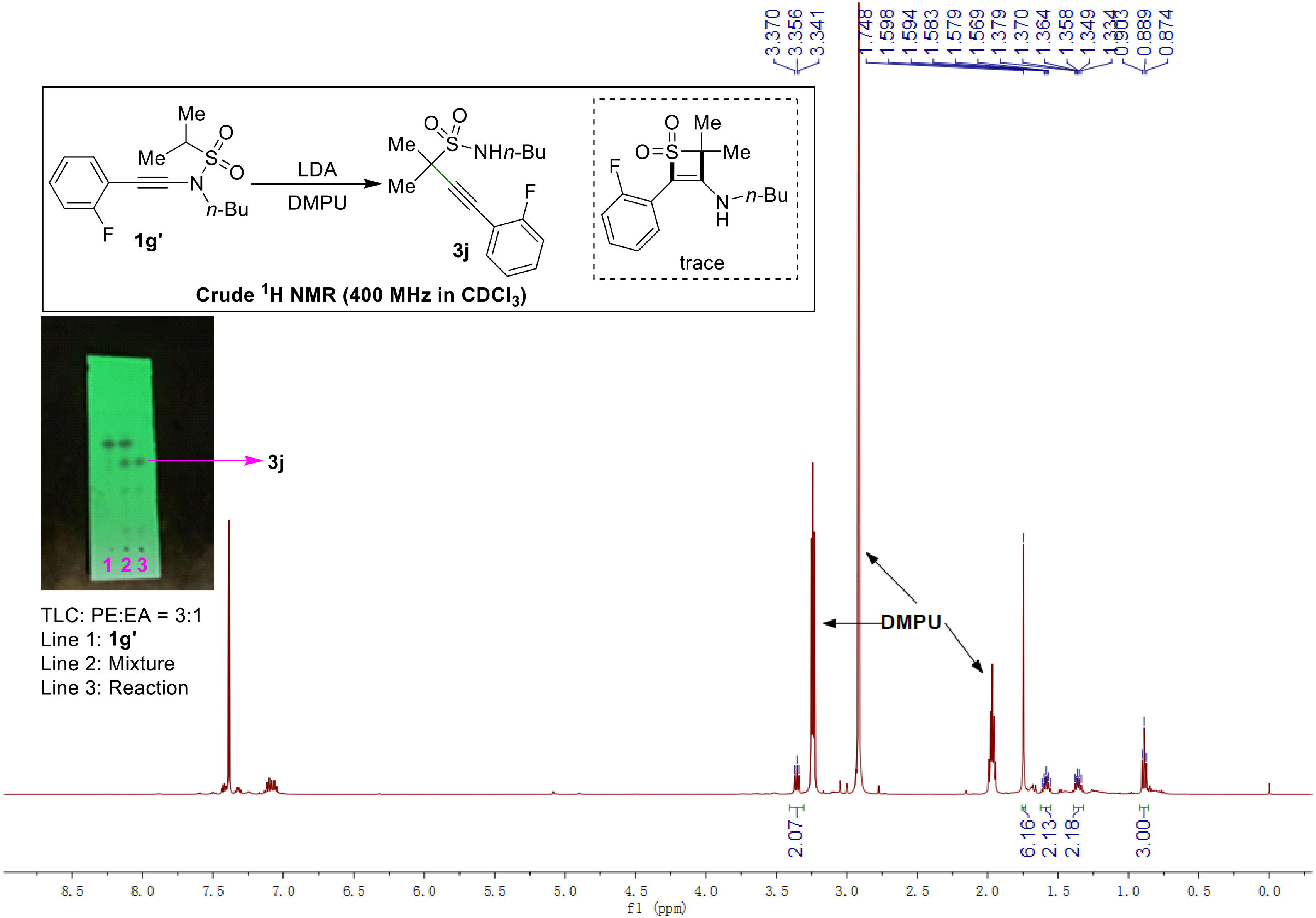
LDA
DMPU
1g'
3j
trace
Crude ¹H NMR (400 MHz in CDCl3)
3j
1 2 3
TLC: PE:EA = 3:1
Line 1: 1g'
Line 2: Mixture
Line 3: Reaction
DMPU
3.370
3.356
3.341
1.748
1.598
1.594
1.583
1.579
1.569
1.379
1.370
1.364
1.358
1.349
1.334
0.903
0.889
0.874
2.07
6.16
2.13
2.18
3.00
8.5
8.0
7.5
7.0
6.5
6.0
5.5
5.0
4.5
4.0
3.5
3.0
2.5
2.0
1.5
1.0
0.5
0.0
f1 (ppm)

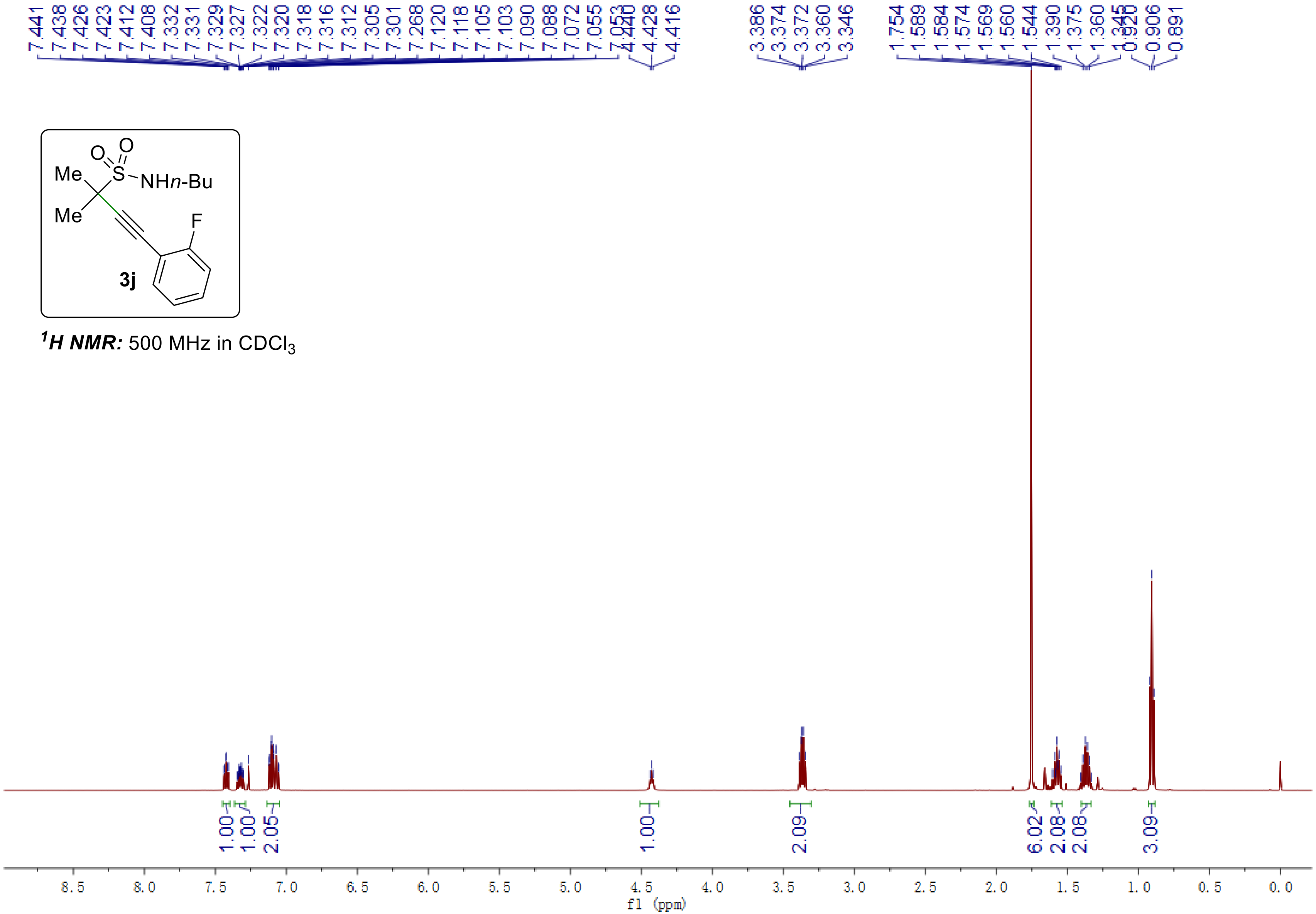
3j
1H NMR: 500 MHz in CDCl3

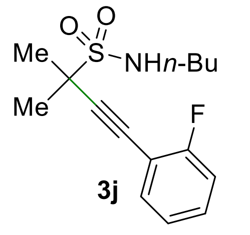

3j

^{13}C NMR: 125 MHz in $CDCl_3$

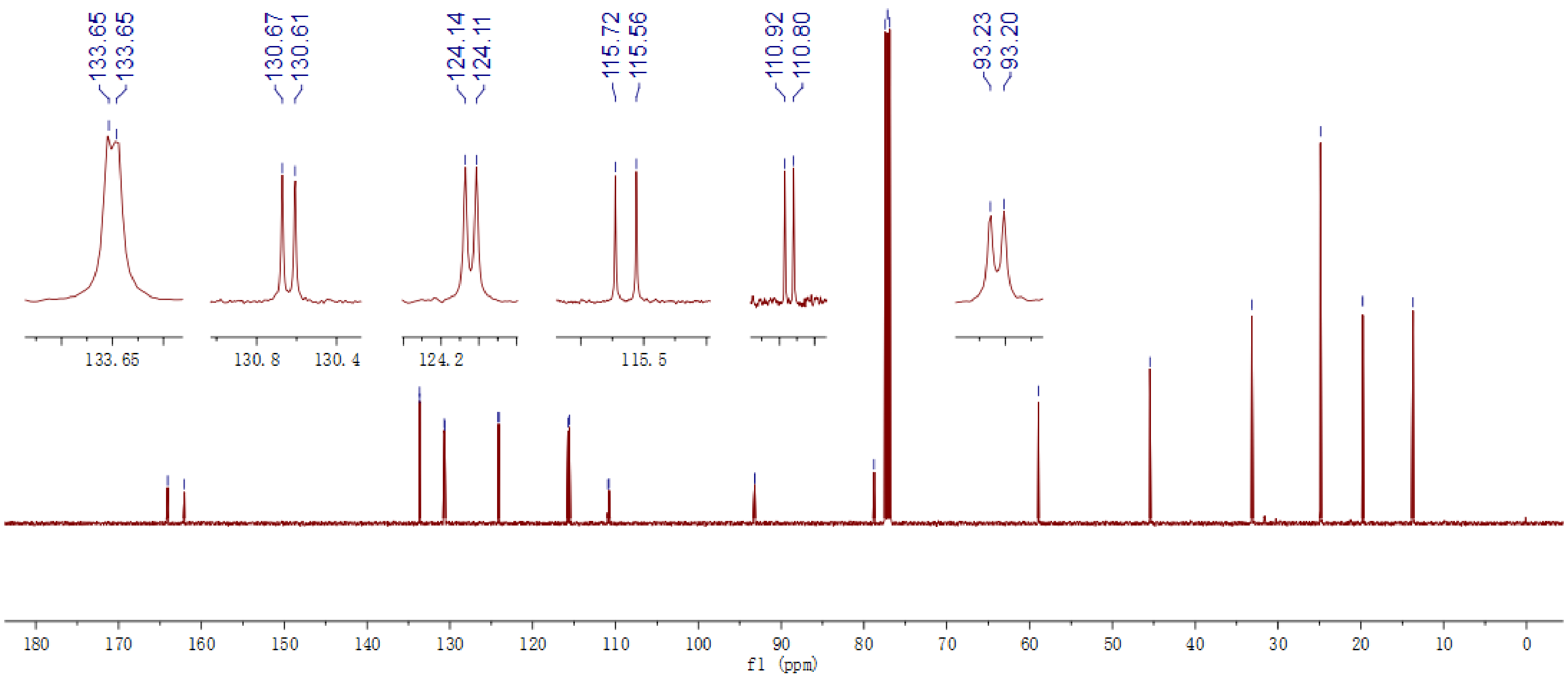

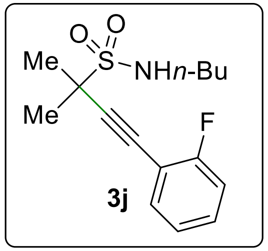

^{19}F NMR: 376 MHz in $CDCl_3$

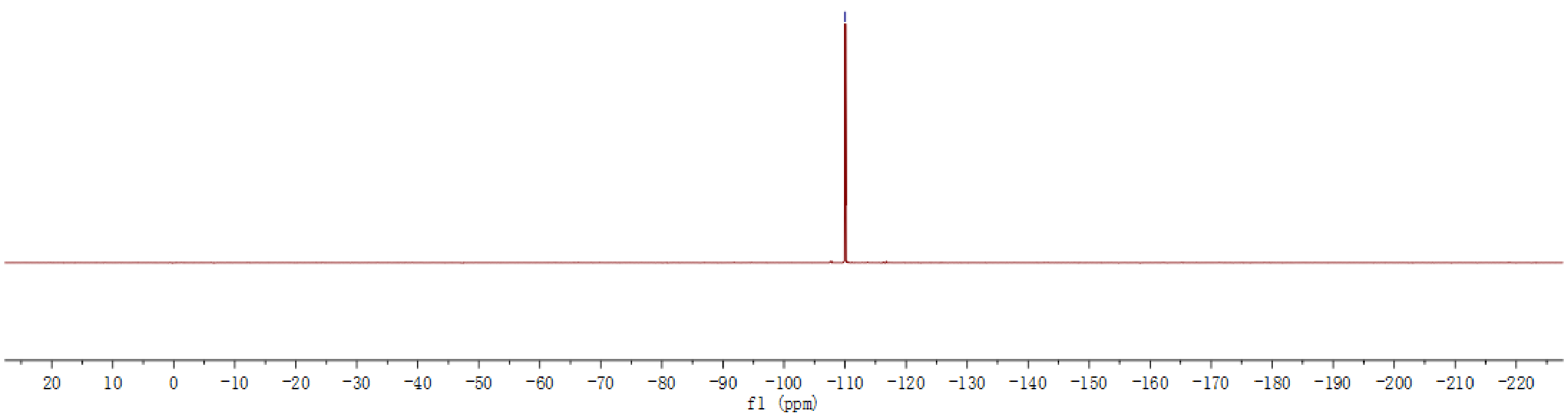

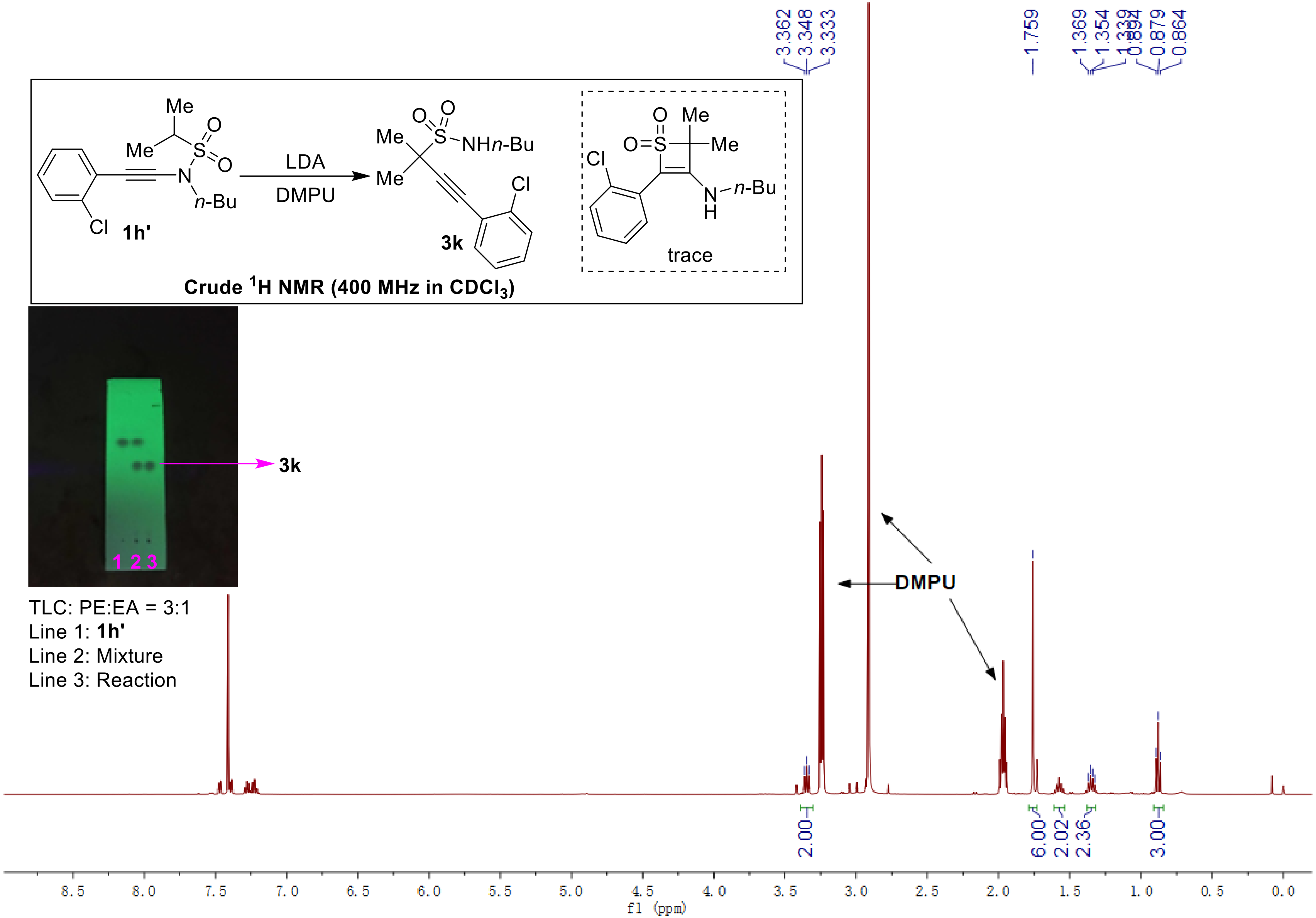
LDA
DMPU
1h'
3k
trace
Crude 1H NMR (400 MHz in CDCl3)
3k
1 2 3
TLC: PE:EA = 3:1
Line 1: 1h'
Line 2: Mixture
Line 3: Reaction
DMPU
3.362
3.348
3.333
1.759
1.369
1.354
1.339
0.894
0.879
0.864
2.00
6.00
2.02
2.36
3.00
f1 (ppm)

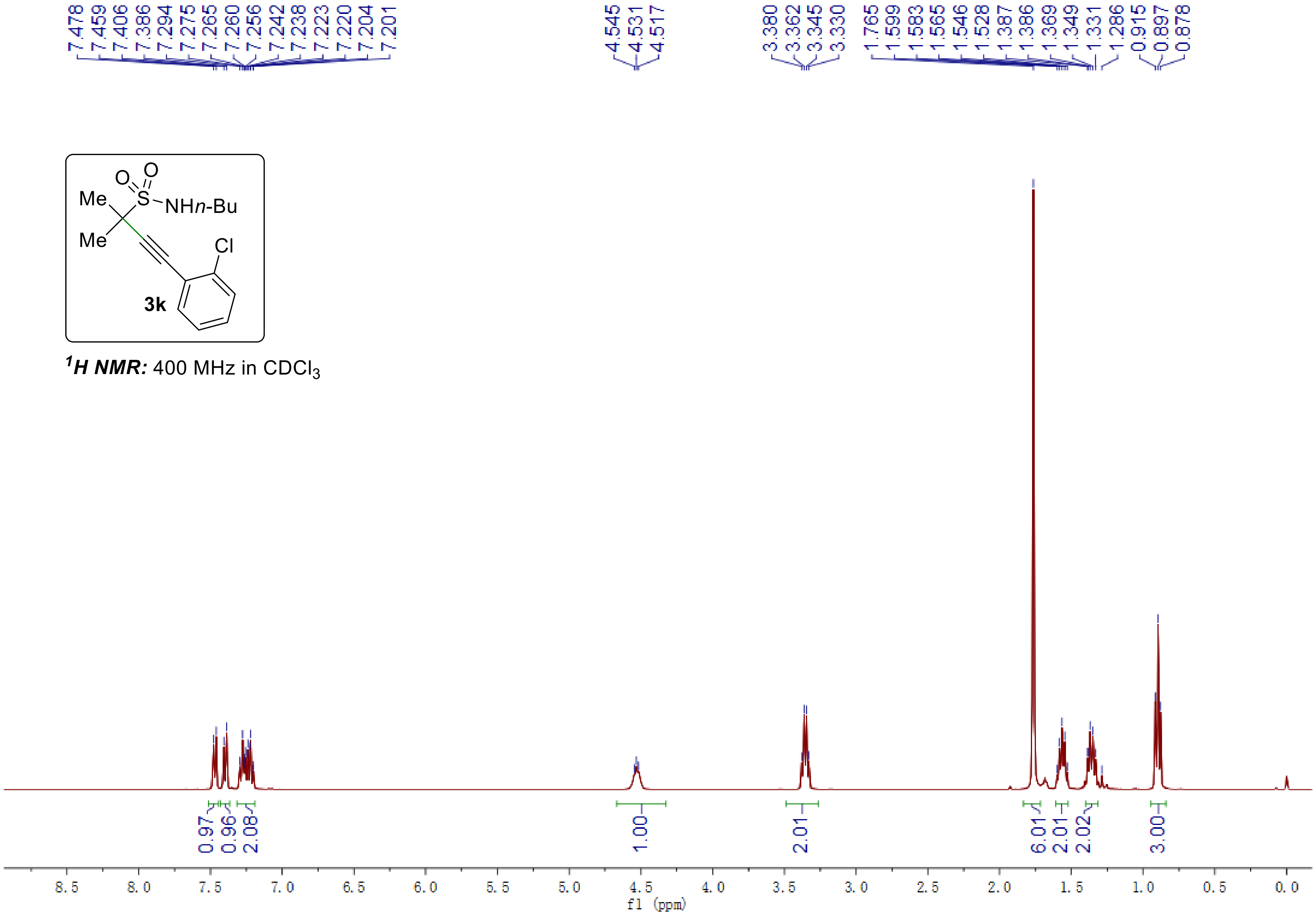

¹H NMR: 400 MHz in $CDCl_3$

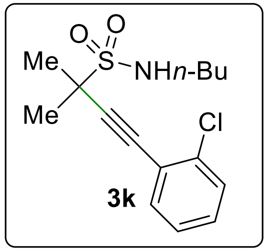

^{13}C NMR: 100 MHz in $CDCl_3$

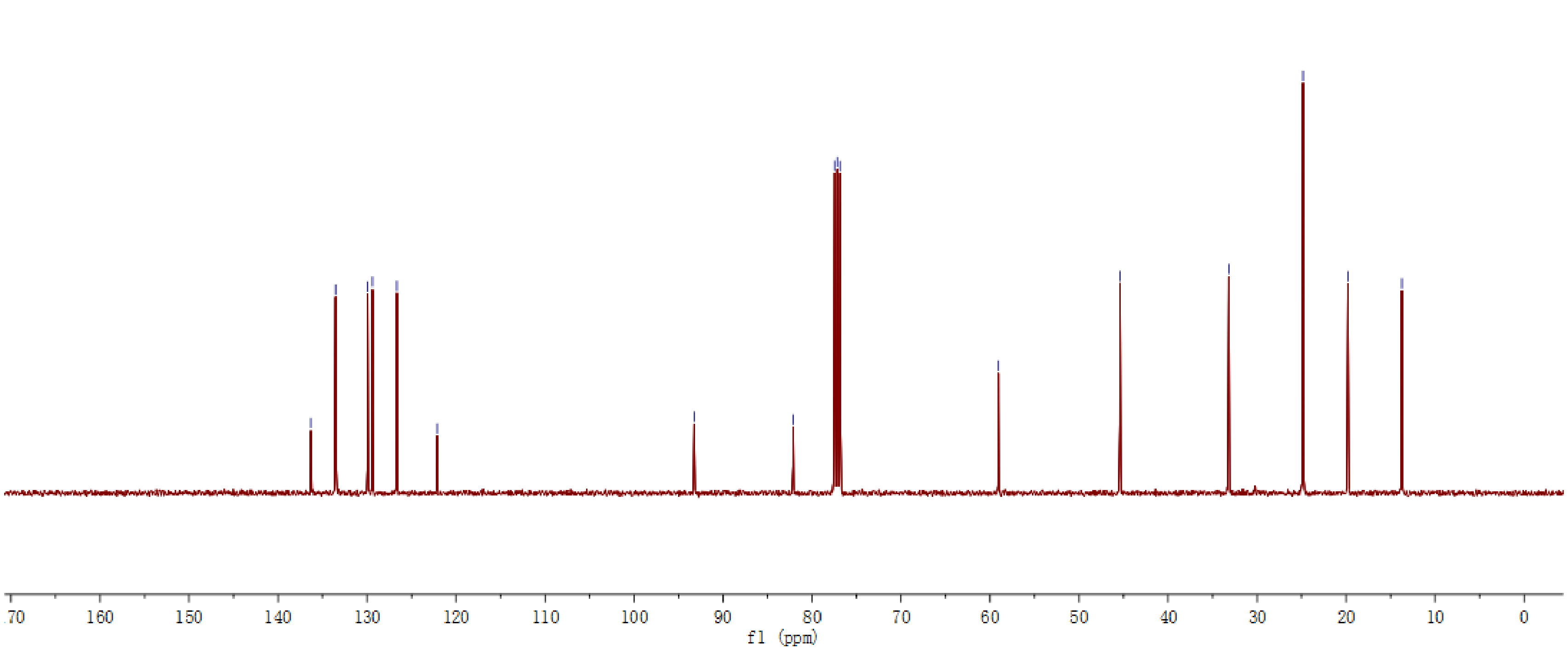

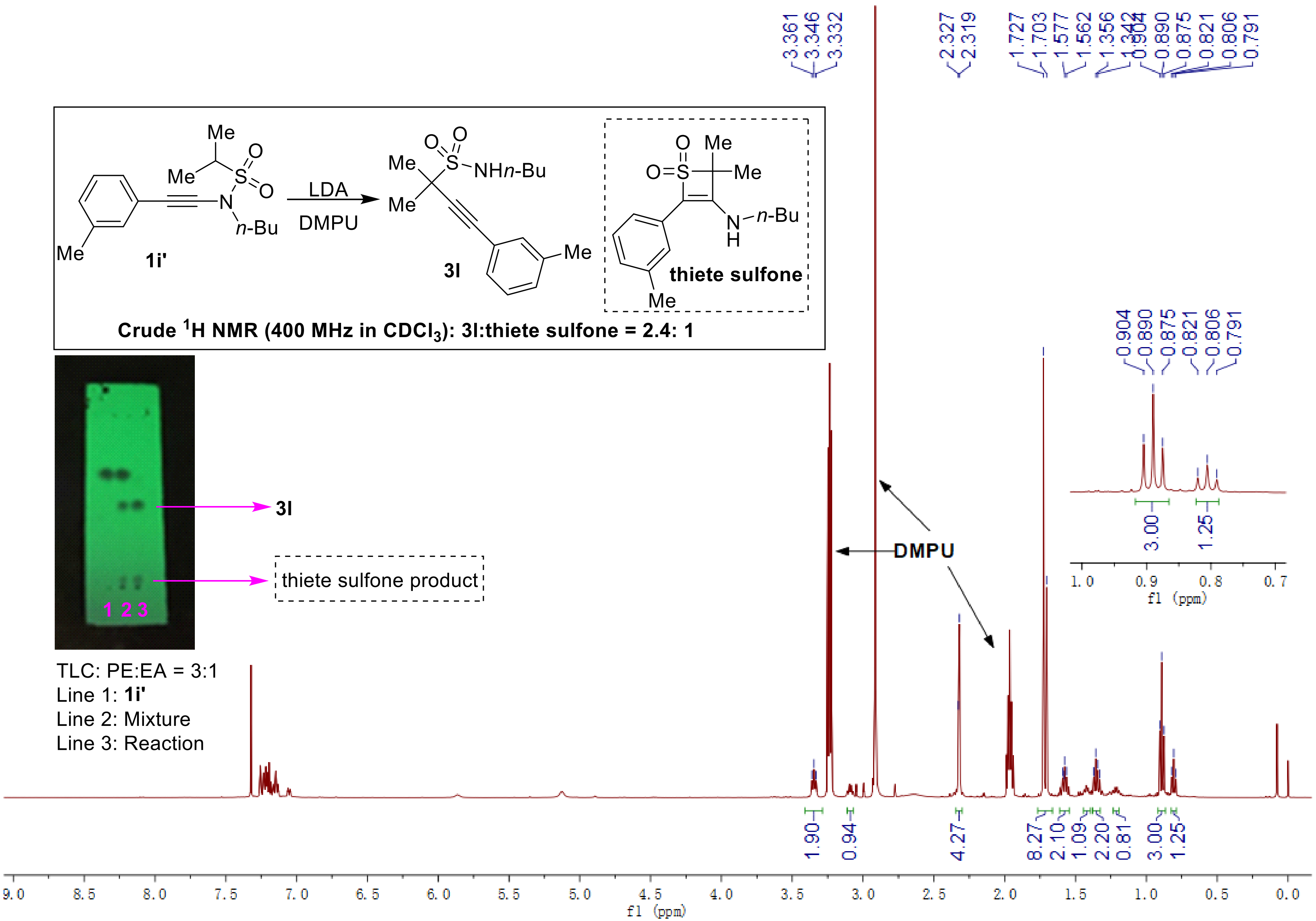

1i'
LDA
DMPU
3l
thiete sulfone
Crude ¹H NMR (400 MHz in CDCl3): 3l:thiete sulfone = 2.4: 1
3l
thiete sulfone product
TLC: PE:EA = 3:1
Line 1: 1i'
Line 2: Mixture
Line 3: Reaction
DMPU
f1 (ppm)

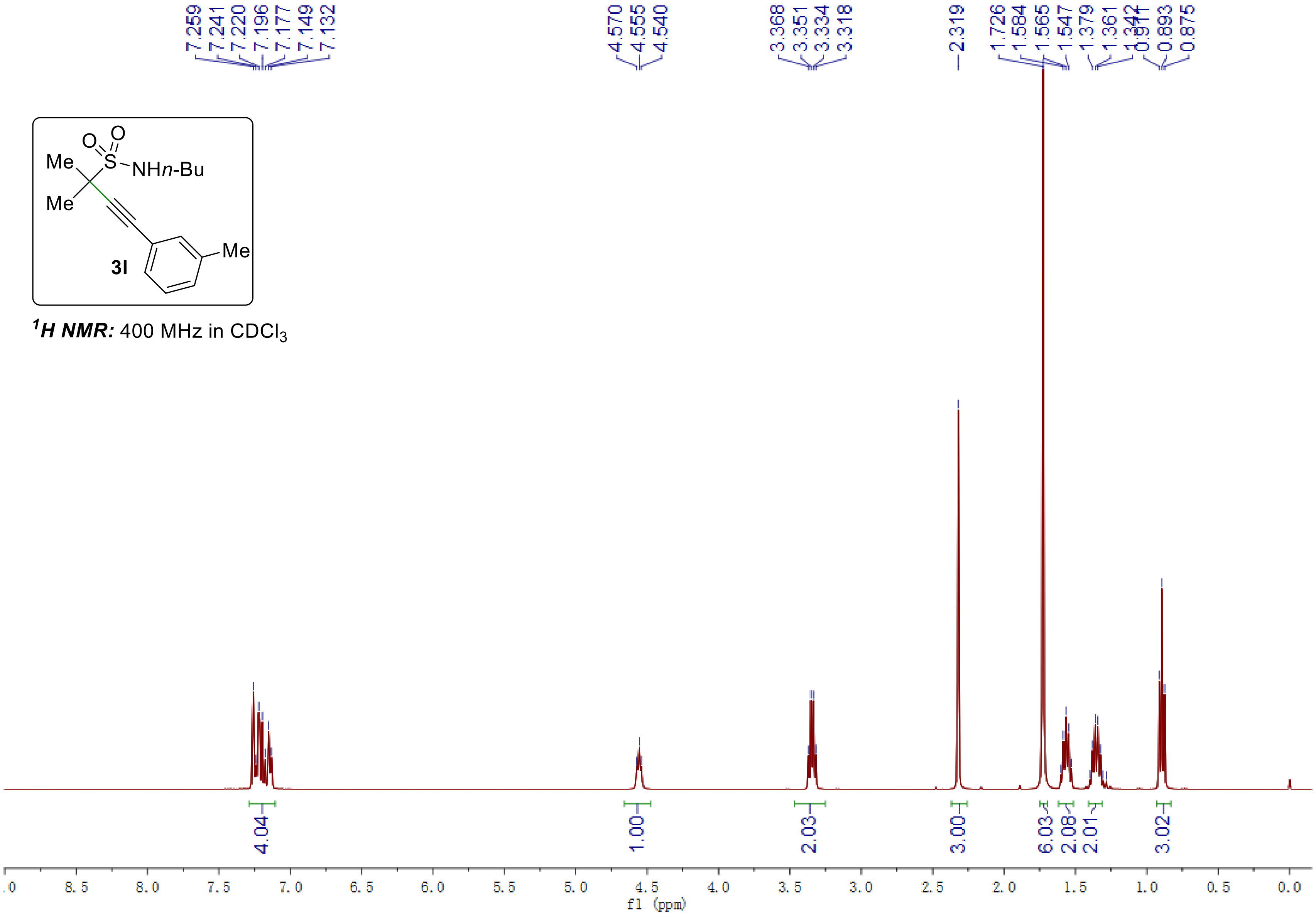
7.259
7.241
7.220
7.196
7.177
7.149
7.132
4.570
4.555
4.540
3.368
3.351
3.334
3.318
2.319
1.726
1.584
1.565
1.547
1.379
1.361
1.342
0.911
0.893
0.875
Me
Me
O
O
S
NH*n*-Bu
Me
3l
^{1}H NMR: 400 MHz in $CDCl_3$
4.04
1.00
2.03
3.00
6.03
2.08
2.01
3.02
f1 (ppm)

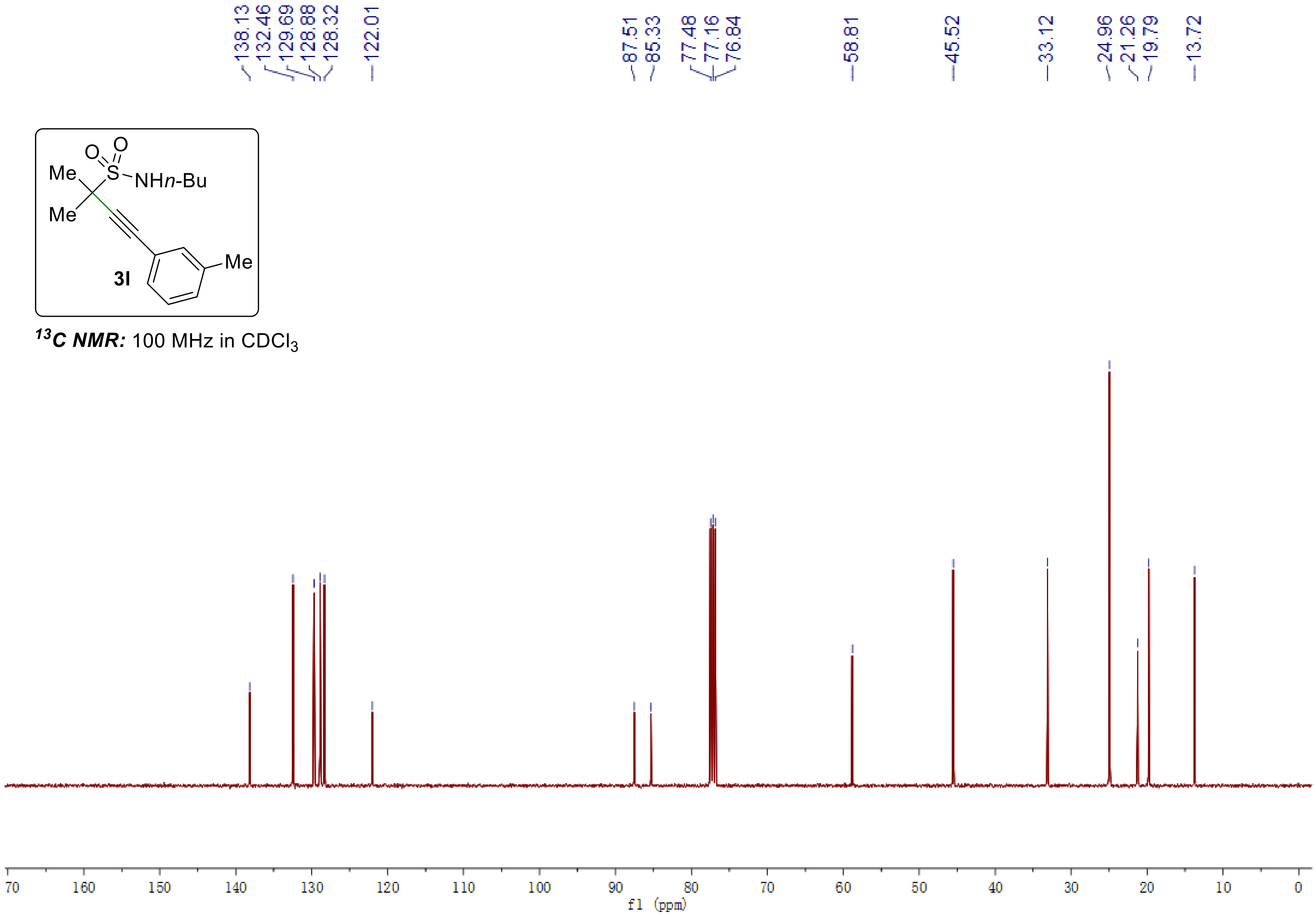
138.13
132.46
129.69
128.88
128.32
122.01
87.51
85.33
77.48
77.16
76.84
58.81
45.52
33.12
24.96
21.26
19.79
13.72
Me
Me
O
O
S
NH*n*-Bu
Me
3l
13C NMR: 100 MHz in CDCl3
170
160
150
140
130
120
110
100
90
80
70
60
50
40
30
20
10
0
f1 (ppm)

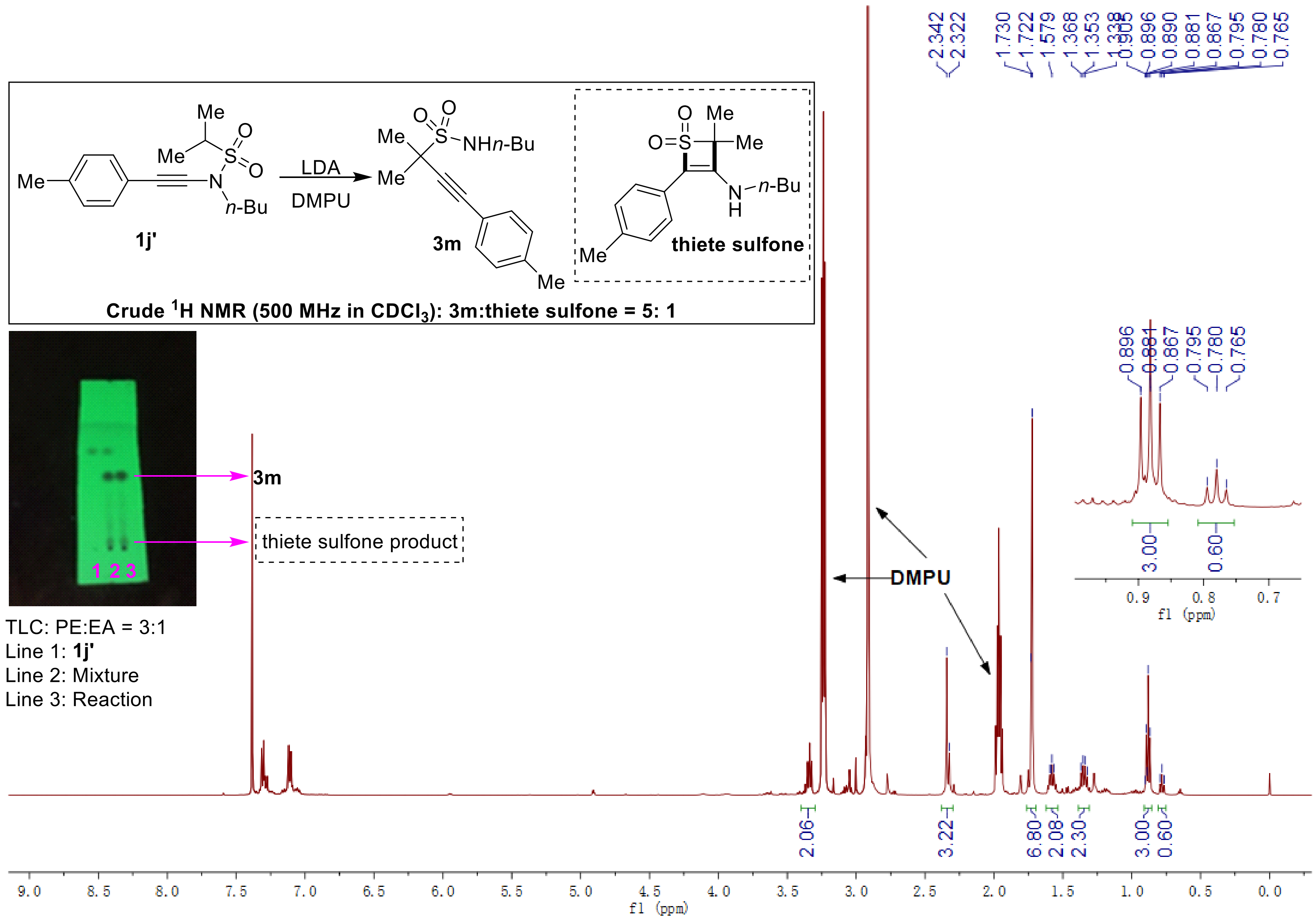
Me
Me
Me
n-Bu
1j'
LDA
DMPU
Me
Me
NHn-Bu
3m
Me
O=S
Me
Me
N
H
n-Bu
Me
thiete sulfone
Crude 1H NMR (500 MHz in CDCl3): 3m:thiete sulfone = 5: 1
1 2 3
3m
thiete sulfone product
DMPU
TLC: PE:EA = 3:1
Line 1: 1j'
Line 2: Mixture
Line 3: Reaction
2.342
2.322
1.730
1.722
1.579
1.368
1.353
0.896
0.890
0.881
0.867
0.795
0.780
0.765
2.06
3.22
6.80
2.08
2.30
3.00
0.60
f1 (ppm)

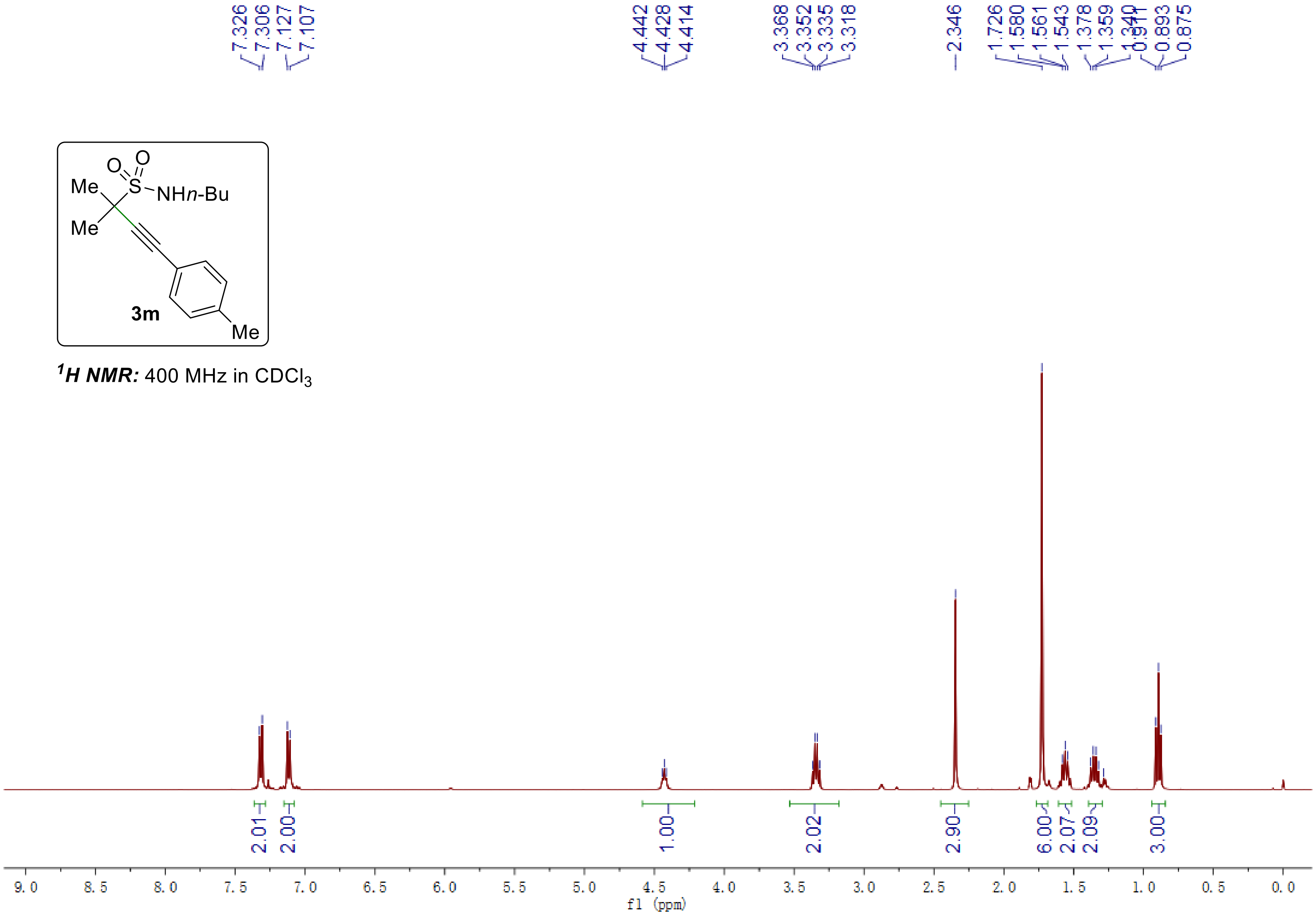

^{1}H NMR: 400 MHz in $CDCl_3$

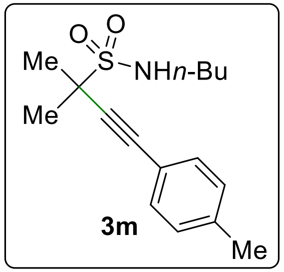

^{13}**_C NMR:_** 100 MHz in $CDCl_3$

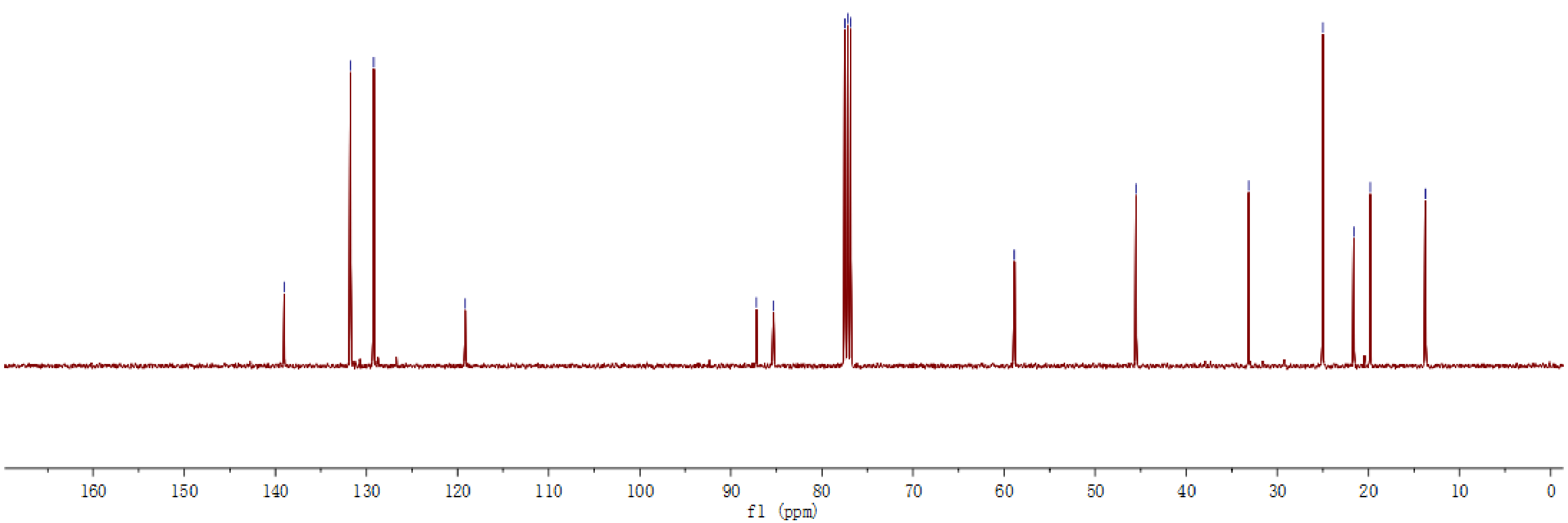

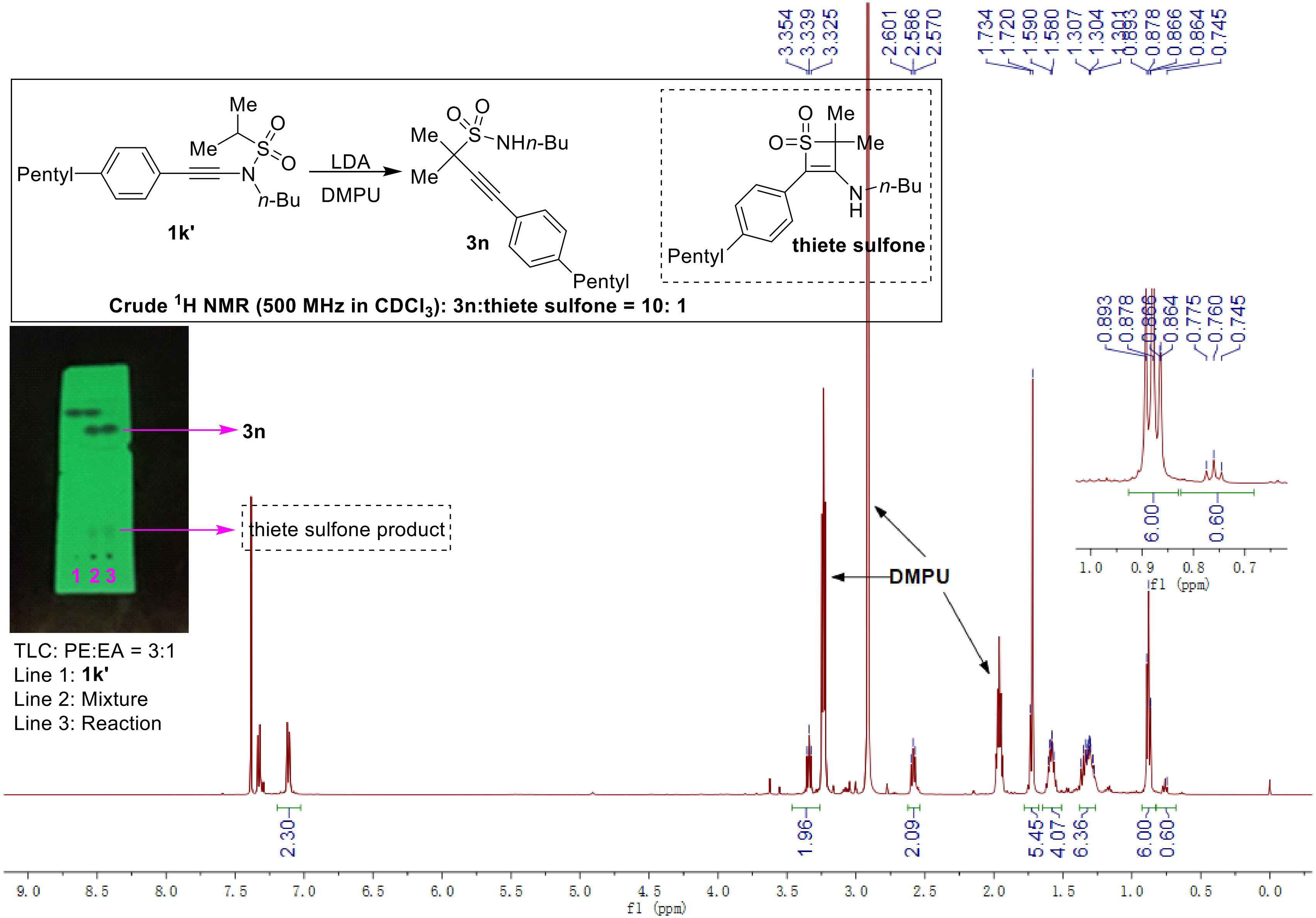
Pentyl
Me
Me
n-Bu
1k'
LDA
DMPU
Me
Me
NHn-Bu
3n
Pentyl
thiete sulfone
Crude 1H NMR (500 MHz in CDCl3): 3n:thiete sulfone = 10: 1
3n
thiete sulfone product
1 2 3
TLC: PE:EA = 3:1
Line 1: 1k'
Line 2: Mixture
Line 3: Reaction
DMPU
3.354
3.339
3.325
2.601
2.586
2.570
1.734
1.720
1.590
1.580
1.307
1.304
1.301
0.893
0.878
0.866
0.864
0.745
0.775
0.760
2.30
1.96
2.09
5.45
4.07
6.36
6.00
0.60
f1 (ppm)

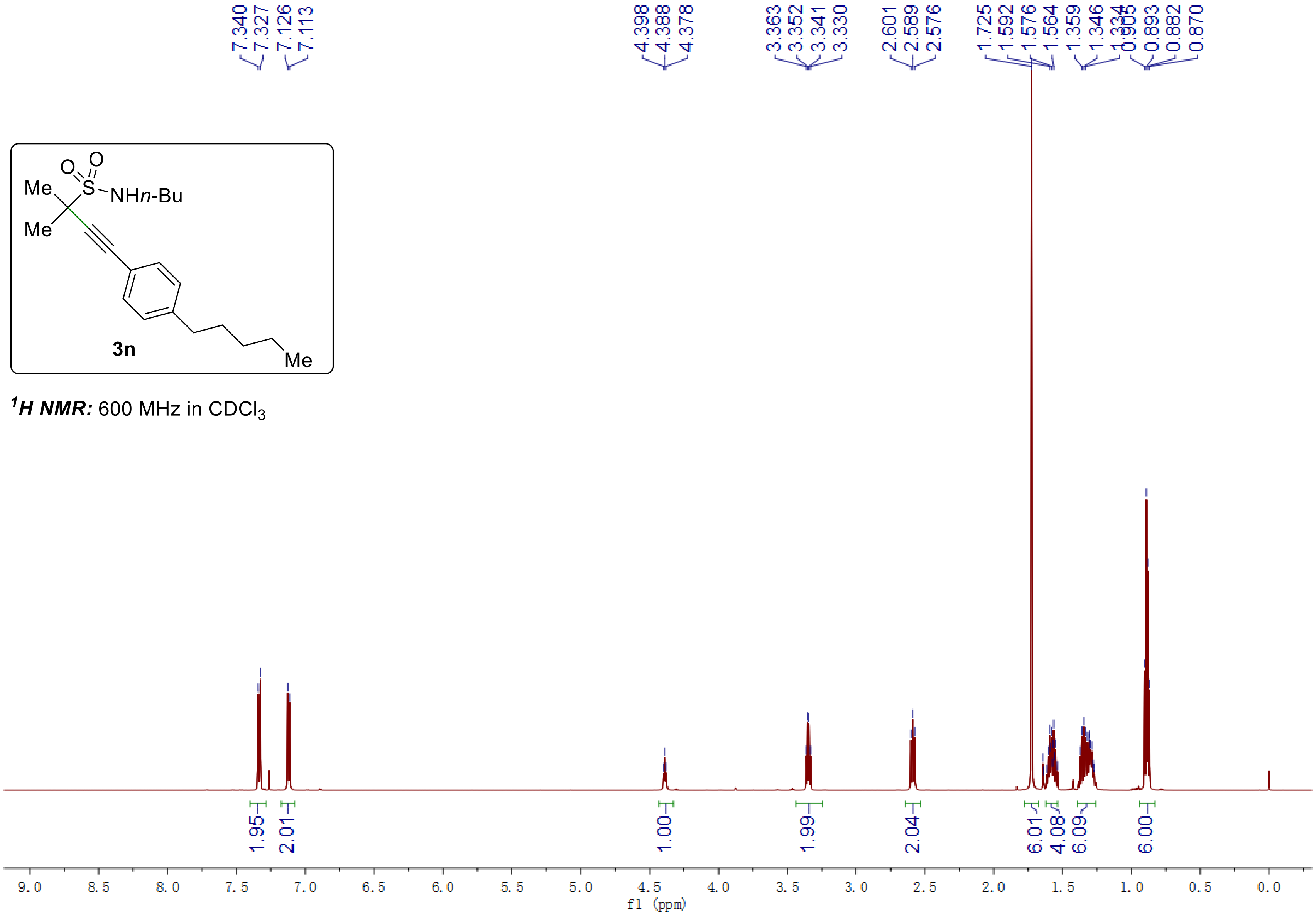

[1]*H NMR:* 600 MHz in $CDCl_3$

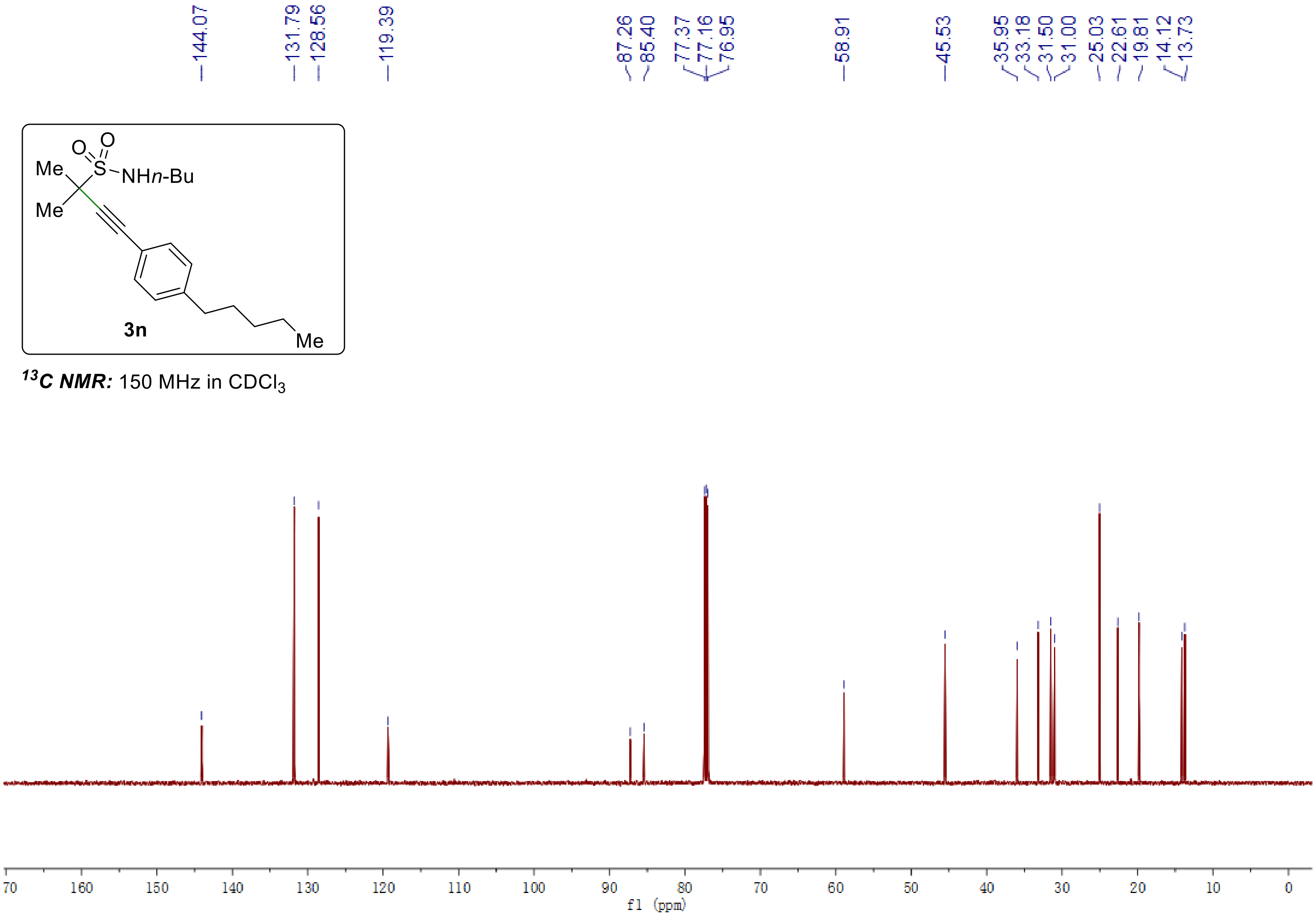
144.07
131.79
128.56
119.39
87.26
85.40
77.37
77.16
76.95
58.91
45.53
35.95
33.18
31.50
31.00
25.03
22.61
19.81
14.12
13.73
O
O
Me
S
NHn-Bu
Me
3n
Me
13C NMR: 150 MHz in CDCl3
170
160
150
140
130
120
110
100
90
80
70
60
50
40
30
20
10
0
f1 (ppm)

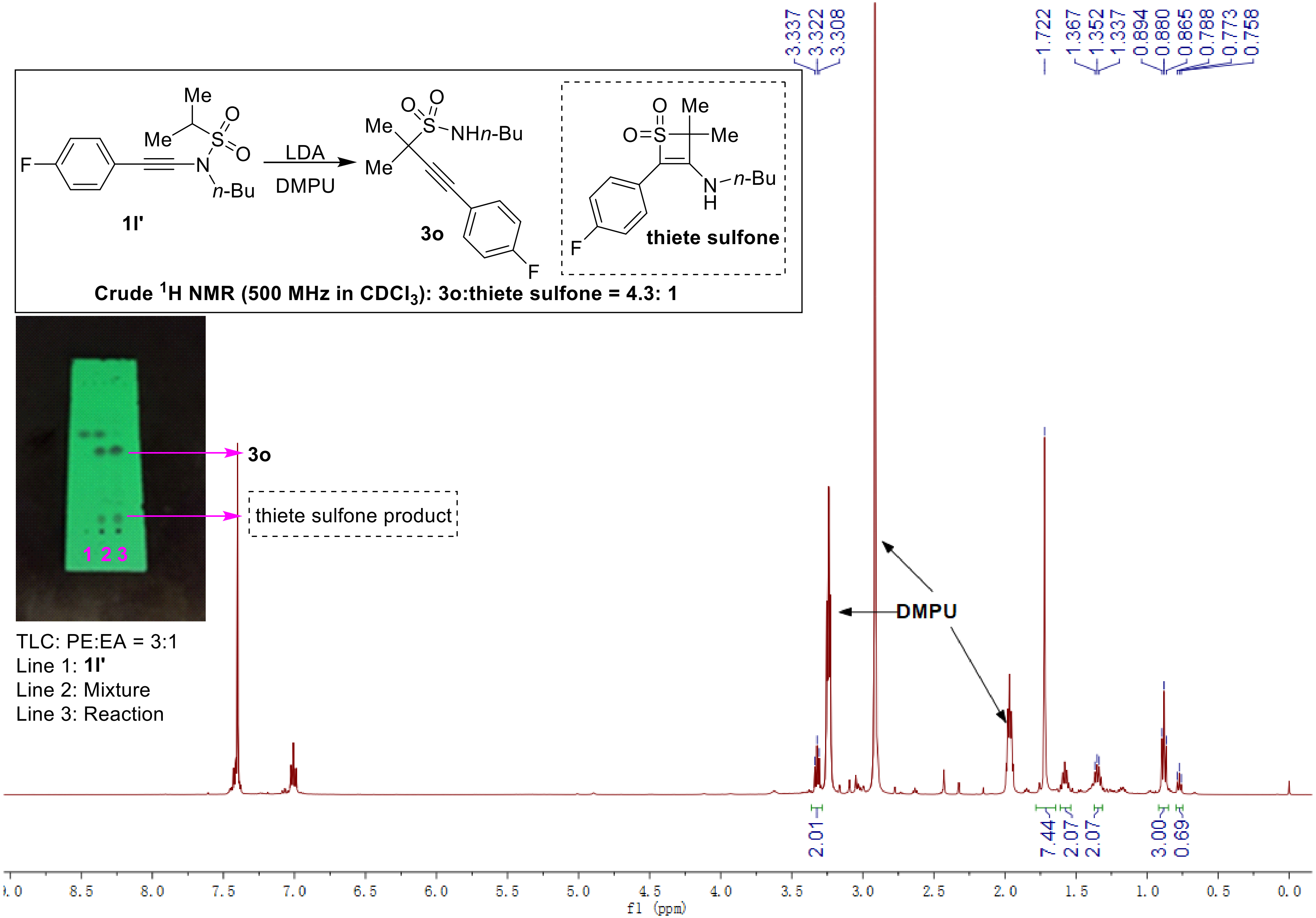
Me
Me
F
N
n-Bu
1l'
LDA
DMPU
Me
Me
NHn-Bu
3o
F
O
O
Me
Me
N
n-Bu
H
F
thiete sulfone
Crude 1H NMR (500 MHz in CDCl3): 3o:thiete sulfone = 4.3: 1
1 2 3
3o
thiete sulfone product
TLC: PE:EA = 3:1
Line 1: 1l'
Line 2: Mixture
Line 3: Reaction
DMPU
3.337
3.322
3.308
1.722
1.367
1.352
1.337
0.894
0.880
0.865
0.788
0.773
0.758
2.01
7.44
2.07
2.07
3.00
0.69
f1 (ppm)

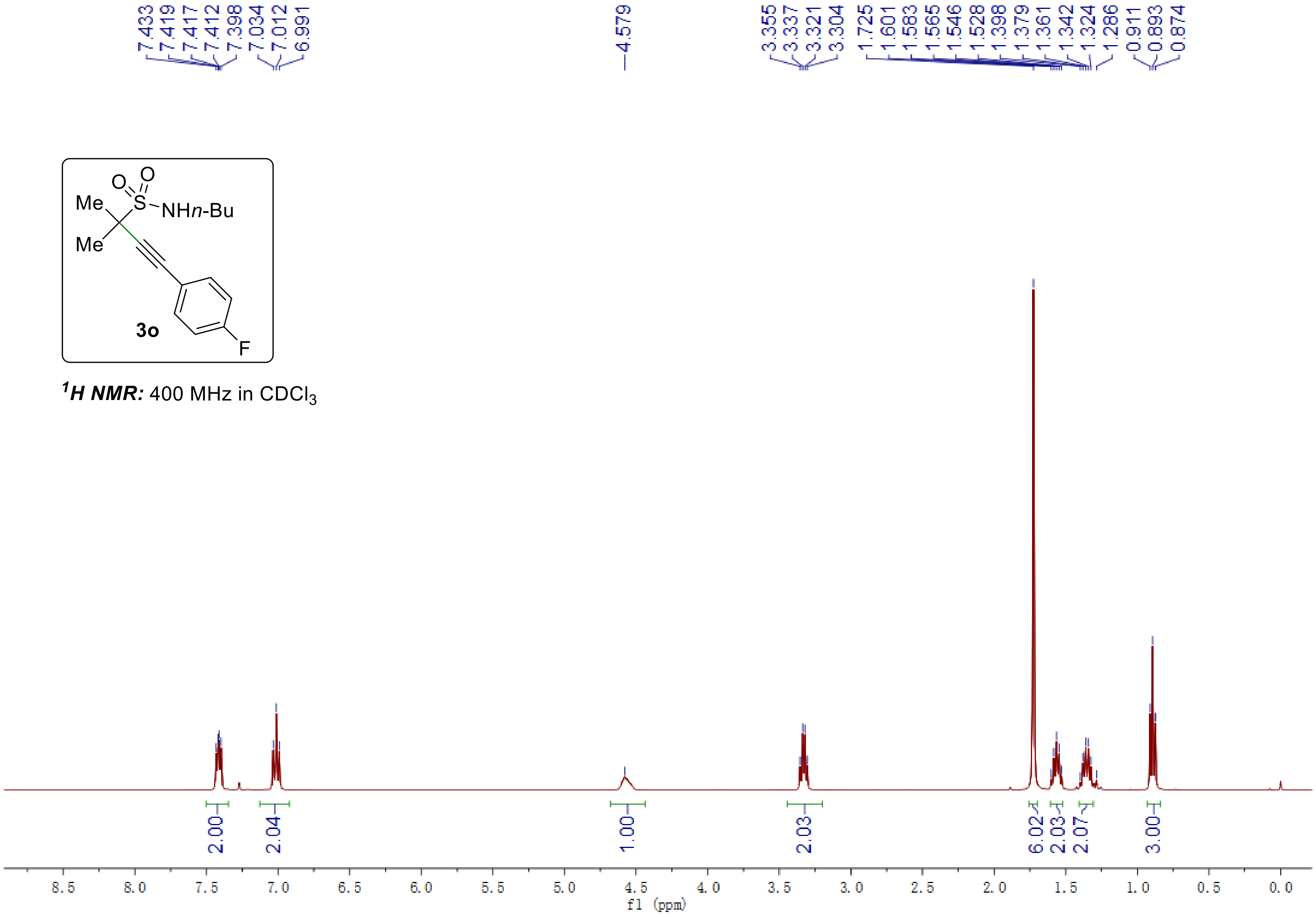
7.433
7.419
7.417
7.412
7.398
7.034
7.012
6.991
4.579
3.355
3.337
3.321
3.304
1.725
1.601
1.583
1.565
1.546
1.528
1.398
1.379
1.361
1.342
1.324
1.286
0.911
0.893
0.874
O
O
Me
S
NH*n*-Bu
Me
3o
F
¹H NMR: 400 MHz in CDCl3
2.00
2.04
1.00
2.03
6.02
2.03
2.07
3.00
8.5
8.0
7.5
7.0
6.5
6.0
5.5
5.0
4.5
4.0
3.5
3.0
2.5
2.0
1.5
1.0
0.5
0.0
f1 (ppm)

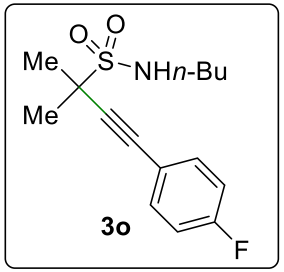

^{13}C NMR: 100 MHz in $CDCl_3$

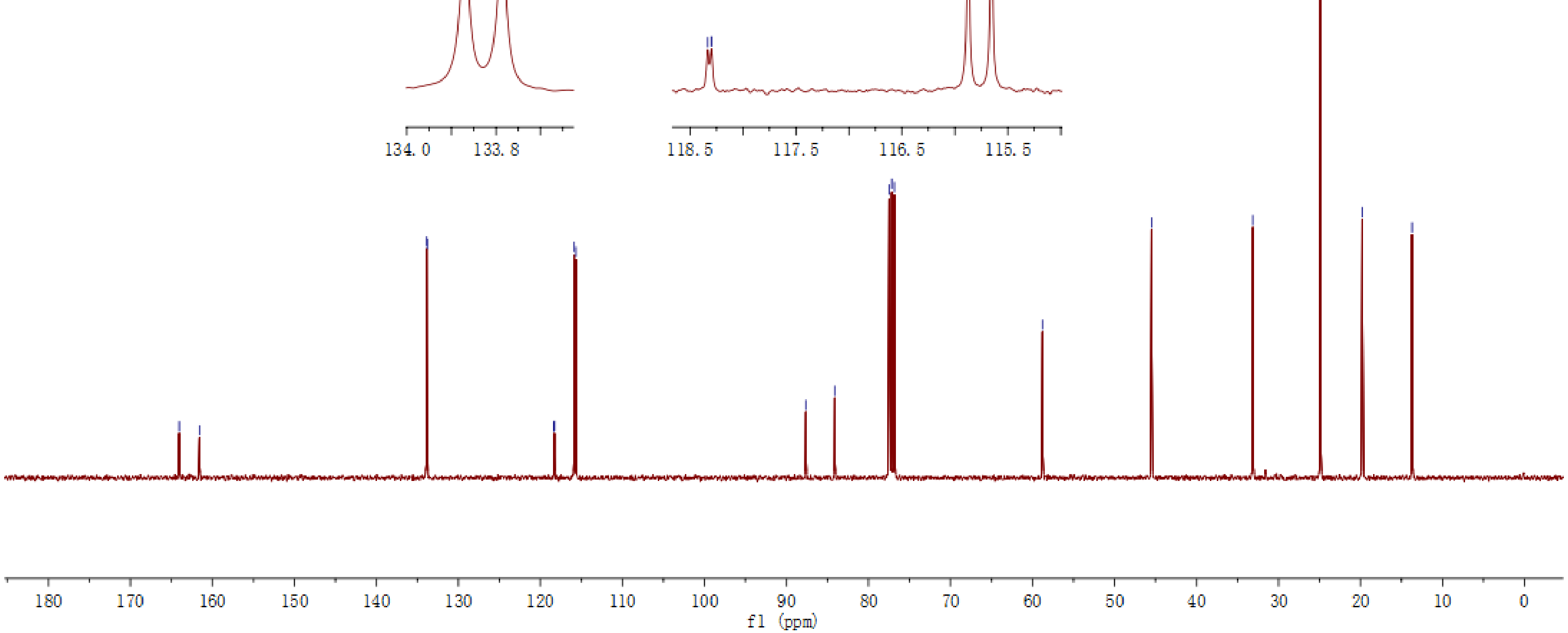

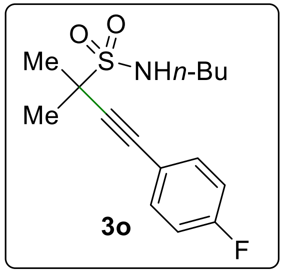

^{19}F NMR: 376 MHz in $CDCl_3$

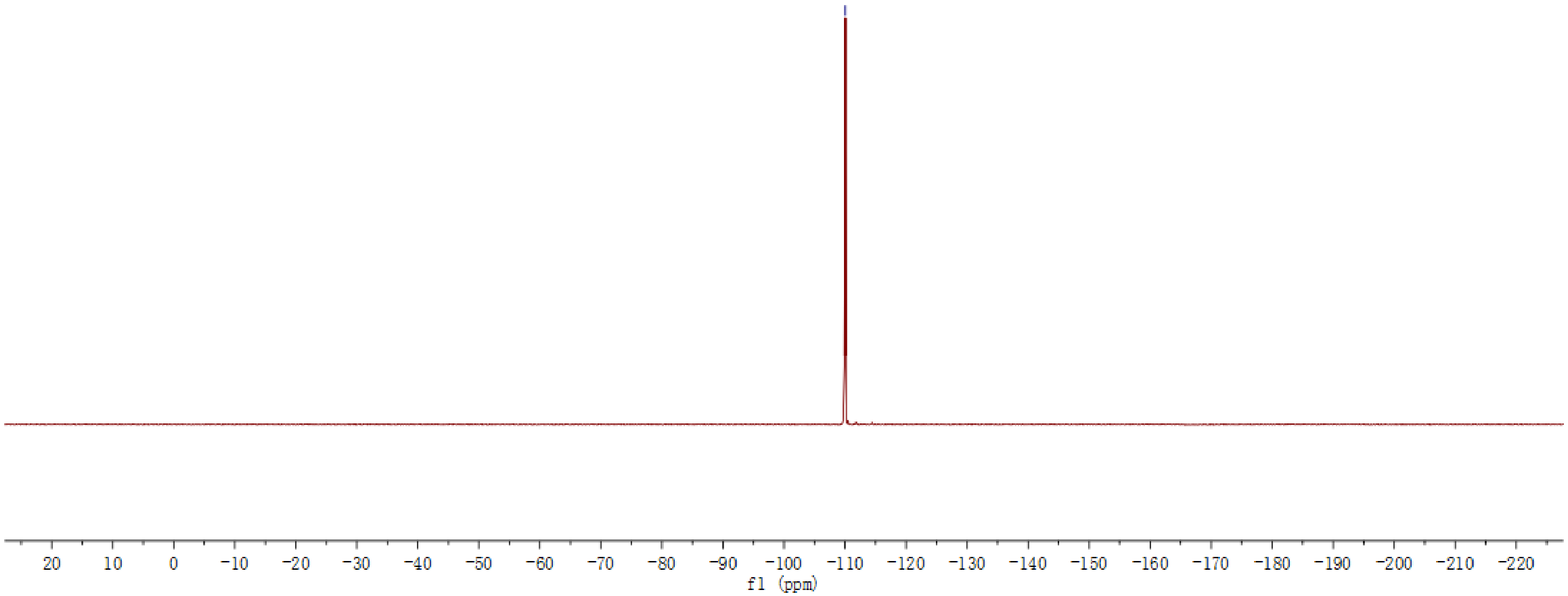

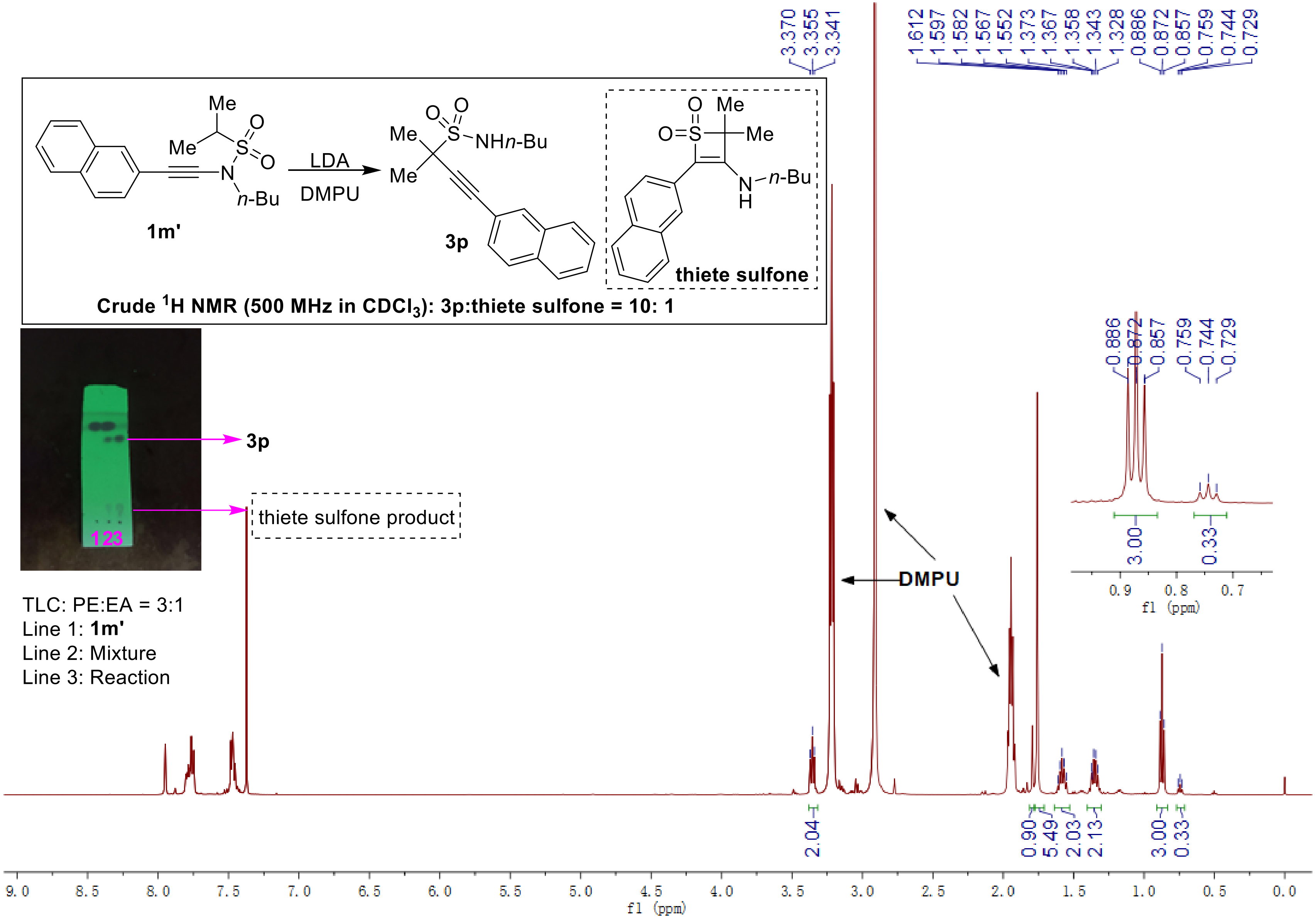
1m'
LDA
DMPU
3p
thiete sulfone
Crude ¹H NMR (500 MHz in CDCl3): 3p:thiete sulfone = 10: 1
3p
thiete sulfone product
TLC: PE:EA = 3:1
Line 1: 1m'
Line 2: Mixture
Line 3: Reaction
DMPU
3.370
3.355
3.341
1.612
1.597
1.582
1.567
1.552
1.373
1.367
1.358
1.343
1.328
0.886
0.872
0.857
0.759
0.744
0.729
2.04
0.90
5.49
2.03
2.13
3.00
0.33
f1 (ppm)

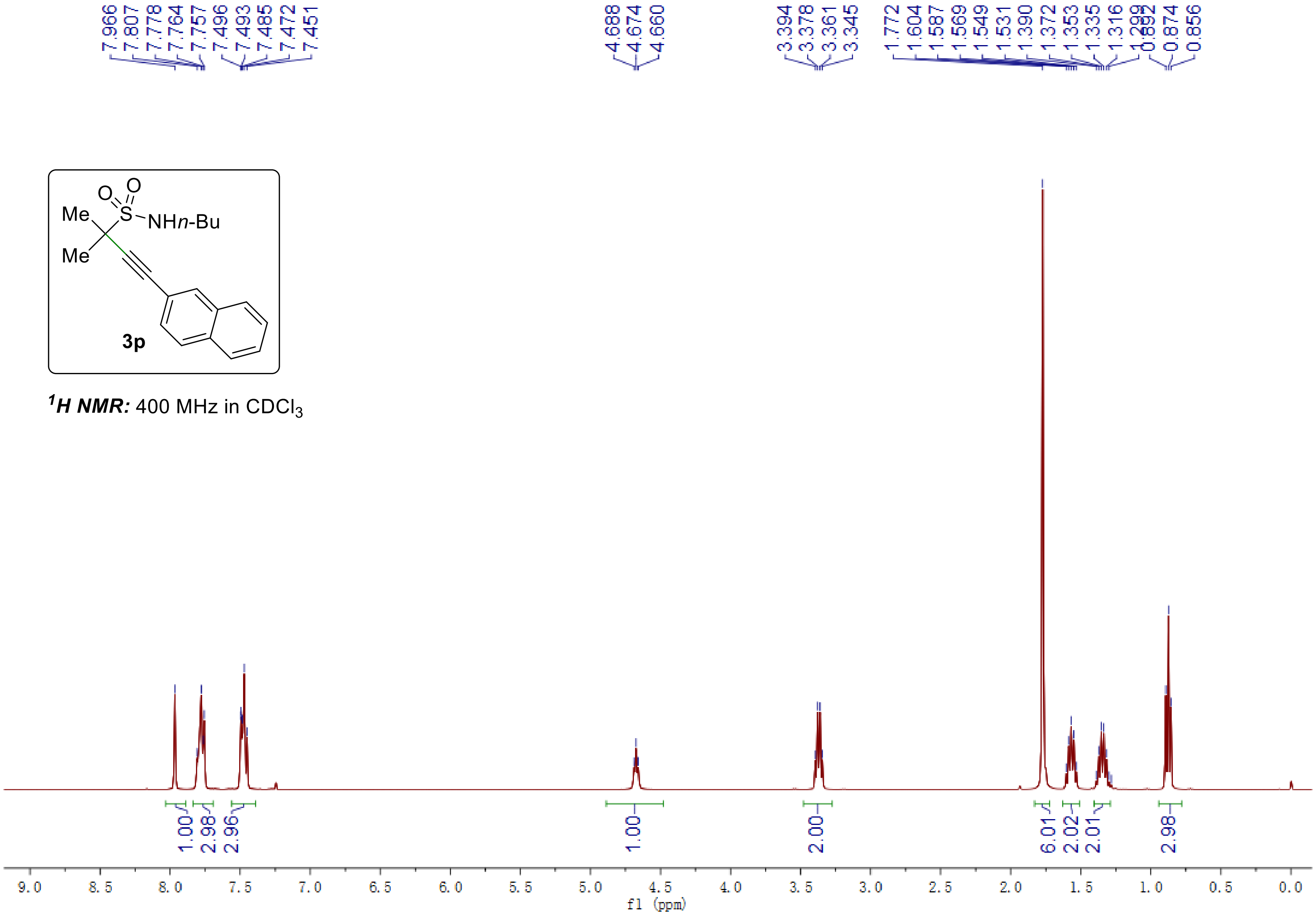

^{1}H NMR: 400 MHz in $CDCl_3$

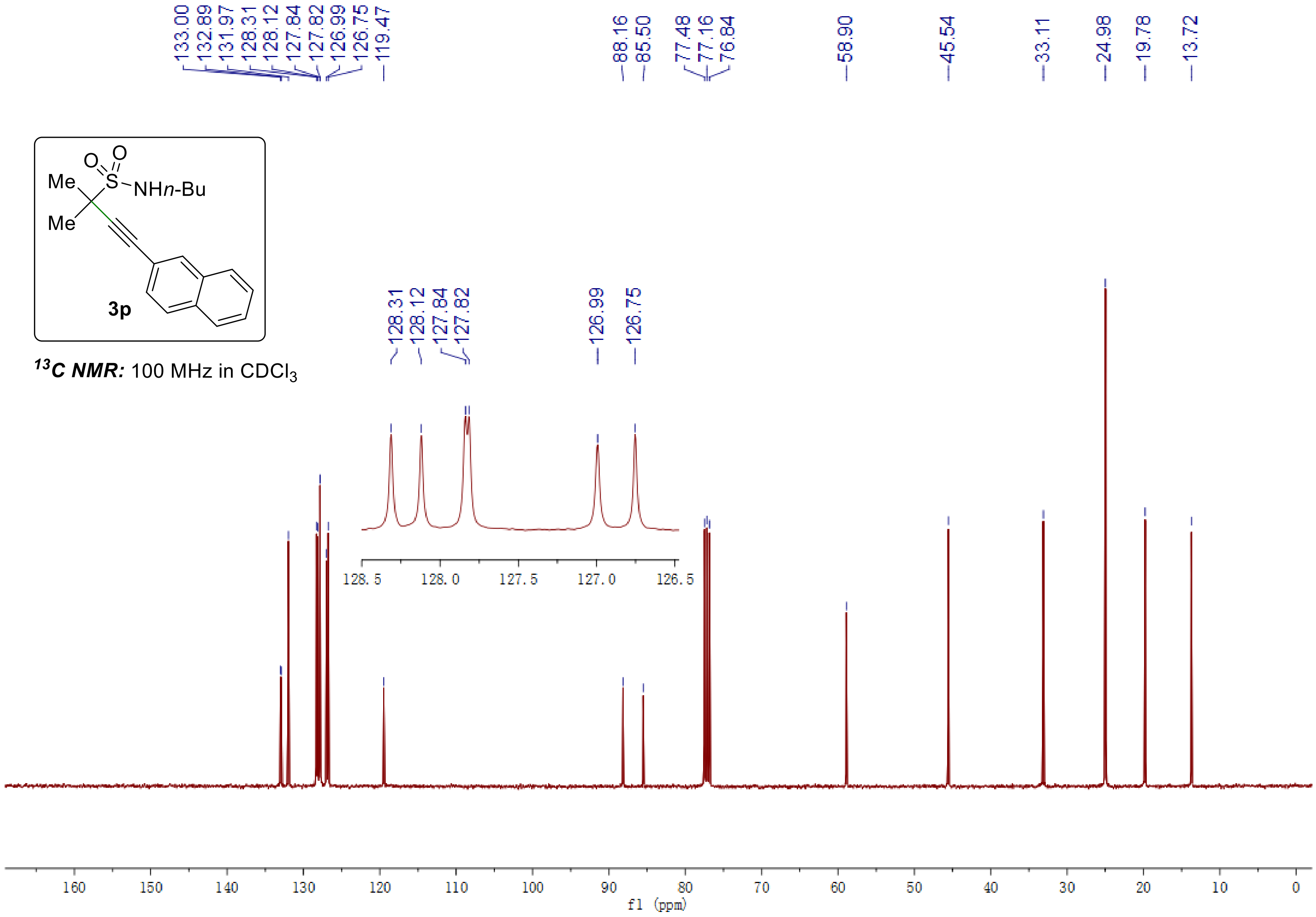
133.00
132.89
131.97
128.31
128.12
127.84
127.82
126.99
126.75
119.47
88.16
85.50
77.48
77.16
76.84
58.90
45.54
33.11
24.98
19.78
13.72
Me
Me
O
O
S
NH*n*-Bu
3p
^{13}C NMR: 100 MHz in CDCl$_3$
f1 (ppm)

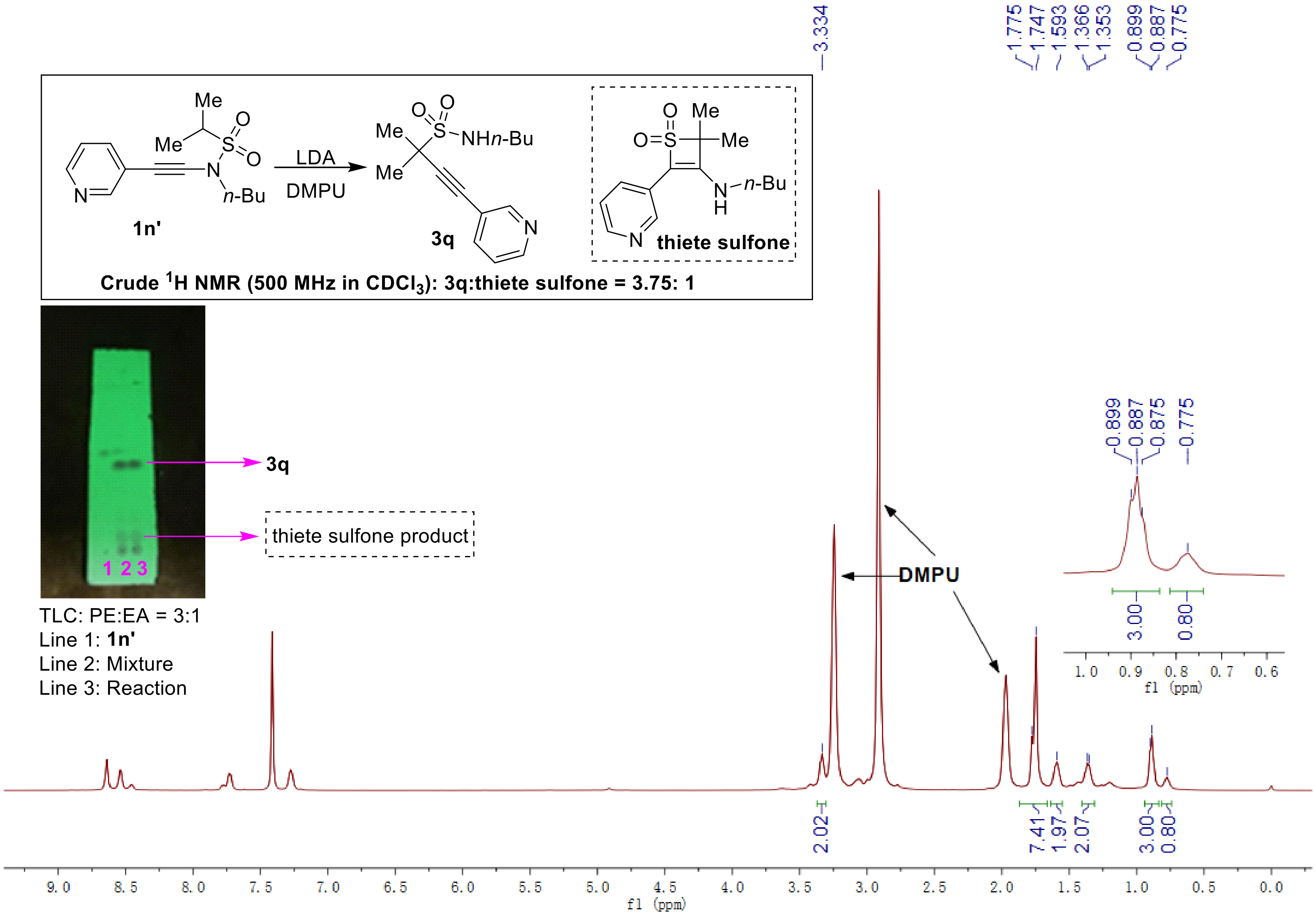

1n'
LDA
DMPU
3q
thiete sulfone
Crude ¹H NMR (500 MHz in CDCl3): 3q:thiete sulfone = 3.75: 1
3q
thiete sulfone product
1 2 3
TLC: PE:EA = 3:1
Line 1: 1n'
Line 2: Mixture
Line 3: Reaction
DMPU
3.334
1.775
1.747
1.593
1.366
1.353
0.899
0.887
0.775
0.875
2.02
7.41
1.97
2.07
3.00
0.80
f1 (ppm)

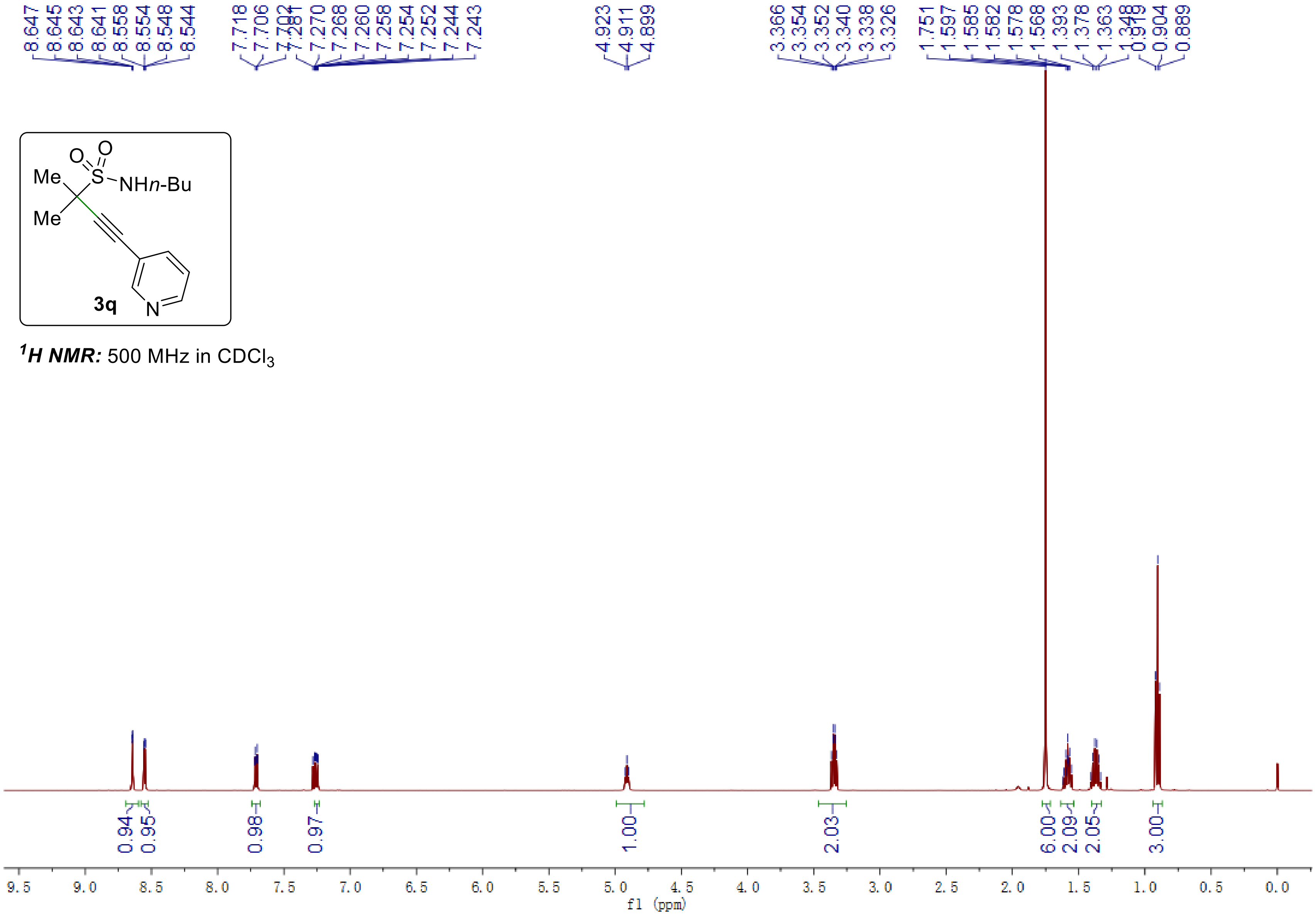

^{1}H NMR: 500 MHz in $CDCl_3$

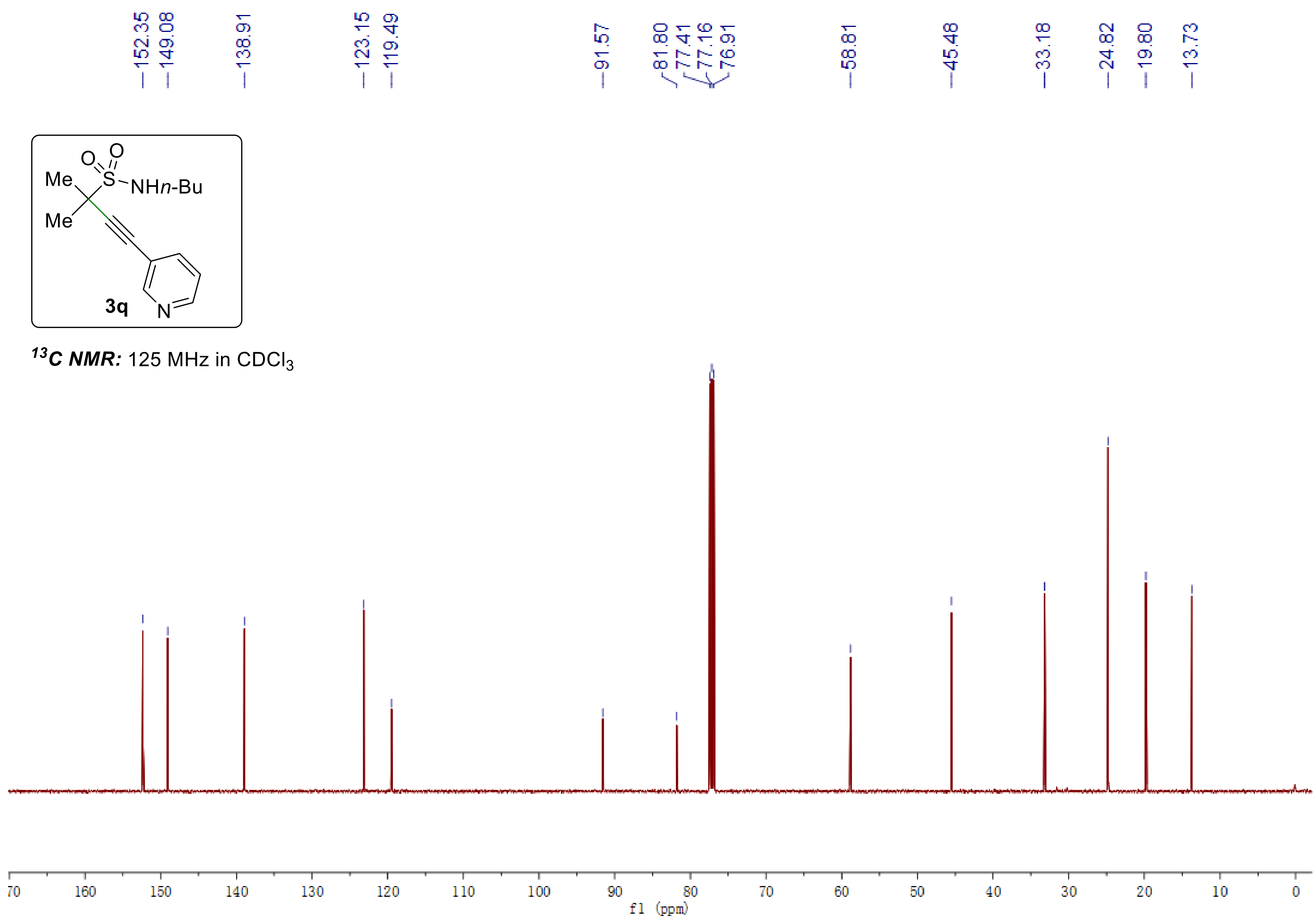

152.35
149.08
138.91
123.15
119.49
91.57
81.80
77.41
77.16
76.91
58.81
45.48
33.18
24.82
19.80
13.73
Me
Me
O
O
S
NHn-Bu
N
3q
13C NMR: 125 MHz in CDCl3
170
160
150
140
130
120
110
100
90
80
70
60
50
40
30
20
10
0
f1 (ppm)

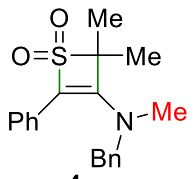

4a

¹H NMR: 500 MHz in $CDCl_3$

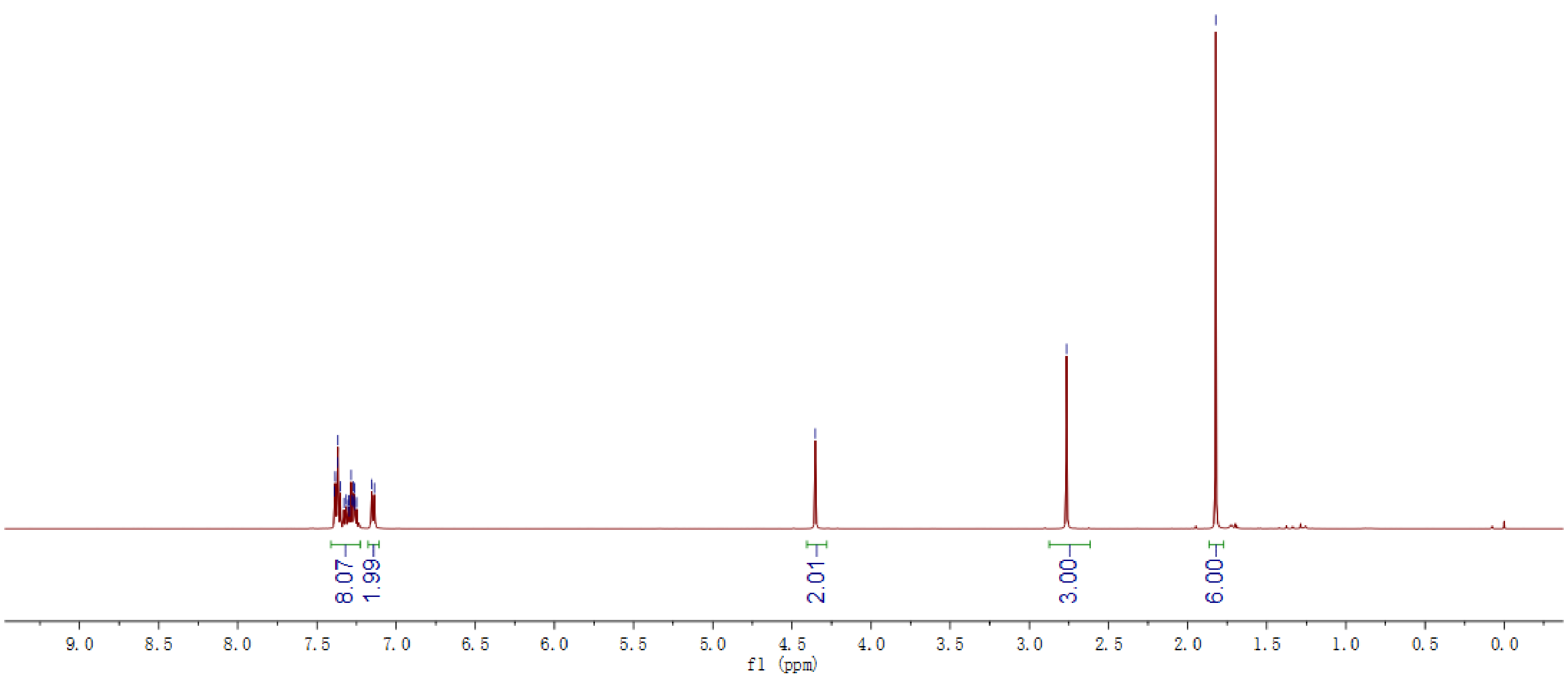

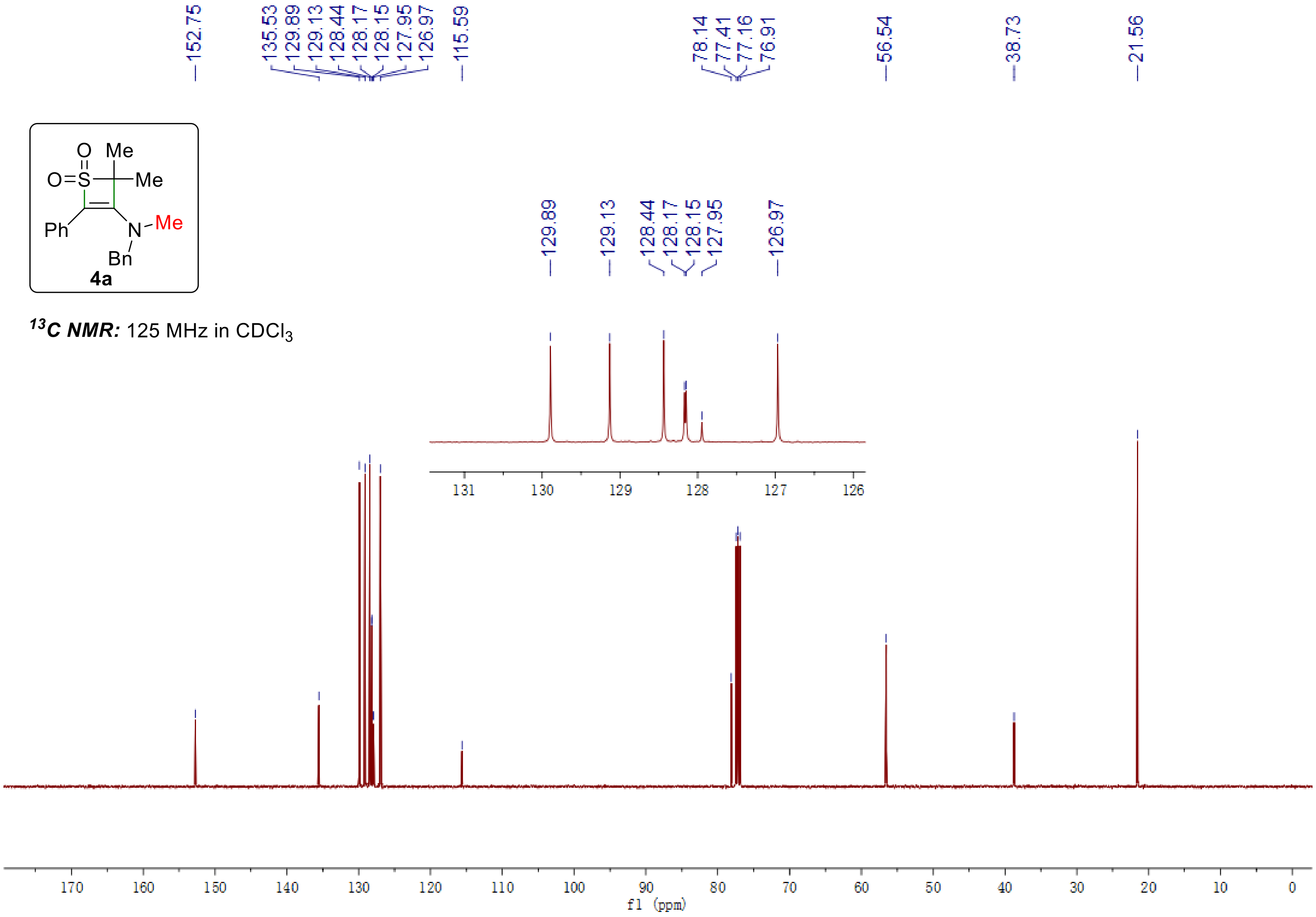
152.75
135.53
129.89
129.13
128.44
128.17
128.15
127.95
126.97
115.59
78.14
77.41
77.16
76.91
56.54
38.73
21.56
O
Me
O=S
Me
Ph
N
Me
Bn
4a
13C NMR: 125 MHz in CDCl3
f1 (ppm)

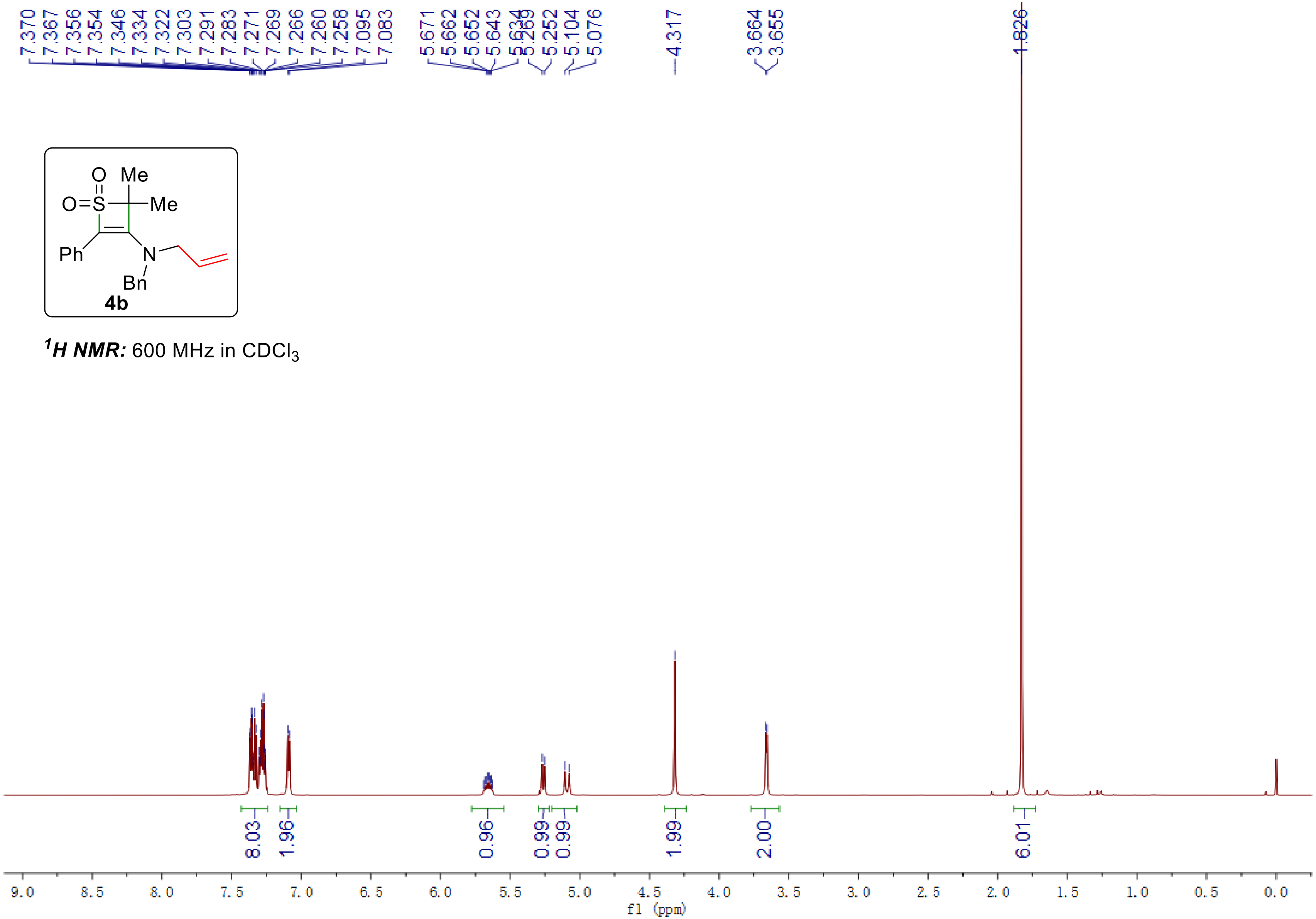
7.370
7.367
7.356
7.354
7.346
7.334
7.322
7.303
7.291
7.283
7.271
7.269
7.266
7.260
7.258
7.095
7.083
5.671
5.662
5.652
5.643
5.252
5.104
5.076
4.317
3.664
3.655
1.826
O
Me
O=S
Me
Ph
N
Bn
4b
^{1}H NMR: 600 MHz in CDCl$_3$
8.03
1.96
0.96
0.99
0.99
1.99
2.00
6.01
9.0
8.5
8.0
7.5
7.0
6.5
6.0
5.5
5.0
4.5
4.0
3.5
3.0
2.5
2.0
1.5
1.0
0.5
0.0
f1 (ppm)

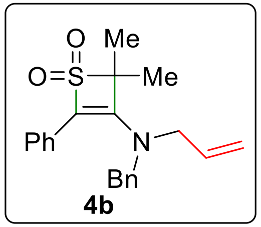

^{13}***C NMR:*** 125 MHz in $CDCl_3$

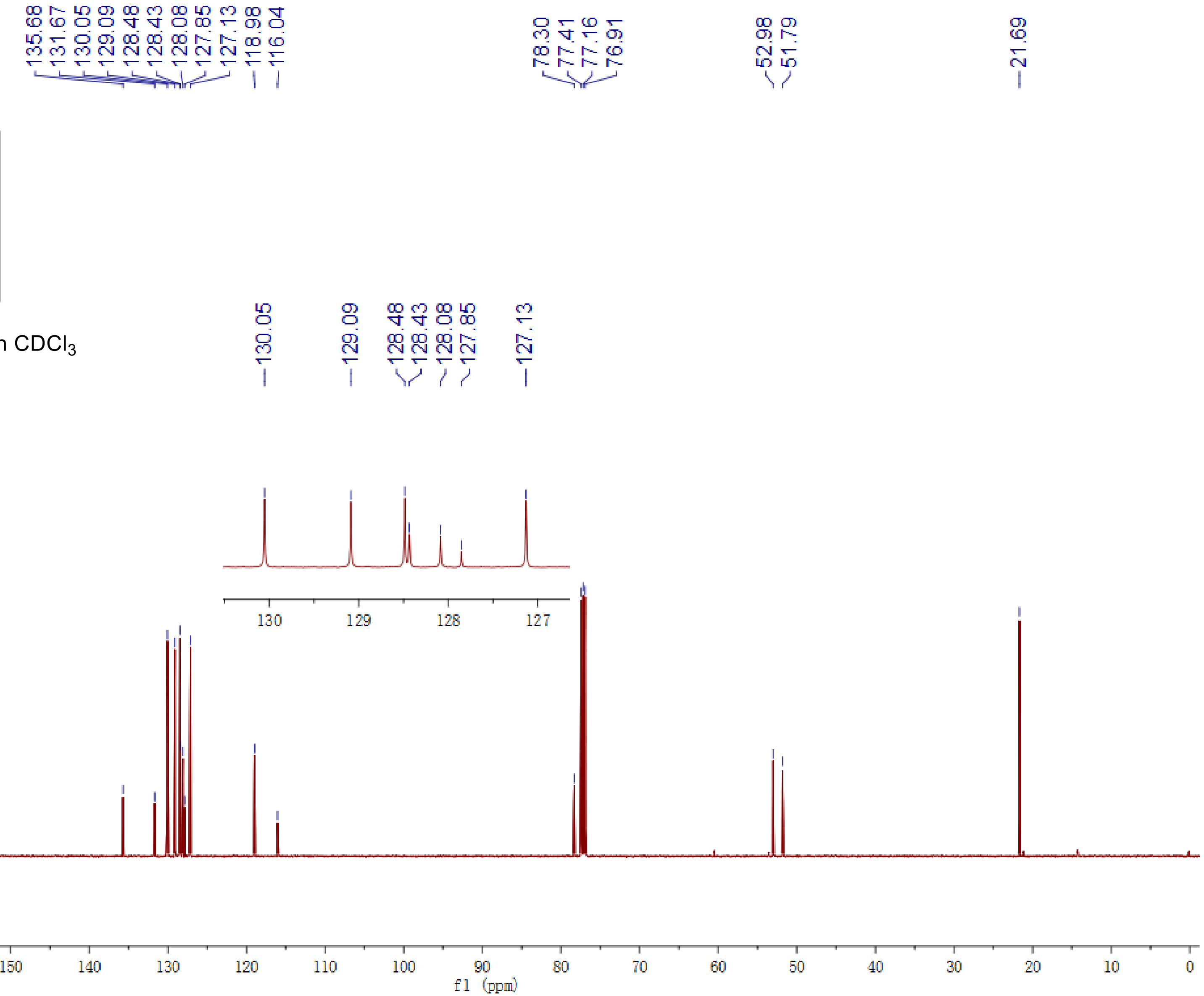

7.447
7.443
7.439
7.431
7.428
7.373
7.369
7.366
7.356
7.352
7.344
7.341
7.337
7.330
7.328
7.319
7.316
7.313
7.308
7.304
7.300
7.297
7.292
7.290
7.261
7.189
7.186
7.172
4.409
3.718
3.713
2.388
2.383
2.378
1.856

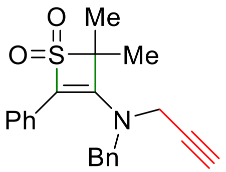

4c

^{1}H NMR: 500 MHz in $CDCl_3$

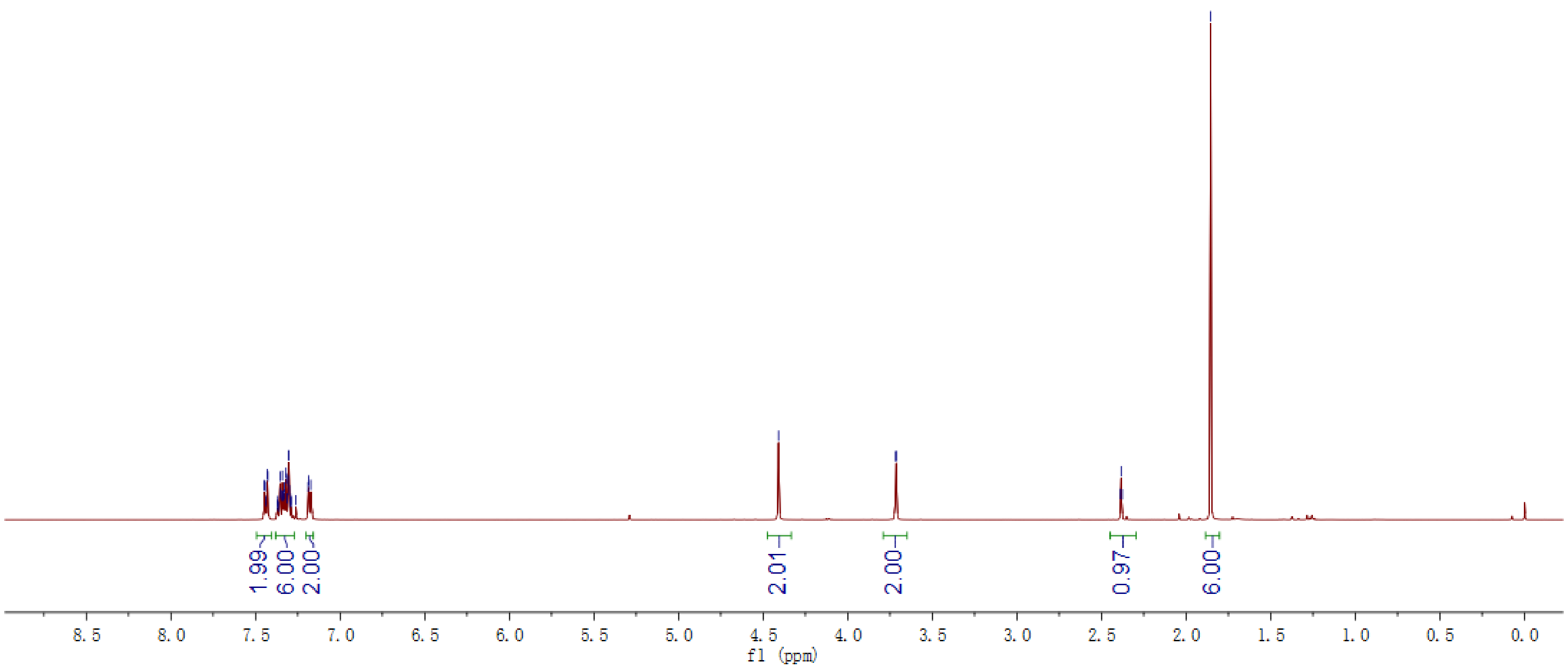

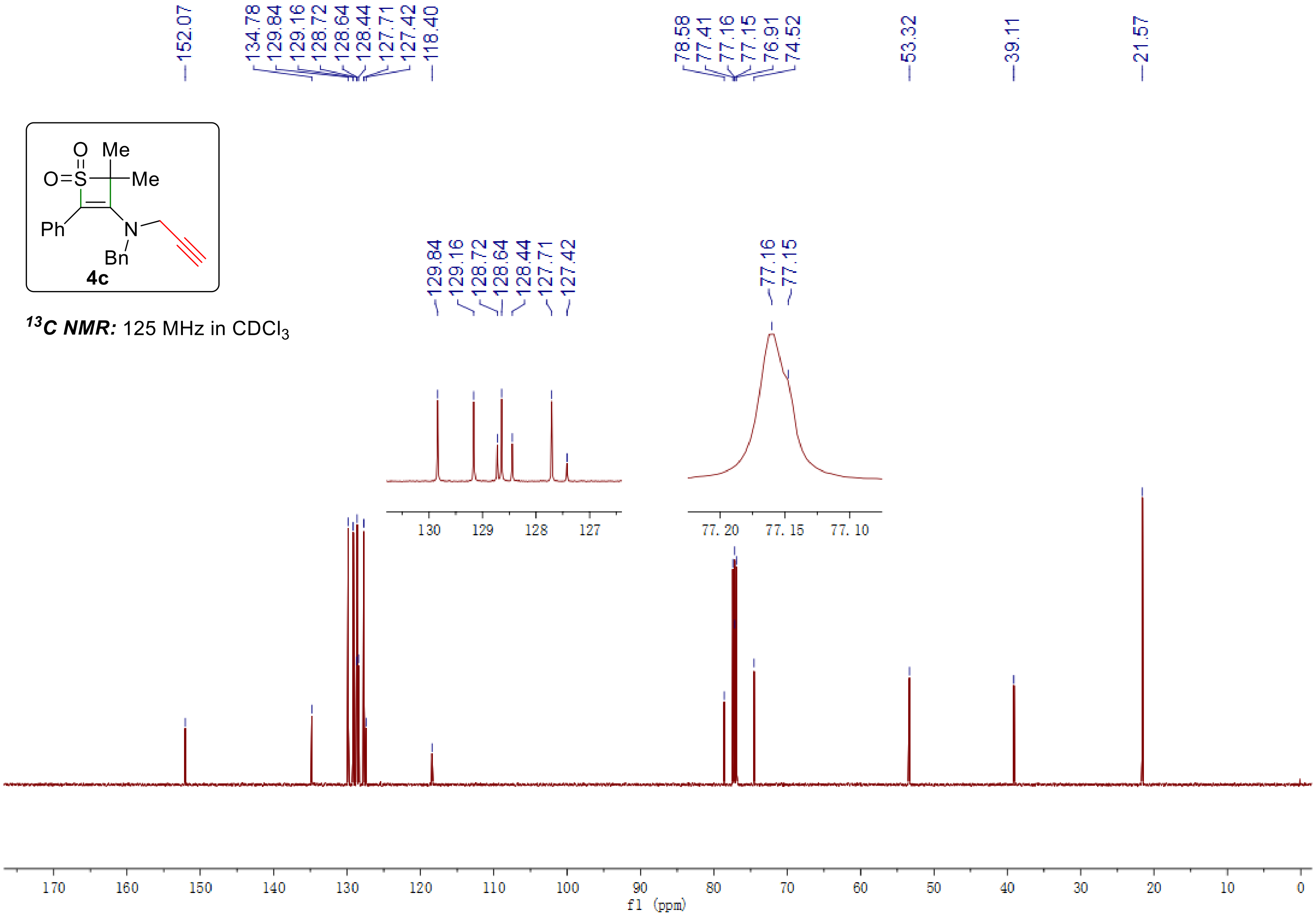
152.07
134.78
129.84
129.16
128.72
128.64
128.44
127.71
127.42
118.40
78.58
77.41
77.16
77.15
76.91
74.52
53.32
39.11
21.57
O
Me
O=S
Me
Ph
N
Bn
4c
13C NMR: 125 MHz in CDCl3
129.84
129.16
128.72
128.64
128.44
127.71
127.42
130
129
128
127
77.16
77.15
77.20
77.15
77.10
170
160
150
140
130
120
110
100
90
80
70
60
50
40
30
20
10
0
f1 (ppm)

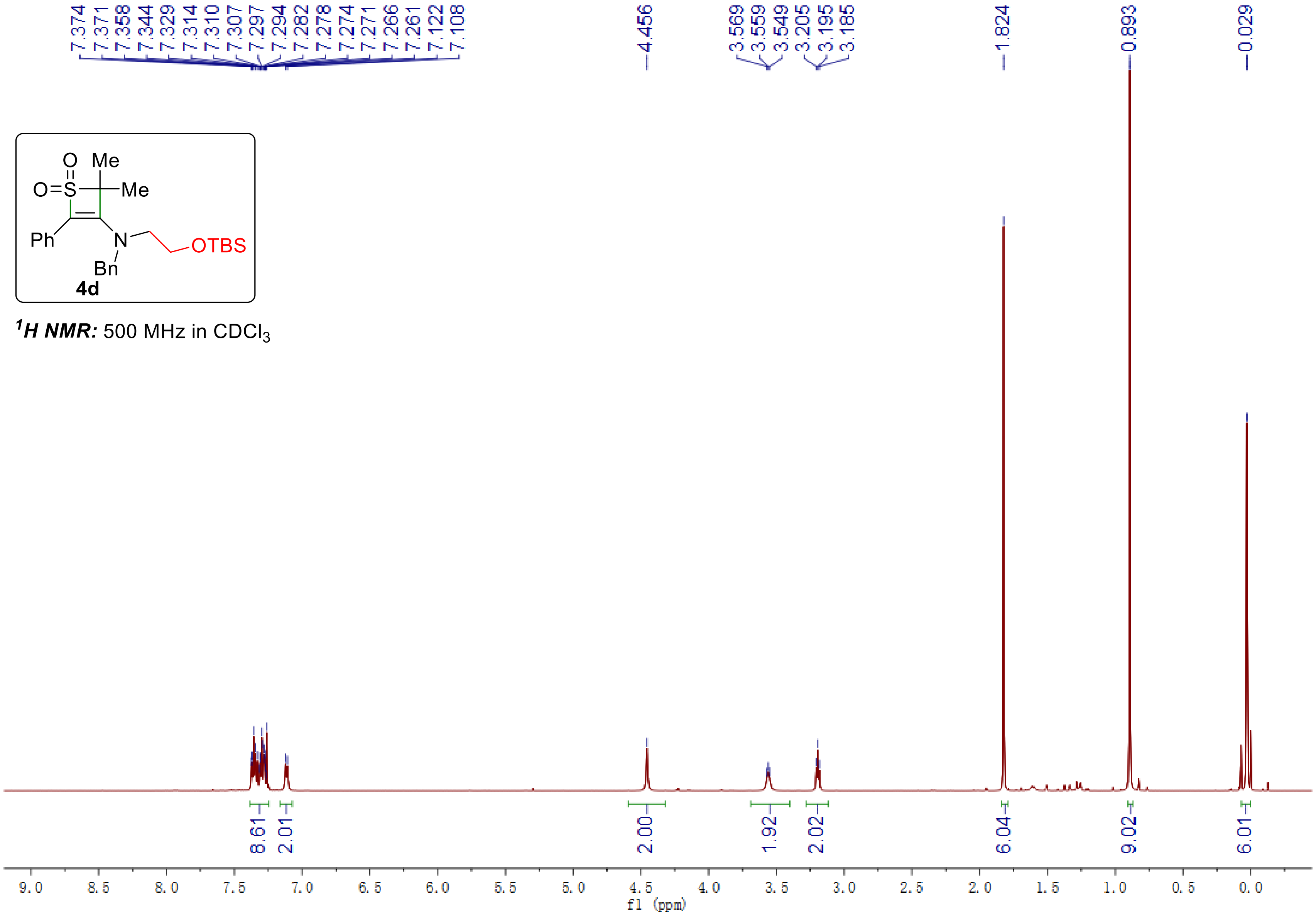
7.374
7.371
7.358
7.344
7.329
7.314
7.310
7.307
7.297
7.294
7.282
7.278
7.274
7.271
7.266
7.261
7.122
7.108
4.456
3.569
3.559
3.549
3.205
3.195
3.185
1.824
0.893
0.029
O
Me
O=S
Me
Ph
N
OTBS
Bn
4d
1H NMR: 500 MHz in CDCl3
8.61
2.01
2.00
1.92
2.02
6.04
9.02
6.01
9.0
8.5
8.0
7.5
7.0
6.5
6.0
5.5
5.0
4.5
4.0
3.5
3.0
2.5
2.0
1.5
1.0
0.5
0.0
f1 (ppm)

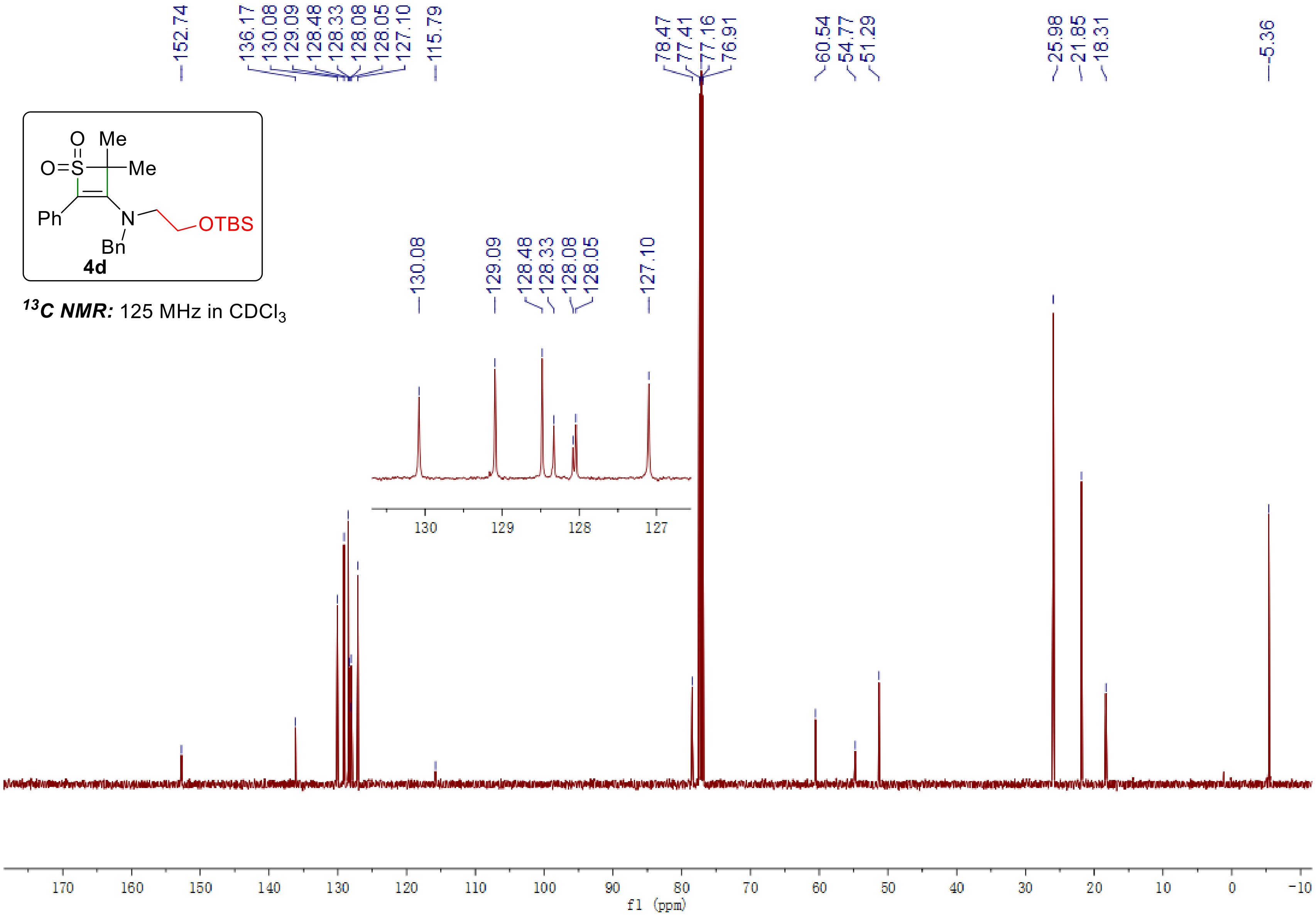

152.74
136.17
130.08
129.09
128.48
128.33
128.08
128.05
127.10
115.79
78.47
77.41
77.16
76.91
60.54
54.77
51.29
25.98
21.85
18.31
-5.36
O
Me
O=S
Me
Ph
N
OTBS
Bn
4d
^{13}C NMR: 125 MHz in $CDCl_3$
130.08
129.09
128.48
128.33
128.08
128.05
127.10
130
129
128
127
170
160
150
140
130
120
110
100
90
80
70
60
50
40
30
20
10
0
-10
f1 (ppm)

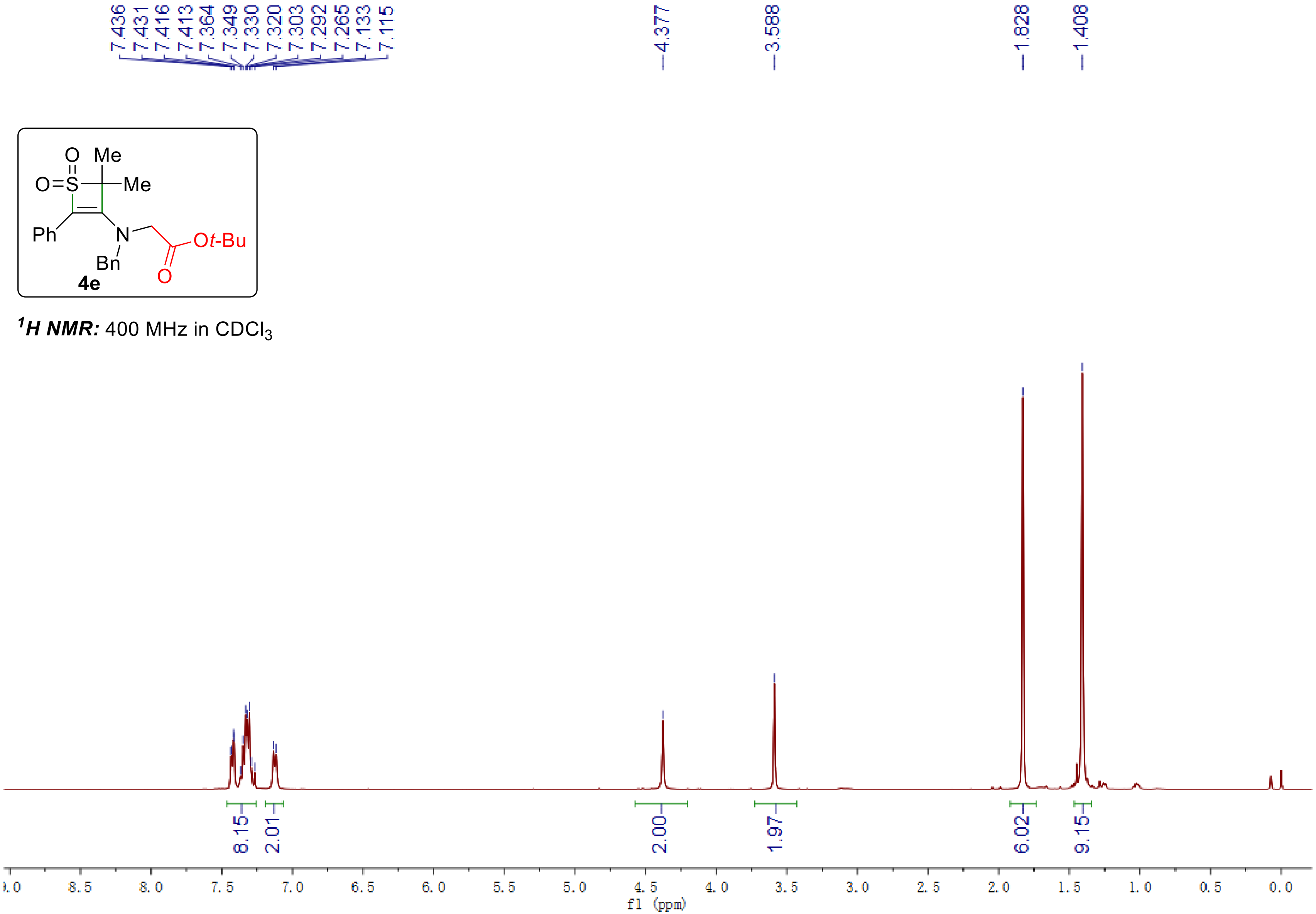

1H NMR: 400 MHz in $CDCl_3$

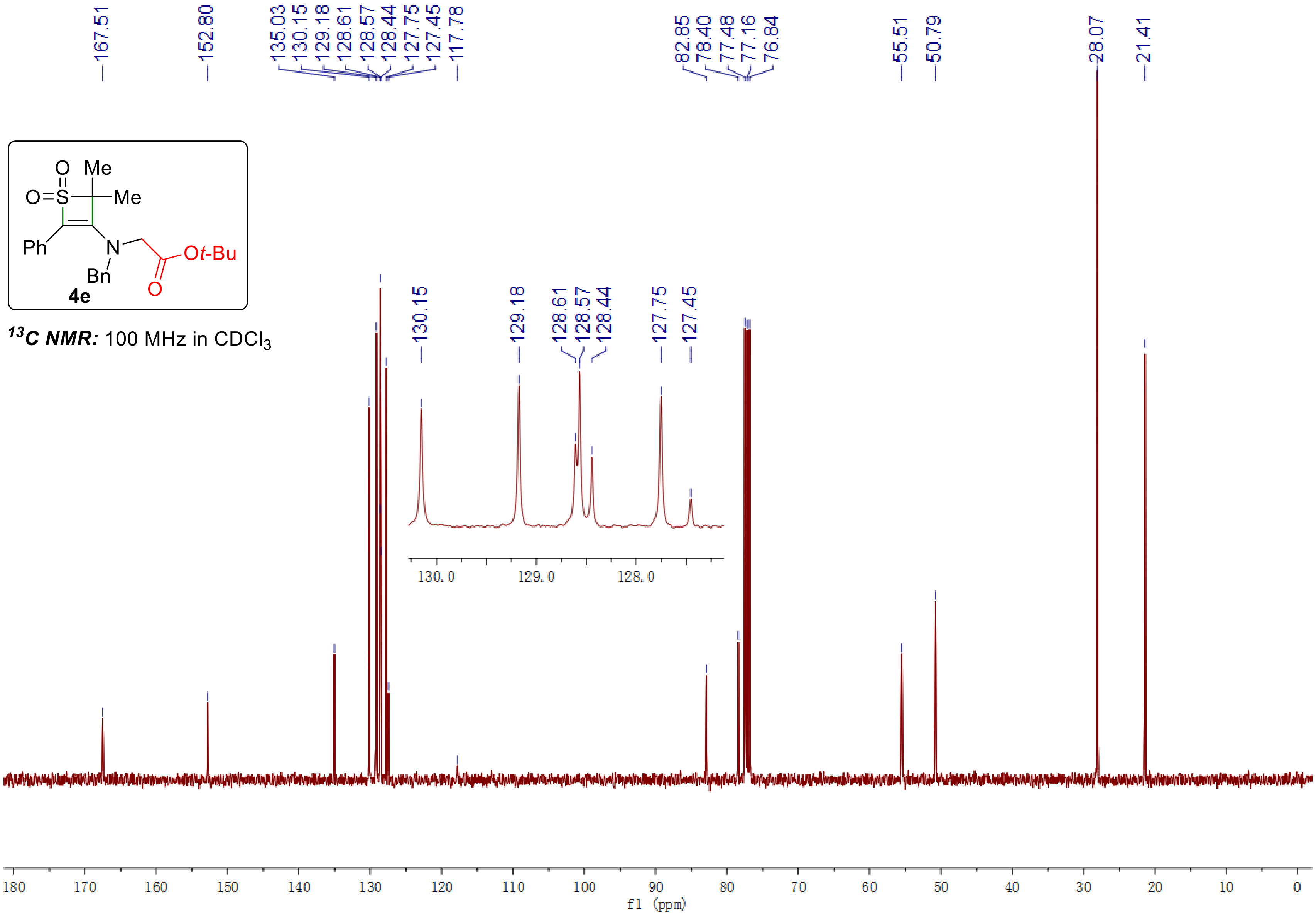
167.51
152.80
135.03
130.15
129.18
128.61
128.57
128.44
127.75
127.45
117.78
82.85
78.40
77.48
77.16
76.84
55.51
50.79
28.07
21.41
O
Me
O=S
Me
Ph
N
Ot-Bu
Bn
O
4e
13C NMR: 100 MHz in CDCl3
f1 (ppm)

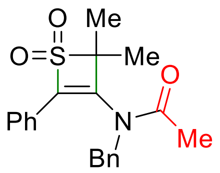

4f

^{1}H NMR: 400 MHz in $CDCl_3$

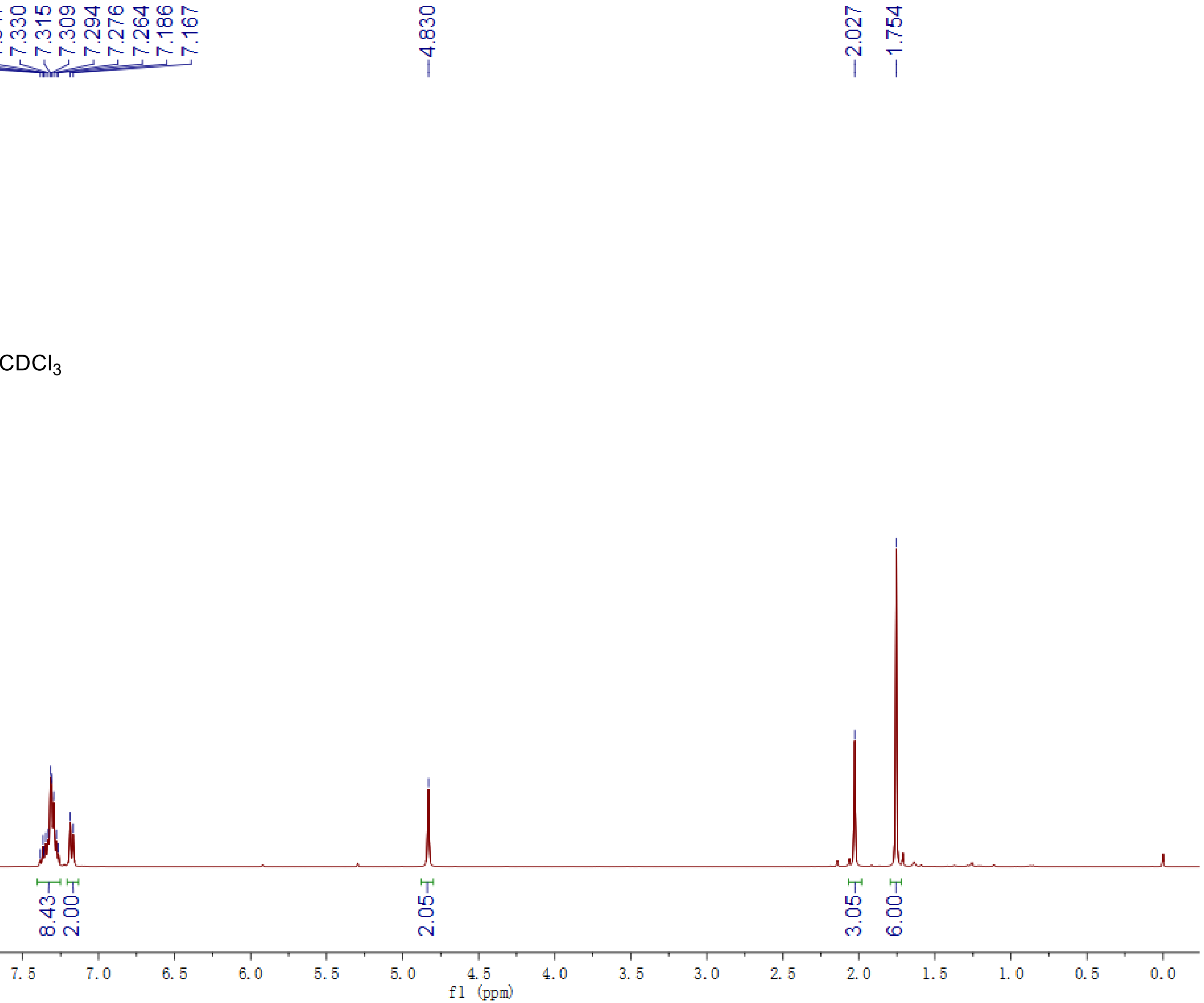

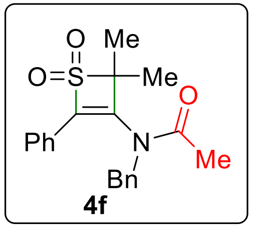

^{13}C NMR: 100 MHz in $CDCl_3$

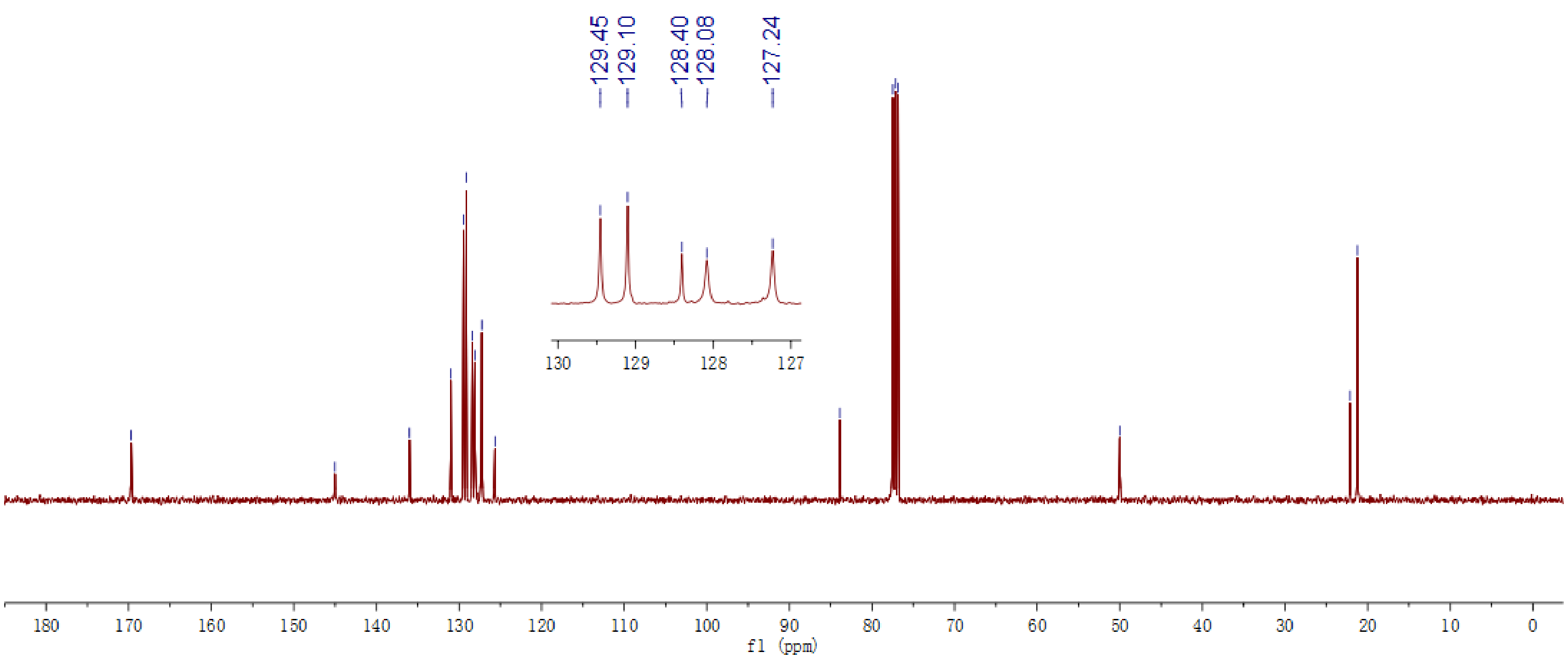

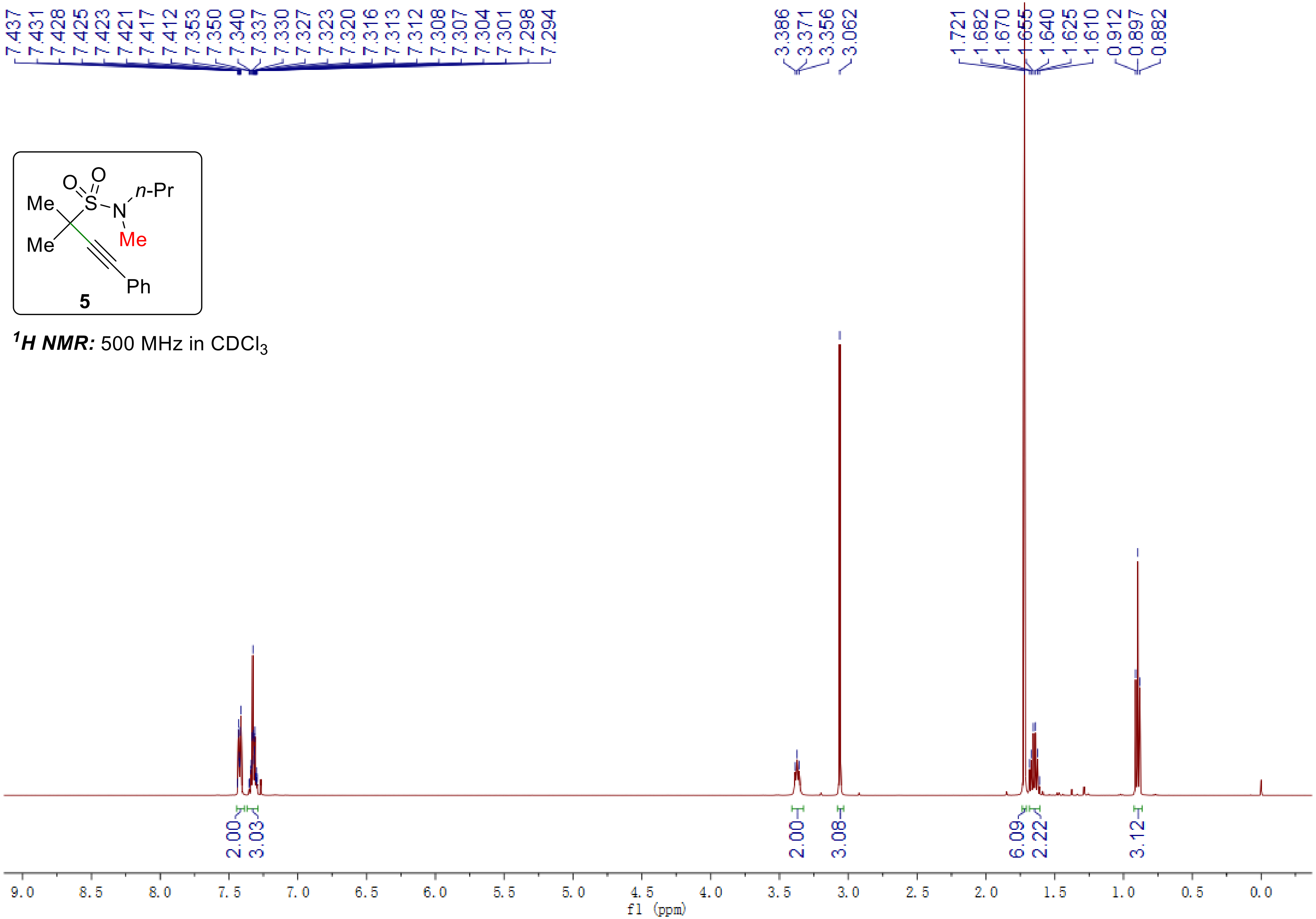
7.437 7.431 7.428 7.425 7.423 7.421 7.417 7.412 7.353 7.350 7.340 7.337 7.330 7.327 7.323 7.320 7.316 7.313 7.312 7.308 7.307 7.304 7.301 7.298 7.294
3.386 3.371 3.356 3.062
1.721 1.682 1.670 1.655 1.640 1.625 1.610 0.912 0.897 0.882
Me
Me
O
O
S
N
n-Pr
Me
Ph
5
1H NMR: 500 MHz in CDCl3
2.00 3.03
2.00
3.08
6.09 2.22
3.12
9.0 8.5 8.0 7.5 7.0 6.5 6.0 5.5 5.0 4.5 4.0 3.5 3.0 2.5 2.0 1.5 1.0 0.5 0.0
f1 (ppm)

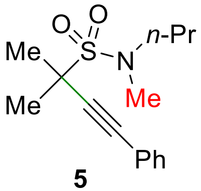

5

^{13}C NMR: 125 MHz in $CDCl_3$

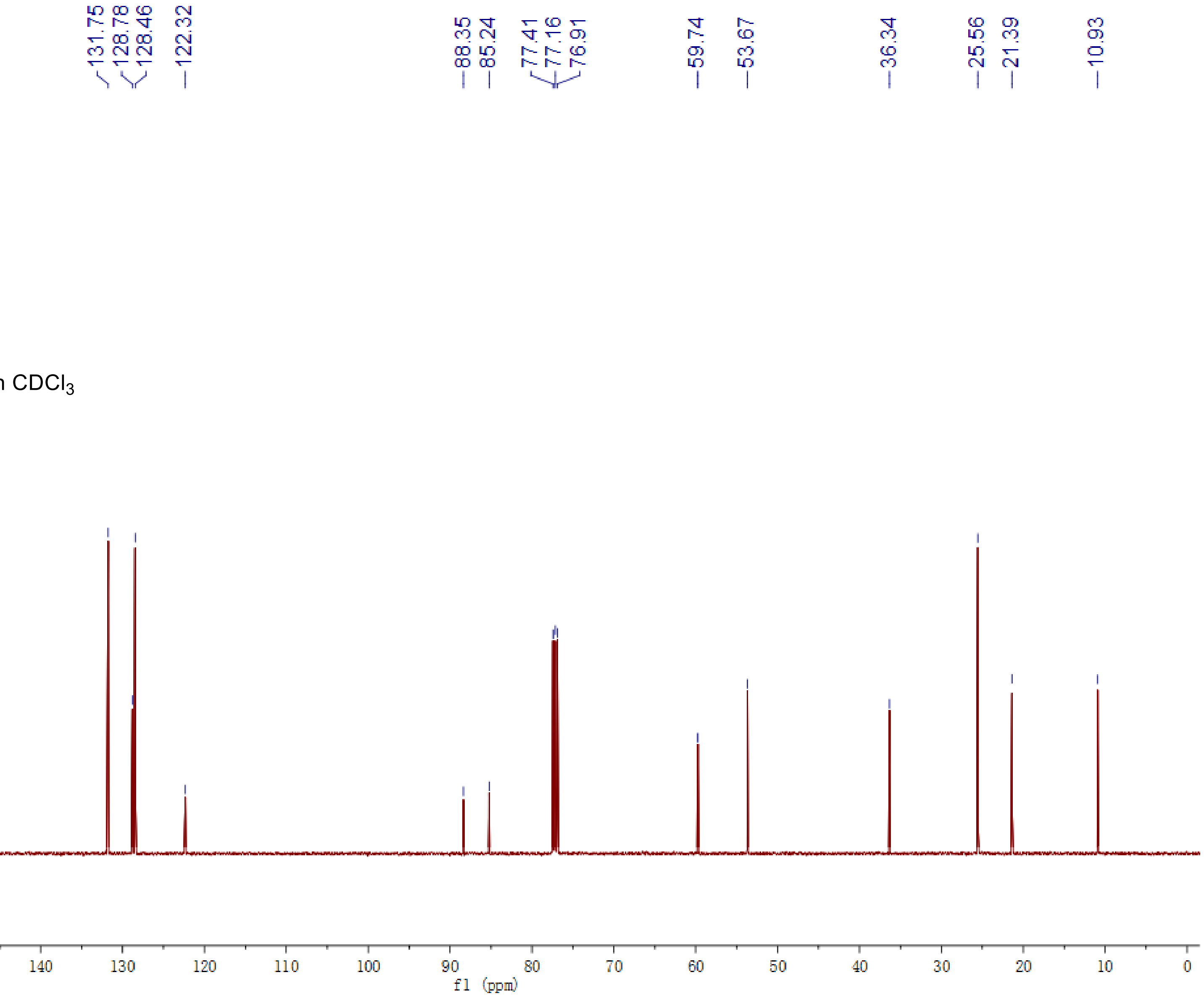

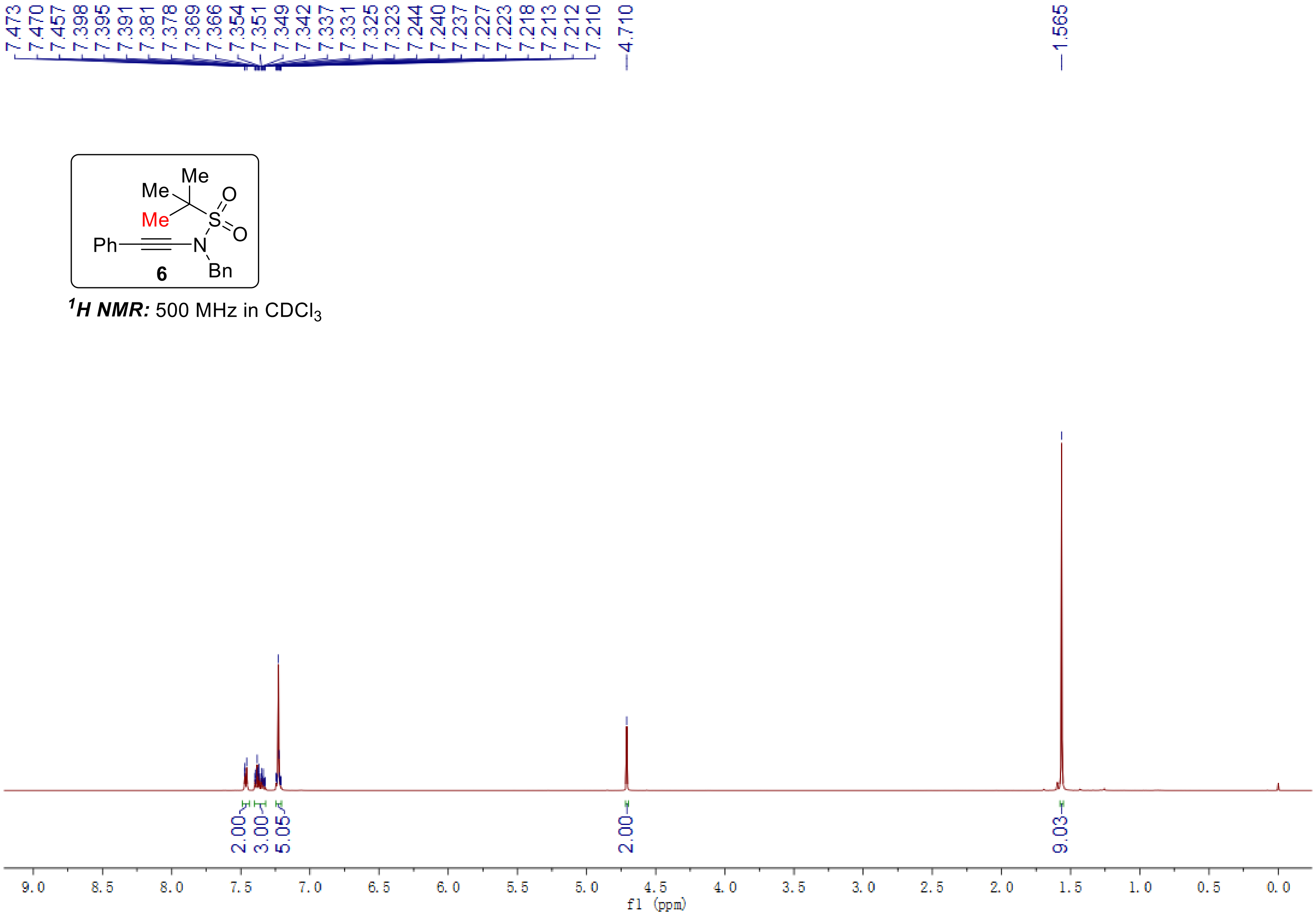
7.473
7.470
7.457
7.398
7.395
7.391
7.381
7.378
7.369
7.366
7.354
7.351
7.349
7.342
7.337
7.331
7.325
7.323
7.244
7.240
7.237
7.227
7.223
7.218
7.213
7.212
7.210
4.710
1.565
Me
Me
Me
O
S
O
Ph
N
Bn
6
¹H NMR: 500 MHz in CDCl3
2.00
3.00
5.05
2.00
9.03
9.0
8.5
8.0
7.5
7.0
6.5
6.0
5.5
5.0
4.5
4.0
3.5
3.0
2.5
2.0
1.5
1.0
0.5
0.0
f1 (ppm)

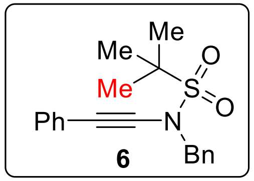

^{1}H NMR: 125 MHz in $CDCl_3$

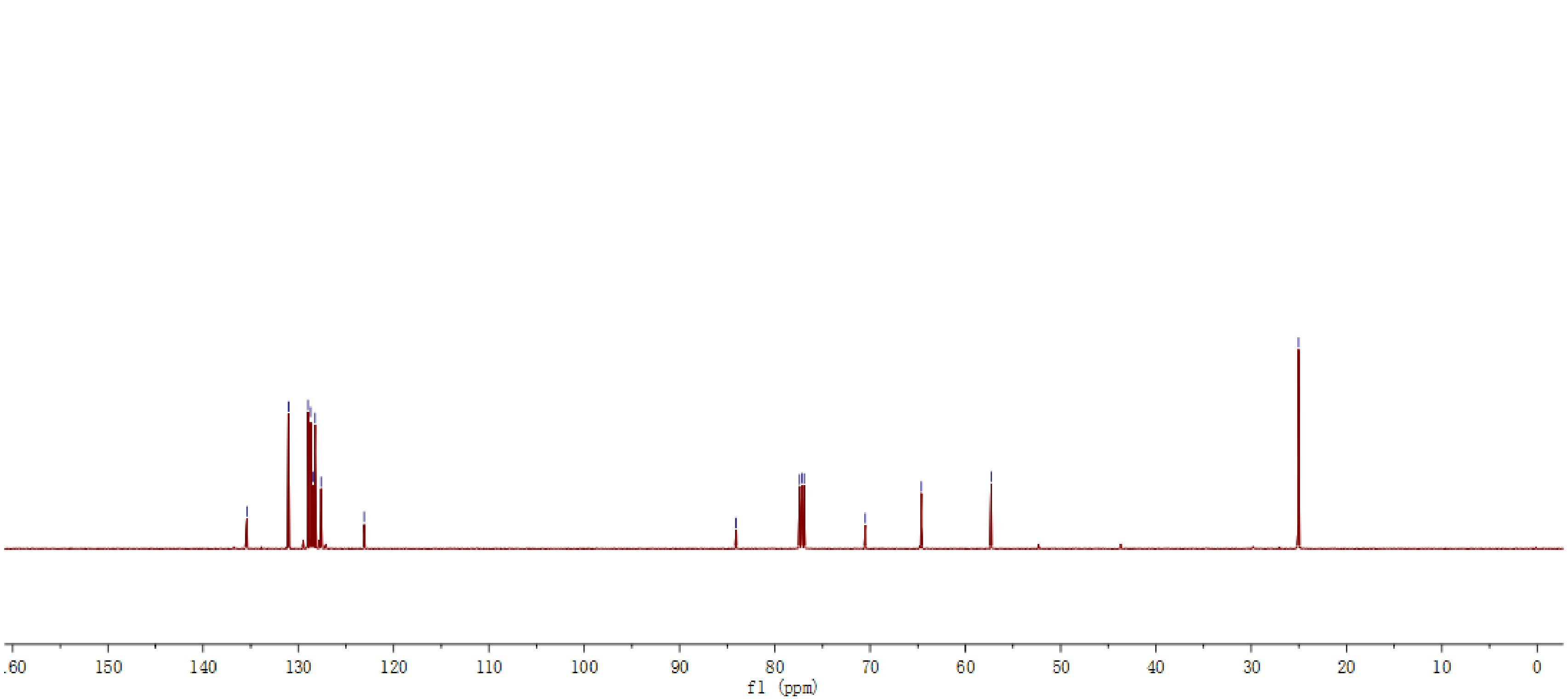

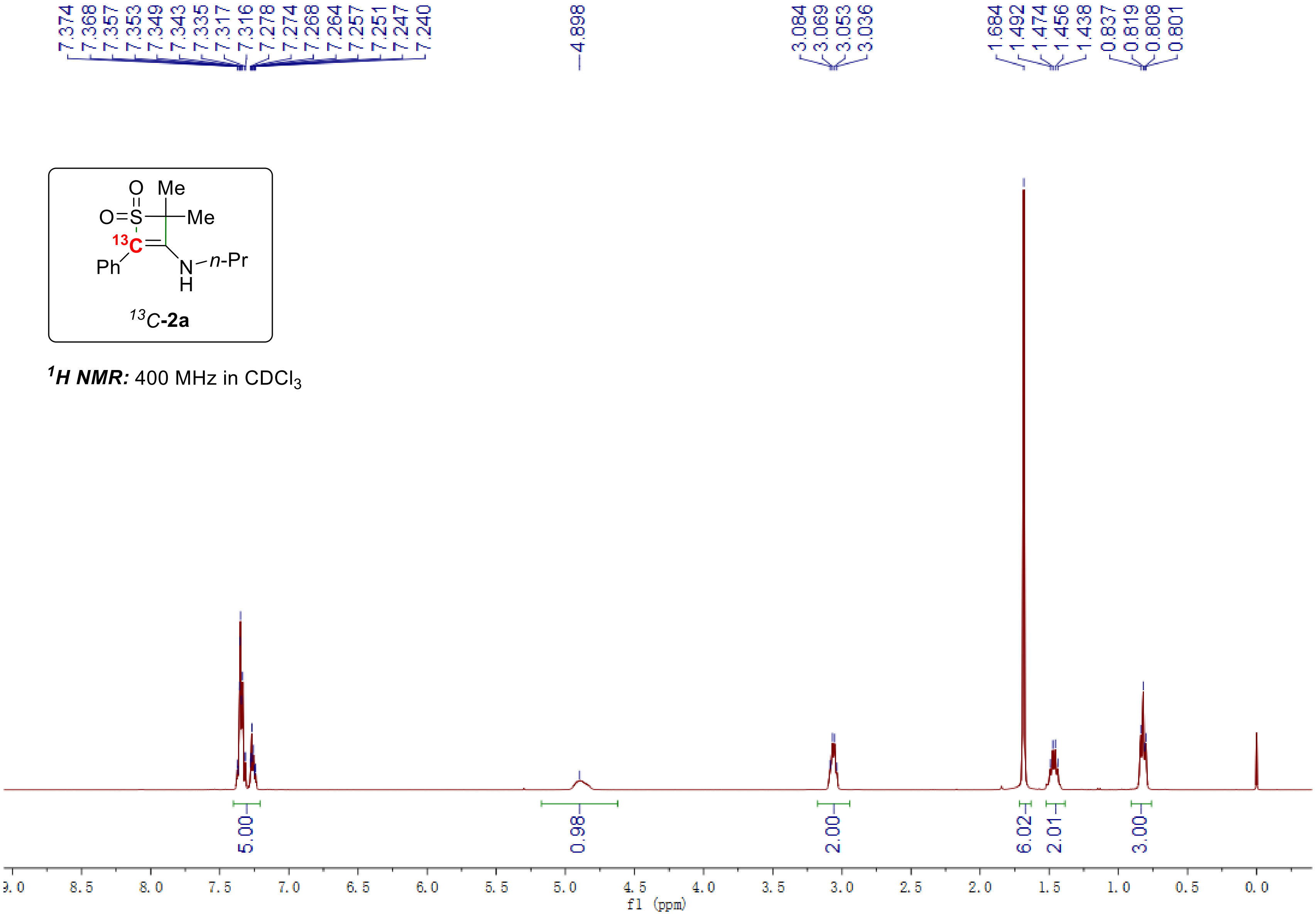

^{1}H NMR: 400 MHz in $CDCl_3$

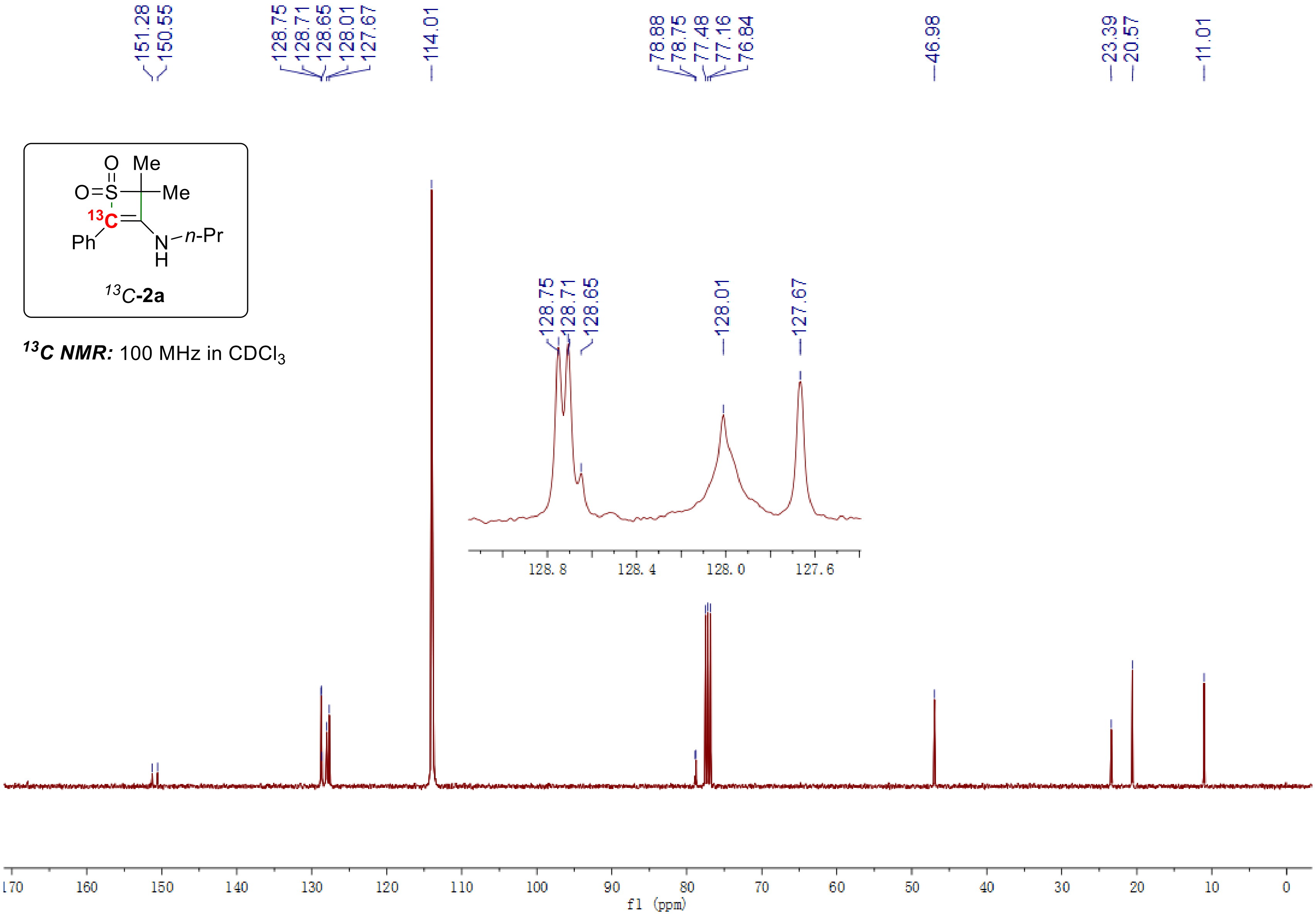
151.28
150.55
128.75
128.71
128.65
128.01
127.67
114.01
78.88
78.75
77.48
77.16
76.84
46.98
23.39
20.57
11.01
O Me
O=S—Me
13C
Ph
N—n-Pr
H
13C-2a
13C NMR: 100 MHz in CDCl3
128.75
128.71
128.65
128.01
127.67
128.8
128.4
128.0
127.6
170
160
150
140
130
120
110
100
90
80
70
60
50
40
30
20
10
0
f1 (ppm)

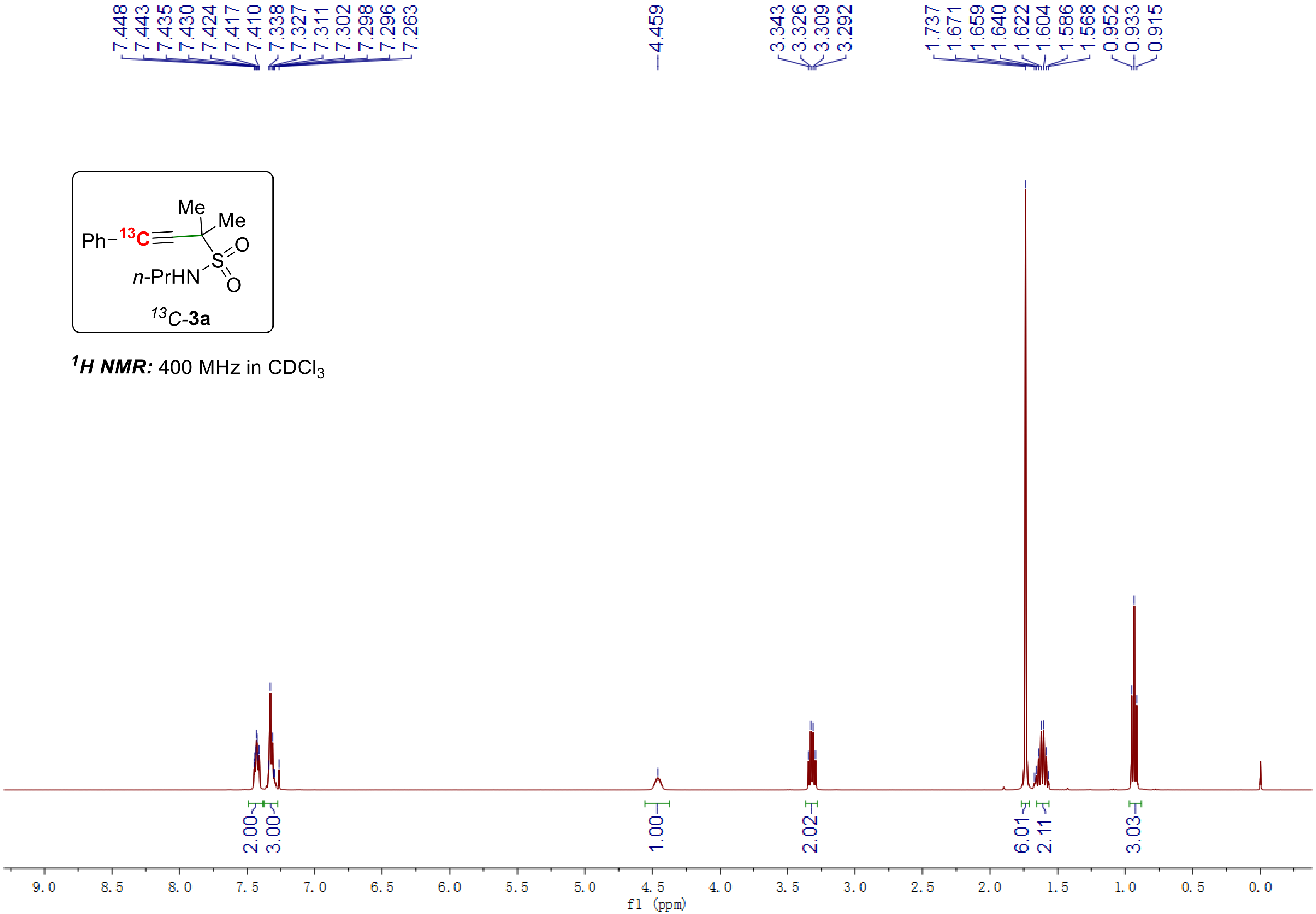
7.448
7.443
7.435
7.430
7.424
7.417
7.410
7.338
7.327
7.311
7.302
7.298
7.296
7.263
4.459
3.343
3.326
3.309
3.292
1.737
1.671
1.659
1.640
1.622
1.604
1.586
1.568
0.952
0.933
0.915
Me
Me
Ph–13C≡
S
O
O
n-PrHN
13C-3a
1H NMR: 400 MHz in CDCl3
2.00
3.00
1.00
2.02
6.01
2.11
3.03
9.0
8.5
8.0
7.5
7.0
6.5
6.0
5.5
5.0
4.5
4.0
3.5
3.0
2.5
2.0
1.5
1.0
0.5
0.0
f1 (ppm)

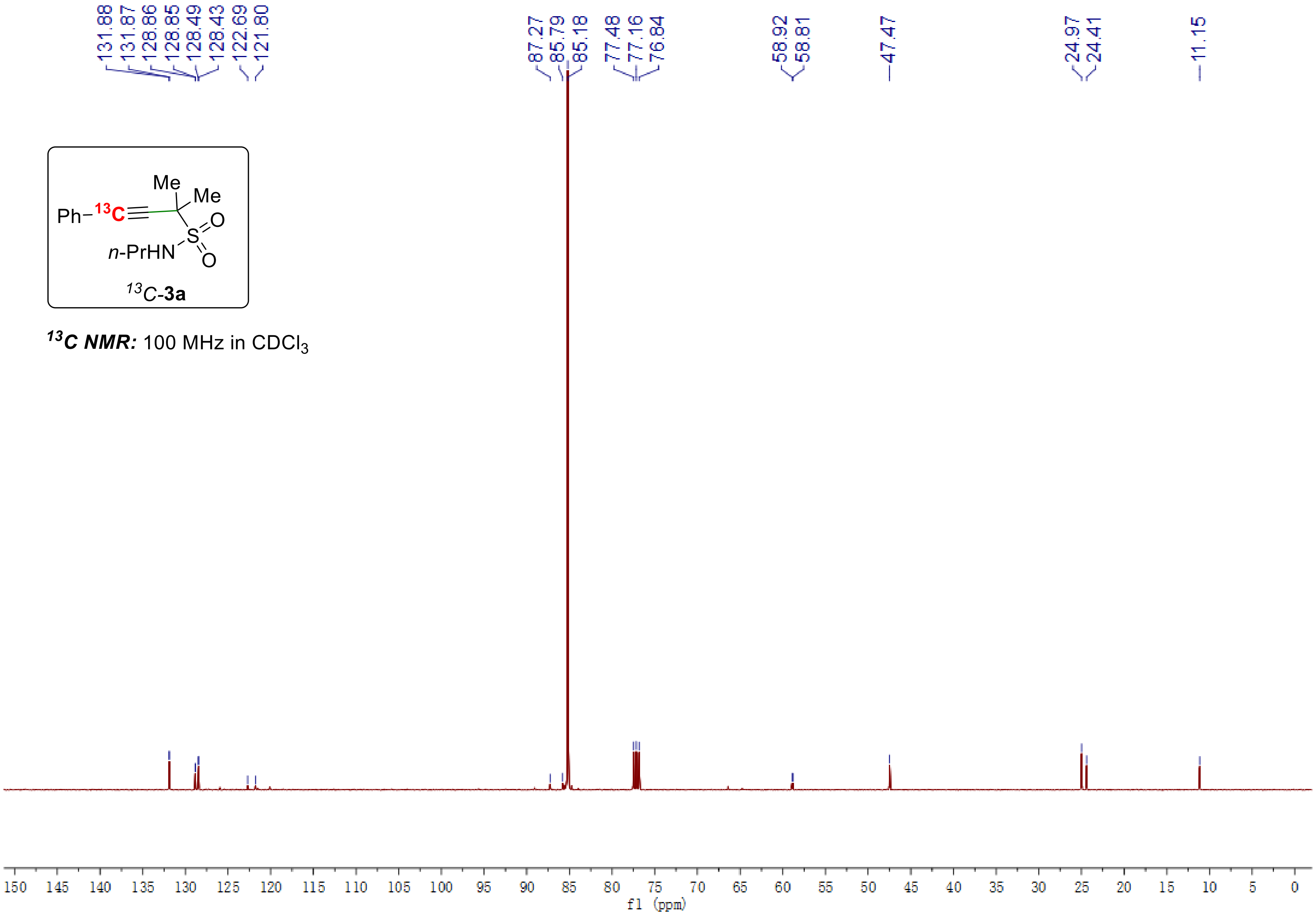
131.88
131.87
128.86
128.85
128.49
128.43
122.69
121.80
87.27
85.79
85.18
77.48
77.16
76.84
58.92
58.81
47.47
24.97
24.41
11.15
Me
Me
Ph–13C
O
S
n-PrHN
O
13C-3a
13C NMR: 100 MHz in CDCl3
150 145 140 135 130 125 120 115 110 105 100 95 90 85 80 75 70 65 60 55 50 45 40 35 30 25 20 15 10 5 0
f1 (ppm)

15. Supplementary References

1. Gottlieb, H. E., Kotlyar, V. & Nudelman, A. NMR chemical shifts of common laboratory solvents as trace impurities. *J. Org. Chem.* **62**, 7512-7515 (1997).
2. Zhang, X. J., Zhang, Y. S., Huang, J., Hsung, R. P., Kurtz, K. C. M., Oppenheimer, J., Petersen, M. E., Sagamanova, I. K., Shen, L. C. & Tracey, M. R. Copper(II)-catalyzed amidations of alkynyl bromides as a general synthesis of ynamides and *Z*-enamides. An intramolecular amidation for the synthesis of macrocyclic ynamides. *J. Org. Chem.* **71**, 4170-4177 (2006).
3. Zhou, B., Li, L., Zhu, X. -Q., Yan, J. -Z., Guo, Y. -L. & Ye, L. -W. Yttrium-catalyzed intramolecular hydroalkoxylation/Claisen rearrangement sequence: Efficient synthesis of medium-sized lactams. *Angew. Chem., Int. Ed.* **56**, 4015-4019 (2017).
4. An, W.-K., Han, M.-Y., Wang, C.-A., Yu, S.-M., Zhang, Y., Bai, S. & Wang, W. Insights into the asymmetric heterogeneous catalysis inporous organic polymers: constructing a TADDOL-embedded chiral catalyst for studying the structure-activity relationship. *Chem.-Eur. J.* **20**, 11019-11028 (2014).
5. Weisenburger, G. A., Faibish, N. C., Pippel, D. J. & Beak, P. Temperature- and electrophile-dependent stereocontrol: A structural and mechanistic investigation of (-)-sparteine-mediated asymmetric lithiation-substitution sequences of *N*-Boc-*N*-(*p*-methoxyphenyl) cinnamylamine. *J. Am. Chem. Soc.* **121**, 9522-9530 (1999).
6. Dixon, L. I., Carroll, M. A., Gregson, T. J., Ellames, G. J., Harrington, R. W. & Clegg, W. Unprecedented regiochemical control in the formation of aryl[1,2-a]imidazopyridines from alkynyliodonium salts: mechanistic insights. *Org. Biomol. Chem.* **11**, 5877-5884 (2013).
7. Sheldrick, G. M. Acta crystallographica section A: foundations of crystallography. **64**, 112 (2008).
8. Dolomanov, O. V., Bourhis, L. J., Gildea, R. J., Howard, J. A. K. & Puschmann, H. OLEX2: a complete structure solution, refinement and analysis program. J. Appl.Crystallogr. **42**, 339-341 (2009).
9. Zhao, Y. & Truhlar, D. G. The M06 suite of density functionals for main group thermochemistry, thermochemical kinetics, noncovalent interactions,

excited states, and transition elements: two new functionals and systematic testing of four M06-class functionals and 12 other functionals. *Theor. Chem. Acc.* **120**, 215−241 (2008).

10. Zhao, Y. & Truhlar, D. G. Density functionals with broad applicability in chemistry. *Acc. Chem. Res.* **41**, 157−167 (2008).

11. Frisch, M. J., Trucks, G. W., Schlegel, H. B., Scuseria, G. E., Robb, M. A., Cheeseman, J. R., Scalmani, G., Barone, V., Mennucci, B., Petersson, G. A., Nakatsuji, H., Caricato, M., Li, X.; Hratchian, H. P., Izmaylov, A. F., Bloino, J., Zheng, G., Sonnenberg, J. L., Hada, M., Ehara, M., Toyota, K., Fukuda, R., Hasegawa, J., Ishida, M., Nakajima, T., Honda, Y., Kitao, O., Nakai, H., Vreven, T., Montgomery, J. A., Jr, Peralta, J. E., Ogliaro, F., Bearpark, M., Heyd, J. J., Brothers, E., Kudin, K. N., Staroverov, V. N., Kobayashi, R., Normand, J., Raghavachari, K., Rendell, A., Burant, J. C., Iyengar, S. S., Tomasi, J., Cossi, M., Rega, N., Millam, J. M., Klene, M., Knox, J. E., Cross, J. B., Bakken, V., Adamo, C., Jaramillo, J., Gomperts, R., Stratmann, R. E., Yazyev, O., Austin, A. J., Cammi, R., Pomelli, C., Ochterski, J. W., Martin, R. L., Morokuma, K., Zakrzewski, V. G., Voth, G. A., Salvador, P., Dannenberg, J. J., Dapprich, S., Daniels, A. D., Farkas, Ö., Foresman, J. B., Ortiz, J. V., Cioslowski, J. & Fox, D. J. *Gaussian 09, revision A.01*; Gaussian, Inc.: Wallingford, CT, 2009.

12. Hariharan, P. C. & Pople, J. A. The influence of polarization functions on molecular orbital hydrogenation energies. *Theor. Chim. Acta* **28**, 213−222 (1973).

13. Hehre, W. J., Ditchfield, R. & Pople, J. A. Self-consistent molecular orbital methods. XII. Further extensions of Gaussian-type basis sets for use in molecular orbital studies of organic molecules. *J. Chem. Phys.* **56**, 2257−2261 (1972).
